# Supplementary material for: Total syntheses of (–)-macrocalyxoformins A and B and (–)-ludongnin C
Source: Nat Commun. 2024 Jul 18;15:6052. doi: 10.1038/s41467-024-50374-1 (PMC11258297; doi:10.1038/s41467-024-50374-1)
Supplement: Supplementary file 1 — Supplementary Information [file 41467_2024_50374_MOESM1_ESM.pdf]

Supplementary Information

Total Syntheses of (–)-Macrocalyxoformins A and B and (–)-Ludongnin C

Zichen Cao, Wenxuan Sun, Jingfu Zhang, Junming Zhuo, Shaoqiang Yang, Xiaocui Song, Yan Ma, Panrui Lu, Ting Han, Chao Li*

*Correspondence to: lichao@nibs.ac.cn

Table of Contents

1. Supplementary Methods 2

1.1 General Procedure for the Preparation of Redox-Active Esters (General procedure A) 3

1.2 General Procedure for the Reductive Decarboxylative Cyclization/Radical-Polar Crossover/*C*-acylation (General procedure B) 3

1.3 Cell culture and Method for IC_{50} Values Determination 5

2. Supplementary Discussion 6

2.1 Mechanistic Investigations 6

2.2 Preparations of Starting Materials in Table 1 14

2.3 Attempts for the Intermolecular Decarboxylative Giese reactions 17

2.4 Attempts for the Intramolecular Decarboxylative Giese reactions 19

3. Supplementary Notes 22

3.1 Experimental Procedure and Spectroscopic Data 22

3.2 Single Crystal X-ray Diffraction Data 78

3.3 NMR Spectra 88

4. Supplementary References 240

1. Supplementary Methods

NMR spectra were recorded on Varian 400 MHz or Bruker DRX−500 MHz instruments at ambient temperature with $CDCl_3$ as the solvent unless otherwise stated. Chemical shifts are reported in parts per million relatives to $CDCl_3$ (^1H, δ 7.26 for $CDCl_3$; ^{13}C, δ 77.16 for $CDCl_3$ unless otherwise stated). Data for ^1H NMR are reported as follows: chemical shift (ppm), multiplicity (s = singlet, d = doublet, t = triplet, q = quartet, m = multiplet, and br = broad), coupling constants (Hz), and integration. High resolution mass spectra (HRMS) were recorded at NIBS Metabolomics Center using an Agilent Tech. 6540 UHD Accurate−Mass Q-TOF LC/MS, or at Peking University Analysis Center using a Bruker Solarix XR FTMS. Optical rotations were recorded on an AUTOPOL III digital polarimeter at 589 nm and are recorded as $[\alpha]_D^{20}$ (concentration in grams/100 mL solvent). Chiral HPLC analysis was performed on an Agilent 1220 series. Analytical thin layer chromatography was performed using 0.25 mm silica gel 60−F plates. Column chromatography was performed using 300−400 mesh silica gel. Yields refer to chromatographically and spectroscopically pure materials, unless otherwise stated. All reactions were carried out under an inert argon atmosphere with dry solvents under anhydrous conditions unless otherwise stated. All glassware was dried in a drying oven before using. Dry CH_3CN (acetonitrile), CH_2Cl_2 (dichloromethane), Et_2O (diethyl ether), THF (tetrahydrofuran), and toluene were obtained by passing the previously degassed solvents through activated alumina columns. The Peking University X−ray Diffraction Laboratory collected and analyzed all X−ray diffraction data.

1.1 General Procedure for the Preparation of Redox-Active Esters (General procedure A)

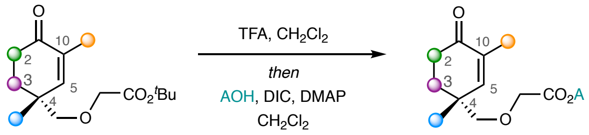

To a cooled (0 °C) solution of the *tert*-butyl ester (1.0 equiv) in CH_2Cl_2 (0.3 M) was added TFA (0.6 mL/mmol *tert*-butyl ester). The reaction mixture was warmed to room temperature and stirred for 3 h. The reaction mixture was concentrated directly, giving the carboxylic acid which was used directly for the next step without further purification.

Note: To rapidly remove TFA completely, the above crude product can be dissolved in toluene and concentrated under vaccum, this process can be repeated until no TFA could be detected by ^{19}F NMR.

N, N'-diisopropylcarbodiimide (DIC, 1.2 equiv) was added dropwise to a cooled (0 °C) mixture of above carboxylic acid (1.0 equiv), AOH (1.1 equiv), and DMAP (0.3 equiv) in anhydrous CH_2Cl_2 (0.2 M). After 4 h stirring at room temperature, the reaction mixture was directly concentrated under reduced pressure. Purification by flash column chromatography gave the RAE.

1.2 General Procedure for the Reductive Decarboxylative Cyclization/Radical-Polar Crossover/*C*-acylation (General procedure B)

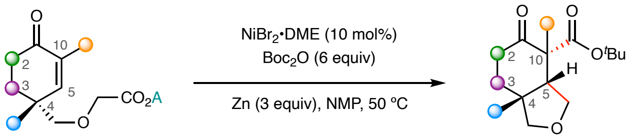

In a glovebox, to the mixture of the RAE (1.0 equiv), $NiBr_2$•DME (10 mol%), and Zn (3.0 equiv) in anhydrous *N*-mehtyl-2-pyrrolidone (NMP, 0.075 M) was added Boc_2O (6.0 equiv) at room temperature. The reaction mixture was moved out of the glove box and warmed to 50 °C. After 14 h stirring, the reaction mixture was filtered through a short pad of Celite, washed with EtOAc. The filtrate was washed with H_2O (2 times), brine (1 time) whereby the aqueous layers were back-extracted with EtOAc (3 times). The combined organic layers were dried over Na_2SO_4, filtrated, and concentrated under reduced pressure. Purification by column chromatography (silica gel) gave the product.

Purity and Sources of Reagents: $NiBr_2$•DME, min 97% (Strem®); Zinc, 99.99% metal basis, 600 mesh (Aladdin®); Boc_2O, 99% (J&K®); *N*-methyl-2-pyrrolidone (NMP), 99.5%, anhydrous (Sigma-Aldrich®).

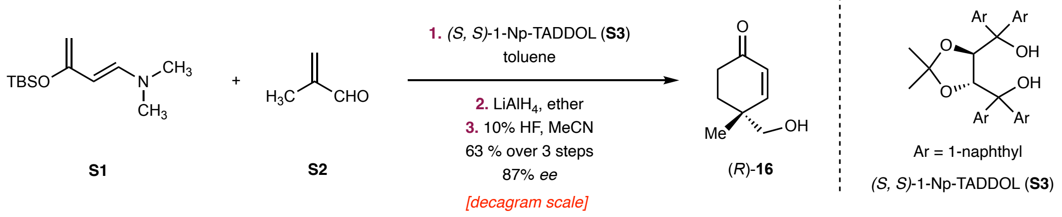

Supplementary Figure 1. Preparation of (*R*)-16

(*R*)-**16** was prepared according to Rawal's and Carreira's procedures with slight modifications [1,2].

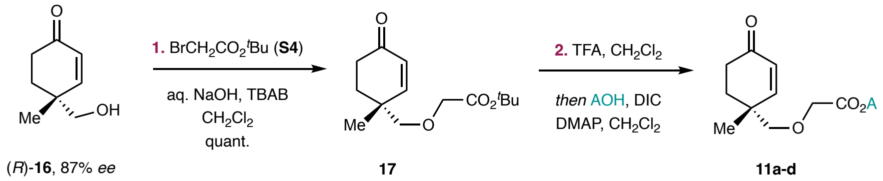

Entry	AOH	11	yield (from **17**)
1	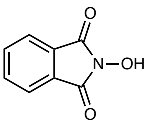 A^{1}OH	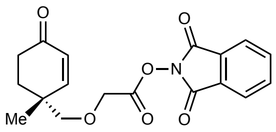 **11a**	83%
2	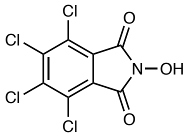 A^{2}OH	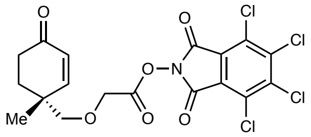 **11b**	76%
3	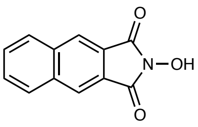 A^{3}OH	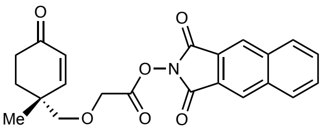 **11c**	69%
4	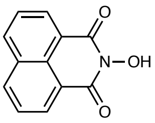 A^{4}OH	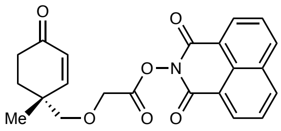 **11d**	81%

Supplementary Figure 2. Preparation of redox-active esters 11

1.3 Cell culture and Method for IC_{50} Values Determination

Cell culture

The human cell lines ME-180, U2OS, A549, HCT-116, SW756, HeLa, SiHa, HuH-7, and SK-CO-1 were obtained from Cell Resource Center, Peking Union Medical College (Beijing, China). All cell lines were confirmed to be mycoplasma free by PCR. Regular adherent cell culture methods were used to culture cells in tissue-culture incubators with 5% CO_2 at 37 °C. A549 was grown in RPMI-1640 medium with 10% fetal bovine serum (FBS) and 2 mM L-glutamine. SK-CO-1 was grown in MEM medium with 10% FBS and 2 mM L-glutamine. All other cells were grown in DMEM medium with 10% FBS and 2 mM L-glutamine.

Cell viability assay

Three thousand cells in 100 μL of medium were plated per well in 96-well flat clear bottom white polystyrene TC-treated microplates (Corning, Corning, USA). Then cells were dosed with a serial dilution of compounds with a D300e digital dispenser (Tecan, Männedorf, Switzerland). Cell survival was measured 72 h later using CellTiter-Glo luminescent cell viability assay kit (Promega, Madison, USA) according to the manufacturer's instructions. Luminescence was recorded by EnVison multimode plate reader (PerkinElmer, Waltham, USA). IC_{50} was determined with GraphPad Prism v8.0.2 using baseline correction (by normalizing to DMSO control), the asymmetric (four parameters) equation, and least squares fit.

2. Supplementary Discussion

2.1 Mechanistic Investigations

Experimental evidence for the radical-polar crossover process

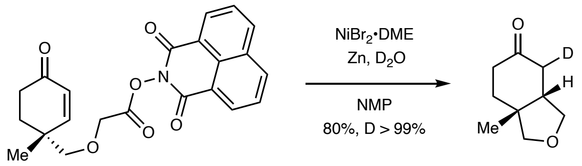

11d D-**18**

Supplementary Figure 3. Replacing Boc$_2$O with D_2O

In a glove box, to the mixture of **11d** (39.3 mg, 0.1 mmol, 1.0 equiv), Zn powder (20 mg, 0.3 mmol, 3.0 equiv), and NiBr$_2$•DME (10 mol%, 0.01 mmol, 3 mg) in NMP (1.3 mL) was added D_2O (36 μL, 2 mmol, 20.0 equiv). The reaction mixture was heated to 50 °C and stirred for 14 h. After cooling to room temperature, the reaction mixture was taken out of the glovebox, filtered through a short pad of Celite, washed with EtOAc (10 mL). The filtrate was washed with H_2O (10 mL×1), brine (10 mL×1) whereby the aqueous layers were back-extracted with EtOAc (10 mL×2). The combined organic layers were dried over Na_2SO_4, filtered, and concentrated under reduced pressure. Purification by column chromatography (silica gel, EtOAc:Petroleum ether, 1:2) gave D-**18** as amorphous solid (12.4 mg, 80%, > 99% D incorporation).

TLC (silica gel, 1:1, EtOAc: Petroleum ether): R_f = 0.5 ($KMnO_4$).

^{1}H NMR (400 MHz, $CDCl_3$): δ 4.02 (dd, J = 9.0, 7.3 Hz, 1H), 3.72 (d, J = 8.6 Hz, 1H), 3.58 (d, J = 8.6 Hz, 1H), 3.51 (ddd, J = 9.0, 6.7, 1.5 Hz, 1H), 2.39 – 2.34 (m, 2H), 2.31 – 2.24 (m, 2H), 2.02 – 1.95 (m, 1H), 1.83 – 1.76 (m, 1H), 1.23 (s, 3H).

^{13}C NMR (101 MHz, $CDCl_3$): δ 212.2, 80.4, 73.7, 45.5, 40.5, 40.2 (m, CHD) 36.5, 32.0, 24.4.

HRMS (EI, m/z): cacl'd for $C_9H_{13}DO_2$ [M]: 155.1057; found 155.1050.

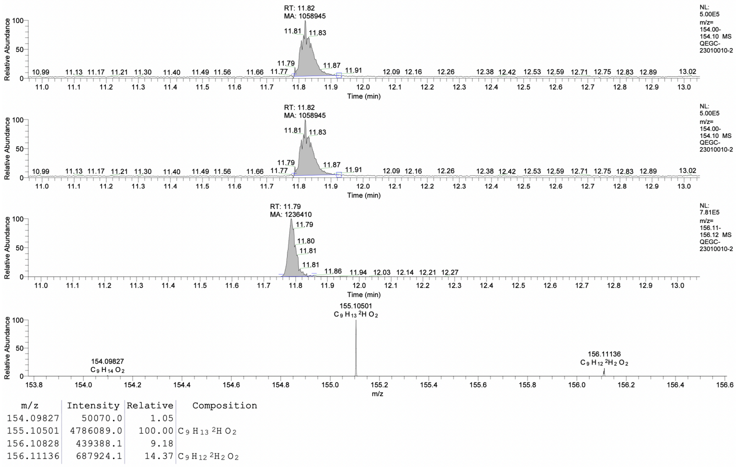

Supplementary Figure 4. The deuterium incorporation was determined by HRMS

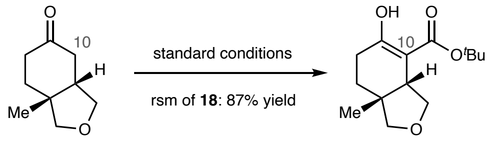

18 **9d**, 0% yield

Supplementary Figure 5. 18 was used as the starting material instead of RAE 11d.

On 0.15 mmol scale, **General procedure B** was followed with **18** as the starting material instead of the RAE. The yield of **9d** was determined by LC/MS.

*Note that **18** was prepared in 80% yield according to the procedure for the preparation of D-**18** (see above) using H_2O instead of D_2O.*

TLC (silica gel, 1:1, EtOAc: Petroleum ether): $R_f = 0.5$ ($KMnO_4$).

^{1}H NMR (400 MHz, $CDCl_3$): δ 4.01 (dd, J = 9.0, 7.4 Hz, 1H), 3.71 (d, J = 8.6 Hz, 1H), 3.57 (d, J = 8.6 Hz, 1H), 3.50 (dd, J = 9.0, 6.5 Hz, 1H), 2.50 – 2.45 (m, 1H), 2.38 – 2.28 (m, 3H), 2.28 – 2.21 (m, 1H), 2.01 – 1.94 (m, 1H), 1.82 – 1.75 (m, 1H), 1.22 (s, 3H).

^{13}C NMR (101 MHz, $CDCl_3$): δ 211.8, 80.2, 73.6, 45.5, 40.4, 40.3, 36.3, 31.9, 24.3.

HRMS (ESI, m/z): cacl'd for $C_9H_{15}O_2$ $[M+H]^+$: 155.1067, found: 155.1063.

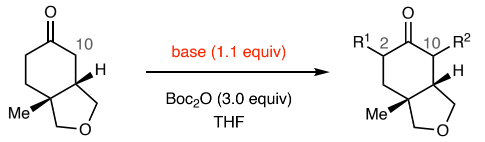

18

R^1 or R^2 = $CO_2{}^tBu$
0-3% yield

entry	base	yield[a]
1	LDA	3%
2	LiHMDS	trace
3	NaHMDS	N.D.
4	KHMDS	trace

[a]Yields were determined by LC/MS

Supplementary Figure 6. Attempts to acylation of 18 with Boc_2O

To a solution of **18** (23 mg, 0.15 mmol, 1.0 equiv) in THF (2 mL) was added the base (1.1 equiv) in above table dropwise at –78 °C. After 1 h stirring at –78 °C, Boc_2O (192 mg, 0.90 mmol, 6.0 equiv) was added. The resulting reaction mixture was warmed to room temperature. After overnight stirring, the reaction mixture was quenched with sat. aq. NH_4Cl (5 mL) and extracted with EtOAc (5 mL×3). The yield of products was determined by LC/MS.

Tracing the origin of the C10 ester group

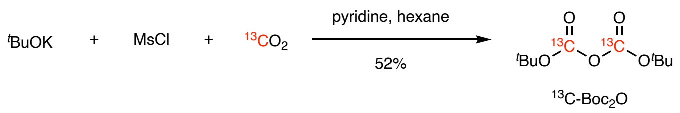

Supplementary Figure 7. Preparation of ^{13}C labeled Boc$_2$O[3]

The two-neck round-bottom flask charged with ^{t}BuOK (4.5 g, 40.0 mmol, 1.0 equiv) was evacuated and backfilled with argon for 3 times before the addition of hexane (48 mL). $^{13}CO_2$ (>99% ^{13}C) was bubbled into above suspension at 0 °C for 1 h. Then pyridine (64 μL, 0.8 mmol, 0.02 equiv) was added followed by the dropwise addition of MsCl (1.58 mL, 20.0 mmol, 0.5 equiv). After 2.5 h stirring at 0 °C, the reaction was quenched with 5% aq. H_2SO_4 (20 mL) and stirred at room temperature for 30 min. The organic phase was separated, washed with sat. aq. $NaHCO_3$ and concentrated under reduced pressure. Purification by column chromatography (silica gel, Petroleum ether:EtOAc, 30:1) provided ^{13}C-Boc$_2$O as colorless oil (2.28 g, 52% yield, >99% ^{13}C incorporation).

^{1}H NMR (400 MHz, $CDCl_3$): δ 1.52 (s, 9H).

^{13}C NMR (101 MHz, $CDCl_3$): δ 146.7, 85.2, 27.4.

HRMS (ESI, m/z): cacl'd for $C_8{}^{13}C_2H_{12}NO_5$ $[M+NH_4]^+$: 238.1559; found 238.1587.

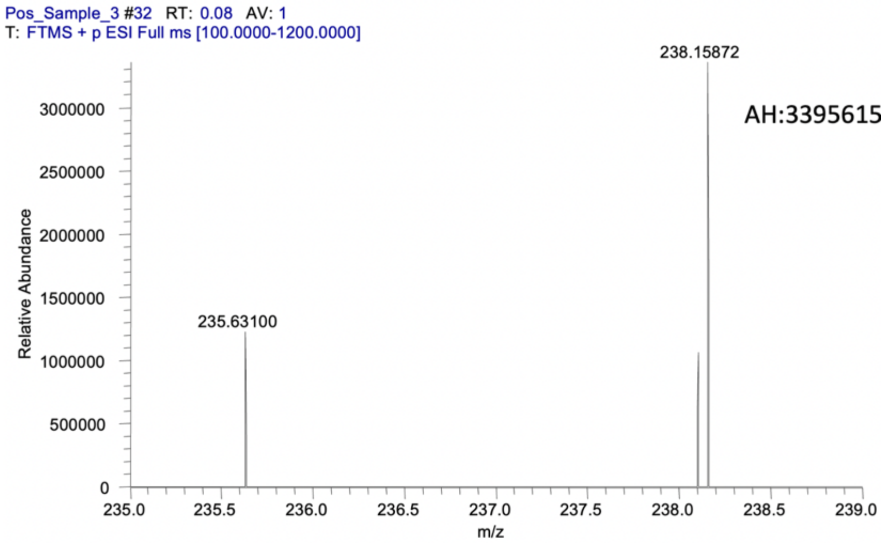

Supplementary Figure 8. The ^{13}C incorporation was determined by HRMS

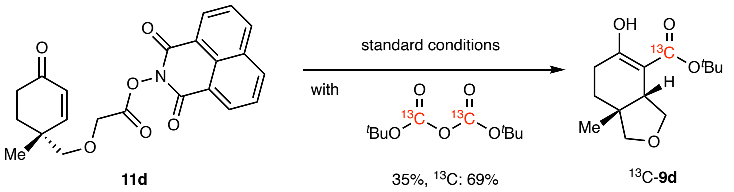

Supplementary Figure 9. Replacing Boc_2O with ^{13}C-Boc_2O

On 0.1 mmol scale, **General procedure B** was followed with **11d** and ^{13}C-Boc_2O. Purification by column chromatography (silica gel, Petroleum ether:EtOAc, 20:1) afforded ^{13}C-**9d** (9 mg, 35% yield, 69% ^{13}C incorporation)

TLC (silica gel, EtOAc: Petroleum ether, 1:5): R_f = 0.4 (UV).

^{1}H NMR (400 MHz, $CDCl_3$): δ 12.44 (s, 1H), 4.22 (t, *J* = 8.4 Hz, 1H), 3.66 – 3.54 (m, 2H), 3.50 (t, *J* = 8.3 Hz, 1H), 2.70 – 2.61 (m, 1H), 2.43 – 2.21 (m, 2H), 1.85 (ddd, *J* = 13.4, 11.5, 5.9 Hz, 1H), 1.49 (s, 9H), 1.47 – 1.43 (m, 1H), 1.06 (s, 3H).

^{13}C NMR (101 MHz, $CDCl_3$): δ 172.7, 171.4, 98.9, 81.6, 80.1, 75.4, 44.3, 40.1, 28.7, 28.5, 26.5, 21.8.

HRMS (ESI, m/z): ^{13}C-9d: calc'd for $C_{13}{}^{13}CH_{21}O_4$ $[M–H]^-$: 254.1479, found 254.1480.

9d: calc'd for $C_{14}H_{21}O_4$ $[M–H]^-$: 253.1445, found 253.1447.

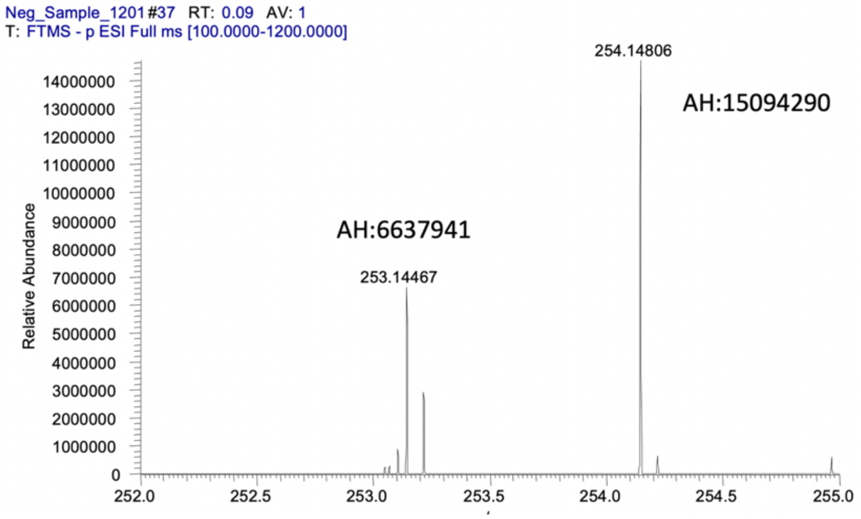

Supplementary Figure 10. The ^{13}C incorporation was determined by HRMS

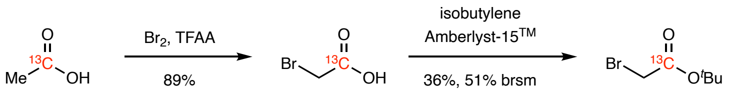

S5 **S6** 13C-**S4**

Supplementary Figure 11. Preparation of ^{13}C-labled *tert*-butyl bromoacetate[4,5]

S6 was prepared according to the reported procedure[4] in 89% yield.

^{1}H NMR (400 MHz, CDCl$_3$): δ 10.38 (br, 1H), 3.90 (d, *J* = 4.7 Hz, 2H).

^{13}C NMR (101 MHz, CDCl$_3$): δ 173.6, 25.3 (d, *J* = 62.8 Hz, 1C).

HRMS (ESI, m/z): cacl'd for $^{13}CCH_2BrO_2$ [M–H]$^-$: 137.9277, found: 137.9270.

^{13}C-**S4** was prepared according to the reported procedure[5] in 36% yield (51% brsm).

^{1}H NMR (400 MHz, CDCl$_3$): δ 3.75 (d, *J* = 4.6 Hz, 2H), 1.48 (s, 9H).

^{13}C NMR (101 MHz, CDCl$_3$): δ 166.2, 82.9, 27.8, 27.7 (d, *J* = 65.7 Hz, 1C).

HRMS (ESI, m/z): cacl'd for $C_5{}^{13}CH_{12}BrO_2$ [M+H]$^+$: 196.0049, found: 195.9932.

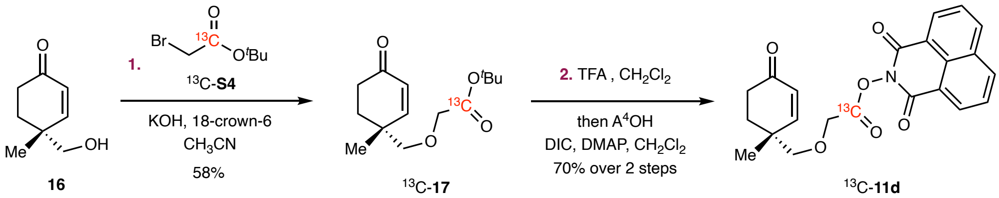

16 ^{13}C-**17** ^{13}C-**11d**

Supplementary Figure 12. Preparation of ^{13}C-labled substrates ^{13}C-11d

To a solution of **16** (546 mg, 3.9 mmol, 1.0 equiv) in CH_3CN (20 mL) was added KOH (262 mg, 4.7 mmol, 1.2 equiv), 18-crown-6 (1.24 g, 4.7 mmol, 1.2 equiv), and ^{13}C-**S4** (760 mg, 3.9 mmol, 1.0 equiv) successively. After 5 h stirring at room temperature, the reaction mixture was concentrated (to *ca.* 5 mL), diluted with H_2O (50 mL), and extracted with CH_2Cl_2 (30 mL×3). The combined organic phases were washed with brine successively, dried over Na_2SO_4, filtrated, and concentrated under reduced pressure. Purification by column chromatography (silica gel, Petroleum ether:EtOAc, 6:1) gave ^{13}C-**17** (577 mg, 58%) as colorless oil.

TLC (silica gel, 1:4, EtOAc: Petroleum ether): R_f = 0.6 (UV).

^{1}H NMR (400 MHz, CDCl$_3$): δ 6.79 (dd, *J* = 10.2, 0.8 Hz, 1H), 5.96 (d, *J* = 10.2 Hz, 1H), 3.97 (dd, *J* = 4.3, 0.6 Hz, 2H), 3.49 – 3.36 (m, 2H), 2.57 – 2.43 (m, 2H), 2.17 (ddd, *J* = 13.5, 9.2, 6.2 Hz, 1H), 1.77 (dddd, *J* = 13.5, 6.7, 5.5, 1.2 Hz, 1H), 1.48 (s, 9H), 1.19 (s, 3H).

^{13}C NMR (101 MHz, CDCl$_3$): δ 199.8, 169.8, 156.1, 128.9, 82.0, 78.6, 69.2 (d, *J* = 62.6 Hz, 1C) 37.6, 34.2, 31.5, 28.4, 22.6.

HRMS (ESI, m/z): cacl'd for $C_{13}{}^{13}CH_{22}O_4Na$ [M+Na]$^+$: 278.1444, found: 278.1440.

Preparation of ^{13}*C-**11d***: On 2.3 mmol scale, **General procedure A** was followed with ^{13}C-**17**. Purification by column chromatography (silica gel, CH_2Cl_2 to CH_2Cl_2:EtOAc, 10:1) afforded ^{13}C-**11d** (635 mg, 70% yield, > 99% ^{13}C incorporation)

TLC (silica gel, 1:1, EtOAc: Petroleum ether): $R_f = 0.4$ (UV, $KMnO_4$).

^{1}H NMR (400 MHz, $CDCl_3$): δ 8.64 (dd, J = 7.2, 1.1 Hz, 2H), 8.30 (dd, J = 8.4, 1.1 Hz, 2H), 7.84 – 7.78 (m, 2H), 6.81 (dd, J = 10.4, 0.9 Hz, 1H), 5.96 (d, J = 10.2 Hz, 1H), 4.60 (dd, J = 4.2, 1.4 Hz, 2H), 3.68 – 3.55 (m, 2H), 2.59 – 2.43 (m, 2H), 2.20 (ddd, J = 13.6, 9.5, 5.8 Hz, 1H), 1.80 (dddd, J = 13.6, 6.8, 5.5, 1.2 Hz, 1H), 1.23 (s, 3H).

^{13}C NMR (101 MHz, $CDCl_3$): δ 199.4, 166.8, 159.4, 155.4, 135.3, 132.2, 131.9, 128.8, 127.6, 127.2, 122.1, 78.6, 66.6 (d, J = 62.6 Hz, 1C), 37.4, 33.9, 31.2, 22.4..

HRMS (ESI, m/z): cacl'd for $C_{21}{}^{13}CH_{20}NO_6$ $[M+H]^+$: 395.1319; found 395.1317.

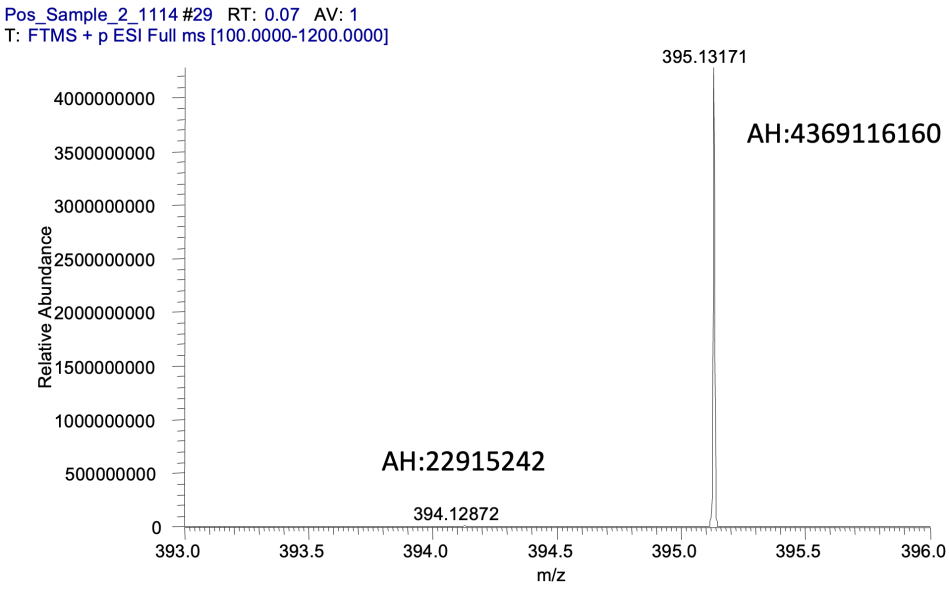

Supplementary Figure 13. The ^{13}C incorporation was determined by HRMS

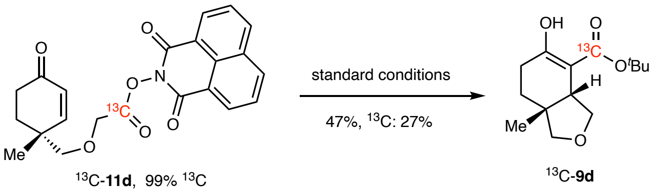

Supplementary Figure 14. Using ^{13}C-11d instead of 11d in standard conditions

On 0.15 mmol scale, **General procedure B** was followed with ^{13}C-**11d**. Purification by column chromatography (silica gel, Petroleum ether:EtOAc, 20:1) afforded ^{13}C-**9d** (18 mg, 47% yield, 27% ^{13}C incorporation)

HRMS (ESI, m/z): ^{13}C-9d: calc'd for $C_{13}{}^{13}CH_{22}O_4Na$ $[M+Na]^+$: 278.1444, found 278.1443.

9d: calc'd for $C_{14}H_{22}O_4Na$ $[M+Na]^+$: 277.1410, found 277.1409.

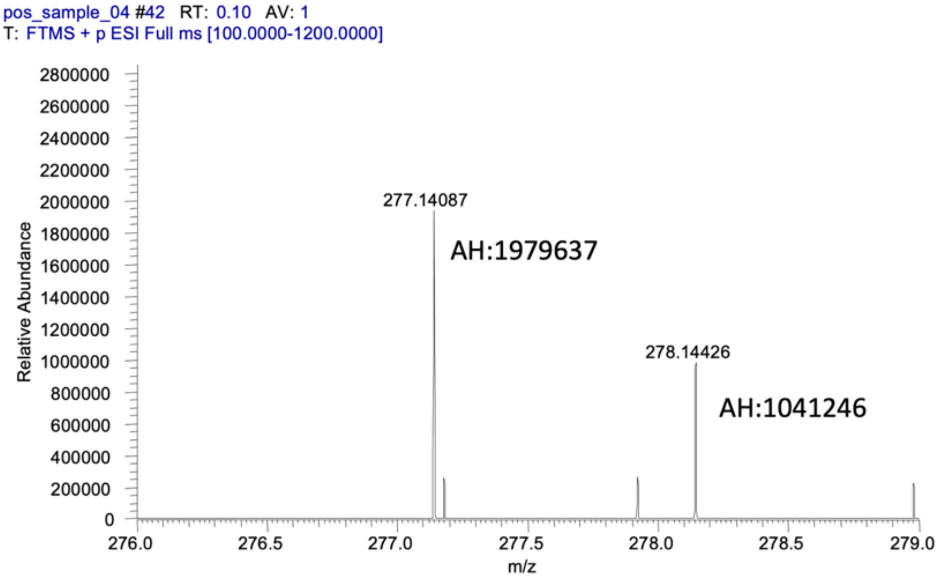

Supplementary Figure 15. The ^{13}C incorporation was determined by HRMS

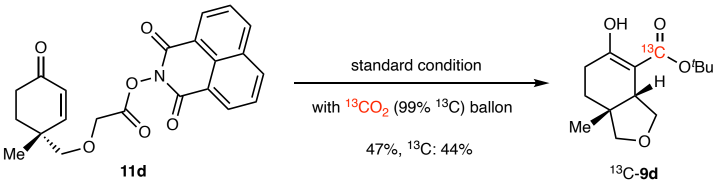

Supplementary Figure 16. Standard reaction under ^{13}C-labled CO_2 atmosphere

On 0.15 mmol scale, **General procedure B** was followed under an atmosphere of $^{13}CO_2$ (> 99% ^{13}C). Purification by column chromatography (silica gel, Petroleum ether:EtOAc, 20:1) afforded ^{13}C-**9d** (18 mg, 47% yield, 44% ^{13}C incorporation)

HRMS (ESI, m/z): ^{13}C-9d: calc'd for $C_{13}{}^{13}CH_{22}O_4Na$ $[M+Na]^+$: 278.1444, found 278.1445.

9d: calc'd for $C_{14}H_{22}O_4Na$ $[M+Na]^+$: 277.1410, found 277.1412.

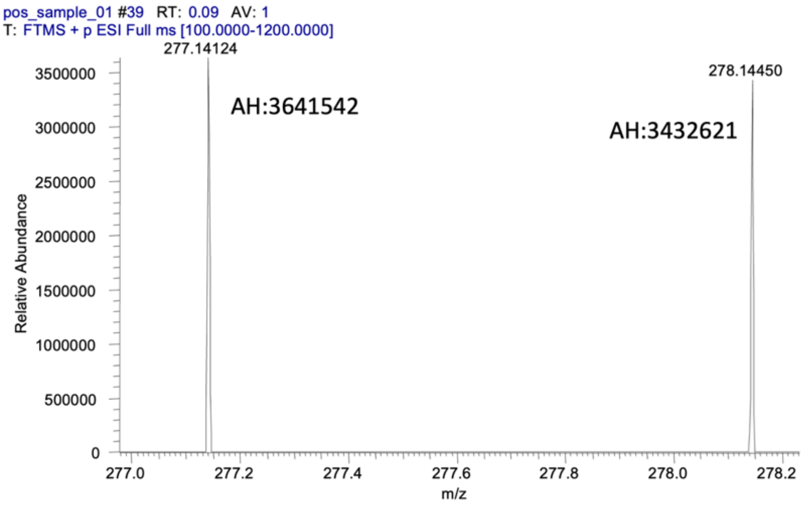

Supplementary Figure 17. The ^{13}C incorporation was determined by HRMS

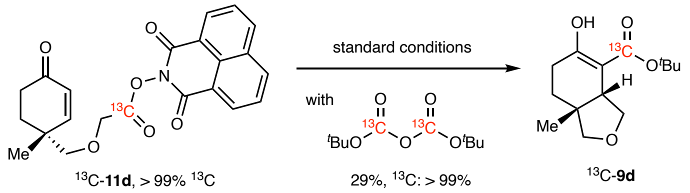

Supplementary Figure 18. Using both ^{13}C-11d and ^{13}C-Boc$_2$O in standard conditions

On 0.15 mmol scale, **General procedure B** was followed with ^{13}C-**11d** and ^{13}C-Boc$_2$O. Purification by column chromatography (silica gel, Petroleum ether:EtOAc, 20:1) afforded ^{13}C-**9d** (11 mg, 29% yield, >99% ^{13}C incorporation).

HRMS (ESI, m/z): ^{13}C-9d: calc'd for $C_{13}{}^{13}CH_{22}O_4Na$ $[M+Na]^+$: 278.1444, found 278.1441.

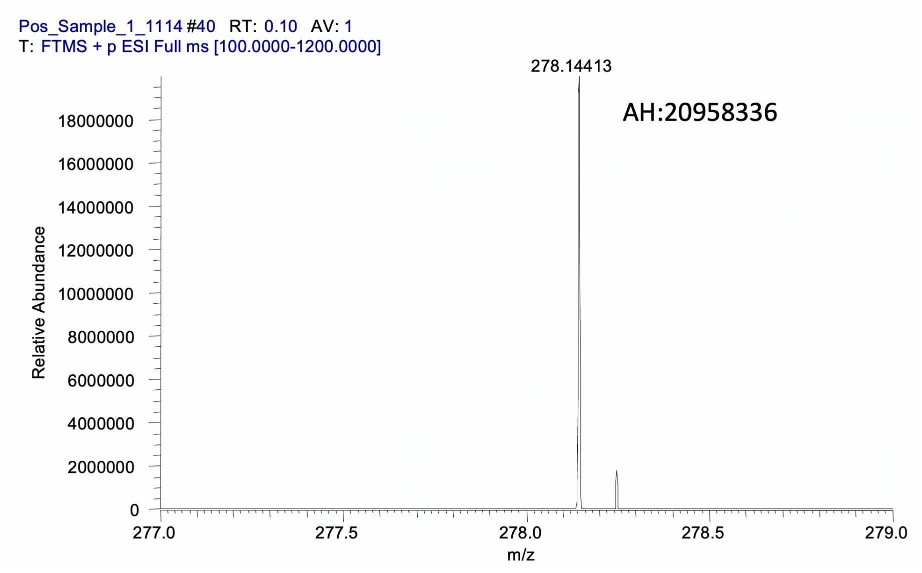

Supplementary Figure 19. The ^{13}C incorporation was determined by HRMS

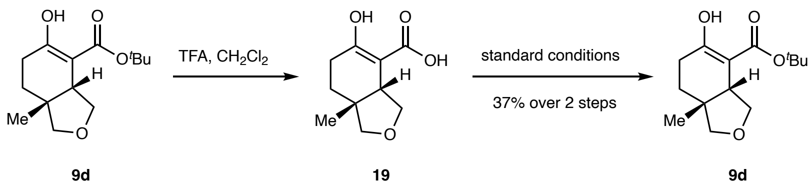

Supplementary Figure 20. Esterification of carboxylic acid 19 with Boc₂O in standard conditions

To a solution of **9d** (38 mg, 0.15 mmol, 1.0 equiv) in CH_2Cl_2 (0.4 mL) was added TFA (0.1 mL). After 4.5 h stirring at room temperature, the reaction mixture was concentrated under reduced pressure to give **19** as light brown oil which was used directly for the next step without further purification.

On 0.15 mmol scale, **General procedure B** was followed with **19** instead of the RAE. The yield of **9d** was determined by LC/MS (37%).

Proposed mechanism

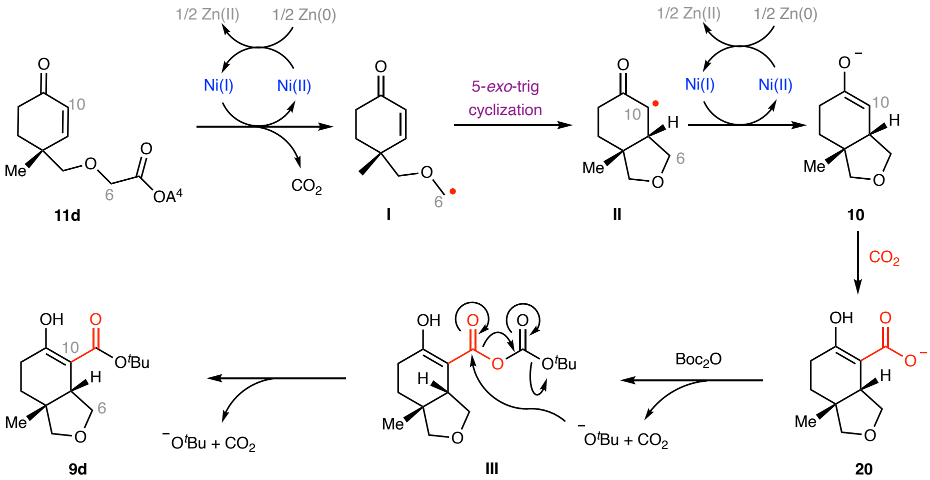

Supplementary Figure 21. Proposed mechanism

2.2 Preparations of Starting Materials in Table 1

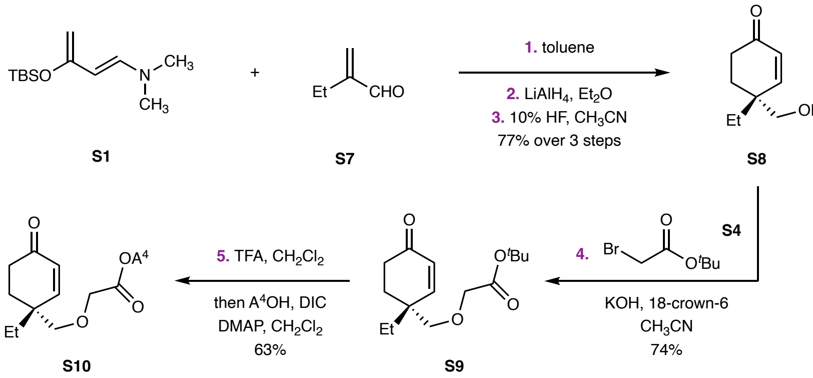

Supplementary Figure 22. Preparation of S10

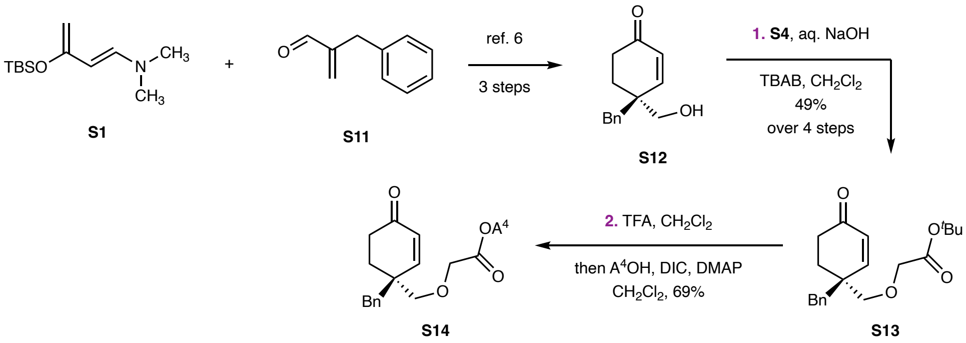

Supplementary Figure 23. Preparation of S14

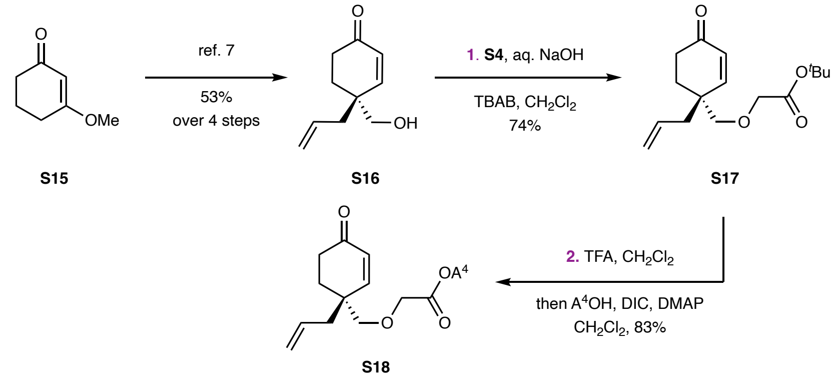

Supplementary Figure 24. Preparation of S18

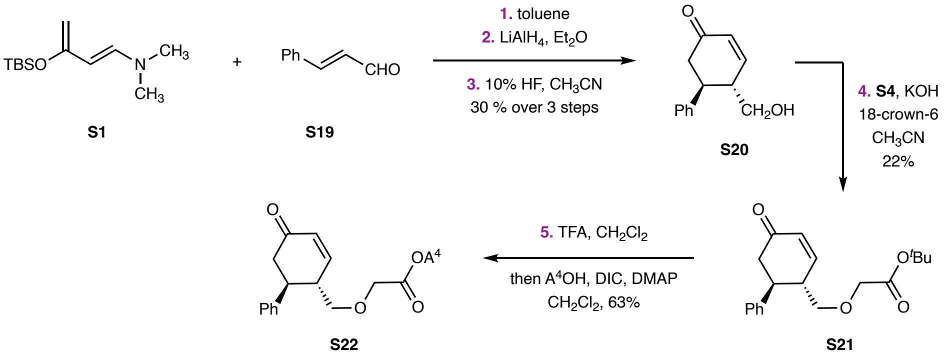

Supplementary Figure 25. Preparation of S22

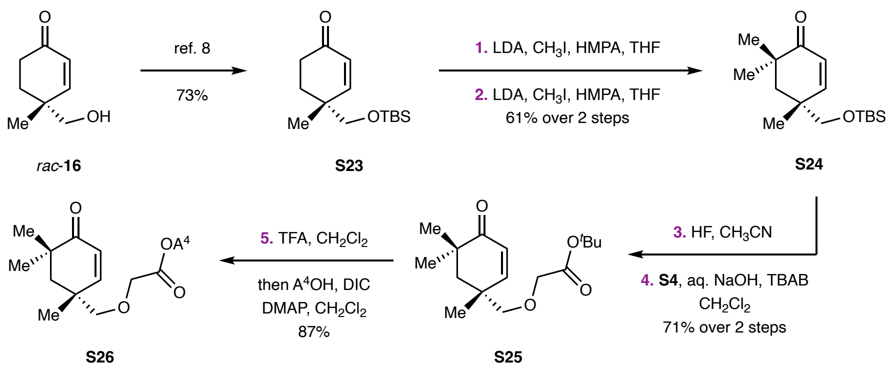

Supplementary Figure 26. Preparation of S26

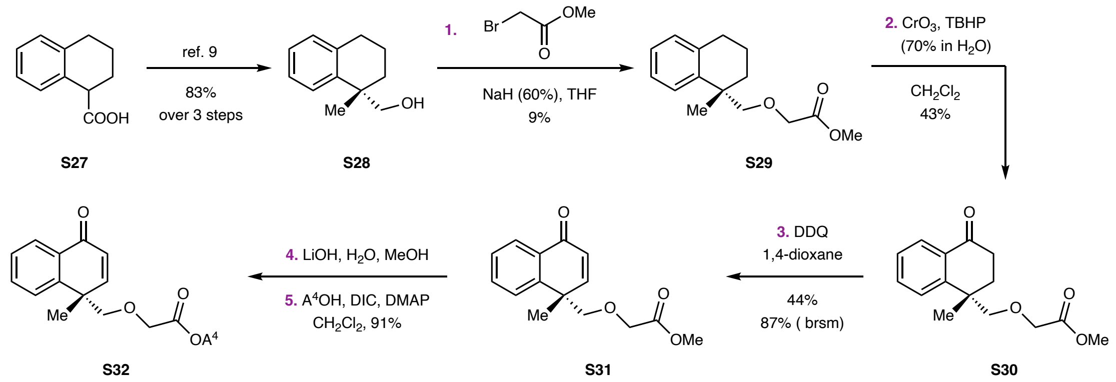

Supplementary Figure 27. Preparation of S32

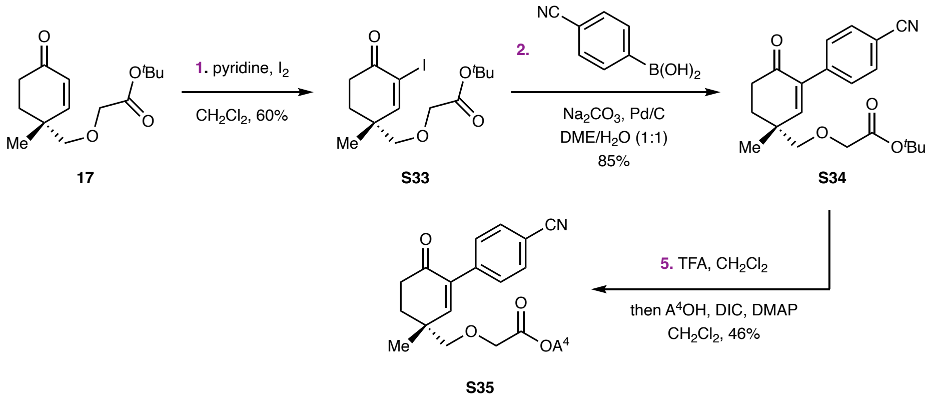

Supplementary Figure 28. Preparation of S35

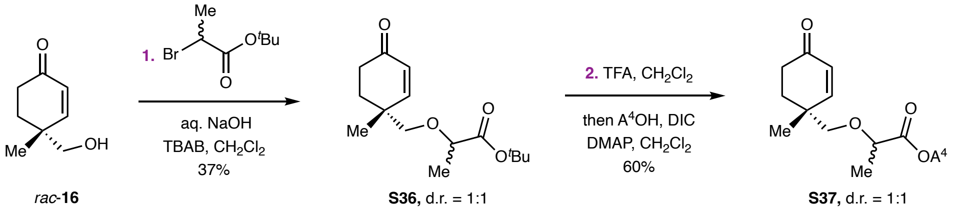

Supplementary Figure 29. Preparation of S37

2.3 Attempts for the Intermolecular Decarboxylative Giese reactions

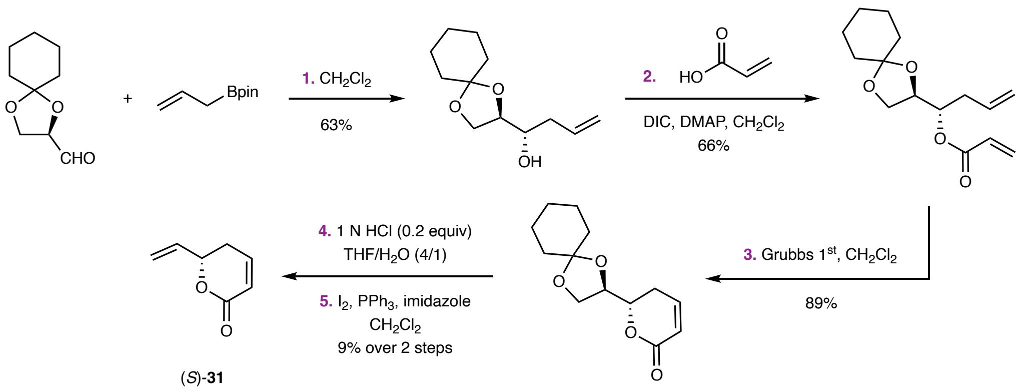

Supplementary Figure 30. Enantioselective preparation of (*S*)-31[10,11]

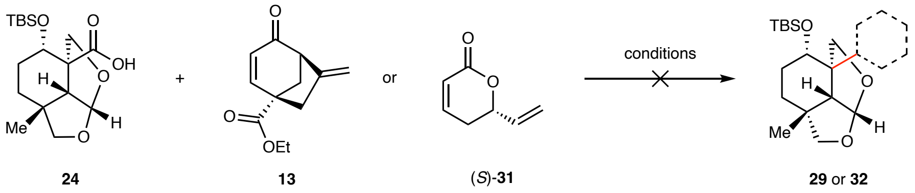

entry	conditions
1[12]	[Ir(df(CF$_3$)ppy)$_2$(dtbbpy)]PF$_6$ (0.1 equiv), K_2HPO_4 (2.0 equiv), DMF argon, 23W CFL
2[13]	Phenathrene (3.0 equiv), 1,4-dicyanobenzene (3.0 equiv) NaOH (1.0 equiv), CH_3CN/H_2O (1:1), argon, Hg lamp (125W)
3[14]	$AgNO_3$ (0.3 equiv), $K_2S_2O_8$ (3.0 equiv), CH_3CN/H_2O (1:1), argon, 40 °C
4[15]	[Acr-Mes]$^+ClO_4^-$ (2.5 mol%), Na_2CO_3 (0.2 equiv), MeOH, air room temperature, blue LEDs

Supplementary Figure 31. Attempts for the intermolecular Giese reaction of 24

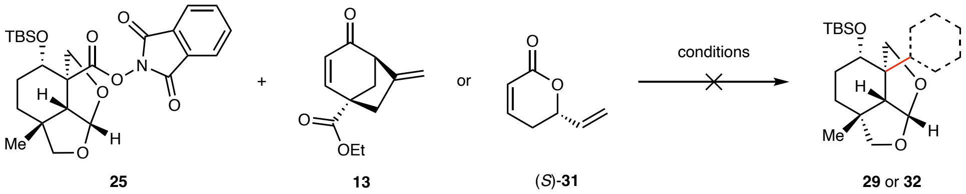

entry	conditions
1[16]	$Ni(ClO_4)_2\cdot 6H_2O$ (1.0 equiv), Zn (10.0 equiv), CH_3CN, argon, 60 °C
2[16]	$Ni(ClO_4)_2\cdot 6H_2O$ (1.0 equiv), Zn (10.0 equiv), LiCl (10.0 equiv), CH_3CN argon, heat (35 °C, 50 °C, 80 °C)
3[17]	$Ru(bpy)_3(PF_6)_2$ (cat.), Hantzsch ester (1.0 equiv), DIPEA (2.0 equiv) CH_2Cl_2, argon, blue LEDs
4[18]	$Gd(OTf)_3$ (50 mol%), Hantzsch ester (1.3 equiv), THF argon, 2 × 34 W blue LEDs
5[19]	NaI (0.1 equiv), PPh_3 (0.1 equiv), CH_3CN, argon, blue LEDs

Note: Decarboxylative product ***S38*** *was detected in all reactions.*

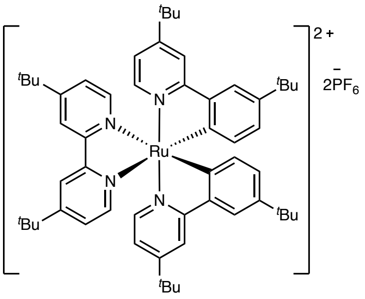

$Ru(bpy)_3(PF_6)_2$

Supplementary Figure 32. Attempts for the intermolecular Giese reaction of RAE 25

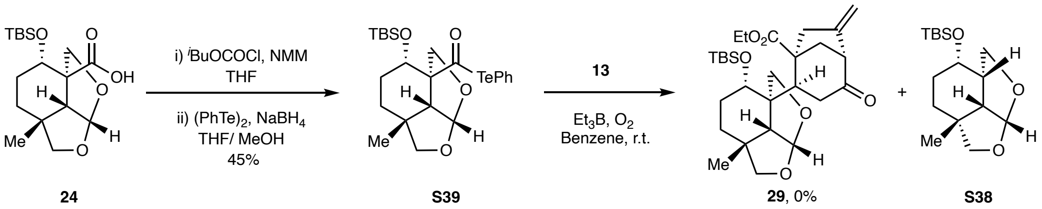

Supplementary Figure 33. Preparation of acyl telluride substrate S39 and attempt for intermolecular Giese reaction with 13

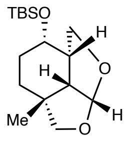

S38

TLC (silica gel, 5:1, Petroleum ether:EtOAc): $R_f = 0.5$ ($KMnO_4$).

^{1}H NMR (400 MHz, $CDCl_3$): δ 5.84 (d, J = 5.2 Hz, 1H), 4.03 – 3.91 (m, 2H), 3.90 – 3.82 (m, 2H), 3.42 (d, J = 8.6 Hz, 1H), 2.62 – 2.53 (m, 1H), 2.35 (dd, J = 9.7, 5.2 Hz, 1H), 1.76 – 1.61 (m, 2H), 1.55 – 1.46 (m, 2H), 1.05 (s, 3H), 0.86 (s, 9H), 0.04 (s, 6H).

^{13}C NMR (101 MHz, $CDCl_3$): δ 111.1, 77.3, 70.4, 69.8, 52.6, 43.8, 39.9, 30.9, 30.1, 25.9, 25.7, 18.0, –4.7, –4.9.

HRMS (ESI, m/z): cacl'd for $C_{16}H_{31}O_3Si$ $[M+H]^+$: 299.2037, found: 299.2033.

$[\alpha]_D^{20.6}$ = +7.3° (c = 0.16, MeOH).

2.4 Attempts for the Intramolecular Decarboxylative Giese reactions

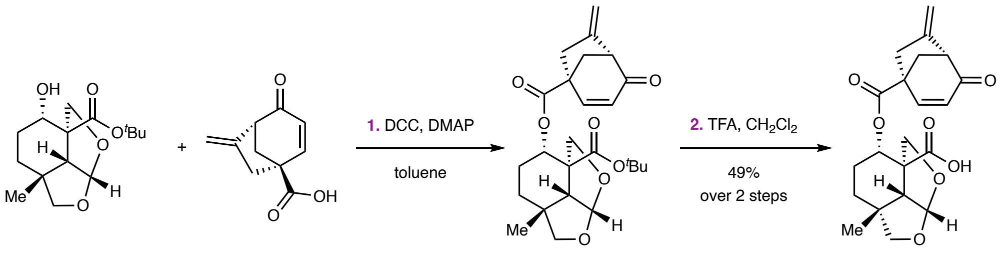

23 **28** **S40** **34**

Supplementary Figure 34. Preparation of carboxylic acid 34

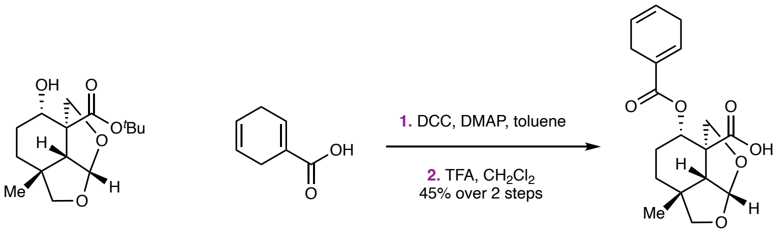

23 **S41** **35**

Supplementary Figure 35. Preparation of carboxylic acid 35

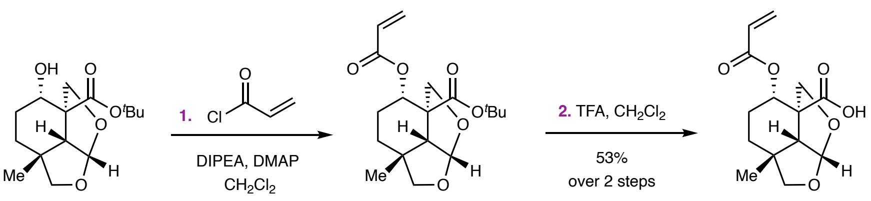

23 **S42** **36**

Supplementary Figure 36. Preparation of carboxylic acid 36

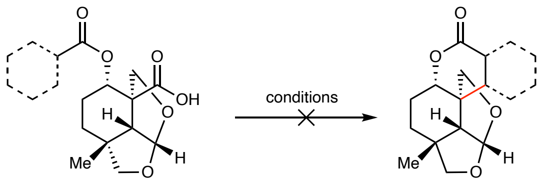

34, 35, 36

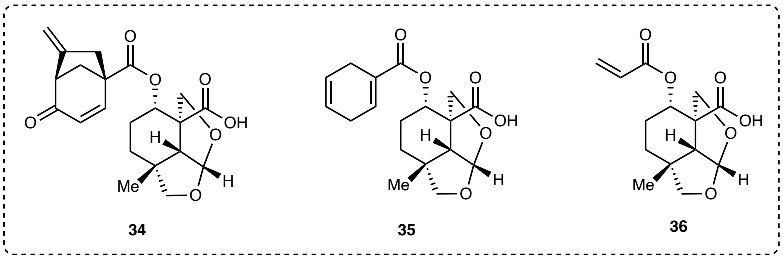

34 **35** **36**

entry	conditions
1[13]	[Ir(df(CF$_3$)ppy)$_2$(dtbbpy)]PF$_6$ (0.1 equiv), K$_2$HPO$_4$ (2.0 equiv)<br>DMF, argon, 23 W CFL
2[14]	Phenathrene (3.0 equiv), 1,4-dicyanobenzene (3.0 equiv)<br>NaOH (1.0 equiv) CH$_3$CN/H$_2$O (1:1), argon, Hg lamp (125 W)
3[15]	AgNO$_3$ (0.3 equiv), K$_2$S$_2$O$_8$ (3.0 equiv), CH$_3$CN/H$_2$O (1:1), argon, 40 °C
4[16]	[Acr-Mes]ClO$_4$ (2.5 mol%), Na$_2$CO$_3$ (0.2 equiv), MeOH, air<br>room temperature, blue LEDs

Supplementary Figure 37. Attempts of intramolecular Giese reactions of carboxylic acids

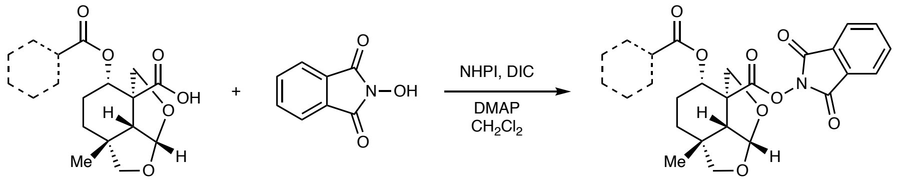

34, 35, 36 A[1]OH **37, 38, 39**

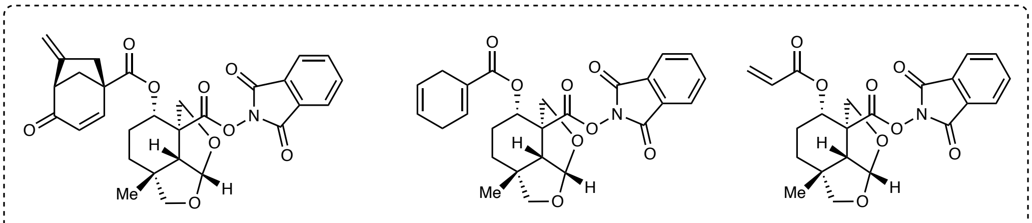

37, 95% **38**, 84% **39**, 71%

Supplementary Figure 38. Preparation of redox-active esters for intramolecular Giese reactions

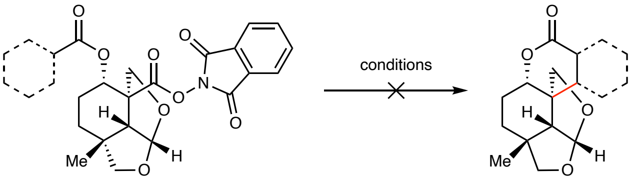

37, 38, 39

entry	conditions
1[16]	$Ni(ClO_4)_2 \cdot 6H_2O$ (1.0 equiv), Zn (10.0 equiv), CH_3CN, argon, 60 °C
2[16]	$Ni(ClO_4)_2 \cdot 6H_2O$ (1.0 equiv), Zn (10.0 equiv), LiCl (10.0 equiv), CH_3CN, argon, heat (35 °C, 50 °C, 80 °C)
3[17]	$Ru(bpy)_3(PF_6)_2$ (cat.), Hantzsch ester (1.0 equiv), DIPEA (2.0 equiv), CH_2Cl_2, argon, blue LEDs
4[18]	$Gd(OTf)_3$ (50 mol%), Hantzsch ester (1.3 equiv), THF, argon 2 × 34 W blue LEDs
5[19]	NaI (0.1 equiv), PPh_3 (0.1 equiv), CH_3CN, argon, blue LEDs

Supplementary Figure 39. Attempts of intramolecular Giese reactions of redox-active esters

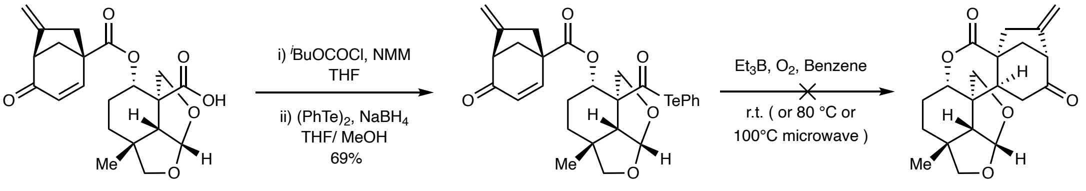

34 **S43** **30**

Supplementary Figure 40. Preparation of acyl telluride substrate and attempts for intramolecular Giese reaction

3. Supplementary Notes

3.1 Experimental Procedure and Spectroscopic Data

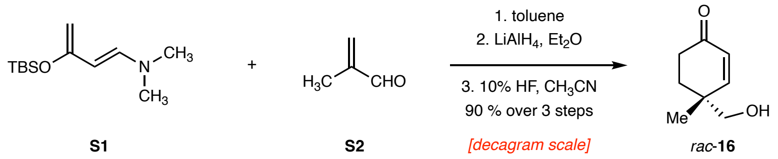

To a cooled solution (0 °C) of **S1** (12.0 g, 52.6 mmol, 1.1 equiv) in toluene (50 mL) was added the 2-methylpropenal (**S2**, 3.32 g/4 mL, 47.4 mmol, 1.0 equiv). The reaction mixture was warmed to room temperature. After 3.5 h stirring, the reaction mixture was concentrated under reduced pressure to give crude product as orange gel, which was used directly for the next step without further purification.

A solution of above crude product (assumed 47.4 mmol, 1.0 equiv) in anhydrous Et_2O (50 mL) was added dropwise to a cooled suspension (–78 °C) of $LiAlH_4$ (2.4 g, 61.6 mmol, 1.3 equiv) in anhydrous Et_2O (50 mL). After stirring at –78 °C for 3 h, the reaction mixture was diluted with Et_2O (200 mL) before being warmed to 0 °C and quenched by slowly addition of H_2O (15 mL). The resulting mixture was stirred violently at room temperature for 30 min before being dried over Na_2SO_4, filtrated, and concentrated under reduced pressure. The crude product was provided as yellow oil, which was used directly for the next step without further purification.

A solution of HF in CH_3CN (5 mL 49% aq. HF + 28 mL CH_3CN) was added dropwise to a solution of above crude product (assumed 47.4 mmol, 1.0 equiv) in CH_3CN (50 mL) at room temperature. After 2.5 h stirring, the reaction was quenched with saturated aq. $NaHCO_3$ (150 mL), extracted with EtOAc (150 mL×3). The combined organic phases were washed with brine, dried over Na_2SO_4, filtrated, and concentrated under reduced pressure. Purification by column chromatography (silica gel, Petroleum ether:EtOAc, 1:1) gave alcohol *rac*-**16**(5.96 g, 90% over 3 steps) as light yellow oil.

TLC (silica gel, 1:2, EtOAc: Petroleum ether): R_f = 0.4 (UV, $KMnO_4$).

^{1}H NMR (400 MHz, $CDCl_3$): δ 6.73 (d, J = 10.2 Hz, 1H), 5.92 (d, J = 10.2 Hz, 1H), 3.69 – 3.30 (m, 2H), 2.83 (m, 1H), 2.61 – 2.37 (m, 2H), 2.05 (m, 1H), 1.71 (m, 1H), 1.11 (s, 3H).

^{13}C NMR (101 MHz, $CDCl_3$): δ 200.2, 156.7, 128.9, 69.8, 38.3, 34.0, 30.9, 22.0.

HRMS (ESI, m/z): cacl'd for $C_8H_{13}O_2$ [M+H]$^+$: 141.0910, found: 141.0908.

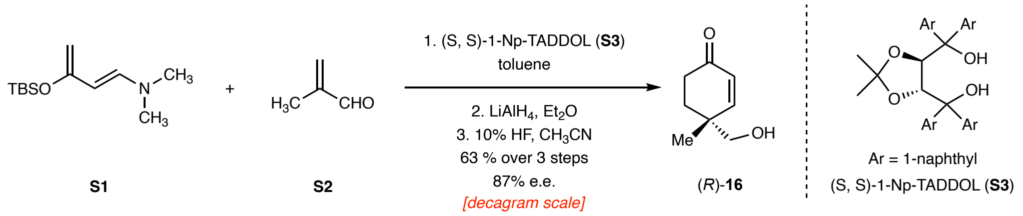

S1 (30 g, 130 mmol, 1.0 equiv) was added dropwise to a cooled (–78 °C) solution of 2-methylpropenal (**S2**, 17.52 g/20.9 mL, 250 mmol, 1.9 equiv) and (*S, S*)-1-Np-TADDOL (**S3**, 8.7 g, 13 mmol, 0.1 equiv) in toluene (110 mL). After 2 days stirring at –78 °C, the reaction mixture was concentrated under reduced pressure to give a crude product which was used directly for the next step without further purification.

A solution of above crude product (assumed 130 mmol, 1.0 equiv) in anhydrous Et_2O (100 mL) was added dropwise over 50 min to a cooled (–78 °C) suspension of $LiAlH_4$ (7.9 g, 210 mmol, 1.6 equiv) in anhydrous Et_2O (100 mL). After stirring at –78 °C for another 3.5 h, the reaction mixture was diluted with Et_2O (400 mL). The reaction mixture was warmed to 0 °C and quenched by slowly addition of H_2O (20 mL). The resulting mixture was stirred violently at room temperature for 30 min before being dried over Na_2SO_4, filtrated, and concentrated under reduced pressure. The crude product was provided as yellow oil which was used directly for the next step without further purification.

A solution of HF in CH_3CN (16 mL 49% aq. HF + 49 mL CH_3CN) was added dropwise to the solution of above crude product (assumed 130 mol, 1.0 equiv) in CH_3CN (150 mL) at room temperature. After 2.5 h stirring, the reaction was quenched slowly with saturated aq. $NaHCO_3$ (400 mL), extracted with EtOAc (400 mL×3). The combined organic phases were washed with brine, dried over Na_2SO_4, filtrated, and concentrated under reduced pressure. Purification by column chromatography (silica gel, Petroleum ether:EtOAc, 1:1) gave alcohol (*R*)-**16** (11.5 g, 63% over 3 steps) as light yellow oil.

TLC (silica gel, 1:2, EtOAc: Petroleum ether): R_f = 0.4 (UV, $KMnO_4$).

^{1}H NMR (400 MHz, $CDCl_3$): δ 6.73 (d, *J* = 10.2 Hz, 1H), 5.92 (d, *J* = 10.2 Hz, 1H), 3.69 – 3.30 (m, 2H), 2.83 (m, 1H), 2.61 – 2.37 (m, 2H), 2.05 (m, 1H), 1.71 (m, 1H), 1.11 (s, 3H).

^{13}C NMR (101 MHz, $CDCl_3$): δ 200.2, 156.7, 128.9, 69.8, 38.3, 34.0, 30.9, 22.0.

HRMS (ESI, m/z): cacl'd for $C_8H_{13}O_2$ $[M+H]^+$: 141.0910, found: 141.0908.

$[\alpha]_D^{23.2}$ = +27.2° (c = 0.99, MeOH).

*Method for determining the enantioselectivity of the asymmetric product, using rac-**16** and (R)-**16**:*

Column: Chiralpak® AD-H

Dimensions: 4.6 × 250 mm

Eluent: *n*-hexanes: IPA = 95:5

Flow rate: 0.5 mL/min

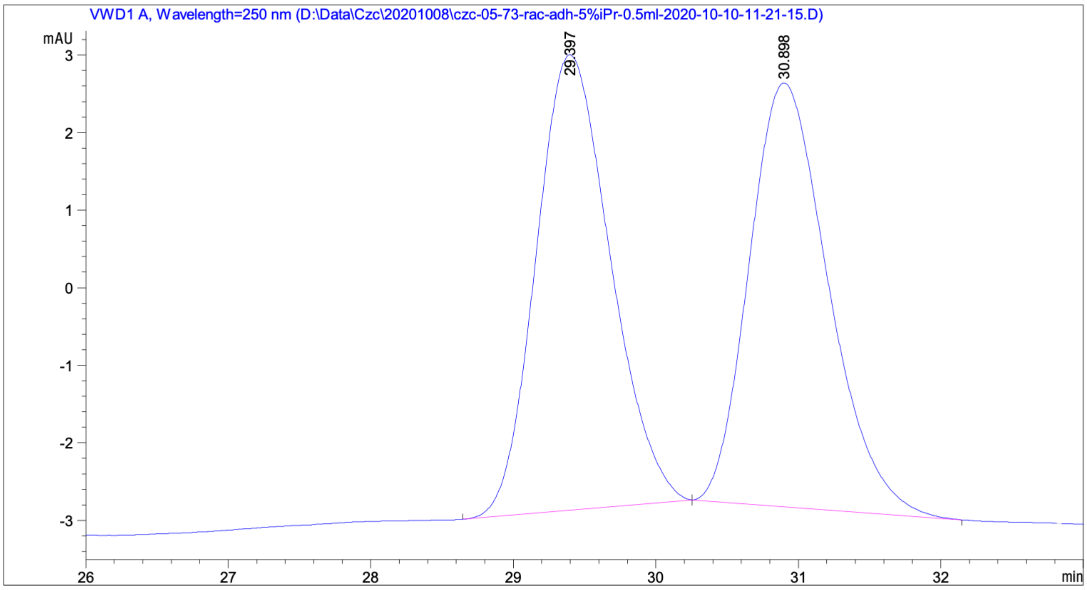

Area Percent Report

Sorted By : Signal
Multiplier : 2.0000
Dilution : 1.0000
Do not use Multiplier & Dilution Factor with ISTDs

Signal 1: VWD1 A, Wavelength=250 nm

Peak #	RetTime [min]	Type	Width [min]	Area [mAU*s]	Height [mAU]	Area %
1	29.397	BB	0.5487	212.82265	5.87439	50.2662
2	30.898	BB	0.5928	210.56831	5.46206	49.7338
Totals :				423.39096	11.33645	

*** End of Report ***

Supplementary Figure 41. The HPLC spectra of *rac*-16.

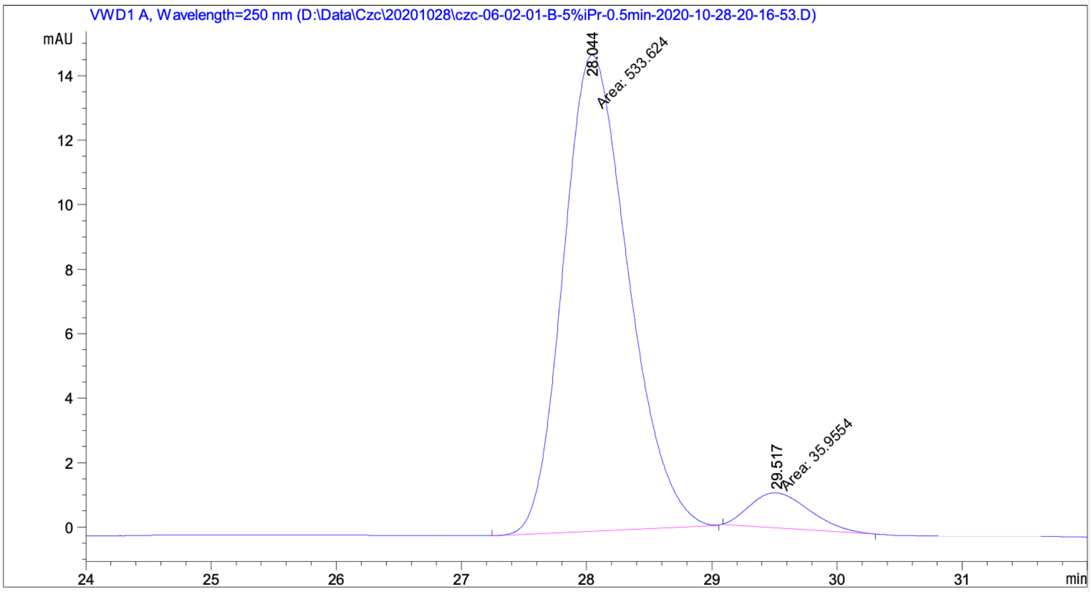


```
=====================================================================
                         Area Percent Report
=====================================================================

Sorted By             :      Signal
Multiplier            :      2.0000
Dilution              :      1.0000
Do not use Multiplier & Dilution Factor with ISTDs


Signal 1: VWD1 A, Wavelength=250 nm

Peak RetTime Type  Width     Area      Height     Area
  #   [min]        [min]   [mAU*s]     [mAU]        %
----|-------|----|-------|----------|----------|--------|
   1  28.044 MM    0.6022  533.62390   14.76876 93.6874
   2  29.517 MM    0.5509   35.95536    1.08773  6.3126

Totals :                   569.57926   15.85650


=====================================================================
                          *** End of Report ***
```

Supplementary Figure 42. The HPLC spectra of the asymmetric product (*R*)-16.

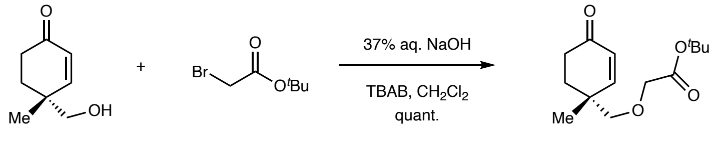

(*R*)-**16** **S4** **17**

To a cooled (0 °C) solution of (*R*)-**16** (22.3 g, 0.16 mol, 1.0 equiv), **S4** (68.5 g/51.2 mL, 0.35 mol, 2.2 equiv), and tetrabutylammonium bromide (TBAB, 52.6 g, 0.16 mol, 1.0 equiv) in CH_2Cl_2 (220 mL) was added aq. NaOH (37%, 220 mL). The reaction mixture was stirred at room temperature for 3 h before being diluted with H_2O (200 mL). The resulting mixture was extracted with CH_2Cl_2 (200 mL×3). The combined organic layers were washed with brine, dried over Na_2SO_4, filtered, and concentrated under reduced pressure. Purification by column chromatography (silica gel, Petroleum ether:EtOAc, 8:1) gave **17** (41.0 g, quant.) as colorless oil.

TLC (silica gel, 1:4, EtOAc:Petroleum ether): R_f = 0.6 (UV, $KMnO_4$).

^{1}H NMR (400 MHz, $CDCl_3$): δ 6.78 (dd, *J* = 10.2, 0.9 Hz, 1H), 5.94 (d, *J* = 10.2 Hz, 1H), 3.96 (d, *J* = 0.6 Hz, 2H), 3.51 – 3.30 (m, 2H), 2.55 – 2.40 (m, 2H), 2.22 – 2.10 (m, 1H), 1.81 – 1.70 (m, 1H), 1.46 (s, 9H), 1.18 (s, 3H).

^{13}C NMR (101 MHz, $CDCl_3$): δ 199.5, 169.4, 155.8, 128.6, 81.7, 78.3, 68.9, 37.3, 33.9, 31.2, 28.1, 22.3.

HRMS (ESI, m/z): cacl'd for $C_{14}H_{22}O_4Na$ $[M+Na]^+$: 277.1410, found: 277.1407.

$[\alpha]_D^{24.6}$ = +39.3° (c = 0.40, MeOH).

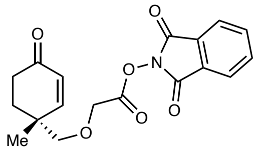

11a

On 2.2 mmol scale, **General procedure A** was followed with **17** and *N*-hydroxyphthalimide to give **11a** (625 mg, 83%) as white amorphous powder (column chromatography: silica gel, EtOAc:Petroleum ether, 1:5).

TLC (silica gel, 1:1, EtOAc:Petroleum ether): R_f = 0.7 (UV, $KMnO_4$).

^{1}H NMR (400 MHz, $CDCl_3$): δ 7.91 (dd, *J* = 5.4, 3.2 Hz, 2H), 7.82 (dd, *J* = 5.6, 3.0 Hz, 2H), 6.77 (dd, *J* = 10.2, 1.1 Hz, 1H), 5.96 (d, *J* = 10.2 Hz, 1H), 4.52 (d, *J* = 1.1 Hz, 2H), 3.58 (d, *J* = 8.4 Hz, 1H), 3.51 (d, *J* = 8.5 Hz, 1H), 2.54 – 2.46 (m, 2H), 2.17 (ddd, *J* = 13.5, 9.0, 6.2 Hz, 1H), 1.84 – 1.74 (m, 1H), 1.21 (s, 3H).

^{13}C NMR (101 MHz, CDCl$_3$): δ 199.4, 166.7, 161.7, 155.2, 135.0, 128.9, 128.7, 124.1, 78.7, 66.4, 37.3, 33.9, 31.2, 22.3.

HRMS (ESI, m/z): cacl'd for $C_{18}H_{17}NO_6Na$ $[M+Na]^+$: 366.0948, found: 366.0945.

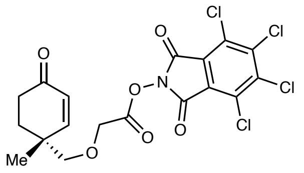

11b

On 0.3 mmol scale, **General procedure A** was followed with **17** and tetrachloro-*N*-hydroxyphthalimide to give product **11b** (109 mg, 76%) as white amorphous powder (column chromatography: silica gel, EtOAc:Petroleum ether, 1:2).

TLC (silica gel, 1:1, EtOAc:Petroleum ether): $R_f = 0.8$ (UV, $KMnO_4$).

^{1}H NMR (400 MHz, CDCl$_3$): *δ* 6.76 (dd, *J* = 10.2, 1.1 Hz, 1H), 5.96 (d, *J* = 10.2 Hz, 1H), 4.52 (d, *J* = 1.0 Hz, 2H), 3.60 – 3.48 (m, 2H), 2.53 – 2.46 (m, 2H), 2.16 (ddd, *J* = 13.6, 8.7, 6.5 Hz, 1H), 1.79 (dtd, *J* = 14.7, 6.5, 6.0, 1.2 Hz, 1H), 1.21 (s, 3H).

^{13}C NMR (101 MHz, CDCl$_3$): *δ* 199.4, 166.5, 157.4, 155.1, 141.4, 130.8, 129.1, 124.7, 78.9, 66.5, 37.4, 34.0, 31.3, 22.4.

HRMS (ESI, m/z): calc'd for $C_{18}H_{13}Cl_4NO_6Na$ $[M+Na]^+$: 501.9389 , found 501.9384.

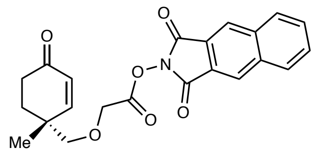

11c

On 0.3 mmol scale, **General procedure A** was followed with **17** and 2-hydroxy-1*H*-benzo[*f*]isoindole-1,3(2*H*)-dione to give product **11c** (82 mg, 69%) as white amorphous powder (column chromatography: silica gel, EtOAc:Petroleum ether, 1:2).

TLC (silica gel, 1:1, EtOAc: Petroleum ether): $R_f = 0.7$ (UV, $KMnO_4$).

^{1}H NMR (400 MHz, CDCl$_3$): δ 8.41 (s, 2H), 8.09 (dd, *J* = 6.2, 3.3 Hz, 2H), 7.76 (dd, *J* = 6.2, 3.2 Hz, 2H), 6.78 (dd, *J* = 10.2, 1.1 Hz, 1H), 5.97 (d, *J* = 10.2 Hz, 1H), 4.55 (d, *J* = 1.2 Hz, 2H), 3.64 – 3.51 (m, 2H), 2.57 – 2.44 (m, 2H), 2.18 (ddd, *J* = 13.5, 9.1, 6.2 Hz, 1H), 1.80 (dddd, *J* = 13.5, 6.7, 5.5, 1.2 Hz, 1H), 1.22 (s, 3H).

^{13}C NMR (101 MHz, CDCl$_3$): δ 199.5, 166.7, 161.4, 155.3, 135.6, 130.6, 130.0, 129.0, 126.2, 124.3, 78.8, 66.6, 37.5, 34.0, 31.3, 22.5.

HRMS (ESI, m/z): cacl'd for $C_{22}H_{20}NO_6$ $[M+H]^+$: 394.1285, found: 398.1278.

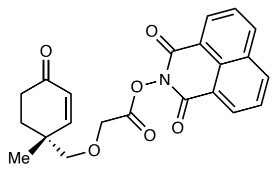

11d

On 160 mmol scale, **General procedure A** was followed with **17** and *N*-hydroxynaphthalimide to give product **11d** (51.0 g, 81%) as light yellow amorphous powder (column chromatography: silica gel, CH_2Cl_2:EtOAc, 10:1).

TLC (silica gel, 1:1, EtOAc:Petroleum ether): $R_f = 0.4$ (UV, $KMnO_4$).

^{1}H NMR (400 MHz, $CDCl_3$): δ 8.64 (dd, *J* = 7.2, 1.1 Hz, 2H), 8.30 (dd, *J* = 8.4, 1.1 Hz, 2H), 7.84 – 7.78 (m, 2H), 6.81 (d, *J* = 10.2 Hz, 1H), 5.96 (d, *J* = 10.2 Hz, 1H), 4.60 (d, *J* = 1.4 Hz, 2H), 3.69 – 3.55 (m, 2H), 2.59 – 2.43 (m, 2H), 2.20 (m, 1H), 1.80 (m, 1H), 1.23 (s, 3H).

^{13}C NMR (101 MHz, $CDCl_3$): δ 199.4, 166.9, 159.4, 155.4, 135.3, 132.1, 131.9, 128.8, 127.6, 127.2, 122.1, 78.6, 66.7, 37.4, 33.9, 31.2, 22.4.

HRMS (ESI, m/z): cacl'd for $C_{22}H_{20}NO_6$ $[M+H]^+$: 394.1285, found: 394.1279.

$[\alpha]_D^{25.0}$ = +21.0° (c = 0.35, $CHCl_3$).

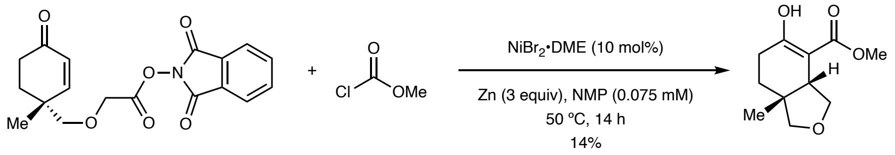

On 0.15 mmol scale, **General procedure B** was followed with RAE **11a** and methyl chloroformate (**12a**) instead of Boc_2O to give product **9a** (4.5 mg, 14%) as light yellow oil (column chromatography: silica gel, Petroleum ether:EtOAc, 50:1 to 20:1).

TLC (silica gel, EtOAc: Petroleum ether, 1:5): $R_f = 0.4$ (UV, $KMnO_4$).

^{1}H NMR (400 MHz, $CDCl_3$): δ 12.21 (s, 1H), 4.22 (t, *J* = 8.4 Hz, 1H), 3.75 (s, 3H), 3.64 (d, *J* = 8.3 Hz, 1H), 3.57 (d, *J* = 8.3 Hz, 1H), 3.48 (t, *J* = 8.4 Hz, 1H), 2.72 (t, *J* = 8.4 Hz, 1H), 2.45 – 2.26 (m, 2H), 1.85 –1.90 (m, 1H), 1.45 – 1.49 (m, 1H), 1.06 (s, 3H).

^{13}C NMR (101 MHz, $CDCl_3$): δ 173.1, 172.2, 97.2, 79.9, 75.0, 51.6, 43.8, 39.9, 28.4, 26.3, 21.6.

HRMS (ESI, m/z): cacl'd for $C_{11}H_{17}O_4$ $[M+H]^+$: 213.1122, found: 213.1117.

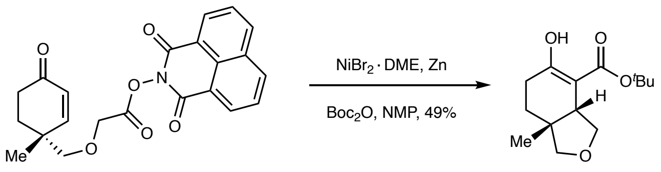

On 0.15 mmol scale, **General procedure B** was followed with RAE **11d** to give the product **9d** (18.7 mg, 49%) as colorless oil (column chromatography: silica gel, Petroleum:EtOAc, 10:1).

TLC (silica gel, EtOAc:Petroleum ether, 1:5): $R_f = 0.4$ (UV, $KMnO_4$).

^{1}H NMR (400 MHz, $CDCl_3$): δ 12.44 (s, 1H), 4.22 (t, *J* = 8.4 Hz, 1H), 3.64 (d, *J* = 8.3 Hz, 1H), 3.56 (d, *J* = 8.3 Hz, 1H), 3.50 (t, *J* = 8.3 Hz, 1H), 2.65 (t, *J* = 8.4 Hz, 1H), 2.44 – 2.30 (m, 1H), 2.32 – 2.19 (m, 1H), 1.85 (m, 1H), 1.49 (s, 9H), 1.48 – 1.42 (m, 1H), 1.06 (s, 3H).

^{13}C NMR (101 MHz, $CDCl_3$): δ 172.7, 171.4, 98.9, 81.6, 80.1, 75.4, 44.2, 40.0, 28.6, 28.4, 26.4, 21.7.

HRMS (ESI, m/z): cacl'd for $C_{14}H_{22}O_4Na$ $[M+Na]^+$: 277.1410, found: 277.1406.

$[\alpha]_D^{22.8}$ = –49.1° (c = 0.32, MeOH).

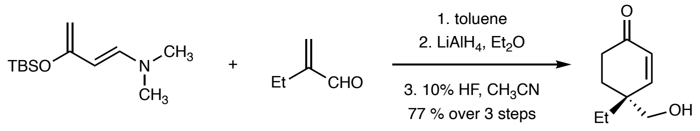

According to the procedure for the preparation of *rac*-**16**, **S8** was prepared in 77% yield over 3 steps from **S7** (4 mmol).

TLC (silica gel, 1:2, EtOAc:Petroleum ether): $R_f = 0.4$ (UV, $KMnO_4$).

^{1}H NMR (400 MHz, $CDCl_3$): δ 6.74 (dt, *J* = 10.3, 0.9 Hz, 1H), 6.04 (d, *J* = 10.3 Hz, 1H), 3.67 – 3.53 (m, 2H), 2.49 (m, 2H), 2.03 – 1.84 (m, 2H), 1.65 – 1.56 (m, 2H), 0.93 (t, *J* = 7.6 Hz, 3H).

^{13}C NMR (101 MHz, $CDCl_3$): δ 200.2, 156.0, 129.6, 67.1, 41.0, 33.9, 27.9, 27.8, 8.4.

HRMS (ESI, m/z): cacl'd for $C_9H_{15}O_2$ $[M+H]^+$: 155.1067, found: 155.1064.

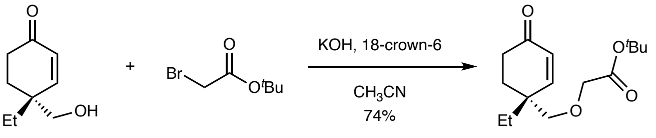

To a solution of **S8** (1.54 g, 10 mmol, 1.0 equiv) in CH_3CN (30 mL) was added KOH (670 mg, 12 mmol, 1.2 equiv), 18-crown-6 (3.16 g, 12 mmol, 1.2 equiv), and **S4** (1.95 g, 10 mmol, 1.0 equiv) successively. After 5 h stirring at room temperature, the reaction mixture was concentrated (to *ca.* 10 mL) and diluted with H_2O (60 mL). The resulting mixture was extracted with CH_2Cl_2 (30 mL×3), the combined organic phases were washed with H_2O and brine successively, dried over Na_2SO_4, filtrated, and concentrated under reduced pressure. Purification by column chromatography (silica gel, Petroleum ether:EtOAc, 9:1) gave **S9** (2.16g, 74%) as colorless oil.

TLC (silica gel, 1:4, EtOAc: Petroleum ether): R_f = 0.5 (UV, $KMnO_4$).

^{1}H NMR (400 MHz, CDCl$_3$): δ 6.81 – 6.79 (m, 1H), 6.00 (d, *J* = 10.3 Hz, 1H), 3.95 (d, *J* = 1.0 Hz, 2H), 3.51 (d, *J* = 8.7 Hz, 1H), 3.40 (d, *J* = 8.7 Hz, 1H), 2.62 – 2.36 (m, 2H), 2.07 – 1.82 (m, 2H), 1.68 – 1.53 (m, 2H), 1.47 (s, 9H), 0.93 (t, *J* = 7.6 Hz, 3H).

^{13}C NMR (101 MHz, CDCl$_3$): δ 199.6, 169.5, 155.4, 129.3, 81.7, 75.8, 69.1, 40.1, 33.9, 28.2, 28.1, 8.4.

HRMS (ESI, m/z): cacl'd for $C_{15}H_{24}O_4Na$ $[M+Na]^+$: 291.1567, found: 291.1564.

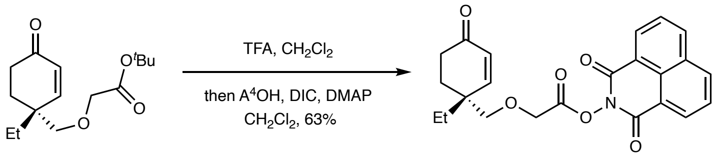

On 6.0 mmol scale, **General procedure A** was followed with **S9** and *N*-hydroxynaphthalimide to give the product **S10** (1.54 g, 63%) as white amorphous powder (column chromatography: silica gel, CH_2Cl_2:EtOAc, 10:1).

TLC (silica gel, 1:1, EtOAc:Petroleum ether): R_f = 0.7 (UV, $KMnO_4$).

^{1}H NMR (400 MHz, CDCl$_3$): δ 8.62 (d, *J* = 7.2 Hz, 2H), 8.28 (d, *J* = 8.4 Hz, 2H), 7.79 (t, *J* = 7.8 Hz, 2H), 6.82 (d, *J* = 11.1 Hz, 1H), 6.00 (d, *J* = 10.3 Hz, 1H), 4.59 (dd, *J* = 2.3, 1.4 Hz, 2H), 3.71 (d, *J* = 8.6 Hz, 1H), 3.61 (d, *J* = 9.9 Hz, 1H), 2.60 – 2.50 (m, 1H), 2.43 (dddd, *J* = 17.5, 8.5, 5.3, 0.9 Hz, 1H), 2.05 (ddd, *J* = 13.8, 8.4, 5.5 Hz, 1H), 1.91 (ddd, *J* = 13.8, 8.4, 5.4 Hz, 1H), 1.73 – 1.54 (m, 2H), 0.94 (t, *J* = 6.9 Hz, 3H).

^{13}C NMR (101 MHz, CDCl$_3$): δ 199.6, 166.8, 159.4, 154.9, 135.2, 132.1, 131.9, 129.4, 127.5, 127.1, 122.1, 76.0, 66.6, 40.1, 33.8, 28.2, 28.1, 8.3.

HRMS (ESI, m/z): cacl'd for $C_{23}H_{22}NO_6$ $[M+H]^+$: 408.1442, found: 408.1437.

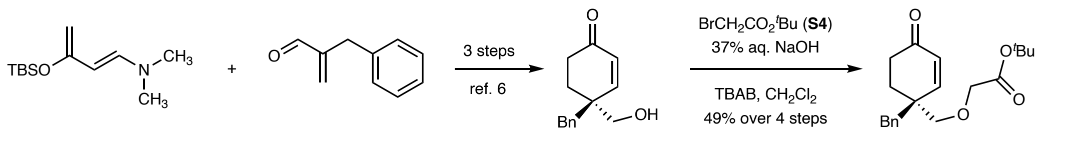

S12 was prepared on a scale of 3.4 mmol according to Rawal's procedure [6] through 3 steps. **S12** was provided as light yellow oil which was used directly for the next step without purification.

According to the procedure of the preparation of **17**, **S13** (591 mg, 49% over 4 steps) was prepared from **S11** (column chromatography: silica gel, Petroleum ether:EtOAc, 10:1).

TLC (silica gel, 1:5, EtOAc:Petroleum ether): $R_f = 0.4$ (UV, $KMnO_4$).

^{1}H NMR (400 MHz, $CDCl_3$): δ 7.32 – 7.28 (m, 2H), 7.24– 7.17 (m, 3H), 6.84 (d, *J* = 10.3 Hz, 1H), 6.02 (d, *J* = 10.3 Hz, 1H), 3.98 (s, 2H), 3.40 (d, *J* = 8.7 Hz, 1H), 3.31 (d, *J* = 8.7 Hz, 1H), 2.93 – 2.84 (m, 2H), 2.50 – 2.43 (m, 2H), 2.06 – 1.99 (m, 1H), 1.92 – 1.85 (m, 1H), 1.47 (s, 9H).

^{13}C NMR (101 MHz, $CDCl_3$): δ 199.3, 169.4, 154.4, 136.5, 130.4, 129.5, 128.3, 126.7, 81.8, 75.2, 69.0, 41.0, 33.8, 28.8, 28.1.

HRMS (ESI, m/z): cacl'd for $C_{20}H_{26}O_4Na$ $[M+Na]^+$: 353.1723, found: 353.1716.

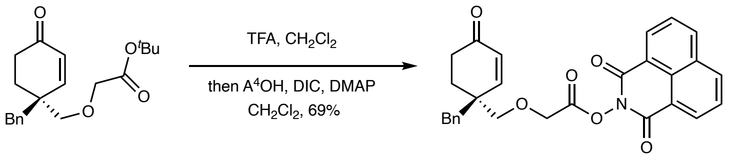

On 1.8 mmol scale, **General procedure A** was followed with **S13** and *N*-hydroxynaphthalimide to give the product **S14** (580 mg, 69%) as white amorphous powder (column chromatography: silica gel, CH_2Cl_2:EtOAc, 10:1).

TLC (silica gel, 1:1, EtOAc:Petroleum ether): $R_f = 0.6$ (UV, $KMnO_4$).

^{1}H NMR (400 MHz, $CDCl_3$) δ 8.65 (dd, *J* = 7.3, 1.2 Hz, 2H), 8.30 (dd, *J* = 8.3, 1.2 Hz, 2H), 7.82 (dd, *J* = 8.2, 7.3 Hz, 2H), 7.31 – 7.26 (m, 2H), 7.26 – 7.20 (m, 3H), 6.85 (d, *J* = 10.3 Hz, 1H), 6.02 (d, *J* = 10.3 Hz, 1H), 4.62 (m, 2H), 3.62 – 3.52 (m, 2H), 2.97 – 2.89 (m, 2H), 2.51 – 2.43 (m, 2H), 2.08 – 2.02 (m, 1H), 1.96 – 1.91 (m, 1H).

^{13}C NMR (101 MHz, $CDCl_3$) δ 199.3, 166.7, 159.4, 153.9, 136.4, 135.3, 132.2, 131.9, 130.5, 129.6, 128.3, 127.5, 127.2, 126.8, 122.1, 75.4, 66.6, 41.0, 41.0, 33.8, 28.8.

HRMS (ESI, m/z): cacl'd for $C_{28}H_{24}NO_6$ $[M+H]^+$: 470.1598, found: 470.1588.

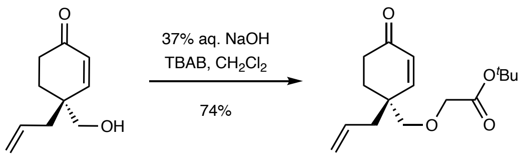

S16 was prepared according to Nicolaou's method [7]. **S17** (338 mg, 74%) was prepared from **S16** (1.5 mmol) according to the procedure of the preparation of **17** (column chromatography: Petroleum ether:EtOAc, 10:1).

TLC (silica gel, 1:4, EtOAc: Petroleum ether): R_f = 0.5 (UV, $KMnO_4$).

^{1}H NMR (400 MHz, $CDCl_3$): δ 6.81 (d, *J* = 10.3 Hz, 1H), 6.01 (d, *J* = 10.3 Hz, 1H), 5.86 – 5.75 (m, 1H), 5.16 – 5.09 (m, 2H), 3.96 (s, 2H), 3.50 (d, *J* = 8.7 Hz, 1H), 3.39 (d, *J* = 8.7 Hz, 1H), 2.58 – 2.41 (m, 2H), 2.32 (dd, *J* = 7.5, 1.3 Hz, 2H), 2.07 – 2.31 (m, 1H), 1.94 – 1.85 (m, 1H), 1.47 (s, 9H).

^{13}C NMR (101 MHz, $CDCl_3$): δ 199.5, 169.4, 154.6, 133.2, 129.5, 119.0, 81.8, 75.7, 69.0, 40.1, 40.0, 33.8, 28.7, 28.1.

HRMS (ESI, m/z): cacl'd for $C_{16}H_{24}O_4Na$ $[M+Na]^+$: 303.1567, found: 303.1560.

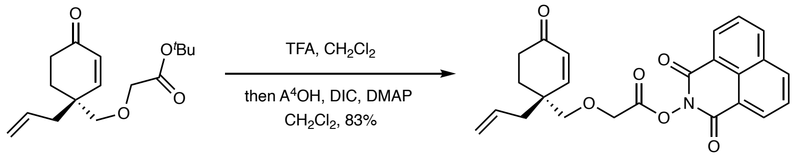

On 1.1 mmol scale, **General procedure A** was followed with **S17** and *N*-hydroxynaphthalimide to give the product **S18** (385 mg, 83%) as white amorphous powder (column chromatography: silica gel, CH_2Cl_2:EtOAc, 10:1).

TLC (silica gel, 1:1, EtOAc:Petroleum ether): R_f = 0.7 (UV, $KMnO_4$).

^{1}H NMR (400 MHz, $CDCl_3$): δ 8.63 (dd, *J* = 7.3, 1.2 Hz, 2H), 8.29 (dd, *J* = 8.3, 1.1 Hz, 2H), 7.80 (dd, *J* = 8.3, 7.3 Hz, 2H), 6.83 (d, *J* = 10.3 Hz, 1H), 6.01 (d, *J* = 10.3 Hz, 1H), 5.88 – 5.75 (m, 1H), 5.18 – 5.10 (m, 2H), 4.60 (d, *J* = 2.2 Hz, 2H), 3.70 (d, *J* = 8.6 Hz, 1H), 3.60 (d, *J* = 8.6 Hz, 1H), 2.59 – 2.41 (m, 2H), 2.35 (d, *J* = 7.5 Hz, 2H), 2.10 – 2.03 (m, 1H), 1.95 – 1.88 (m, 1H).

^{13}C NMR (101 MHz, $CDCl_3$): δ 199.4, 166.8, 159.4, 154.1, 135.3, 133.1, 132.2, 131.9, 129.6, 127.6, 127.2, 122.1, 119.2, 75.9, 66.6, 40.1, 40.0, 33.8, 28.6.

HRMS (ESI, m/z): cacl'd for $C_{24}H_{22}NO_6$ $[M+H]^+$: 420.1442, found: 420.1432.

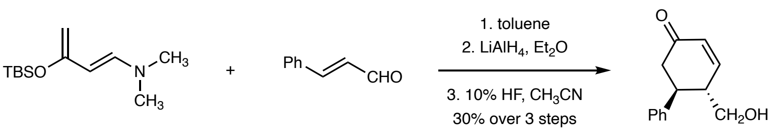

S1 **S19** **S20**

S20 was prepared in 30% yield from **S19** (22 mmol) according to the procedure for the preparation of *rac*-**16**.

TLC (silica gel, 1:1, EtOAc:Petroleum ether): $R_f = 0.4$ (UV).

^{1}H NMR (400 MHz, $CDCl_3$): δ 7.37 – 7.33 (m, 2H), 7.32 – 7.26 (m, 2H), 7.24 (m, 1H), 7.10 (dd, J = 10.2, 2.1 Hz, 1H), 6.18 (ddd, J = 10.2, 2.8, 0.9 Hz, 1H), 3.71 (dd, J = 10.6, 3.9 Hz, 1H), 3.46 (dd, J = 10.6, 6.3 Hz, 1H), 3.24 (ddd, J = 13.2, 10.5, 4.6 Hz, 1H), 2.86 – 2.77 (m, 1H), 2.77 – 2.66 (m, 2H).

^{13}C NMR (101 MHz, $CDCl_3$): δ 198.3, 151.8, 141.8, 130.4, 129.7, 127.49, 127.47, 63.1, 45.3, 45.1, 43.3.

HRMS (ESI, m/z): cacl'd for $C_{13}H_{15}O_2$ $[M+H]^+$: 203.1067, found: 203.1063.

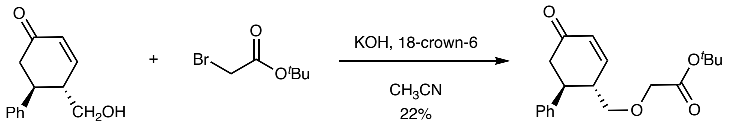

S20 **S4** **S21**

To a solution of **S20** (1.3 g, 6.4 mmol, 1.0 equiv) in CH_3CN (30 mL) was added KOH (430 mg, 7.7 mmol, 1.2 equiv), 18-crown-6 (2.03 g, 7.7 mmol, 1.2 equiv), and **S4** (1.25 g, 6.4 mmol, 1.0 equiv) successively. After 5 h stirring at room temperature, the reaction mixture was concentrated (to *ca.* 10 mL) and diluted with H_2O (60 mL). The resulting mixture was extracted with CH_2Cl_2 (30 mL×3). The combined organic phases were washed with brine, dried over Na_2SO_4, filtrated, and concentrated under reduced pressure. Purification by column chromatography (silica gel, Petroleum ether:EtOAc, 9:1) gave **S21** (410 mg, 22%) as colorless oil.

TLC (silica gel, 1:4, EtOAc:Petroleum ether): $R_f = 0.3$ (UV).

^{1}H NMR (400 MHz, $CDCl_3$): δ 7.38 – 7.32 (m, 2H), 7.29 – 7.26 (m, 1H), 7.25 – 7.21 (m, 3H), 6.17 (ddd, J = 10.2, 2.7, 0.9 Hz, 1H), 3.96 – 3.80 (m, 2H), 3.55 (dd, J = 8.8, 3.8 Hz, 1H), 3.29 – 3.19 (m, 2H), 2.94 – 2.87 (m, 1H), 2.75 – 2.63 (m, 2H), 1.44 (s, 9H).

^{13}C NMR (101 MHz, $CDCl_3$): δ 198.7, 169.3, 151.8, 141.7, 129.6, 128.9, 127.4, 127.2, 81.8, 71.3, 68.9, 45.1, 43.33, 43.30, 28.1.

HRMS (ESI, m/z): cacl'd for $C_{19}H_{24}O_4Na$ $[M+Na]^+$: 291.1567, found: 291.1564.

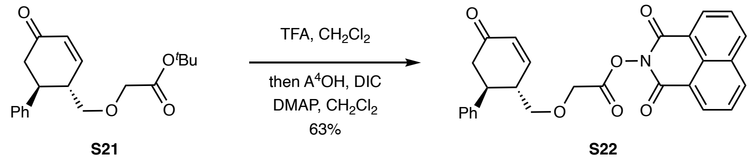

On 1.4 mmol scale, **General procedure A** was followed with **S21** and *N*-hydroxynaphthalimide to give the product **S22** (401 mg, 63%) as white amorphous powder (column chromatography: silica gel, CH_2Cl_2:EtOAc, 10:1).

TLC (silica gel, 2:1, EtOAc: Petroleum ether): R_f = 0.2 (UV, $KMnO_4$).

^{1}H NMR (400 MHz, $CDCl_3$): δ 8.63 (dd, J = 7.3, 1.2 Hz, 2H), 8.30 (dd, J = 8.3, 1.2 Hz, 2H), 7.81 (dd, J = 8.3, 7.3 Hz, 2H), 7.37 – 7.32 (m, 2H), 7.28 (m, 2H), 7.23 – 7.20 (m, 2H), 6.15 (ddd, J = 10.0, 2.7, 0.8 Hz, 1H), 4.58 – 4.43 (m, 2H), 3.73 (dd, J = 8.8, 3.7 Hz, 1H), 3.51 (dd, J = 8.8, 6.4 Hz, 1H), 3.35 – 3.27 (m, 1H), 2.99 – 2.92 (m, 1H), 2.78 – 2.61 (m, 2H).

^{13}C NMR (101 MHz, $CDCl_3$): δ 198.8, 166.9, 159.5, 151.8, 141.2, 135.4, 132.3, 132.0, 129.9, 129.1, 127.7, 127.5, 127.4, 127.3, 122.2, 71.9, 66.7, 45.2, 43.5, 43.3.

HRMS (ESI, m/z): cacl'd for $C_{27}H_{22}NO_6$ $[M+H]^+$: 456.1442, found: 456.1432.

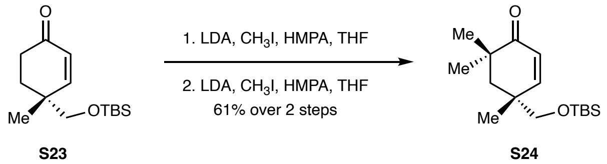

S23 was prepared from *rac*-**16** according to Yuan's method[8].

Freshly prepared LDA (0.38 M in THF, 8.2 mL, 3.1 mmol, 2.6 equiv) was added dropwise to a cooled solution (–78 °C) of **S23** (105 mg, 1.2 mmol, 1.0 equiv) in anhydrous THF (1.0 mL). After 1 h stirring at –78 °C, HMPA (1.42 g/1.37 mL, 7.9 mmol, 6.6 equiv) was added. The reaction mixture was stirred at the same temperature for another 50 min before the addition of CH_3I (682 mg/0.3 mL, 4.8 mmol, 4.0 equiv). The reaction mixture was warmed to room temperature and stirred for 5 h before being quenched with sat. aq. NH_4Cl (50 mL) and extracted with EtOAc (50 mL×3). The combined organic phases were washed with brine, dried over Na_2SO_4, filtrated, and concentrated under reduced pressure to give light yellow oil which was used directly for the next step without further purification.

Freshly prepared LDA (0.8 M in THF, 3 mL, 2.4 mmol, 2.0 equiv) was added dropwise to a cooled solution (–78 °C) of above crude product (assumed 1.2 mmol) in anhydrous THF (1 mL). The resulting mixture was stirred at the same temperature for 1 h before the addition of HMPA (1.3 g/1.25 mL, 7.2 mmol, 6.0 equiv). After stirring at –78 °C for 1 h, CH_3I (682 mg/0.3 mL, 4.8 mmol, 4.0 equiv) was added. The reaction mixture was warmed to room temperature and stirred overnight. The reaction mixture was quenched with sat. aq. NH_4Cl (50 mL) and extracted with Et_2O (50 mL×3). The combined organic phases were washed with brine, dried

over Na_2SO_4, filtrated, and concentrated under reduce pressure. Purification by column chromatography (silica gel, Petroleum ether:EtOAc, 10 :1) gave **S24** (205 mg, 61% over 2 steps) as colorless oil.

TLC (silica gel, 1:5, EtOAc: Petroleum ether): $R_f = 0.6$ (UV, $KMnO_4$).

^{1}H NMR (400 MHz, $CDCl_3$): δ 6.62 (d, J = 10.2 Hz, 1H), 5.88 (d, J = 10.2 Hz, 1H), 3.40 – 3.34 (m, 2H), 1.89 (d, J = 14.2 Hz, 1H), 1.60 – 1.52 (m, 1H), 1.20 (s, 3H), 1.18 (s, 3H), 1.13 (s, 3H), 0.89 (s, 9H), 0.03 (s, 6H).

^{13}C NMR (101 MHz, $CDCl_3$): δ 204.9, 154.2, 126.6, 71.4, 44.0, 40.9, 38.5, 28.0, 27.1, 25.8, 24.2, 18.3, –5.5.

HRMS (ESI, m/z): cacl'd for $C_{19}H_{31}O_2Si$ $[M+H]^+$: 283.2088, found: 283.2082.

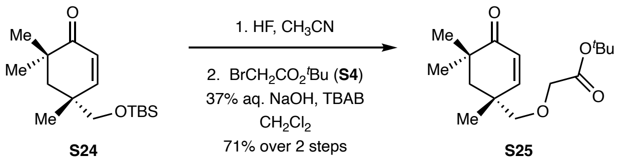

S24 S25

To the solution of **S24** (175 mg, 0.62 mmol) in CH_3CN (6.3 mL) was added aq. 49% HF (0.63 mL) dropwise at room temperature. After 1 h stirring, the reaction was quenched slowly with saturated aq. $NaHCO_3$ (40 mL), extracted with EtOAc (40 mL×3). The combined organic phases were washed with brine, dried over Na_2SO_4, filtrated, and concentrated under reduced pressure. The residue was used directly for the next step without further purification.

According to the procedure of the preparation of **17**, **S25** (125 mg, 71% over 2 steps) was prepared from the above crude product (assumed 0.62 mmol) (column chromatography: silica gel, Petroleum ether:EtOAc, 10:1).

TLC (silica gel, 1:4, EtOAc:Petroleum ether): $R_f = 0.5$ (UV, $KMnO_4$).

^{1}H NMR (400 MHz, $CDCl_3$): δ 6.68 (d, J = 10.2 Hz, 1H), 5.91 (d, J = 10.2 Hz, 1H), 3.96 (s, 2H), 3.40 – 3.29 (m, 2H), 2.03 (d, J = 14.4 Hz, 1H), 1.66 – 1.61 (m, 1H), 1.48 (s, 9H), 1.26 (s, 3H), 1.21 (s, 3H), 1.15 (s, 3H).

^{13}C NMR (101 MHz, $CDCl_3$): δ 204.5, 169.5, 153.4, 126.8, 81.7, 79.9, 69.2, 44.3, 40.9, 37.6, 28.2, 28.0, 27.2, 24.5.

HRMS (ESI, m/z): cacl'd for $C_{16}H_{26}O_4Na$ $[M+Na]^+$: 305.1723, found: 305.1720.

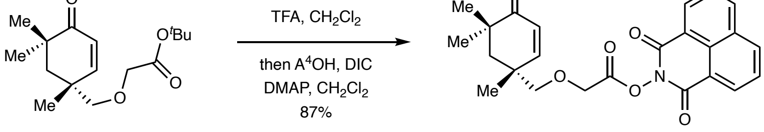

S25 S26

On 1.4 mmol scale, **General procedure A** was followed with **S25** and *N*-hydroxynaphthalimide to give the product **S26** (519 mg, 87%) as white amorphous powder (column chromatography: silica gel, CH_2Cl_2:EtOAc, 10:1).

TLC (silica gel, 1:2, EtOAc:Petroleum ether): R_f = 0.2 (UV, $KMnO_4$).

^{1}H NMR (400 MHz, CDCl$_3$): δ 8.64 (dd, *J* = 7.3, 1.1 Hz, 2H), 8.30 (dd, *J* = 8.2, 1.2 Hz, 2H), 7.81 (dd, *J* = 8.2, 7.3 Hz, 2H), 6.70 (d, *J* = 10.2 Hz, 1H), 5.91 (d, *J* = 10.2 Hz, 1H), 4.60 (d, *J* = 1.6 Hz, 2H), 3.57 (d, *J* = 8.3 Hz, 1H), 3.52 (d, *J* = 8.3 Hz, 1H), 2.07 (d, *J* = 14.3 Hz, 1H), 1.70 – 1.63 (m, 1H), 1.29 (s, 3H), 1.21 (s, 3H), 1.16 (s, 3H).

^{13}C NMR (101 MHz, CDCl$_3$): δ 204.4, 166.8, 159.4, 153.0, 135.2, 132.2, 131.9, 127.7, 127.2, 127.0, 122.2, 80.0, 66.7, 44.2, 40.9, 37.6, 27.9, 27.2, 24.6.

HRMS (ESI, m/z): cacl'd for $C_{24}H_{24}NO_6$ $[M+H]^+$: 422.1598, found: 422.1588.

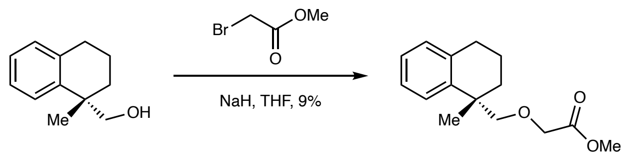

S28 was prepared according to Li's method[9].

NaH (60% dispersion in mineral oil, 318 mg, 7.9 mmol, 1.4 equiv) was added slowly to the solution of **S28** (1.0 g, 5.7 mmol, 1.0 equiv) in anhydrous THF (10 mL) at 0 °C. The resulting mixture was stirred at 0 °C for 1 h before the addition of methyl bromoacetate (1.13 g/0.7 mL, 7.4 mmol, 1.3 equiv). The reaction mixture was warmed to room temperature and stirred for another 4 h. The reaction mixture was cooled to 0 °C, quenched with H_2O (50 mL), and extracted with EtOAc (50 mL×3). The combined organic phases were washed with brine, dried over Na_2SO_4, filtrated, and concentrated under reduced pressure. Purification by column chromatography (silica gel, Petroleum ether:EtOAc, 10:1) gave **S29** (130 mg, 9%) as colorless oil.

TLC (silica gel, 1:3, EtOAc:Petroleum ether): R_f = 0.8 ($KMnO_4$).

^{1}H NMR (400 MHz, CDCl$_3$) δ 7.34 – 7.32 (m, 1H), 7.15 – 7.06 (m, 3H), 4.07 (d, *J* = 3.7 Hz, 2H), 3.73 (s, 3H), 3.63 (d, *J* = 8.9 Hz, 1H), 3.48 (d, *J* = 8.9 Hz, 1H), 2.78 (t, *J* = 6.4 Hz, 2H), 2.11 – 2.05 (m, 1H), 1.87 – 1.75 (m, 2H), 1.61 – 1.54 (m, 1H), 1.33 (s, 3H).

^{13}C NMR (101 MHz, CDCl$_3$) δ 171.2, 141.7, 137.4, 129.3, 126.9, 125.9, 125.7, 80.2, 68.7, 51.7, 38.2, 33.4, 30.6, 26.7, 19.3.

HRMS (ESI, m/z): cacl'd for $C_{15}H_{24}O_3N$ $[M+NH_4]^+$: 266.1750, found: 266.1746.

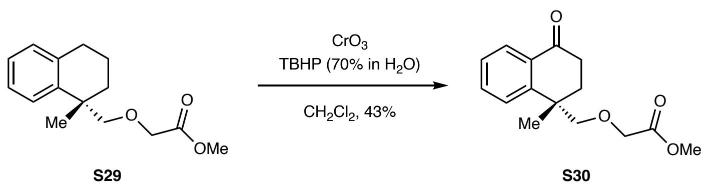

To a cooled solution (0 °C) of **S29** (520 mg, 2.1 mmol, 1.0 equiv) in CH_2Cl_2 (12 mL), TBHP (70% in H_2O, 3.1 mL) and CrO_3 (290 mg, 2.9 mmol, 1.4 equiv) were added successively. The reaction mixture was warmed to room temperature and stirred for 2 h before being diluted with H_2O (20 mL). The organic phase was separated, and the aqueous phase was extracted with CH_2Cl_2 (20 mL×3). The combined organic phases were washed with brine, dried over Na_2SO_4, filtrated, and concentrated. Purification by column chromatography (silica gel, Petroleum ether:EtOAc, 5:1) gave **S30** (239 mg, 43%) as colorless oil.

TLC (silica gel, 1:3, EtOAc:Petroleum ether): R_f = 0.4 (UV, $KMnO_4$).

^{1}H NMR (400 MHz, CDCl$_3$) δ 8.06 – 8.03 (m, 1H), 7.54 – 7.52 (m, 1H), 7.47 – 7.45 (m, 1H), 7.35 – 7.31 (m, 1H), 4.11 – 4.01 (m, 2H), 3.75 (d, J = 9.0 Hz, 1H), 3.72 (s, 3H), 3.51 (d, J = 9.0 Hz, 1H), 2.92 – 2.82 (m, 1H), 2.74 – 2.66 (m, 1H), 2.41 – 2.35 (m, 1H), 2.00 – 1.93 (m, 1H), 1.43 (s, 3H).

^{13}C NMR (101 MHz, CDCl$_3$) δ 198.3, 170.8, 148.1, 133.6, 132.2, 127.5, 126.9, 126.4, 78.6, 68.5, 51.8, 38.3, 34.7, 31.9, 25.0.

HRMS (ESI, m/z): cacl'd for $C_{15}H_{19}O_4$ $[M+H]^+$: 263.1278, found: 263.1273.

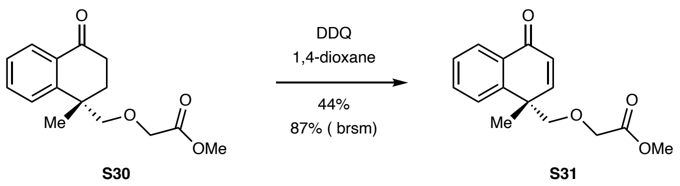

To a solution of **S30** (237 mg, 0.9 mmol, 1.0 equiv) in 1,4-dioxane (4 mL) was added 2,3-dichloro-5.6-dicyano-1.4-benzoquinone (DDQ, 286 mg, 1.3 mmol, 1.4 equiv). After heating at 100 °C for 18 h, the reaction mixture was cooled to room temperature and quenched with sat. aq. $NaHCO_3$ (20 mL). The resulting mixture was extracted with CH_2Cl_2 (20 mL×3). The combined organic phases were washed with brine, dried over Na_2SO_4, filtrated, and concentrated under reduced pressure. Purification by column chromatography (silica gel, Petroleum ether:EtOAc, 3:1) gave **S31** (103 mg, 44%) and **S30** (118 mg, 50%).

TLC (silica gel, 1:3, EtOAc: Petroleum ether): R_f = 0.5 (UV, $KMnO_4$).

^{1}H NMR (400 MHz, CDCl$_3$): δ 8.20 – 8.18 (m, 1H), 7.60 – 7.53 (m, 2H), 7.43 – 7.39 (m, 1H), 7.08 (d, J = 10.3 Hz, 1H), 6.49 (d, J = 10.3 Hz, 1H), 4.03 – 3.94 (m, 2H), 3.82 (d, J = 9.0 Hz, 1H), 3.73 – 3.68 (m, 4H), 1.50 (s, 3H).

^{13}C NMR (101 MHz, CDCl$_3$): δ 184.9, 170.5, 154.0, 146.0, 132.5, 131.6, 128.4, 127.2, 127.0, 126.2, 79.3, 68.6, 51.8, 42.2, 24.4.

HRMS (ESI, m/z): cacl'd for $C_{15}H_{16}O_4Na$ $[M+Na]^+$: 283.0941, found: 283.0935.

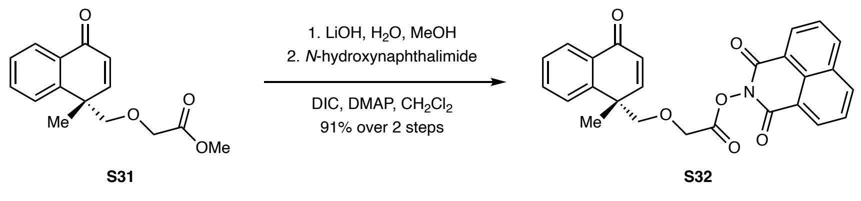

To a solution of **S31** (105 mg, 0.4 mmol, 1.0 equiv) in MeOH (4 mL) was added H_2O (2 mL) and LiOH (49 mg, 2.0 mmol, 5.0 equiv) successively at room temperature. The resulting mixture was stirred at room temperature for 1 h before being acidified with 0.1 N HCl (30 mL). The resulting mixture was extracted with EtOAc (30 mL×3). The combined organic phases were washed with brine, dried over Na_2SO_4, filtrated, and concentrated under reduce pressure. The residue was used directly for the next step without further purification.

To the mixture of above carboxylic acid (assumed 0.4 mmol, 1.0 equiv), *N*-hydroxynaphthalimide (94 mg, 0.44 mmol, 1.1 equiv), and DMAP (15 mg, 0.12 mmol, 0.3 equiv) in anhydrous CH_2Cl_2 (5 mL) was added DIC (61 mg/74 μL, 0.48 mmol, 1.2 equiv). The reaction mixture was stirred at room temperature for 3 h before being concentrated under reduced pressure. Purified by column chromatography (silica gel, CH_2Cl_2:EtOAc, 10:1) gave **S32** (161 mg, 91% over 2 steps) as white amorphous powder.

TLC (silica gel, 1:1, EtOAc:Petroleum ether): R_f = 0.5 (UV, $KMnO_4$).

^{1}H NMR (400 MHz, CDCl$_3$) δ 8.65 (dd, J = 7.3, 1.2 Hz, 2H), 8.30 (dd, J = 8.4, 1.1 Hz, 2H), 8.25 – 8.17 (m, 1H), 7.82 (dd, J = 8.2, 7.3 Hz, 2H), 7.63 – 7.57 (m, 2H), 7.45 – 7.41 (m, 1H), 7.12 (d, J = 10.3 Hz, 1H), 6.51 (d, J = 10.3 Hz, 1H), 4.55 (d, J = 17.1 Hz, 1H) , 4.47 (d, J = 17.1 Hz, 1H), 4.00 (d, J = 8.7 Hz, 1H), 3.88 (d, J = 8.7 Hz, 1H), 1.55 (s, 3H).

^{13}C NMR (101 MHz, CDCl$_3$) δ 184.8, 166.6, 159.4, 153.7, 145.8, 135.3, 132.6, 132.2, 131.9, 131.6, 128.5, 127.6, 127.3, 127.2, 127.0, 126.3, 122.1, 79.2, 66.8, 42.1, 24.5.

HRMS (ESI, m/z): cacl'd for $C_{26}H_{19}NO_6Na$ $[M+Na]^+$: 464.1104, found: 464.1095.

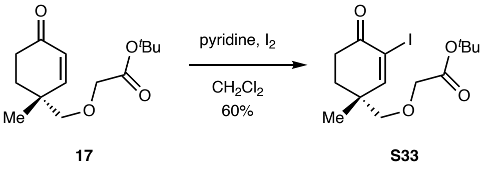

Pyridine (1.6 mL, 19.5 mmol, 5.0 equiv) was added dropwise to a solution of **17** (1.0 g, 3.9 mmol, 1.0 equiv) and I_2 (2.2 g, 8.6 mmol, 2.2 equiv) in CH_2Cl_2 (40 mL). After 2 h stirring at

room temperature, the reaction mixture was quenched with sat. aq. $Na_2S_2O_3$ (40 mL), extracted with CH_2Cl_2 (40 mL×3). The combined organic phases were washed with brine, dried over Na_2SO_4, filtrated, and concentrated under reduced pressure. Purification by column chromatography (silica gel, Petroleum ether:EtOAc, 5:1) gave **S33** (896 mg, 60%) as yellow oil.

TLC (silica gel, 1:2, EtOAc:Petroleum ether): R_f = 0.7 (UV, $KMnO_4$).

^{1}H NMR (400 MHz, CDCl$_3$): δ 7.58 (s, 1H), 3.96 (s, 2H), 3.50 – 3.35 (m, 2H), 2.84 – 2.61 (m, 2H), 2.26 – 2.13 (m, 1H), 1.89 – 1.76 (m, 1H), 1.48 (s, 9H), 1.21 (s, 3H).

^{13}C NMR (101 MHz, CDCl$_3$): δ 192.0, 169.4, 164.3, 103.8, 81.9, 77.7, 69.0, 42.4, 33.1, 31.5, 28.2, 22.2.

HRMS (ESI, m/z): cacl'd for $C_{14}H_{21}IO_4$ $[M+Na]^+$: 403.0377, found: 403.0372.

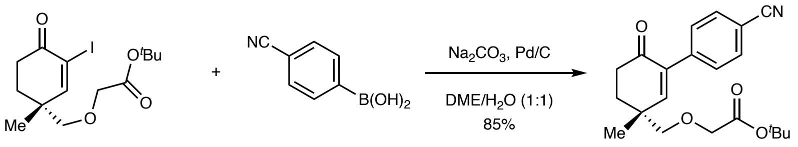

S33 **S34**

Pd/C (19 mg, 5% w.t.) was added in one portion to a mixture of **S33** (380 mg, 1.0 mmol, 1.0 equiv), 4-cyanophenylboronic acid (294 mg, 2.0 mmol, 2.0 equiv), and Na_2CO_3 (222 mg, 2.0 mmol, 2.0 equiv) in DME/H_2O (1:1, 11 mL). After stirring at room temperature overnight, the reaction mixture was diluted with H_2O (20 mL), extracted with EtOAc (20 mL×3). The combined organic phases were washed with brine, dried over Na_2SO_4, filtrated, and concentrated under reduced pressure. Purification by column chromatography (silica gel, Petroleum ether:EtOAc, 2:1) gave **S34** (302 mg, 85%) as yellow oil.

TLC (silica gel, 1:2, EtOAc:Petroleum ether): R_f = 0.7 (UV).

^{1}H NMR (400 MHz, CDCl$_3$): δ 7.62 – 7.60 (m, 2H), 7.46 – 7.44 (m, 2H), 6.94 (s, 1H), 3.98 (s, 2H), 3.55 – 3.45 (m, 2H), 2.70 – 2.65 (m, 2H), 2.31 – 2.22 (m, 1H), 1.88 – 1.81 (m, 1H), 1.47 (s, 9H), 1.28 (s, 3H).

^{13}C NMR (101 MHz, CDCl$_3$): δ 197.1, 169.6, 155.4, 141.1, 137.7, 131.8, 129.7, 119.0, 111.4, 82.0, 78.7, 69.0, 38.3, 34.8, 31.3, 28.3, 22.6.

HRMS (ESI, m/z): cacl'd for $C_{21}H_{25}NO_4Na$ $[M+Na]^+$: 378.1676, found: 378.1668.

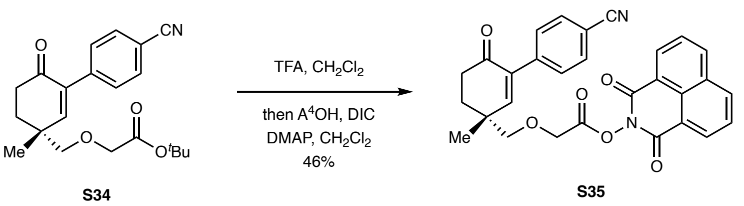

On 0.5 mmol scale, **General procedure A** was followed with **S34** and *N*-hydroxynaphthalimide to give the product **S35** (116 mg, 46%) as white amorphous powder (column chromatography: silica gel, CH_2Cl_2:EtOAc, 10:1).

TLC (silica gel, 1:2, EtOAc:Petroleum ether): R_f = 0.4 (UV, $KMnO_4$).

^{1}H NMR (400 MHz, $CDCl_3$): δ 8.57 (dd, *J* = 7.3, 1.2 Hz, 2H), 8.26 (dd, *J* = 8.3, 1.1 Hz, 2H), 7.76 (dd, *J* = 8.2, 7.3 Hz, 2H), 7.56 – 7.50 (m, 2H), 7.42 (d, *J* = 8.3 Hz, 2H), 6.96 (s, 1H), 4.61 (s, 2H), 3.77 – 3.64 (m, 2H), 2.81 – 2.54 (m, 2H), 2.37 – 2.23 (m, 1H), 1.93 – 1.76 (m, 1H), 1.31 (s, 3H).

^{13}C NMR (101 MHz, $CDCl_3$): δ 197.0, 166.9, 159.4, 154.9, 141.0, 137.7, 135.4, 132.1, 131.9, 131.8, 129.6, 127.5, 127.2, 122.0, 118.9, 111.2, 78.9, 66.7, 38.2, 34.7, 31.1, 22.5.

HRMS (ESI, m/z): cacl'd for $C_{29}H_{23}N_2O_6$ $[M+H]^+$: 495.1551, found: 495.1544.

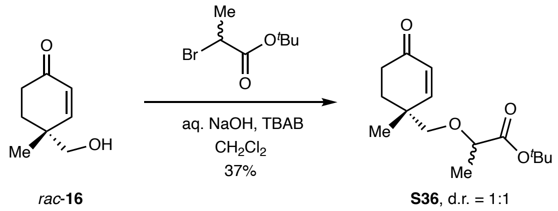

To a cooled (0 °C) solution of *rac*-**16** (140 mg, 1.0 mmol, 1.0 equiv), *tert*-butyl 2-bromopropionate (420 mg/0.33 mL, 2.0 mmol, 2.0 equiv) and tetrabutylammonium bromide (TBAB, 322 mg, 1.0 mmol, 1.0 equiv) in CH_2Cl_2 (1.5 mL) was added aq. NaOH (37%, 1.5 mL). The reaction mixture was stirred at room temperature for 4 h before being diluted with H_2O (10 mL). The resulting mixture was extracted with CH_2Cl_2 (10 mL×3). The combined organic layers were washed with brine, dried over Na_2SO_4, filtrated, and concentrated under reduced pressure. Purification by column chromatography (silica gel, Petroleum ether:EtOAc, 10:1) gave **S36** (99 mg, 37%, d.r. = 1:1) as colorless oil.

TLC (silica gel, 1:4, EtOAc:Petroleum ether): R_f = 0.5 (UV, $KMnO_4$).

^{1}H NMR (500 MHz, $CDCl_3$): δ 6.77 (dd, *J* = 10.2, 7.8 Hz, 2H), 5.93 (dd, *J* = 10.2, 1.8 Hz, 2H), 3.79 (q, *J* = 6.8 Hz, 2H), 3.52 (d, *J* = 8.6 Hz, 1H), 3.45 (d, *J* = 8.6 Hz, 1H), 3.23 (d, *J* = 8.7 Hz, 1H), 3.17 (d, *J* = 8.5 Hz, 1H), 2.56 – 2.44 (m, 4H), 2.16 – 2.08 (m, 2H), 1.78 – 1.71 (m, 2H), 1.46 (s, 9H), 1.46 (s, 9H), 1.34 (t, *J* = 6.5 Hz, 6H), 1.17 (s, 3H) 1.16 (s, 3H).

^{13}C NMR (126 MHz, CDCl$_3$): δ 199.6, 199.5, 172.3, 172.2, 156.1, 156.0, 128.51, 128.49, 81.3, 76.9, 76.5, 75.8, 75.7, 37.23, 37.20, 34.0, 33.9, 31.5, 31.3, 28.01, 28.00, 22.45, 22.40, 18.43, 18.41.

HRMS (ESI, m/z): cacl'd for $C_{15}H_{24}O_4Na$ [M+Na]$^+$: 291.1567, found: 291.1569

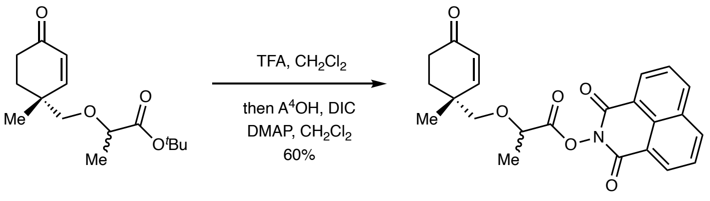

S36 **S37**, d.r. = 1:1

On 0.67 mmol scale, **General procedure A** was followed with **S36** and *N*-hydroxynaphthalimide to give the product **S37** (165 mg, 60%, d.r. = 1:1) as white amorphous powder (column chromatography: silica gel, CH_2Cl_2:EtOAc, 10:1).

TLC (silica gel, 1:10, EtOAc: CH_2Cl_2): R_f = 0.6 (UV, $KMnO_4$).

^{1}H NMR (500 MHz, CDCl$_3$): δ 8.63 (t, J = 6.5 Hz, 4H), 8.29 (d, J = 8.4 Hz, 4H), 7.81 (t, J = 7.8 Hz, 4H), 6.84 – 6.82 (m, 2H), 5.96 (d, J = 10.2 Hz, 2H), 4.44 (qd, J = 6.8, 2.7 Hz, 2H), 3.83 (d, J = 8.5 Hz, 1H), 3.78 (d, J = 8.7 Hz, 1H), 3.48 (d, J = 8.7 Hz, 1H), 2.61 – 2.45 (m, 4H), 2.25 – 2.17 (m, 2H), 1.85 – 1.77 (m, 2H), 1.72 (dd, J = 6.8, 5.7 Hz, 6H), 1.24 (s, 3H), 1.23 (s, 3H).

^{13}C NMR (126 MHz, CDCl$_3$): δ 199.59, 199.56, 169.4, 159.4, 155.8, 155.7, 135.2, 132.03, 131.99, 131.87, 128.7, 127.6, 127.1, 122.1, 77.3, 76.9, 74.14, 74.13, 37.29, 37.28, 34.0, 33.9, 31.4, 31.2, 22.5, 22.4, 18.8.

HRMS (ESI, m/z): cacl'd for $C_{23}H_{22}NO_6$ [M+H]$^+$: 408.1442, found: 408.1444.

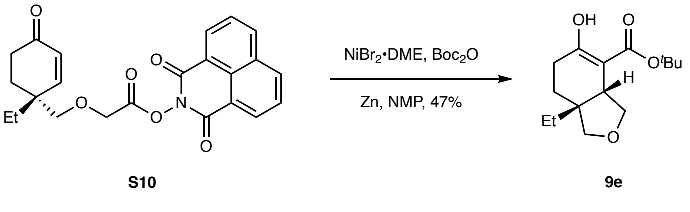

S10 **9e**

On 0.15 mmol scale, **General procedure B** was followed with **S10** to give the product **9e** (19.0 mg, 47%) as colorless oil (column chromatography: silica gel, Petroleum:EtOAc, 10:1).

TLC (silica gel, 1:4, EtOAc:Petroleum ether): R_f = 0.7 (UV, $KMnO_4$).

^{1}H NMR (400 MHz, CDCl$_3$): δ 12.44 (s, 1H), 4.20 (t, J = 8.4 Hz, 1H), 3.63 (d, J = 2.1 Hz, 2H), 3.50 (t, J = 8.4 Hz, 1H), 2.64 (t, J = 8.4 Hz, 1H), 2.36 – 2.17 (m, 2H), 1.78 – 1.59 (m, 3H), 1.49 (s, 9H), 1.33 (dd, J = 14.2, 7.3 Hz, 1H), 0.86 (t, J = 7.6 Hz, 3H).

^{13}C NMR (101 MHz, CDCl$_3$): δ 172.5, 171.5, 98.7, 81.4, 78.1, 75.0, 43.9, 43.4, 28.3, 27.7, 26.0, 24.5, 9.2.

HRMS (ESI, m/z): cacl'd for $C_{15}H_{24}O_4Na$ [M+Na]$^+$: 291.1567, found: 291.1561.

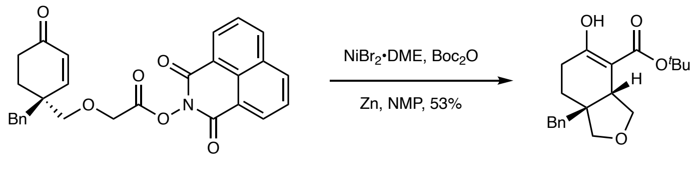

S14 **9f**

On 0.15 mmol scale, **General procedure B** was followed with **S14** to give the product **9f** (26.2 mg, 53%) as colorless oil (column chromatography: silica gel, Petroleum:EtOAc, 10:1).

^{1}H NMR (400 MHz, CDCl$_3$): δ 12.49 (s, 1H), 7.31 – 7.26 (m, 2H), 7.26 – 7.21 (m, 1H), 7.15 – 7.04 (m, 2H), 4.21 (t, J = 8.5 Hz, 1H), 3.86 (d, J = 8.4 Hz, 1H), 3.54– 3.49 (m, 2H), 2.86 – 2.78 (m, 2H), 2.67 (d, J = 13.8 Hz, 1H), 2.56 – 2.44 (m, 1H), 2.31 – 2.22 (m, 1H), 1.78 – 1.70 (m, 1H), 1.58– 1.56 (m, 1H), 1.51 (s, 9H).

^{13}C NMR (101 MHz, CDCl$_3$): δ 172.4, 171.2, 138.0, 129.7, 128.3, 126.5, 98.6, 81.6, 77.6, 74.8, 44.2, 43.2, 40.8, 28.3, 26.1, 24.7.

HRMS (ESI, m/z): cacl'd for $C_{20}H_{26}O_4Na$ [M+Na]$^+$: 353.1723, found: 353.1715.

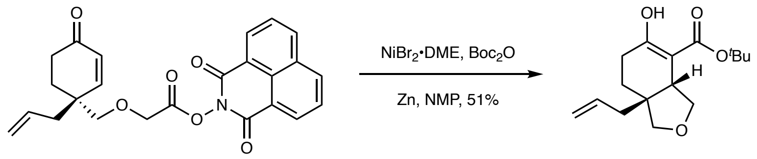

S18 **9g**

On 0.15 mmol scale, **General procedure B** was followed with **S18** to give the product **9g** (21.5 mg, 51%) as colorless oil (column chromatography: silica gel, Petroleum ether:EtOAc, 10:1).

TLC (silica gel, 1:4, EtOAc:Petroleum ether): R_f = 0.6 (UV, KMnO$_4$).

^{1}H NMR (400 MHz, CDCl$_3$): δ 12.46 (s, 1H), 5.76 – 5.63 (m, 1H), 5.14 – 5.03 (m, 2H), 4.20 (t, J = 8.4 Hz, 1H), 3.70 (d, J = 8.5 Hz, 1H), 3.60 (d, J = 8.5 Hz, 1H), 3.49 (t, J = 8.4 Hz, 1H), 2.69 (t, J = 8.4 Hz, 1H), 2.39 – 2.29 (m, 2H), 2.27 – 2.18 (m, 1H), 2.10 – 2.02 (m, 1H), 1.78 – 1.61 (m, 1H), 1.67 – 1.60 (m, 1H), 1.49 (s, 9H).

^{13}C NMR (101 MHz, CDCl$_3$): δ 172.5, 171.5, 134.2, 118.0, 98.6, 81.5, 77.9, 74.9, 43.4, 43.0, 39.8, 28.3, 25.8, 25.4.

HRMS (ESI, m/z): cacl'd for $C_{16}H_{24}O_4Na$ [M+Na]$^+$: 303.1567, found: 303.1559.

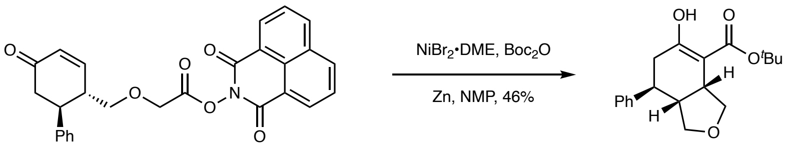

S22 **9h**

On 0.15 mmol scale, **General procedure B** was followed with **S22** to give the product **9h** (21.8 mg, 46%) as colorless oil (column chromatography: silica gel, Petroleum ether:EtOAc, 20:1).

TLC (silica gel, 1:9, EtOAc:Petroleum ether): R_f = 0.4 (UV, $KMnO_4$).

^{1}H NMR (400 MHz, $CDCl_3$): δ 12.49 (s, 1H), 7.36 – 7.30 (m, 2H), 7.26 – 7.21 (m, 1H), 7.22 – 7.17 (m, 2H), 4.24 (t, *J* = 8.2 Hz, 1H), 3.72 (dd, *J* = 9.0, 5.5 Hz, 1H), 3.58 – 3.51 (m, 2H), 3.24 – 3.15 (m, 1H), 2.87 (td, *J* = 11.8, 5.3 Hz, 1H), 2.60 – 2.41 (m, 3H), 1.52 (s, 9H).

^{13}C NMR (101 MHz, $CDCl_3$): δ 172.2, 171.8, 142.8, 129.3, 127.8, 126.9, 98.5, 81.7, 73.8, 71.3, 43.4, 40.0, 38.7, 37.2, 28.3.

HRMS (ESI, m/z): cacl'd for $C_{19}H_{24}O_4Na$ $[M+Na]^+$: 339.1567, found: 339.1569.

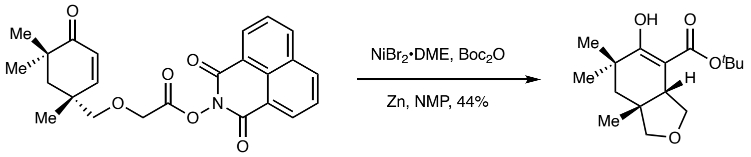

S26 **9i**

On 0.15 mmol scale, **General procedure B** was followed with **S26** to give the product **9i** (18.6 mg, 44%) as colorless oil (column chromatography: silica gel, Petroleum ether:EtOAc, 20:1).

TLC (silica gel, 1:9, EtOAc:Petroleum ether): R_f = 0.5 (UV, $KMnO_4$).

^{1}H NMR (400 MHz, $CDCl_3$): δ 12.75 (s, 1H), 4.19 (t, *J* = 8.5 Hz, 1H), 3.63 (d, *J* = 8.3 Hz, 1H), 3.51 – 3.44 (m, 2H), 2.74 (td, *J* = 8.6, 1.6 Hz, 1H), 1.87 (d, *J* = 14.1 Hz, 1H), 1.49 (s, 9H), 1.43 (d, *J* = 13.6 Hz, 1H), 1.28 (s, 3H), 1.20 (s, 3H), 1.15 (s, 3H).

^{13}C NMR (101 MHz, $CDCl_3$) δ 177.4, 173.2, 96.6, 81.9, 81.4, 75.7, 45.0, 44.6, 39.6, 35.6, 30.3, 28.3, 27.8, 23.9.

HRMS (ESI, m/z): cacl'd for $C_{16}H_{26}O_4Na$ $[M+Na]^+$: 305.1723, found: 305.1719.

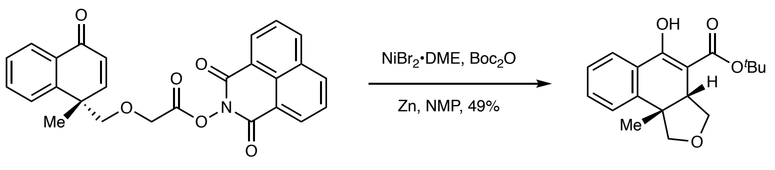

S32 **9j**

On 0.15 mmol scale, **General procedure B** was followed with **S32** to give the product **9j** (22.2 mg, 49%) as colorless oil (column chromatography: silica gel, Petroleum ether:EtOAc, 20:1).

TLC (silica gel, 1:9, EtOAc:Petroleum ether): R_f = 0.5 (UV, $KMnO_4$).

^{1}H NMR (400 MHz, $CDCl_3$): δ 12.75 (s, 1H), 7.89 (dd, *J* = 8.2, 1.5 Hz, 1H), 7.48 – 7.41 (m, 1H), 7.26 – 7.33 (m, 2H), 4.33 (d, *J* = 8.7 Hz, 1H), 4.27 (t, *J* = 8.0 Hz, 1H), 3.97 (d, *J* = 8.7 Hz, 1H), 3.31 (dd, *J* = 9.8, 8.1 Hz, 1H), 3.08 (dd, *J* = 9.8, 7.9 Hz, 1H), 1.56 (s, 9H), 1.33 (s, 3H).

^{13}C NMR (101 MHz, $CDCl_3$): δ 172.8, 162.9, 143.2, 131.5, 127.7, 126.8, 126.2, 124.7, 95.9, 81.9, 80.6, 75.2, 44.9, 44.5, 28.4, 28.1.

HRMS (ESI, m/z): cacl'd for $C_{18}H_{22}O_4Na$ $[M+Na]^+$: 325.1410, found: 325.1407.

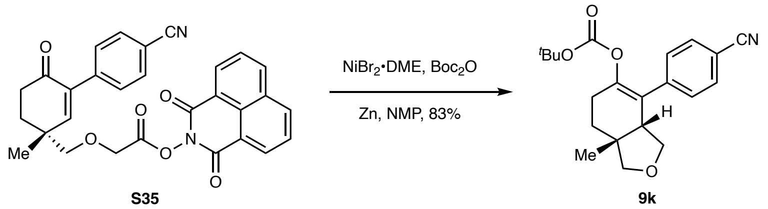

S35 **9k**

On 0.15 mmol scale, **General procedure B** was followed with **S35** to give the product **9k** (44.3 mg, 83%) as colorless oil (column chromatography: silica gel, Petroleum ether:EtOAc, 5:1).

TLC (silica gel, 1:2, EtOAc:Petroleum ether): R_f = 0.5 (UV, $KMnO_4$).

^{1}H NMR (400 MHz, $CDCl_3$): δ 7.68 – 7.56 (m, 2H), 7.38 – 7.26 (m, 2H), 3.91 (t, *J* = 8.4 Hz, 1H), 3.73 (d, *J* = 8.4 Hz, 1H), 3.62 (d, *J* = 8.4 Hz, 1H), 3.60 – 3.55 (m, 1H), 2.79 (t, *J* = 8.3 Hz, 1H), 2.48 – 2.30 (m, 2H), 2.05 – 1.96 (m, 1H), 1.65 (dd, *J* = 13.5, 7.9 Hz, 1H), 1.33 (s, 9H), 1.21 (s, 3H).

^{13}C NMR (101 MHz, $CDCl_3$): δ 150.8, 145.2, 143.0, 132.2, 129.0, 123.9, 118.9, 111.1, 83.3, 79.9, 72.9, 50.3, 40.4, 29.6, 27.5, 24.8, 22.2.

HRMS (ESI, m/z): cacl'd for $C_{21}H_{26}NO_4$ $[M+H]^+$: 356.1857, found: 356.1851.

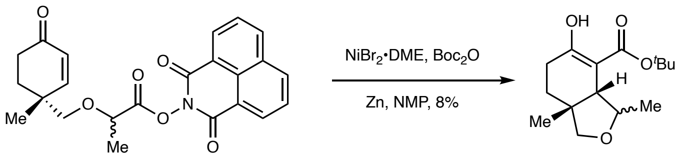

S37 **S44**, d.r.= 4:1

On 0.15 mmol scale, **General procedure B** was followed with **S37** to give the product **S44** (3.2 mg, 8%, d.r. = 4:1) as colorless oil (column chromatography: silica gel, Petroleum ether:EtOAc, 20:1).

TLC (silica gel, 1:10, EtOAc:Petroleum ether): R_f = 0.6 (UV, $KMnO_4$).

^{1}H NMR (500 MHz, $CDCl_3$, both diastereomers**):** δ 12.85 (s, 0.25H), 12.54 (s, 1H), 4.34 – 4.28 (m, 1H), 3.74 – 3.69 (m, 0.25H), 3.65 (d, J = 8.2 Hz, 0.25H), 3.60 – 3.58 (m, 1.25H), 3.47 (d, J = 8.5 Hz, 1H), 2.75 (d, J = 9.2 Hz, 1H), 2.40 – 2.31 (m, 1.75H), 2.30 – 2.24 (m, 1H), 1.98 – 1.93 (m, 0.25H), 1.91– 1.84 (m, 1H), 1.53 (s, 2.25H), 1.50 (s, 9H), 1.45 – 1.41 (m, 1.25H), 1.32 (d, J = 6.1 Hz, 0.75H), 1.05 (s, 0.75H), 1.03 (d, J = 6.8 Hz, 6H).

^{13}C NMR (101 MHz, $CDCl_3$): δ172.8, 172.6, 172.5, 171.9, 97.4, 96.9, 82.9, 81.8, 81.4, 78.7, 78.3, 78.1, 50.3, 46.4, 41.2, 40.4, 29.0, 28.96, 28.3, 28.2, 26.6, 26.1, 22.2, 21.4, 21.2, 19.6.

HRMS (ESI, m/z): cacl'd for $C_{15}H_{24}O_4Na$ $[M+Na]^+$: 291.1567, found: 291.1569.

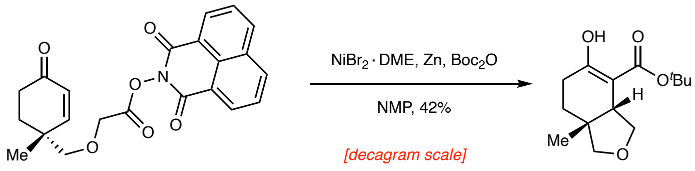

11d **9d**

*Decagram scale synthesis of **9d** was conducted according to **General Procedure B** with slight modification.*

In a glovebox, di-*tert*-butyl-dicarbonate (Boc_2O, 7.0 g/7.35 mL, 31.88 mmol, 6.0 equiv) was added to a mixture of **11d** (12.0 g, 30.5 mmol, 1.0 equiv), $NiBr_2$•DME (940 mg, 3.05 mmol, 0.1 equiv), and Zn (6.0 g, 91.5 mmol, 3.0 equiv) in anhydrous *N*-mehtyl-2-pyrrolidone (NMP, 430 mL, 0.07 mM) at room temperature. The reaction mixture was moved out of glove box and warmed to 50 °C. After 14 h stirring, the reaction mixture was filtrated through celite, washed with EtOAc (1000 mL). The filtrate was washed with H_2O (1000 mL×3) and brine (1000 mL ×1) whereby the aqueous layers were back-extracted with EtOAc (1000 mL×3). The combined organic layers were dried over Na_2SO_4, filtrated, and concentrated under reduced pressure. Purified by column chromatography (silica gel, Petroleum ether:EtOAc, 50:1 to 20:1) gave **9d** (3.25 g, 42%) as light yellow oil.

TLC (silica gel, EtOAc:Petroleum ether, 1:5): R_f = 0.4 (UV, $KMnO_4$).

^{1}H NMR (400 MHz, CDCl$_3$): δ 12.44 (s, 1H), 4.22 (t, J = 8.4 Hz, 1H), 3.64 (d, J = 8.3 Hz, 1H), 3.56 (d, J = 8.3 Hz, 1H), 3.50 (t, J = 8.3 Hz, 1H), 2.65 (t, J = 8.4 Hz, 1H), 2.44 – 2.30 (m, 1H), 2.32 – 2.19 (m, 1H), 1.85 (m, 1H), 1.49 (s, 9H), 1.48 – 1.42 (m, 1H), 1.06 (s, 3H).

^{13}C NMR (101 MHz, CDCl$_3$): δ 172.7, 171.4, 98.9, 81.6, 80.1, 75.4, 44.2, 40.0, 28.6, 28.4, 26.4, 21.7.

HRMS (ESI, m/z): cacl'd for $C_{14}H_{22}O_4Na$ $[M+Na]^+$: 277.1410, found: 277.1406.

$[\alpha]_D^{22.8}$ = –49.1° (c = 0.32, MeOH).

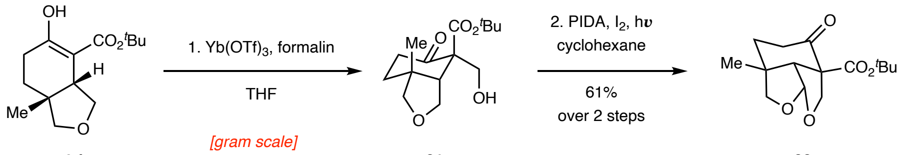

9d **21** **22**

Formalin (35%, 8 mL, 78.6 mmol, 10 equiv) was added to the mixture of **9d** (2.0 g, 7.86 mmol, 1.0 equiv) and Yb(OTf)$_3$ (490 mg, 0.79 mmol, 0.1 equiv) in THF (32 mL) at room temperature. After overnight stirring, the reaction mixture was diluted with H_2O (40 mL), extracted with EtOAc (40 mL×3). The combined organic layers were washed with brine, dried over Na_2SO_4, filtrated, and concentrated under reduced pressure. The crude product of **21** was provided as yellow oil which was used directly for the next step without further purification.

The two-neck round-bottom flask equipped with a reflux condenser was charged crude **21** (assumed 7.86 mmol, 1.0 equiv), (diacetoxyiodo)benzene (PIDA, 10.6 g, 33.0 mmol, 4.2 equiv), and I_2 (6.0 g, 23.6 mmol, 3.0 equiv). After evacuating and backfilling with argon for 3 times, anhydrous cyclohexane (250 mL) was added to the flask. The reaction mixture was irradiated by a halogen lamp and a 250 W incandescent lamp simultaneously until refluxing violently. After 3 h stirring, the light was removed, and the reaction mixture was cooled to room temperature before being quenched with sat. aq. $Na_2S_2O_3$ (100 mL). The organic phase was separated, and aqueous phase was extracted with EtOAc (100 mL×3). The combined organic layers were washed with brine, dried over Na_2SO_4, filtrated, and concentrated under reduced pressure. Purification by column chromatography (silica gel, Petroleum ether:EtOAc, 10:1) gave **22** (1.36 g, 61% over 2 steps) as light yellow oil.

TLC (silica gel, 1:4, EtOAc:Petroleum ether): R_f = 0.3 (phosphomolybdic acid).

^{1}H NMR (400 MHz, CDCl$_3$): δ 5.78 (d, J = 5.2 Hz, 1H), 4.28 – 4.17 (m, 2H), 3.85 (d, J = 9.1 Hz, 1H), 3.56 (d, J = 9.1 Hz, 1H), 3.05 (d, J = 5.3 Hz, 1H), 2.49 – 2.46 (m, 2H), 2.14 – 2.06 (m, 1H), 1.96 – 1.90 (m, 1H), 1.43 (s, 9H), 1.23 (s, 3H).

^{13}C NMR (101 MHz, CDCl$_3$): δ 205.6, 168.7, 110.1, 82.9, 76.0, 72.1, 64.8, 59.7, 40.4, 34.2, 29.8, 28.2, 27.8.

HRMS (ESI, m/z): cacl'd for $C_{13}H_{22}O_5Na$ $[M+Na]^+$: 305.1359, found: 305.1368.

$[\alpha]_D^{22.7}$ = –18.2° (c = 0.72, MeOH).

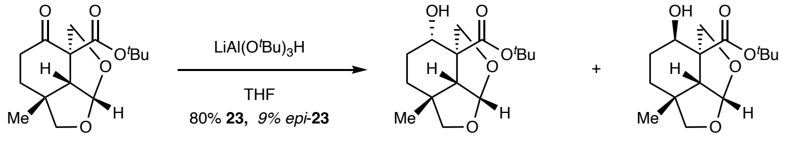

22 **23** *epi*-**23**

LiAl(O^{t}Bu)$_3$H (36.1 mL, 1.0 M in THF, 36.1 mmol, 6.0 equiv) was added dropwise to a cooled (0 °C) solution of **22** (1.7 g, 6.02 mmol, 1.0 equiv) in anhydrous THF (20 mL). After stirring at 0 °C for 2 h, the reaction mixture was quenched with 1 N HCl (10 mL), diluted with H_2O (50 mL), and extracted with EtOAc (50 mL×3). The combined organic phases were washed with brine, dried over Na_2SO_4, filtrated, and concentrated under reduced pressure. Purification by column chromatography (silica gel, Petroleum ether:EtOAc, 4:1) gave **23** (1.37 g, 80%) as light yellow oil and *epi*-**23** (150 mg, 9%) as white amorphous powder.

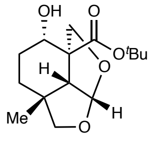

23

TLC (silica gel, 1:2, EtOAc:Petroleum ether, twice): R_f = 0.3 (phosphomolybdic acid)

^{1}H NMR (400 MHz, $CDCl_3$): δ 5.85 (d, *J* = 5.2 Hz, 1H), 4.12 (m, 2H), 4.10 – 4.04 (m, 1H), 3.85 (d, *J* = 8.7 Hz, 1H), 3.46 – 3.36 (m, 2H), 2.69 (d, *J* = 5.2 Hz, 1H), 1.80 – 1.60 (m, 4H), 1.48 (s, 9H), 1.10 (s, 3H).

^{13}C NMR (101 MHz, $CDCl_3$): δ 175.8, 111.9, 82.9, 76.9, 72.4, 71.3, 58.4, 55.2, 40.7, 30.6, 30.0, 28.0, 24.8.

HRMS (ESI, m/z): cacl'd for $C_{15}H_{24}O_5Na$ $[M+Na]^+$: 307.1516, found: 307.1513.

$[\alpha]_D^{24.0}$ = +19.7° (c = 1.52, $CHCl_3$).

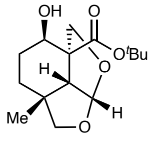

epi-**23**

TLC (silica gel, 1:2, EtOAc:Petroleum ether, twice): R_f = 0.35 (phosphomolybdic acid).

^{1}H NMR (400 MHz, $CDCl_3$): δ 5.88 (d, *J* = 5.1 Hz, 1H), 4.17 (t, *J* = 3.0 Hz, 1H), 4.11 (d, *J* = 9.3 Hz, 1H), 3.76 (d, *J* = 8.7 Hz, 1H), 3.72 (d, *J* = 9.3 Hz, 1H), 3.46 (d, *J* = 8.7 Hz, 1H), 2.94 (d, *J* = 5.2 Hz, 1H), 1.84 (m, 1H), 1.74 (dt, *J* = 10.2, 2.8 Hz, 2H), 1.48 (s, 9H), 1.44 – 1.40 (m, 1H), 1.16 (s, 3H).

^{13}C NMR (101 MHz, CDCl$_3$): δ 173.9, 111.8, 82.0, 77.4, 74.5, 65.1, 59.3, 50.4, 39.4, 30.8, 27.9, 24.2, 23.8.

HRMS (ESI, m/z): cacl'd for $C_{15}H_{24}O_5Na$ $[M+Na]^+$: 307.1516, found: 307.1512.

$[\alpha]_D^{21.9}$ = –31.4° (c = 0.11, MeOH).

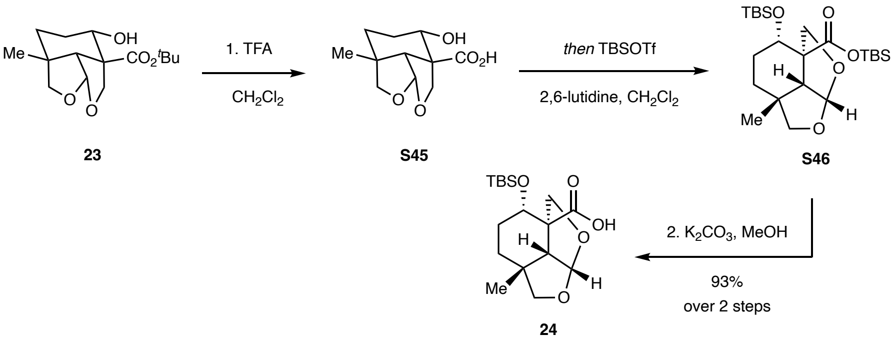

TFA (1 mL) was added dropwise to a cooled (0 °C) solution of **23** (230 mg, 0.81 mmol, 1.0 equiv) in CH_2Cl_2 (4 mL). The reaction mixture was warmed to room temperature and stirred for 2 h before being concentrated under reduced pressure. The crude product **S45** sd as brown oil which was used directly for the next step without further purification.

Note: To rapidly remove TFA completely, the above crude product can be dissolved in toluene and concentrated under vaccum, this process can be repeated until no TFA could be detected by ^{19}F NMR.

To a cooled (0 °C) solution of crude **S45** (assumed 0.81 mmol, 1.0 equiv) and 2,6-lutidine (1.74 g/1.9 mL, 16.3 mmol, 12.0 equiv) in anhydrous CH_2Cl_2 (20 mL) was added *tert*-butyldimethylsilyl trifluoromethanesulfonate (TBSOTf, 2.14 g/1.8 mL, 8.09 mmol, 10.0 equiv). After 15 min stirring, the reaction mixture was warmed to room temperature and stirred for another 3.5 h. The reaction mixture was diluted with CH_2Cl_2 (20 mL) and washed with 20% aqueous solution of citric acid (30 mL). The aqueous phase was separated and extracted with EtOAc (20 mL×3). The combined organic phases were dried over Na_2SO_4, filtrated, and concentrated under reduced pressure. The crude product of **S46** was provided as brown oil which was used directly for the next step without further purification.

A mixture of MeOH/H_2O (12 mL/4 mL) was added to the mixture of the crude product **S46** (assumed 0.81 mmol, 1.0 equiv) and K_2CO_3 (1.12 g, 8.09 mmol, 10.0 equiv) at room temperature. After 30 min stirring, the reaction mixture was diluted with H_2O (20 mL), acidified with 1 N HCl (20 mL), and extracted with EtOAc (40 mL×3). The combined organic phases were washed with brine, dried over Na_2SO_4, filtrated, and concentrated under reduced pressure. Purification by column chromatography (silica gel, CH_2Cl_2:MeOH, 20:1) gave product **24** (259 mg, 93% over 2 steps) as light-red amorphous powder.

TLC (silica gel, 10:1, CH_2Cl_2:MeOH): R_f = 0.4 (UV, $KMnO_4$).

^{1}H NMR (400 MHz, CDCl$_3$): δ 5.85 (d, J = 5.2 Hz, 1H), 4.45 (d, J = 10.0 Hz, 1H), 4.36 – 4.28 (m, 1H), 3.98 (d, J = 10.0 Hz, 1H), 3.89 (d, J = 8.7 Hz, 1H), 3.45 (d, J = 8.7 Hz, 1H), 2.68 (d, J = 5.2 Hz, 1H), 1.78 – 1.74 (m, 1H), 1.71 – 1.62 (m, 3H), 1.12 (s, 3H), 0.82 (s, 9H), 0.03 (s, 3H), –0.01 (s, 3H).

^{13}C NMR (101 MHz, CDCl$_3$): δ 181.5, 110.5, 76.9, 73.0, 70.3, 59.5, 57.0, 40.1, 30.2, 26.5, 25.6, 17.8, –4.2, –5.3.

HRMS (ESI, m/z): cacl'd for $C_{17}H_{31}O_5Si$ $[M+H]^+$: 343.1936, found: 343.1930.

$[\alpha]_D^{21.4}$ = –33.3° (c = 0.10, MeOH).

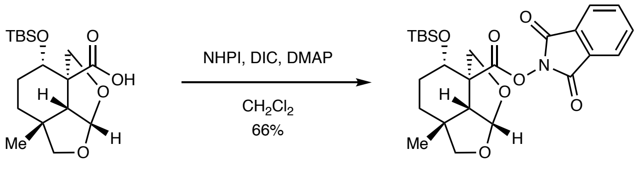

To the mixure of **24** (220 mg, 0.64 mmol, 1.0 equiv), NHPI (116 mg, 0.71 mmol, 1.1 equiv), and DMAP (39 mg, 0.32 mmol, 0.5 equiv) in anhydrous CH_2Cl_2 (2 mL) was added DIC (90 mg/110 μL) at room temperature. After 2 h stirring, the reaction mixture was concentrated under reduced pressure. Purification by column chromatography (silica gel, CH_2Cl_2) gave redox-active ester **25** (205 mg, 66%) as white amorphous powder.

TLC (silica gel, 1:1, EtOAc:Petroleum ether): R_f = 0.3 (phosphomolybdic acid)

^{1}H NMR (400 MHz, CDCl$_3$): δ 7.88 (m, 2H), 7.80 (m, 2H), 6.02 (d, J = 5.2 Hz, 1H), 4.57 (m, 1H), 4.53 (d, J = 10.6 Hz, 1H), 4.16 (d, J = 10.4 Hz, 1H), 3.93 (d, J = 8.8 Hz, 1H), 3.48 (d, J = 8.8 Hz, 1H), 2.93 (d, J = 5.2 Hz, 1H), 1.82 – 1.67 (m, 4H), 1.17 (s, 3H), 0.84 (s, 9H), 0.10 (s, 3H), 0.09 (s, 3H).

^{13}C NMR (101 MHz, CDCl$_3$): δ 171.9, 161.7, 134.8, 129.0, 123.9, 111.2, 76.7, 72.0, 70.8, 58.8, 57.4, 40.3, 30.2, 30.0, 26.6, 25.6, 17.8, –4.1, –5.4.

HRMS (ESI, m/z): cacl'd for $C_{25}H_{34}NO_7Si$ $[M+H]^+$: 488.2099, found: 488.2096.

$[\alpha]_D^{21.7}$ = –17.2° (c = 0.16, MeOH).

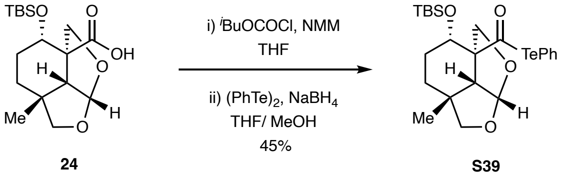

NMM (*N*-methylmorpholine, 8.3 mg, 0.082 mmol, 1.4 equiv) and ^{i}BuOCOCl (11.2 mg, 0.82 mmol, 1.4 equiv) were added to a solution of **24** (20 mg, 0.058 mmol, 1.0 equiv) in THF (0.4 mL) at 0 °C under Ar. The reaction mixture was stirred for another 30 min. MeOH (40 μL) was added dropwise to a suspension of $(PhTe)_2$ (33.5 mg, 0.082 mmol, 1.4 equiv) and $NaBH_4$ (7.5 mg, 0.2 mmol, 3.4 equiv) in THF (0.4 mL) at 0 °C. After both reaction mixture was stirred at 0 °C for 30 min, the latter reaction mixture was added dropwise to the former one. The resultant mixture was stirred at room temperature for 40 min and was then filtered through a pad of silica gel and concentrated. The residue was purified by flash column chromatography (silica gel, Petroleum ether:EtOAc, 10:1) gave product **S39** (21 mg, 69%) as yellow solid.

TLC (silica gel, 5:1, EtOAc:Petroleum ether): R_f = 0.7 (UV, $KMnO_4$)

^{1}H NMR (500 MHz, $CDCl_3$): δ 7.72 – 7.66 (m, 2H), 7.41 – 7.37 (m, 1H), 7.31 (t, *J* = 7.5 Hz, 2H), 5.97 (d, *J* = 5.2 Hz, 1H), 4.46 – 4.41 (m, 2H), 4.18 (d, *J* = 11.0 Hz, 1H), 3.95 (d, *J* = 8.8 Hz, 1H), 3.47 (d, *J* = 8.8 Hz, 1H), 2.43 (d, *J* = 5.2 Hz, 1H), 1.74 – 1.64 (m, 3H), 1.58 – 1.48 (m, 1H), 1.10 (s, 3H), 0.82 (s, 9H), 0.02 (s, 3H), -0.01 (s, 3H).

^{13}C NMR (126 MHz, $CDCl_3$): δ 209.0, 140.6, 129.3, 128.8, 114.7, 110.9, 77.3, 71.23, 71.21, 70.9, 56.4, 40.7, 30.1, 30.0, 27.0, 25.7, 17.9, –4.1, –5.0.

HRMS (ESI, m/z): cacl'd for $C_{23}H_{34}O_4SiTeNa$ $[M+Na]^+$: 555.1181, found: 555.1167.

$[\alpha]_D^{21.1}$ = +18.5° (c = 0.12, MeOH).

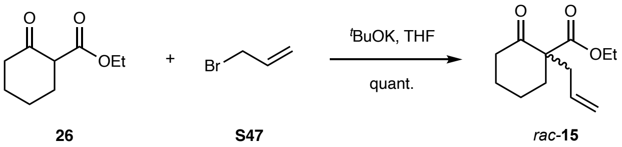

26 (1.12 g/0.8 mL, 6.6 mmol, 1.05 equiv) was added to a solution of **S47** (1.06 g/1 mL, 6.2 mmol, 1.0 equiv) and ^{t}BuOK (740 mg, 6.6 mmol, 1.05 equiv) in anhydrous THF (12 mL) at room temperature. After heating at 70 °C for 18 h, the reaction mixture was cooled to room temperature and acidified with 1 N HCl (20 mL). The aqueous phase was extracted with CH_2Cl_2 (20 mL×3). The combined organic phases were washed with brine, dried over Na_2SO_4, filtrated, and concentrated under reduced pressure. Purification by column chromatography (silica gel, Petroleum ether:EtOAc, 70:1) gave product *rac*-**15** (1.33 g, quant.) as colorless oil.

TLC (silica gel, 10:1, Petroleum ether:EtOAc): R_f = 0.5 (UV).

^{1}H NMR (400 MHz, CDCl$_3$): δ 5.78 – 5.66 (m, 1H), 5.06 – 4.98 (m, 2H), 4.18 (q, *J* = 7.1 Hz, 2H), 2.60 (m, 1H), 2.53 – 2.37 (m, 3H), 2.37 – 2.28 (m, 1H), 2.08 – 1.93 (m, 1H), 1.71 – 1.53 (m, 3H), 1.53 – 1.39 (m, 1H), 1.25 (t, *J* = 7.1 Hz, 3H).

^{13}C NMR (101 MHz, CDCl$_3$): δ 207.6, 171.5, 133.3, 118.3, 61.2, 60.9, 41.1, 39.3, 35.8, 27.5, 22.5, 14.2.

HRMS (ESI, m/z): cacl'd for $C_{12}H_{19}O_3$ $[M+H]^+$: 211.1329, found: 211.1326.

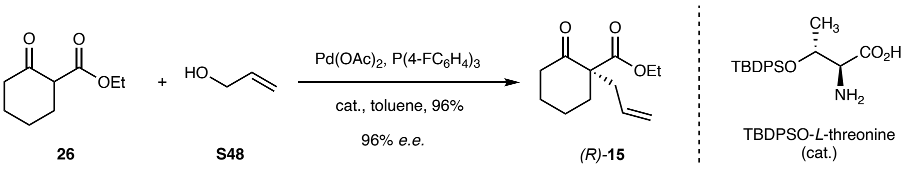

(*R*)**-15** was prepared according to Yoshida's method with slight modification[20].

26 (5.0 g/4.7 mL, 29.4 mmol, 1.0 equiv) and **S48** (1.88 g/2.2 mL, 32.3 mmol, 1.1 equiv) were added successively to a solution of $Pd(OAc)_2$ (330 mg, 1.47 mmol, 5% equiv), $P(4\text{-}F\text{-}C_6H_4)_3$ (tris(4-fluorophenyl)phosphine, 1.39 g, 4.4 mmol, 0.15 equiv), and TBDPSO-*L*-threonine (2.1 g, 5.9 mmol, 0.2 equiv) in anhydrous toluene (35 mL). After heating at 40 °C for 16 h, the reaction mixture was cooled to room temperature and concentrated under reduced pressure. Purification by column chromatography (silica gel, Petroleum ether:EtOAc, 70:1) gave product (*R*)-**15** (5.9 g, 96%, 96% *ee*) as colorless oil.

TLC (silica gel, 10:1, Petroleum ether:EtOAc): R_f = 0.5 (UV).

^{1}H NMR (400 MHz, CDCl$_3$): δ 5.78 – 5.66 (m, 1H), 5.06 – 4.98 (m, 2H), 4.18 (q, *J* = 7.1 Hz, 2H), 2.60 (m, 1H), 2.53 – 2.37 (m, 3H), 2.37 – 2.28 (m, 1H), 2.08 – 1.93 (m, 1H), 1.71 – 1.53 (m, 3H), 1.53 – 1.39 (m, 1H), 1.25 (t, *J* = 7.1 Hz, 3H).

^{13}C NMR (101 MHz, CDCl$_3$): δ 207.6, 171.5, 133.3, 118.3, 61.2, 60.9, 41.1, 39.3, 35.8, 27.5, 22.5, 14.2.

HRMS (ESI, m/z): cacl'd for $C_{12}H_{19}O_3$ $[M+H]^+$: 211.1329, found: 211.1323.

$[\alpha]_D^{22.7}$ = +100.2° (c =0.71, $CHCl_3$).

*Method for determining the enantioselectivity of the asymmetric product, using rac-**15** and (R)-**15**:*

Column: Chiralpak® AD-H

Dimensions: 4.6 × 250 mm

Eluent: hexanes: IPA = 99.81: 0.19

Flow rate: 1.0 mL/min

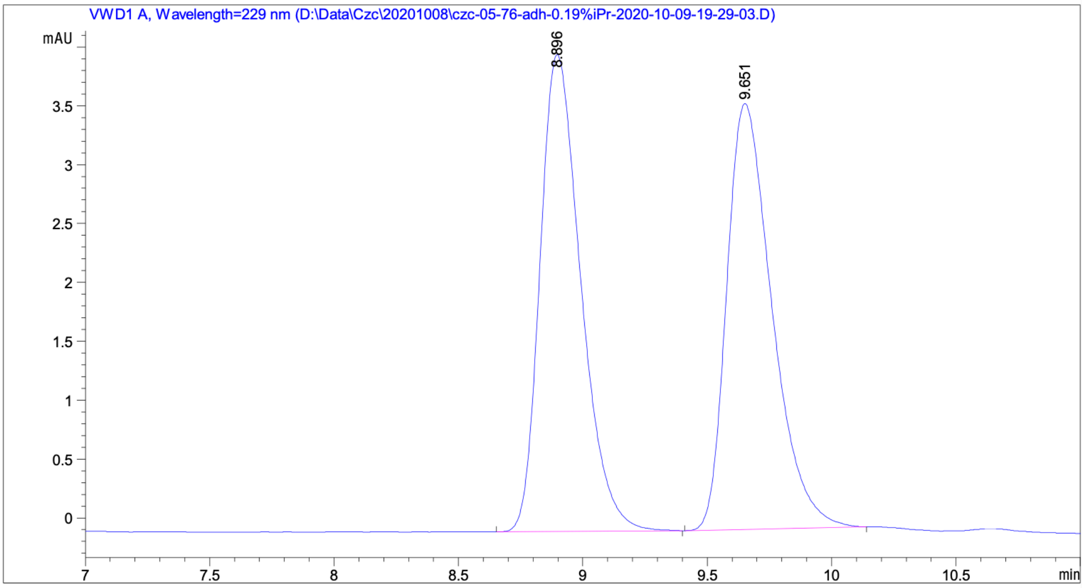

===
Area Percent Report
===

Sorted By : Signal
Multiplier : 2.0000
Dilution : 1.0000
Do not use Multiplier & Dilution Factor with ISTDs

Signal 1: VWD1 A, Wavelength=229 nm

Peak #	RetTime [min]	Type	Width [min]	Area [mAU*s]	Height [mAU]	Area %
1	8.896	BB	0.1746	46.03860	4.05174	50.1733
2	9.651	BB	0.1935	45.72064	3.61600	49.8267

Totals : 91.75924 7.66774

Supplementary Figure 43. The HPLC spectra of *rac*-15.

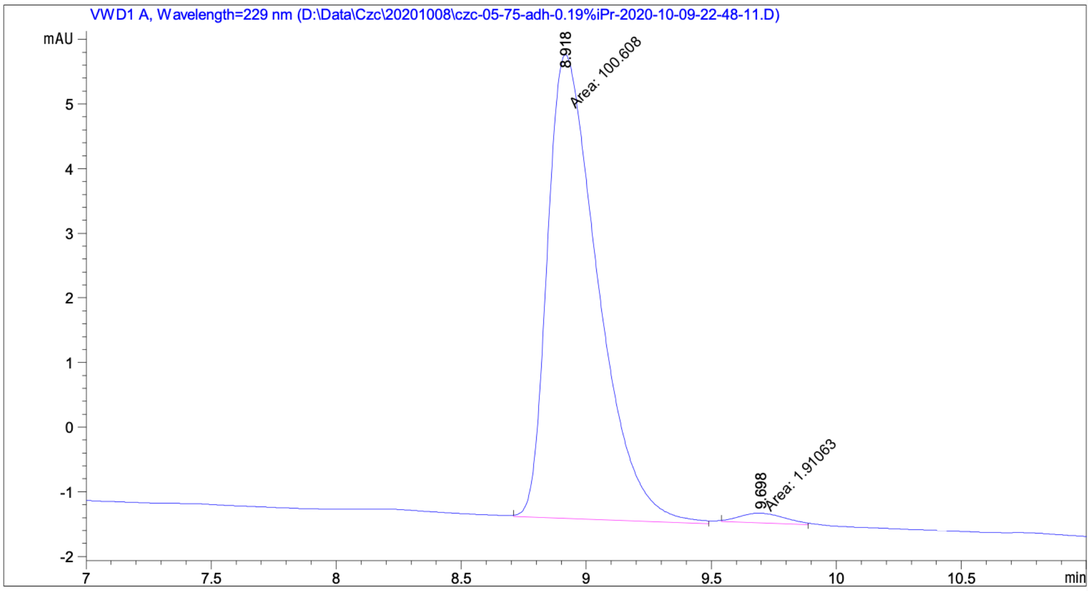


```
=====================================================================
                         Area Percent Report
=====================================================================

Sorted By             :      Signal
Multiplier            :      2.0000
Dilution              :      1.0000
Do not use Multiplier & Dilution Factor with ISTDs


Signal 1: VWD1 A, Wavelength=229 nm

Peak RetTime Type  Width     Area      Height     Area
  #   [min]        [min]   [mAU*s]     [mAU]        %
----|-------|----|-------|----------|----------|--------|
   1   8.918 MM    0.2337  100.60783    7.17409  98.1363
   2   9.698 MM    0.2103    1.91063 1.51451e-1   1.8637

Totals :                   102.51846    7.32554
```

Supplementary Figure 44. The HPLC spectra of the asymmetric product (*R*)-15.

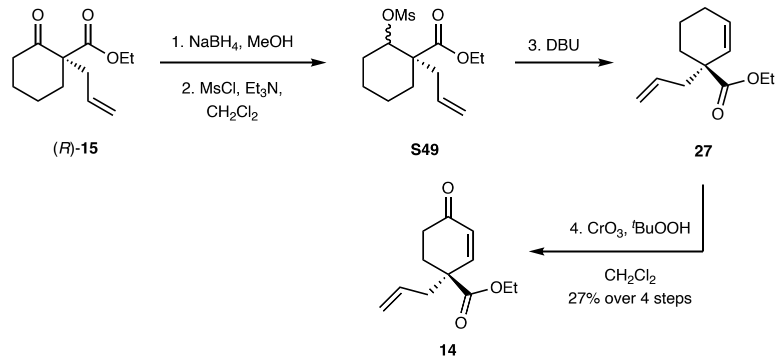

A freshly prepared solution of $NaBH_4$ (1.67 g, 44.15 mmol, 3.0 equiv) in MeOH (25 mL) was added dropwise to a cooled (0 °C) solution of (*R*)-**15** (3.09 g, 14.72 mmol, 1.0 equiv) in MeOH (5 mL). After stirring at 0 °C for 30 min, the reaction mixture was quenched by slow addition of acetone (5 mL). The resulting mixture was evaporated under reduced pressure to give the crude product which was used directly for the next step without further purification.

Et_3N (2.98 g/4.09 mL, 29.44 mmol, 2.0 equiv) and methanesulfonyl chloride (MsCl, 3.37 g/2.28 mL, 29.44 mmol, 2.0 equiv) were added dropwise successively to a cooled (0 °C) solution of above crude product (assumed 14.72 mmol, 1.0 equiv) in anhydrous CH_2Cl_2 (50 mL). After slowly warming to room temperature over 1 h, the reaction mixture was poured into H_2O (50 mL), and the organic phase was separated. The aqueous phase was extracted with EtOAc (50 mL×3). The combined organic phases were washed with sat. aq. $NaHCO_3$ (50 mL) and brine successively, dried over Na_2SO_4, filtrated, and concentrated under reduced pressure. The crude product **S49** was provided as yellow oil which was used directly for the next step without further purification.

The Schlenk tube charged with above crude product **S49** (assumed 14.72 mmol, 1.0 equiv) and 1, 8-diazabicyclo[5,4,0]undec-7-ene (DBU, 8.96 g/8.8 mL, 58.9 mmol, 4.0 equiv) was heating at 110 °C for 7 h. The reaction mixture was cooled to room temperature, diluted with H_2O (40 mL), and extracted with EtOAc (30 mL×3). The combined organic phases were washed with 1 N HCl (60 mL), sat. aq. $NaHCO_3$ (60 mL), and brine successively, dried over Na_2SO_4, filtrated, and concentrated under reduced pressure. The crude product of **27** was used directly for the next step without further purification.

70% aqueous of ^{t}BuOOH (3.9 mL, 28.16 mmol, 4.0 equiv) was added dropwise to a cooled (0 °C) suspension of CrO_3 (141 mg, 1.41 mmol, .0.2 equiv) in CH_2Cl_2 (3 mL). After 10 min stirring, the solution of crude product of **27** (assumed 14.72 mmol, 1.0 equiv) in CH_2Cl_2 (10 mL) was added dropwise at a rate keeping the internal temperature below 6 °C. Then the reaction mixture was stirred at 0 °C for 2 h before being warmed to 10 °C. After 18 h stirring at 10 °C, the reaction mixture was cooled to 0 °C, quenched with sat. aq. $NaHSO_3$ (50 mL) and extracted with EtOAc (50 mL×3). The combined organic phases were washed with sat. aq. $NaHCO_3$ and brine successively, dried over Na_2SO_4, filtrated, and concentrated under reduced pressure. Purification by column chromatography (silica gel, Petroleum ether:EtOAc, 20:1) gave product **14** (817 mg, 27% over 4 steps) as colorless oil.

TLC (silica gel, 5:1, Petroleum ether:EtOAc): $R_f = 0.5$ (UV).

^{1}H NMR (400 MHz, $CDCl_3$): δ 6.91 (dd, J = 10.2, 1.2 Hz, 1H), 6.01 (d, J = 10.2 Hz, 1H), 5.70 (m, 1H), 5.15 (m, 2H), 4.19 (m, 2H), 2.65 – 2.30 (m, 5H), 2.01 (m, 1H), 1.27 (t, J = 7.1 Hz, 3H).

^{13}C NMR (101 MHz, $CDCl_3$): δ 198.4, 172.7, 150.5, 131.9, 129.18, 119.6, 61.4, 47.5, 42.8, 34.5, 30.2, 14.2.

HRMS (ESI, m/z): cacl'd for $C_{12}H_{17}O_3$ $[M+H]^+$: 209.1172, found: 209.1171.

$[\alpha]_D^{21.6}$ = –62.2° (c = 1.13, MeOH).

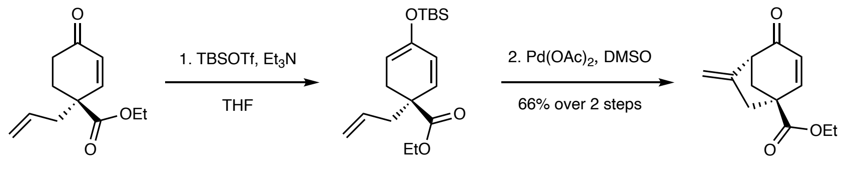

13 was prepared from **14** according to Liu's method with slight modification[21].

To a cooled (–60 °C) solution of **14** (817 mg, 3.92 mmol, 1.0 equiv) in anhydrous THF (5 mL) was added Et_3N (1.59 g/2.19 mL, 15.69 mmol. 4.0 equiv). After 20 min stirring, TBSOTf (2.59 g/2.25 mL, 9.81 mmol, 2.5 equiv) was added dropwise to the above mixture. After 2 h stirring at –60 °C, the reaction mixture was quenched with sat. aq. $NaHCO_3$ (20 mL), extracted with EtOAc (20 mL×3). The combined organic phases were washed with brine, dried over Na_2SO_4, filtrated, and concentrated under reduced pressure. The crude product **S50** was used directly for the next step without further purification.

Under an atmosphere of O_2, to a solution of the above crude product **S50** (assumed 3.92 mmol, 1.0 equiv) in DMSO (35 mL) was added $Pd(OAc)_2$ (150 mg, 0.67 mmol, 0.17 equiv). The reaction mixture was warmed to 45 °C. After 17.5 h stirring, the reaction mixture was diluted with EtOAc (50 mL), filtrated through celite, and washed with EtOAc (20 mL). The filtrate was washed with H_2O (70 mL×3) and brine (70 mL×1) whereby the aqueous layers were back-extracted with EtOAc (50 mL×3). The organic phases were combined, dried over Na_2SO_4, filtrated, and concentrated under reduced pressure. Purification by column chromatography (silica gel, Petroleum ether:EtOAc, 20:1) gave product **13** (531 mg, 66% over 2 steps) as colorless oil.

TLC (silica gel, 5:1, Petroleum ether:EtOAc): $R_f = 0.4$ (UV).

^{1}H NMR (400 MHz, $CDCl_3$): δ 7.47 (dd, J = 9.9, 1.8 Hz, 1H), 5.87 (dd, J = 9.9, 1.6 Hz, 1H), 5.32 (m, 1H), 5.10 (m, 1H), 4.25 (q, J = 7.1 Hz, 2H), 3.53 (m, 1H), 2.94 (dt, J = 15.8, 2.6 Hz, 1H), 2.58 (m, 1H), 2.39 – 2.25 (m, 2H), 1.31 (t, J = 7.1 Hz, 3H).

^{13}C NMR (101 MHz, CDCl$_3$): δ 197.4, 172.8, 152.2, 143.2, 126.7u, 113.1, 61.6, 58.0, 51.6, 43.2, 41.1, 14.1.

HRMS (ESI, m/z): cacl'd for $C_{12}H_{15}O_3$ $[M+H]^+$: 207.1016, found: 207.1014.

$[\alpha]_D^{22.3}$ = +95.0° (c = 1.82, MeOH).

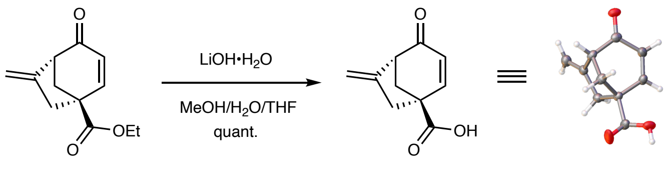

13 28 X-ray of **28**

To a solution of **13** (370 mg, 1.8 mmol, 1.0 equiv) in MeOH (10 mL) was added a solution of LiOH·H_2O (188 mg, 4.5 mmol, 2.5 equiv) in H_2O (2 mL). THF (400 μL) was added to the reaction mixture. After stirring at room temperature for 30 min, the reaction mixture was concentrated, acidified with 0.5 N HCl (50 mL), and extracted with EtOAc (30 mL×3). The combined organic phases were dried over Na_2SO_4, filtrated, and concentrated under reduced pressure to give carboxylic acid **28** (330 mg, quant.) which was used directly for the next step.

TLC (silica gel, 10:1, CH_2Cl_2:MeOH): R_f = 0.7 (UV).

^{1}H NMR (400 MHz, CDCl$_3$) δ 7.48 (dd, J = 9.9, 1.7 Hz, 1H), 5.92 (dd, J = 9.9, 1.5 Hz, 1H), 5.36 (s, 1H), 5.15 (s, 1H), 3.58 (m, 1H), 3.02 (dt, J = 15.7, 2.6 Hz, 1H), 2.69 – 2.62 (m, 1H), 2.44 – 2.33 (m, 2H).

^{13}C NMR (101 MHz, CDCl$_3$): δ 197.1, 177.9, 151.1, 142.7, 127.1, 113.5, 57.9, 51.3, 43.3, 41.4.

HRMS (ESI, m/z): cacl'd for $C_{10}H_9O_3$ $[M-H]^-$: 177.0557, found: 177.0553.

$[\alpha]_D^{20.6}$ = +136.7° (c = 0.22, MeOH).

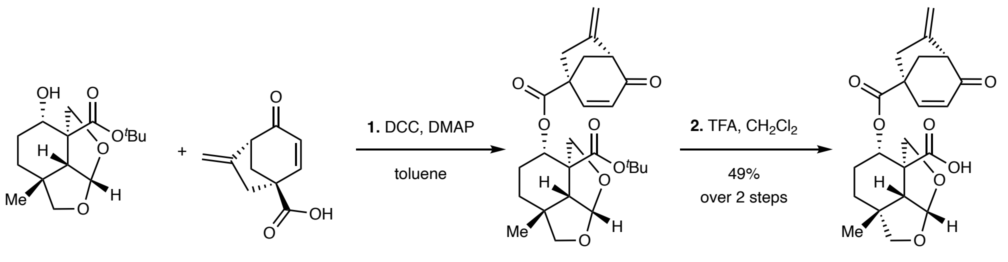

23 28 S40 34

A solution of *N,N'*-dicyclohexylacarbodiimide (DCC, 588 mg, 2.85 mmol, 3.0 equiv) in anhydrous toluene (5 mL) was added dropwise to the mixture of **23** (270 mg, 0.95 mmol, 1.0 equiv), **28** (334 mg, 1.88 mmol, 2.0 equiv), and DMAP (1.39 g, 11.4 mmol, 12.0 equiv) in anhydrous toluene (5 mL). After stirring at room temperature overnight, the reaction mixture was diluted with H_2O (10 mL) and extracted with Et_2O (10 mL×3). The combined organic phases were washed with brine, dried over Na_2SO_4, filtrated, and concentrated under reduced

pressure. The residue was redissolved with Et_2O (5 mL) and filtrated to remove the insoluble solid to give crude product of **S40** as colorless oil which was used directly for the next step without further purification.

TFA (0.4 mL) was added dropwise to a cooled (0 °C) solution of crude **S40** (assumed 0.95 mmol, 1.0 equiv) in CH_2Cl_2 (2 mL). After warming to room temperature and stirring for 4 h, the reaction mixture was concentrated under reduced pressure. Purification by column chromatography (silica gel, CH_2Cl_2:MeOH, 20:1) gave product **34** (180 mg, 49% over 2 steps) as colorless oil.

TLC (silica gel, 10:1, CH_2Cl_2:MeOH): R_f = 0.3 (UV).

^{1}H NMR (400 MHz, $CDCl_3$): δ 7.35 (d, J = 9.9 Hz, 1H), 5.88 (m, 2H), 5.51 (m, 1H), 5.34 (s, 1H), 5.13 (s, 1H), 4.42 (d, J = 9.7 Hz, 1H), 4.08 (d, J = 9.8 Hz, 1H), 3.91 (d, J = 8.8 Hz, 1H), 3.53 (m, 2H), 2,90 (m, 2H), 2.55 (d, J = 15.9 Hz, 1H), 2.25 (m, 2H), 1.96 – 1.82 (m, 2H), 1.75 (m, 2H), 1.18 (s, 3H).

^{13}C NMR (101 MHz, $CDCl_3$): δ 197.2, 178.2, 171.8, 151.5, 142.7, 127.0, 113.6, 110.6, 77.2, 74.5, 70.3, 57.8, 56.7, 56.4, 51.8, 43.1, 41.0, 40.2, 30.1, 29.8, 22.9.

HRMS (ESI, m/z): cacl'd for $C_{21}H_{23}O_7$ $[M–H]^-$: 387.1449, found: 387.1455.

$[\alpha]_D^{21.6}$ = +5.3° (c = 0.25, MeOH).

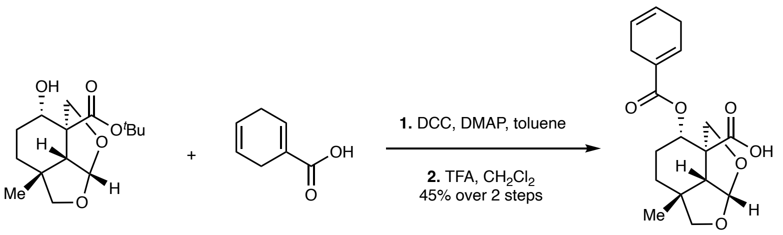

23 S41 35

A solution of DCC (792 mg, 3.84 mmol, 3.0 equiv) in anhydrous toluene (5 mL) was added dropwise to a solution of **23** (364 mg, 1.28 mmol, 1.0 equiv), **S41** (477 mg, 3.84 mmol, 3.0 equiv), and DMAP (1.88 g, 15.36 mmol, 12.0 equiv) in anhydrous toluene (15 mL). After stirring at room temperature for 4 h, the reaction mixture was diluted with H_2O (20 mL), extracted with Et_2O (20 mL×3). The combined organic phases were washed with brine, dried over Na_2SO_4, filtrated, and concentrated under reduced pressure. The residue was redissolved with Et_2O (10 mL) and filtrated to remove the insoluble solid to give a crude product which was used directly for the next step without further purification.

TFA (3 mL) was added dropwise to a solution of above crude product (assumed 1.28 mmol, 1.0 equiv) in CH_2Cl_2 (15 mL). After stirring at room temperature for 2 h, the reaction mixture was concentrated under reduced pressure. Purification by column chromatography (silica gel, CH_2Cl_2:MeOH, 20:1) gave **35** (192 mg, 45% over 2 steps) as white amorphous powder.

TLC (silica gel, 10:1, CH_2Cl_2:MeOH): R_f = 0.4 (UV, $KMnO_4$).

^{1}H NMR (400 MHz, CDCl$_3$): δ 6.89 (d, *J* = 1.8 Hz, 1H), 5.90 (d, *J* = 5.2 Hz, 1H), 5.79 – 5.61 (m, 2H), 5.47 (dd, *J* = 11.3, 4.7 Hz, 1H), 4.48 (d, *J* = 9.8 Hz, 1H), 4.11 (d, *J* = 9.8 Hz, 1H), 3.92 (d, *J* = 8.8 Hz, 1H), 3.50 (d, *J* = 8.8 Hz, 1H), 2.96 – 2.78 (m, 5H), 1.99 – 1.89 (m, 1H), 1.87 – 1.59 (m, 3H), 1.16 (s, 3H).

^{13}C NMR (101 MHz, CDCl$_3$): δ 178.8, 165.8, 137.7, 127.3, 124.2, 122.2, 110.6, 77.2, 73.6, 70.5, 56.9, 56.5, 40.2, 30.1, 29.8, 27.1, 25.0, 22.7.

HRMS (ESI, m/z): cacl'd for $C_{18}H_{21}O_6$ $[M–H]^-$: 333.1343, found: 333.1348.

$[\alpha]_D^{20.8}$ = –12.3° (c = 0.13, MeOH).

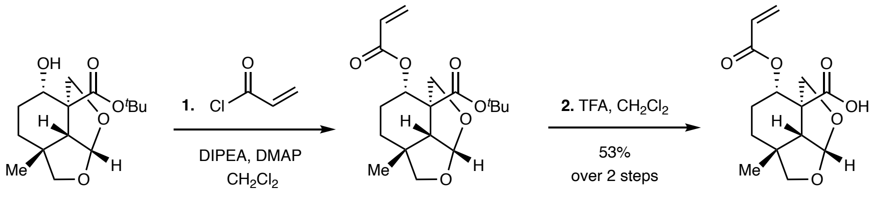

23 **S42** **36**

Acryloyl chloride (96 mg/85 *μ*L, 1.06 mmol, 6.0 equiv) was added dropwise to a cooled (–78 °C) solution of **23** (50 mg, 0.18 mmol, 1.0 equiv), *N, N*-diisopropylethylamine (DIPEA, 227 mg/290 *μ*L, 1.76 mmol, 10.0 equiv), and DMAP (2.2 mg, 0.02 mmol, 0.1 equiv) in anhydrous CH_2Cl_2 (1 mL). After stirring at –78 °C for 4 h, the reaction mixture was quenched with MeOH (0.1 mL), diluted with H_2O (1 mL), extracted with CH_2Cl_2 (2 mL×3). The combined organic phases were washed with brine, dried over Na_2SO_4, filtrated, and concentrated under reduced pressure to give crude product **S42** which was used directly for the next step without further purification.

TFA (0.1 mL) was added dropwise to a solution of the crude product **S42** (assumed 0.18 mmol, 1.0 equiv) in CH_2Cl_2 (0.5 mL). After stirring at room temperature for 2 h, the reaction mixture was concentrated under reduced pressure. Purification by column chromatography (silica gel, CH_2Cl_2:MeOH, 20:1) gave product **36** (27 mg, 53% over 2 steps) as white amorphous powder.

TLC (silica gel, 10:1, CH_2Cl_2:MeOH): R_f = 0.6 (UV).

^{1}H NMR (400 MHz, CDCl$_3$): δ 6.35 (d, *J* = 17.2 Hz, 1H), 6.05 (dd, *J* = 17.3, 10.5 Hz, 1H), 5.89 (d, *J* = 5.2 Hz, 1H), 5.84 (d, *J* = 10.4 Hz, 1H), 5.49 (m, 1H), 4.47 (d, *J* = 10.1 Hz, 1H), 4.08 (d, *J* = 9.2 Hz, 1H), 3.91 (d, *J* = 8.6 Hz, 1H), 3.49 (d, *J* = 8.8 Hz, 1H), 2.87 (d, *J* = 5.1 Hz, 1H), 1.97 – 1.67 (m, 4H), 1.16 (s, 3H).

^{13}C NMR (101 MHz, CDCl$_3$): δ 178.8, 165.2, 131.7, 127.9, 110.5, 77.2, 74.0, 70.5, 57.0, 56.4, 40.3, 30.1, 29.8, 22.7.

HRMS (ESI, m/z): cacl'd for $C_{14}H_{17}O_6$ $[M–H]^-$: 281.1030, found: 281.1032.

$[\alpha]_D^{22.3}$ = –2.1° (c = 0.48, MeOH).

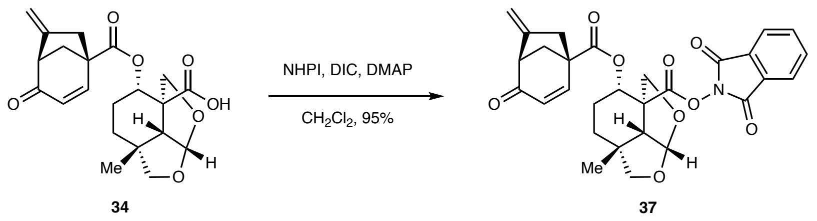

A solution of DIC (11.5 mg/14 μL, 0.04 mmol, 0.5 equiv) in CH_2Cl_2 (1 mL) was added to a solution of **34** (32 mg, 0.08 mmol, 1.0 equiv), NHPI (14.8 mg, 0.09 mmol, 1.1 equiv), and DMAP (5.0 mg, 0.04 mmol, 0.5 equiv) in anhydrous CH_2Cl_2 (1 mL). After stirring at room temperature for 2 h, the reaction mixture was concentrated under reduced pressure. Purification by column chromatography (silica gel, Petroleum ether:EtOAc, 2:1) gave **37** (40 mg, 95%) as white amorphous powder.

TLC (silica gel, 1:1, Petroleum ether:EtOAc): R_f = 0.3 (UV).

^{1}H NMR (400 MHz, $CDCl_3$): δ 7.88 (m, 2H), 7.81 (m, 2H), 7.41 (dd, J = 9.9, 1.8 Hz, 1H), 6.03 (d, J = 5.1 Hz, 1H), 5.88 (dd, J = 9.9, 1.6 Hz, 1H), 5.71 (m, 1H), 5.33 (m, 1H), 5.11 (m, 1H), 4.60 (d, J = 10.1 Hz, 1H), 4.23 (d, J = 10.1 Hz, 1H), 3.94 (d, J = 8.9 Hz, 1H), 3.55 (m, 2H), 3.03 (d, J = 5.1 Hz, 1H), 2.90 (dt, J = 16.0, 2.6 Hz, 1H), 2.60 (d, J = 15.9 Hz, 1H), 2.38 – 2.23 (m, 2H), 2.12 – 1.98 (m, 1H), 1.97 – 1.66 (m, 3H), 1.23 (s, 3H).

^{13}C NMR (101 MHz, $CDCl_3$): δ 197.2, 171.6, 170.6, 161.6, 151.7, 142.9, 135.0, 128.8, 126.9, 124.2, 113.3, 110.7, 77.2, 74.5, 70.3, 57.9, 57.1, 56.1, 51.9, 43.0, 40.9, 40.4, 30.1, 29.8, 22.4.

HRMS (ESI, m/z): cacl'd for $C_{29}H_{28}NO_9$ $[M+H]^+$: 534.1764, found: 534.1753.

$[\alpha]_D^{19.2}$ = +80.2° (c = 0.44, MeOH).

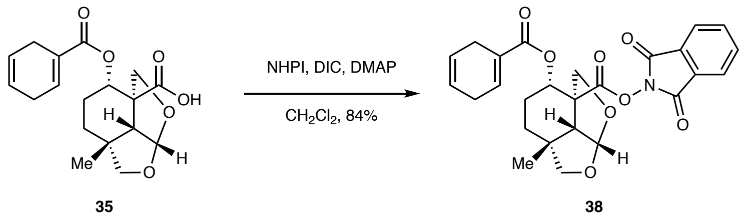

DIC (42 mg/51 μL, 0.33 mmol, 1.1 equiv) was added to a solution of **35** (100 mg, 0.30 mmol, 1.0 equiv), NHPI (53.8 mg, 0.33 mmol, 1.1 equiv), and DMAP (18.3 mg, 0.15 mmol, 0.5 equiv) in anhydrous CH_2Cl_2 (5 mL). After stirring at room temperature for 2.5 h, the reaction mixture was concentrated under reduced pressure. Purification by column chromatography (silica gel, Petroleum ether:EtOAc, 5:1) gave product **38** (121 mg, 84%) as white amorphous powder.

TLC (silica gel, 1:1, Petroleum ether:EtOAc): R_f = 0.5 (UV).

^{1}H NMR (400 MHz, $CDCl_3$): δ 7.89 (m, 2H), 7.80 (m, 2H), 6.94 (m, 1H), 6.07 (d, J = 5.1 Hz, 1H), 5.79 (d, J = 10.2 Hz, 1H), 5.67 (m, 2H), 4.66 (d, J = 10.2 Hz, 1H), 4.31 (d, J = 10.2 Hz, 1H), 3.98 (d, J = 8.9 Hz, 1H), 3.56 (d, J = 8.9 Hz, 1H), 3.04 (d, J = 5.2 Hz, 1H), 2.91 (m, 4H), 2.18 – 2.01 (m, 1H), 1.87 – 1.64 (m, 3H), 1.24 (s, 3H).

^{13}C NMR (101 MHz, CDCl$_3$): δ 170.8, 165.6, 161.6, 137.5, 134.8, 128.9, 127.3, 124.3, 124.1, 122.2, 110.9, 77.23, 73.3, 70.7, 57.1, 56.1, 40.4, 30.2, 29.8, 27.1, 25.0, 22.4.

HRMS (ESI, m/z): cacl'd for $C_{26}H_{25}NO_8Na$ $[M+Na]^+$: 502.1472, found: 502.1468.

$[\alpha]_D^{21.2}$ = +1.6° (c = 0.26, CDCl$_3$).

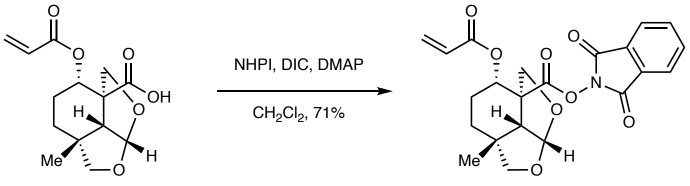

36 39

DIC (18 mg/22 μL, 0.14 mmol, 1.5 equiv) was added to a solution of **36** (26 mg, 0.09 mmol, 1.0 equiv), NHPI (23 mg, 0.14 mmol, 1.5 equiv), and DMAP (5.7 mg, 0.05 mmol, 0.5 equiv) in anhydrous CH_2Cl_2 (1 mL). After stirring at room temperature for 2.5 h, the reaction mixture was concentrated under reduced pressure. Purification by column chromatography (silica gel, CH_2Cl_2:EtOAc, 10:1) gave product **39** (27 mg, 71 %) as white amorphous powder.

TLC (silica gel, 1:1, Petroleum ether:EtOAc): R_f = 0.5 (UV).

^{1}H NMR (400 MHz, CDCl$_3$): δ 7.88 – 7.85 (m, 2H), 7.80 – 7.76 (m, 2H), 6.40 (dd, J = 17.3, 1.4 Hz, 1H), 6.11 (dd, J = 17.3, 10.5 Hz, 1H), 6.04 (d, J = 5.1 Hz, 1H), 5.87 (dd, J = 10.5, 1.4 Hz, 1H), 5.68 (m, 1H), 4.62 (d, J = 10.2 Hz, 1H), 4.27 (d, J = 10.2 Hz, 1H), 3.95 (d, J = 8.9 Hz, 1H), 3.54 (d, J = 8.8 Hz, 1H), 3.02 (d, J = 5.1 Hz, 1H), 2.17 – 2.03 (m, 1H), 1.93 – 1.65 (m, 3H), 1.22 (s, 3H).

^{13}C NMR (101 MHz, CDCl$_3$): δ 170.7, 164.9, 161.6, 134.9, 131.6, 128.9, 127.8, 124.1, 110.8, 77.2, 73.5, 70.6, 57.2, 56.0, 40.4, 30.2, 29.8, 22.4.

HRMS (ESI, m/z): cacl'd for $C_{22}H_{21}NO_8Na$ $[M+Na]^+$: 450.1159, found: 450.1153.

$[\alpha]_D^{24.2}$ = +22.7° (c = 0.33, MeOH).

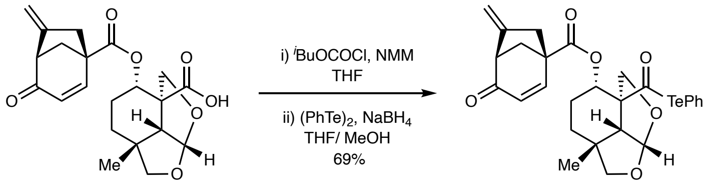

34 S43

NMM (N-methylmorpholine, 32 mg, 0.32 mmol, 1.4 equiv) and ^{i}BuOCOCl (43 mg, 0.32 mmol, 1.4 equiv) were added to a solution of **34** (87 mg, 0.23 mmol, 1.0 equiv) in THF (2 mL) at 0 °C under Ar. The reaction mixture was stirred for another 30 min. MeOH (200 μL) was added dropwise to a suspension of $(PhTe)_2$ (129 mg, 0.32mmol, 1.4 equiv) and $NaBH_4$ (29 mg, 0.77 mmol, 3.4 equiv) in THF (2 mL) at 0 °C. After both reaction mixture was stirred at 0 °C for 30

min, the latter reaction mixture was added dropwise to the former one. The resultant mixture was stirred at room temperature for 40 min and was then filtered through a pad of silica gel and concentrated. The residue was purified by flash column chromatography (silica gel, Petroleum ether:EtOAc, 5:1) gave product **S43** (59 mg, 45%) as llight yellow solid.

TLC (silica gel, 1:1, EtOAc:Petroleum ether): $R_f = 0.6$ (UV, $KMnO_4$)

^{1}H NMR (500 MHz, $CDCl_3$): δ 7.66 – 7.64 (m, 2H), 7.42 – 7.38 (m, 1H), 7.31 (t, J = 7.5 Hz, 3H), 6.01 (d, J = 5.0 Hz, 1H), 5.86 (dd, J = 9.8, 1.6 Hz, 1H), 5.56 – 5.53 (m, 1H), 5.33 (s, 1H), 5.10 (s, 1H), 4.40 (d, J = 10.6 Hz, 1H), 4.21 (d, J = 10.6 Hz, 1H), 3.96 (d, J = 8.9 Hz, 1H), 3.54 (d, J = 8.9 Hz, 1H), 3.51 (d, J = 4.6 Hz, 1H), 2.76 (dt, J = 15.9, 2.6 Hz, 1H), 2.61 (d, J = 5.1 Hz, 1H), 2.50 (dd, J = 15.7, 2.3 Hz, 1H), 2.27 – 2.15 (m, 2H), 2.08 – 1.98 (m, 1H), 1.83 – 1.79 (m, 1H), 1.73 – 1.64 (m, 3H), 1.16 (s, 3H).

^{13}C NMR (126 MHz, $CDCl_3$): δ 207.3, 197.0, 171.4, 151.4, 142.7, 140.4, 129.5, 129.1, 127.0, 114.4, 113.4, 110.7, 77.5, 73.8, 70.4, 68.2, 57.8, 56.4, 51.7, 43.0, 41.0, 40.8, 29.9, 29.6, 22.7.

HRMS (ESI, m/z): cacl'd for $C_{27}H_{29}O_6Te$ $[M+H]^+$: 579.1021, found: 579.1010.

$[\alpha]_D^{22.1}$ = +33.3° (c = 0.21, MeOH).

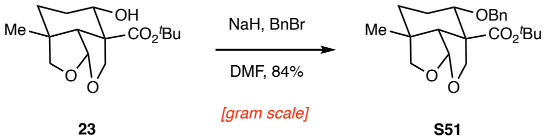

23 **S51**

A solution of **23** (1.38 g, 4.85 mmol, 1.0 equiv) in anhydrous DMF (5 mL) was added dropwise to a cooled (0 °C) suspension of NaH (60%, 0.58 g, 14.56 mmol, 3.0 equiv) in anhydrous DMF (10 mL). After stirring at 0 °C for 30 min, benzyl bromide (BnBr, 1.24 g/0.86 mL, 7.28 mmol, 1.5 equiv) was added. The reaction mixture was warmed to room temperature and stirred for 2.5 h. The reaction mixture was quenched with sat. aq. NH_4Cl (20 mL), diluted with H_2O (40 mL). The resulting mixture was extracted with EtOAc (60 mL). The organic phase was washed with H_2O (40 mL×2), brine (40 mL×1) whereby the aqueous layers were back-extracted with EtOAc (40 mL×2). The combined organic layers were washed, dried over Na_2SO_4, filtrated, and concentrated under reduced pressure. Purification by column chromatography (silica gel, Petroleum ether:EtOAc, 10:1) gave **S51** (1.52 g, 84%) as white amorphous powder.

TLC (silica gel, 1:2, EtOAc:Petroleum ether): $R_f = 0.6$ (UV, phosphomolybdic acid).

^{1}H NMR (400 MHz, $CDCl_3$): δ 7.33 – 7.24 (m, 5H), 5.81 (d, J = 5.3 Hz, 1H), 4.58 (d, J = 11.6 Hz, 1H), 4.49 (d, J = 9.8 Hz, 1H), 4.44 (d, J = 11.5 Hz, 1H), 4.12 – 4.04 (m, 1H), 3.97 (d, J = 9.8 Hz, 1H), 3.86 (d, J = 8.7 Hz, 1H), 3.41 (d, J = 8.7 Hz, 1H), 2.61 (d, J = 5.2 Hz, 1H), 1.95 – 1.86 (m, 1H), 1.83 – 1.75 (m, 1H), 1.63 – 1.52 (m, 2H), 1.45 (s, 9H), 1.11 (s, 3H).

^{13}C NMR (101 MHz, CDCl$_3$): δ 174.7, 138.4, 128.3, 127.6, 127.3, 110.4, 81.5, 80.2, 76.8, 71.5, 70.7, 58.8, 57.8, 40.4, 30.4, 30.3, 27.9, 22.3.

HRMS (ESI, m/z): cacl'd for $C_{22}H_{30}O_5Na$ $[M+Na]^+$: 397.1985, found: 397.1980.

$[\alpha]_D^{23.2}$ = +16.2° (c = 0.96, MeOH).

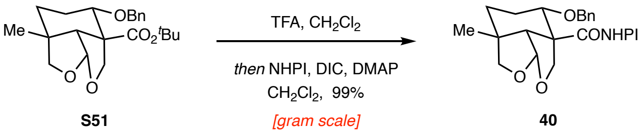

TFA (8.5 mL) was added dropwise to a cooled (0 °C) solution of **S51** (5.98 g, 15.97 mmol, 1.0 equiv) in CH_2Cl_2 (43 mL). After warming to room temperature and stirring for 4 h, the reaction mixture was concentrated under reduced pressure directly to give the crude carboxylic acid as yellow powder, which was used directly for the next step without further purification.

Note: To rapidly remove TFA completely, the above crude product can be dissolved in toluene and concentrated under vaccum, this process can be repeated until no TFA could be detected by ^{19}F NMR.

N,N-diisopropylcarbodiimide (DIC, 2.22g/2.72 mL, 17.6 mmol, 1.1 equiv) was added dropwise to a cooled (0 °C) mixture of above crude carboxylic acid (assumed 15.97 mmol, 1.0 equiv), NHPI (2.87 g, 17.57 mmol, 1.1 equiv), and DMAP (0.98 g, 8.0 mmol, 0.5 equiv) in anhydrous CH_2Cl_2 (80 mL). After warming to room temperature and stirring for 8 h, the reaction mixture was concentrated under reduced pressure. Purification by column chromatography (silica gel, CH_2Cl_2 to CH_2Cl_2:EtOAc, 10:1) gave RAE **40** (7.32 g, 99%) as light yellow powder.

TLC (silica gel, 2:1, Petroleum ether:EtOAc): R_f = 0.6 (UV, phosphomolybdic acid).

^{1}H NMR (400 MHz, CDCl$_3$): δ 7.90 (dd, *J* = 5.6, 3.0 Hz, 2H), 7.81 (dd, *J* = 5.5, 3.1 Hz, 2H), 7.38 – 7.27 (m, 5H), 6.00 (d, *J* = 5.2 Hz, 1H), 4.70 (d, *J* = 11.2 Hz, 1H), 4.64 (d, *J* = 6.7 Hz, 1H), 4.61 (d, *J* = 6.3 Hz, 1H), 4.41 – 4.31 (m, 1H), 4.15 (d, *J* = 10.5 Hz, 1H), 3.93 (d, *J* = 8.8 Hz, 1H), 3.50 (d, *J* = 8.9 Hz, 1H), 2.98 (d, *J* = 5.4 Hz, 1H), 2.03 – 1.94 (m, 1H), 1.85 (m, 1H), 1.71 – 1.61 (m, 2H), 1.19 (s, 3H).

^{13}C NMR (101 MHz, CDCl$_3$): δ 171.9, 162.0, 138.1, 135.0, 129.1, 128.4, 127.9, 127.8, 124.1, 110.6, 79.1, 77.0, 72.2, 70.6, 57.8, 57.6, 40.6, 30.3, 30.2, 22.5.

HRMS (ESI, m/z): cacl'd for $C_{26}H_{25}NO_7Na$ $[M+Na]^+$: 486.1523, found: 486.1517.

$[\alpha]_D^{23.0}$ = +19.2° (c = 0.20, MeOH).

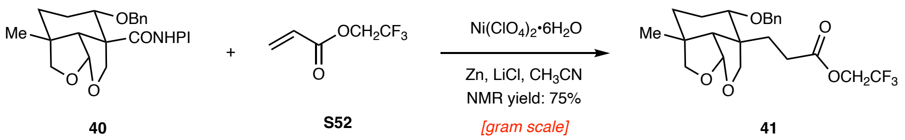

To a mixture of **40** (3.0 g, 6.46 mmol, 1.0 equiv), **S52** (20 g/16.4 mL, 129.2 mmol, 20.0 equiv), $Ni(ClO_4)_2·6H_2O$ (472 mg, 1.29 mmol, 0.2 equiv), LiCl (822 mg, 19.38 mmol, 3.0 equiv), and Zn (844 mg, 12.92 mmol, 2.0 equiv) was added anhydrous CH_3CN (30 mL) under argon. After heating to 35 °C and stirring for 19 h, the reaction mixture was filtrated through celite and washed with EtOAc (40 mL). The filtrate was washed with sat. aq. NH_4Cl (50 mL), the aqueous layer was extracted with EtOAc (30 mL×2). The combined organic layers were washed with brine, filtrated, and concentrated under reduced pressure. Purification by a flash column chromatography (silica gel, Petroleum ether:EtOAc, 10:1) gave **41** which was contaminated with phthalimide. To remove the excess phthalimide, the mixture was dissolved in CH_2Cl_2 (1 mL), filtrated, and washed with CH_2Cl_2 (1 mL). The filtrate was concentrated under reduced pressure. This process was repeated for 6 times, which can remove most of the phthalimide. Finally, 2.1 g product **41** was provided which contains a few phthalimide (NMR ratio: 1: 20). The yield of **41** was determined by NMR (75%).

*Note: **41** is not stable to silica gel.*

TLC (silica gel, 1:3, EtOAc:Petroleum ether): $R_f = 0.5$ (UV, phosphomolybdic acid).

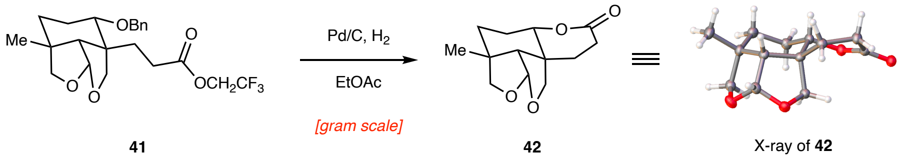

The round-bottom flask charged with the mixture of **41** and phthalimide (NMR ratio: 20:1, 4.05 g, assumed 9.3 mmol, 1.0 equiv) and Pd/C (10%, 1.0 g, 30% w.t.) was evacuated and backfilled with argon for 3 times before being evacuated and backfilled with H_2 for 3 times. EtOAc (500 mL) was added to the flask and the reaction mixture was stirred at room temperature for 8 days under the atmosphere of H_2. The reaction mixture was filtrated through a short plug of celite® and washed with EtOAc (100 mL). The filtrate was concentrated under reduced pressure to give crude product of **42** (2.21 g) as amorphous powder, which was used directly for the next step without further purification.

*For characterization of **42**, 10 mg of crude product **42** was further purified by pre-TLC (silica gel, 1:1, EtOAc:Petroleum ether). The structure was confirmed by X-ray crystallography.*

TLC (silica gel, 1:1, EtOAc:Petroleum ether): $R_f = 0.3$ (phosphomolybdic acid).

^{1}H NMR (400 MHz, $CDCl_3$): δ 5.90 (d, J = 5.2 Hz, 1H), 4.41 (m, 1H), 4.00 (dd, J = 9.7, 2.4 Hz, 1H), 3.89 (d, J = 8.8 Hz, 1H), 3.65 (d, J = 9.7 Hz, 1H), 3.48 (d, J = 8.8 Hz, 1H), 2.84 – 2.65

(m, 2H), 2.10 (m, 1H), 2.05 (d, J = 5.4 Hz, 1H), 1.94 – 1.82 (m, 3H), 1.74 – 1.64 (m, 1H), 1.64 – 1.55 (m, 1H), 1.10 (s, 3H).

^{13}C NMR (101 MHz, CDCl$_3$): δ 171.3, 110.4, 81.2, 77.4, 71.0, 60.1, 45.5, 41.4, 32.6, 30.5, 30.3, 27.8, 23.0.

HRMS (ESI, m/z): cacl'd for $C_{13}H_{19}O_4$ [M+H]$^+$: 239.1278, found: 239.1275.

$[\alpha]_D^{24.2}$ = –73.9° (c = 0.17, MeOH).

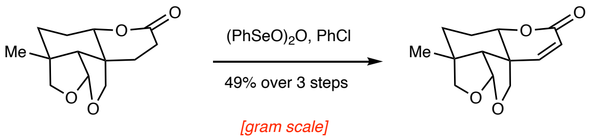

42 **S53**

A sealed tube charged with **42** (2.21 g, assumed 9.3 mmol, 1.0 equiv), $(PhSeO)_2O$ (5.02 g, 13.95 mmol, 1.5 equiv), and anhydrous chlorobenzene (PhCl, 60 mL) was heated to 135 °C. After 3 h stirring, the reaction mixture was cooled to room temperature and concentrated under reduced pressure. Purification by column chromatography (silica gel, Petroleum ether:EtOAc, 2:1) gave **S53** (1.43 g, 49% from **40** over 3 steps) as white amorphous powder.

TLC (silica gel, 1:1, EtOAc:Petroleum ether): R_f = 0.5 (UV, phosphomolybdic acid).

^{1}H NMR (400 MHz, CDCl$_3$): δ 6.96 (d, J = 9.5 Hz, 1H), 5.90 (d, J = 9.5 Hz, 1H), 5.89 (d, J = 5.0 Hz, 1H), 4.46 (m, 1H), 4.22 (d, J = 9.3 Hz, 1H), 3.81 (d, J = 8.9 Hz, 1H), 3.72 (d, J = 9.3 Hz, 1H), 3.43 (d, J = 8.9 Hz, 1H), 2.29 (d, J = 5.0 Hz, 1H), 1.95 – 1.74 (m, 3H), 1.66 – 1.49 (m, 1H), 1.05 (s, 3H).

^{13}C NMR (101 MHz, CDCl$_3$): δ 163.8, 157.1, 119.8, 109.9, 81.0, 77.8, 74.7, 56.8, 46.9, 40.9, 30.7, 30.2, 22.4.

HRMS (ESI, m/z): cacl'd for $C_{13}H_{17}O_4$ [M+H]$^+$: 237.1122, found: 237.1118.

$[\alpha]_D^{24.2}$ = +107.9° (c = 0.32, MeOH).

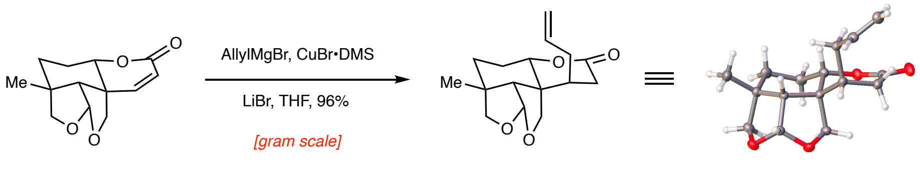

S53 **43**, single diastereomer X-ray of **43**

To a cooled (0 °C) suspension of CuBr·DMS (2.36 g, 11.5 mmol, 1.9 equiv) in anhydrous THF (20 mL) was added a solution of LiBr (1.05 g, 12.1 mmol, 2.0 equiv) in anhydrous THF (10 mL). After stirring for 20 min, the resulting mixture was cooled to –78 °C. After dropwise addition of AllylMgBr (1.0 M in 2-Me-THF, 12.7 mL, 12.7 mmol, 2.1 equiv), the reaction

mixture was stirred for 20 min. To this cooled mixture, a solution of **S53** (1.43 g, 6.05 mmol, 1.0 equiv) in THF (5 mL) was added dropwise. After 2.5 h stirring, the reaction mixture was quenched with sat. aq. NH_4Cl (50 mL) and extracted with EtOAc (50 mL×3). The combined organic layers were washed with brine, dried over Na_2SO_4, filtrated, and concentrated under reduced pressure. Purification by column chromatography (silica gel, Petroleum ether:EtOAc, 2:1) gave **43** (1.61 g, 96%) as light yellow amorphous powder.

*Note: The structure of **43** was confirmed by X-ray crystallography.*

TLC (silica gel, 1:1, EtOAc: Petroleum ether): R_f = 0.3 ($KMnO_4$).

^{1}H NMR (400 MHz, $CDCl_3$): δ 5.80 (d, J = 5.3 Hz, 1H), 5.74 – 5.61 (m, 1H), 5.16 – 5.07 (m, 2H), 4.37 (m, 1H), 4.02 (d, J = 9.8 Hz, 1H), 3.88 (d, J = 8.8 Hz, 1H), 3.53 (d, J = 9.8 Hz, 1H), 3.45 (d, J = 8.8 Hz, 1H), 2.74 – 2.62 (m, 1H), 2.43 (d, J = 5.5 Hz, 1H), 2.43 – 2.32 (m, 2H), 2.09 – 1.98 (m, 2H), 1.88 – 1.78(m, 3H), 1.55 – 1.39 (m, 1H), 1.08 (s, 3H).

^{13}C NMR (101 MHz, $CDCl_3$): δ 171.9, 134.4, 118.7, 110.7, 77.4, 77.1, 73.5, 53.5, 49.5, 41.0, 37.1, 36.7, 34.2, 30.6, 29.6, 22.8.

HRMS (ESI, m/z): cacl'd for $C_{16}H_{23}O_4$ $[M+H]^+$: 278.1591, found: 279.1589.

$[\alpha]_D^{25.1}$ = –69.1° (c = 0.21, MeOH).

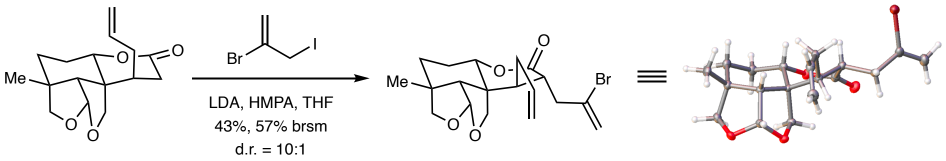

43 44 X-ray of 44

Freshly prepared lithium diisopropylamide (LDA, 0.5 M in THF, 4.0 mL, 2.0 equiv) was added dropwise to a cooled (–78 °C) solution of **43** (279 mg, 1.0 mmol, 1.0 equiv) in anhydrous THF (10 mL). After stirring for 3 h, hexamethylphosphoramide (HMPA, 1.8 g/1.8 mL, 2.0 mmol, 10.0 equiv) was added dropwise. The resulting mixture was continuing stirred at –78 °C for another 3 h followed by the addition of 2-bromo-3-iodopropene (1.23 g/0.52 mL, 5.0 mmol, 5.0 equiv) in one portion. The reaction mixture was slowly warmed to –4 °C over 20 h, before being quenched with sat. aq. NH_4Cl (10 mL), diluted with H_2O (10 mL) and extracted with EtOAc (10 mL×3). The combined organic layers were washed with brine, dried over Na_2SO_4, filtrated, and concentrated under reduced pressure. Purification by column chromatography (silica gel, Petroleum ether:*tert*-butyl methyl ether, 4:1) gave product **44** (171 mg, 43%, 57% brsm, d.r. = 10:1) as light yellow oil and recovered **43** (68 mg, 24%).

*Note: The structure of **44** was confirmed by X-ray crystallography.*

TLC (silica gel, 1:2, EtOAc:Petroleum ether): R_f = 0.5 (phosphomolybdic acid).

^{1}H NMR (400 MHz, CDCl$_3$): δ 5.94 – 5.85 (m, 2H), 5.81 (d, *J* = 5.3 Hz, 1H), 5.51 (d, *J* = 1.8 Hz, 1H), 5.31 – 5.24 (m, 2H), 4.52 – 4.47 (m, 1H), 4.03 (d, *J* = 9.9 Hz, 1H), 3.92 (d, *J* = 8.8 Hz, 1H), 3.50 (d, *J* = 8.8 Hz, 1H), 3.36 (d, *J* = 9.9 Hz, 1H), 3.23 – 3.19 (m, 1H), 2.86 – 2.81 (m, 1H), 2.65 (d, *J* = 5.3 Hz, 1H), 2.48 – 2.41 (m, 3H), 1.97 – 1.91 (m, 1H), 1.86 – 1.78 (m, 3H), 1.54 – 1.50 (m, 1H), 1.12 (s, 3H).

^{13}C NMR (101 MHz, CDCl$_3$): δ 173.3, 134.2, 131.2, 120.3, 119.4, 111.3, 77.5, 77.2, 74.5, 53.5, 51.1, 45.0, 43.45, 42.3, 41.2, 35.1, 30.6, 29.5, 22.9.

HRMS (ESI, m/z): cacl'd for $C_{19}H_{26}BrO_4$ $[M+H]^+$: 397.1009, found: 397.1005.

$[\alpha]_D^{23.8}$ = –81.5° (c = 0.14, MeOH).

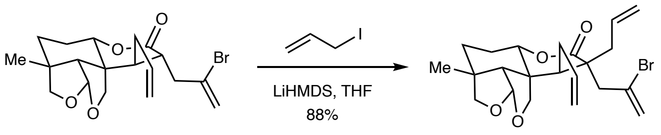

44 **S54**

To a cooled (0 °C) solution of **44** (136 mg, 0.34 mmol. 1.0 equiv) in anhydrous THF (3 mL) was added lithium bis(trimethylsilyl)amide (LiHMDS, 1.0 M in THF, 1.03 mL, 1.03 mmol, 3.0 equiv) dropwise. After stirring for 2 h, allyl iodide (457 mg/ 248 mL, 2.72 mmol, 8.0 equiv) was added to the resulting mixture in one portion. The reaction mixture was stirred for another 2 h before being quenched with sat. aq. NH_4Cl (10 mL) and extracted with EtOAc (10 mL×3). The combined organic layers were washed brine, filtrated, and concentrated under reduced pressure. Purification by column chromatography (silica gel, Petroleum ether:EtOAc, 5:1) gave the **S54** (130 mg, 88%) as light yellow amorphous powder.

TLC (silica gel, 1:2, EtOAc:Petroleum ether): R_f = 0.45 (UV).

^{1}H NMR (400 MHz, CDCl$_3$): δ 5.92 – 5.82 (m, 2H), 5.72 (d, *J* = 5.3 Hz, 1H), 5.69 (d, *J* = 1.7 Hz, 1H), 5.68 – 5.64 (m, 1H), 5.22 – 5.10 (m, 4H), 4.72 – 4.68 (m, 1H), 4.02 (d, *J* = 9.7 Hz, 1H), 3.89 (d, *J* = 8.8 Hz, 1H), 3.48 (t, *J* = 9.3 Hz, 2H), 3.04 (d, *J* = 15.1 Hz, 1H), 2.84 (m, 1H), 2.79 (s, 1H), 2.77 (d, *J* = 8.8 Hz, 1H), 2.57 – 2.47 (m, 3H), 2.34 – 2.18 (m, 1H), 1.97 – 1.88 (m, 1H), 1.87 – 1.79 (m, 2H), 1.58 – 1.53 (m, 1H), 1.07 (s, 3H).

^{13}C NMR (101 MHz, CDCl$_3$): δ 173.7, 138.0, 132.1, 127.8, 123.9, 120.1, 116.7, 111.2, 77.8, 76.5, 74.2, 53.4, 52.2, 51.4, 46.9, 44.0, 41.0, 37.3, 31.7, 30.5, 29.4, 23.2.

HRMS (ESI, m/z): cacl'd for $C_{22}H_{30}BrO_4$ $[M+H]^+$: 437.1322, found: 437.1317.

$[\alpha]_D^{22.8}$ = –28.1° (c = 0.55, MeOH).

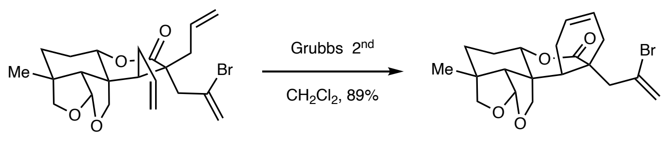

S54 | **45**, single diastereomer

A mixture of **S54** (130 mg, 0.3 mmol, 1.0 equiv) and Grubbs 2nd (25 mg, 0.03 mmol, 0.1 equiv) in CH_2Cl_2 (60 mL, 0.005 M) was stirred at room temperature for 4h before being concentrated under reduced pressure. Purification by column chromatography (silica gel, Petroleum ether:EtOAc, 5:1) gave **45** (109 mg, 89%) as light yellow amorphous powder.

TLC (silica gel, 1:2, EtOAc:Petroleum ether, twice): R_f = 0.5 ($KMnO_4$).

^{1}H NMR (400 MHz, $CDCl_3$): δ 6.07 – 6.02 (m, 1H), 5.968 – 5.93 (m, 1H), 5.88 (s, 1H), 5.86 (d, J = 5.3 Hz, 1H), 5.69 (s, 1H), 4.66 –4.62 (m, 1H), 4.01 (d, J = 9.9 Hz, 1H), 3.92 (d, J = 8.8 Hz, 1H), 3.75 (d, J = 9.8 Hz, 1H), 3.49 (d, J = 8.8 Hz, 1H), 3.31 (d, J = 14.6 Hz, 1H), 2.64 (d, J = 14.7 Hz, 1H), 2.60 – 2.57 (m, 1H), 2.47 – 2.41 (m, 1H), 2.39 (d, J = 5.3 Hz, 1H), 2.30 – 2.18 (m, 2H), 1.93 – 1.80 (m, 3H), 1.60 – 1.46 (m, 2H), 1.12 (s, 3H).

^{13}C NMR (101 MHz, $CDCl_3$): δ 175.1, 128.6, 127.2, 127.0, 124.1, 110.6, 77.5, 76.8, 72.9, 53.9, 50.6, 49.5, 49.0, 41.1, 37.7, 35.8, 30.8, 29.7, 25.2, 22.9.

HRMS (ESI, m/z): cacl'd for $C_{20}H_{26}BrO_4$ $[M+H]^+$: 409.1009, found: 409.1005.

$[\alpha]_D^{23.8}$ = –67.6° (c = 0.07, MeOH).

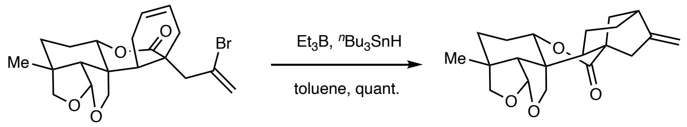

45 | **S55**

To a cooled (–78 °C) solution of **45** (109 mg, 0.26 mmol, 1.0 equiv) in anhydrous toluene (10 mL) was added ^{n}Bu$_3$SnH (180 mg/170 μL, 0.62 mmol, 2.4 equiv) under argon. A solution of Et_3B (1.0 M in hexane, 310 μL, 0.31 mmol, 1.2 equiv) was diluted with anhydrous toluene (2 mL) before being added slowly to the mixture of **45** and ^{n}Bu$_3$SnH. After stirring at –78 °C for 17 h, the reaction mixture was concentrated under reduced pressure. Purification by column chromatography (silica gel, Petroleum ether:EtOAc, 5:1) gave **S55** (88 mg, quant.) as white amorphous powder.

Note: The trace amount of O_2 in argon balloon could initiate the reaction.

TLC (silica gel, 1:2, EtOAc:Petroleum ether, twice): R_f = 0.5 ($KMnO_4$).

^{1}H NMR (400 MHz, $CDCl_3$): δ 5.81 (d, J = 5.3 Hz, 1H), 5.01 – 4.89 (m, 1H), 4.90 – 4.88 (m, 1H), 4.56 – 4.53 (m, 1H), 4.03 (d, J = 9.8 Hz, 1H), 3.90 (d, J = 8.8 Hz, 1H), 3.54 (d, J = 9.8 Hz, 1H), 3.47 (d, J = 8.8 Hz, 1H), 3.22 (dt, J = 16.1, 3.0 Hz, 1H), 2.79 – 2.75 (m, 1H), 2.35 (d, J =

5.4 Hz, 1H), 2.30 – 2.15 (m, 2H), 2.01 – 1.90 (m, 2H), 1.90 – 1.76 (m, 3H), 1.64 – 1.55 (m, 2H), 1.52 – 1.42 (m, 2H), 1.41 – 1.31 (m, 1H), 1.09 (s, 3H).

^{13}C NMR (101 MHz, CDCl$_3$): δ 176.1, 155.4, 110.9, 107.5, 77.2, 75.9, 72.6, 53.2, 50.9, 49.1, 47.5, 45.1, 41.30, 39.0, 37.4, 31.1, 30.7, 29.4, 23.1, 19.0.

HRMS (ESI, m/z): cacl'd for $C_{20}H_{27}O_4$ [M+H]$^+$: 331.1904, found: 331.1900.

$[\alpha]_D^{23.5}$ = –54.7° (c = 0.30, MeOH).

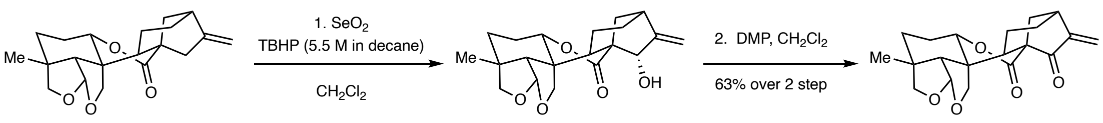

S55 **S56** macrocalyxoformin B (**6**)

To the sealed tube charged with the mixture of **S55** (10.0 mg, 0.03 mmol, 1.0 equiv) and SeO_2 (1 mg, 0.01 mmol, 0.3 equiv) in CH_2Cl_2 (0.5 mL) was added *tert*-butyl hydroperoxide (TBHP, 5.5 M in decane, 17 μL, 0.09 mmol, 3.0 equiv). After heating to 40 °C and stirring for 3 h, the reaction mixture was cooled to room temperature, quenched with sat. aq. $Na_2S_2O_3$ (1 mL). The resulting mixture was extracted with CH_2Cl_2 (1 mL×3) and EtOAc (1 mL×3) successively. The combined CH_2Cl_2 layers and the combined EtOAc layers were washed with brine respectively. The organic phases were combined, dried over Na_2SO_4, filtrated, and concentrated under reduced pressure. The crude product **S56** was provided as yellow oil which was used directly for the next step without further purification.

*Note: Comparing the crude ^{1}H NMR data of **S56** with natural macrocalyxoformin A (**5**) to confirm the stereoselectivity of oxidation.*

Dess-Martin periodinane (DMP, 32 mg, 0.075 mmol, 2.5 equiv) was added slowly to a cooled (0 °C) solution of the above crude product **S56** (assumed 0.03 mmol) in anhydrous CH_2Cl_2 (0.5 mL). After 5 h stirring at room temperature, the reaction mixture was quenched with sat. aq. $NaHCO_3$ (1 mL) and extracted with CH_2Cl_2 (1 mL×3). The combined organic layers were washed brine, dried over Na_2SO_4, filtrated, and concentrated under reduced pressure. Purification by pre-TLC (silica gel, Petroleum ether:EtOAc, 1:1) gave macrocalyxoformin B (**6**) (6.5 mg, 63% over 2 steps) as white amorphous powder.

*Note: The structure of macrocalyxoformin B (**6**) was confirmed by X-ray crystallography.*

TLC (silica gel, 1:1, EtOAc:Petroleum ether, twice): R_f = 0.3 (UV).

^{1}H NMR (400 MHz, CDCl$_3$): δ 6.07 (s, 1H), 5.81 (d, *J* = 5.3 Hz, 1H), 5.50 (s, 1H), 4.47 (dd, *J* = 11.4, 5.3 Hz, 1H), 4.04 (d, *J* = 10.0 Hz, 1H), 3.92 (d, *J* = 8.8 Hz, 1H), 3.78 (d, *J* = 10.2 Hz, 1H), 3.49 (d, *J* = 8.6 Hz, 1H), 3.12 (m, 1H), 2.38 (m, 2H), 2.31 (d, *J* = 5.3 Hz, 1H), 2.10 – 2.05 (m, 1H), 2.02 – 1.81 (m, 4H), 1.72 – 1.64 (m, 2H), 1.56 – 1.50 (m, 2H), 1.10 (s, 3H).

^{1}H NMR (400 MHz, C_5D_5N): δ 6.06 (d, J = 5.3 Hz, 1H), 6.02 (s, 1H), 5.34 (s, 1H), 4.68 (dd, J = 11.5, 5.3 Hz, 1H), 4.26 (d, J = 9.5 Hz, 1H), 4.12 (d, J = 9.5 Hz, 1H), 3.97 (d, J = 8.8 Hz, 1H), 3.43 (d, J = 8.8 Hz, 1H), 2.90 (dd, J = 9.4, 4.5 Hz, 1H), 2.52 (d, J = 11.9 Hz, 1H), 2.37 (d, J = 5.3 Hz, 1H), 2.18 – 2.09 (m, 2H), 2.06 – 1.96 (m, 1H), 1.95 – 1.89 (m, 1H), 1.86 – 1.80 (m, 1H), 1.62 – 1.54 (m, 2H), 1.42 (m, 1H), 1.33 – 1.23 (m, 2H), 1.03 (s, 3H).

^{13}C NMR (101 MHz, C_5D_5N): δ 200.4, 171.4, 151.1, 118.2, 111.5, 77.1, 76.4, 72.9, 56.6, 53.3, 51.0, 43.0, 41.4, 35.1, 32.6, 30.4, 29.7, 29.2, 23.2, 19.4.

HRMS (ESI, m/z): cacl'd for $C_{20}H_{25}O_4$ $[M+H]^+$: 345.1697, found: 345.1692

$[\alpha]_D^{25.3}$ = –97.0° (c = 0.10, $CHCl_3$).

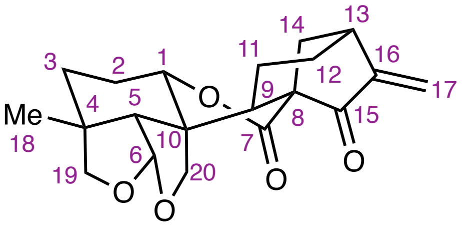

(–)-macrocalyxoformin B (**6**)

Supplementary Table 1. ^{1}H NMR data comparison of natural and synthetic (–)-macrocalyxoformin B[22]

Natural (–)-macrocalyxoformin B	Synthetic (–)-macrocalyxoformin B
**^{1}H NMR ($CDCl_3$)**<br>**δ [ppm, mult, *J* (Hz)]**	**^{1}H NMR (400 MHz, $CDCl_3$)**<br>**δ [ppm, mult, *J* (Hz)]**
6.28 (s, 1H)	6.07 (s, 1H)
6.01 (d, *J* = 5.2 Hz, 1H)	5.81 (d, *J* = 5.3 Hz, 1H)
5.68 (s, 1H)	5.50 (s, 1H)
4.64 (q, J= 6.0, 11.0 Hz, 1H)	4.47 (dd, *J* =11.4, 5.3 Hz, 1H)
4.21 (d, *J* = 9.0 Hz, 1H)	4.04 (d, *J* = 10.0 Hz, 1H)
4.08 (d, *J* = 10.0 Hz, 1H)	3.92 (d, *J* = 8.8 Hz, 1H)
3.91 (d, *J* = 9.0 Hz, 1H)	3.78 (d, *J* = 10.2 Hz, 1H)
3.61 (d, *J* = 10.0 Hz, 1H)	3.49 (d, *J* = 8.8 Hz, 1H)
3.25 (m, 1H)	3.12 (m, 1H)
	2.38 (m, 2H)
2.40 (d, *J* = 5.2 Hz, 1H)	2.31 (d, *J* = 5.3 Hz, 1H)
	2.10 – 2.05 (m, 1H)<br>2.02 – 1.81 (m, 4H)<br>1.72 – 1.64 (m, 2H)<br>1.56 – 1.50 (m, 2H)
1.14 (s, 3H)	1.10 (s, 3H)

Note: The original isolation papers only provide partial ^{1}H NMR data which was shown as above.

Supplementary Table 2. ^{13}**C NMR data comparison of natural and synthetic (–)-macrocalyxoformin B**[22]

	Natural (–)-macrocalyxoformin B	Synthetic (–)-macrocalyxoformin B
**C**	^{13}C NMR (125 MHz, C_5D_5N)<br>δ (ppm)	^{13}C NMR (101 MHz, C_5D_5N)<br>δ (ppm)
**1**	76.3	76.4
**2**	23.2	23.2
**3**	29.1	29.2
**4**	41.3	41.4
**5**	53.3	53.3
**6**	111.4	111.5
**7**	171.2	171.4
**8**	56.5	56.6
**9**	42.9	43.0
**10**	50.9	51.0
**11**	19.3	19.4
**12**	29.6	29.7
**13**	35.0	35.1
**14**	32.5	32.6
**15**	200.3	200.4
**16**	151.0	151.1
**17**	118.0	118.2
**18**	30.3	30.4
**19**	77.0	77.1
**20**	72.8	72.9

Supplementary Table 3. Specific rotation comparison of natural and synthetic (–)-macrocalyxoformin B[22]

	Natural (–)-macrocalyxoformin B	Synthetic (–)-macrocalyxoformin B
$[\alpha]^{t}_{D}$	$[\alpha]^{6}_{D}$ = –164.9° (c = 1.00, $CHCl_3$)	$[\alpha]^{25.3}_{D}$ = –97.0° (c = 0.10, $CHCl_3$)

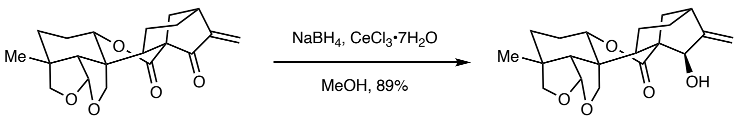

(–)-macrocalyxoformin B (**6**) (–)-macrocalyxoformin A (**5**)

Anhydrous MeOH (0.5 mL) was added to a mixture of macrocalyxoformin B (**6**) (5.0 mg, 14.5 μmol, 1.0 equiv), $NaBH_4$ (5.5 mg, 0.145 mmol, 10.0 equiv), and $CeCl_3 \cdot 7H_2O$ (54 mg, 0.145 mmol, 10.0 equiv) at 0 °C. After stirring at 0 °C for 10 min, the reaction was quenched with acetone (0.5 mL). The resulting mixture was diluted with Et_2O (5 mL), dried over Na_2SO_4, filtrated, and concentrated under reduced pressure. Purification by pre-TLC (silica gel, Petroleum ether:EtOAc, 1:1) gave macrocalyxoformin A (**5**) (4.5 mg, 89%) as white amorphous powder.

TLC (silica gel, 2:1, EtOAc:Petroleum ether): R_f = 0.3 (phosphomolybdic acid).

^{1}H NMR (400 MHz, $CDCl_3$): δ 5.83 (d, *J* = 5.4 Hz, 1H), 5.19 – 5.12 (m, 2H), 5.07 – 5.01 (m, 1H), 4.54 (dd, *J* = 11.6, 5.3 Hz, 1H), 3.96 (d, *J* = 9.4 Hz, 1H), 3.92 (d, *J* = 8.7 Hz, 1H), 3.73 (d, *J* = 9.4 Hz, 1H), 3.47 (d, *J* = 8.7 Hz, 1H), 2.80 – 2.71 (m, 1H), 2.38 (d, *J* = 5.9 Hz, 2H), 2.23 (m, 2H), 1.98 – 1.89 (m, 1H), 1.87 – 1.79 (m, 2H), 1.67 – 1.60 (m, 2H), 1.57 – 1.44 (m, 3H), 1.09 (s, 3H).

^{13}C NMR (101 MHz, $CDCl_3$): δ 175.3, 156.5, 110.8, 109.4, 78.2, 77.1, 75.9, 72.2, 53.1, 52.1, 50.5, 41.2, 36.3, 35.4, 32.6, 32.2, 30.6, 29.3, 22.9, 18.6.

HRMS (ESI, m/z): cacl'd for $C_{20}H_{27}O_5$ $[M+H]^+$: 347.1853, found: 347.1854.

$[\alpha]_D^{24.4}$ = –20.0° (c = 0.025, MeOH).

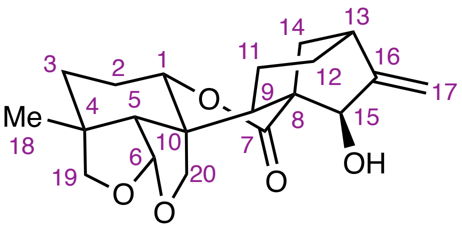

(–)-macrocalyxoformin A (**5**)

Supplementary Table 4. ^{1}H NMR data comparison of natural and synthetic (–)-macrocalyxoformin A[23]

Natural (–)-macrocalyxoformin A		Synthetic (–)-macrocalyxoformin A
^{1}H NMR (90 MHz, $CDCl_3$)<br>δ [ppm, mult, *J* (Hz)]	^{1}H NMR (60 MHz, $CDCl_3$)<br>δ [ppm, mult, *J* (Hz)]	^{1}H NMR (400 MHz, $CDCl_3$)<br>δ [ppm, mult, *J* (Hz)]
6.00 (d, *J* = 5.2 Hz, 1H)	5.81 (d, *J* = 5.2 Hz, 1H)	5.83 (d, *J* = 5.4 Hz, 1H)
5.32 (m, 2H)	5.15 – 5.01 (m, 3H)	5.19 – 5.12 (m, 2H)
5.17 (br, 1H)		5.07 – 5.01 (m, 1H)
4.67<br>(dd, *J* = 7.0, 9.0 Hz, 1H)	4.53 (m, 1H)	4.54<br>(dd, *J* = 11.6, 5.3 Hz, 1H)
4.10 (d, *J* = 10.0 Hz, 1H)	3.97 (d, *J* = 10.0 Hz, 1H)	3.96 (d, *J* = 9.4 Hz, 1H)
4.05 (d, *J* = 9.0 Hz, 1H)	3.92 (d, *J* = 9.0 Hz, 1H)	3.92 (d, *J* = 8.7 Hz, 1H)
3.85 (d, *J* = 10.0 Hz, 1H)	3.70 (d, *J* = 10.0 Hz, 1H)	3.73 (d, *J* = 9.4 Hz, 1H)
3.57 (d, *J* = 9.0 Hz, 1H)	3.64 (d, *J* = 9.0 Hz, 1H)	3.47 (d, *J* = 8.7 Hz, 1H)
2.9 (m, 1H)	2.71 (m, 1H)	2.80 – 2.71 (m, 1H)
	2.55 (s, 1H, 15-OH)	
2.48 (d, *J* = 5.2 Hz, 1H)	2.37 (d, *J* = 5.2 Hz, 1H)	2.38 (d, *J* = 5.9 Hz, 1H)
2.31 (d, *J* = 5.0 Hz, 1H)		2.38 (d, *J* = 5.9 Hz, 1H)
		2.23 (m, 2H)<br>1.98 – 1.89 (m, 1H)<br>1.87 – 1.79 (m, 2H)<br>1.67 – 1.60 (m, 2H)<br>1.57 – 1.44 (m, 3H)
1.14 (s, 3H)	1.09 (s, 3H)	1.09 (s, 3H)

Note: The original isolation papers only provide partial ^{1}H NMR data which was shown as above.

Supplementary Table 5. ^{13}C NMR data comparison of natural and synthetic (–)-macrocalyxoformin A[23]

	Natural (–)-macrocalyxoformin A	Synthetic (–)-macrocalyxoformin A
**C**	^{13}C NMR (23 MHz, $CDCl_3$) δ (ppm)	^{13}C NMR (101 MHz, $CDCl_3$) δ (ppm)
**1**	78.1	78.2
**2**	23.0	22.9
**3**	29.5	29.3
**4**	41.1	41.2
**5**	53.2	53.1
**6**	110.6	110.8
**7**	174.5	175.3
**8**	52.2	52.1
**9**	36.4	36.3
**10**	50.4	50.5
**11**	18.7	18.6
**12**	32.7	32.6
**13**	35.5	35.4
**14**	32.2	32.2
**15**	75.7	75.9
**16**	156.4	156.5
**17**	108.9	109.4
**18**	30.6	30.6
**19**	76.9	77.1
**20**	72.1	72.2

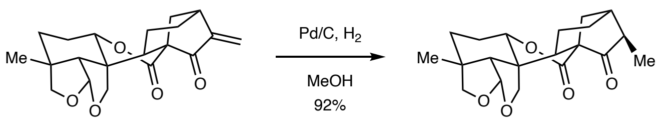

(–)-macrocalyxoformin B (**6**) (–)-ludongnin C (**7**)

A mixture of macrocalyxoformin B (**6**) (5.2 mg, 0.015 mmol, 1.0 equiv) and Pd/C (cat.) in MeOH (1.2 mL) was bubbled with H_2 for 15 min. Then the reaction mixture was stirred under H_2 for another 15 min. The reaction mixture was filtrated through a short plug of celite®, washed with CH_2Cl_2 (2 mL). The filtrate was concentrated under reduced pressure to give ludongnin C (**7**) (4.8 mg, 92%) as white amorphous powder.

Note: Ludongnin C (7) is unstable in silica gel.

^{1}H NMR (400 MHz, C_5D_5N): δ 6.05 (d, *J* = 5.2 Hz, 1H), 4.65 (dd, *J* = 11.5, 5.3 Hz, 1H), 4.25 (d, *J* = 9.4 Hz, 1H), 4.09 (d, *J* = 9.4 Hz, 1H), 3.96 (d, *J* = 8.8 Hz, 1H), 3.43 (d, *J* = 8.7 Hz, 1H), 2.57 (dd, *J* = 12.0, 1.5 Hz, 1H), 2.47 – 2.39 (m, 1H), 2.40 (m, 1H), 2.37 (d, *J* = 5.2 Hz, 1H), 2.02 (m, 1H), 1.92 (m, 2H), 1.83 (m, 1H), 1.72 (m, 1H), 1.62 (m, 1H), 1.49 – 1.40 (m, 3H), 1.29 (m, 1H), 1.03 (s, 3H), 1.00 (d, *J* = 6.9 Hz, 3H).

^{13}C NMR (101 MHz, C_5D_5N): δ 215.0, 171.9, 111.2, 76.9, 76.5, 73.0, 56.6, 53.0, 50.6, 49.4, 43.7, 41.1, 33.9, 32.6, 30.2, 29.0, 23.0, 19.12, 19.05, 10.2.

HRMS (ESI, m/z): cacl'd for $C_{20}H_{27}O_5$ $[M+H]^+$: 347.1853, found: 347.1853.

$[\alpha]_D^{22.3}$ = –43.9° (c = 0.07, MeOH).

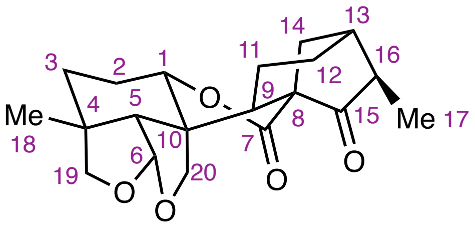

(–)-ludongnin C (**7**)

Supplementary Table 6. ^{1}H NMR data comparison of natural and synthetic (–)-ludongnin C[24]

Natural (–)-ludongnin C	Synthetic (–)-ludongnin C
^{1}H NMR (500 MHz, C_5D_5N)<br>δ [ppm, mult, *J* (Hz)]	^{1}H NMR (400 MHz, C_5D_5N)<br>δ [ppm, mult, *J* (Hz)]
6.03 (d, *J* = 5.2 Hz, 1H)	6.05 (d, *J* = 5.2 Hz, 1H)
4.64 (dd, *J* = 5.3, 11.6 Hz, 1H)	4.65 (dd, *J* = 11.5, 5.3 Hz, 1H)
4.23 (d, *J* = 9.0 Hz, 1H)	4.25 (d, *J* = 9.4 Hz, 1H)
4.06 (d, *J* = 9.0 Hz, 1H)	4.09 (d, *J* = 9.4 Hz, 1H)
3.96 (d, *J* = 8.7 Hz, 1H)	3.96 (d, *J* = 8.8 Hz, 1H)
3.42 (d, *J* = 8.7 Hz, 1H)	3.43 (d, *J* = 8.7 Hz, 1H)
2.59 (d, *J* = 12.0 Hz, 1H)	2.57 (dd, *J* = 12.0, 1.5 Hz, 1H)
2.43 (m, 1H)	2.47 – 2.39 (m, 1H)
2.41 (m, 1H)	2.40 (m, 1H)
2.37 (d, *J* = 5.2 Hz, 1H)	2.37 (d, *J* = 5.2 Hz, 1H)
2.10 (m, 1H), 2.07 (m, 1H)<br>1.86 (m, 1H), 1.86 – 1.78 (m, 2H)<br>1.72 (m, 1H)<br>1.70 – 1.34 (m, 1H),<br>1.62 (m, 1H), 1.40 (m, 1H)	2.02 (m, 1H)<br>1.92 (m, 2H), 1.83 (m, 1H)<br>1.72 (m, 1H)<br>1.62 (m, 1H), 1.49 – 1.40 (m, 3H),<br>1.29 (m, 1H)
1.04 (s, 3H)	1.03 (s, 3H)
0.99 (d, *J* = 6.9 Hz, 3H)	1.00 (d, *J* = 6.9 Hz, 3H)

Note: The original isolation papers only provide partial ^{1}H NMR data which was shown as above.

Supplementary Table 7. ^{13}C NMR data comparison natural and synthetic (–)-ludongnin C[24]

	Natural (–)-ludongnin C	Synthetic (–)-ludongnin C
**C**	^{13}C NMR (125 MHz, C_5D_5N) δ (ppm)	^{13}C NMR (101 MHz, C_5D_5N) δ (ppm)
**1**	76.5	76.5
**2**	23.0	23.0
**3**	29.1	29.0
**4**	41.4	41.1
**5**	53.0	53.0
**6**	111.3	111.2
**7**	171.9	171.9
**8**	56.6	56.6
**9**	43.7	43.7
**10**	50.6	50.6
**11**	19.2	19.12
**12**	19.2	19.05
**13**	32.7	32.6
**14**	33.9	33.9
**15**	215.0	215.0
**16**	49.4	49.4
**17**	10.3	10.2
**18**	30.3	30.2
**19**	77.0	76.9
**20**	73.1	73.0

Supplementary Table 8. Specific rotation comparison of natural and synthetic (–)-ludongnin C[24]

	Natural (–)-ludongnin C	Synthetic (–)-ludongnin C
$[\alpha]_D^t$	$[\alpha]_D^{20}$ = –110.0° (c = 0.82, MeOH)	$[\alpha]_D^{22.3}$ = –43.9° (c = 0.07, MeOH)

3.2 Single Crystal X-ray Diffraction Data

X-ray crystallographic data for 28

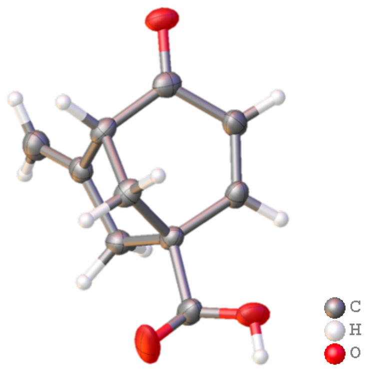

Supplementary Table 9. Crystal and refinement data for 28

CCDC number	2238607
Empirical formula	$C_{10}H_{10}O_3$
Formula weight	178.18
Temperature/K	100
Crystal system	orthorhombic
Space group	$P2_12_12_1$
a/Å	8.5543(2)
b/Å	8.6954(3)
c/Å	11.4663(3)
α/°	90
β/°	90
γ/°	90
Volume/Å^3	852.90(4)
Z	4
ρ_{calc}g/cm^3	1.388
μ/mm^{-1}	0.852
F(000)	376.0
Crystal size/mm^3	0.5 × 0.38 × 0.21
Radiation	CuKα (λ = 1.54184)
2Θ range for data collection/°	12.776 to 133.096
Index ranges	$-10 \le h \le 9, -10 \le k \le 10, -13 \le l \le 13$
Reflections collected	9862
Independent reflections	1493 [R_{int} = 0.0440, R_{sigma} = 0.0200]
Data/restraints/parameters	1493/7/129
Goodness-of-fit on F^2	1.050
Final R indexes [I>=2σ (I)]	R_1 = 0.0335, wR_2 = 0.0869

Final R indexes [all data]	R_1 = 0.0337, wR_2 = 0.0873
Largest diff. peak/hole / e Å^{-3}	0.21/–0.18
Flack parameter	0.11(10)

Supplementary Table 10. Fractional Atomic Coordinates (×10^4) and Equivalent Isotropic Displacement Parameters (Å^2×10^3) for 13. U(eq)is defined as 1/3 of of the trace of the orthogonalised U^{ij} tensor

Atom	x	y	z	U(eq)
O1	3772.9(17)	5792(2)	8884.2(14)	35.7(4)
O2	10719.6(17)	5768(2)	8541.7(15)	37.7(4)
C4	7759(2)	6851(2)	8660.8(19)	25.5(5)
C5	5695(2)	4855(2)	7526.6(19)	25.2(5)
C6	10007(3)	5673(3)	7538(2)	30.7(5)
C7	6320(3)	6774(3)	9106.2(19)	29.0(5)
C8	5138(2)	5782(3)	8561(2)	27.6(5)
C9	5864(2)	5985(2)	6520.1(19)	24.8(5)
C(A)	7539(2)	6548(3)	6498(2)	26.5(5)
C(B)	8251(2)	5888(2)	7630.0(19)	23.3(5)
C(C)	7395(2)	4324(2)	7719(2)	27.7(5)
C(D)	4745(3)	6387(3)	5792(2)	33.0(6)

X-ray crystallographic data for 42

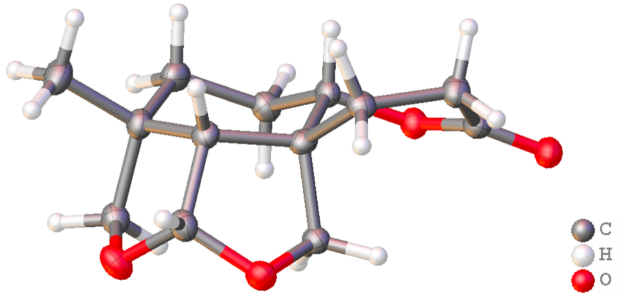

Supplementary Table 11. Crystal and refinement data for 42

CCDC number	2238608
Empirical formula	$C_{13}H_{18}O_4$
Formula weight	238.27
Temperature/K	100.01(10)
Crystal system	triclinic
Space group	P-1
a/Å	6.2829(2)
b/Å	7.4556(3)
c/Å	12.9124(6)
α/°	85.971(3)
β/°	76.505(4)
γ/°	73.279(3)
Volume/Å^3	563.28(4)
Z	2
ρ_{calc}g/cm^3	1.405
μ/mm^{-1}	0.851
F(000)	256.0
Crystal size/mm^3	0.27 × 0.13 × 0.05
Radiation	Cu Kα (λ = 1.54184)
2Θ range for data collection/°	7.04 to 152.676
Index ranges	$-7 \le h \le 7$, $-9 \le k \le 9$, $-14 \le l \le 15$
Reflections collected	18645
Independent reflections	2250 [R_{int} = 0.0642, R_{sigma} = 0.0413]
Data/restraints/parameters	2250/0/155
Goodness-of-fit on F^2	1.065
Final R indexes [I>=2σ (I)]	R_1 = 0.0636, wR_2 = 0.1741
Final R indexes [all data]	R_1 = 0.0729, wR_2 = 0.1798
Largest diff. peak/hole / e Å^{-3}	0.37/–0.29

Supplementary Table 12. Fractional Atomic Coordinates (×10^4) and Equivalent Isotropic Displacement Parameters (Å^2×10^3) for 31. U(eq)is defined as 1/3 of of the trace of the orthogonalised U^{ij} tensor

Atom	x	y	z	U(eq)
O8	7531(3)	4987(2)	5489.6(13)	22.7(4)
O14	2424(3)	3625(2)	7901.4(13)	25.2(4)
O16	9114(3)	2386(2)	4565.0(13)	25.4(4)
O2	322(3)	6428(2)	8812.2(14)	27.9(4)
C9	8626(4)	3144(3)	5423.1(19)	21.7(5)
C11	7581(4)	2846(3)	7483.4(18)	21.9(5)
C12	5718(4)	4634(3)	7329.7(18)	20.2(5)
C4	3406(4)	7759(3)	8392.1(19)	22.7(5)
C13	4406(4)	5596(3)	8405.6(18)	21.9(5)
C7	6893(4)	5853(3)	6538.4(18)	20.7(5)
C3	997(4)	7965(3)	8240(2)	24.5(5)
C15	3842(4)	4118(3)	6945.3(19)	21.5(5)
C6	5452(4)	7837(3)	6436.3(19)	23.1(5)
C10	9252(4)	2146(3)	6414.2(19)	25.4(5)
C1	2277(4)	4851(3)	8720(2)	24.7(5)
C5	4762(4)	8754(3)	7530(2)	25.1(5)
C17	3221(5)	8540(4)	9488(2)	31.6(6)

X-ray crystallographic data for 43

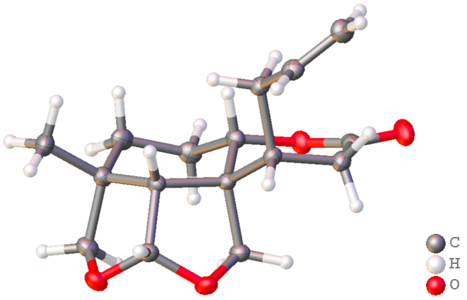

Supplementary Table 13. Crystal and refinement data for 43

CCDC number	2238609
Empirical formula	$C_{16}H_{22}O_4$
Formula weight	278.34
Temperature/K	100.00(10)
Crystal system	monoclinic
Space group	$P2_1$
a/Å	9.8313(2)
b/Å	6.82390(10)
c/Å	10.9824(2)
α/°	90.00
β/°	109.487(2)
γ/°	90.00
Volume/Å^3	694.58(2)
Z	2
ρ_{calc}g/cm^3	1.331
μ/mm^{-1}	0.768
F(000)	300.0
Crystal size/mm^3	0.28 × 0.06 × 0.03
Radiation	CuKα (λ = 1.54184)
2Θ range for data collection/°	8.54 to 144.2
Index ranges	$-11 \leq h \leq 12$, $-8 \leq k \leq 8$, $-13 \leq l \leq 13$
Reflections collected	8881
Independent reflections	2676 [R_{int} = 0.0250, R_{sigma} = 0.0204]
Data/restraints/parameters	2676/1/183
Goodness-of-fit on F^2	1.064
Final R indexes [I>=2σ (I)]	R_1 = 0.0291, wR_2 = 0.0776
Final R indexes [all data]	R_1 = 0.0297, wR_2 = 0.0781
Largest diff. peak/hole / e Å^{-3}	0.27/–0.15
Flack parameter	0.07(15)

Supplementary Table 14. Fractional Atomic Coordinates (×10^4) and Equivalent Isotropic Displacement Parameters (Å^2×10^3) for 32. U(eq)is defined as 1/3 of of the trace of the orthogonalised U^{ij} tensor

Atom	x	y	z	U(eq)
O4	-906.4(10)	8183.8(16)	1330.2(10)	25.9(2)
O15	1377.3(10)	8742.4(15)	1155.1(9)	24.4(2)
O10	4609.4(10)	6295.8(16)	4587.9(9)	23.3(2)
O16	6819.2(10)	6400.9(18)	4529.5(11)	30.8(3)
C1	2505.2(13)	6227(2)	2593.6(12)	16.6(3)
C11	5549.9(15)	6212(2)	3932.0(14)	22.9(3)
C13	3447.6(14)	5273(2)	1869.9(13)	18.1(3)
C2	891.2(14)	5680(2)	1975.6(13)	17.5(3)
C14	2452.8(14)	8458(2)	2395.3(13)	20.8(3)
C3	259.1(14)	7369(2)	1016.5(13)	21.1(3)
C18	4065.4(15)	2147(2)	939.7(14)	22.4(3)
C17	3479.2(14)	3008(2)	1919.6(13)	19.7(3)
C8	2250.8(15)	6249(2)	4824.9(13)	23.4(3)
C6	-51.0(15)	5659(2)	2865.7(14)	20.8(3)
C9	3122.0(14)	5591(2)	3999.9(13)	19.2(3)
C12	4997.5(15)	6030(2)	2472.6(14)	22.7(3)
C7	775.4(15)	5259(2)	4301.1(14)	24.0(3)
C5	-708.7(16)	7729(2)	2647.2(15)	24.6(3)
C19	5319.2(15)	1264(3)	1207.9(14)	26.6(3)
C20	-1248.0(15)	4136(2)	2360.5(16)	27.4(3)

X-ray crystallographic data for 44

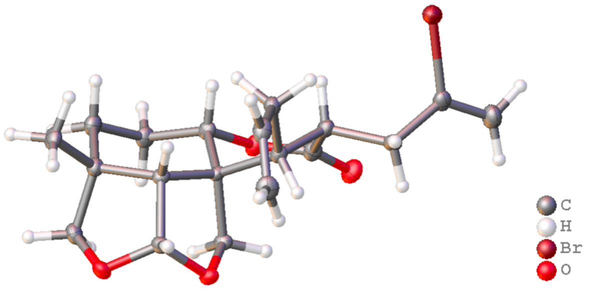

Supplementary Table 15. Crystal and refinement data of 44

CCDC number	2238610
Empirical formula	$C_{19}H_{25}BrO_4$
Formula weight	397.30
Temperature/K	100.00(10)
Crystal system	monoclinic
Space group	$P2_1$
a/Å	10.5744(2)
b/Å	6.51790(10)
c/Å	13.4598(3)
α/°	90
β/°	105.254(2)
γ/°	90
Volume/Å^3	895.00(3)
Z	2
ρ_{calc}g/cm^3	1.474
μ/mm^{-1}	3.294
F(000)	412.0
Crystal size/mm^3	0.25 × 0.03 × 0.02
Radiation	CuKα (λ = 1.54184)
2Θ range for data collection/°	6.806 to 153.488
Index ranges	$-12 \leq h \leq 13$, $-8 \leq k \leq 8$, $-16 \leq l \leq 15$
Reflections collected	22472
Independent reflections	3569 [R_{int} = 0.0538, R_{sigma} = 0.0313]
Data/restraints/parameters	3569/1/218
Goodness-of-fit on F^2	1.098
Final R indexes [I>=2σ (I)]	R_1 = 0.0287, wR_2 = 0.0723
Final R indexes [all data]	R_1 = 0.0302, wR_2 = 0.0731
Largest diff. peak/hole / e Å^{-3}	0.36/–0.50
Flack parameter	–0.007(11)

Supplementary Table 16. Fractional Atomic Coordinates (×10^4) and Equivalent Isotropic Displacement Parameters (Å^2×10^3) for 33. U(eq)is defined as 1/3 of of the trace of the orthogonalised U^{ij} tensor

Atom	x	y	z	U(eq)
Br01	9781.7(3)	10236.6(7)	7225.7(3)	30.27(13)
O002	6437(2)	322(5)	7562.9(15)	17.0(4)
O003	5680(2)	5588(4)	5697.5(16)	18.6(6)
O004	7382(2)	5449(6)	5051.5(17)	23.9(6)
O005	4612(2)	-242(3)	8215(2)	20.3(6)
C006	3260(3)	4873(6)	7264(3)	20.8(9)
C007	7488(3)	4666(5)	7721(2)	14.9(7)
C008	9288(3)	5903(5)	6928(3)	17.4(7)
C009	7807(3)	5857(5)	8744(3)	17.0(7)
C00A	8307(3)	4638(6)	9716(3)	22.3(9)
C00B	5462(3)	3156(5)	8218(2)	13.9(7)
C00C	9879(3)	7700(6)	6516(3)	20.5(7)
C00D	6978(3)	5639(5)	5812(3)	18.5(8)
C00E	10505(4)	7683(7)	5781(3)	27.4(9)
C00F	3779(3)	5074(8)	6307(2)	18.9(7)
C00G	7825(3)	6063(6)	6884(3)	17.2(7)
C00H	5797(3)	837(5)	8336(3)	16.6(7)
C00I	5237(3)	5497(7)	6648(2)	15.5(7)
C00J	3619(4)	3565(7)	9054(3)	23.3(8)
C00K	6086(3)	1831(5)	6759(3)	15.4(7)
C00L	8700(4)	2712(6)	9809(3)	24.3(8)
C00M	3552(4)	1031(6)	7649(3)	20.2(7)
C00N	6052(3)	3860(5)	7334(2)	12.9(6)
C00O	3946(3)	3207(6)	8020(3)	17.2(7)

X-ray crystallographic data for (–)-macrocalyxoformin B (6)

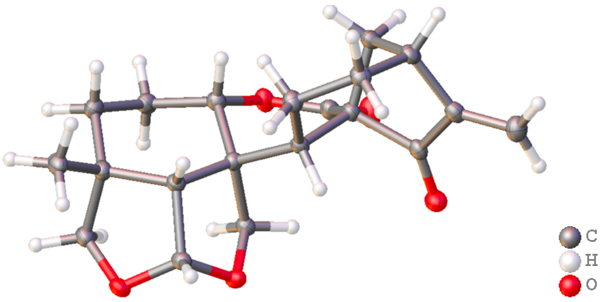

Supplementary Table 17. Crystal and refinement data for (–)-macrocalyxoformin B (6)

CCDC number	2238611
Empirical formula	$C_{20}H_{24}O_5$
Formula weight	344.39
Temperature/K	99.99(10)
Crystal system	orthorhombic
Space group	$P2_12_12_1$
a/Å	7.77845(7)
b/Å	12.99940(13)
c/Å	15.95018(15)
α/°	90
β/°	90
γ/°	90
Volume/Å^3	1612.81(3)
Z	4
ρ_{calc}g/cm^3	1.418
μ/mm^{-1}	0.827
F(000)	736.0
Crystal size/mm^3	0.25 × 0.18 × 0.08
Radiation	Cu Kα (λ = 1.54184)
2Θ range for data collection/°	8.776 to 153.634
Index ranges	-9 ≤ h ≤ 9, -16 ≤ k ≤ 16, -20 ≤ l ≤ 20
Reflections collected	29782
Independent reflections	3354 [R_{int} = 0.0382, R_{sigma} = 0.0155]
Data/restraints/parameters	3354/0/228
Goodness-of-fit on F^2	1.042
Final R indexes [I>=2σ (I)]	R_1 = 0.0275, wR_2 = 0.0720
Final R indexes [all data]	R_1 = 0.0280, wR_2 = 0.0723
Largest diff. peak/hole / e Å^{-3}	0.21/–0.15
Flack parameter	–0.04(6)

Supplementary Table 18. Fractional Atomic Coordinates (×10^4) and Equivalent Isotropic Displacement Parameters (Å^2×10^3) for macrocalyxoformin B (6). U(eq)is defined as 1/3 of of the trace of the orthogonalised U^{ij} tensor

Atom	x	y	z	U(eq)
O10	4775.6(16)	8761.4(9)	3196.8(7)	19.9(3)
O4	3077.3(15)	6696.9(10)	424.9(7)	20.1(3)
O19	5458.4(14)	6559.4(9)	1318.4(7)	18.8(3)
O22	8772.2(16)	6634.0(10)	3496.1(7)	23.8(3)
O24	7326.8(18)	8729.0(10)	3793.0(8)	28.3(3)
C3	3735(2)	6253.6(13)	1165.6(10)	17.4(3)
C17	4702(2)	6468.7(12)	3168.8(10)	15.6(3)
C11	6064(2)	8257.3(13)	3586.8(10)	19.3(3)
C9	3297(2)	8165.7(12)	2910.3(10)	17.1(3)
C14	5380(2)	5941.5(13)	4923.4(10)	19.4(3)
C18	5439(2)	7427.5(13)	1865.2(10)	16.9(3)
C1	3965(2)	7193.3(12)	2481.5(10)	15.1(3)
C16	3378(2)	5804.1(13)	3639.5(10)	19.6(3)
C23	8450(2)	5210.8(14)	4927.7(12)	24.1(4)
C20	7498(2)	6551.5(13)	3920.6(10)	17.6(3)
C5	2108(2)	7594.2(13)	642.1(10)	19.7(3)
C6	1251(2)	7331.4(13)	1480.7(10)	18.0(3)
C7	728(2)	8281.3(14)	1990.8(10)	20.6(3)
C12	5790(2)	7135.0(12)	3809.5(10)	16.7(3)
C8	2258(2)	8873.7(13)	2350.4(10)	19.8(3)
C15	4123(2)	5256.2(13)	4417.2(11)	20.1(3)
C21	7216(2)	5812.4(13)	4631.2(10)	19.2(3)
C13	5024(2)	7071.9(13)	4706.0(10)	19.2(3)
C2	2637(2)	6651.2(13)	1908.1(9)	15.7(3)
C25	-327(2)	6659.5(15)	1300.0(11)	22.3(4)

3.3 NMR Spectra

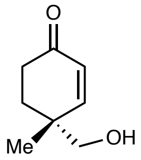

rac-**16**

^{1}H NMR (400 MHz, $CDCl_3$)

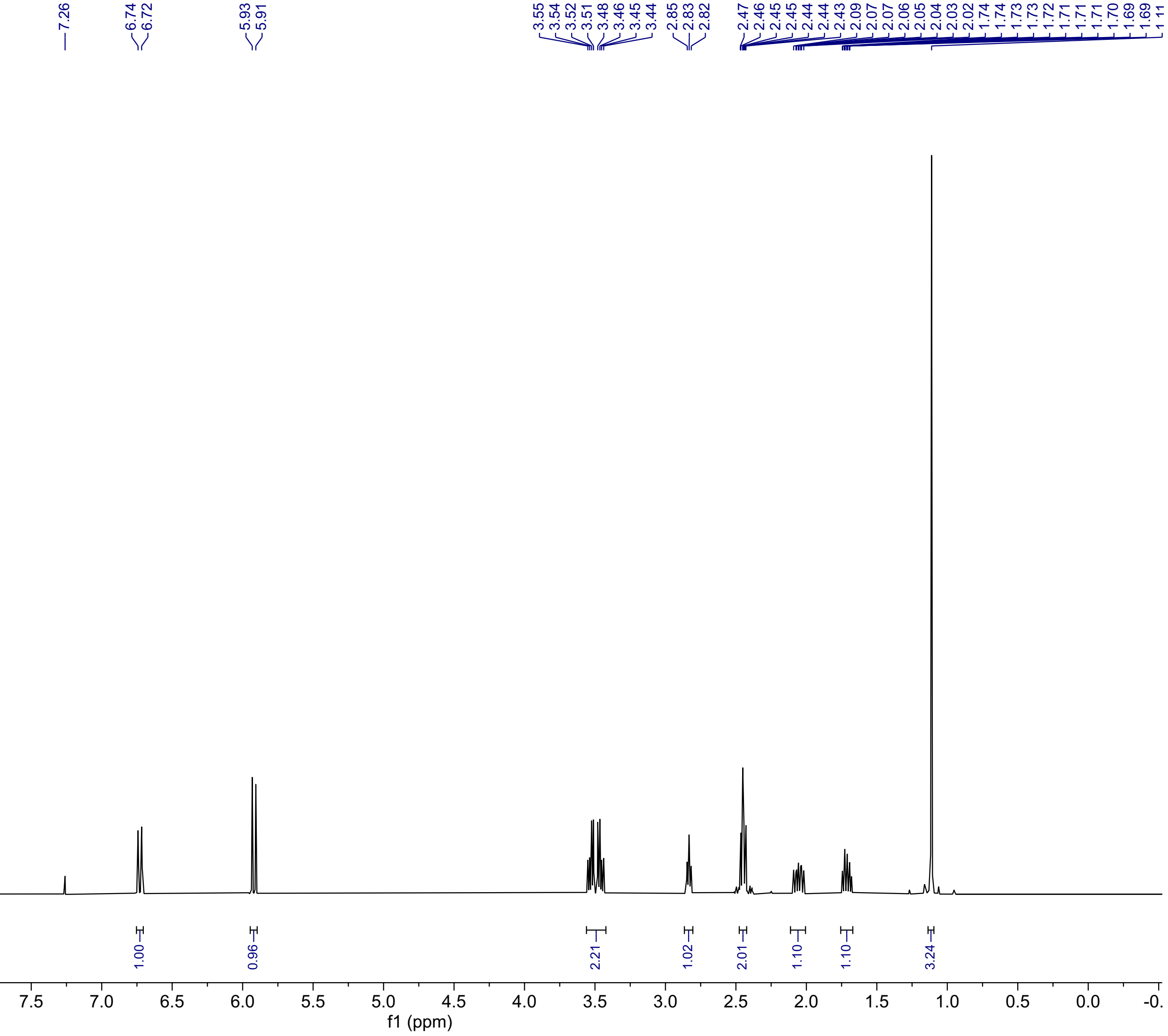

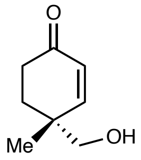

rac-**16**

^{13}C NMR (101 MHz, CDCl$_3$)

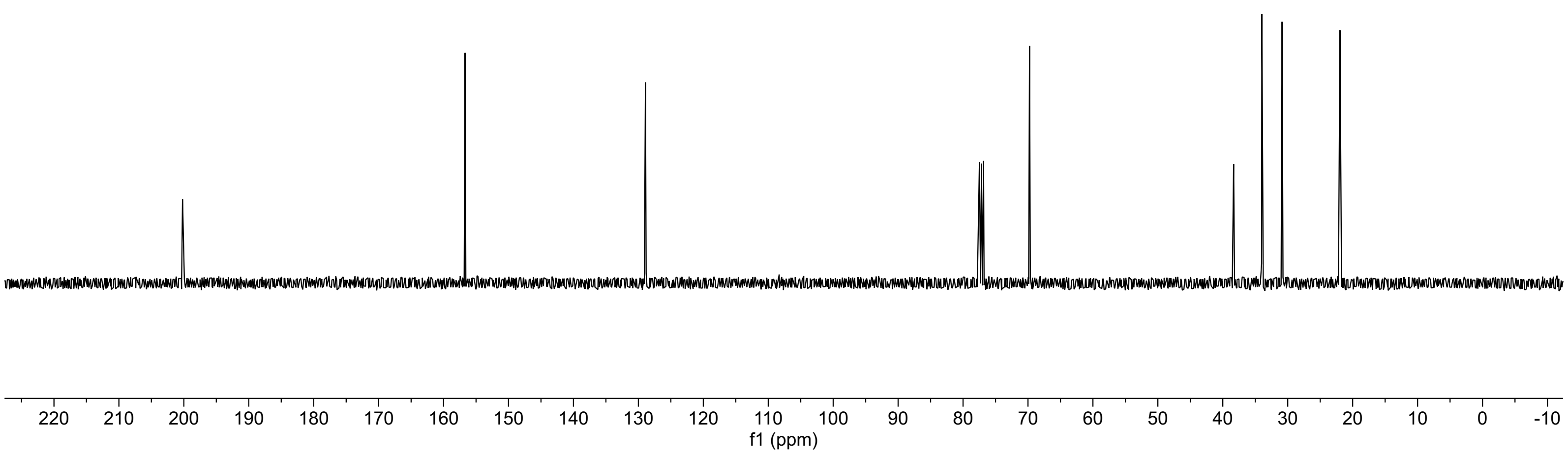

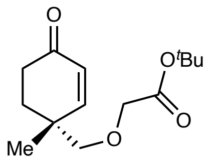

17

^{1}H NMR (400 MHz, CDCl$_3$)

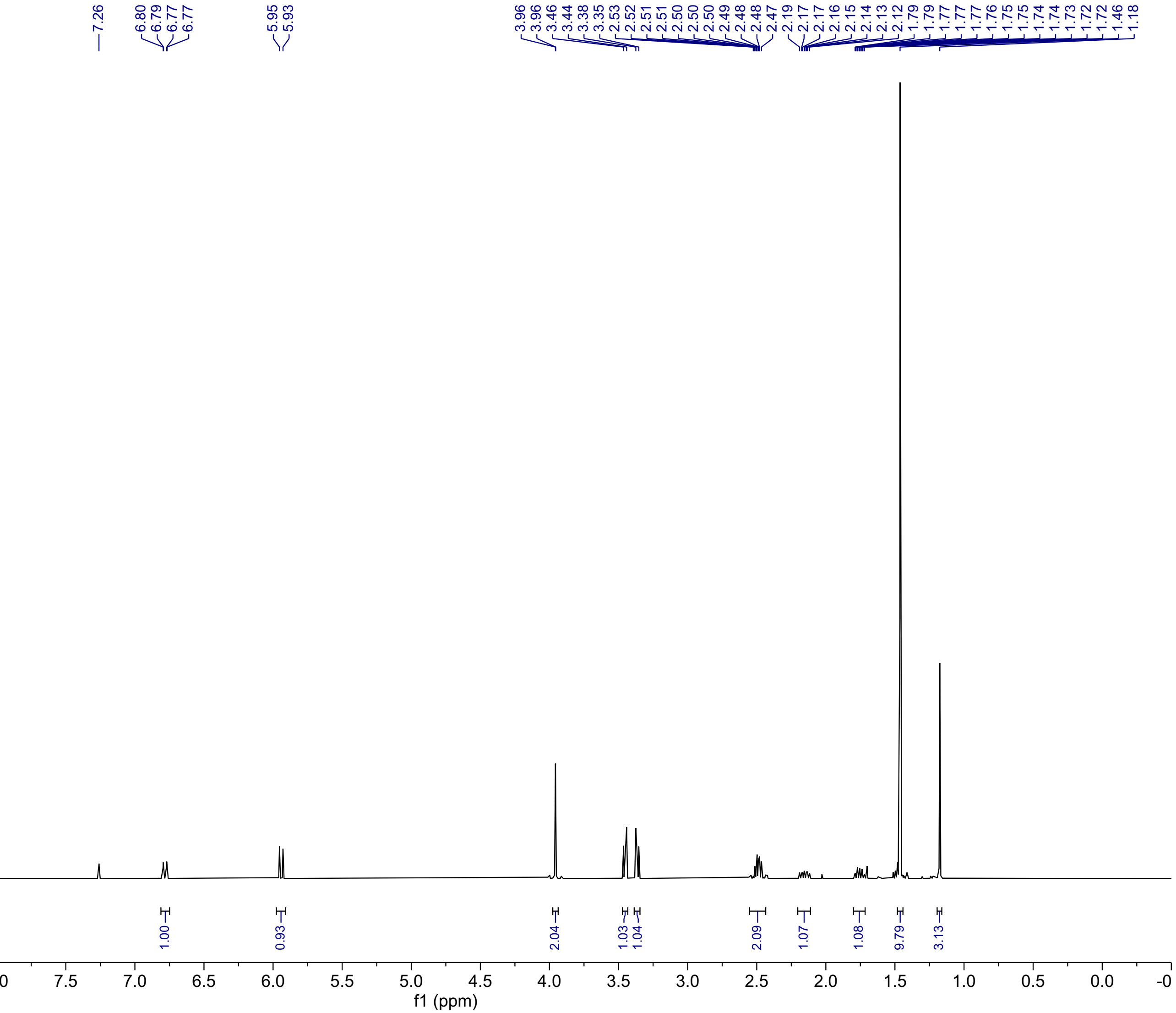

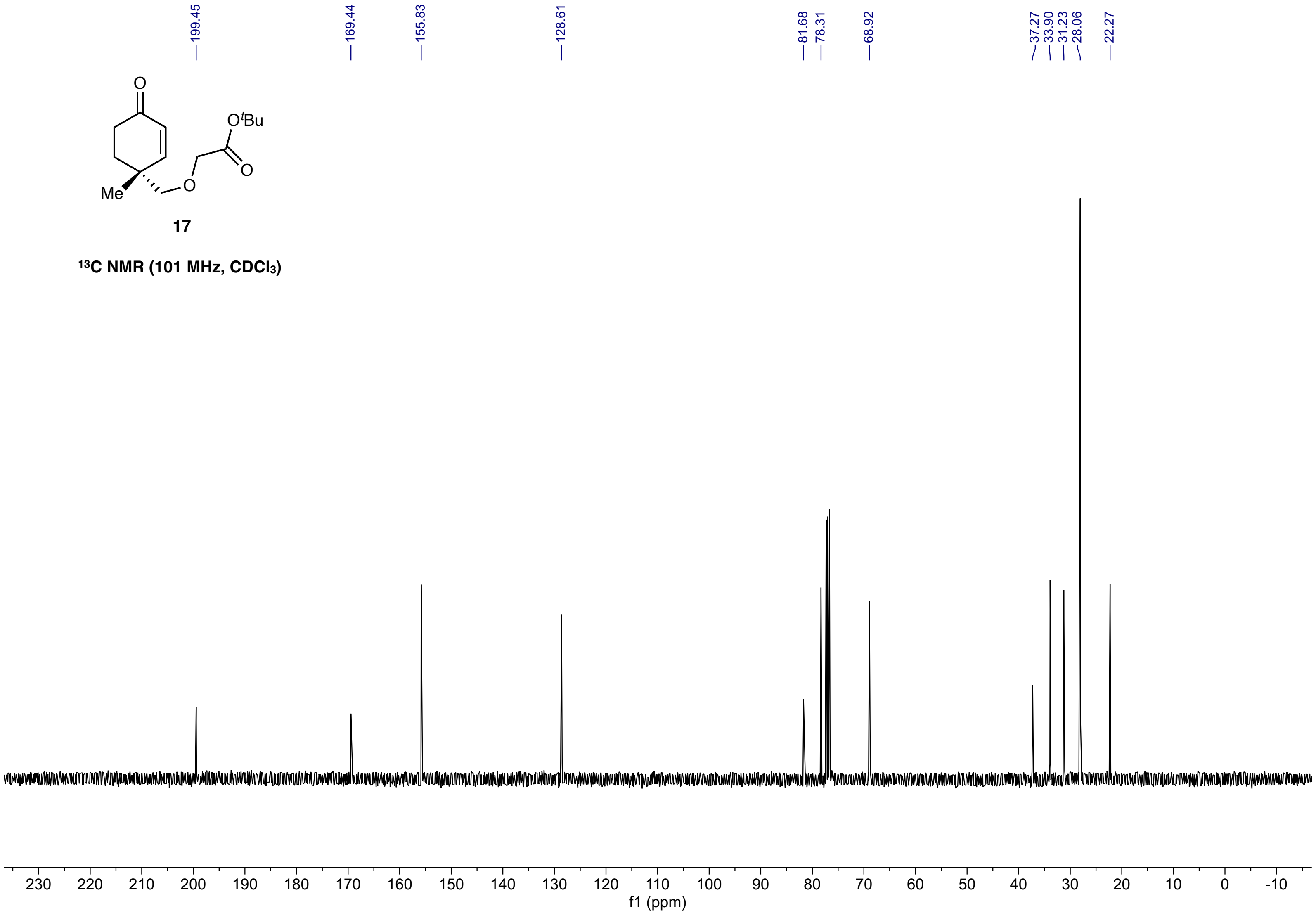
199.45
169.44
155.83
128.61
81.68
78.31
68.92
37.27
33.90
31.23
28.06
22.27
O
O^{t}Bu
O
O
Me
17
^{13}C NMR (101 MHz, $CDCl_3$)
230
220
210
200
190
180
170
160
150
140
130
120
110
100
90
80
70
60
50
40
30
20
10
0
-10
f1 (ppm)

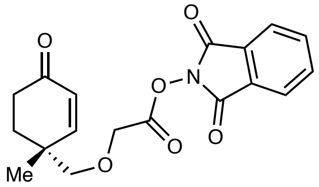

11a

^{1}H NMR (400 MHz, CDCl$_3$)

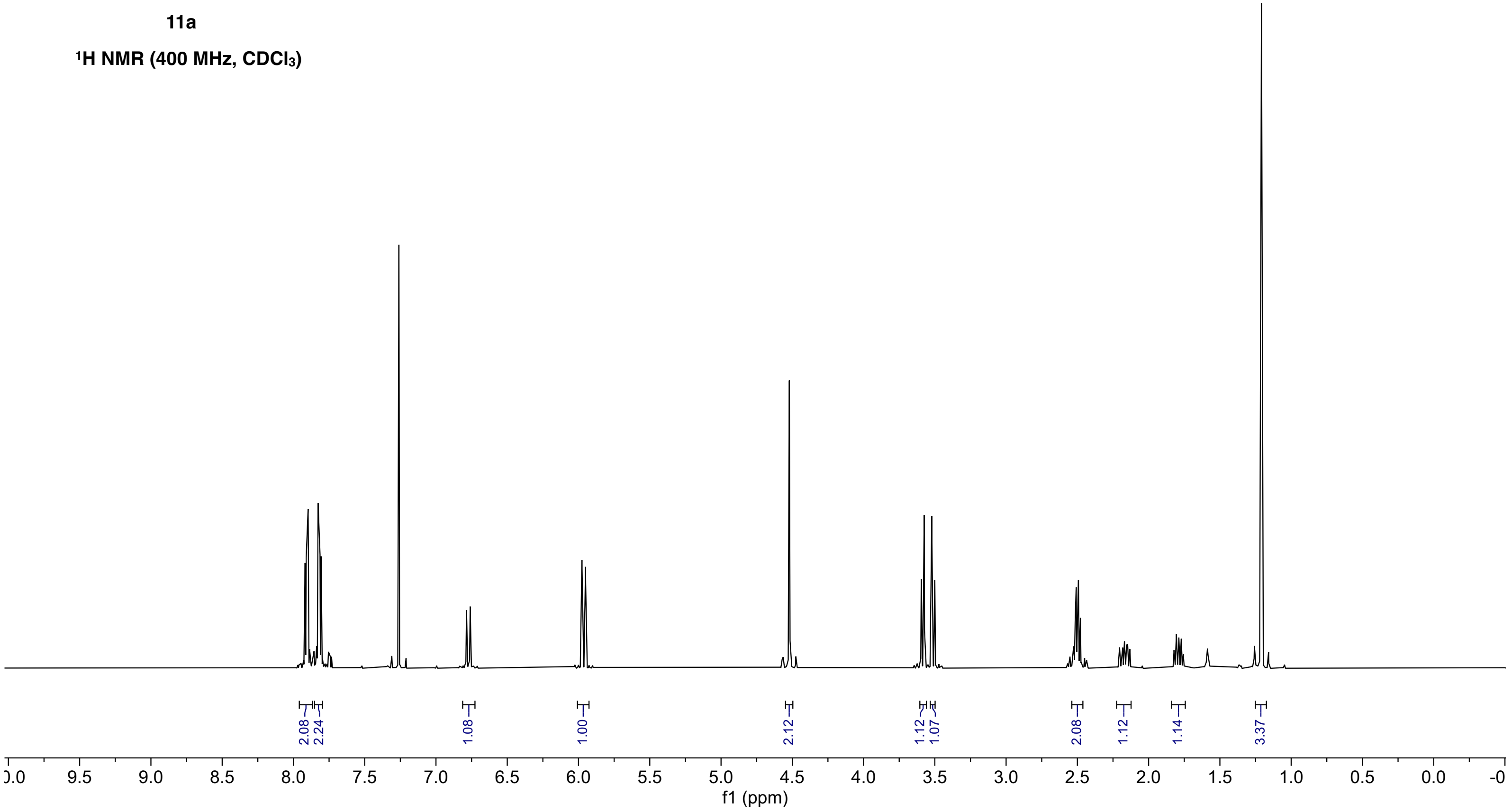

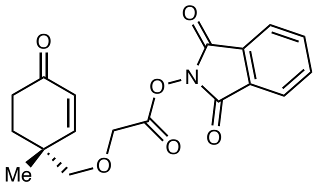

11a

^{13}C NMR (101 MHz, $CDCl_3$)

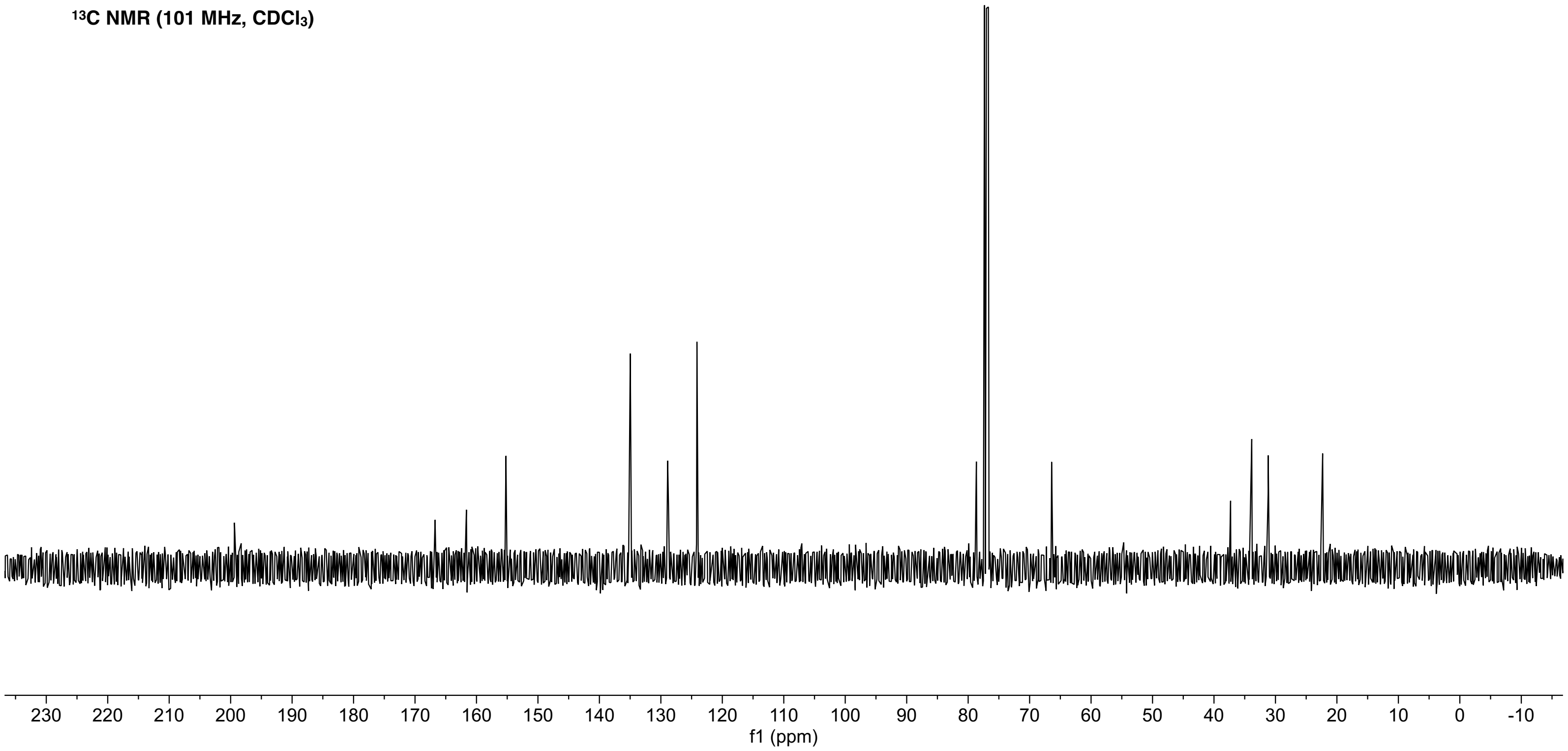

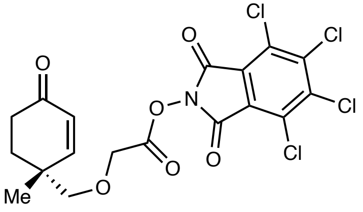

11b

^{1}H NMR (400 MHz, CDCl$_3$)

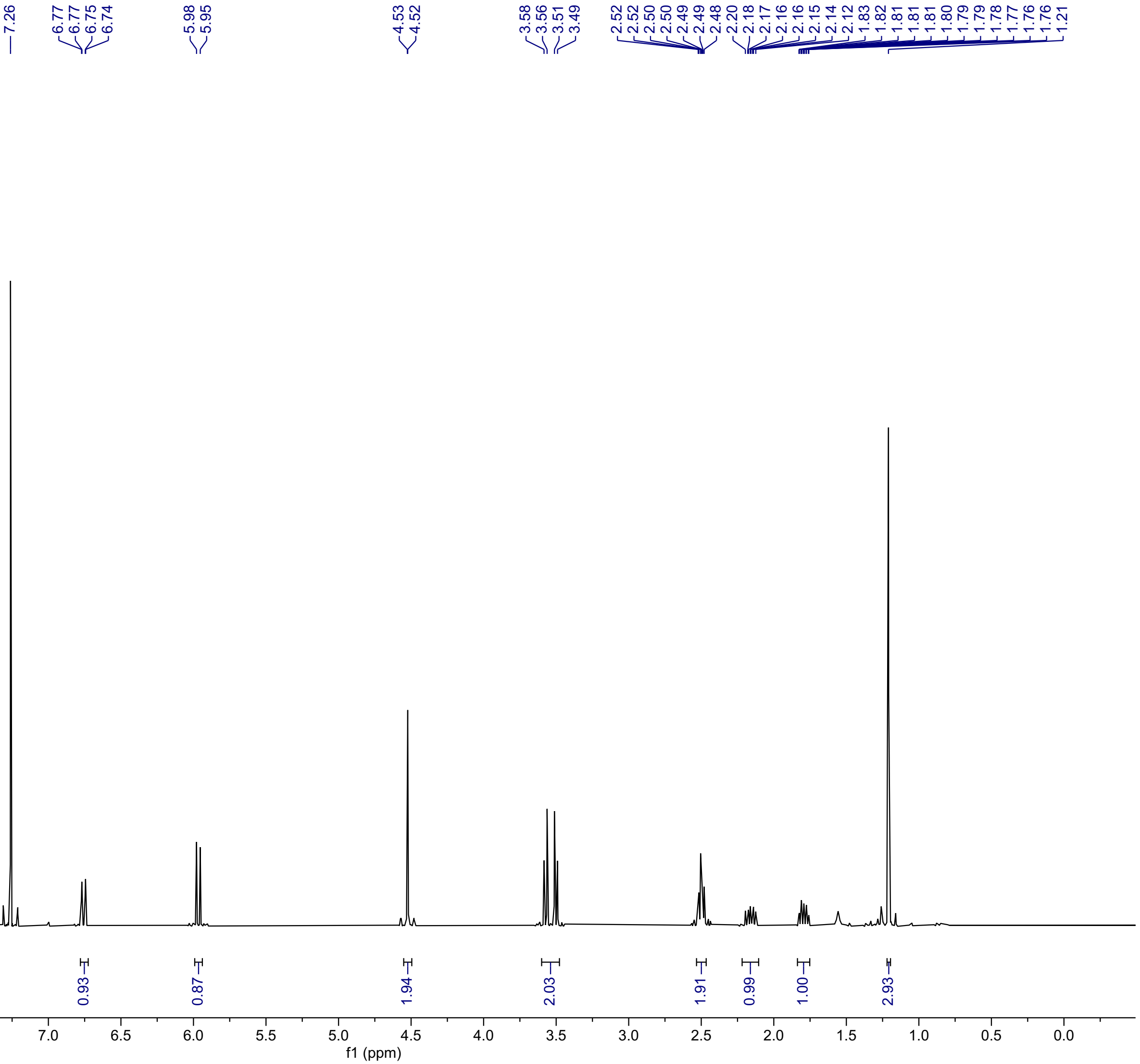

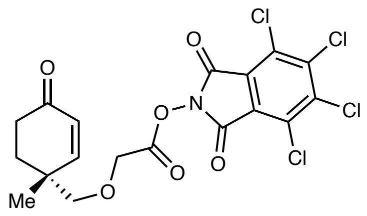

11b

^{13}C NMR (101 MHz, CDCl$_3$)

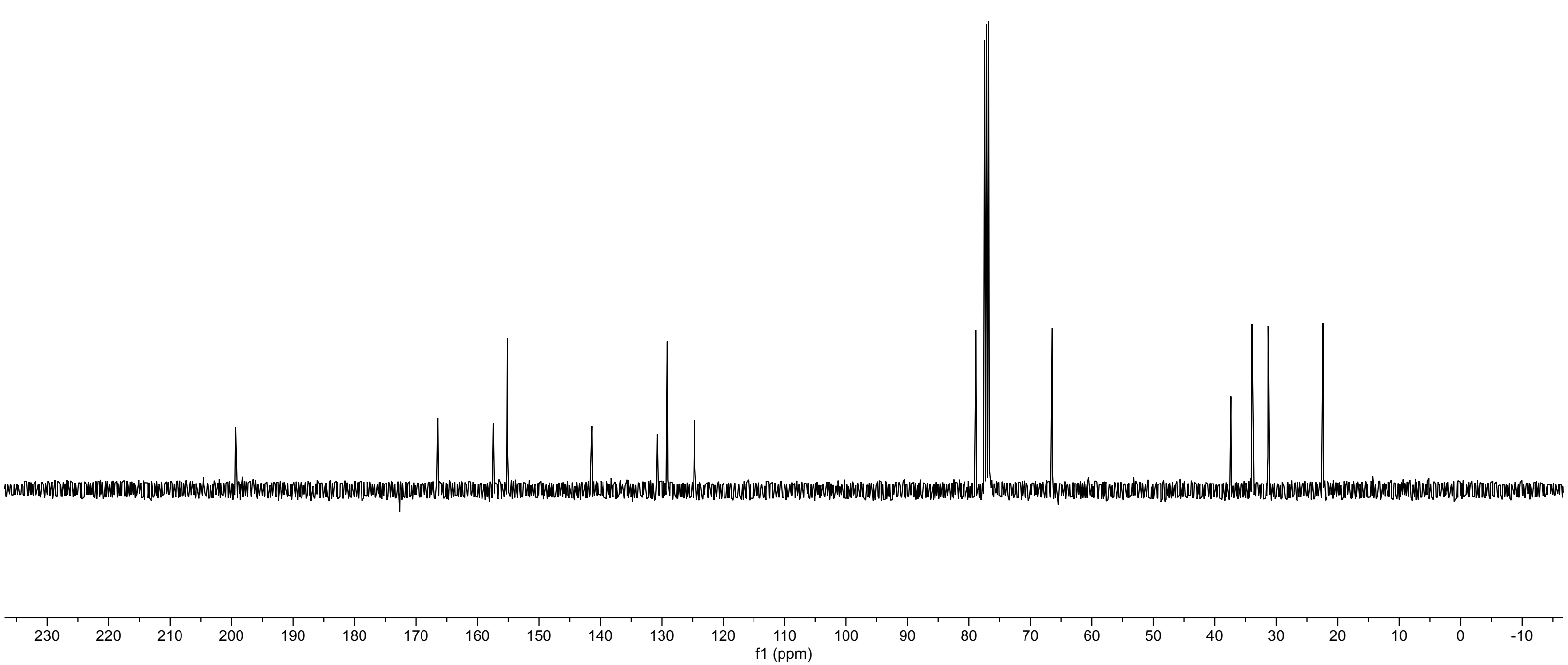

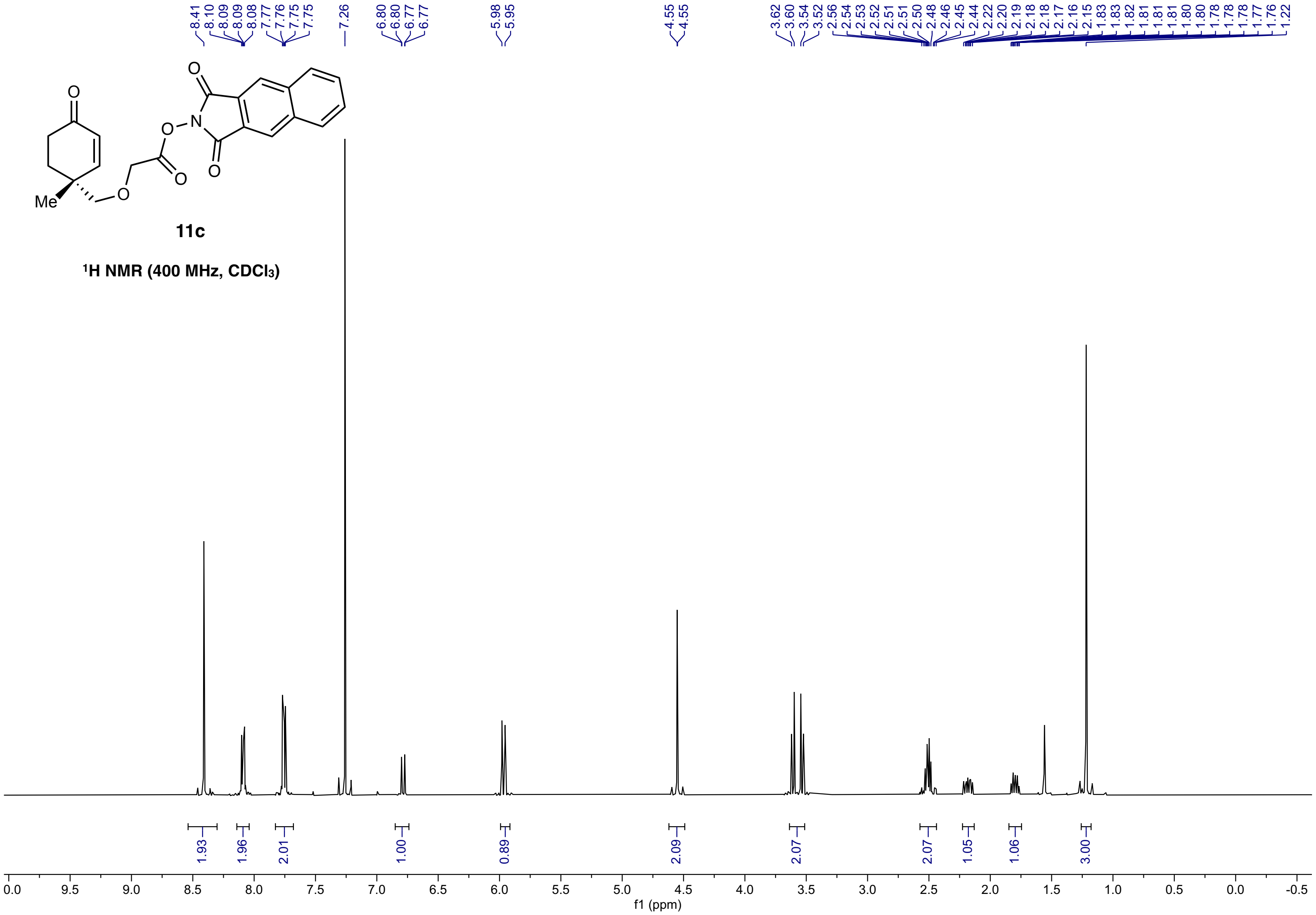

11c

^{1}H NMR (400 MHz, $CDCl_3$)

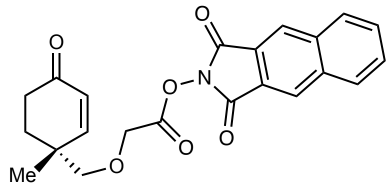

11c

^{13}C NMR (101 MHz, CDCl$_3$)

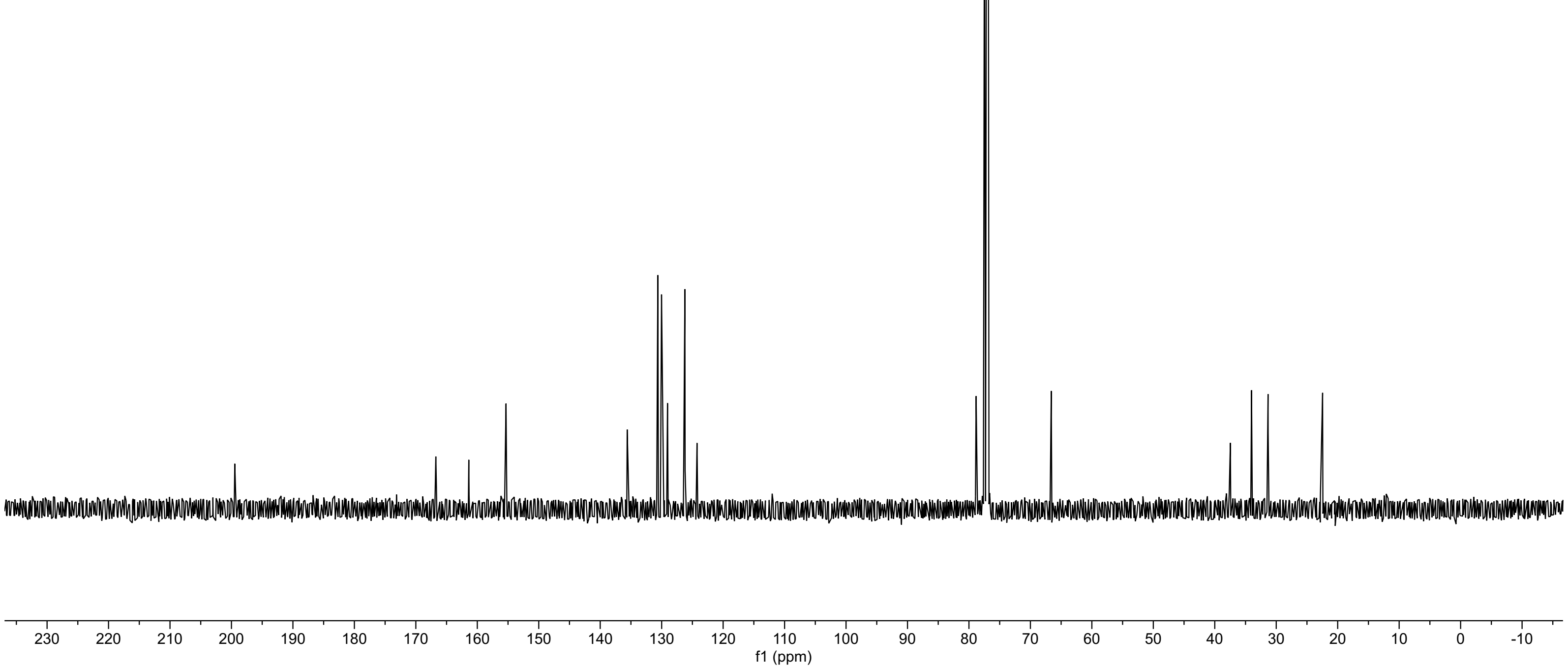

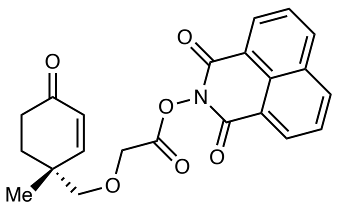

11d

^{1}H NMR (400 MHz, CDCl3)

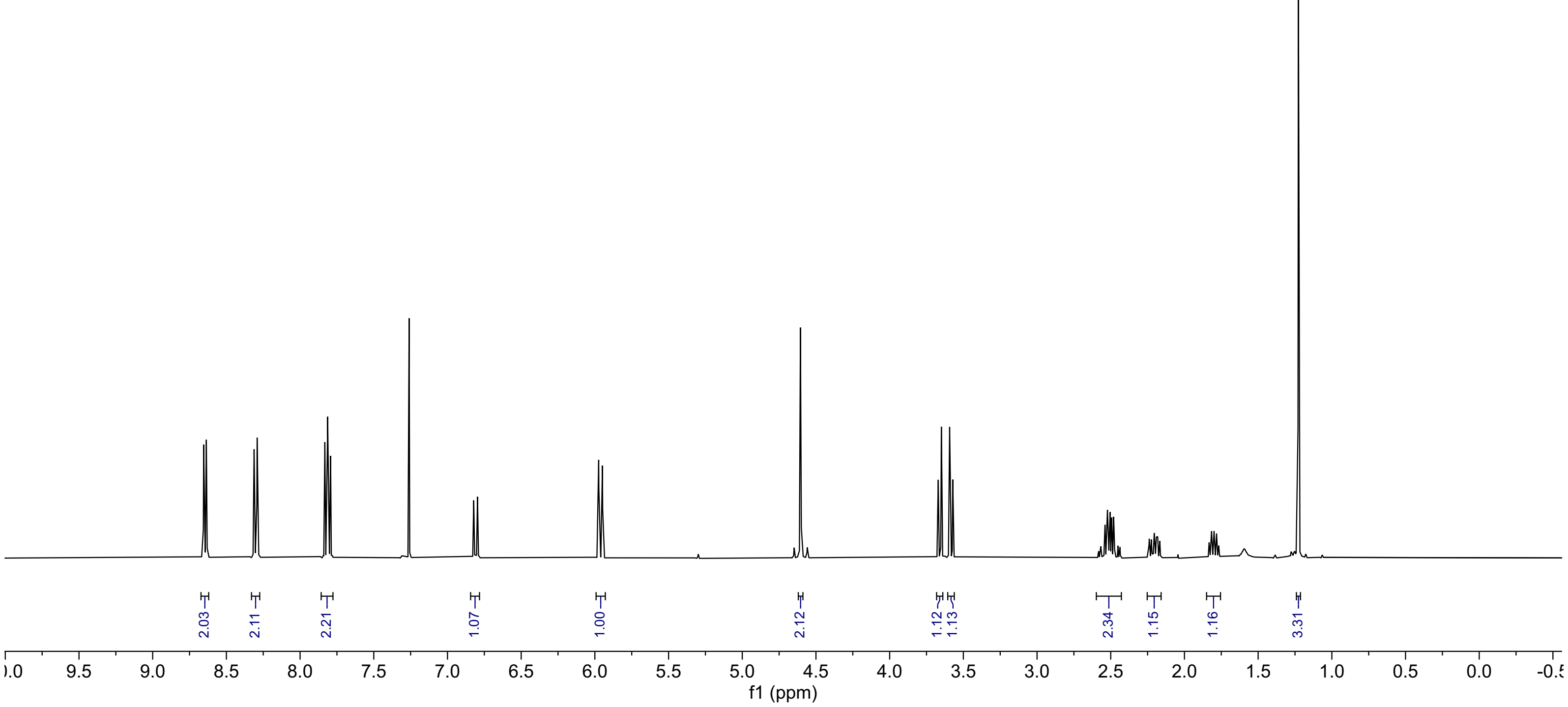

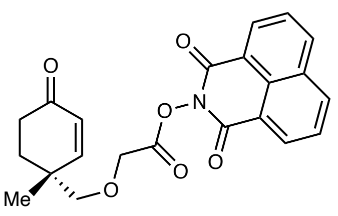

11d

^{13}C NMR (101 MHz, $CDCl_3$)

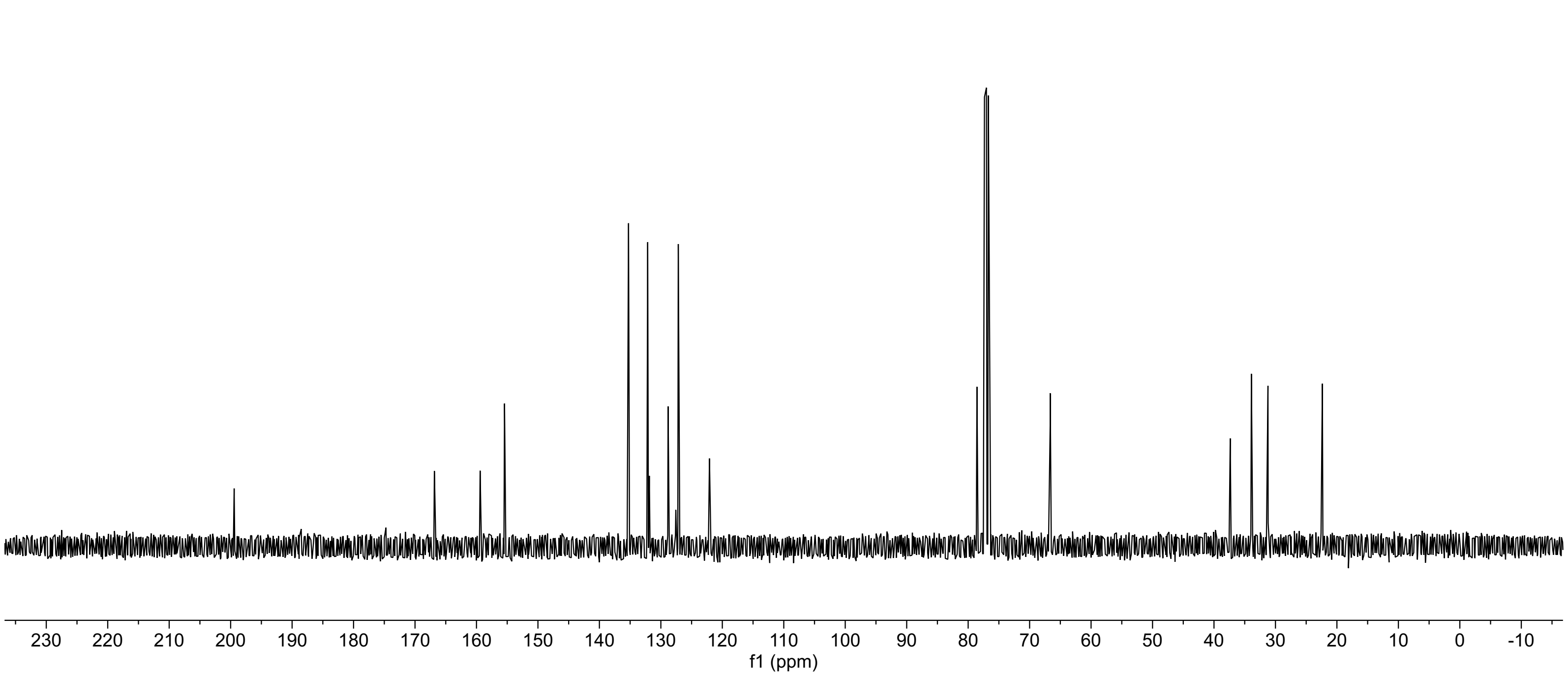

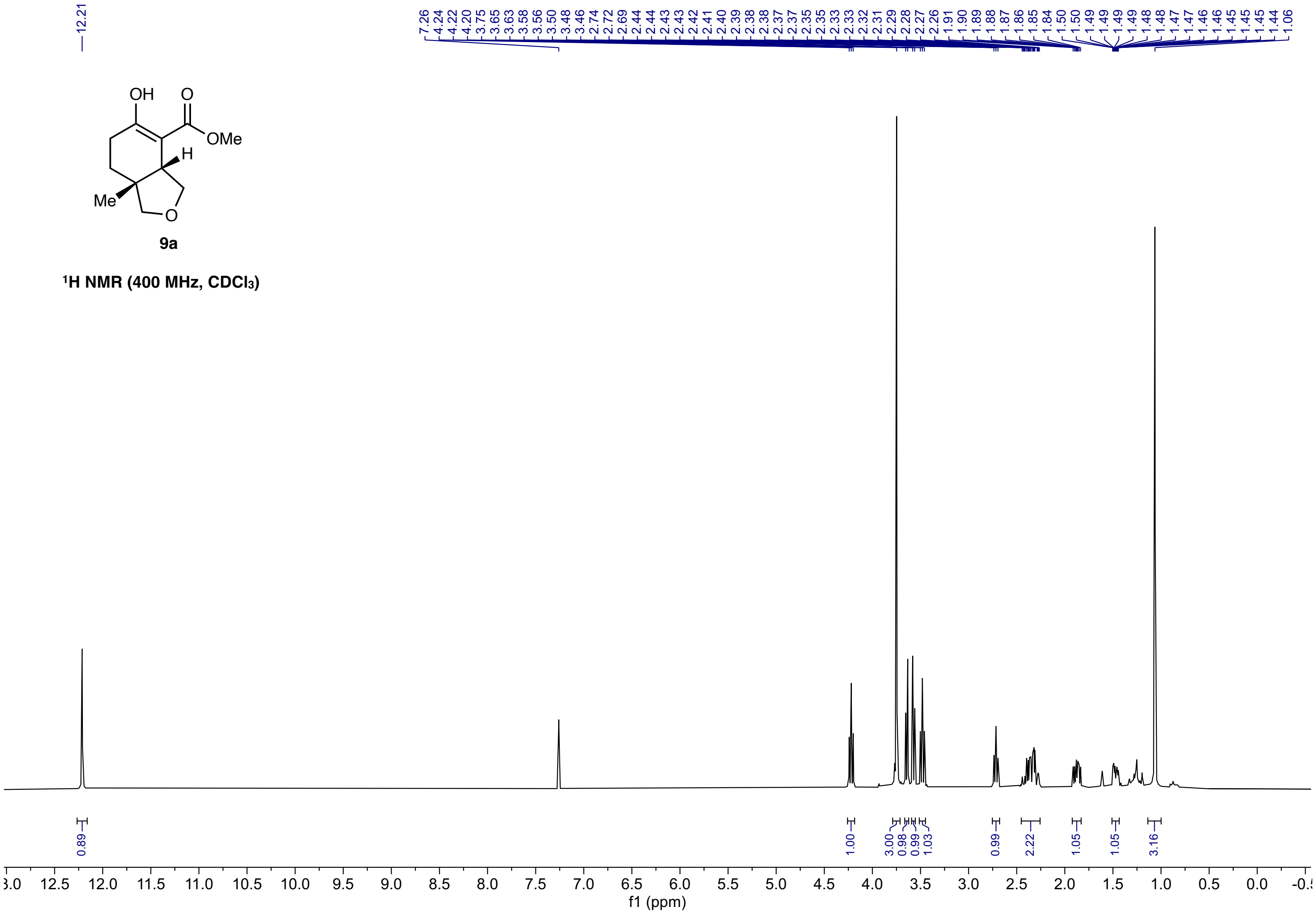
12.21
7.26 4.24 4.22 4.20 3.75 3.65 3.63 3.58 3.56 3.50 3.48 3.46 2.74 2.72 2.69 2.44 2.44 2.43 2.43 2.42 2.41 2.40 2.39 2.38 2.38 2.37 2.37 2.35 2.35 2.33 2.33 2.32 2.31 2.29 2.28 2.27 2.26 1.91 1.90 1.89 1.88 1.87 1.86 1.85 1.84 1.50 1.49 1.49 1.49 1.49 1.48 1.48 1.47 1.46 1.46 1.45 1.45 1.45 1.44 1.06
OH
O
OMe
H
Me
O
9a
¹H NMR (400 MHz, CDCl₃)
0.89
1.00
3.00
0.98
0.99
1.03
0.99
2.22
1.05
1.05
3.16
f1 (ppm)

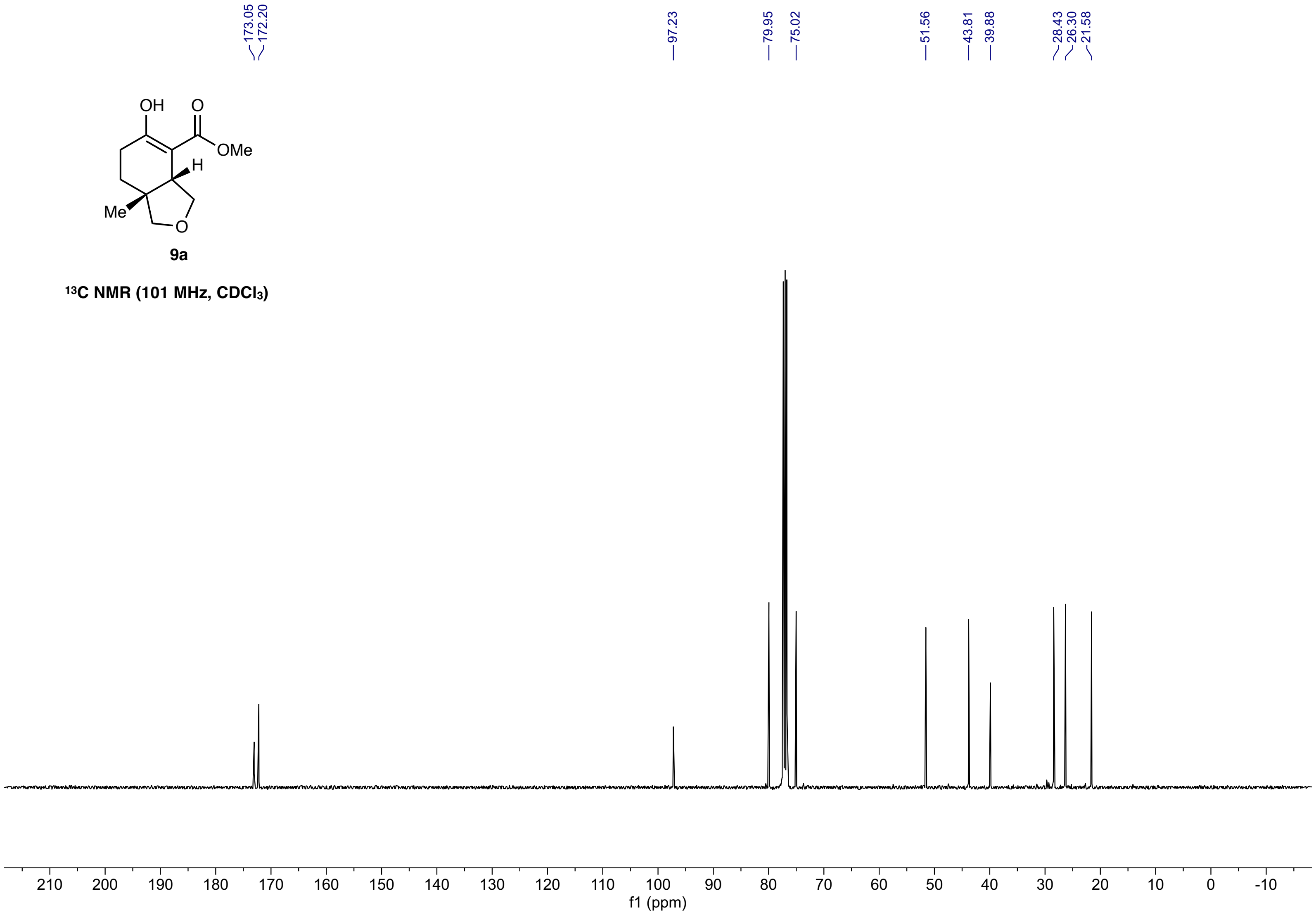
173.05
172.20
97.23
79.95
75.02
51.56
43.81
39.88
28.43
26.30
21.58
OH
O
OMe
H
Me
O
9a
^{13}C NMR (101 MHz, CDCl$_3$)
210 200 190 180 170 160 150 140 130 120 110 100 90 80 70 60 50 40 30 20 10 0 -10
f1 (ppm)

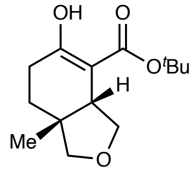

9d

^{1}H NMR (400 MHz, $CDCl_3$)

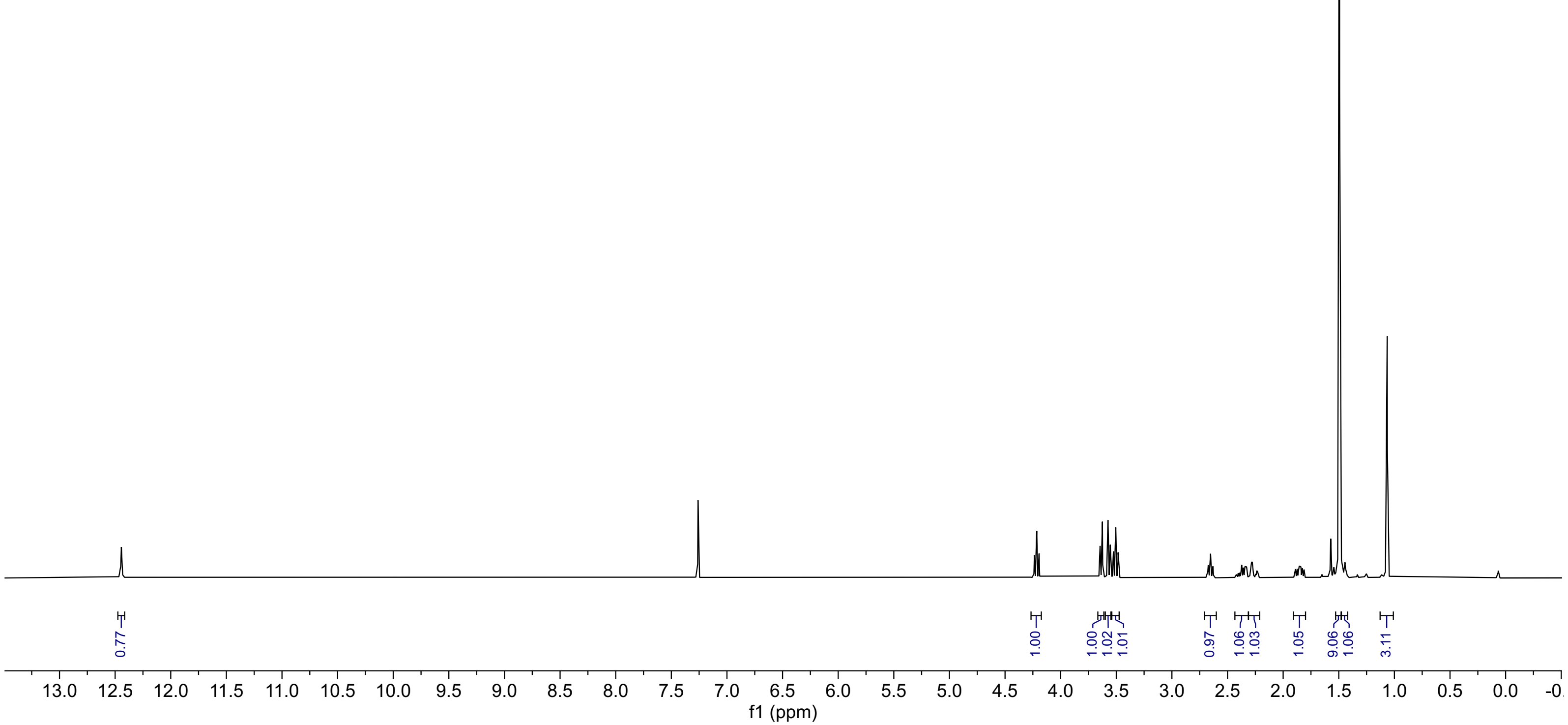

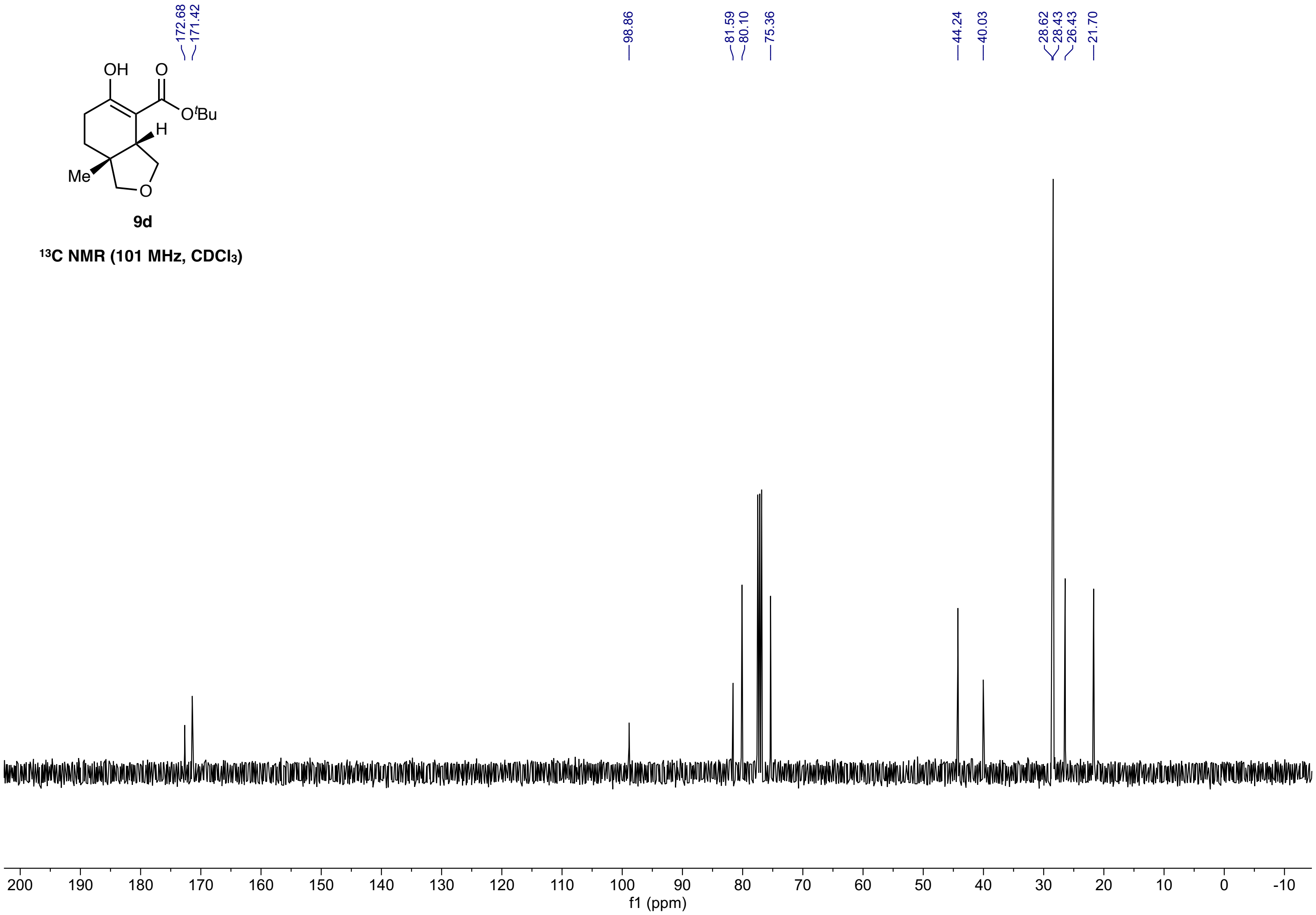

9d

^{13}C NMR (101 MHz, CDCl$_3$)

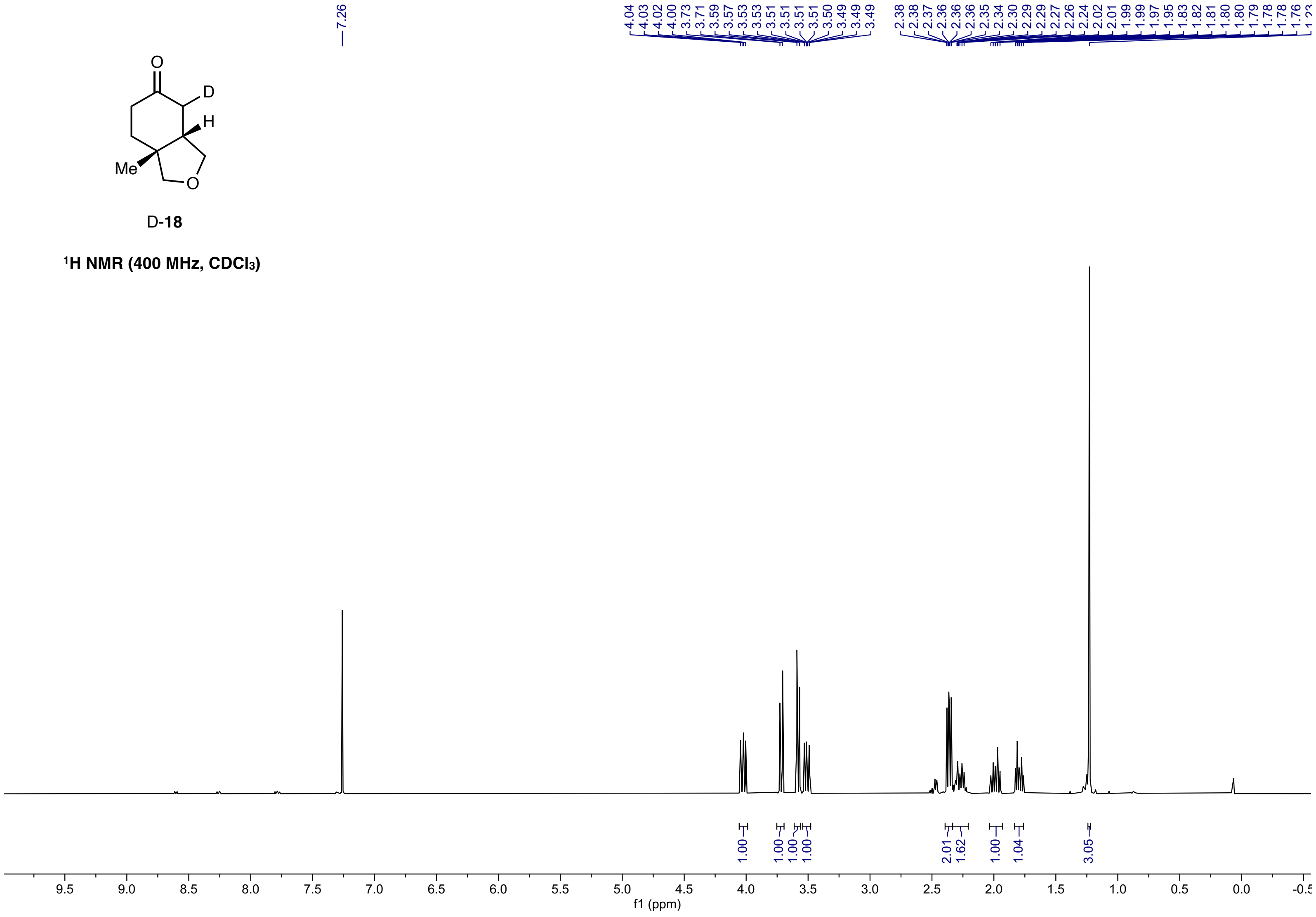

D-18
1H NMR (400 MHz, CDCl3)
7.26
4.04 4.03 4.02 4.00 3.73 3.71 3.59 3.57 3.53 3.53 3.51 3.51 3.51 3.51 3.50 3.49 3.49
2.38 2.38 2.37 2.36 2.36 2.36 2.35 2.34 2.30 2.29 2.29 2.27 2.26 2.24 2.02 2.01 1.99 1.99 1.97 1.95 1.83 1.82 1.81 1.80 1.79 1.78 1.76
1.00 1.00 1.00 1.00 2.01 1.62 1.00 1.04 3.05
f1 (ppm)

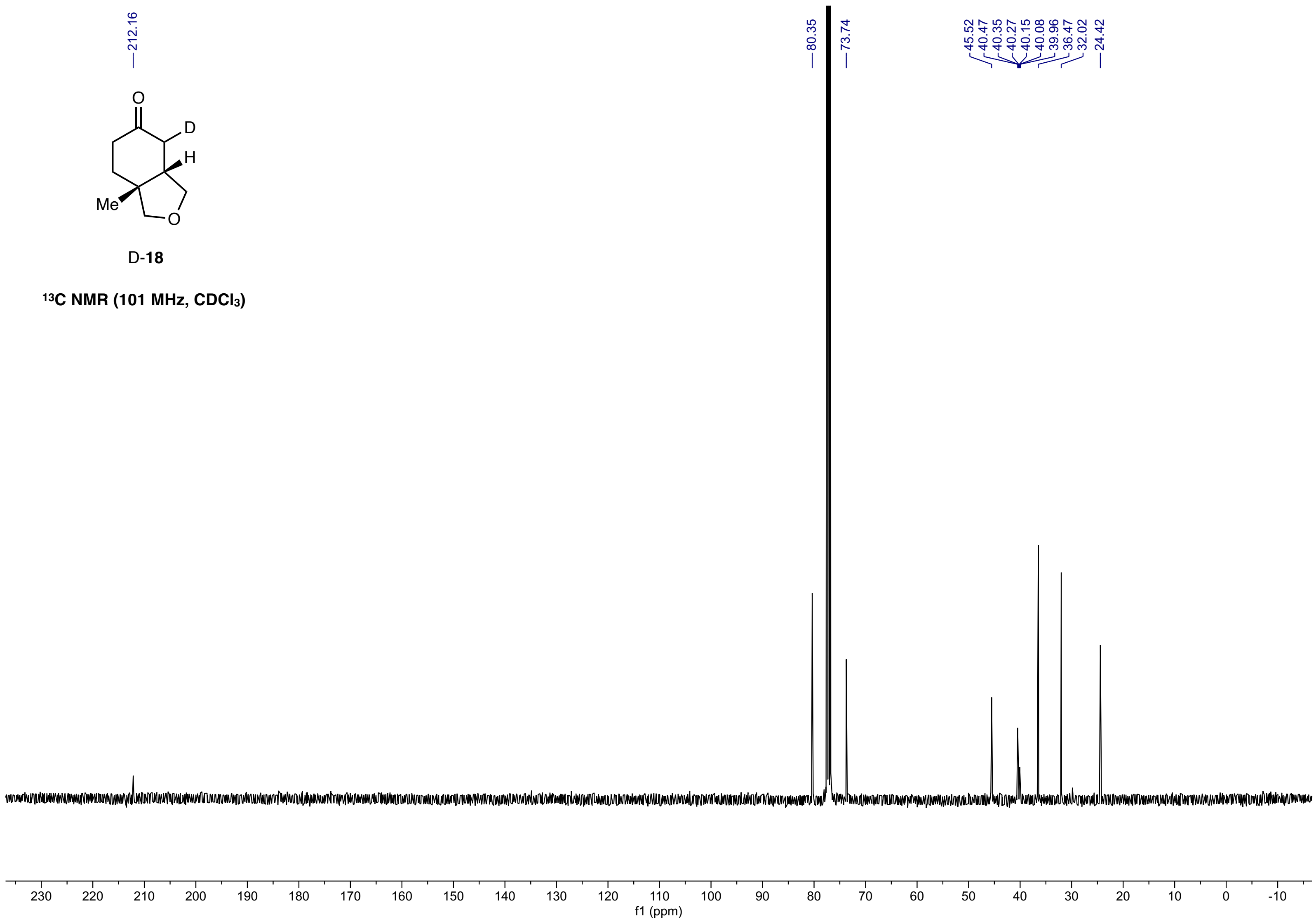

212.16
80.35
73.74
45.52
40.47
40.35
40.27
40.15
40.08
39.96
36.47
32.02
24.42
O
D
H
Me
O
D-**18**
¹³C NMR (101 MHz, CDCl₃)
230
220
210
200
190
180
170
160
150
140
130
120
110
100
90
80
70
60
50
40
30
20
10
0
-10
f1 (ppm)

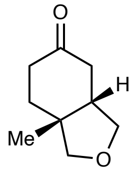

18

^{1}H NMR (400 MHz, $CDCl_3$)

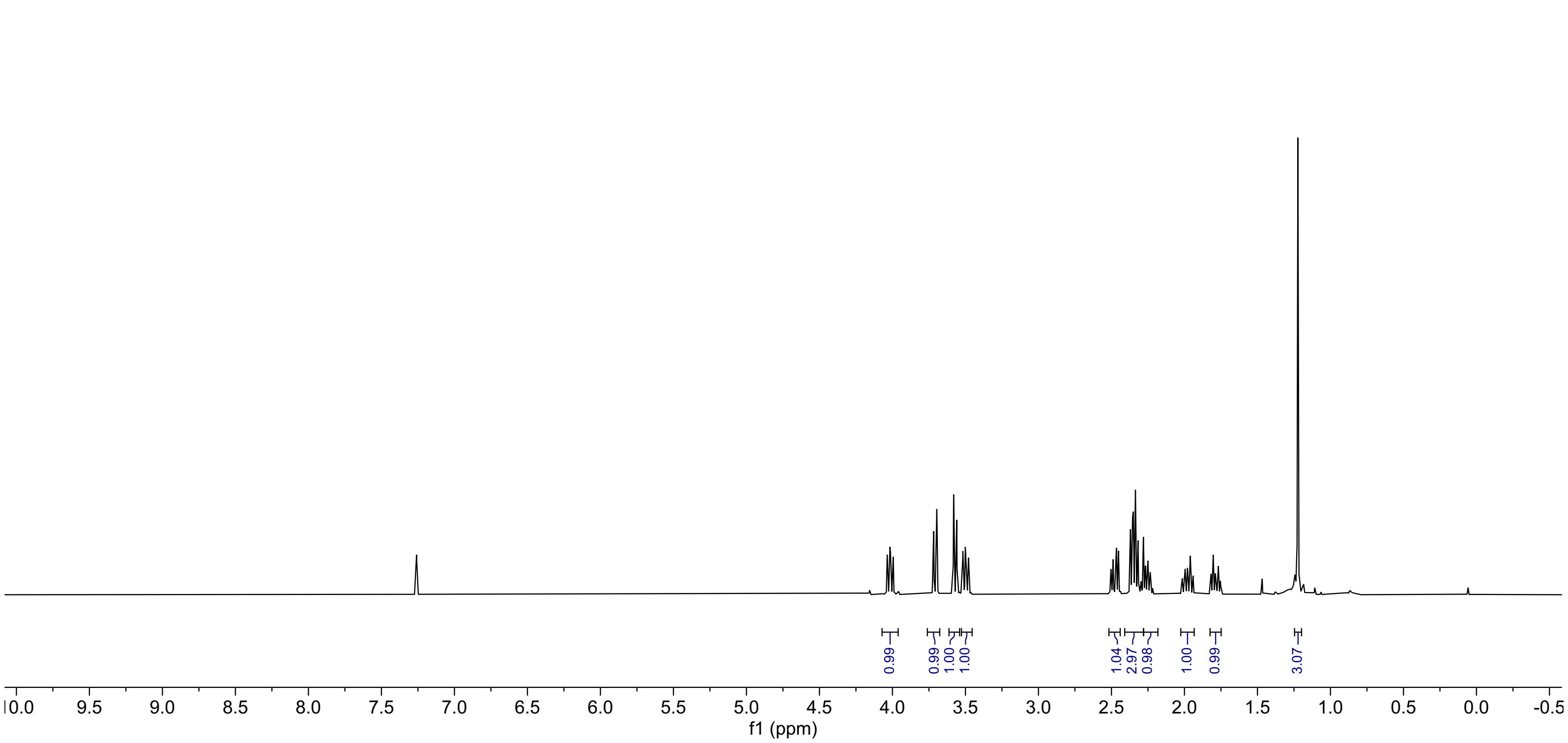

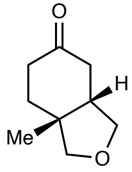

18

^{13}C NMR (101 MHz, $CDCl_3$)

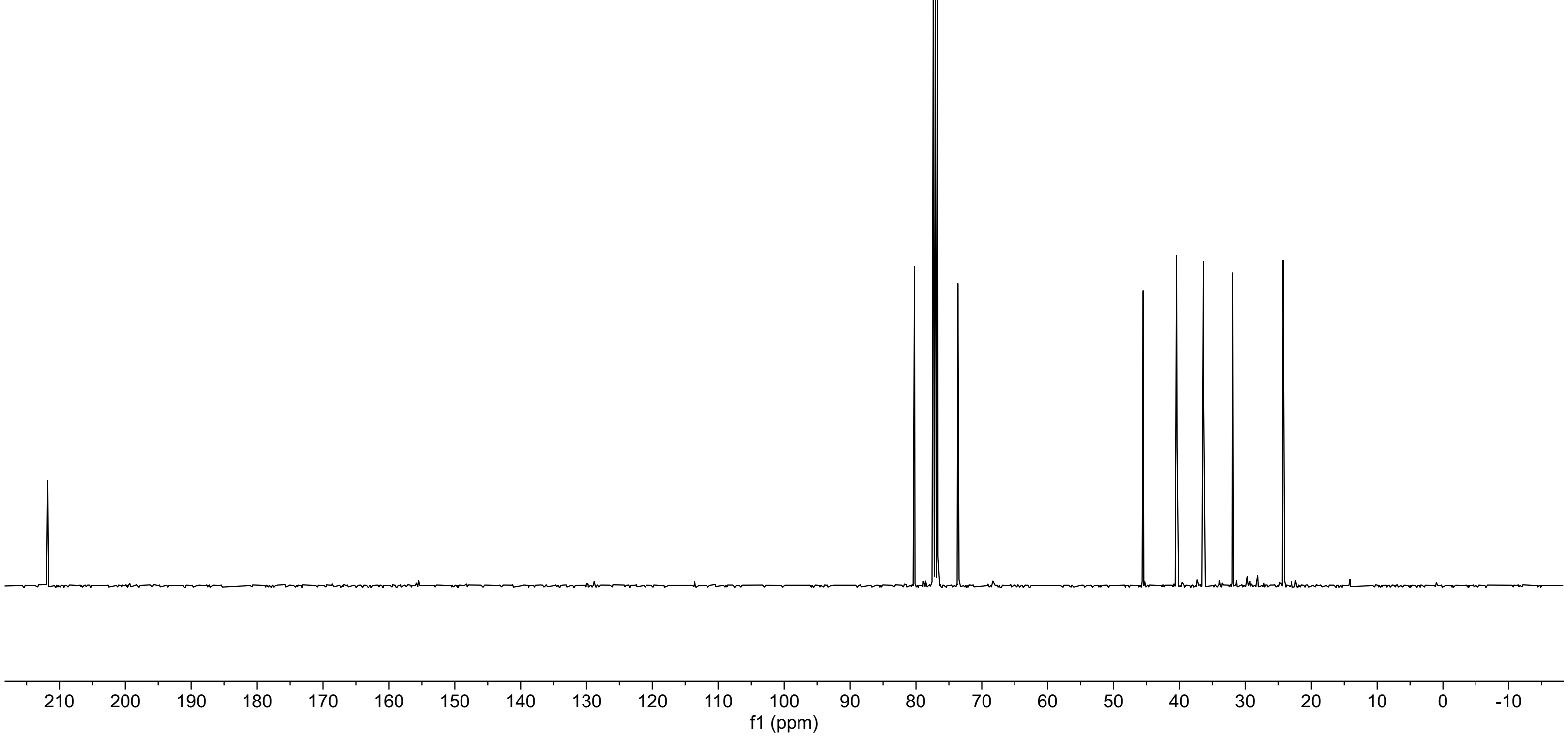

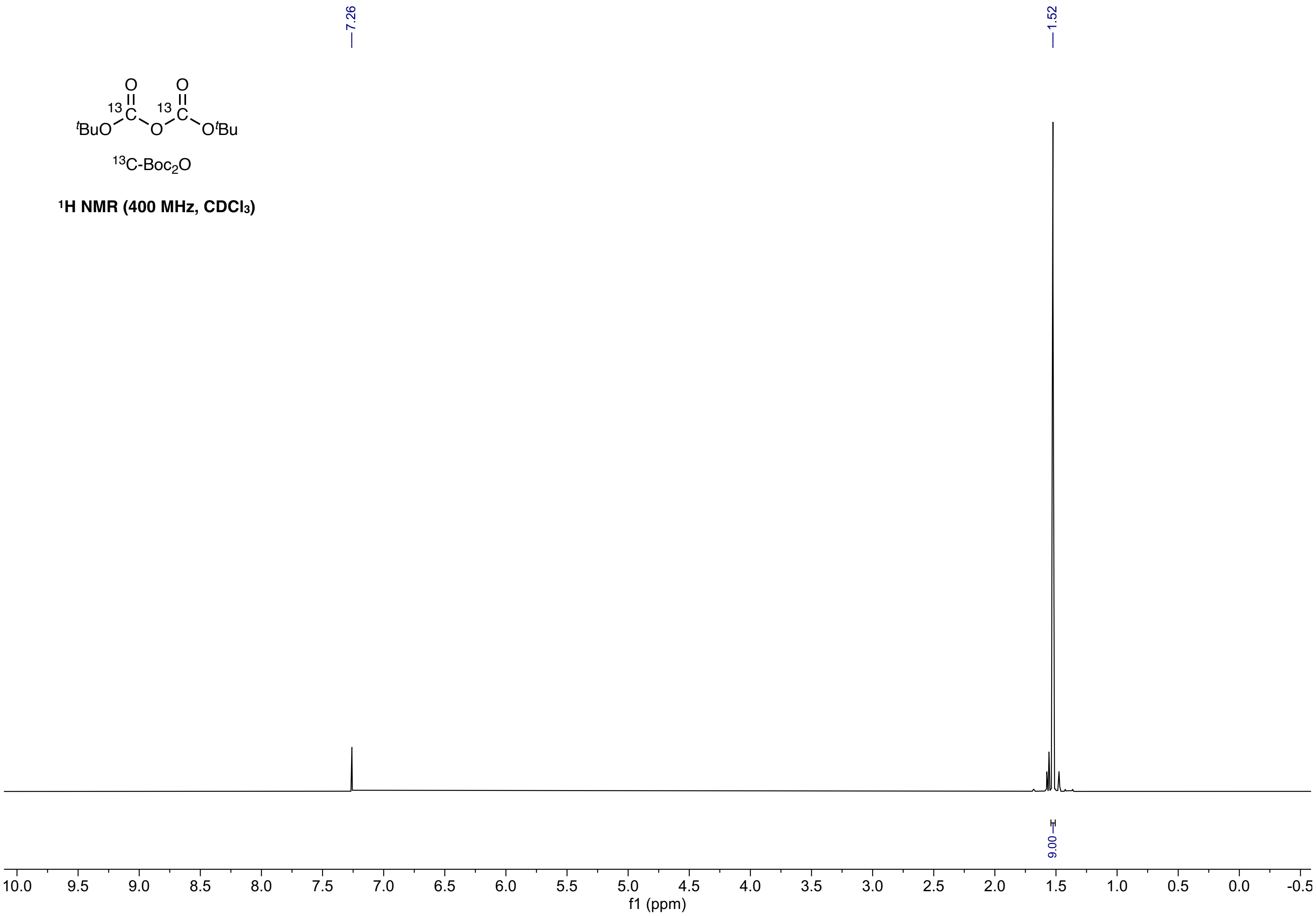
7.26
1.52
^{13}C-Boc$_2$O
^{1}H NMR (400 MHz, CDCl$_3$)
9.00
10.0 9.5 9.0 8.5 8.0 7.5 7.0 6.5 6.0 5.5 5.0 4.5 4.0 3.5 3.0 2.5 2.0 1.5 1.0 0.5 0.0 -0.5
f1 (ppm)

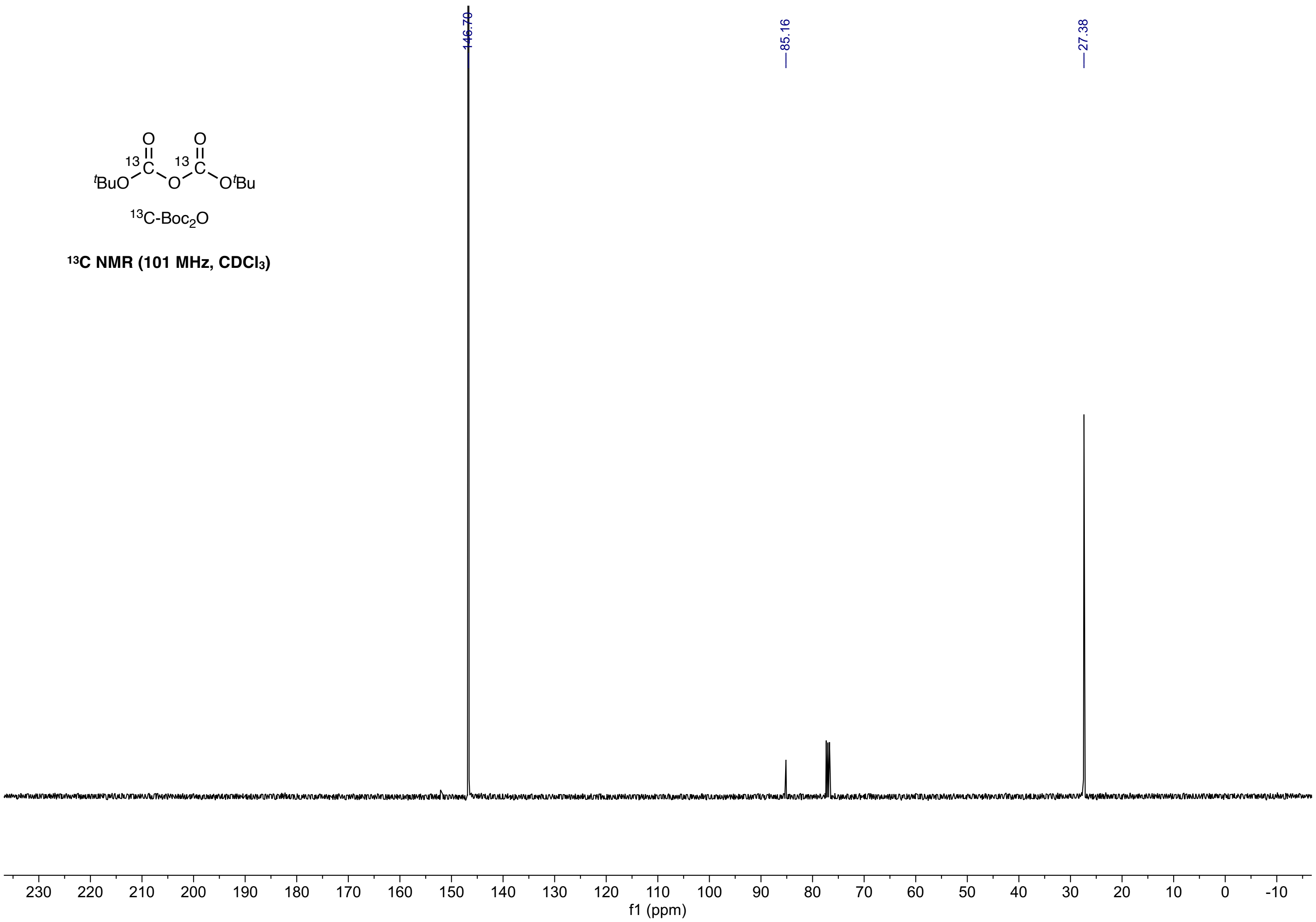
146.70
85.16
27.38
^{t}BuO
O
O^{t}Bu
13
^{13}C-Boc$_2$O
^{13}C NMR (101 MHz, CDCl$_3$)
230 220 210 200 190 180 170 160 150 140 130 120 110 100 90 80 70 60 50 40 30 20 10 0 -10
f1 (ppm)

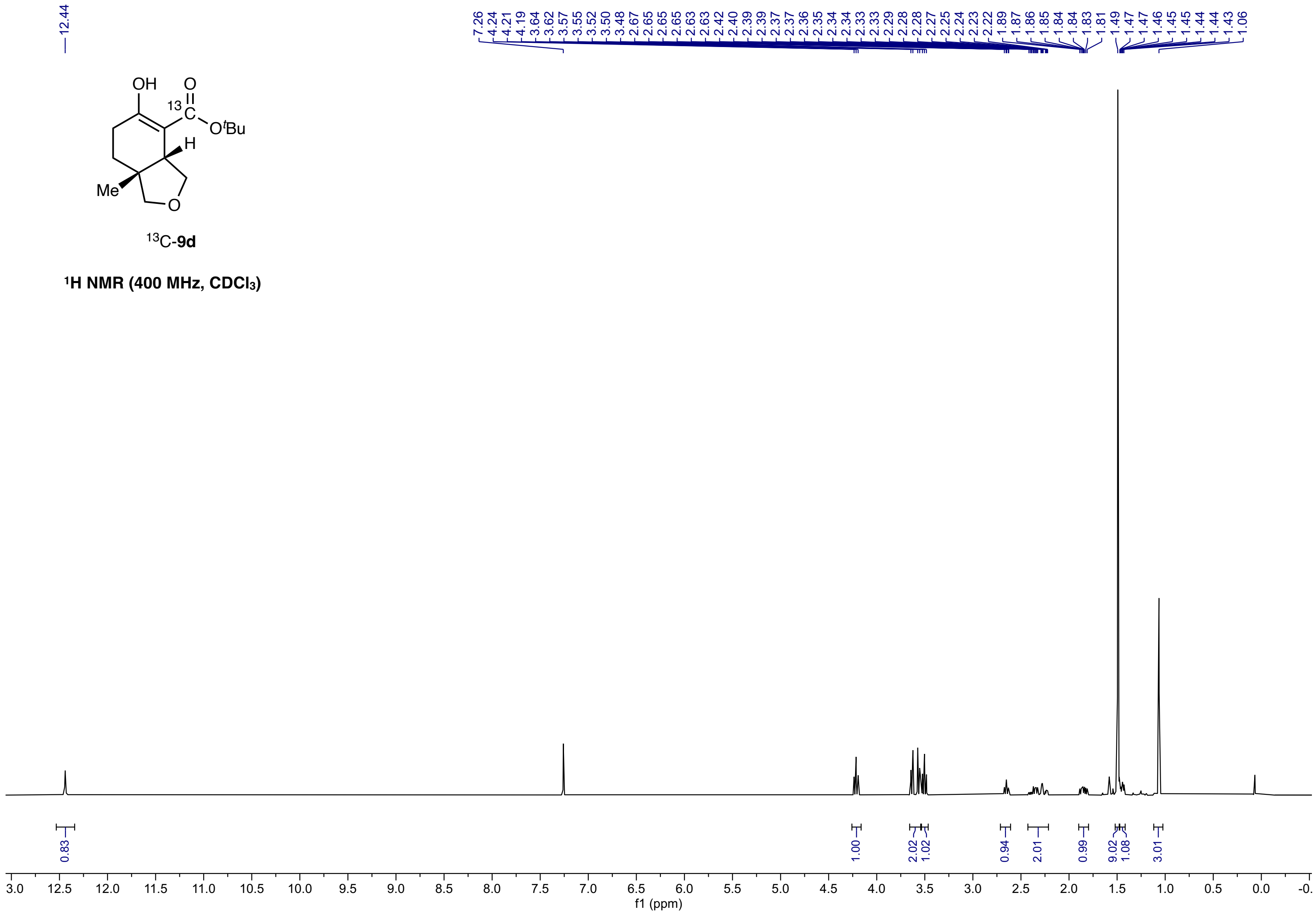
12.44
7.26 4.24 4.21 4.19 3.64 3.62 3.57 3.55 3.52 3.50 3.48 2.67 2.65 2.65 2.63 2.63 2.42 2.40 2.39 2.37 2.37 2.36 2.35 2.34 2.34 2.33 2.33 2.29 2.28 2.28 2.27 2.25 2.24 2.23 2.22 1.89 1.87 1.86 1.85 1.84 1.84 1.83 1.81 1.49 1.47 1.47 1.46 1.45 1.45 1.44 1.44 1.43 1.06
OH
O
13
C
O^tBu
H
Me
O
^13C-**9d**
¹H NMR (400 MHz, CDCl₃)
0.83
1.00
2.02
1.02
0.94
2.01
0.99
9.02
1.08
3.01
f1 (ppm)

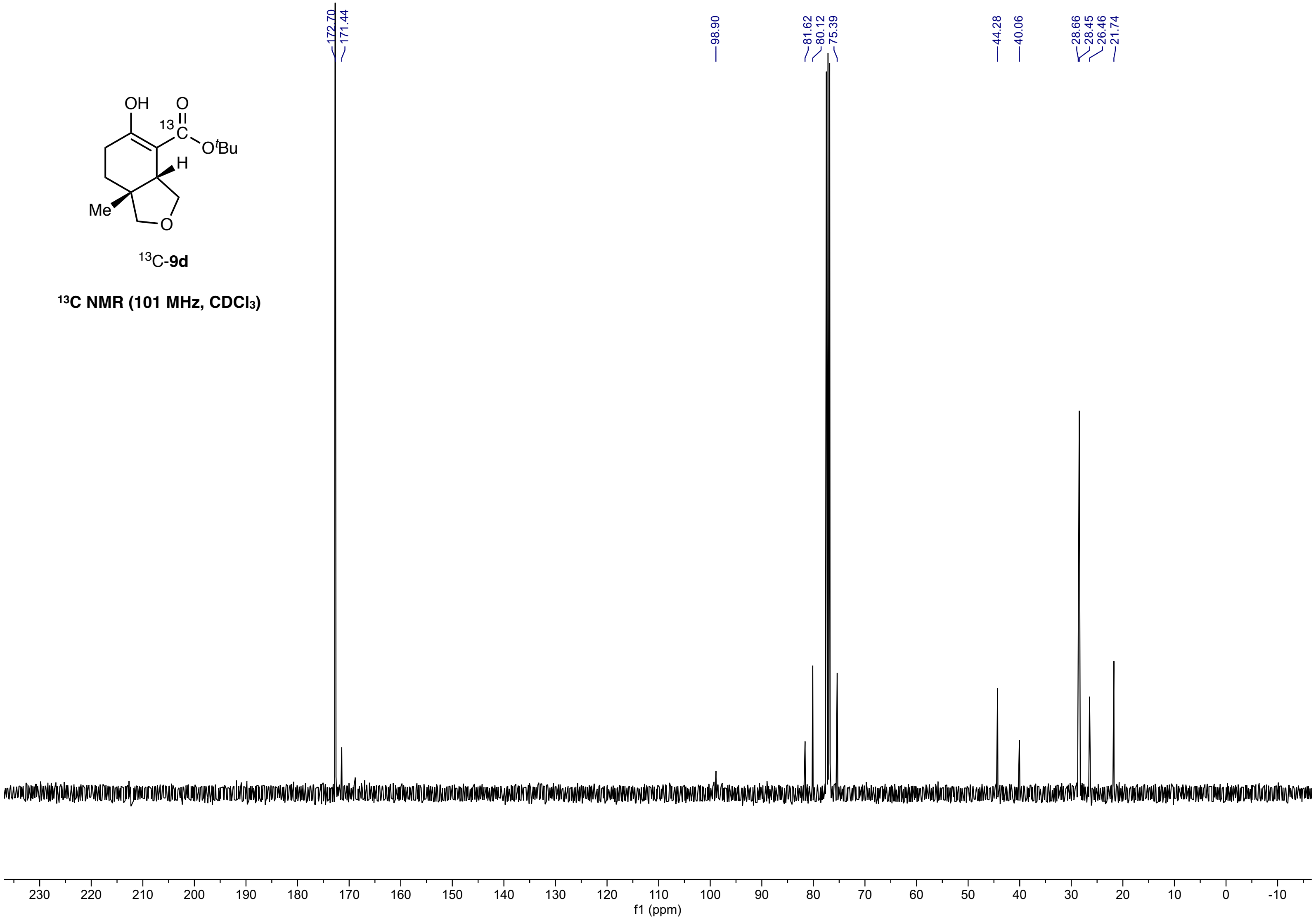

^{13}C-**9d**

^{13}C NMR (101 MHz, $CDCl_3$)

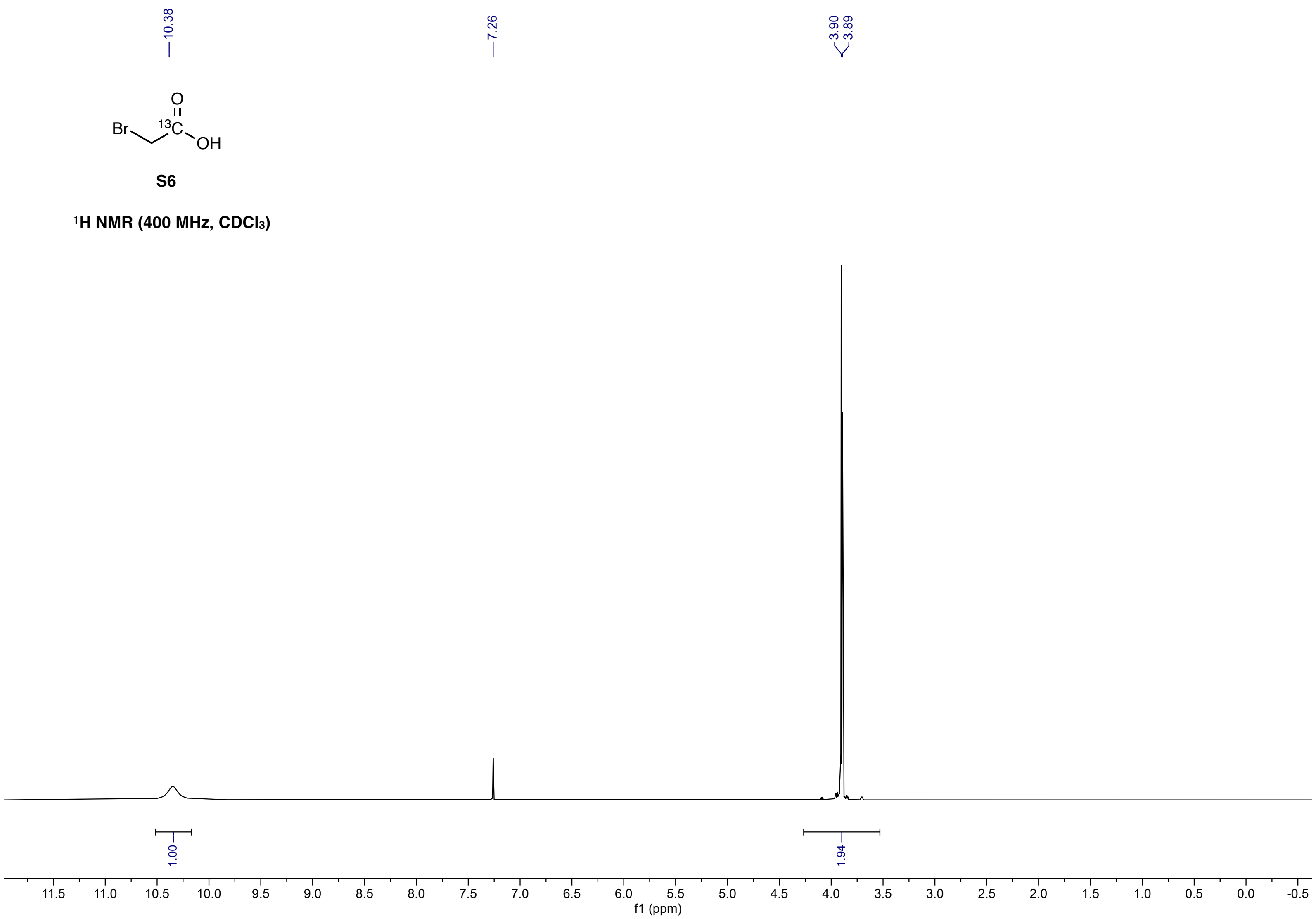
10.38
7.26
3.90
3.89
Br
13C
O
OH
S6
1H NMR (400 MHz, CDCl3)
1.00
1.94
11.5 11.0 10.5 10.0 9.5 9.0 8.5 8.0 7.5 7.0 6.5 6.0 5.5 5.0 4.5 4.0 3.5 3.0 2.5 2.0 1.5 1.0 0.5 0.0 -0.5
f1 (ppm)

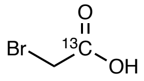

S6

^{13}C NMR (101 MHz, $CDCl_3$)

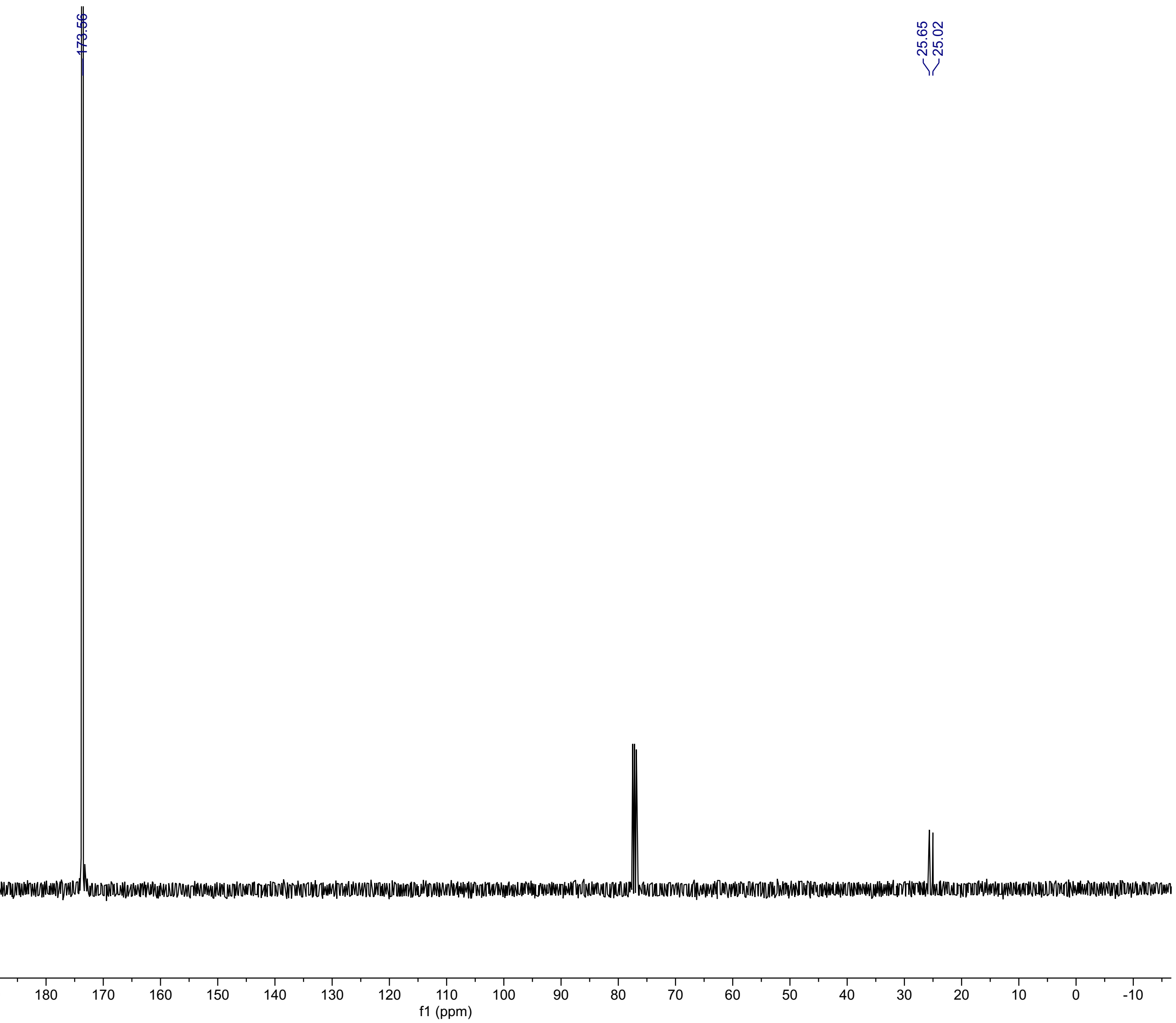

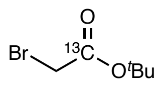

^{13}C-**S4**

^{1}H NMR (400 MHz, $CDCl_3$)

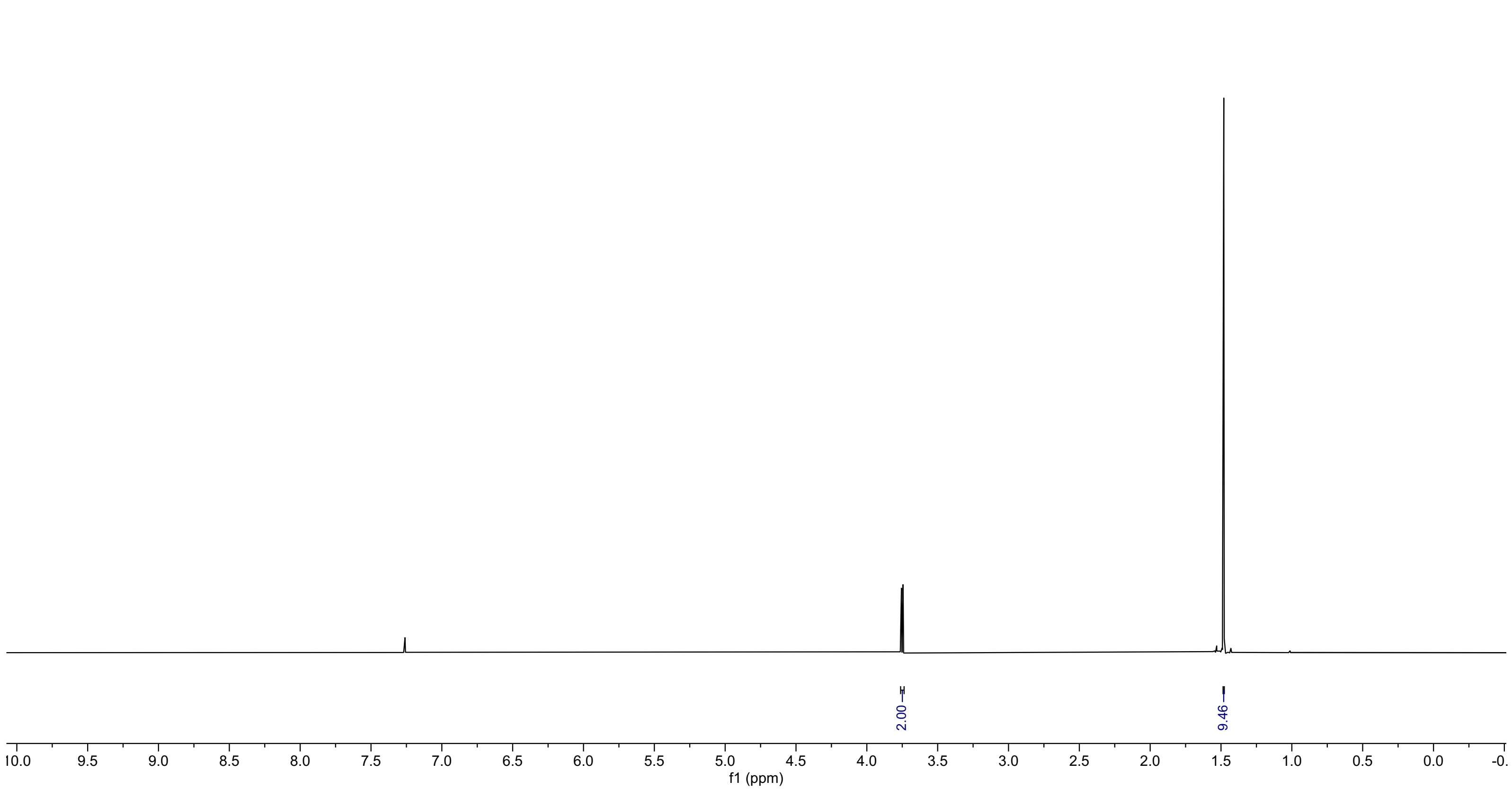

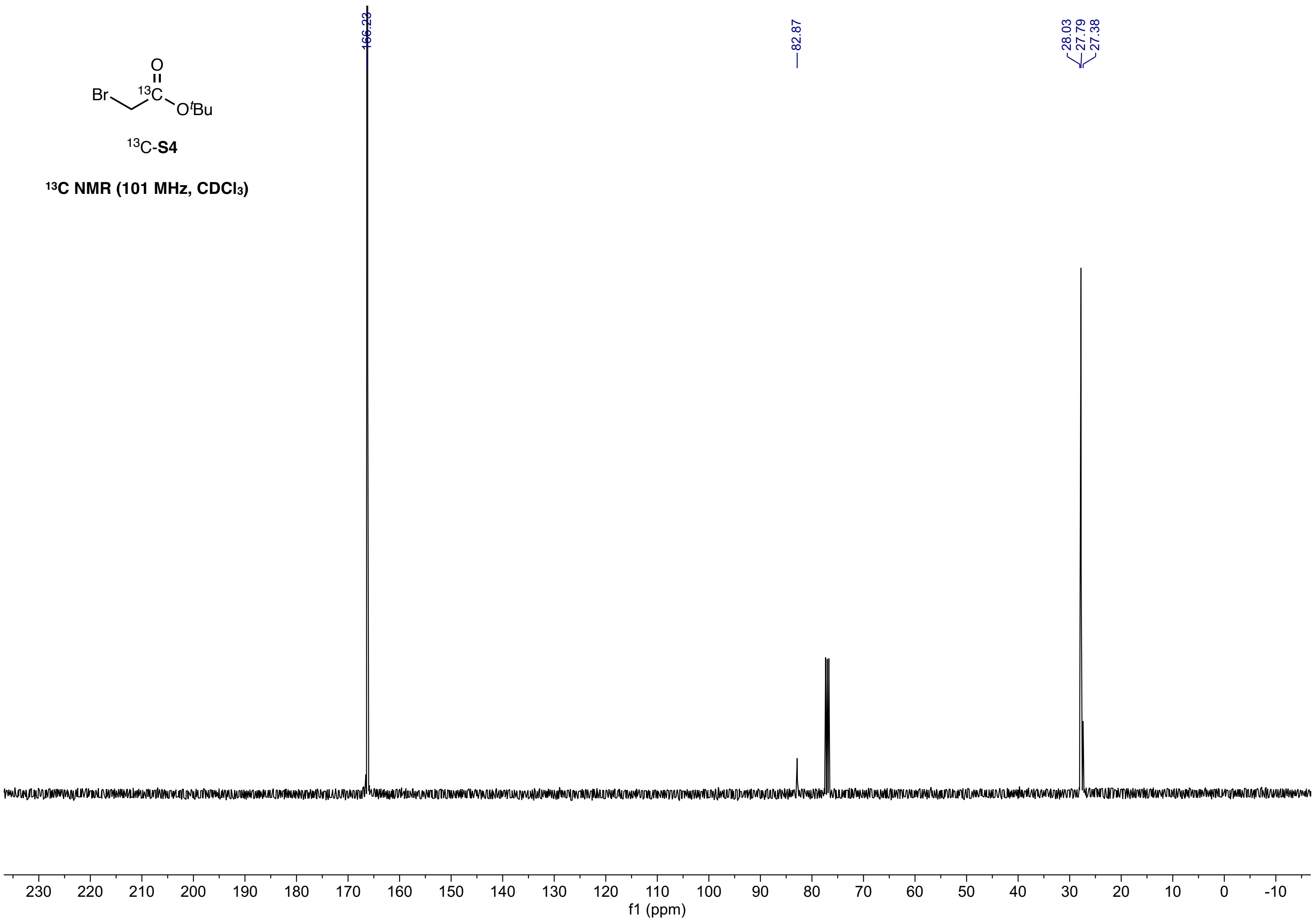
Br
13C
O
OtBu
13C-S4
13C NMR (101 MHz, CDCl3)
166.23
82.87
28.03
27.79
27.38
230 220 210 200 190 180 170 160 150 140 130 120 110 100 90 80 70 60 50 40 30 20 10 0 -10
f1 (ppm)

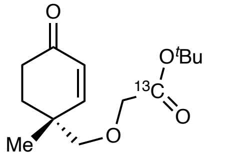

^{13}C-**17**

^{1}H NMR (400 MHz, CDCl$_3$)

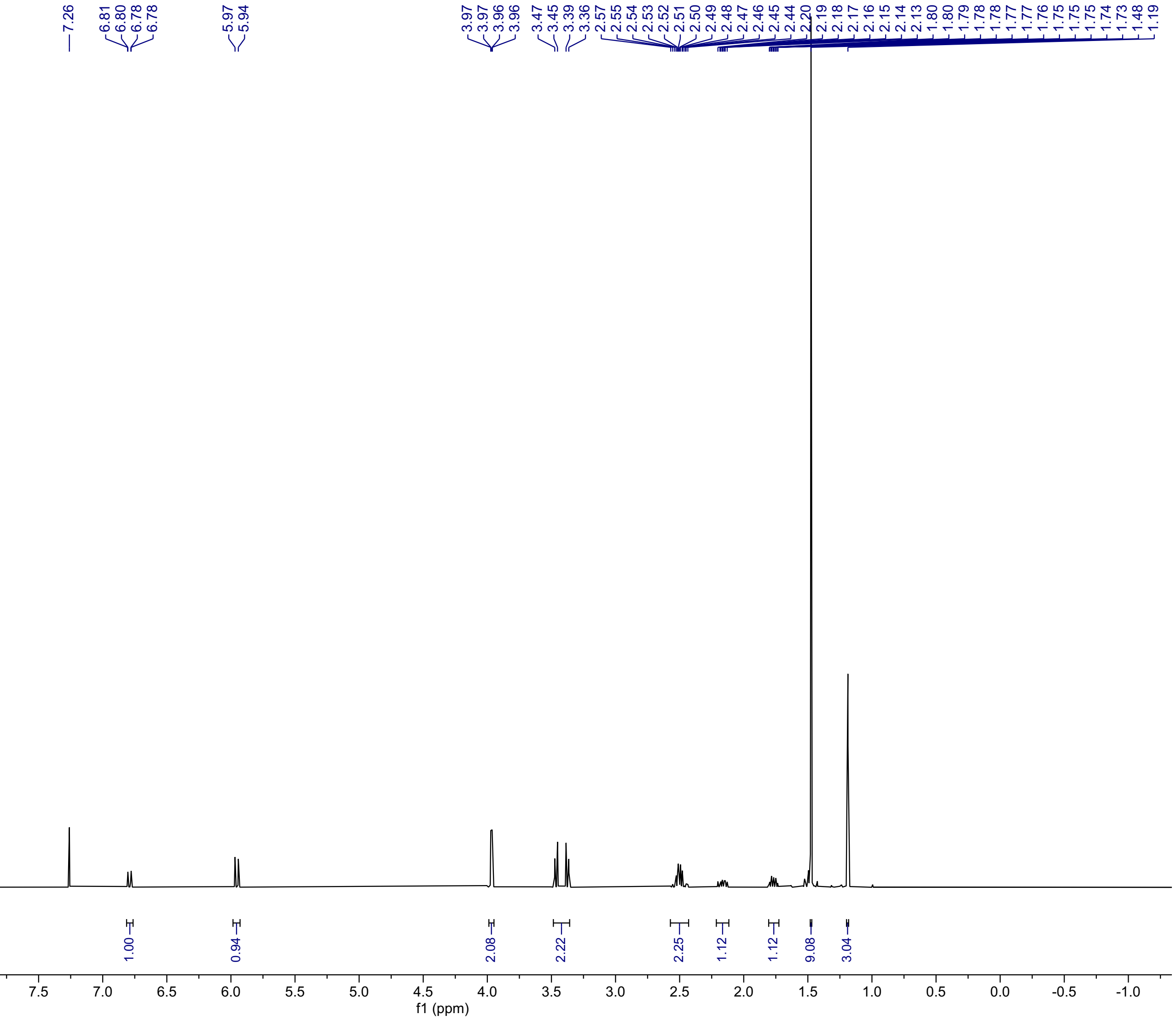

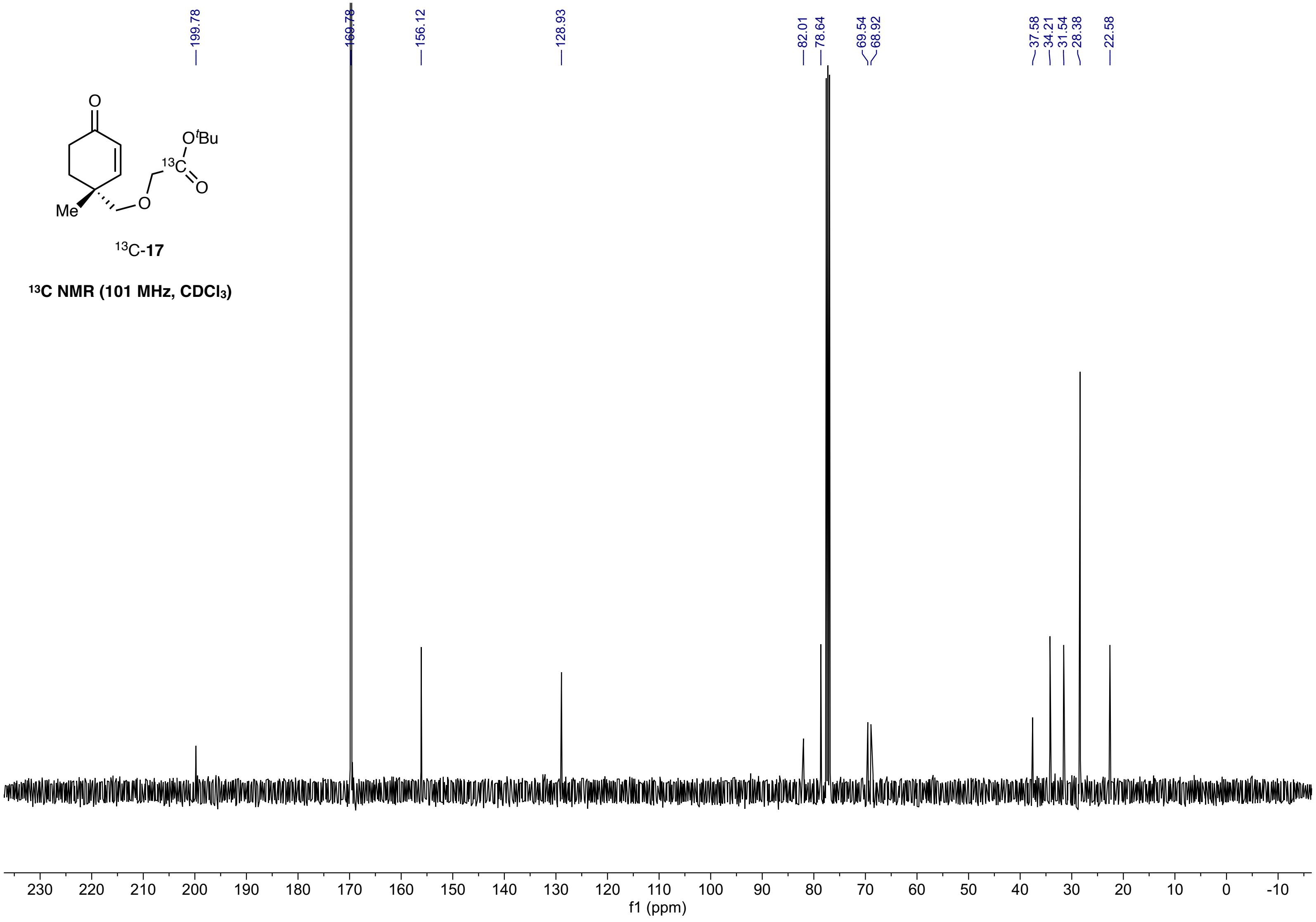
199.78
169.78
156.12
128.93
82.01
78.64
69.54
68.92
37.58
34.21
31.54
28.38
22.58
O
O^tBu
13C
O
Me
O
13C-17
13C NMR (101 MHz, CDCl3)
230 220 210 200 190 180 170 160 150 140 130 120 110 100 90 80 70 60 50 40 30 20 10 0 -10
f1 (ppm)

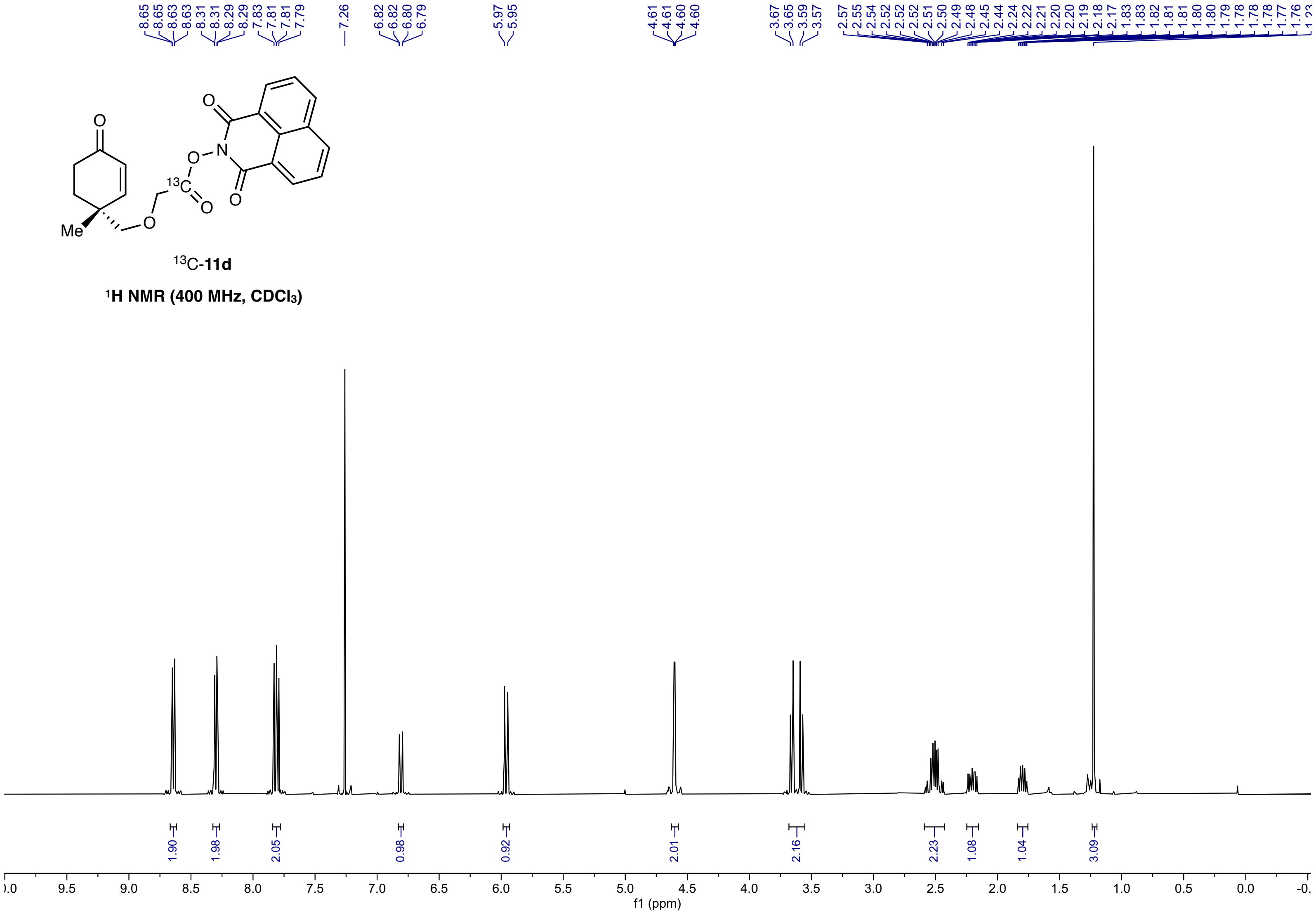

^{13}C-**11d**

^{1}H NMR (400 MHz, $CDCl_3$)

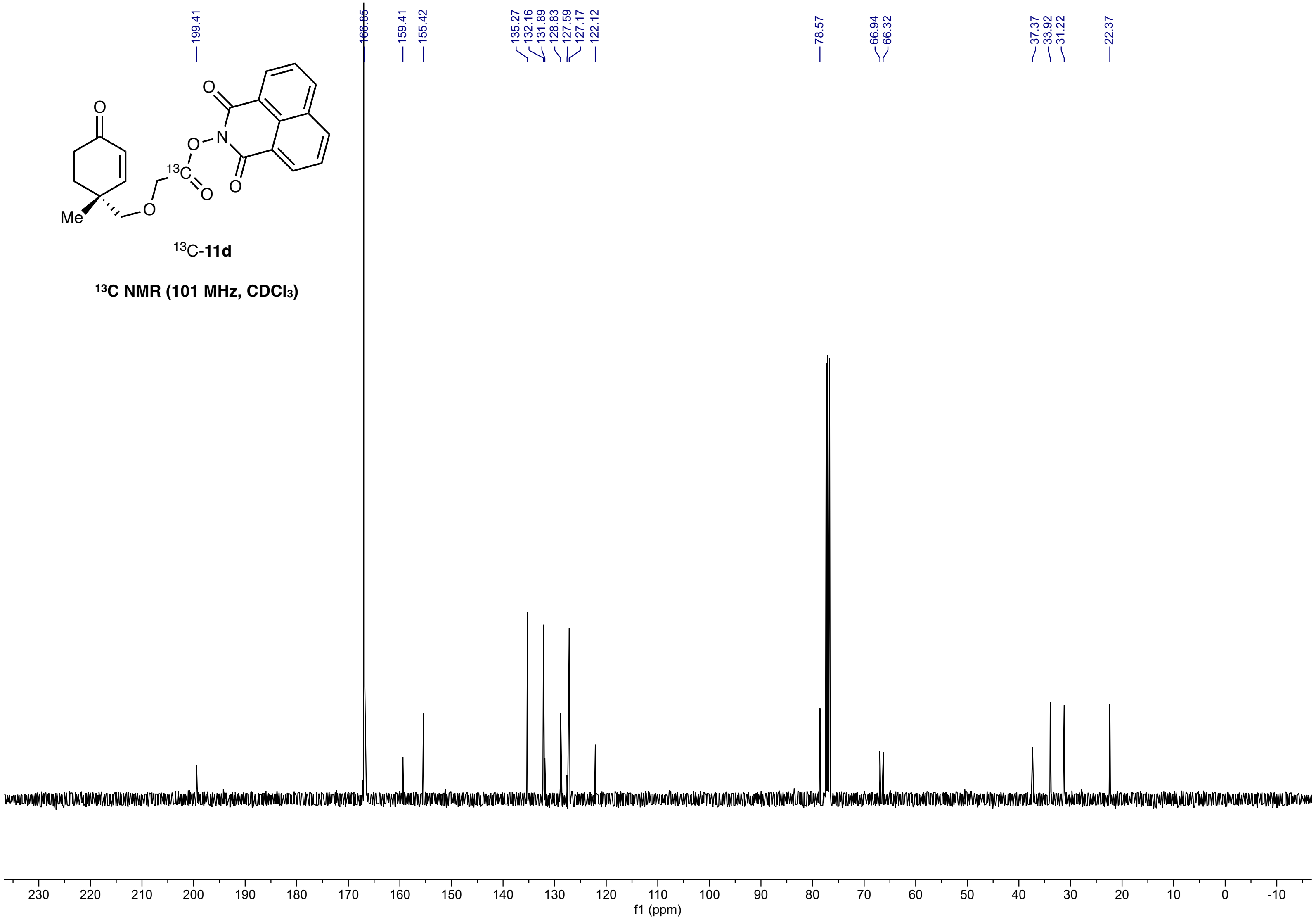

^{13}C-**11d**

^{13}C NMR (101 MHz, CDCl$_3$)

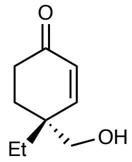

S8

^{1}H NMR (400 MHz, CDCl$_3$)

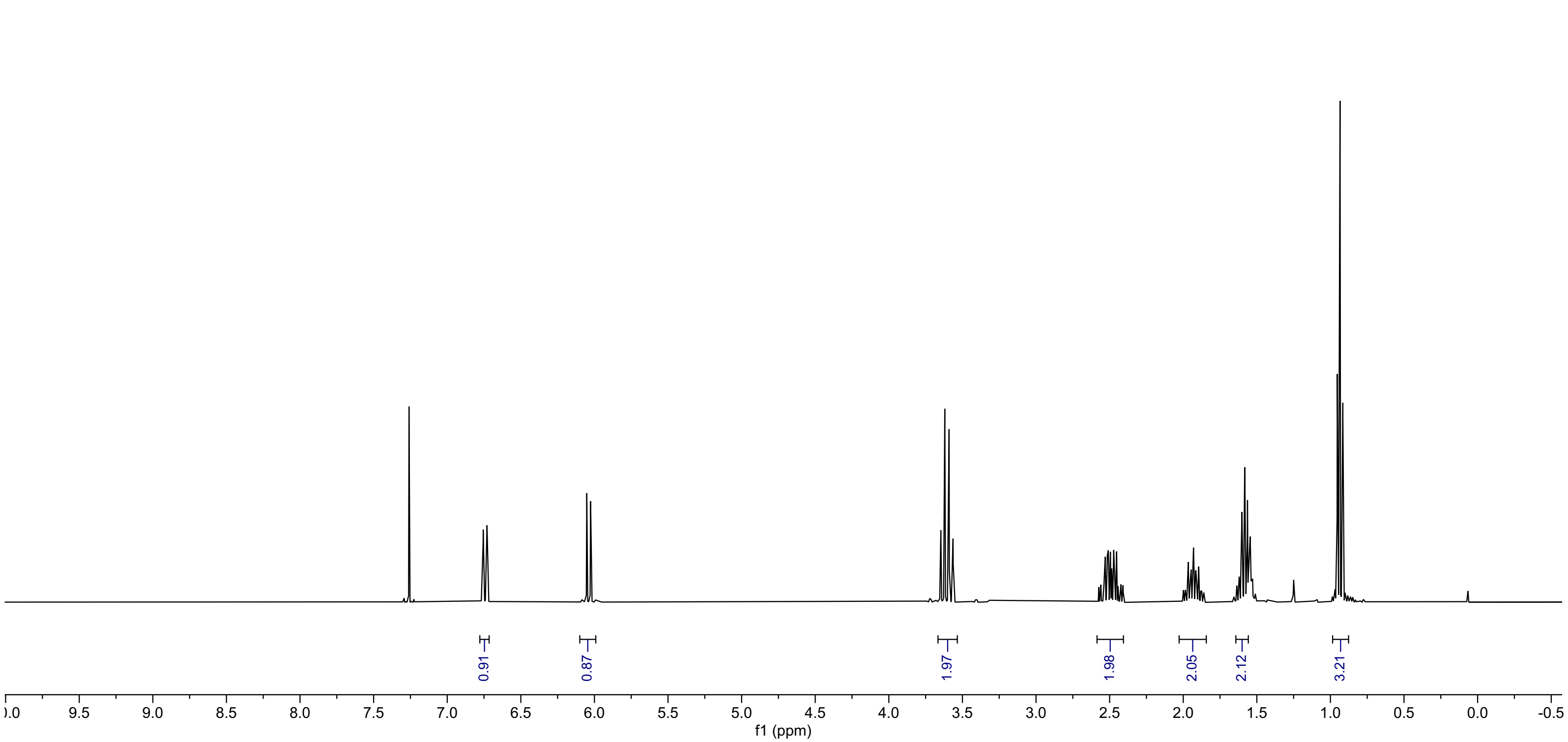

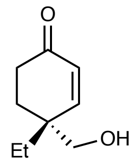

S8

^{13}C NMR (101 MHz, $CDCl_3$)

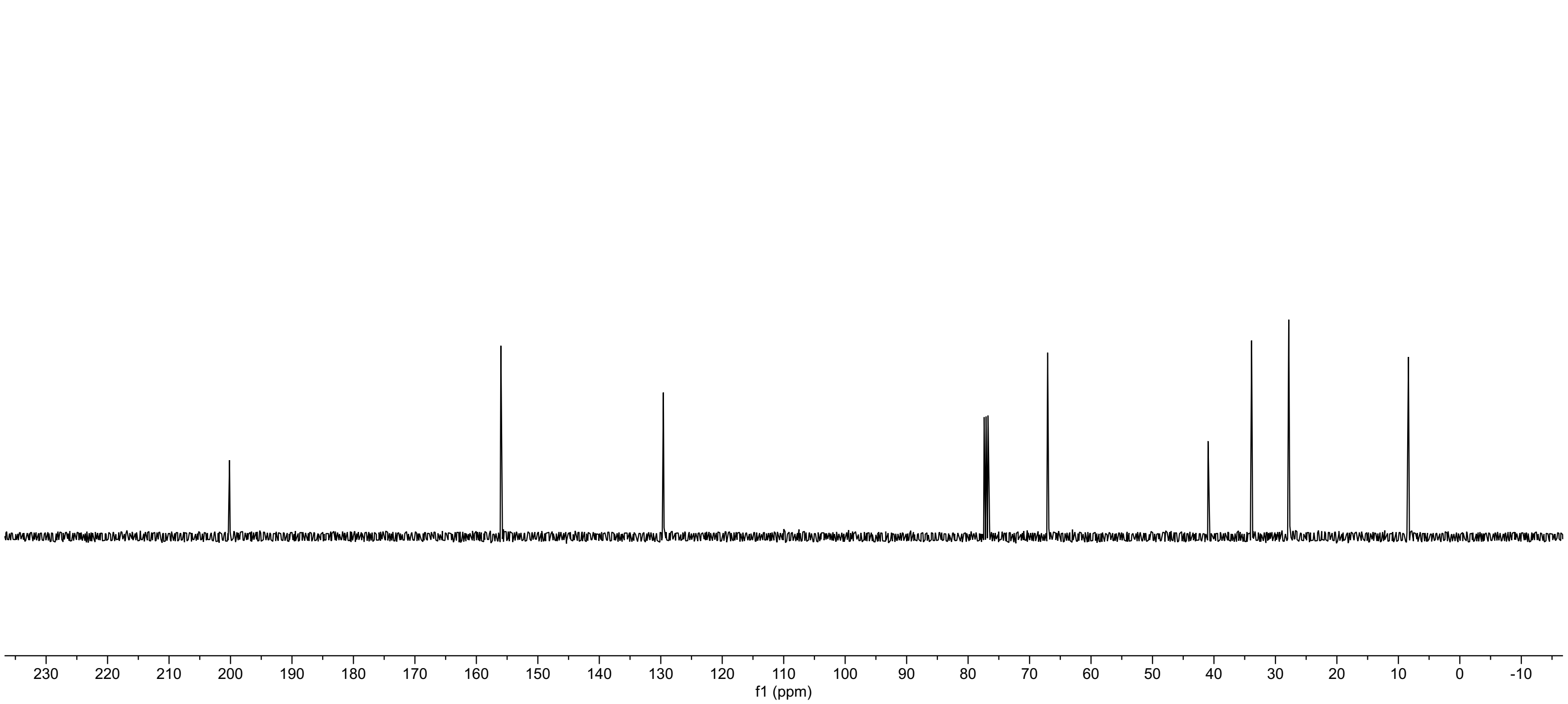

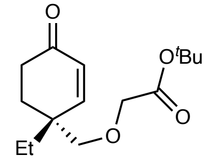

S9

^{1}H NMR (400 MHz, CDCl$_3$)

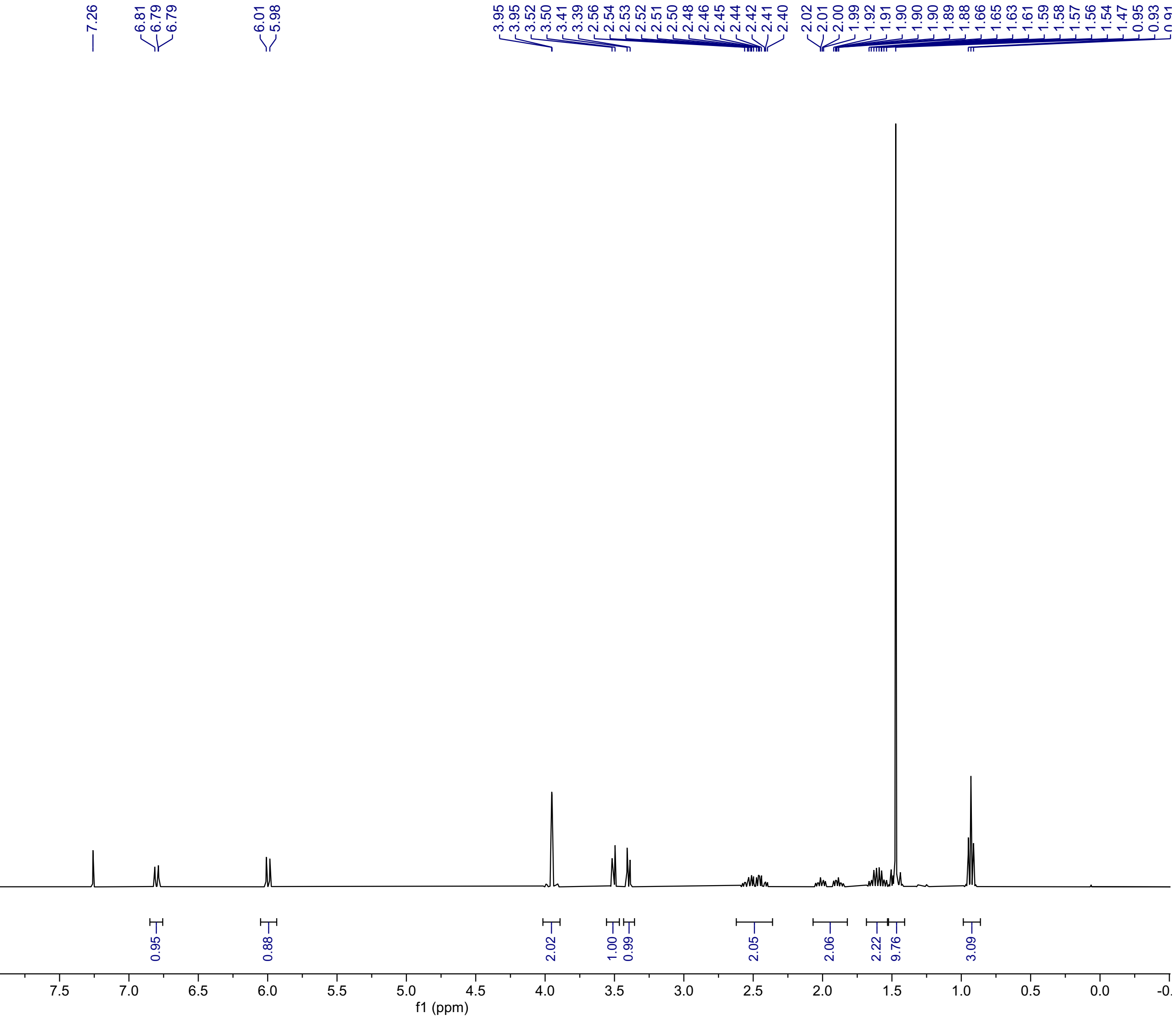

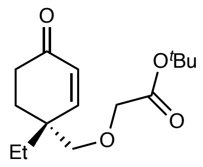

S9

^{13}C NMR (101 MHz, $CDCl_3$)

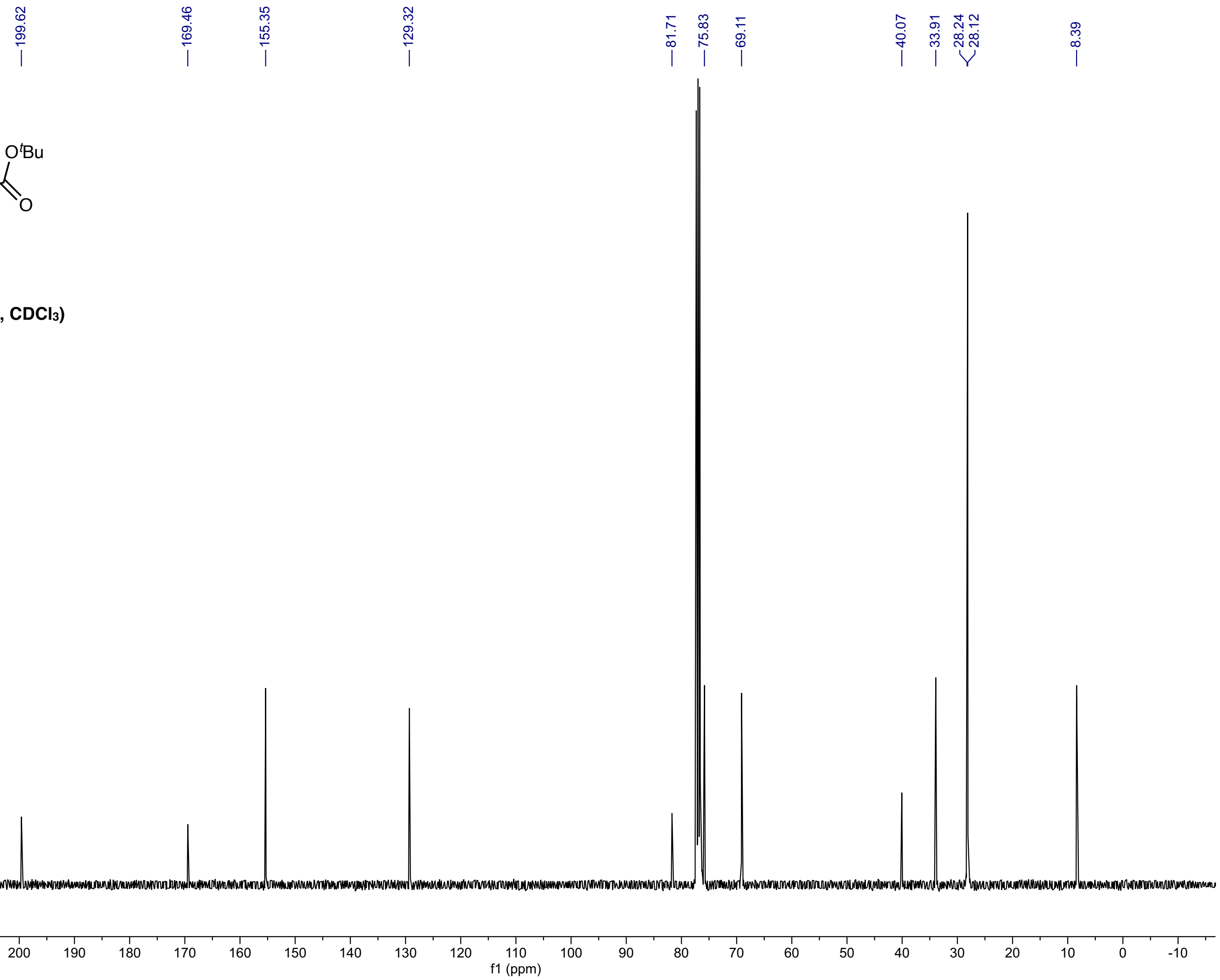

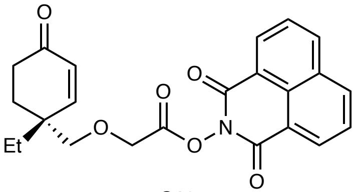

S10

¹H NMR (400 MHz, CDCl₃)

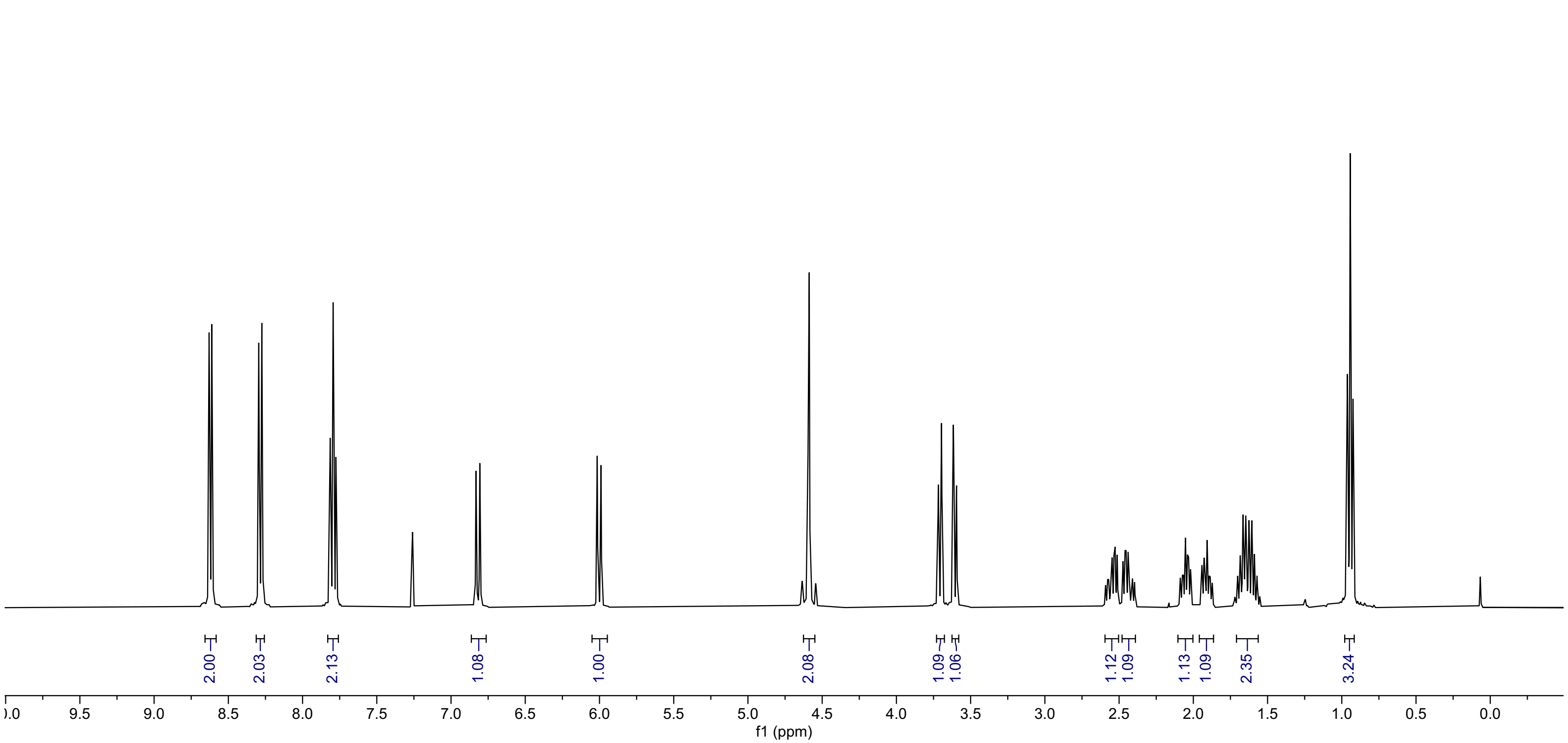

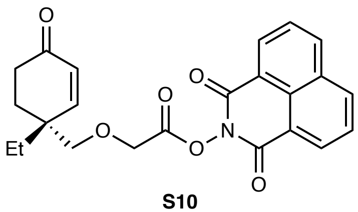

S10

^{13}C NMR (101 MHz, CDCl$_3$)

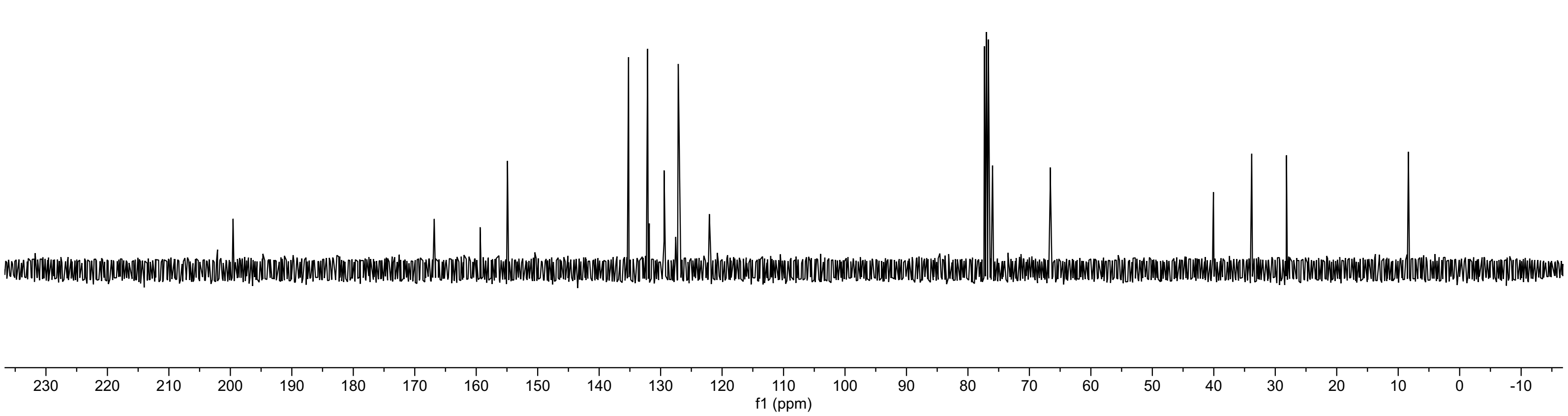

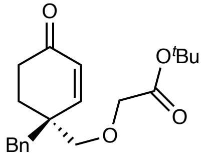

S13

¹H NMR (400 MHz, CDCl₃)

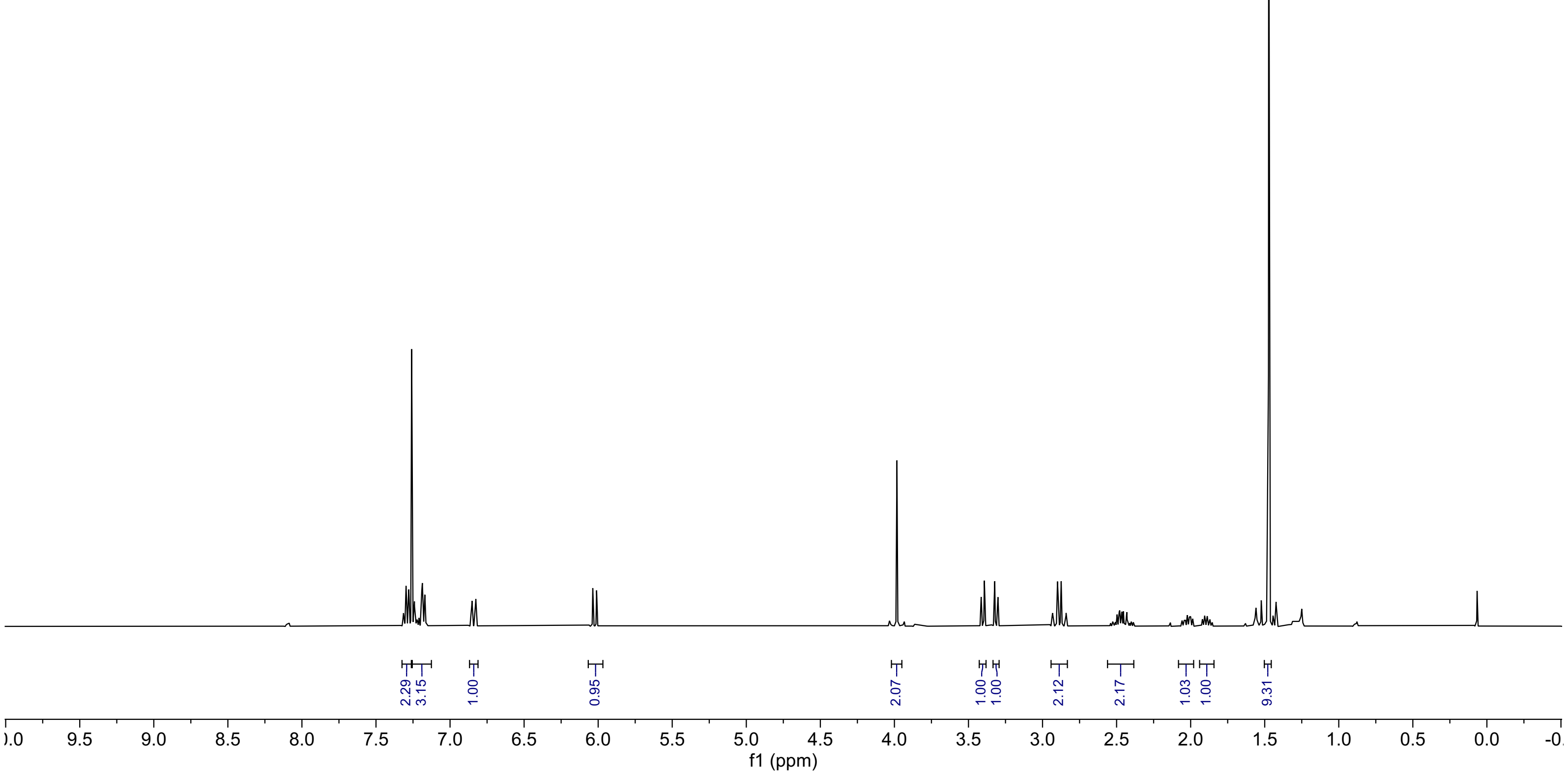

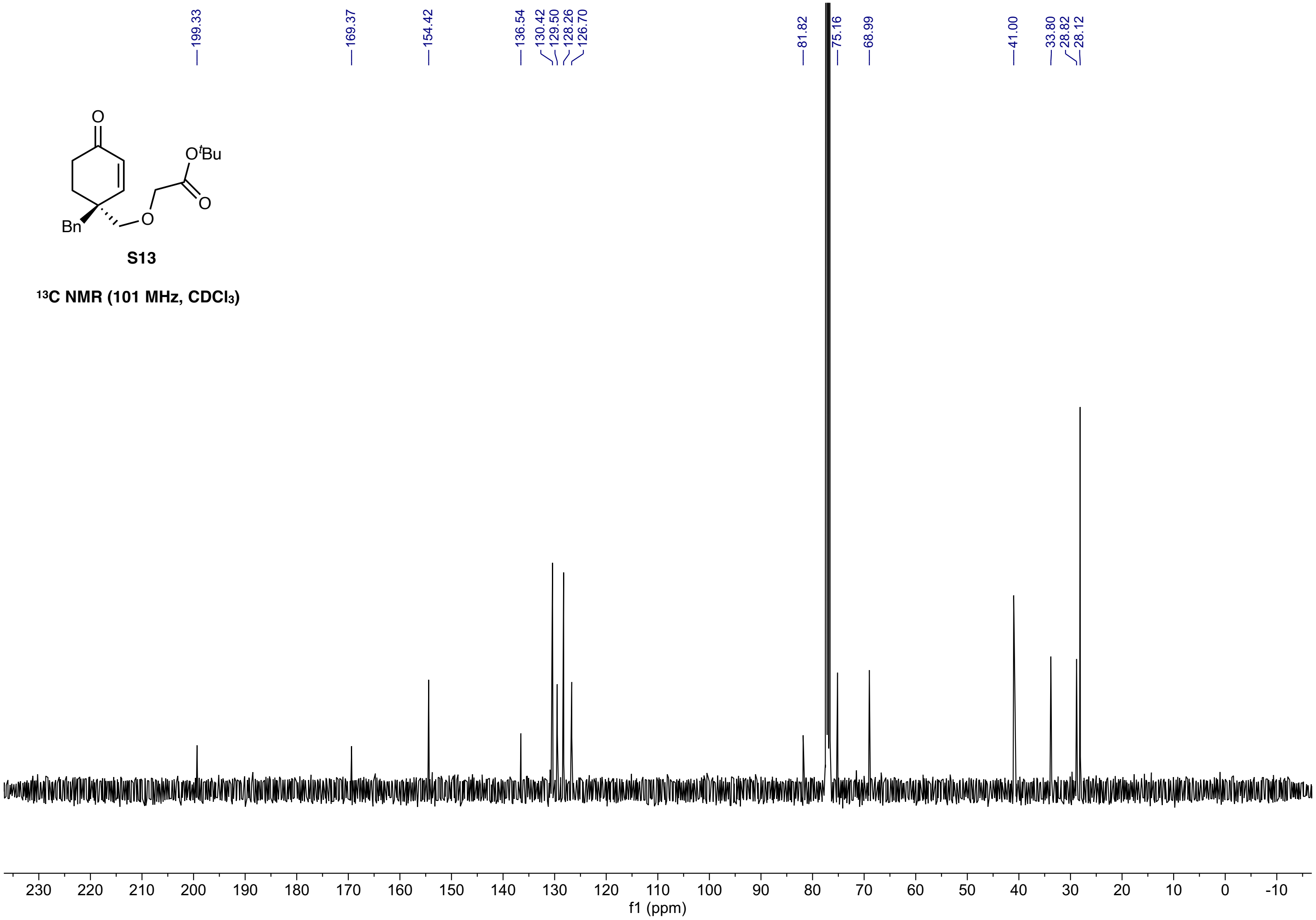

S13

^{13}C NMR (101 MHz, CDCl$_3$)

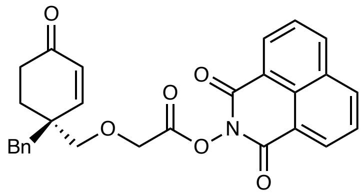

S14

^{1}H NMR (400 MHz, $CDCl_3$)

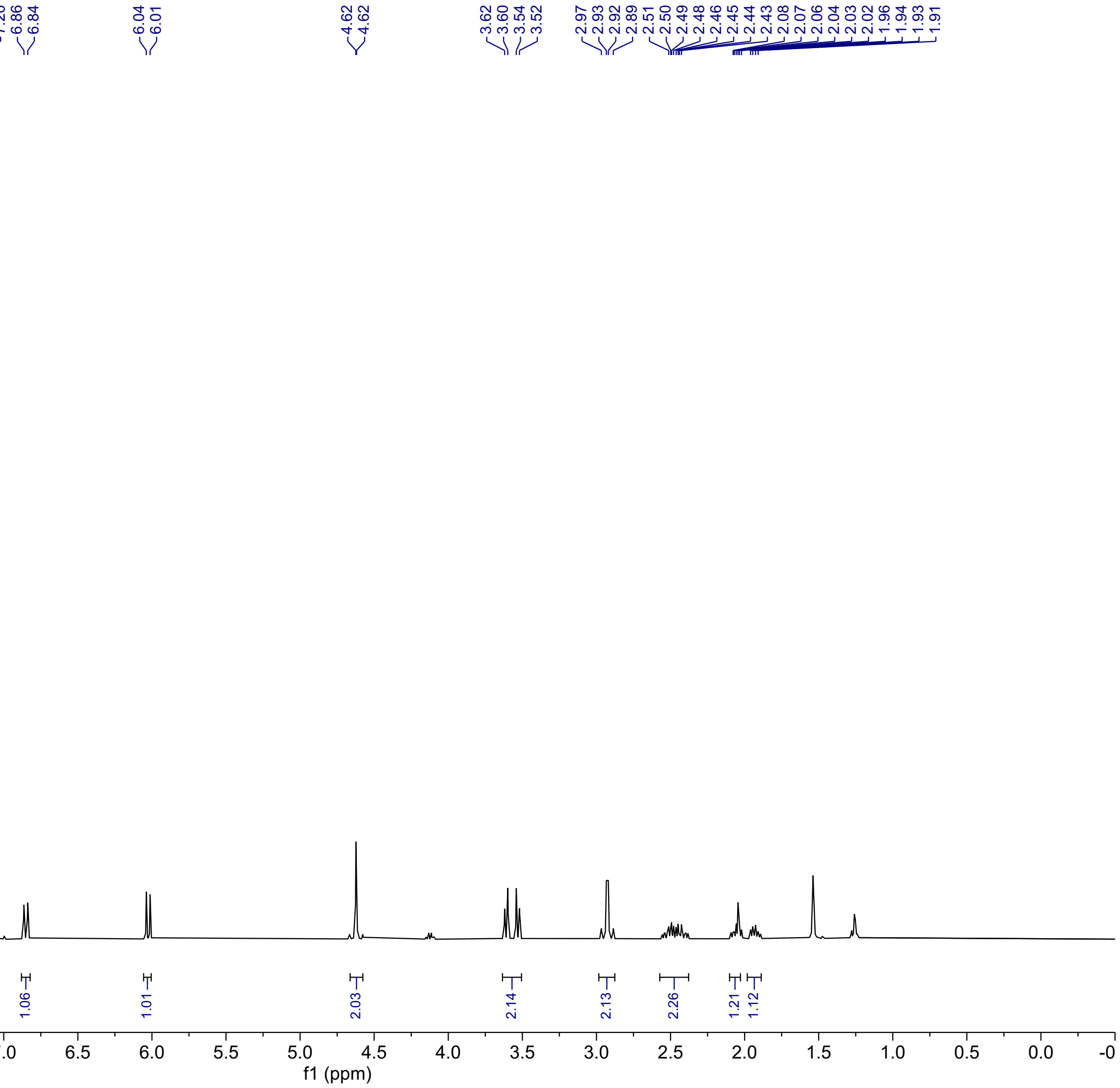

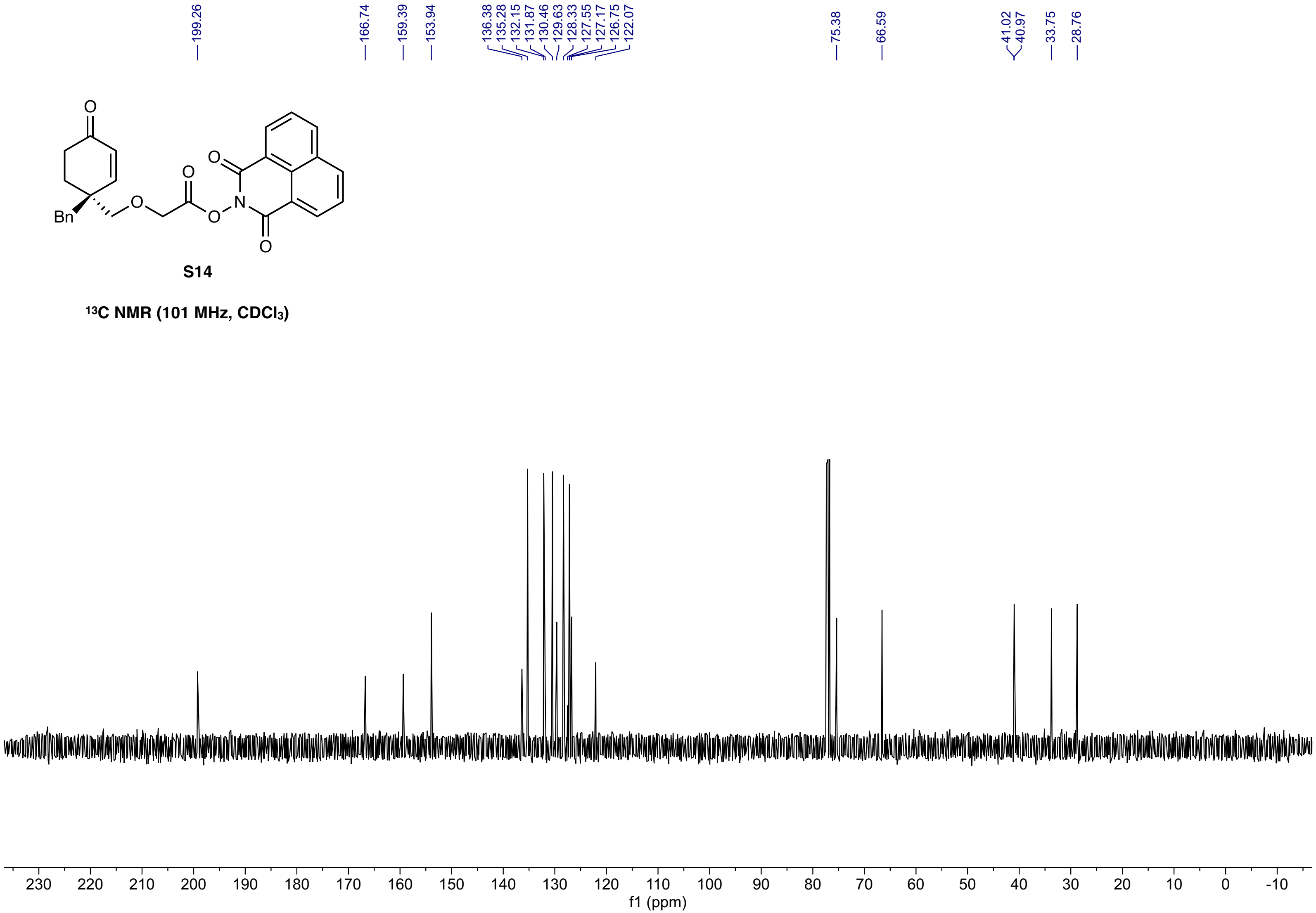
199.26
166.74
159.39
153.94
136.38
135.28
132.15
131.87
130.46
129.63
128.33
127.55
127.17
126.75
122.07
75.38
66.59
41.02
40.97
33.75
28.76
Bn
O
N
S14
f1 (ppm)

^{13}C NMR (101 MHz, CDCl$_3$)

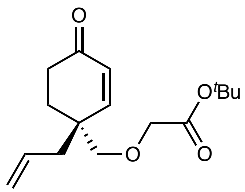

S17

^{1}H NMR (400 MHz, $CDCl_3$)

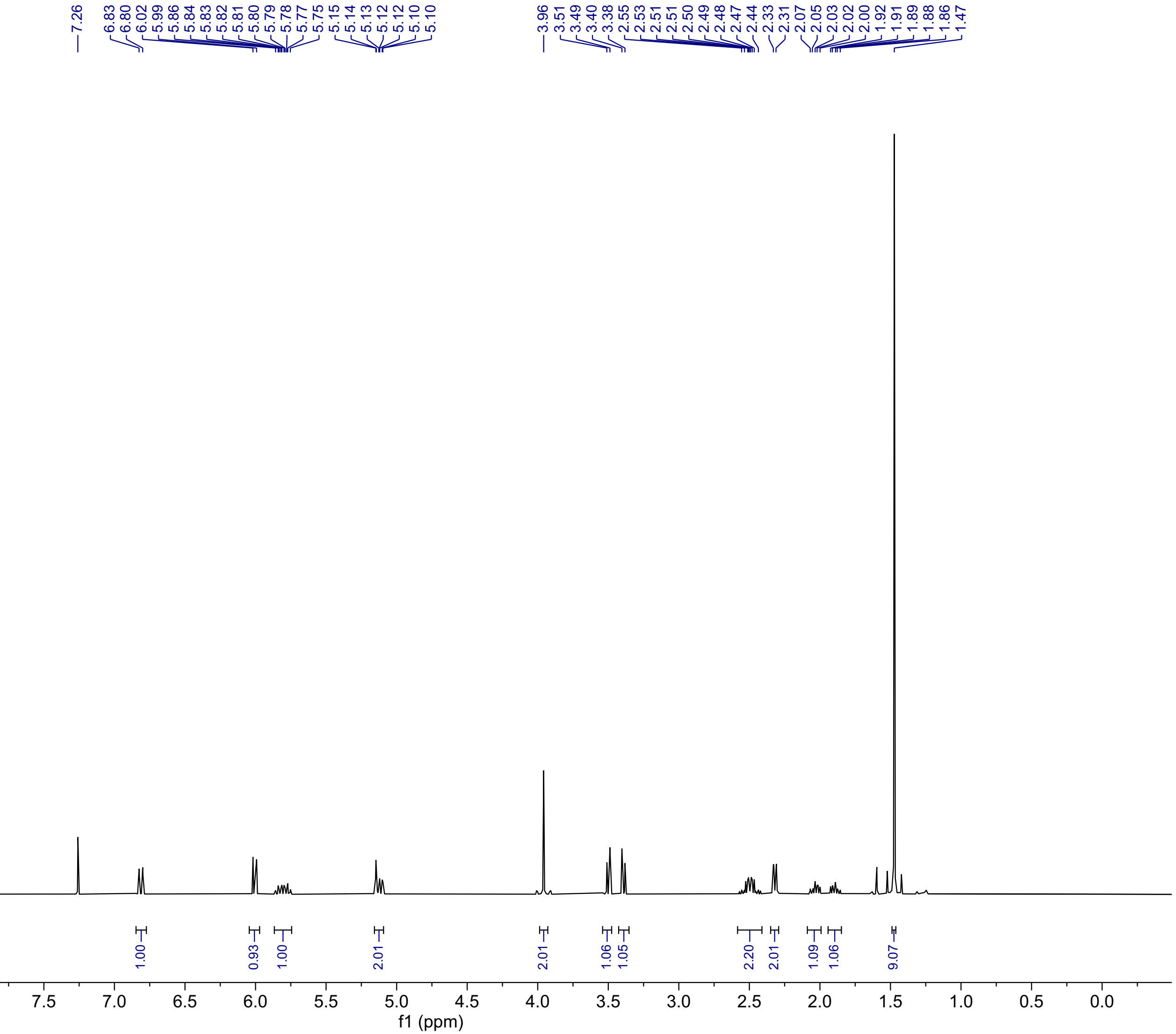

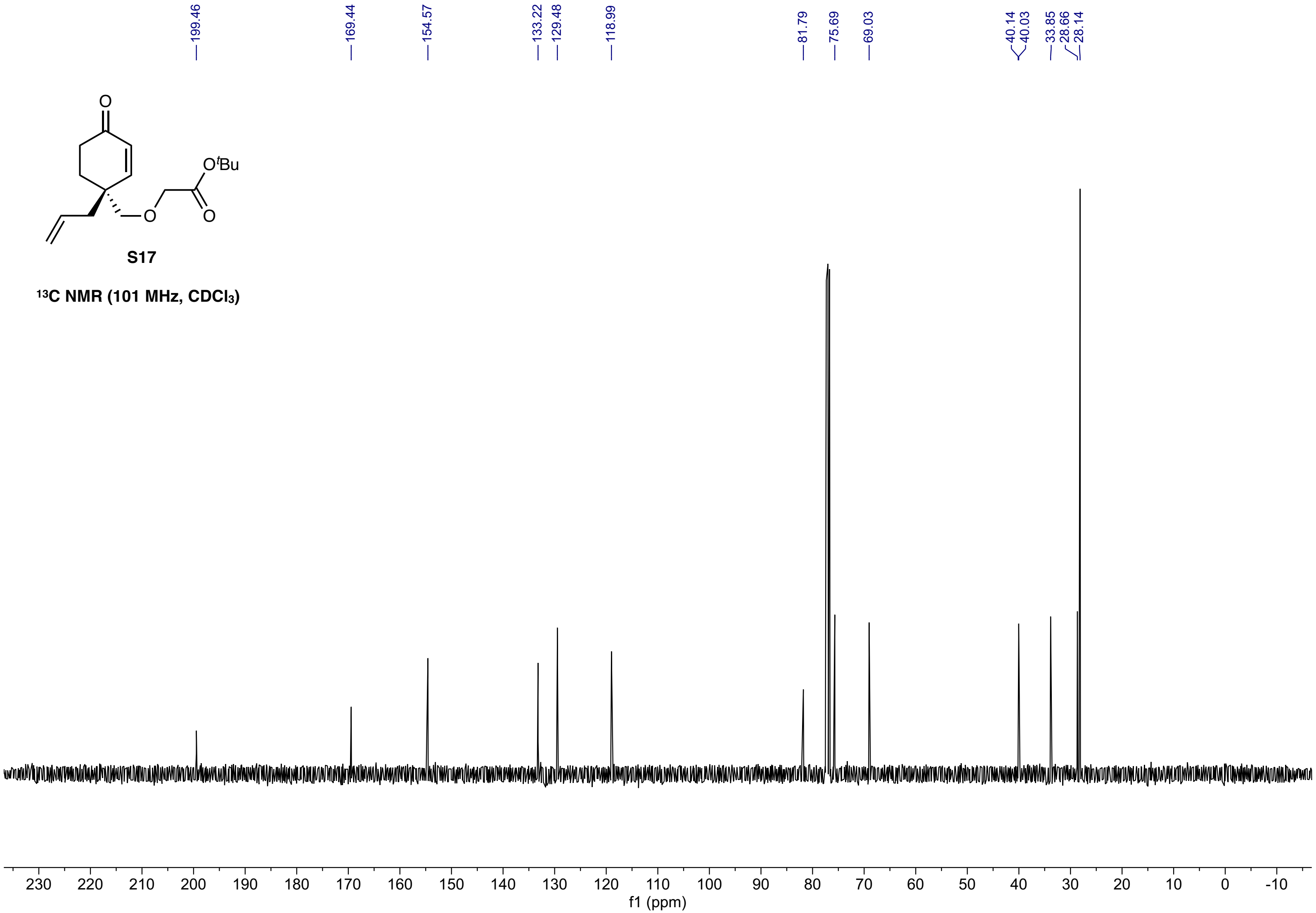
O
O^tBu
O
O
S17
13C NMR (101 MHz, CDCl3)
199.46
169.44
154.57
133.22
129.48
118.99
81.79
75.69
69.03
40.14
40.03
33.85
28.66
28.14
230 220 210 200 190 180 170 160 150 140 130 120 110 100 90 80 70 60 50 40 30 20 10 0 -10
f1 (ppm)

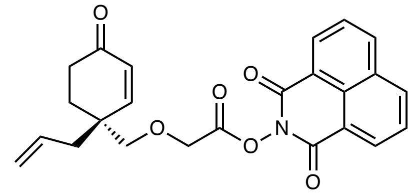

S18

^{1}H NMR (400 MHz, $CDCl_3$)

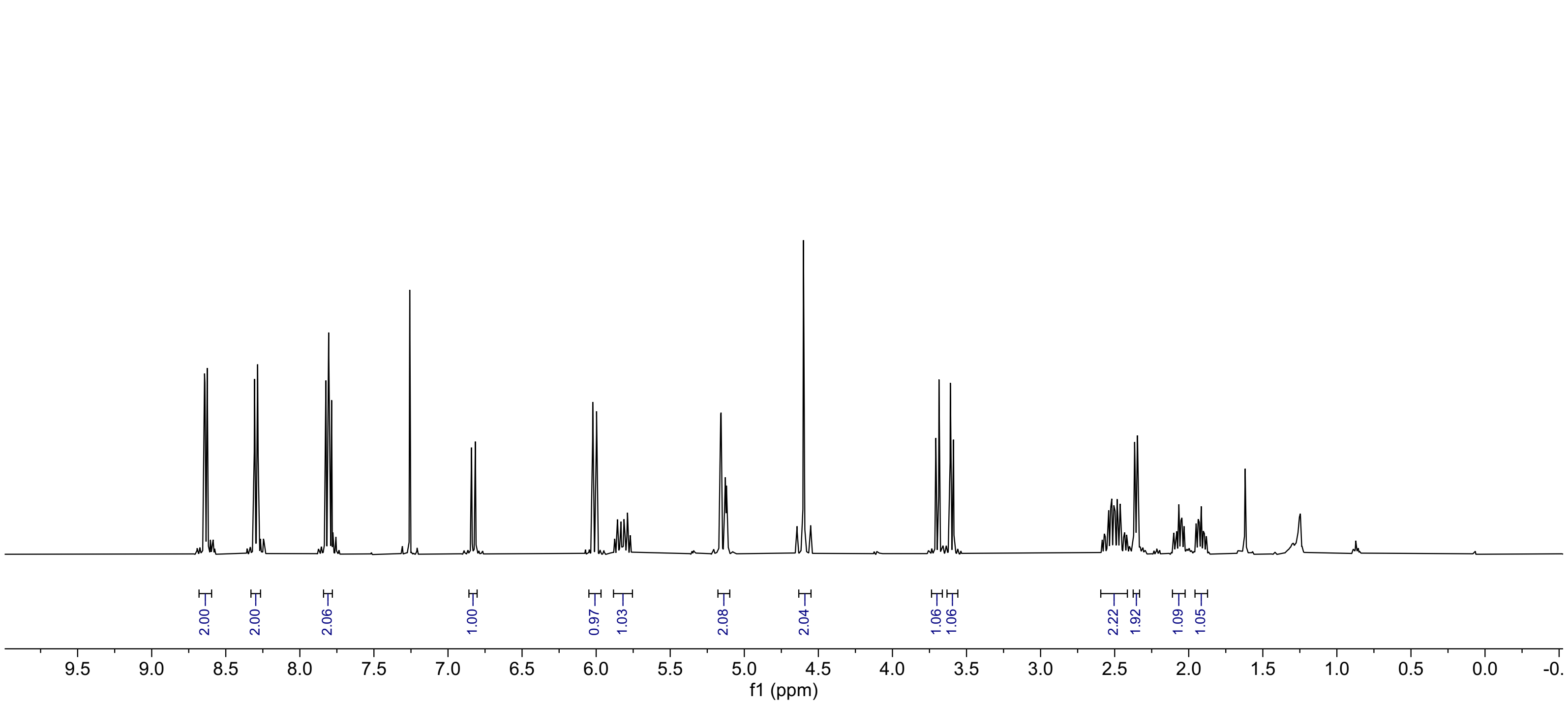

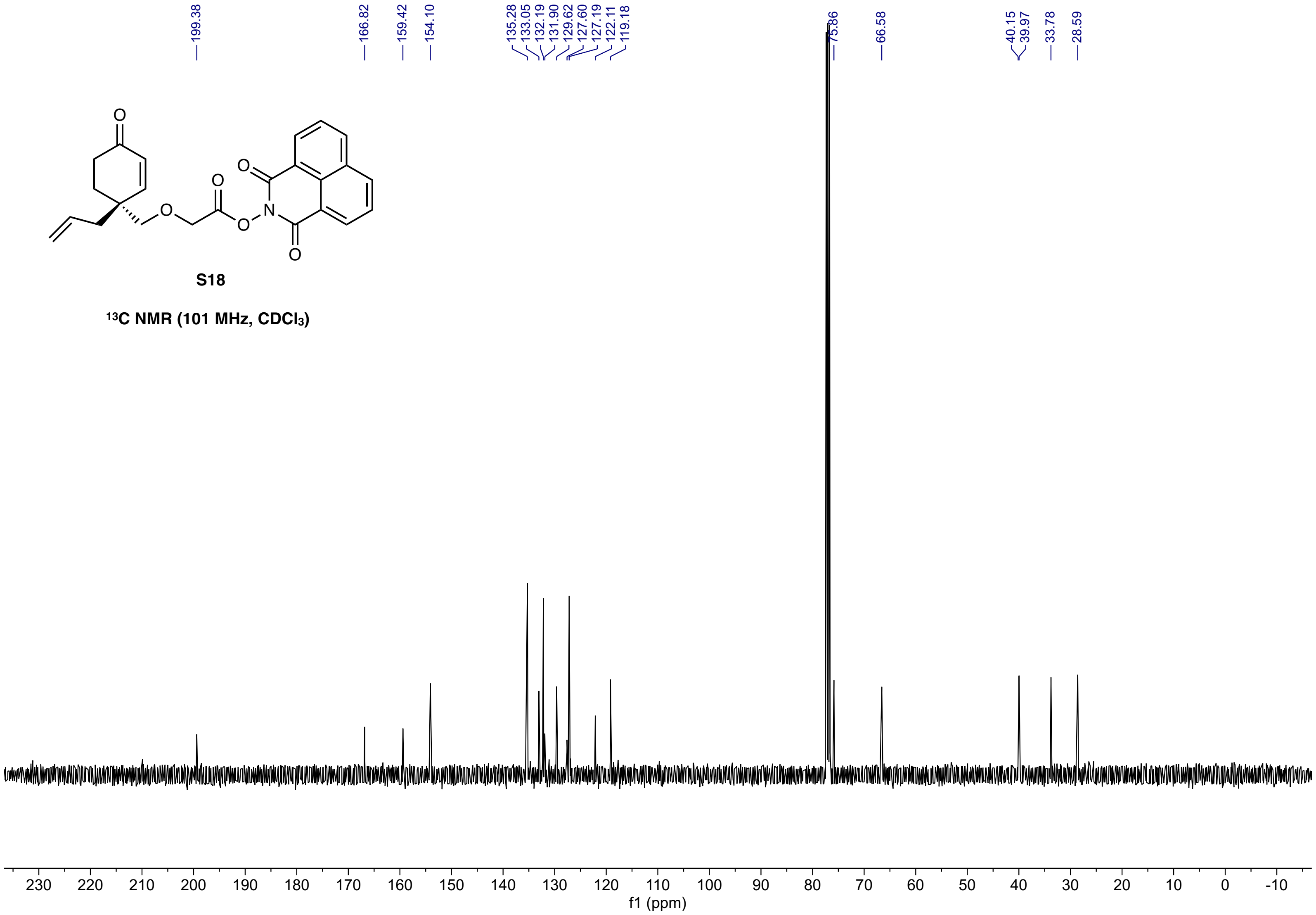

S18

^{13}C NMR (101 MHz, $CDCl_3$)

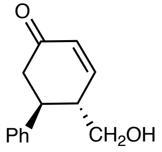

S20

^{1}H NMR (400 MHz, CDCl$_3$)

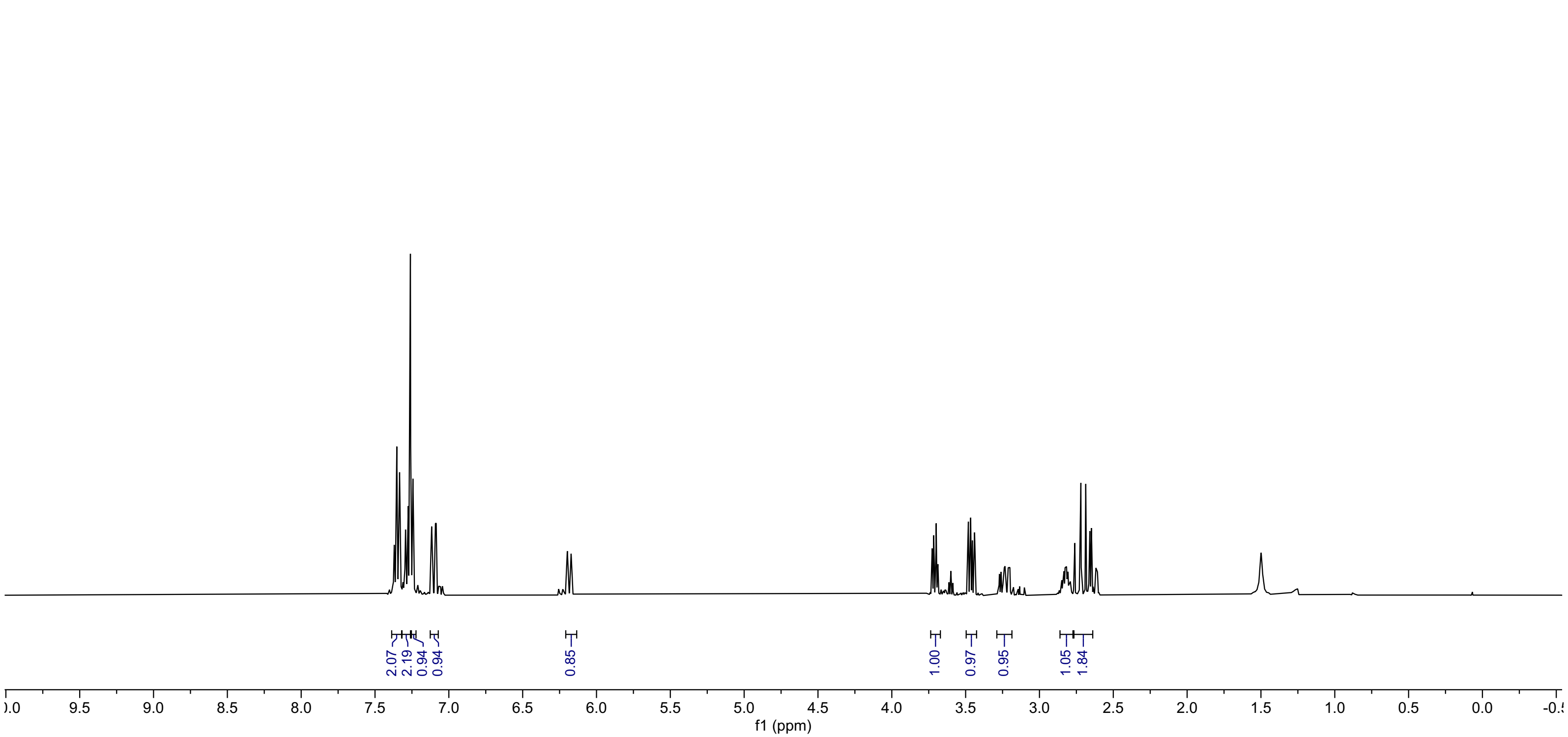

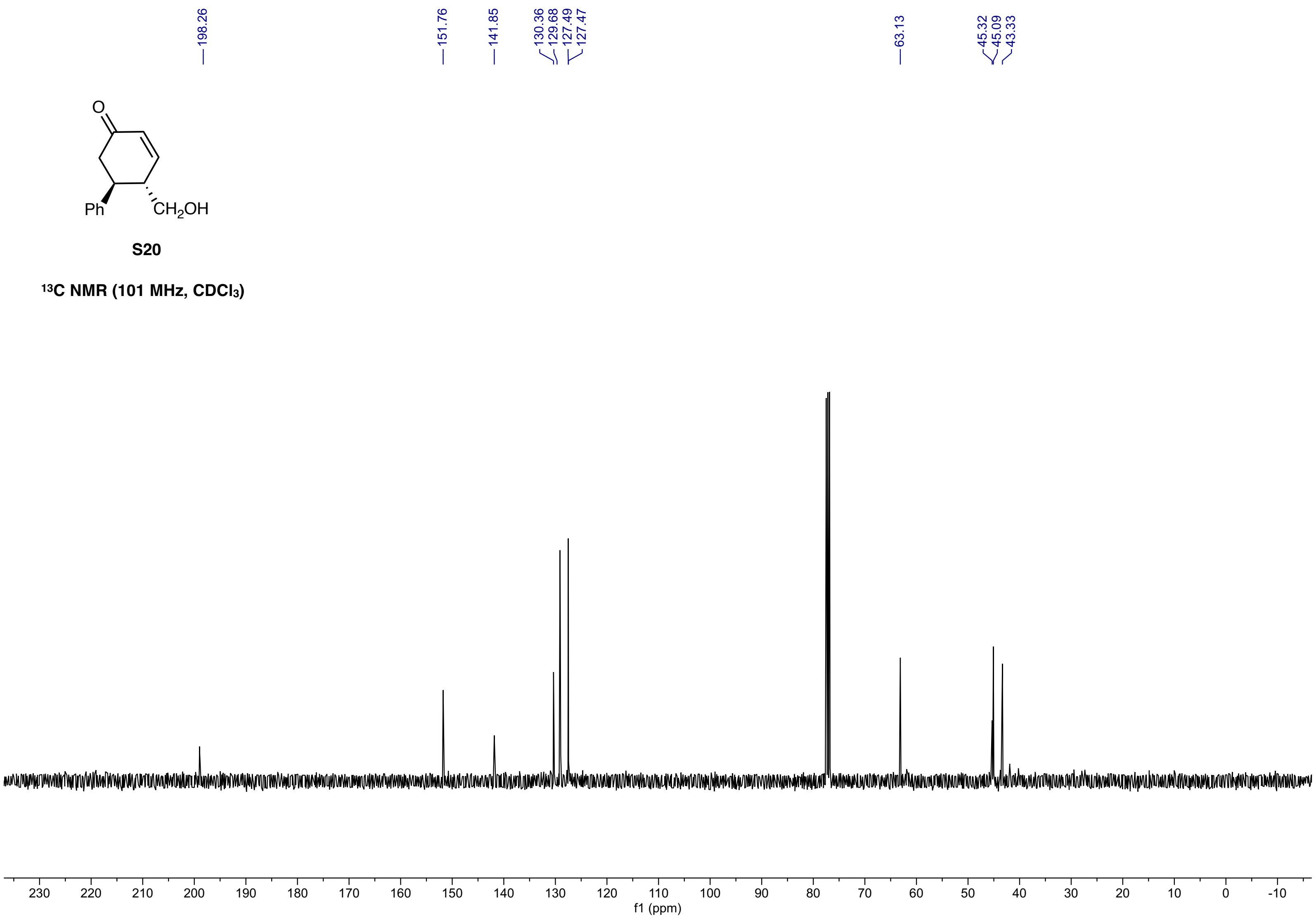

S20

^{13}C NMR (101 MHz, $CDCl_3$)

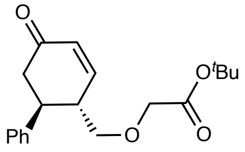

S21

^{1}H NMR (400 MHz, $CDCl_3$)

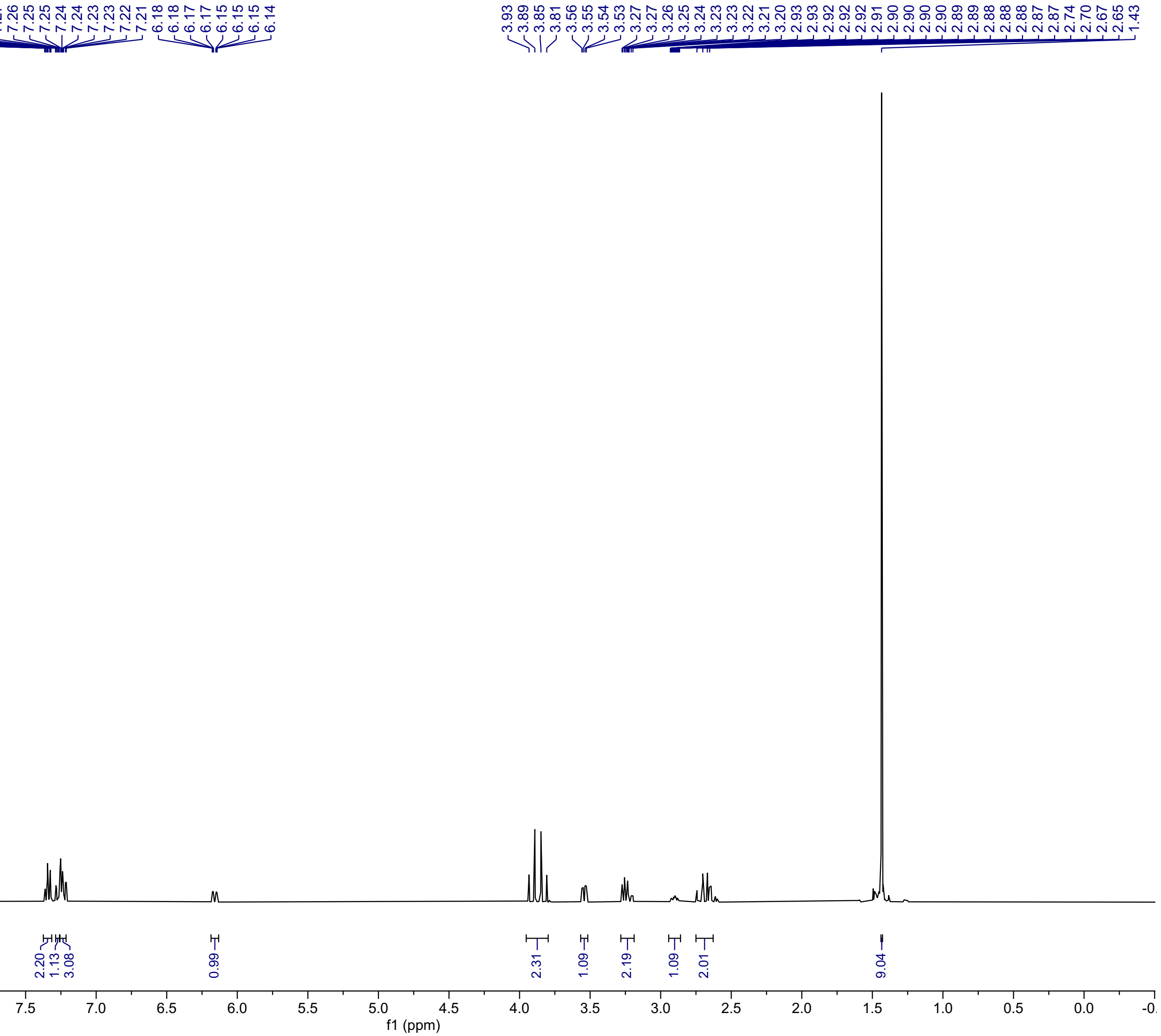

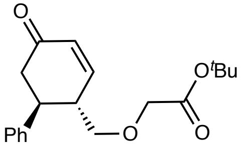

S21

^{13}C NMR (101 MHz, CDCl$_3$)

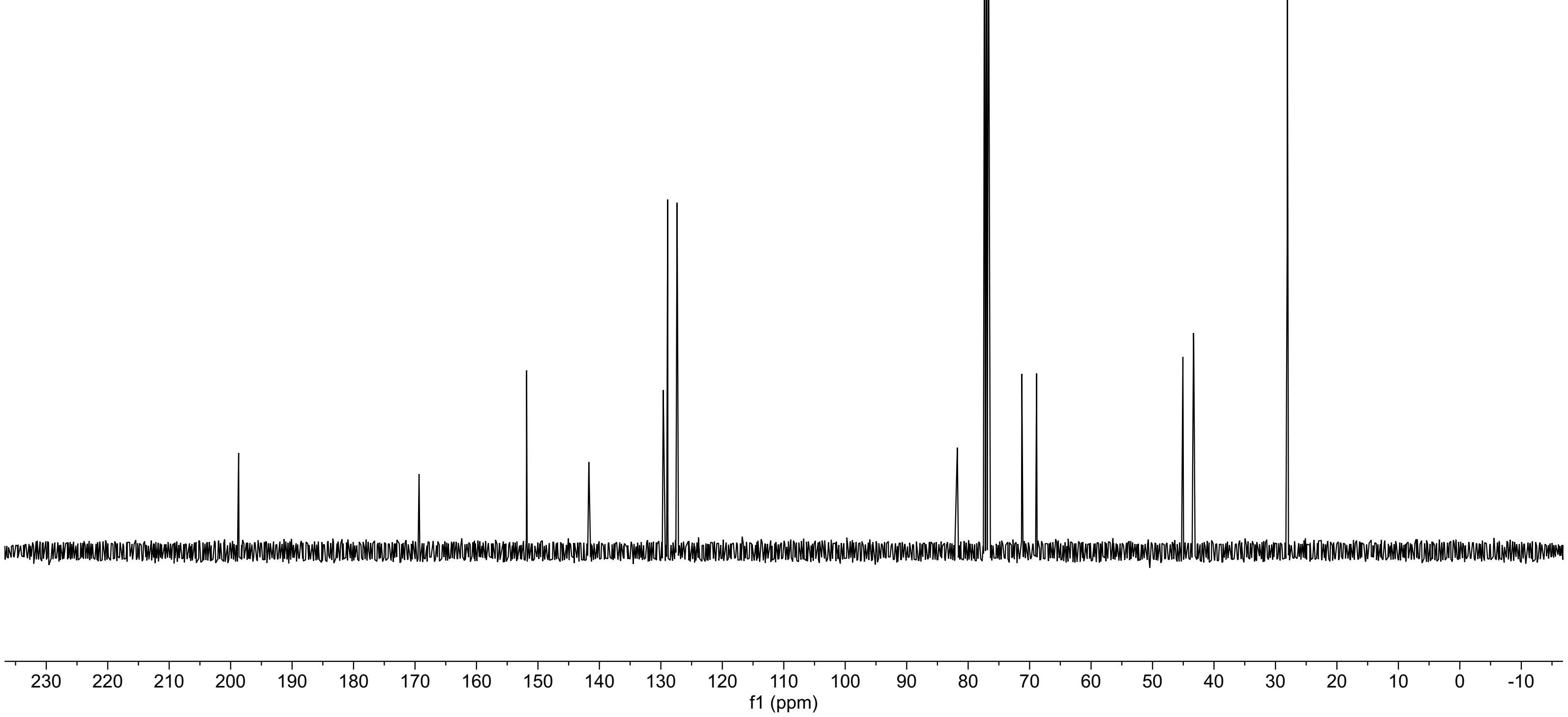

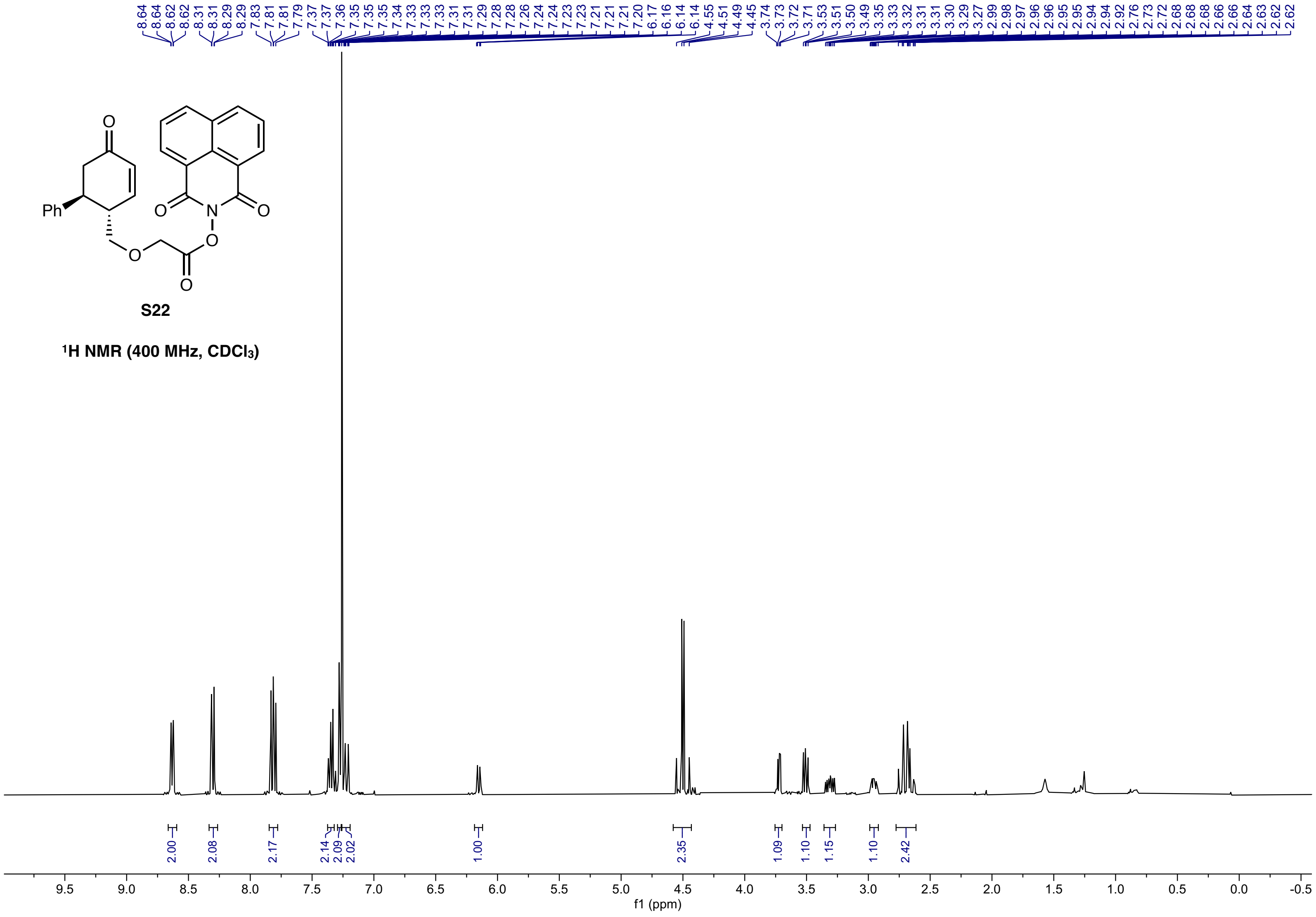

S22

^{1}H NMR (400 MHz, CDCl$_3$)

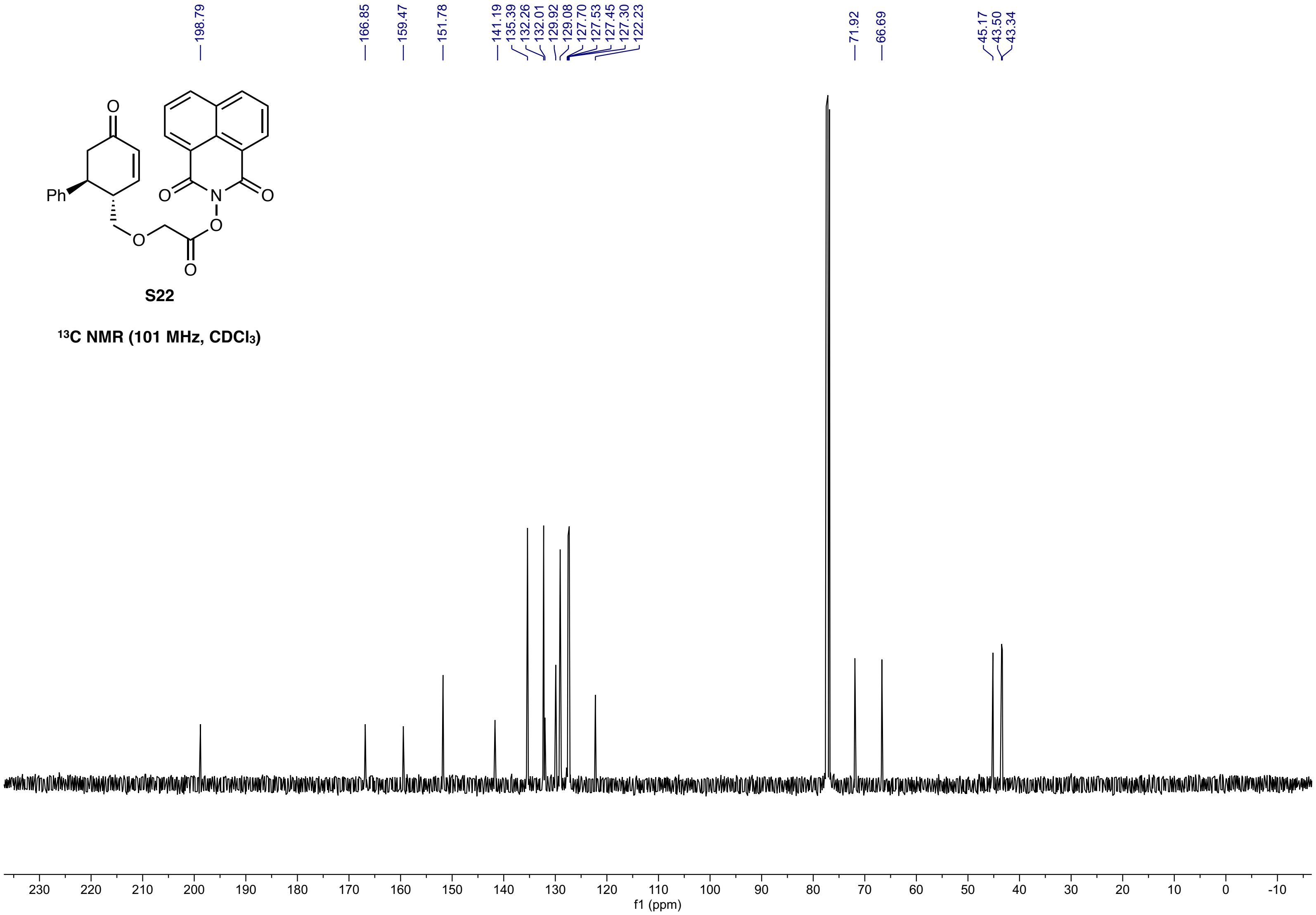
198.79
166.85
159.47
151.78
141.19
135.39
132.26
132.01
129.92
129.08
127.70
127.53
127.45
127.30
122.23
71.92
66.69
45.17
43.50
43.34
O
O
N
O
Ph
O
O
O
O
S22
^{13}C NMR (101 MHz, $CDCl_3$)
230 220 210 200 190 180 170 160 150 140 130 120 110 100 90 80 70 60 50 40 30 20 10 0 -10
f1 (ppm)

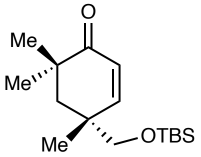

S24

^{1}H NMR (400 MHz, $CDCl_3$)

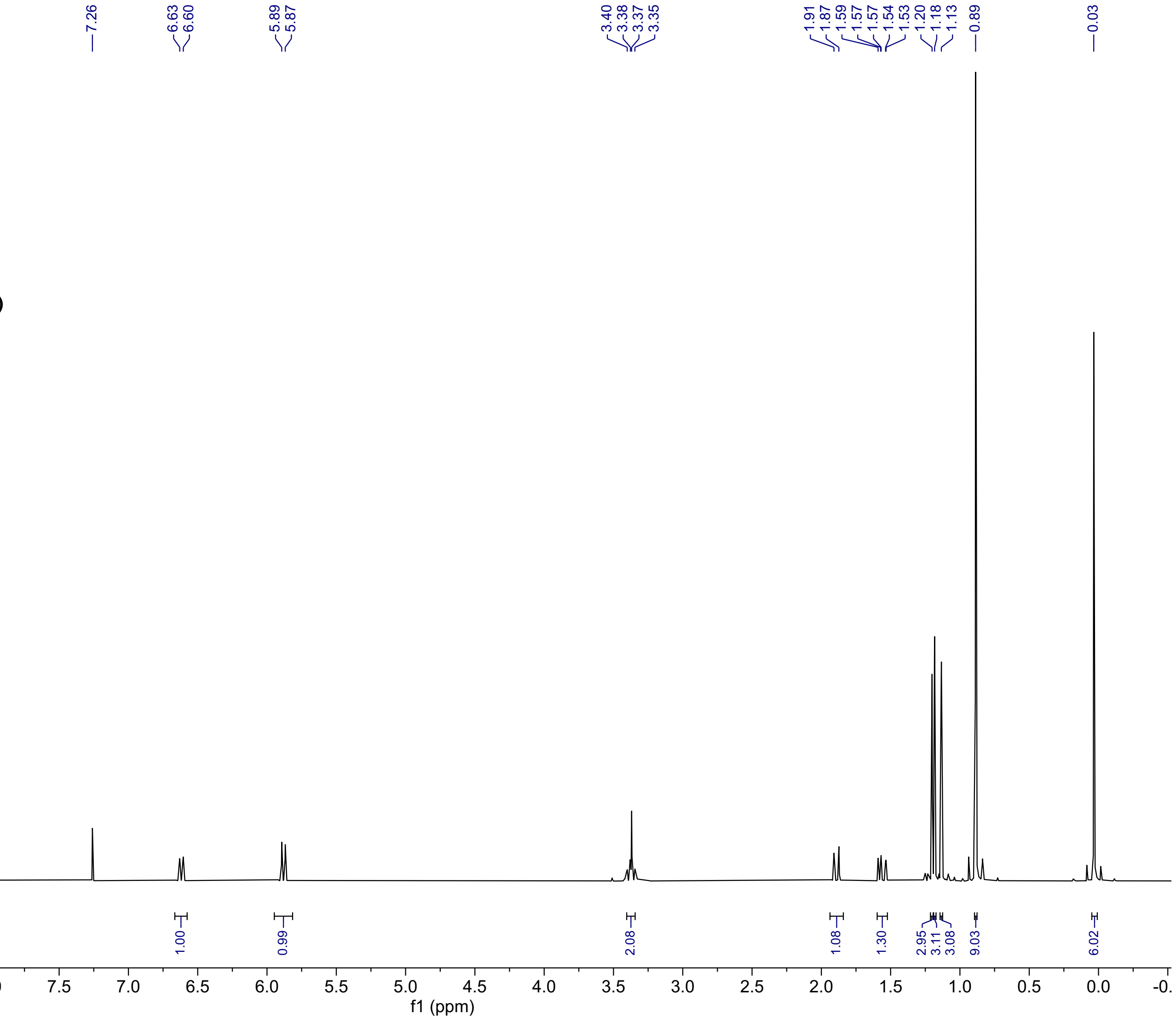

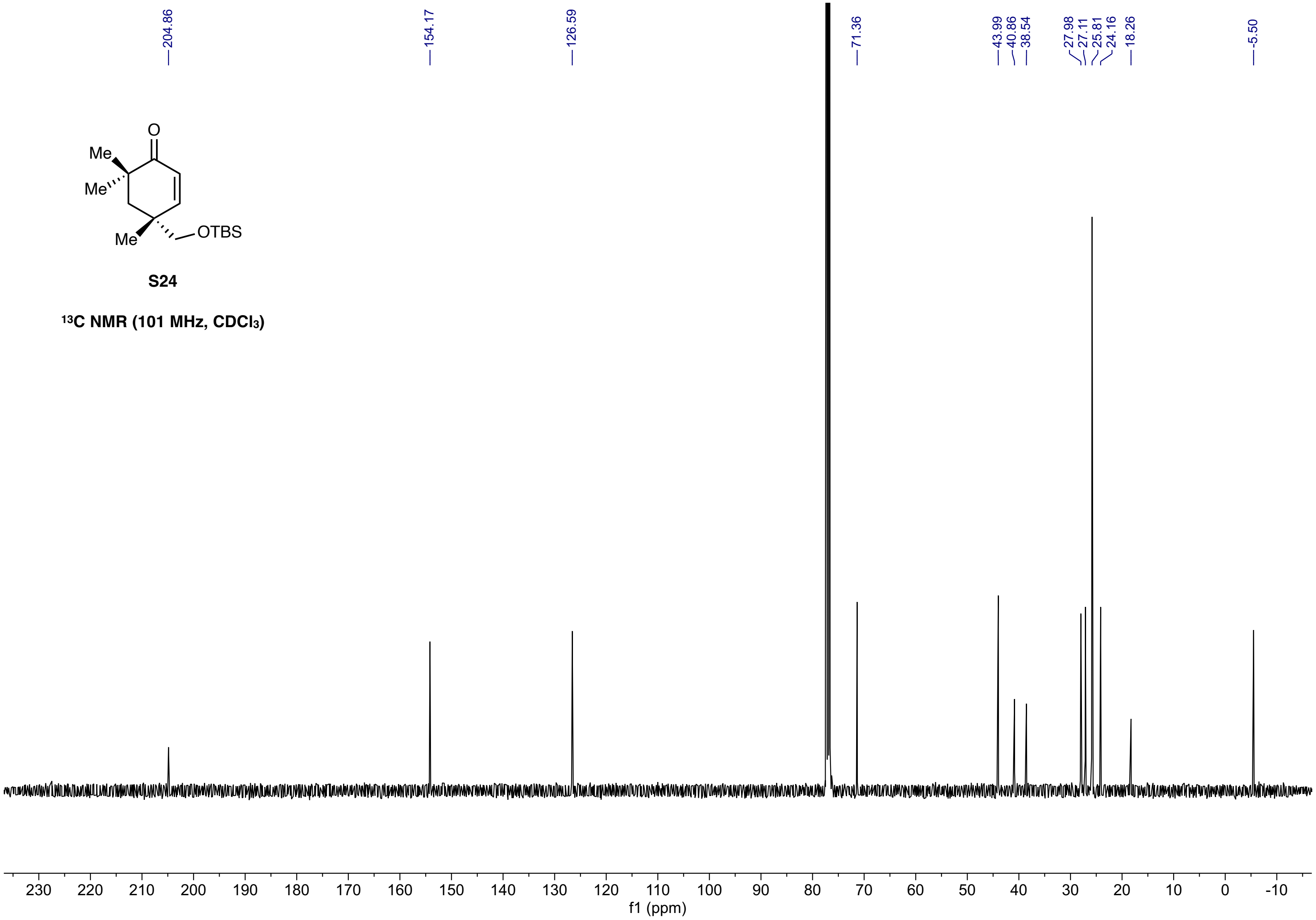

S24

^{13}C NMR (101 MHz, CDCl$_3$)

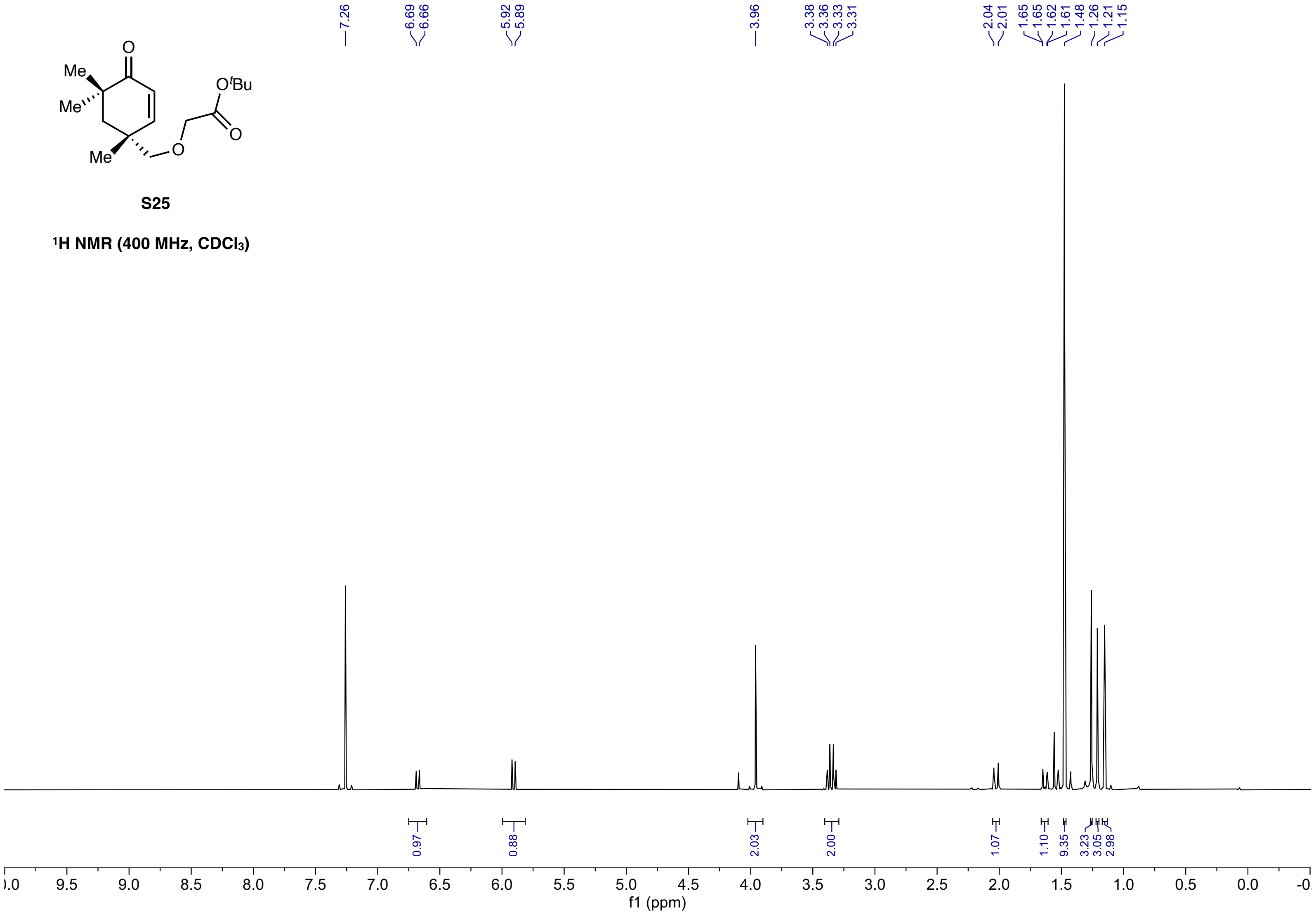
S25
¹H NMR (400 MHz, CDCl₃)
7.26
6.69
6.66
5.92
5.89
3.96
3.38
3.36
3.33
3.31
2.04
2.01
1.65
1.65
1.62
1.61
1.48
1.26
1.21
1.15
f1 (ppm)

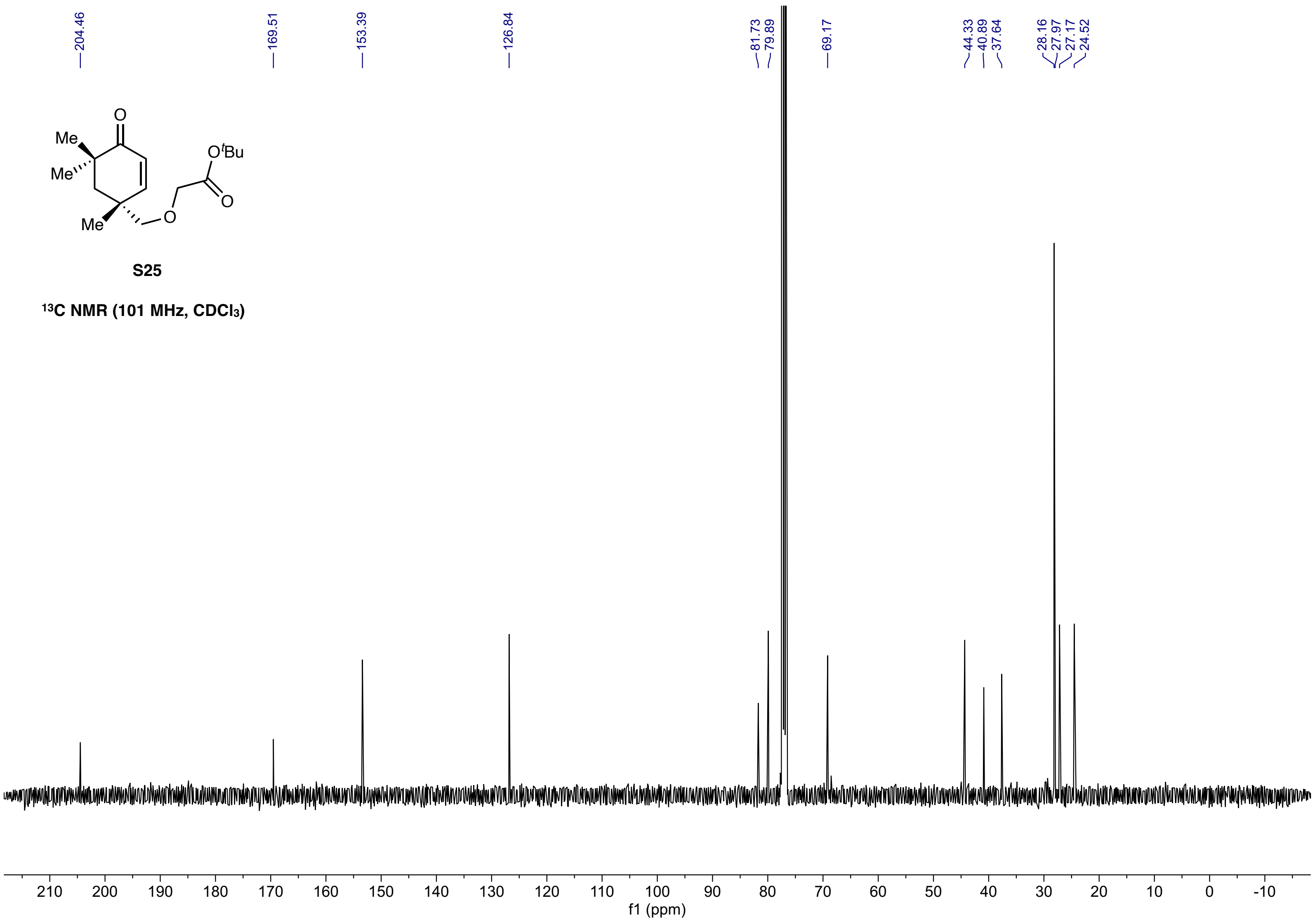
204.46
169.51
153.39
126.84
81.73
79.89
69.17
44.33
40.89
37.64
28.16
27.97
27.17
24.52
Me
Me
Me
O
O^tBu
O
O
S25
13C NMR (101 MHz, CDCl3)
210
200
190
180
170
160
150
140
130
120
110
100
90
80
70
60
50
40
30
20
10
0
-10
f1 (ppm)

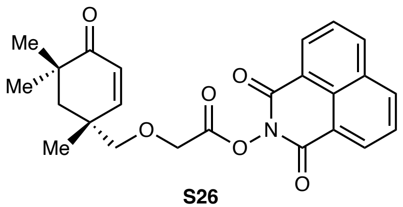

S26

^{1}H NMR (400 MHz, CDCl₃)

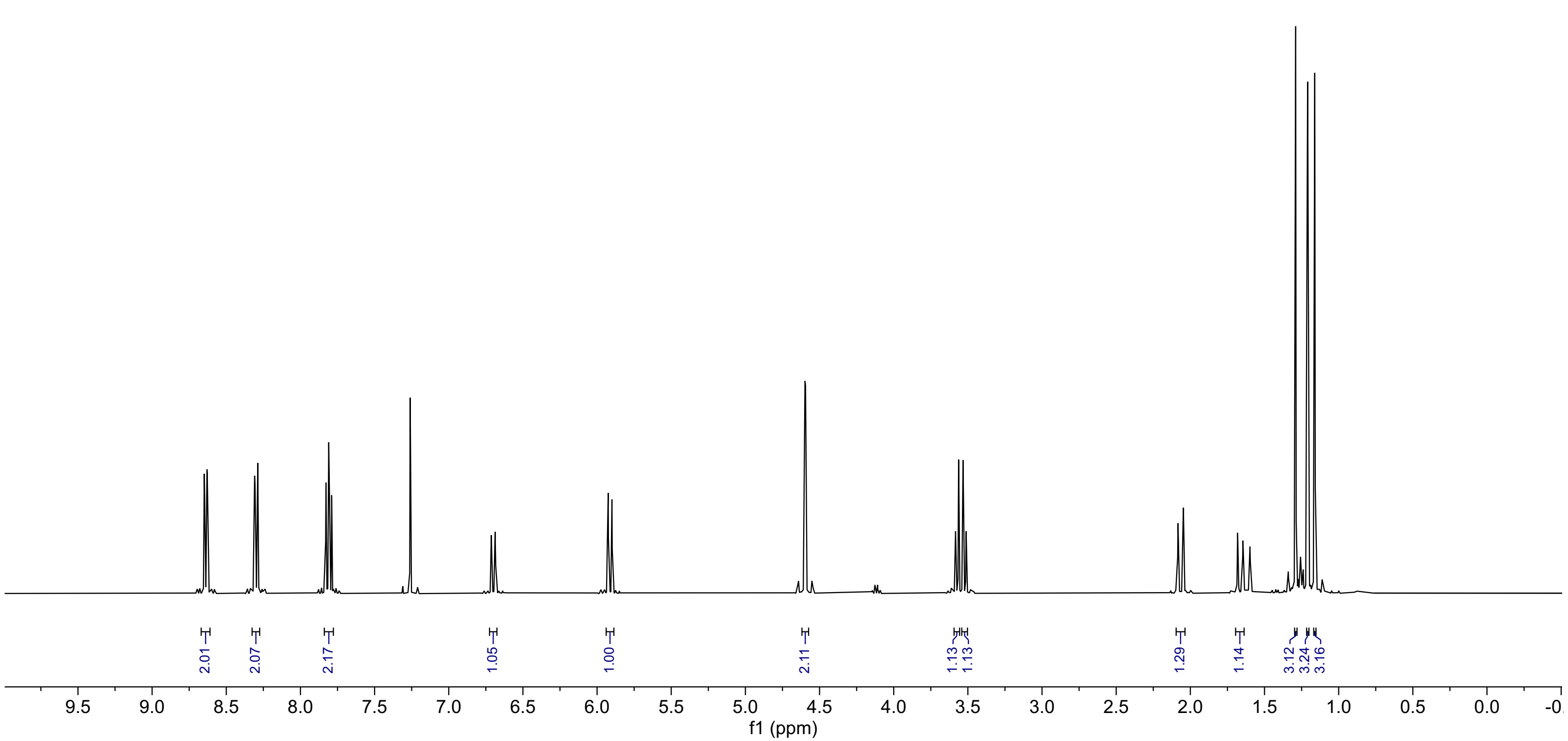

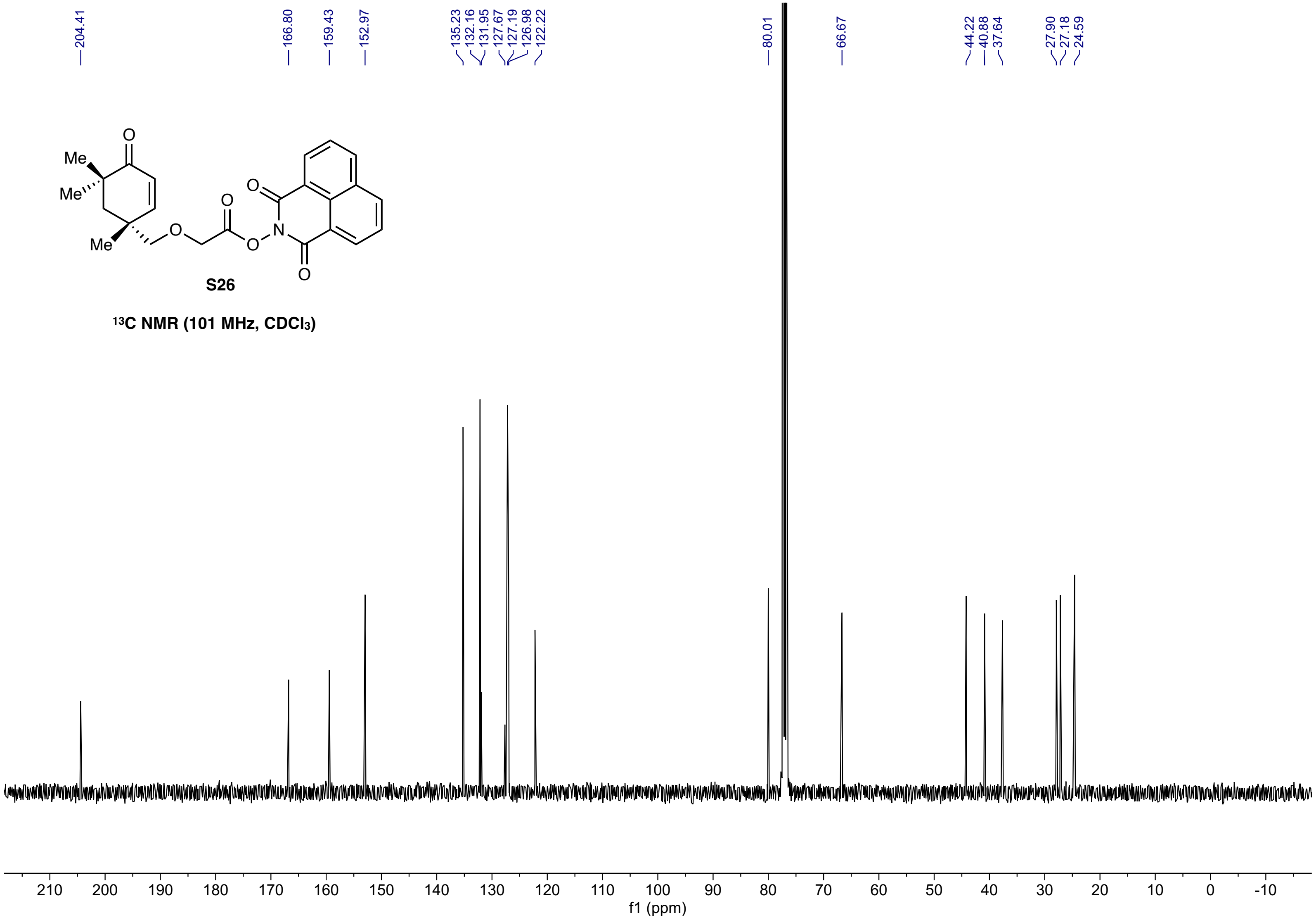
204.41
166.80
159.43
152.97
135.23
132.16
131.95
127.67
127.19
126.98
122.22
80.01
66.67
44.22
40.88
37.64
27.90
27.18
24.59
Me
Me
Me
O
O
O
O
N
O
O
S26
13C NMR (101 MHz, CDCl3)
210 200 190 180 170 160 150 140 130 120 110 100 90 80 70 60 50 40 30 20 10 0 -10
f1 (ppm)

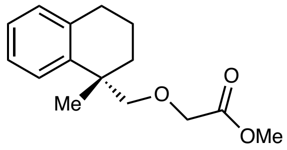

S29

^{1}H NMR (400 MHz, $CDCl_3$)

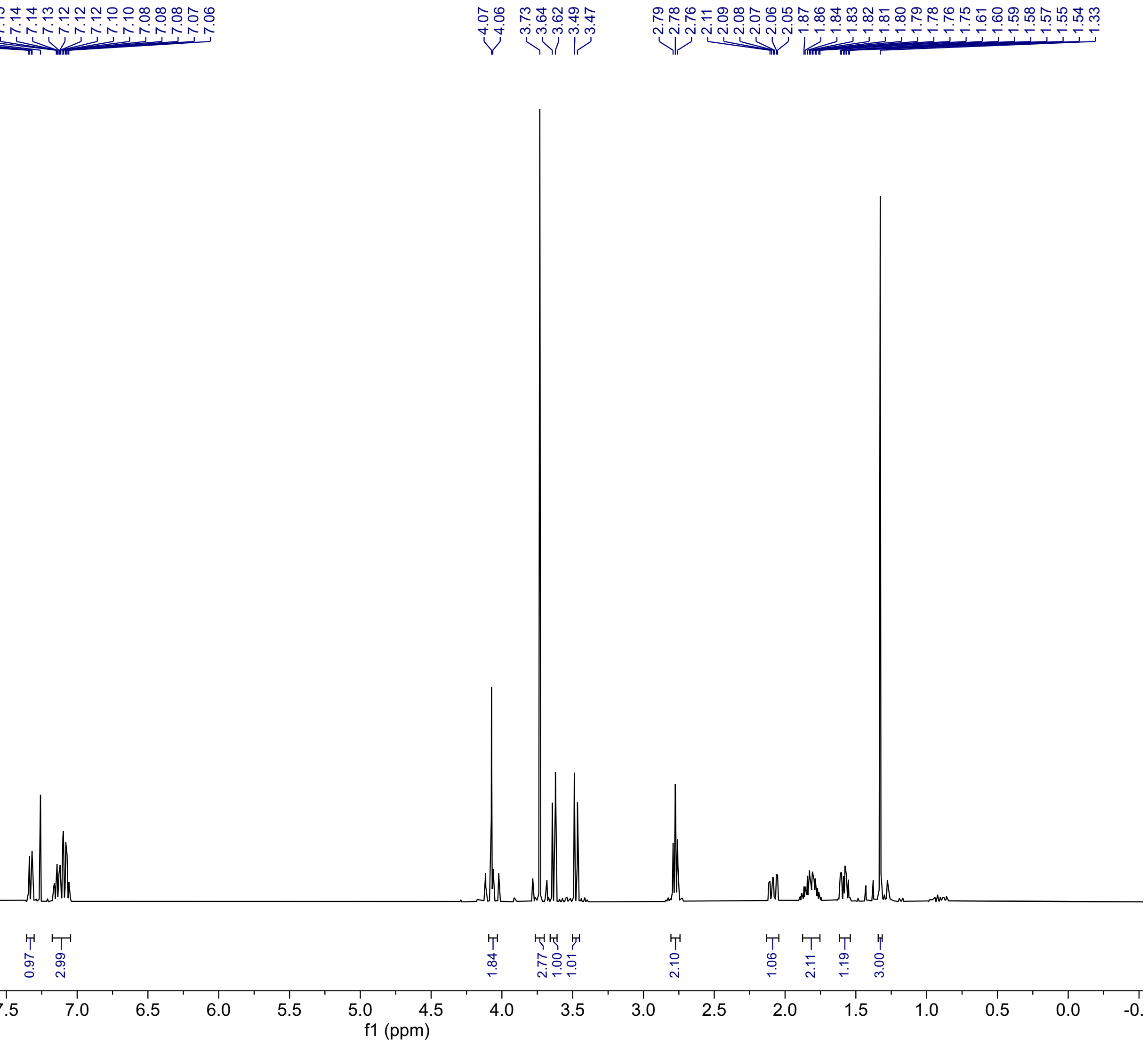

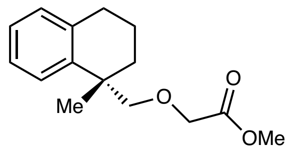

S29

^{13}C NMR (101 MHz, CDCl$_3$)

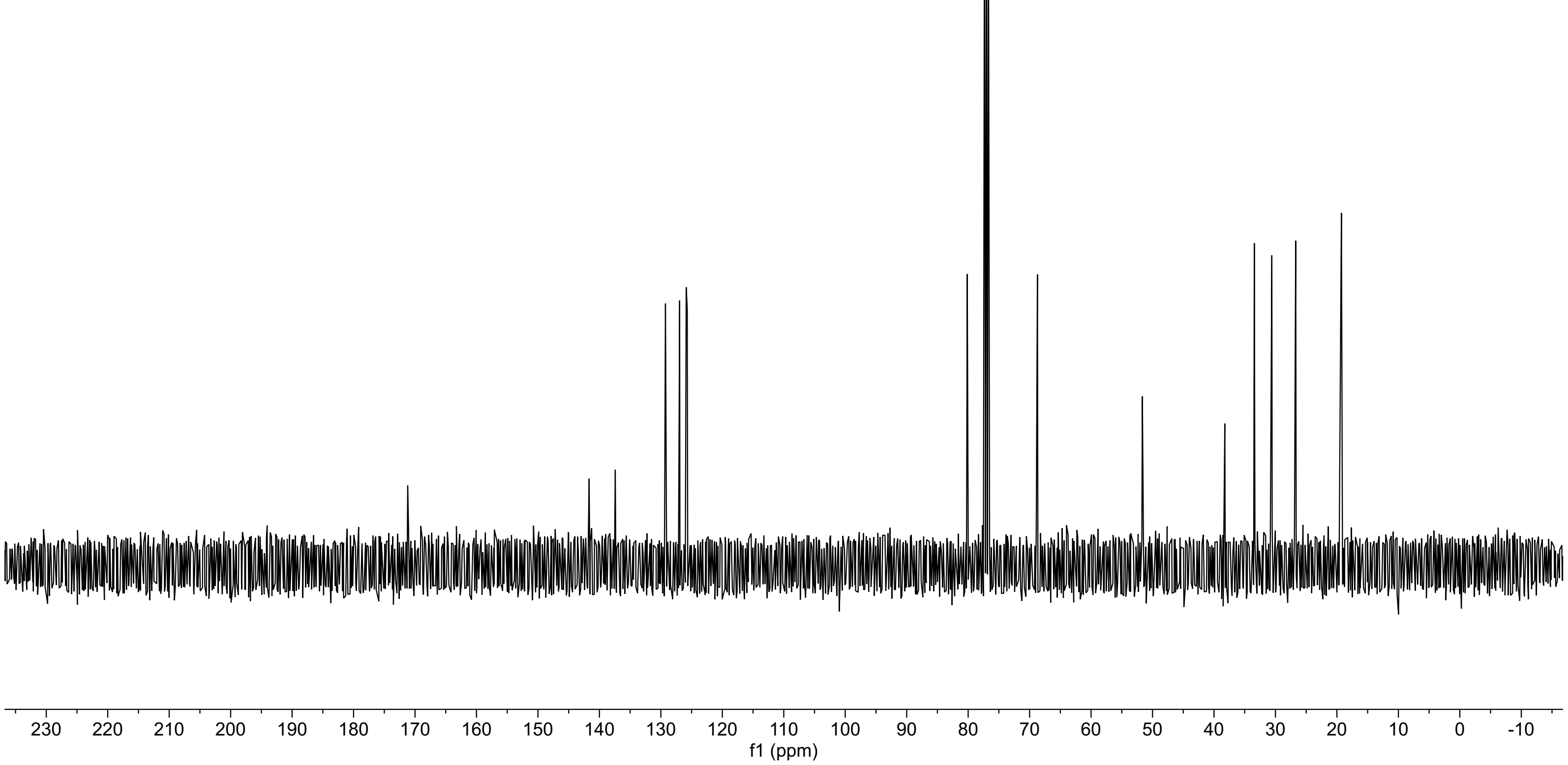

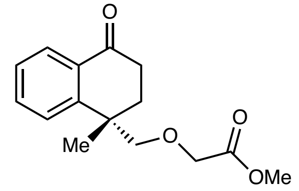

S30

^{1}H NMR (400 MHz, $CDCl_3$)

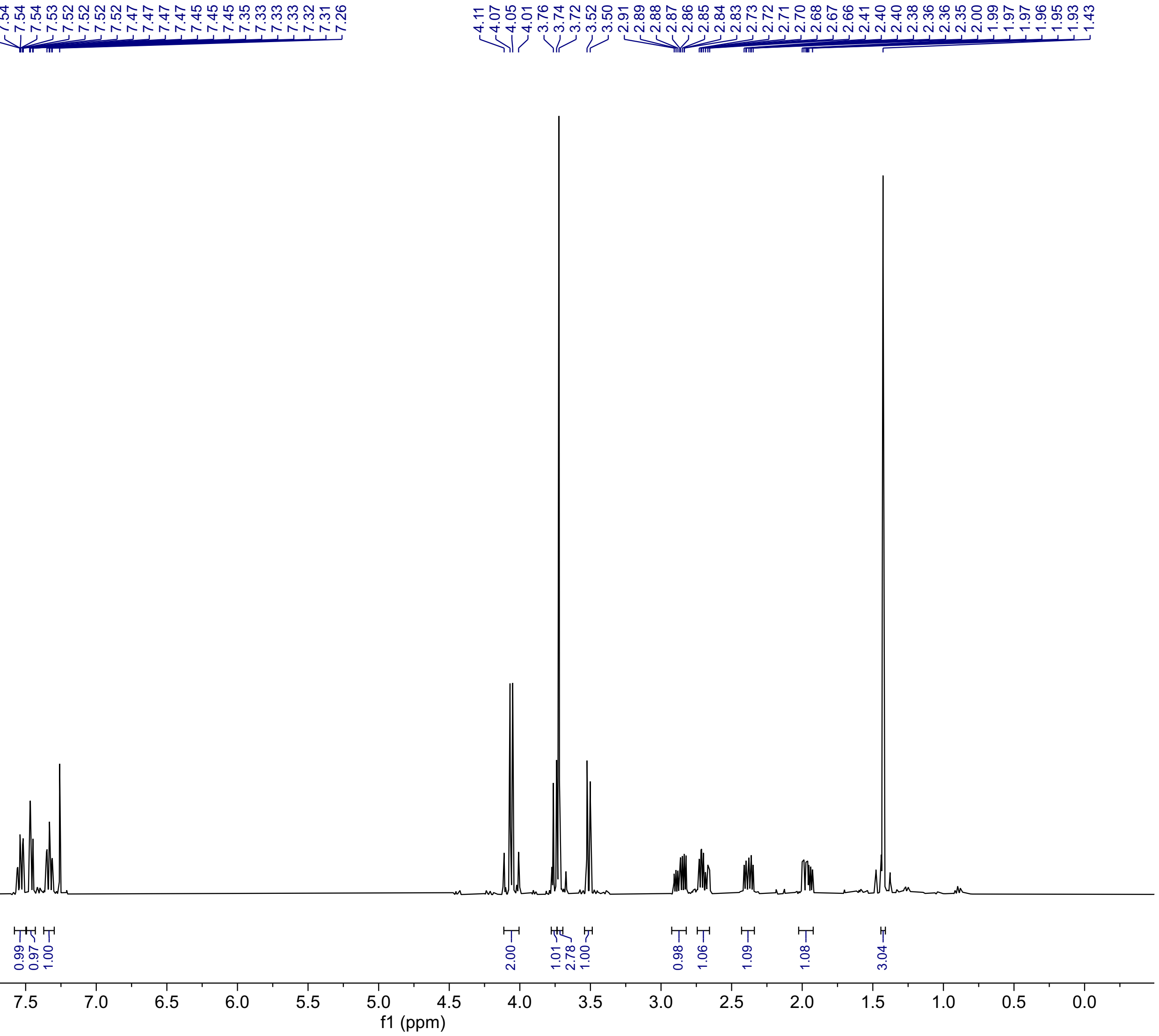

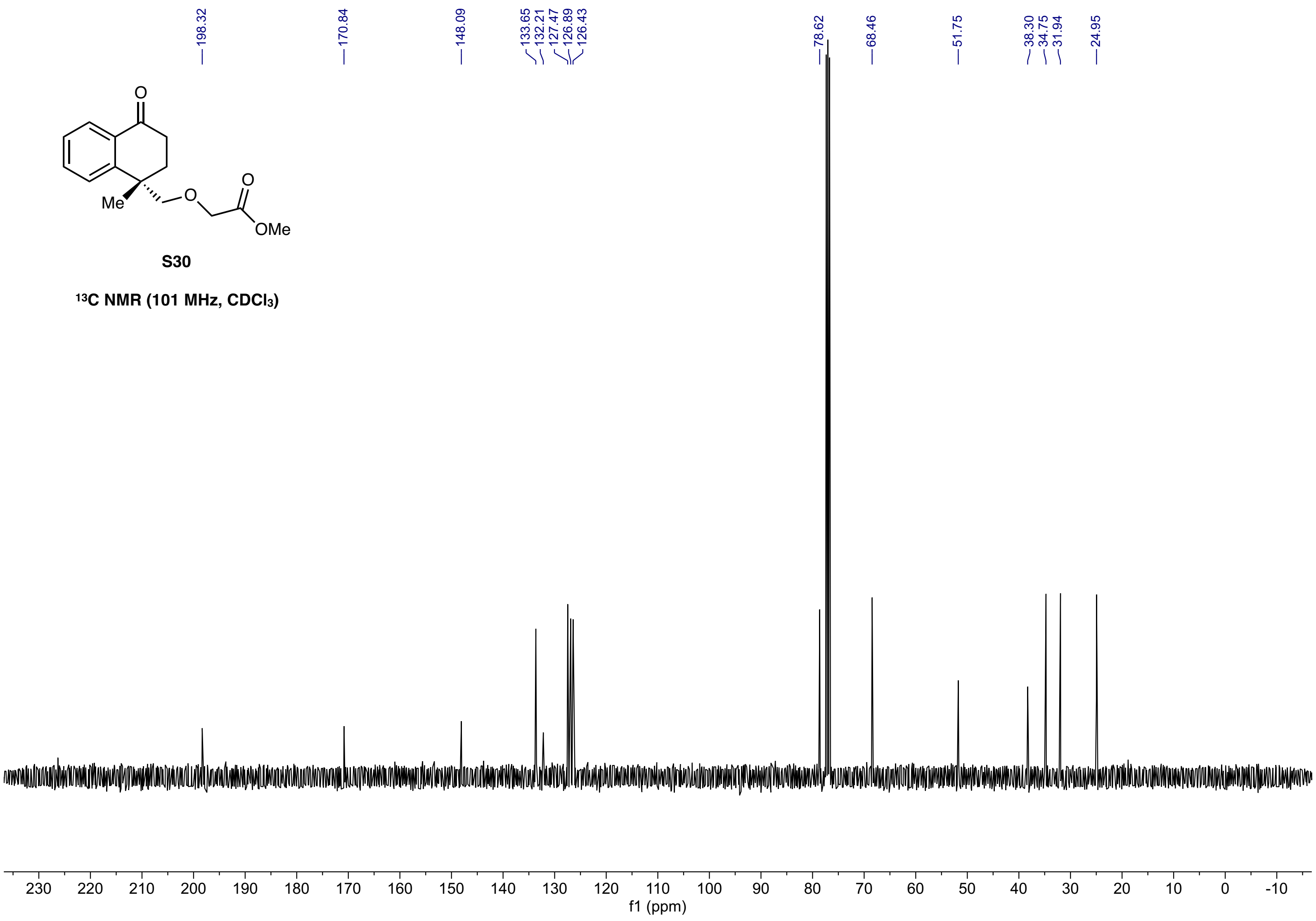

S30

^{13}C NMR (101 MHz, $CDCl_3$)

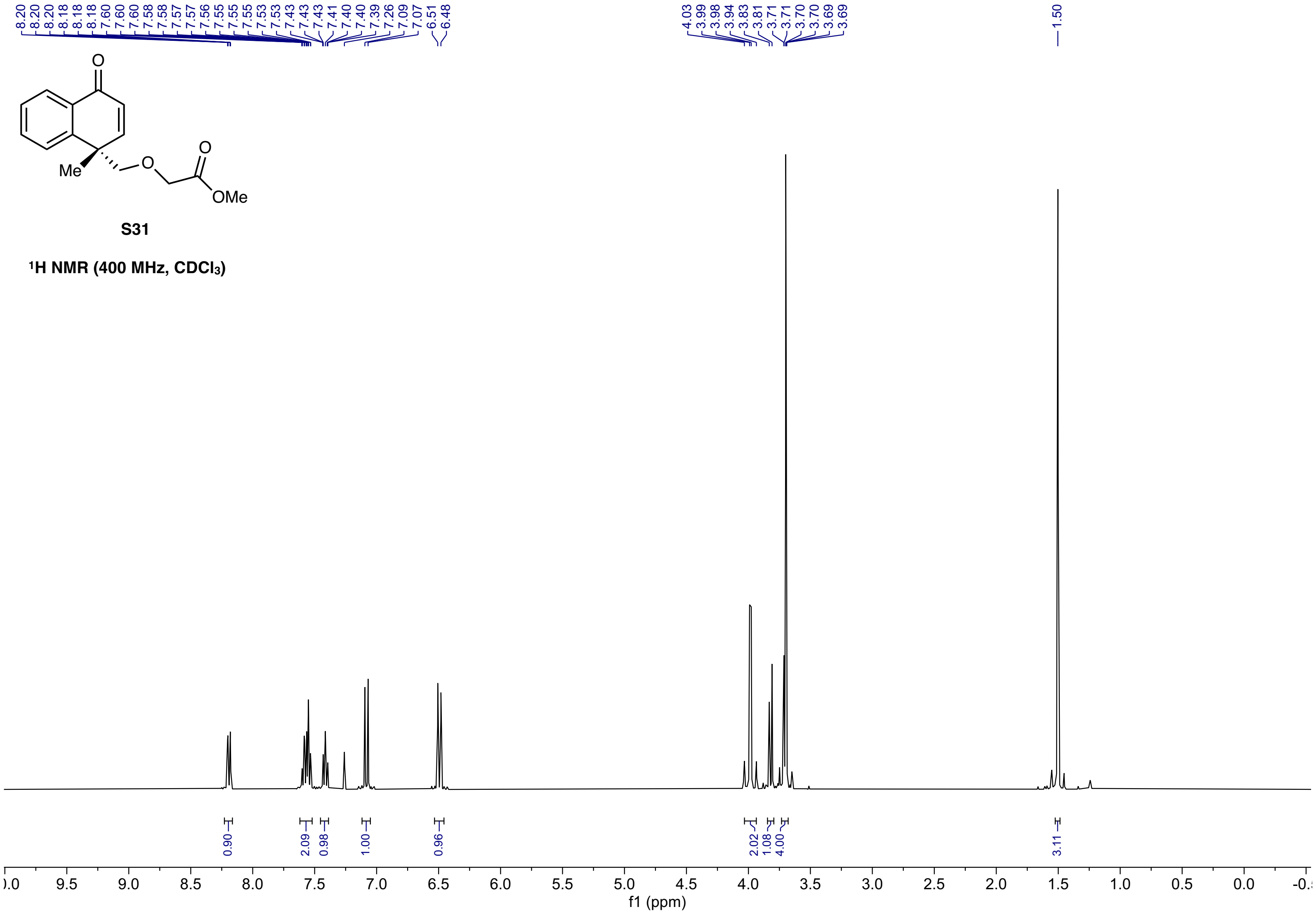
S31
^1H NMR (400 MHz, $CDCl_3$)
f1 (ppm)

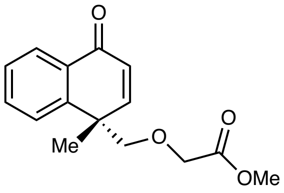

S31

^{13}C NMR (101 MHz, $CDCl_3$)

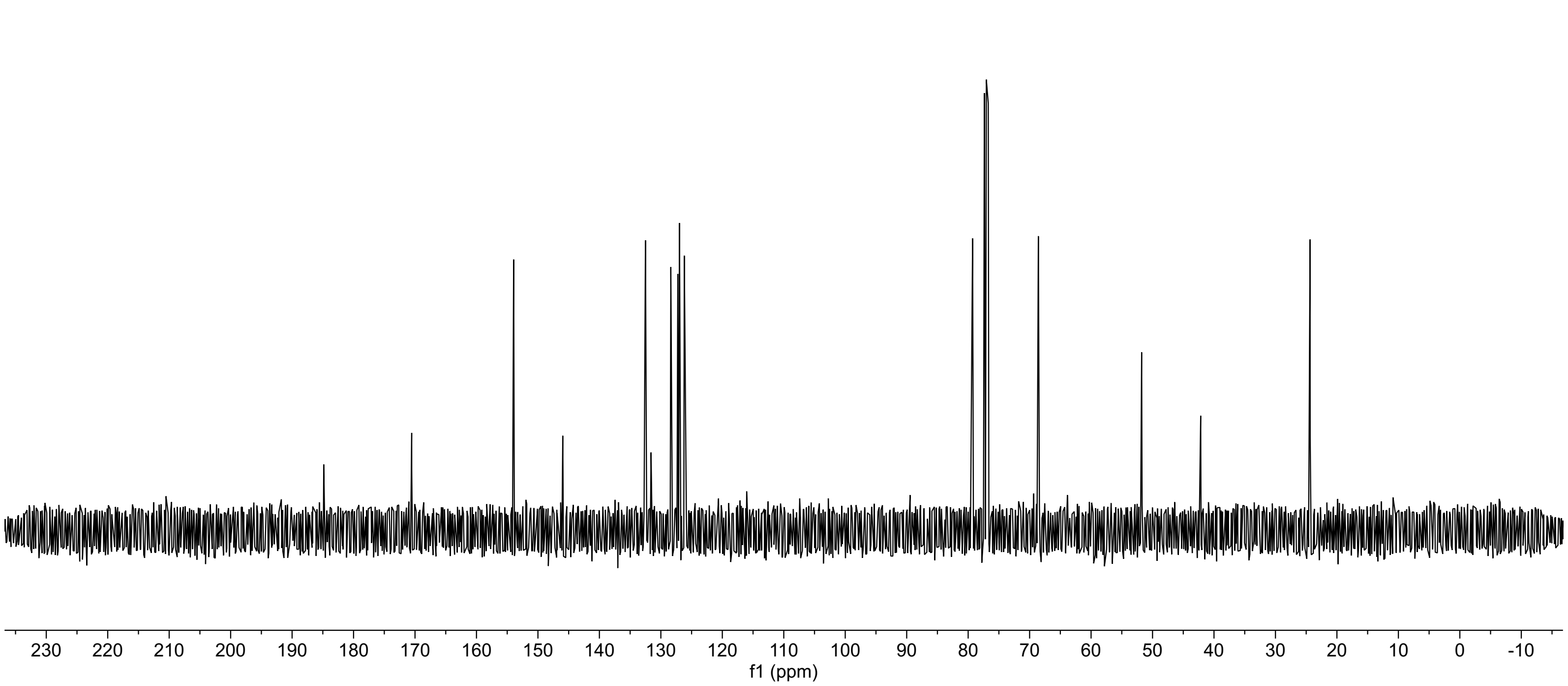

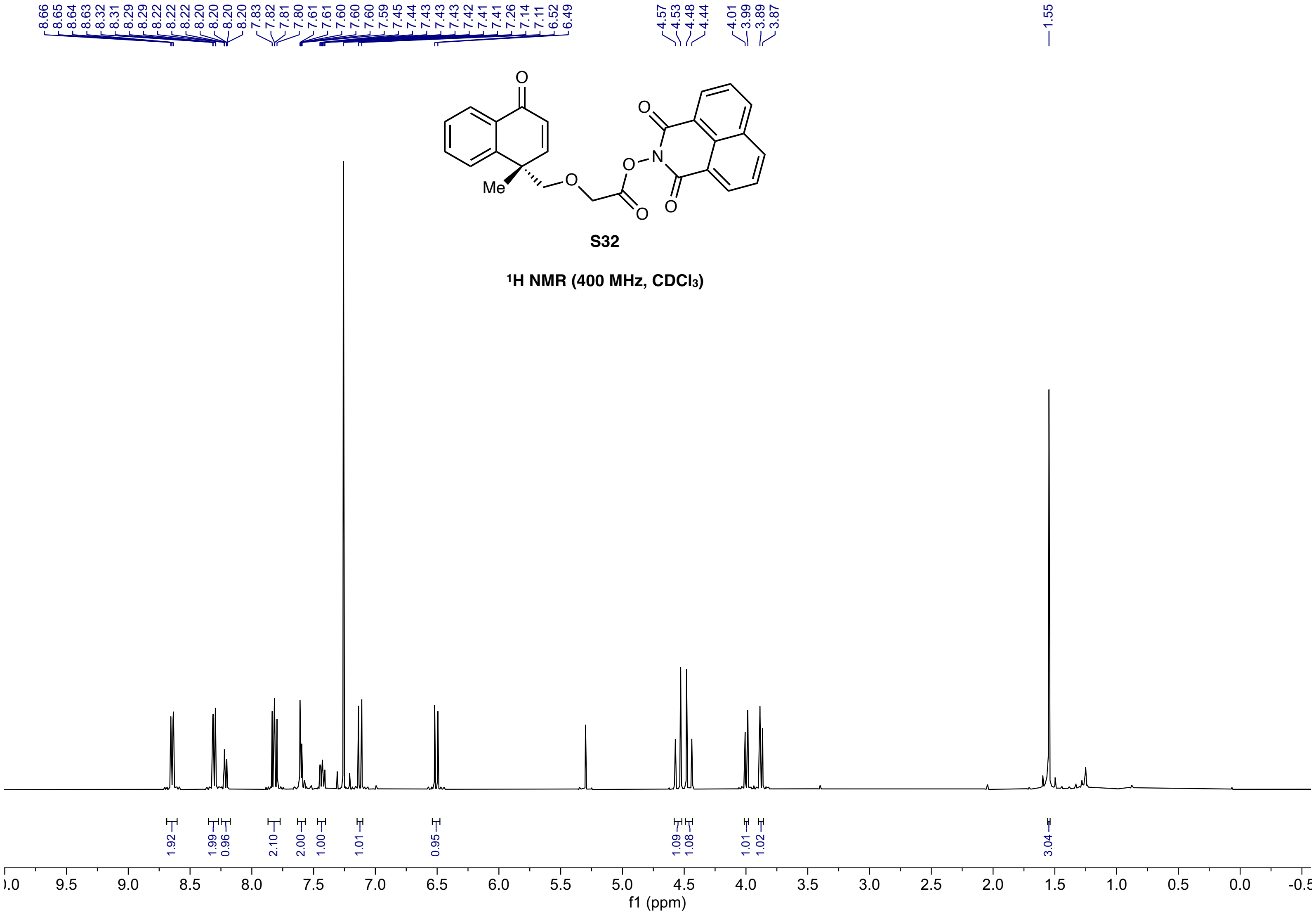

8.66 8.65 8.64 8.63 8.32 8.31 8.29 8.29 8.22 8.22 8.22 8.20 8.20 8.20 7.83 7.82 7.81 7.80 7.61 7.61 7.60 7.60 7.60 7.59 7.45 7.44 7.43 7.43 7.43 7.42 7.41 7.41 7.26 7.14 7.11 6.52 6.49
4.57 4.53 4.48 4.44 4.01 3.99 3.89 3.87
1.55
Me
S32
¹H NMR (400 MHz, CDCl₃)
1.92 1.99 0.96 2.10 2.00 1.00 1.01 0.95 1.09 1.08 1.01 1.02 3.04
f1 (ppm)

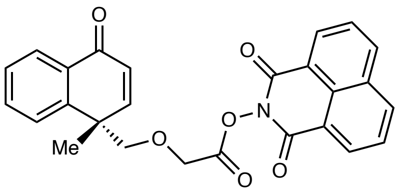

S32

^{13}C NMR (101 MHz, CDCl$_3$)

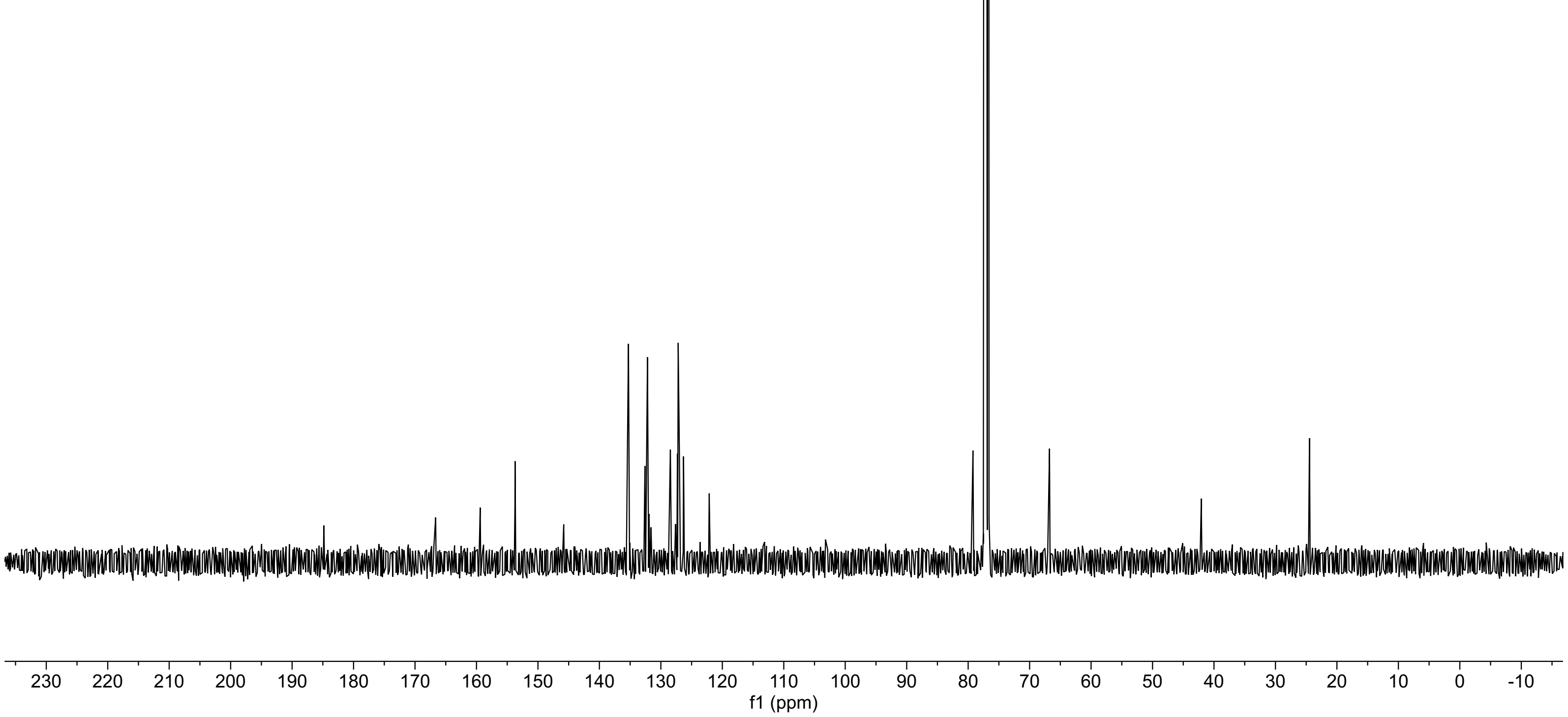

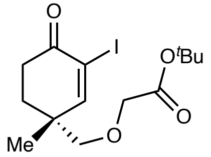

S33

^{1}H NMR (400 MHz, $CDCl_3$)

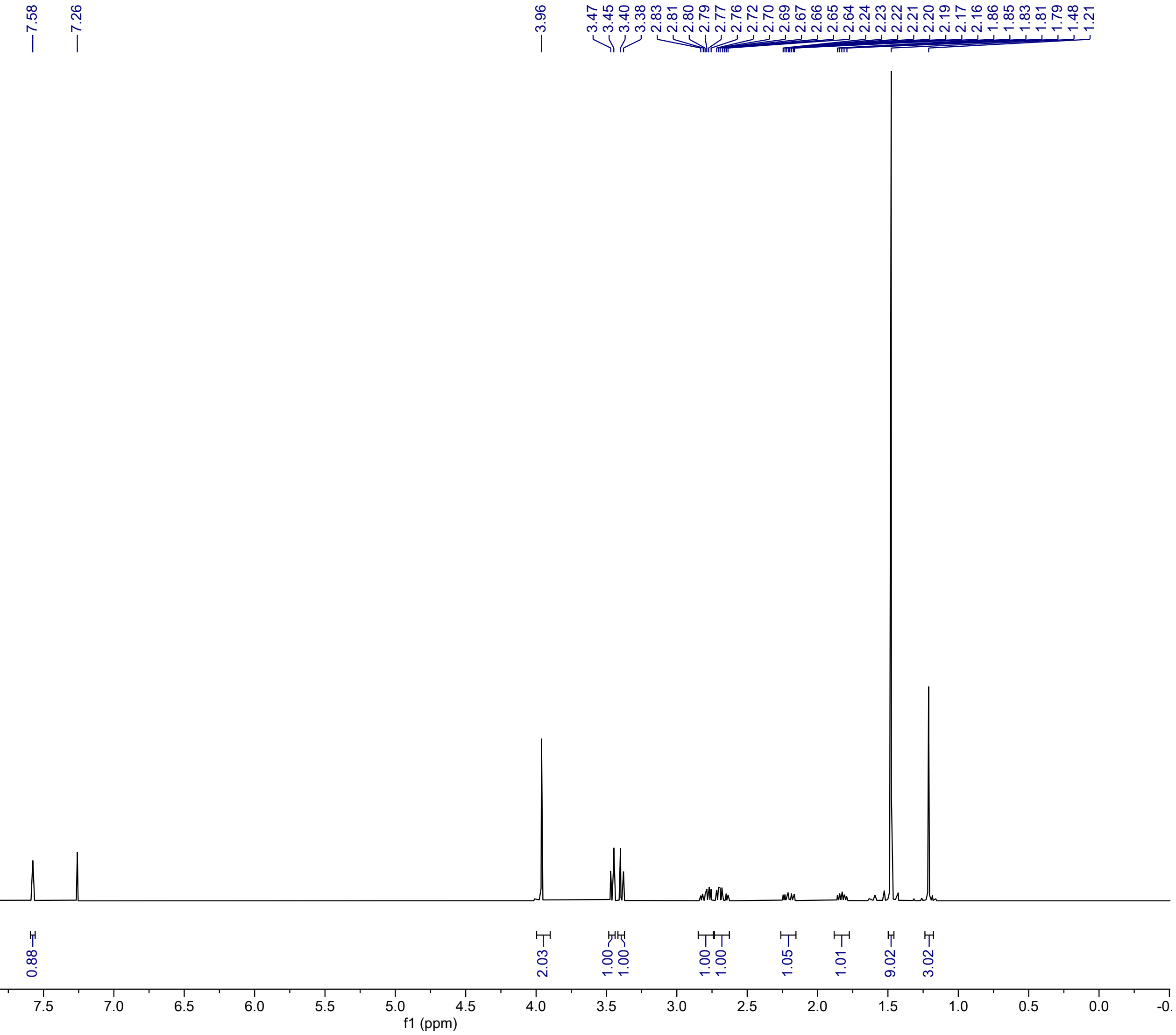

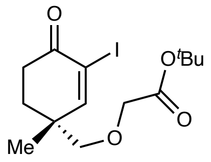

S33

^{13}C NMR (101 MHz, $CDCl_3$)

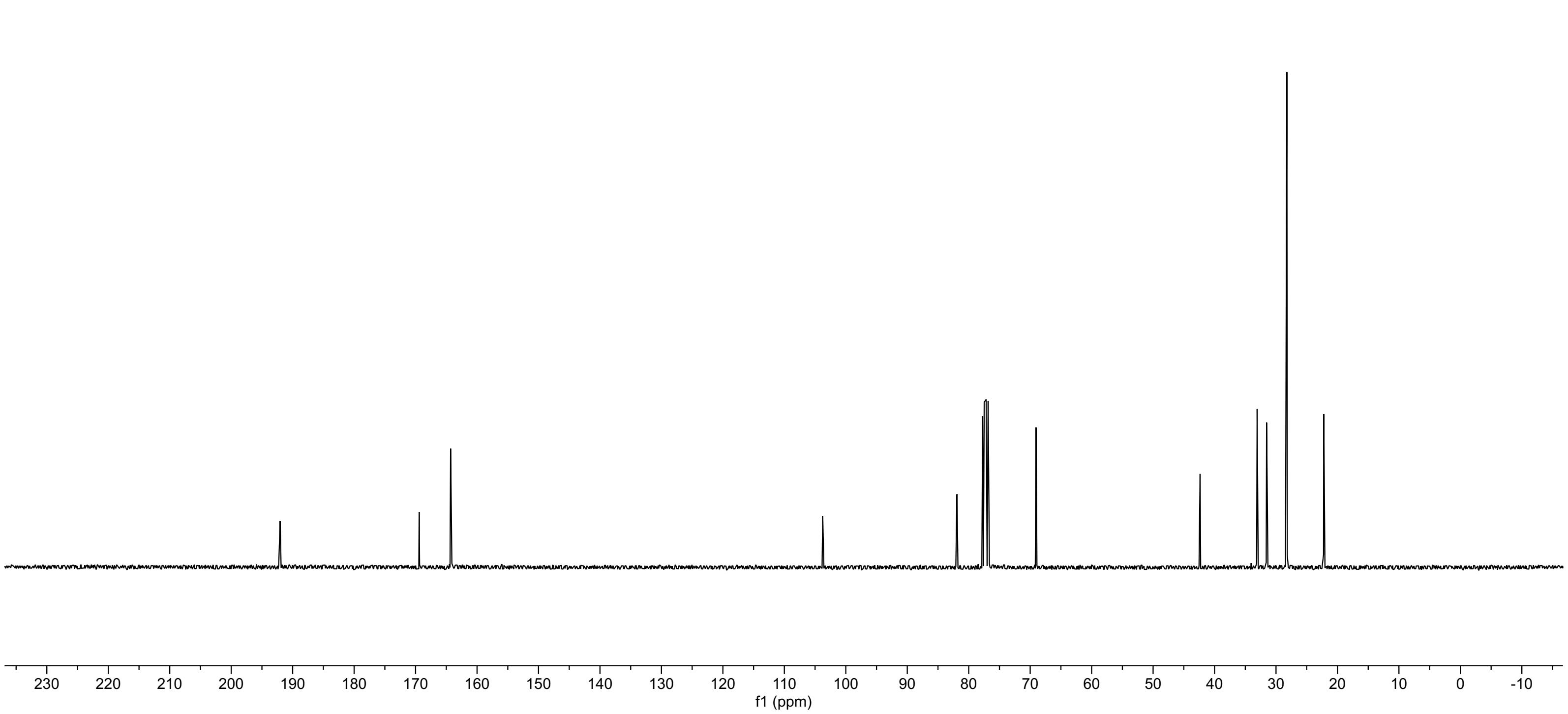

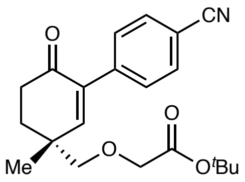

S34

^{1}H NMR (400 MHz, $CDCl_3$)

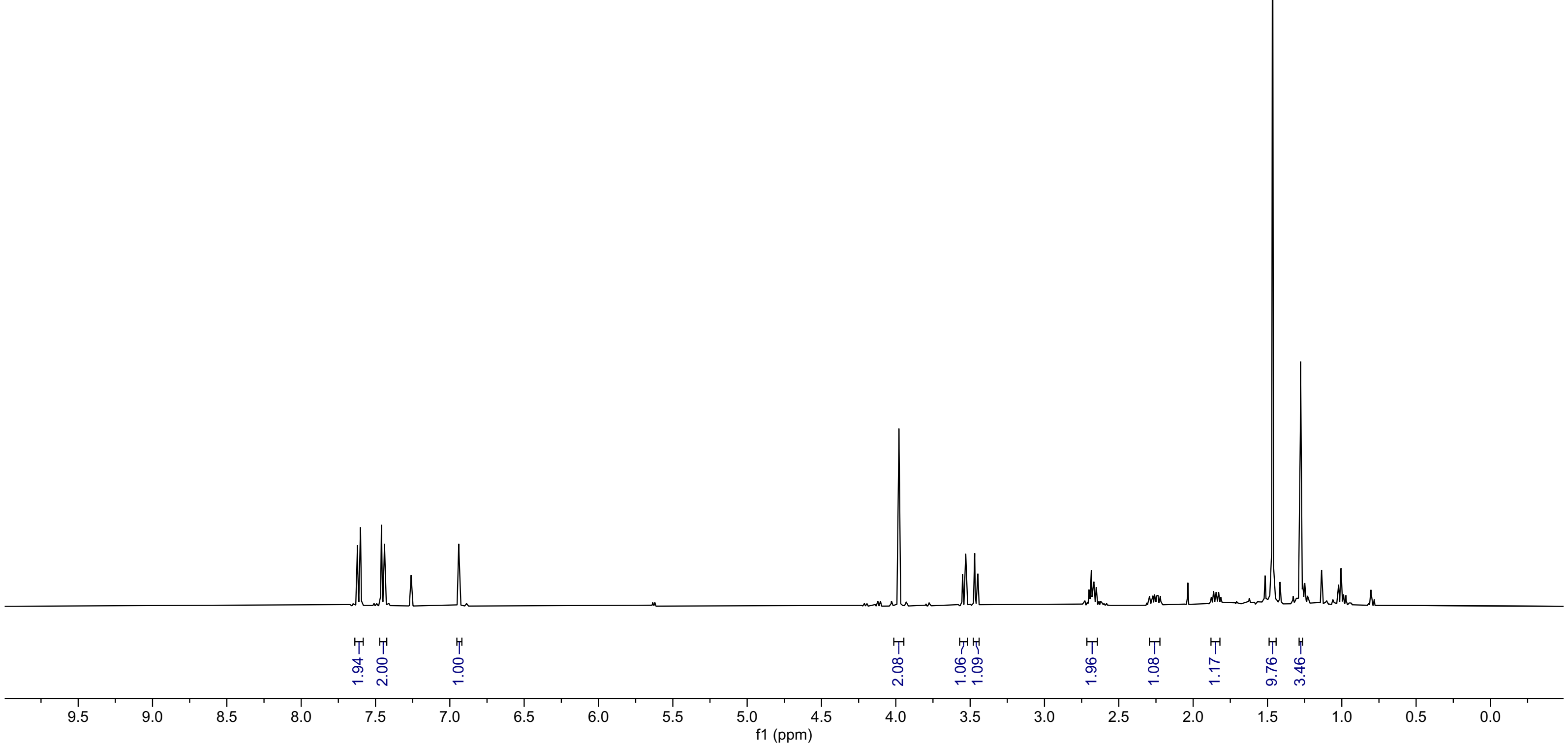

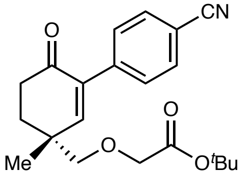

S34

^{13}C NMR (101 MHz, CDCl$_3$)

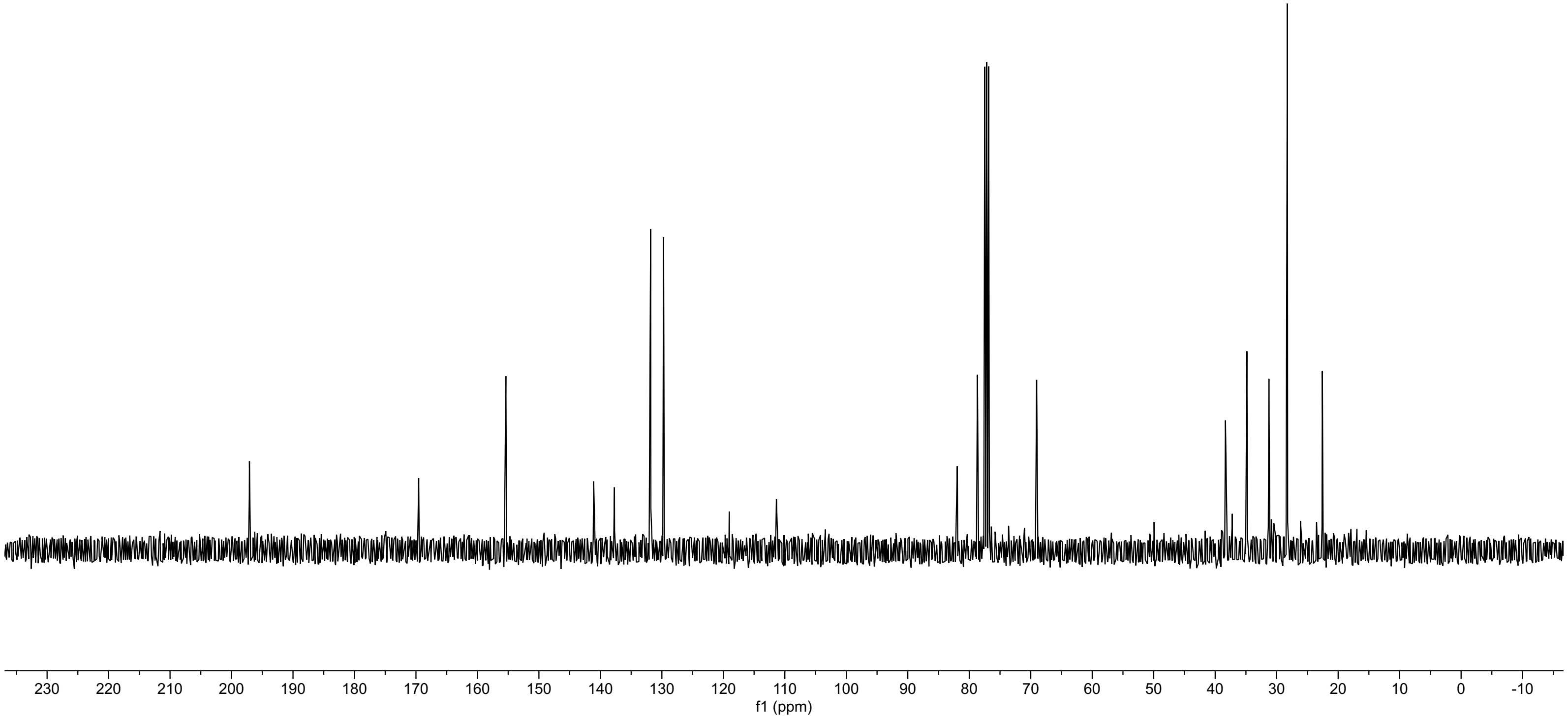

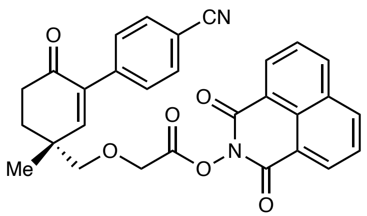

S35

^{1}H NMR (400 MHz, $CDCl_3$)

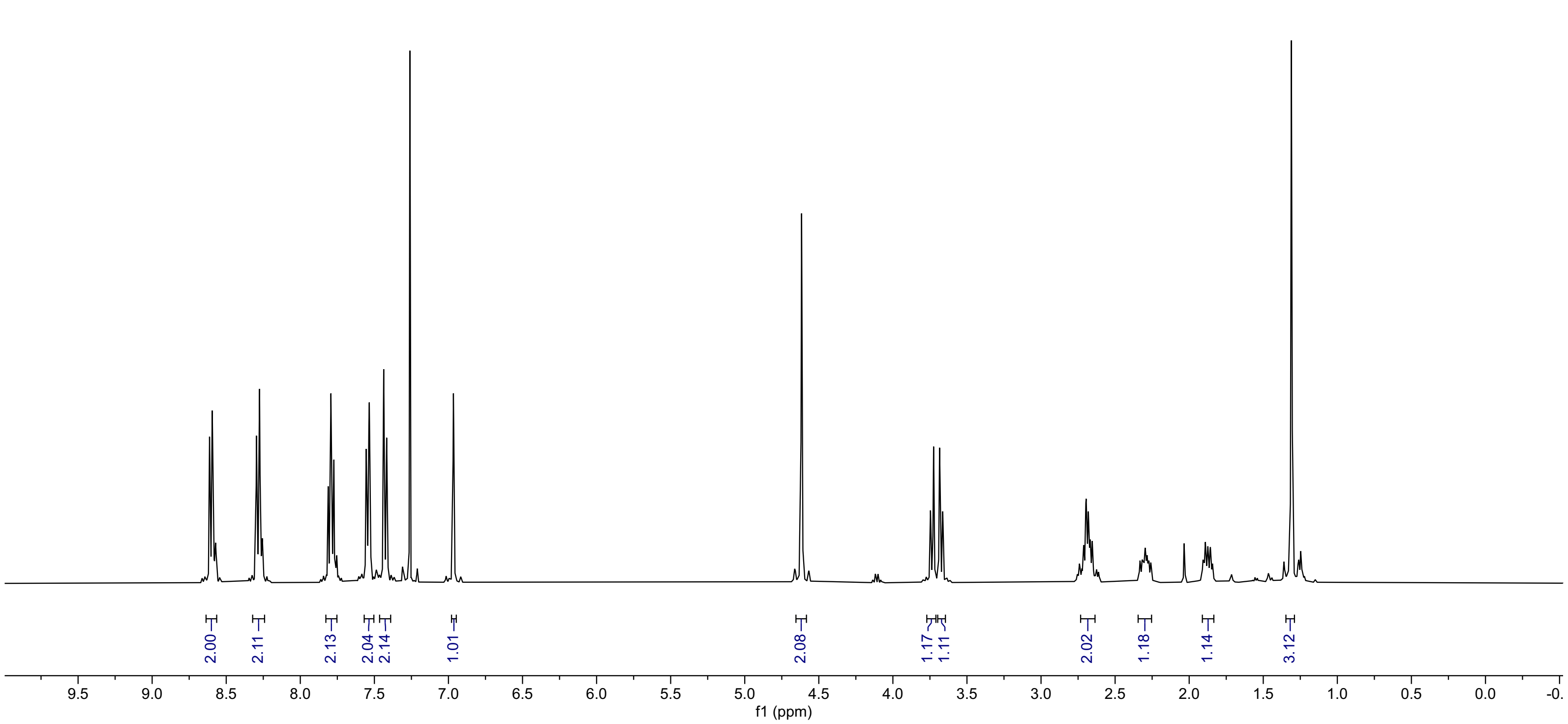

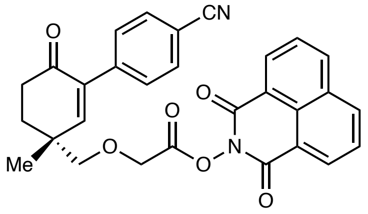

S35

^{13}C NMR (101 MHz, $CDCl_3$)

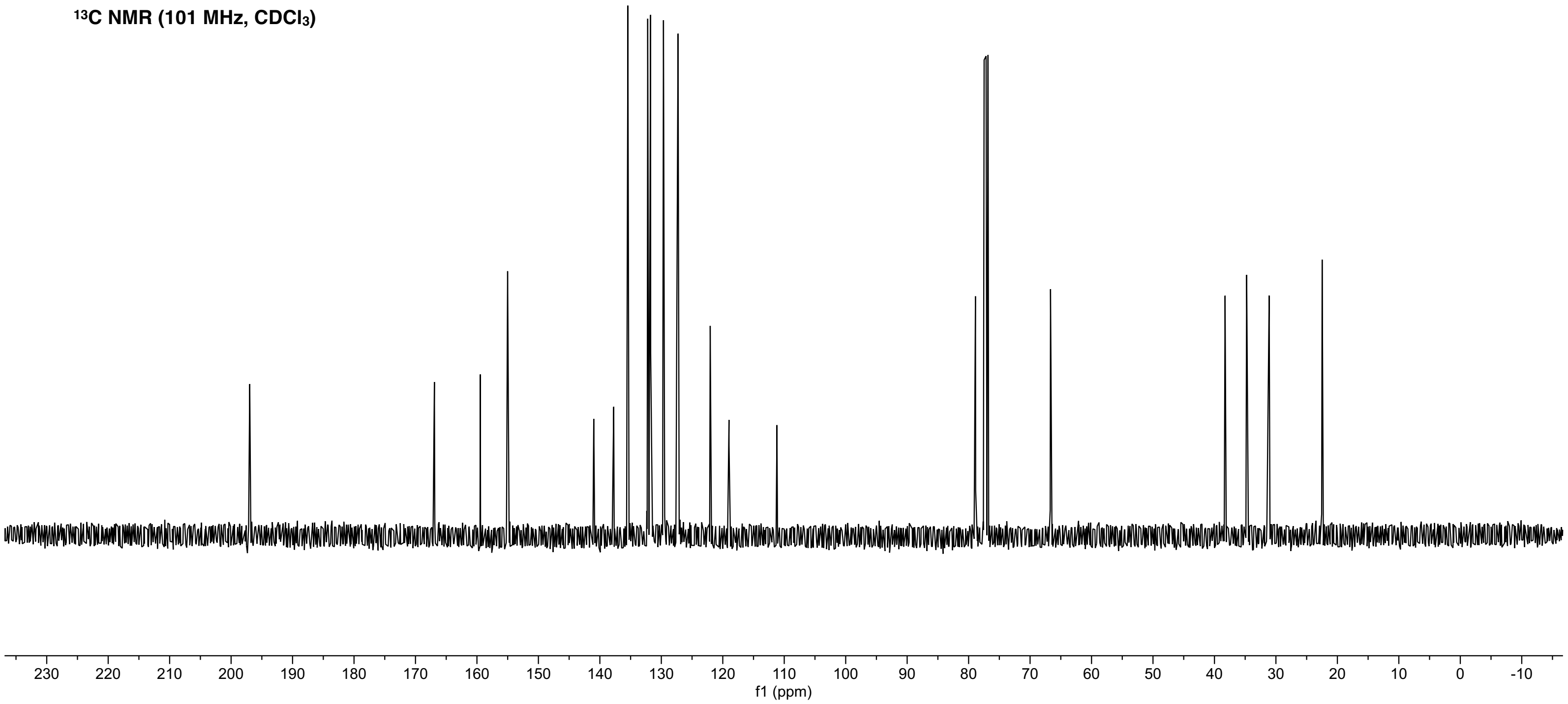

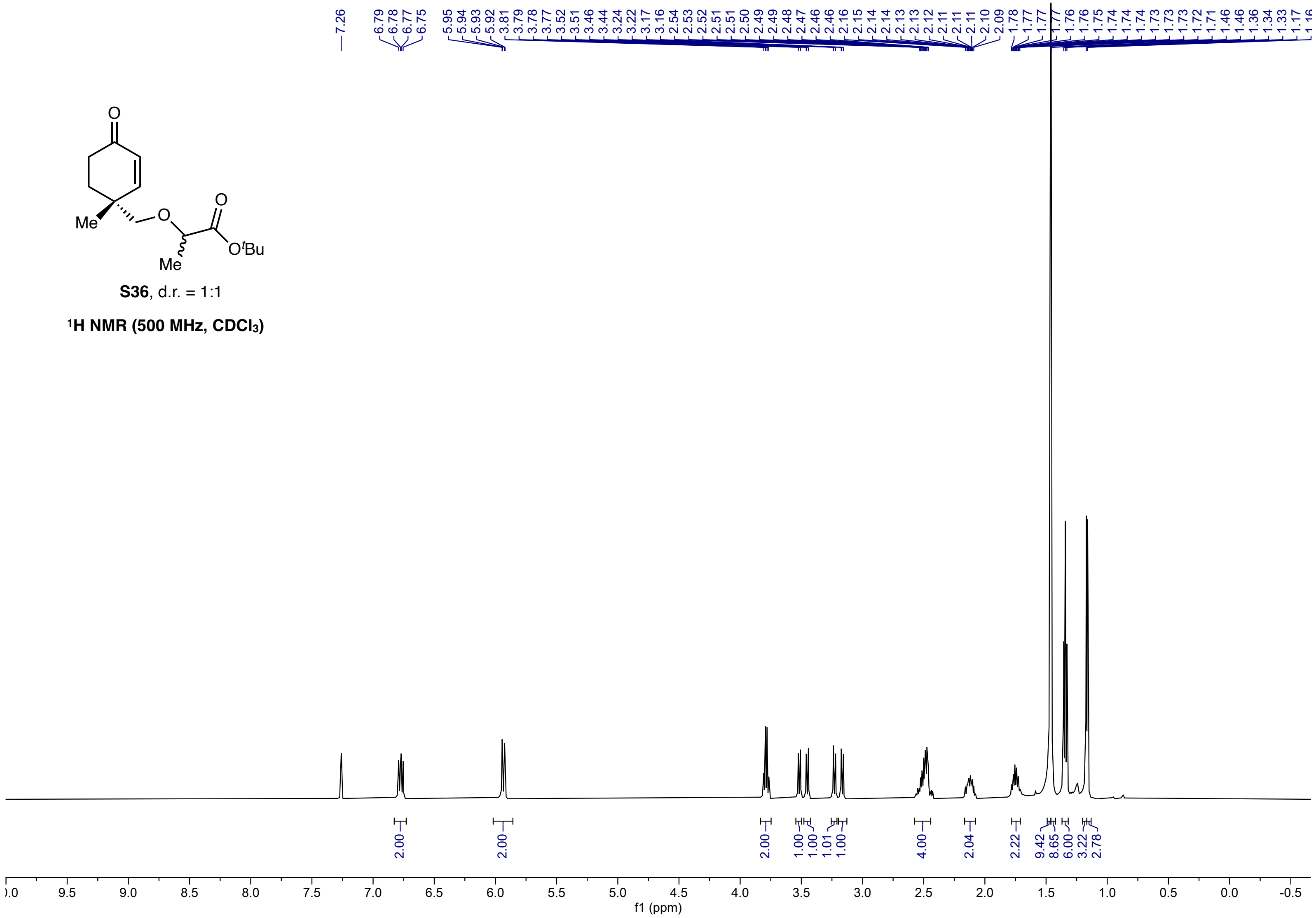

S36, d.r. = 1:1

^{1}H NMR (500 MHz, CDCl$_3$)

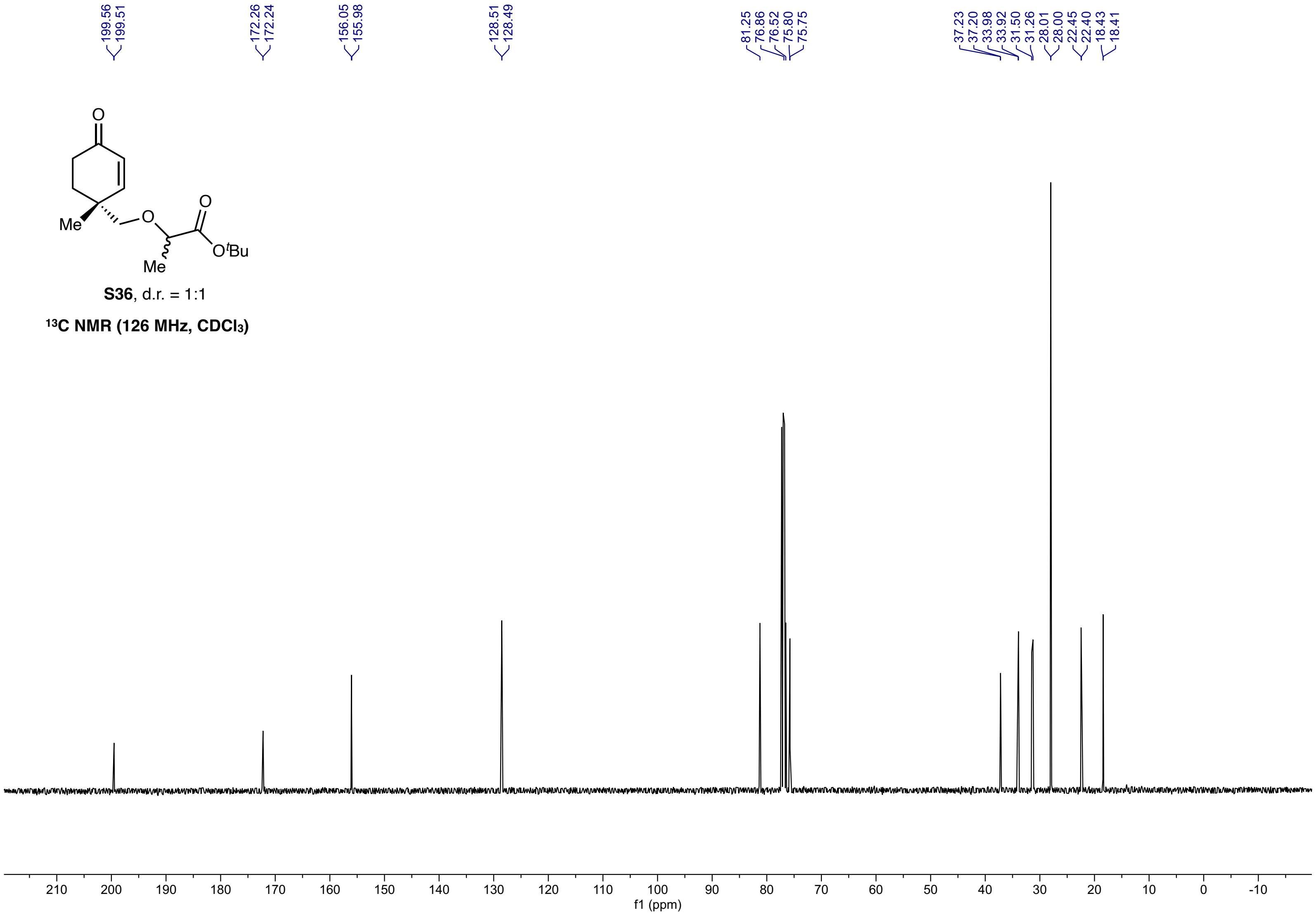

S36, d.r. = 1:1

^{13}C NMR (126 MHz, CDCl$_3$)

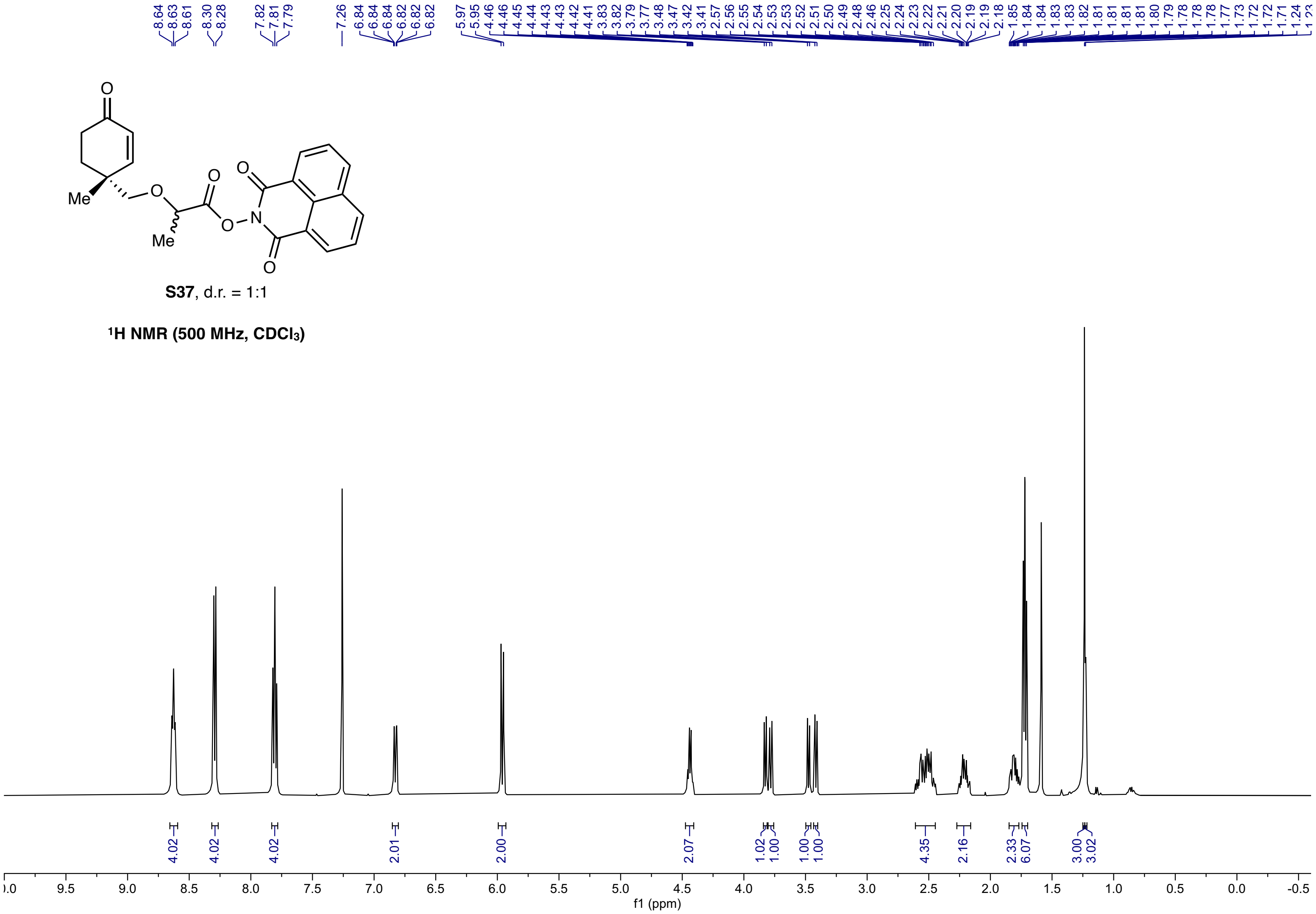
S37, d.r. = 1:1
¹H NMR (500 MHz, CDCl₃)
f1 (ppm)

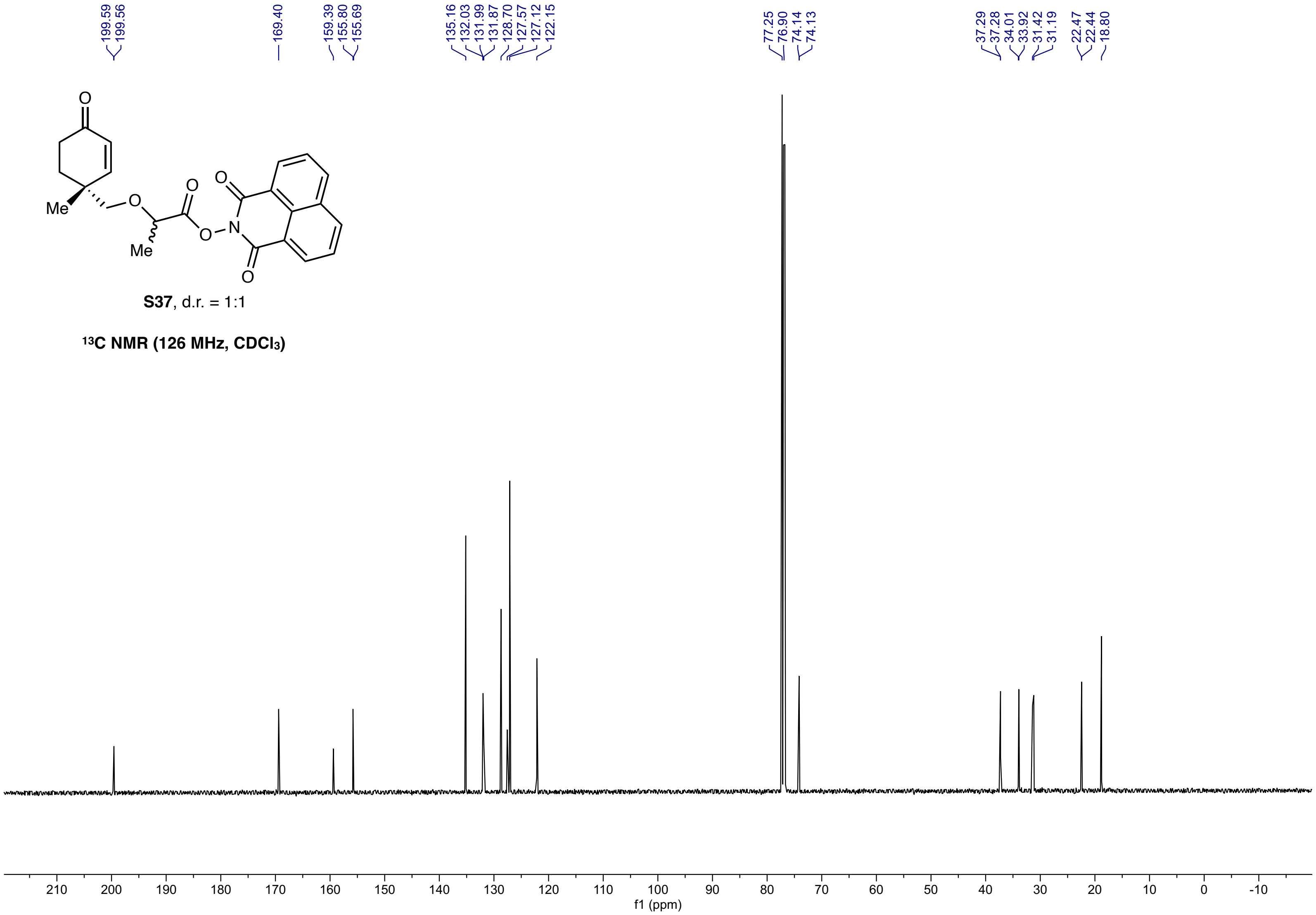

S37, d.r. = 1:1

^{13}C NMR (126 MHz, $CDCl_3$)

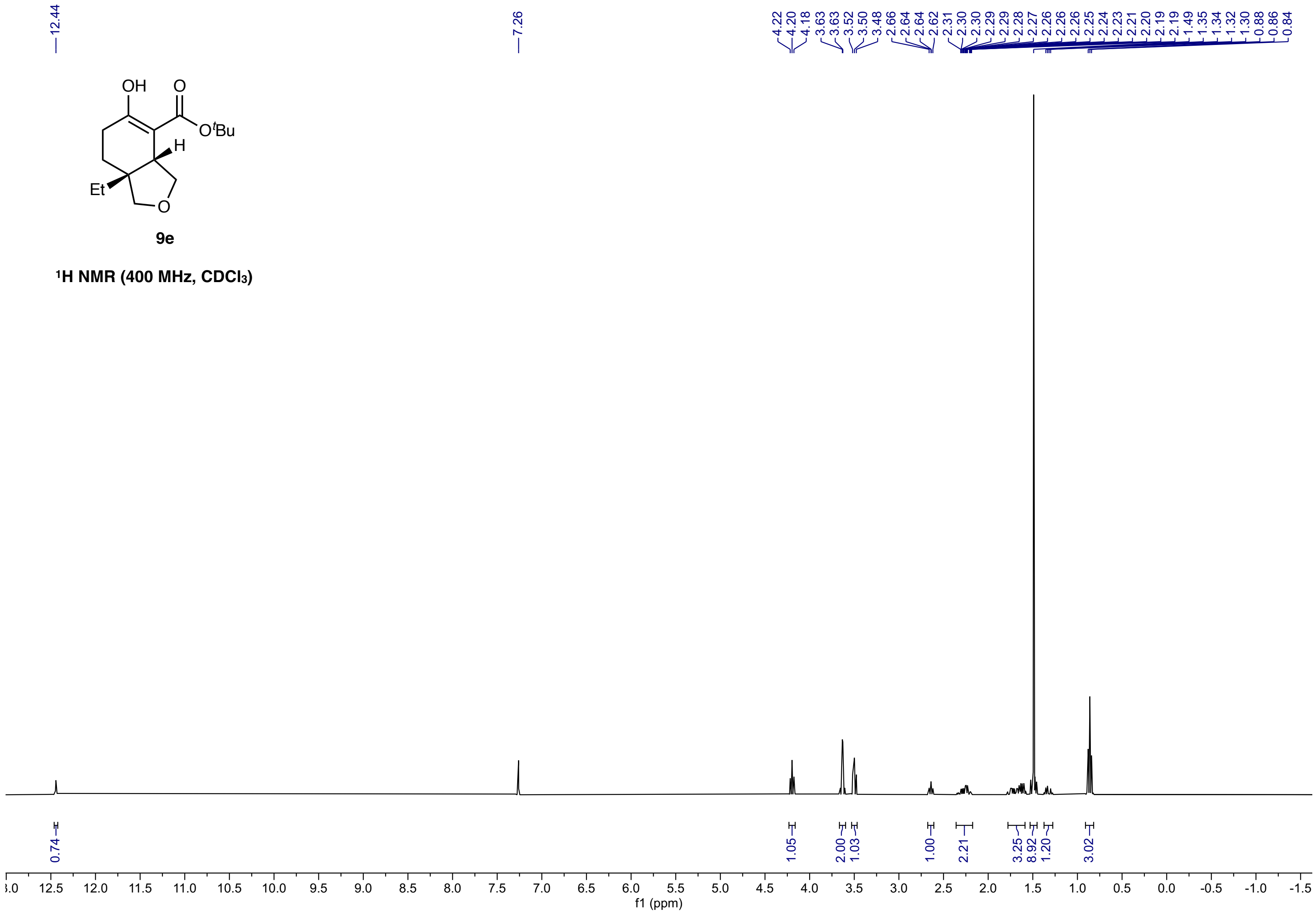

12.44
7.26
4.22
4.20
4.18
3.63
3.63
3.52
3.50
3.48
2.66
2.64
2.64
2.62
2.31
2.30
2.30
2.29
2.29
2.28
2.27
2.26
2.26
2.26
2.25
2.24
2.23
2.21
2.20
2.19
2.19
1.49
1.35
1.34
1.32
1.30
0.88
0.86
0.84
OH
O
O^tBu
H
Et
O
9e
1H NMR (400 MHz, CDCl3)
0.74
1.05
2.00
1.03
1.00
2.21
3.25
8.92
1.20
3.02
f1 (ppm)

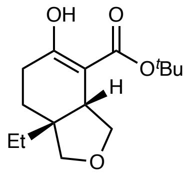

9e

^{13}C NMR (101 MHz, CDCl$_3$)

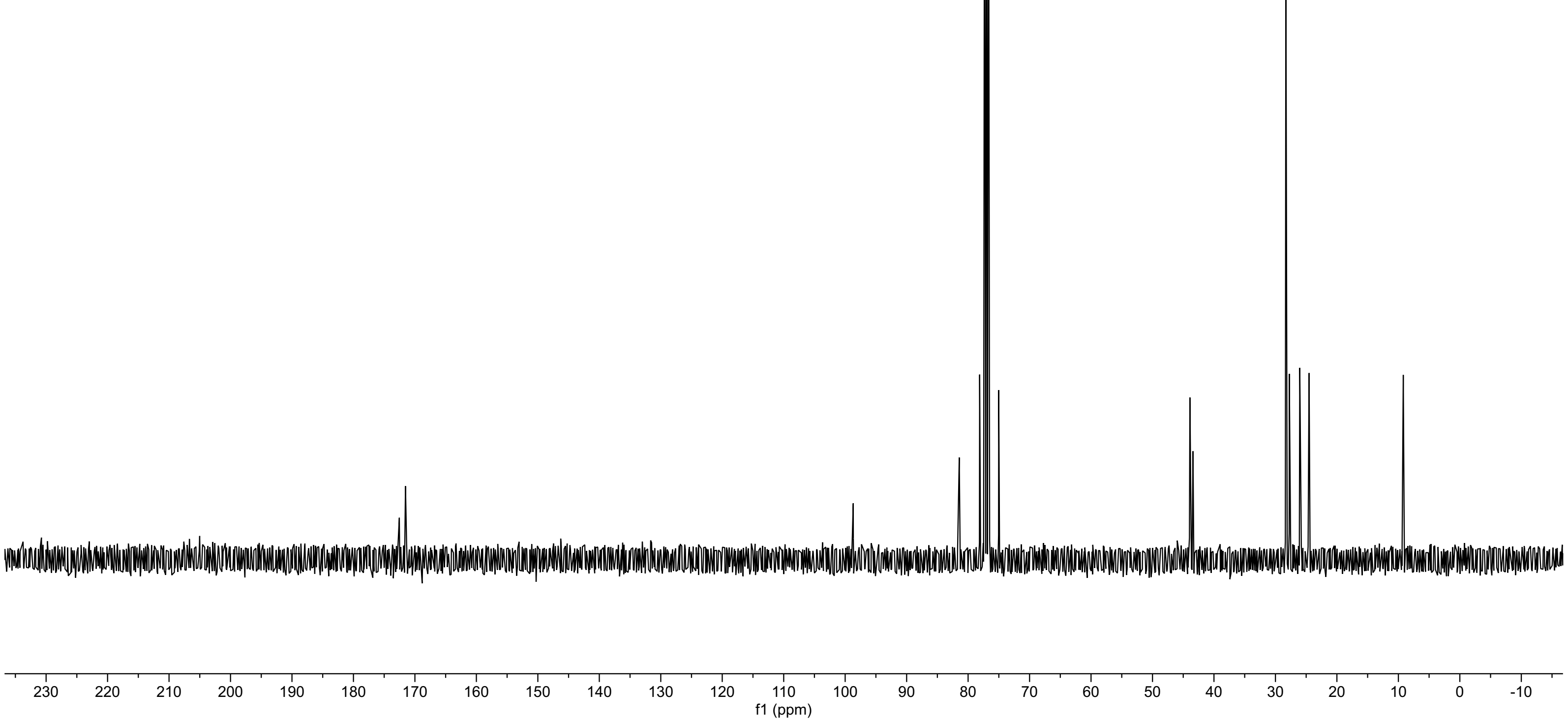

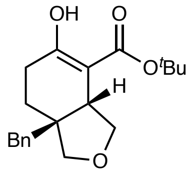

9f

^{1}H NMR (400 MHz, $CDCl_3$)

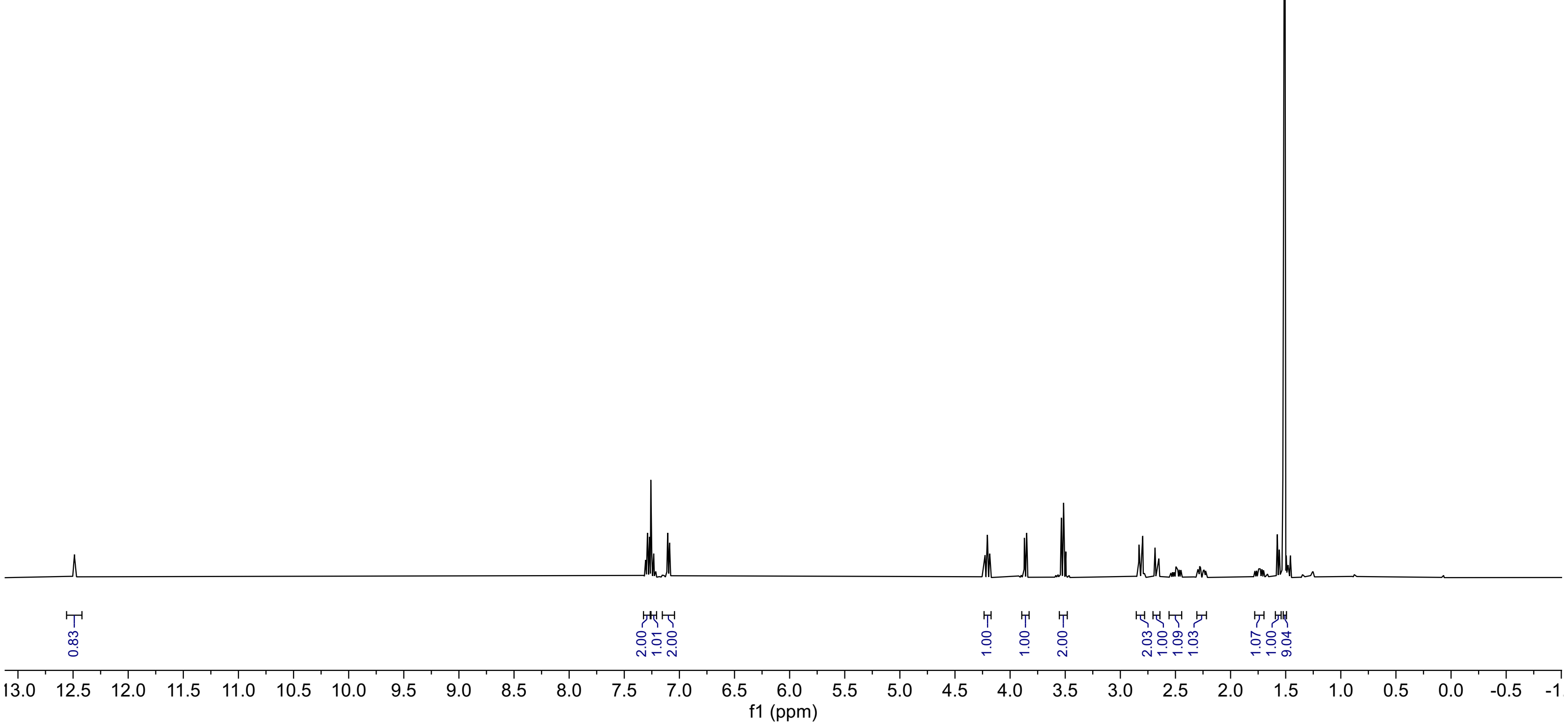

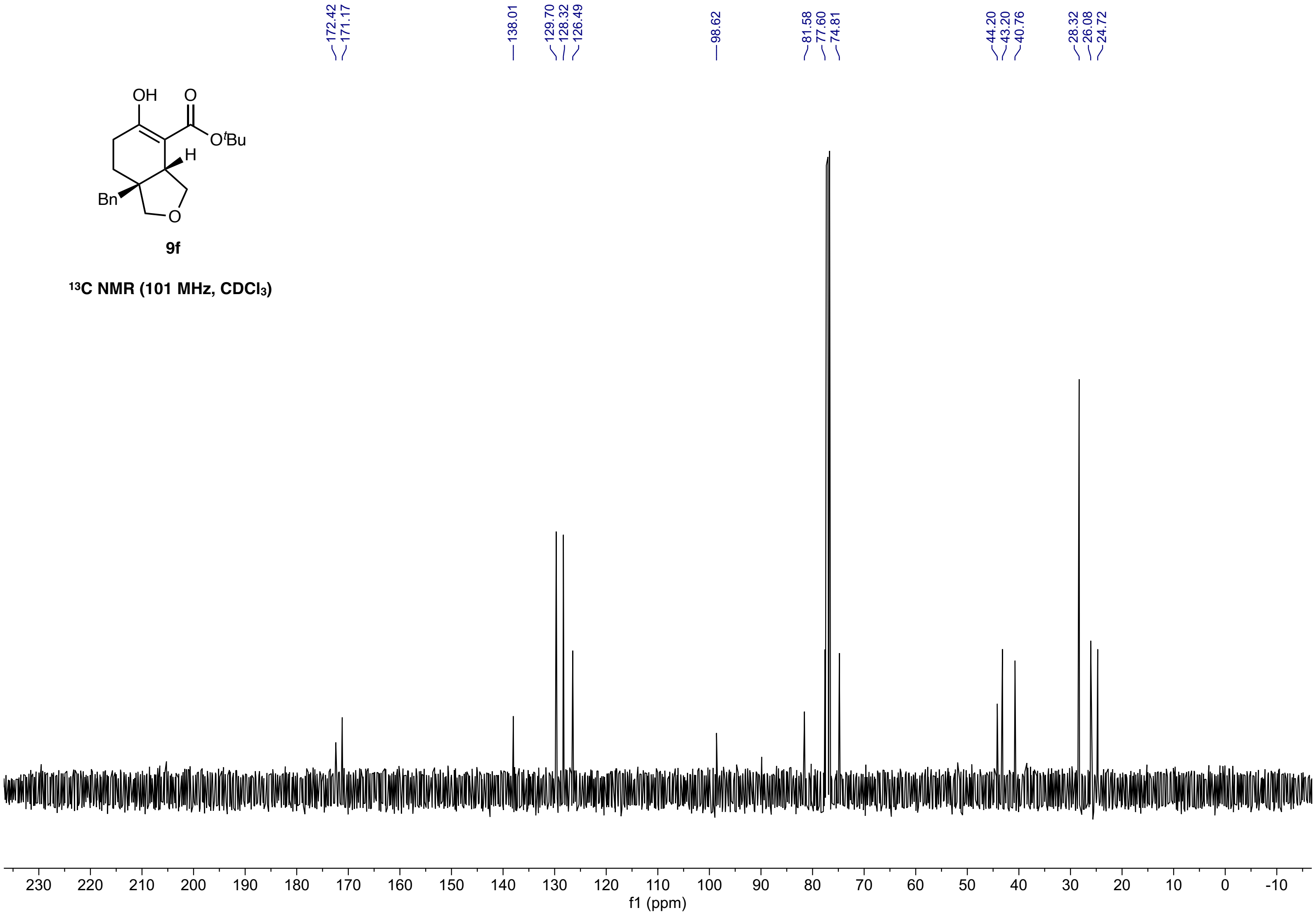

^{13}C NMR (101 MHz, CDCl$_3$)

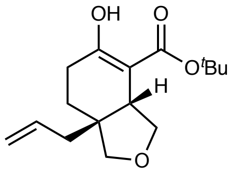

9g

^{1}H NMR (400 MHz, CDCl$_3$)

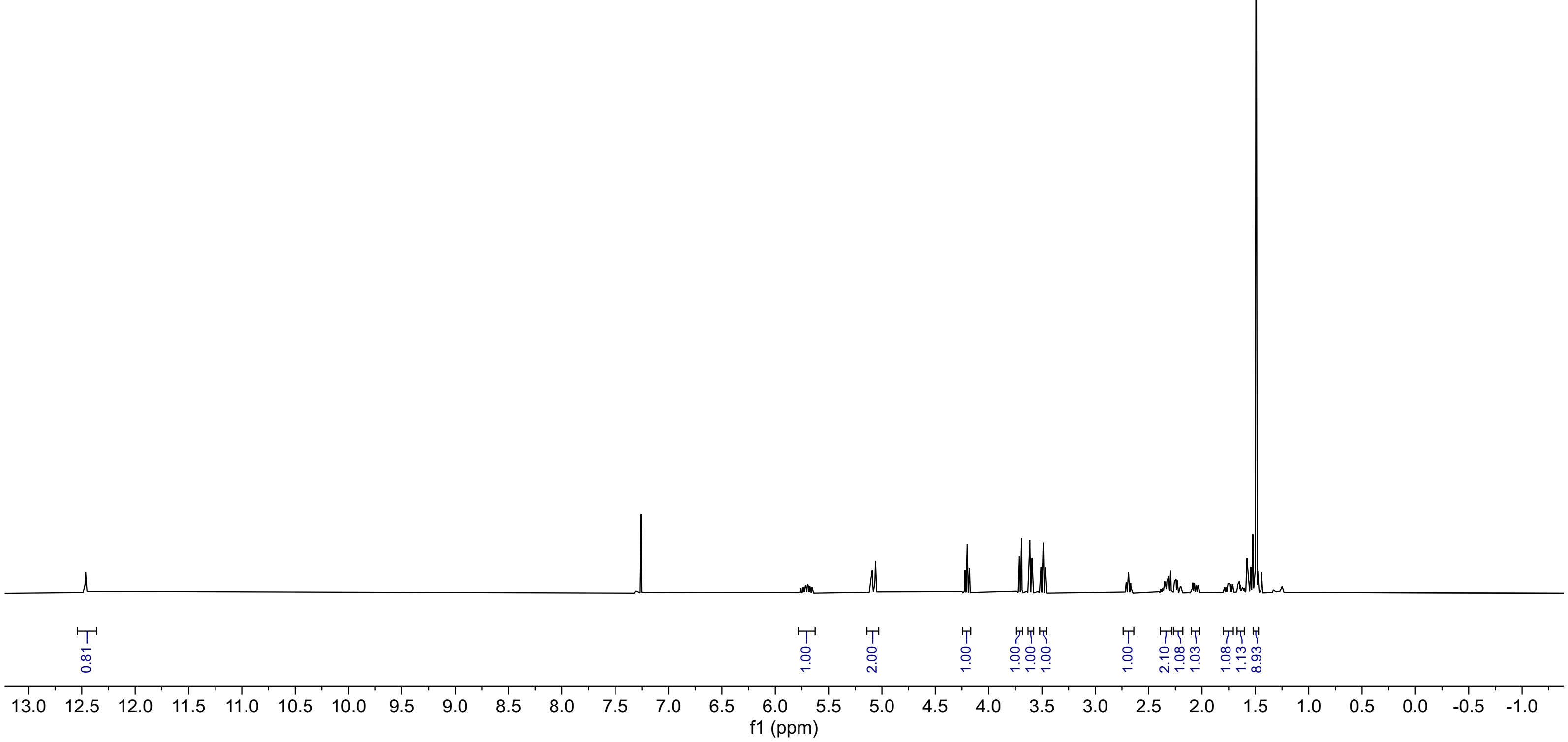

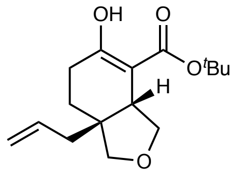

9g

^{13}C NMR (101 MHz, CDCl$_3$)

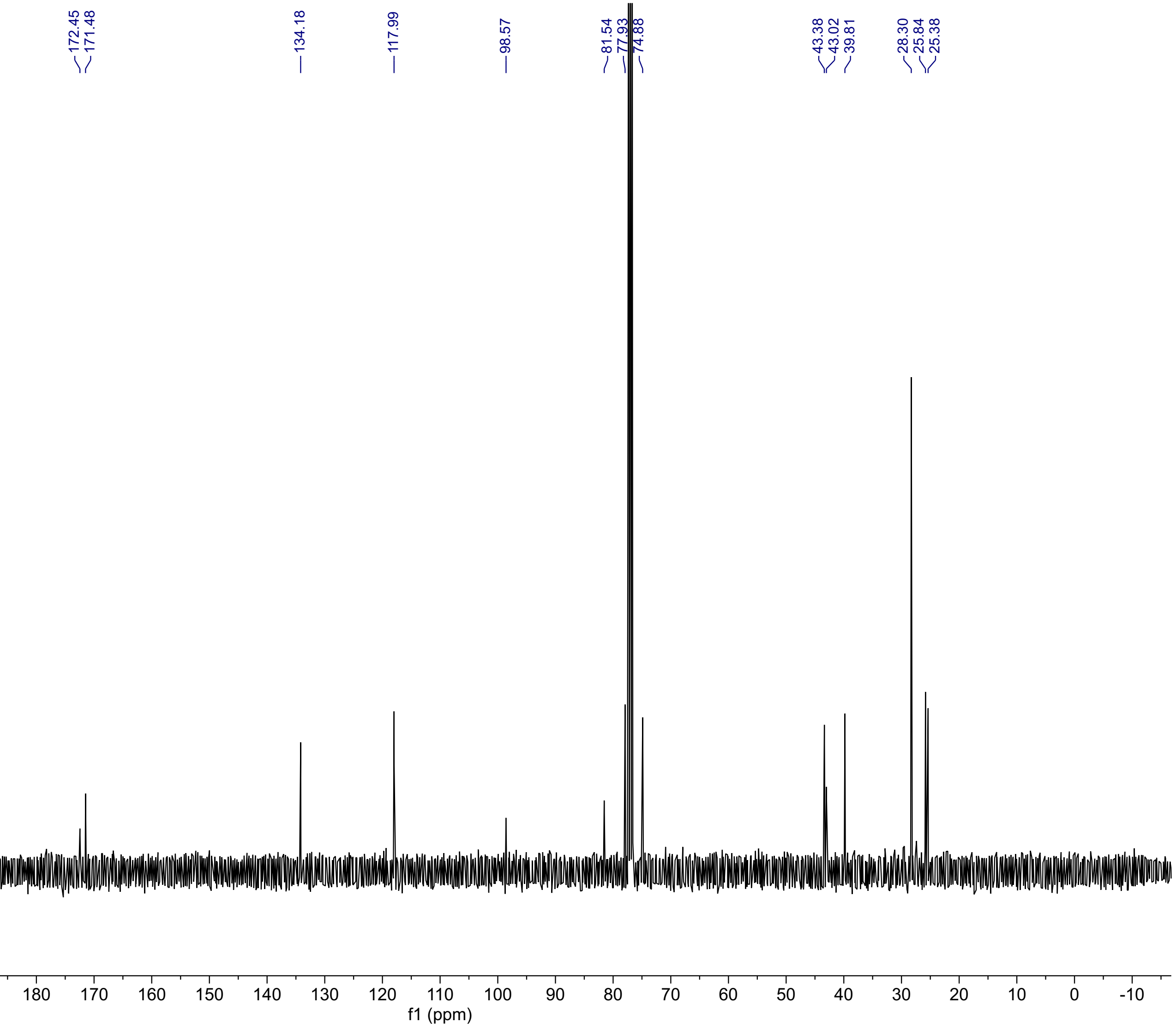

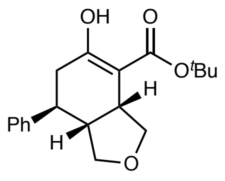

9h

^{1}H NMR (400 MHz, $CDCl_3$)

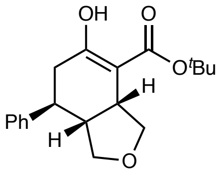

9h

^{13}C NMR (101 MHz, CDCl$_3$)

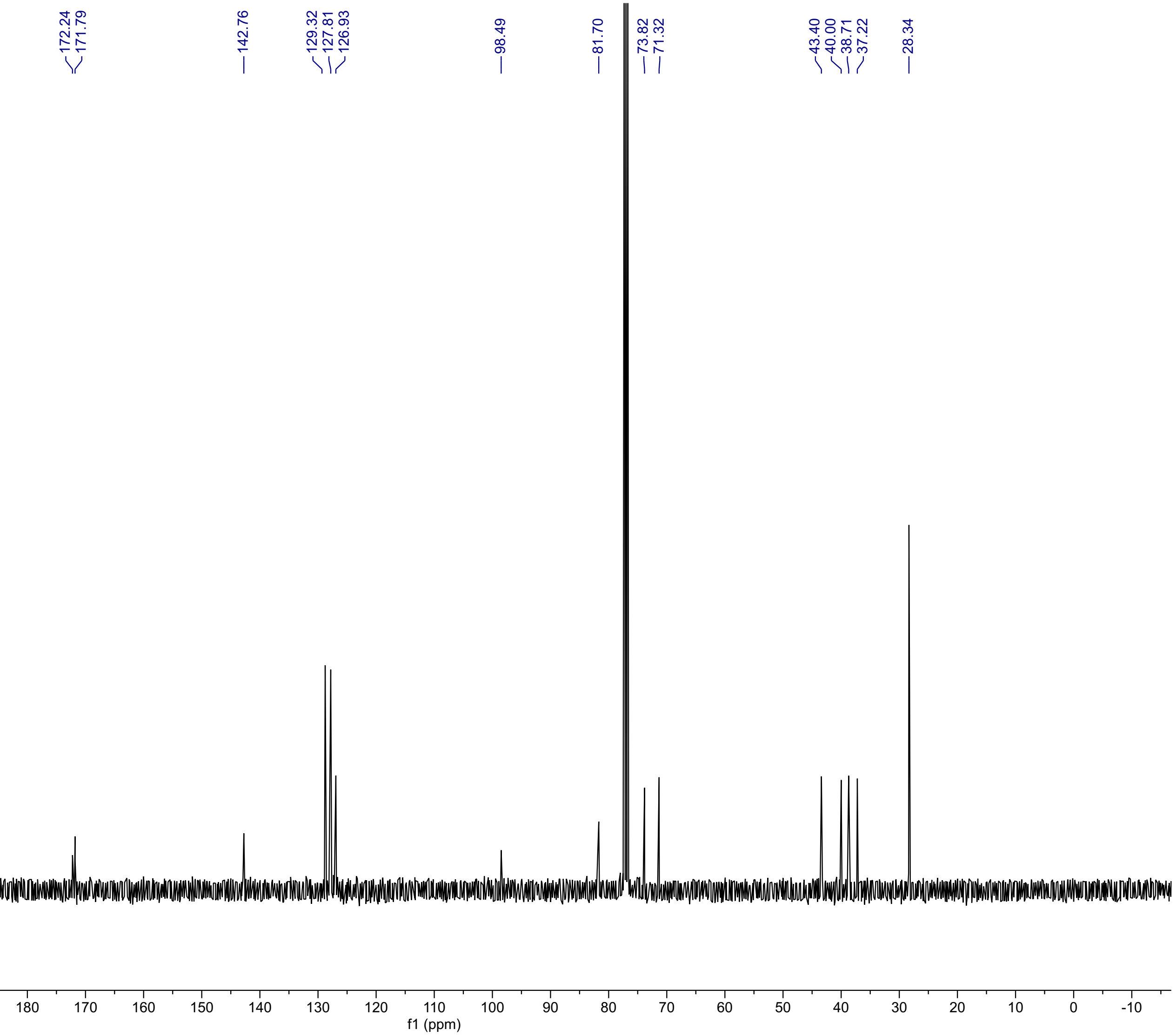

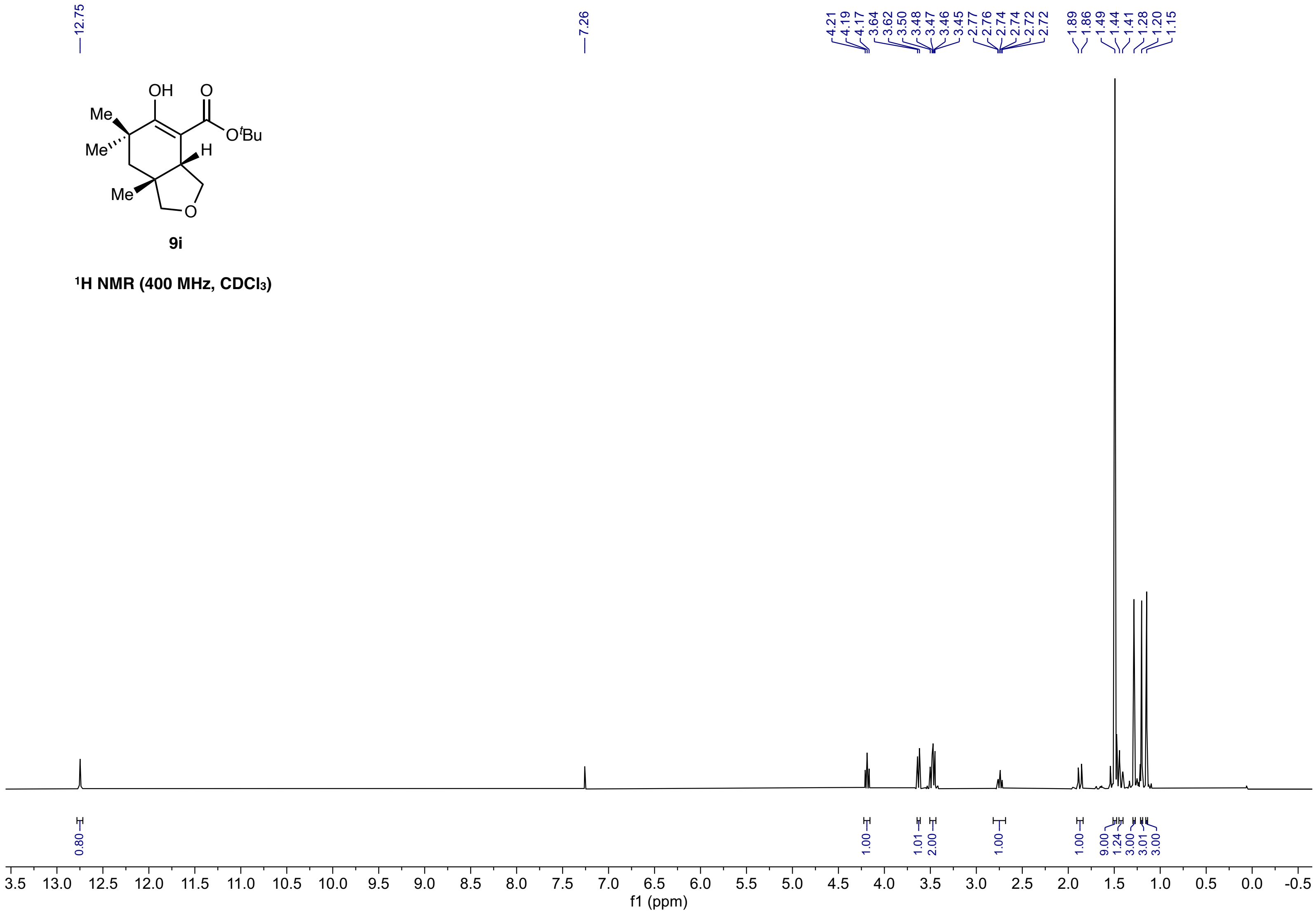
OH
O
Me
Me
O^tBu
H
Me
O
9i
¹H NMR (400 MHz, CDCl3)
12.75
7.26
4.21 4.19 4.17 3.64 3.62 3.50 3.48 3.47 3.46 3.45 2.77 2.76 2.74 2.74 2.72 2.72
1.89 1.86 1.49 1.44 1.41 1.28 1.20 1.15
0.80
1.00
1.01
2.00
1.00
1.00
9.00 1.24 3.00 3.01 3.00
f1 (ppm)

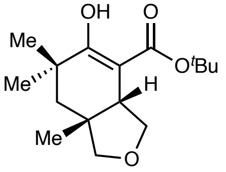

9i

^{13}C NMR (101 MHz, CDCl$_3$)

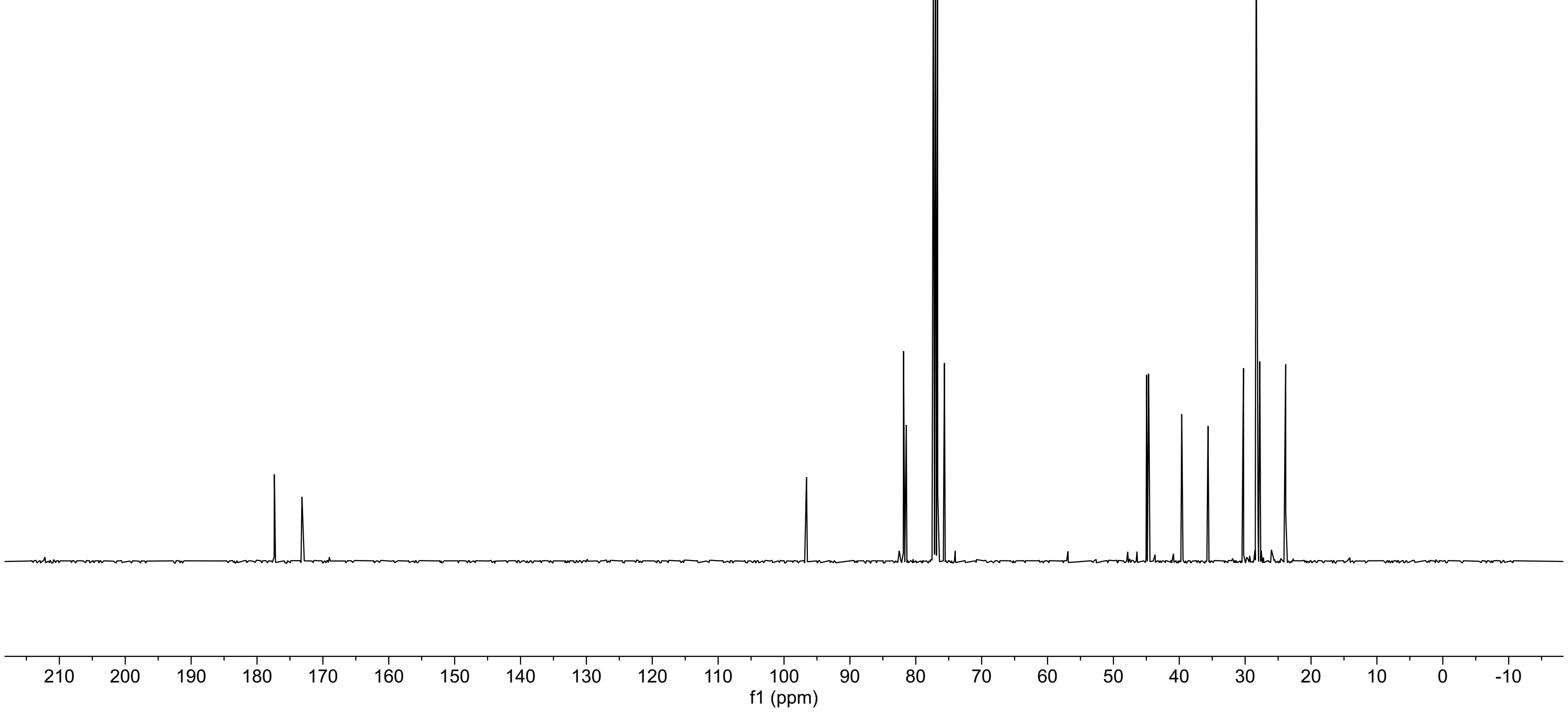

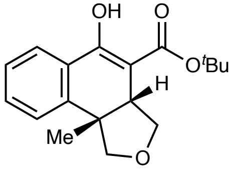

9j

^{1}H NMR (400 MHz, CDCl$_3$)

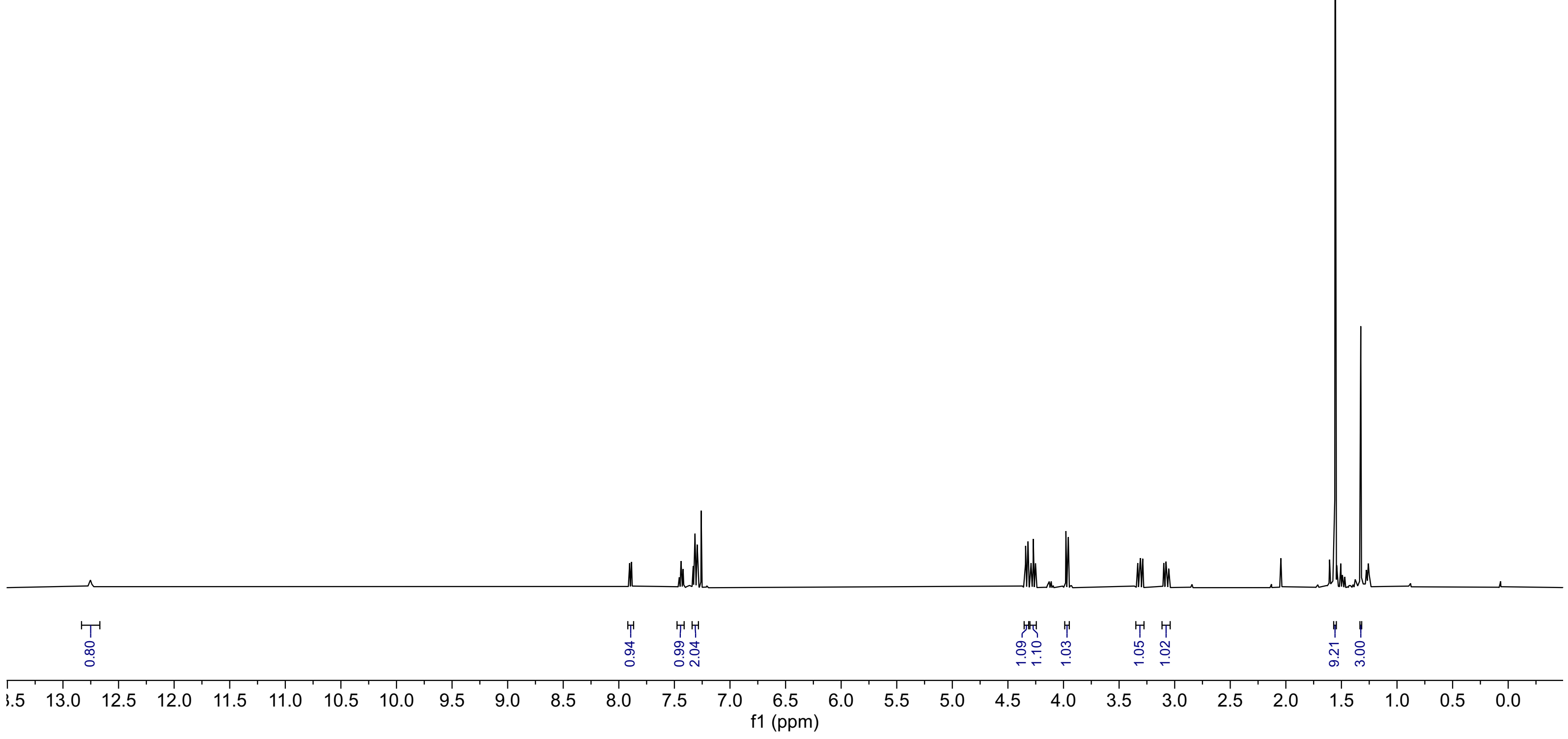

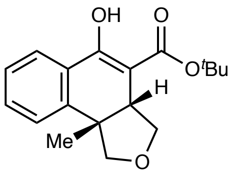

9j

^{13}C NMR (101 MHz, $CDCl_3$)

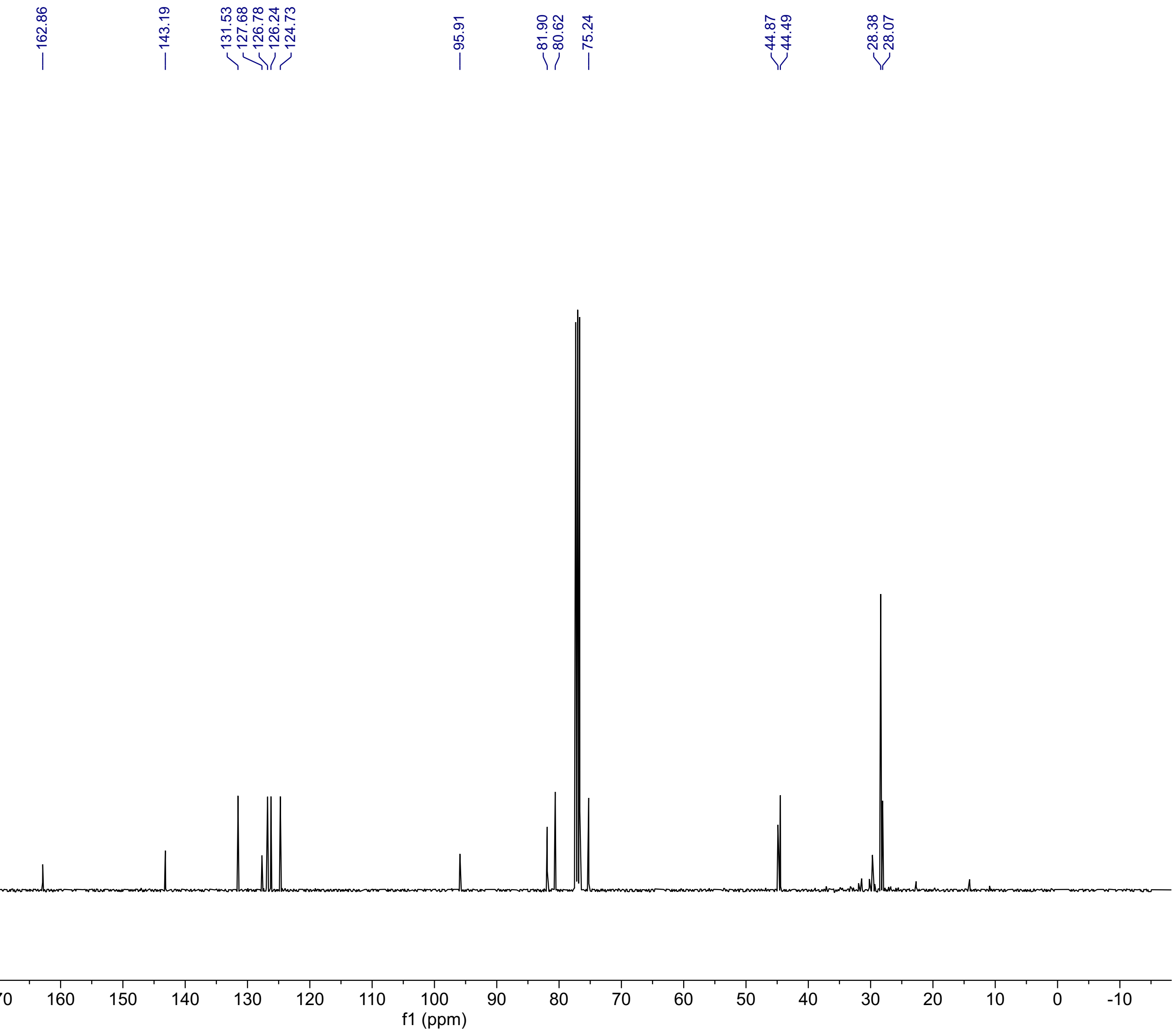

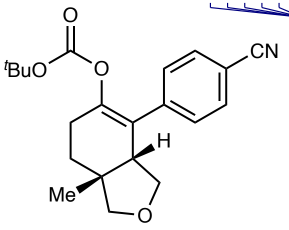

9k

^{1}H NMR (400 MHz, $CDCl_3$)

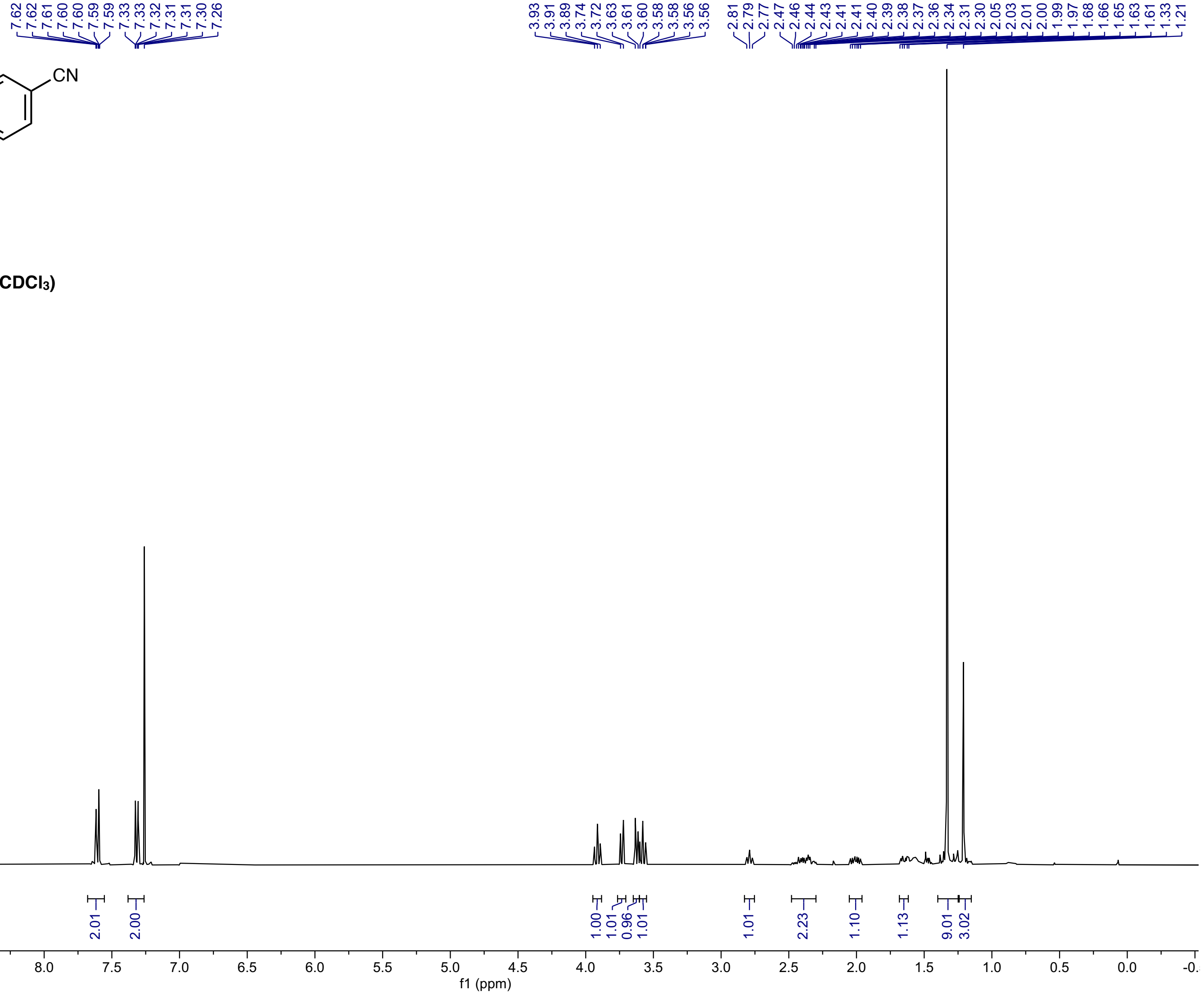

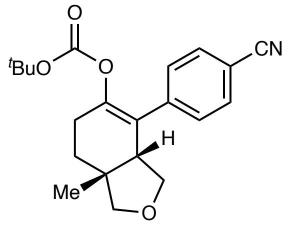

9k

^{13}C NMR (101 MHz, CDCl$_3$)

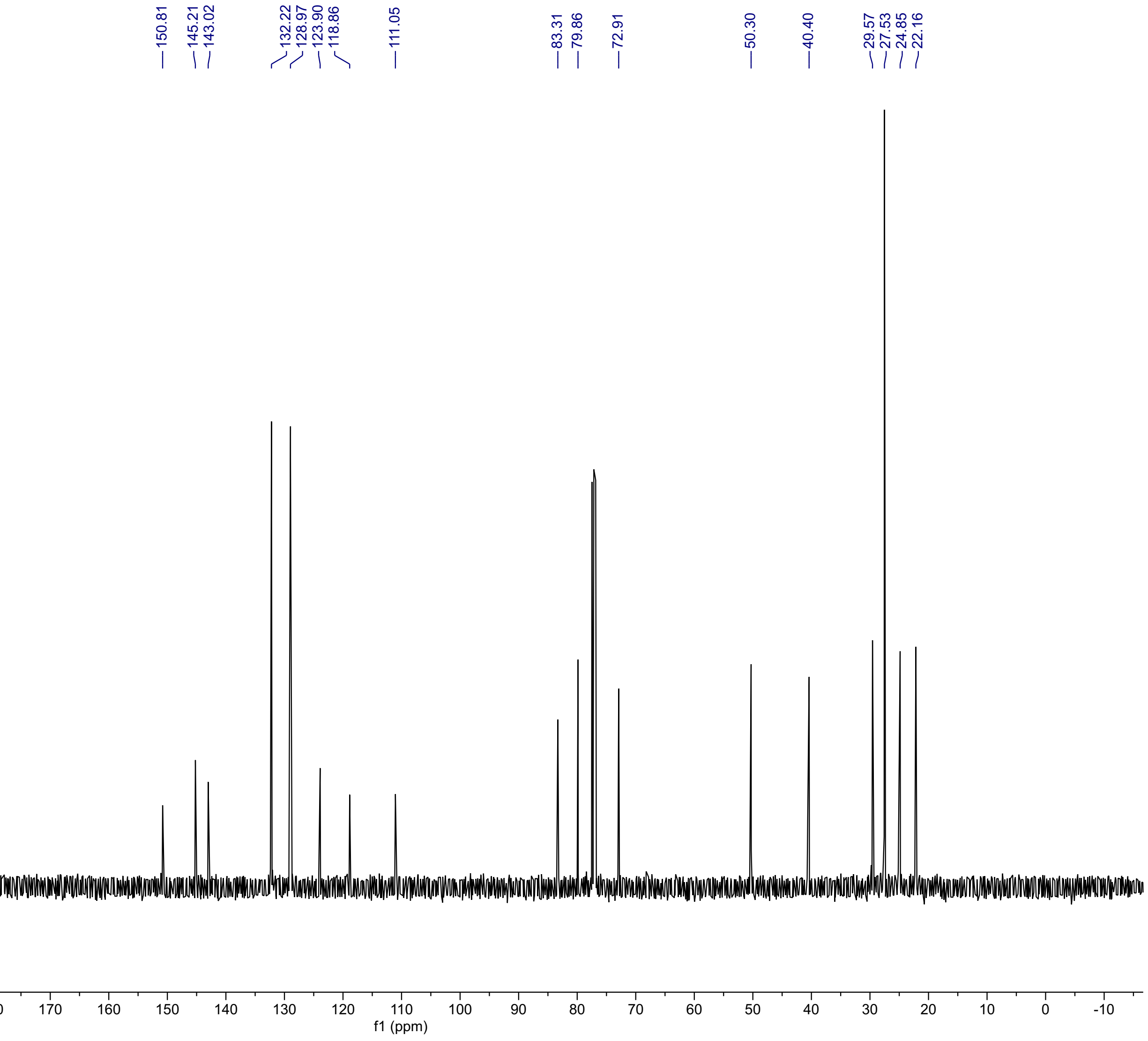

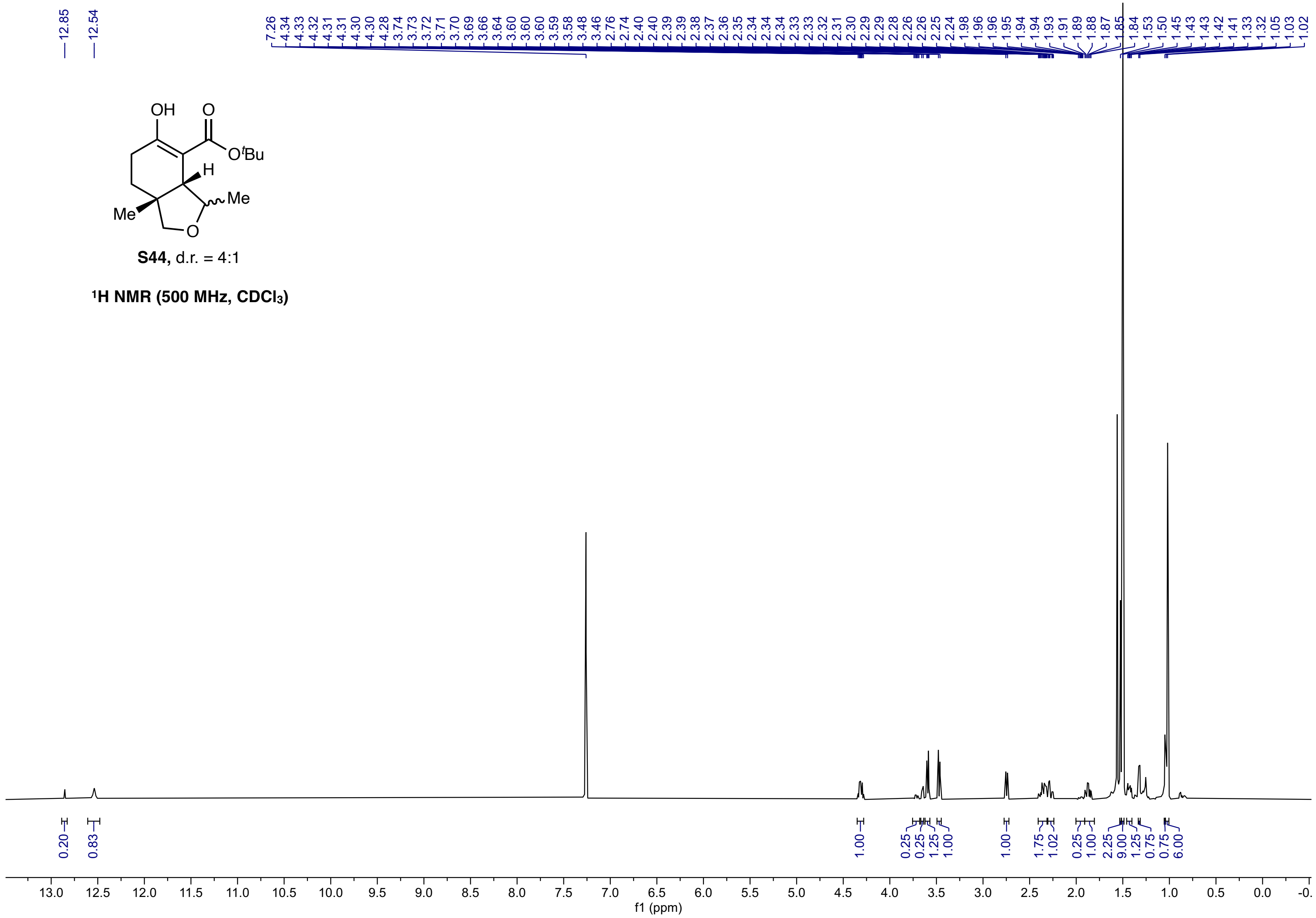
12.85
12.54
7.26 4.34 4.33 4.32 4.31 4.31 4.30 4.28 3.74 3.73 3.72 3.71 3.70 3.69 3.66 3.64 3.60 3.60 3.60 3.59 3.58 3.48 3.46 2.76 2.74 2.40 2.40 2.39 2.39 2.38 2.37 2.36 2.35 2.34 2.34 2.34 2.33 2.33 2.32 2.31 2.30 2.29 2.29 2.28 2.26 2.26 2.25 2.24 1.98 1.96 1.96 1.95 1.94 1.94 1.93 1.91 1.89 1.88 1.87 1.85 1.84 1.53 1.50 1.45 1.43 1.43 1.42 1.41 1.33 1.32 1.05 1.03 1.02
OH
O
O^{t}Bu
H
Me
Me
O
S44, d.r. = 4:1
^{1}H NMR (500 MHz, CDCl$_3$)
0.20
0.83
1.00
0.25
0.25
1.25
1.00
1.00
1.75
1.02
0.25
1.00
2.25
9.00
1.25
0.75
0.75
6.00
13.0 12.5 12.0 11.5 11.0 10.5 10.0 9.5 9.0 8.5 8.0 7.5 7.0 6.5 6.0 5.5 5.0 4.5 4.0 3.5 3.0 2.5 2.0 1.5 1.0 0.5 0.0 -0.
f1 (ppm)

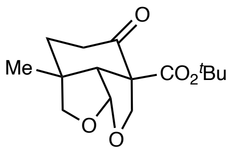

22

^{1}H NMR (400 MHz, CDCl$_3$)

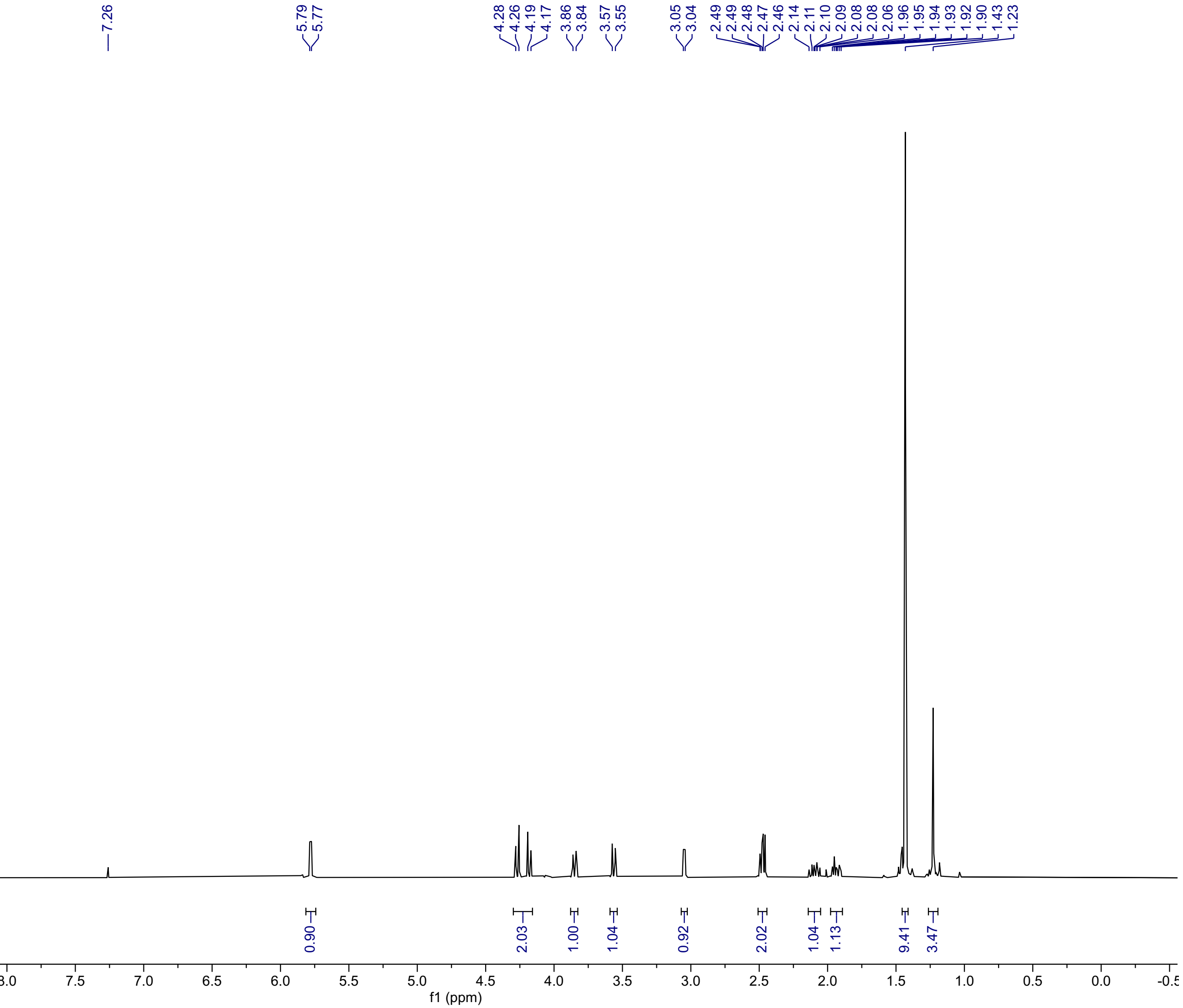

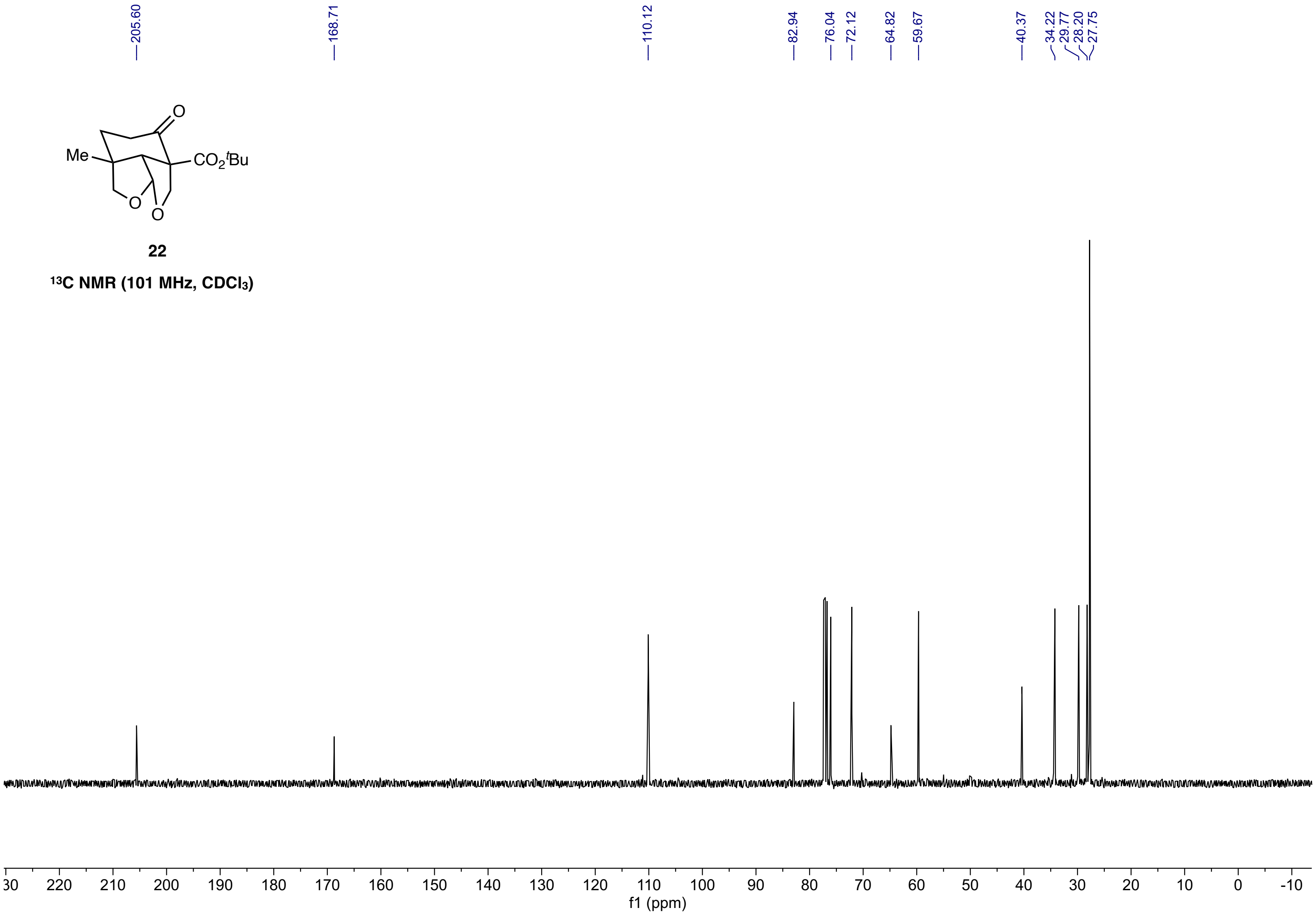

22

^{13}C NMR (101 MHz, $CDCl_3$)

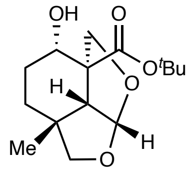

23

^{1}H NMR (400 MHz, $CDCl_3$)

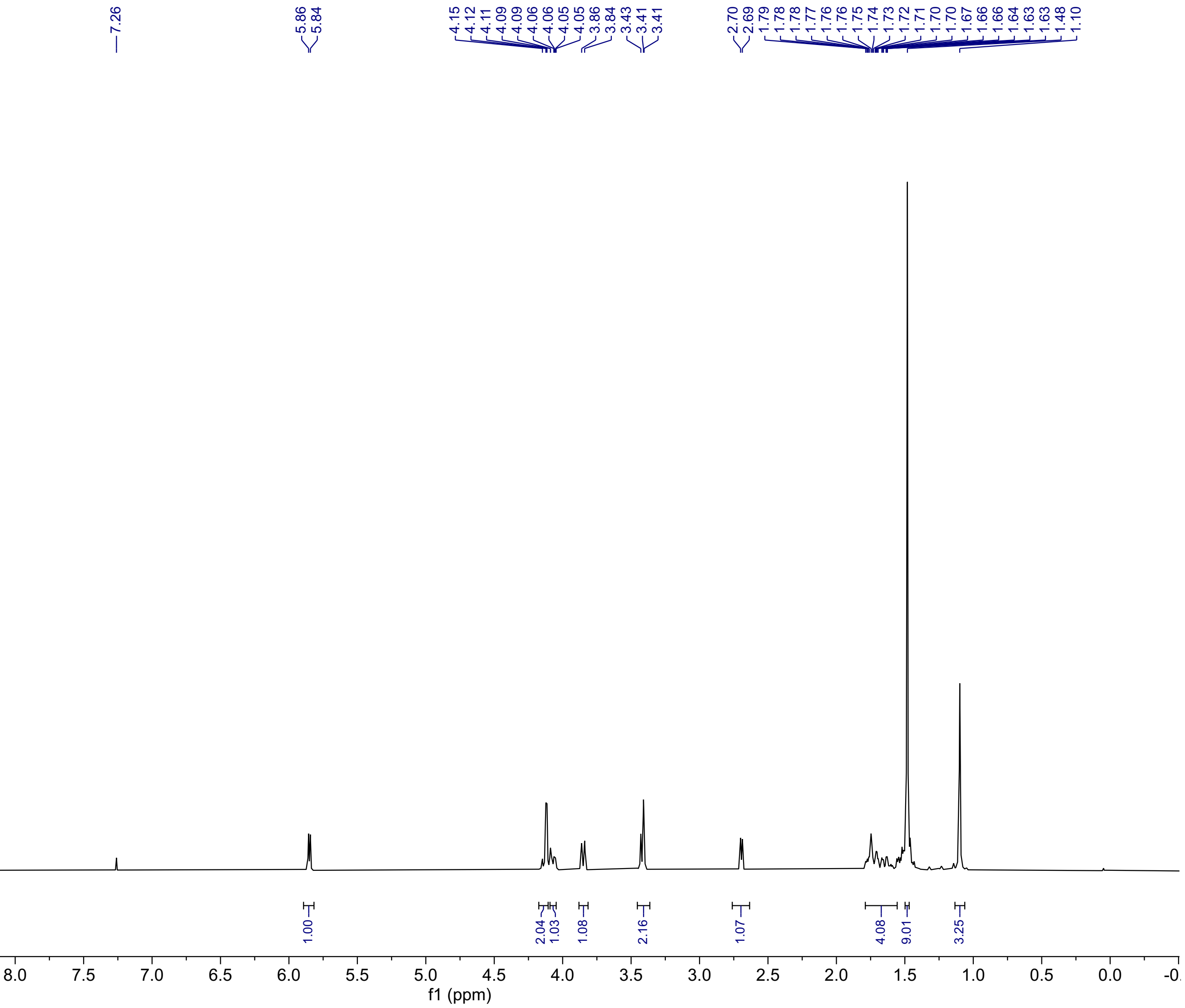

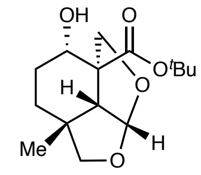

23

^{13}C NMR (101 MHz, CDCl$_3$)

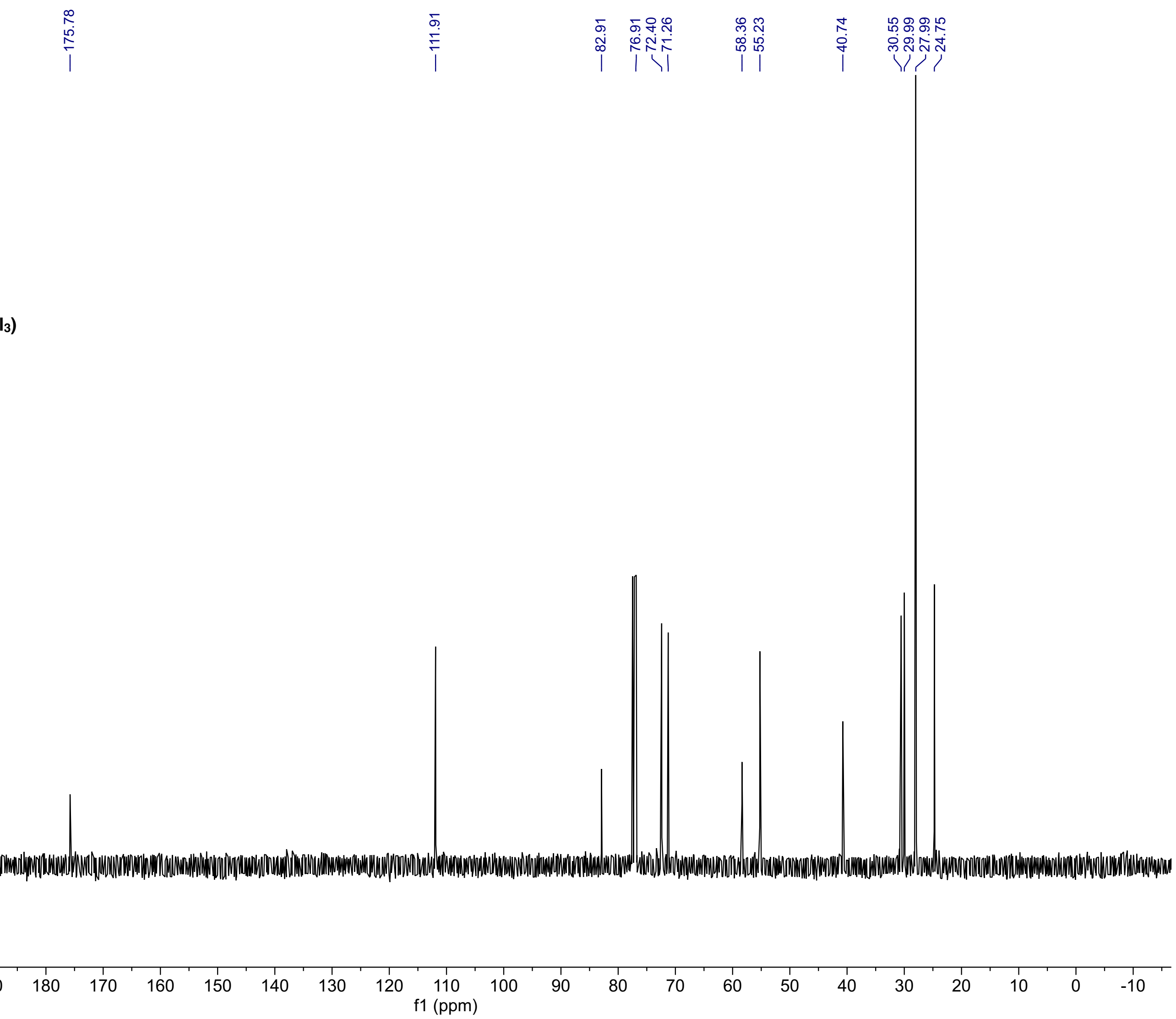

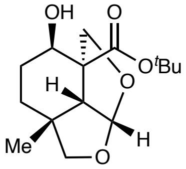

epi-**23**

^{1}H NMR (400 MHz, CDCl$_3$)

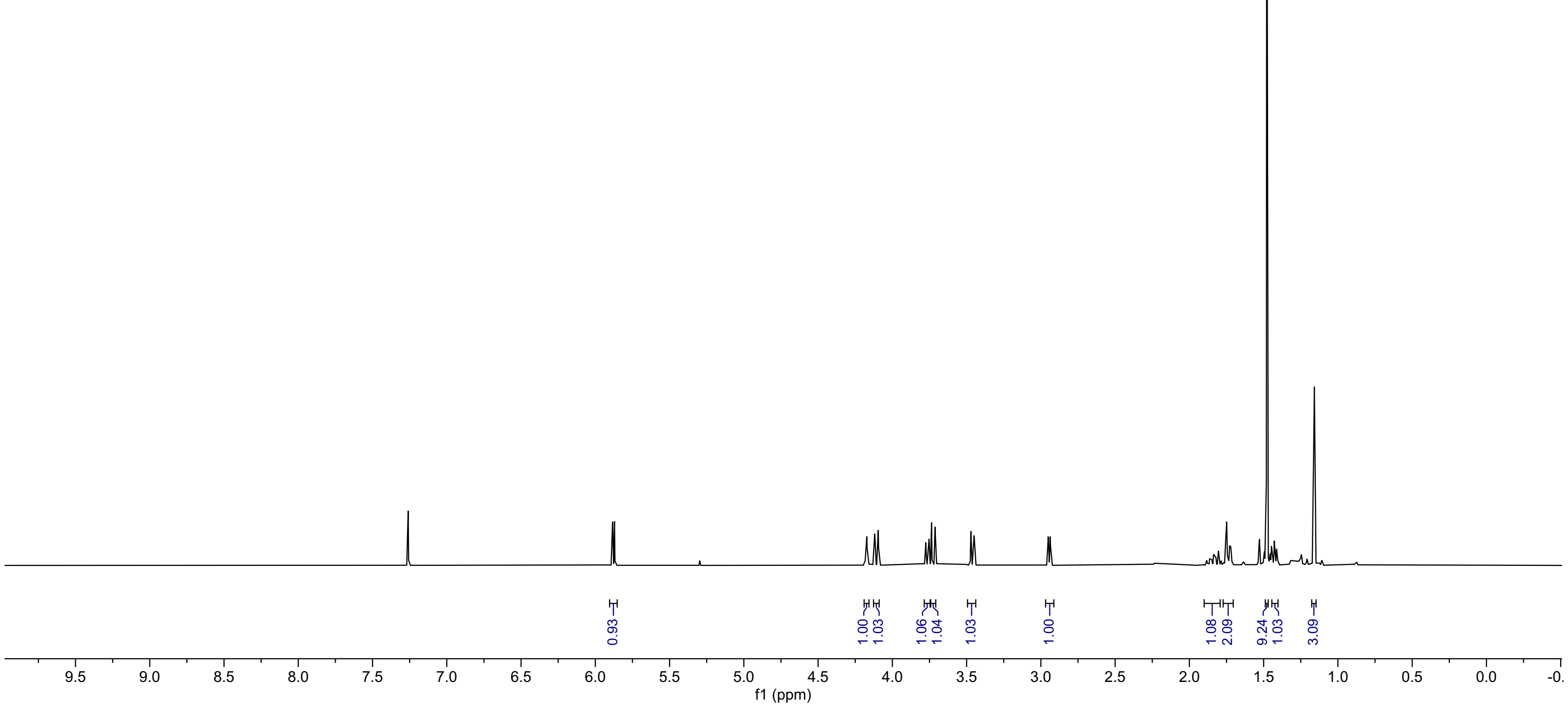

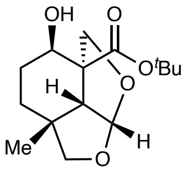

epi-**23**

^{13}C NMR (101 MHz, CDCl$_3$)

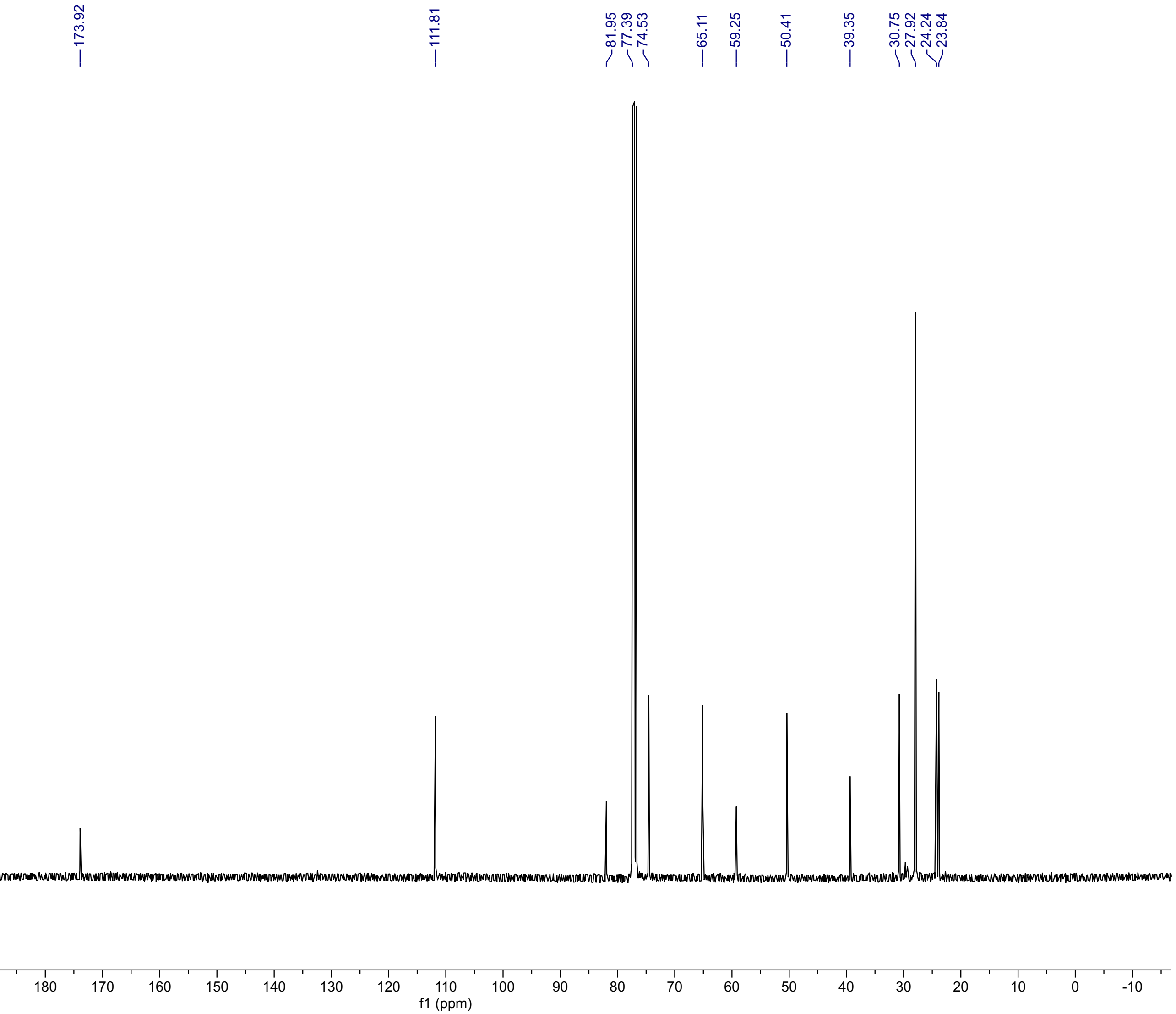

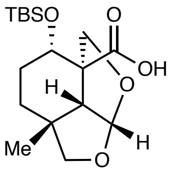

24

^{1}H NMR (400 MHz, CDCl$_3$)

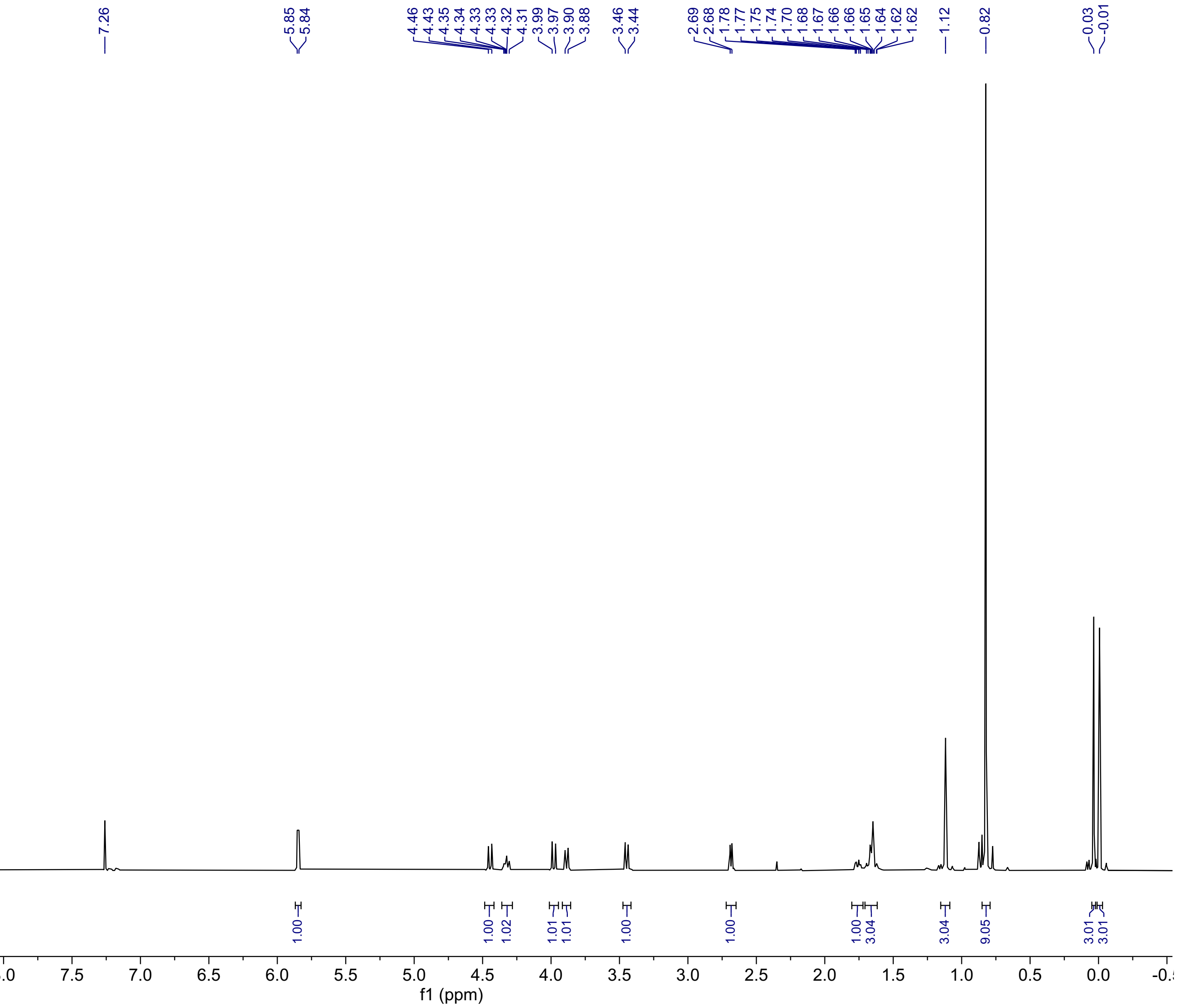

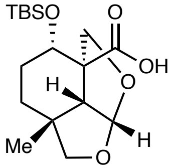

24

^{13}C NMR (101 MHz, CDCl$_3$)

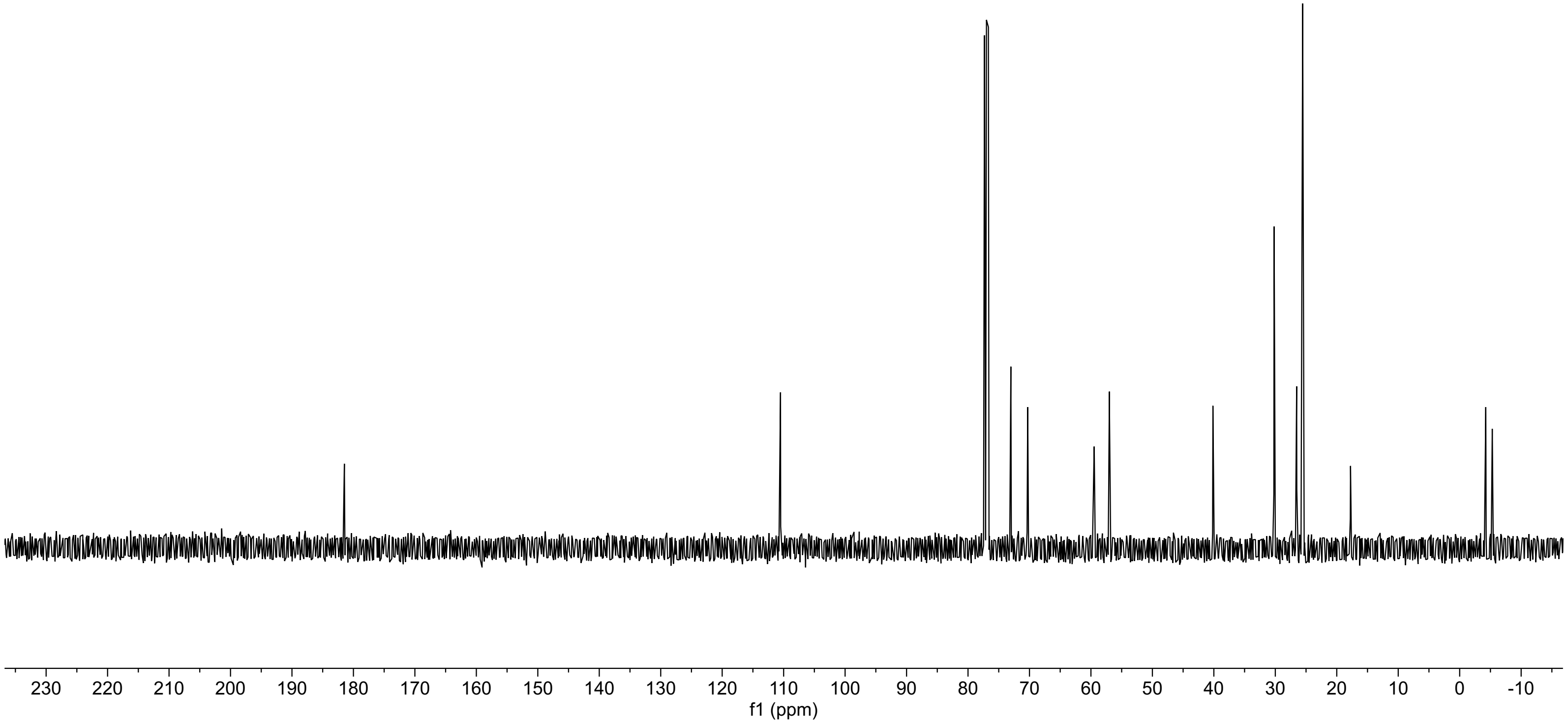

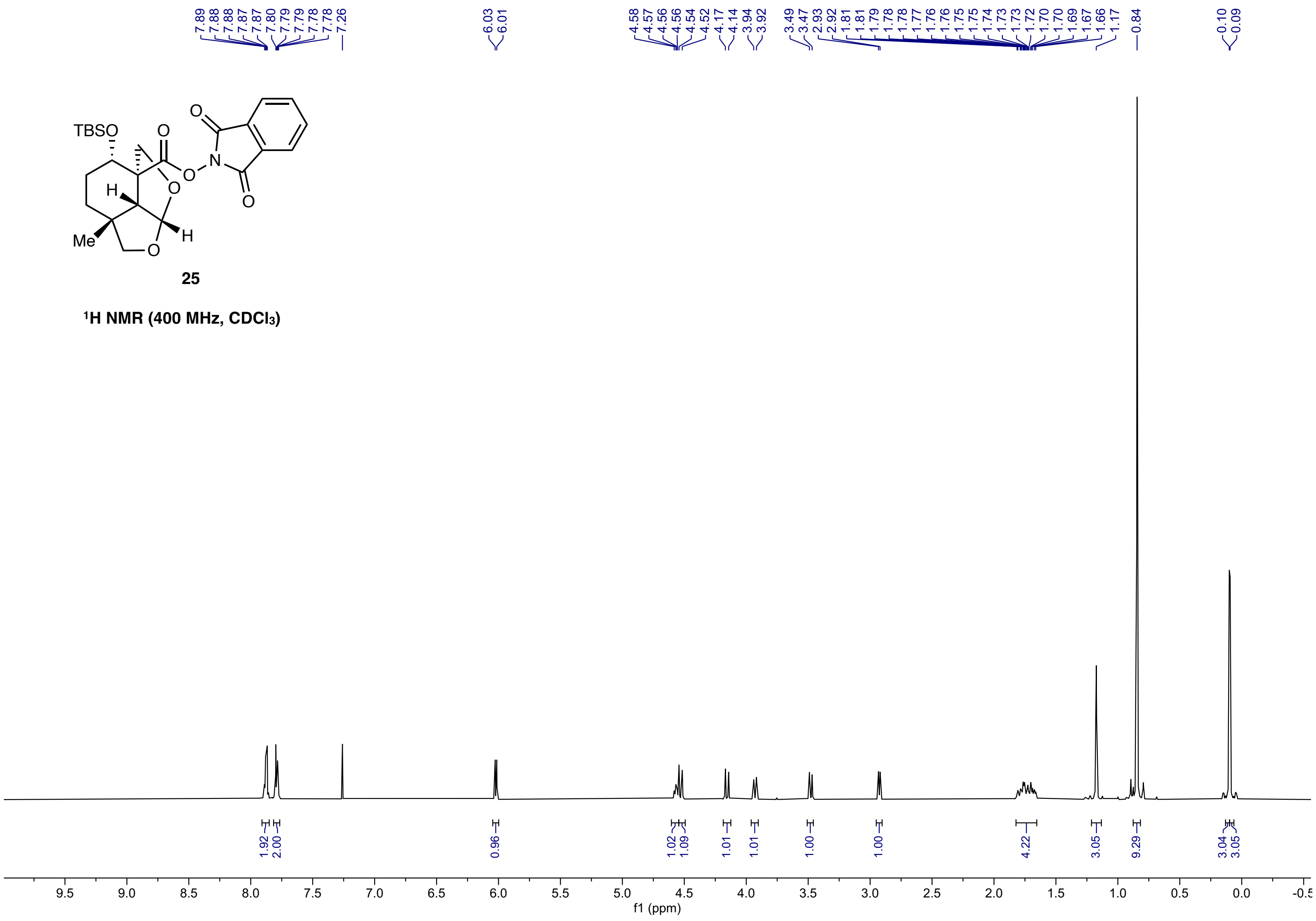
TBSO
Me
25
^1H NMR (400 MHz, $CDCl_3$)
7.89 7.88 7.88 7.87 7.87 7.80 7.79 7.79 7.78 7.78 7.26
6.03 6.01
4.58 4.57 4.56 4.56 4.54 4.52 4.17 4.14 3.94 3.92
3.49 3.47 2.93 2.92 1.81 1.81 1.79 1.78 1.78 1.77 1.76 1.75 1.75 1.74 1.73 1.73 1.72 1.70 1.70 1.69 1.67 1.66 1.17
0.84
0.10 0.09
1.92 2.00 0.96 1.02 1.09 1.01 1.01 1.00 1.00 4.22 3.05 9.29 3.04 3.05
f1 (ppm)

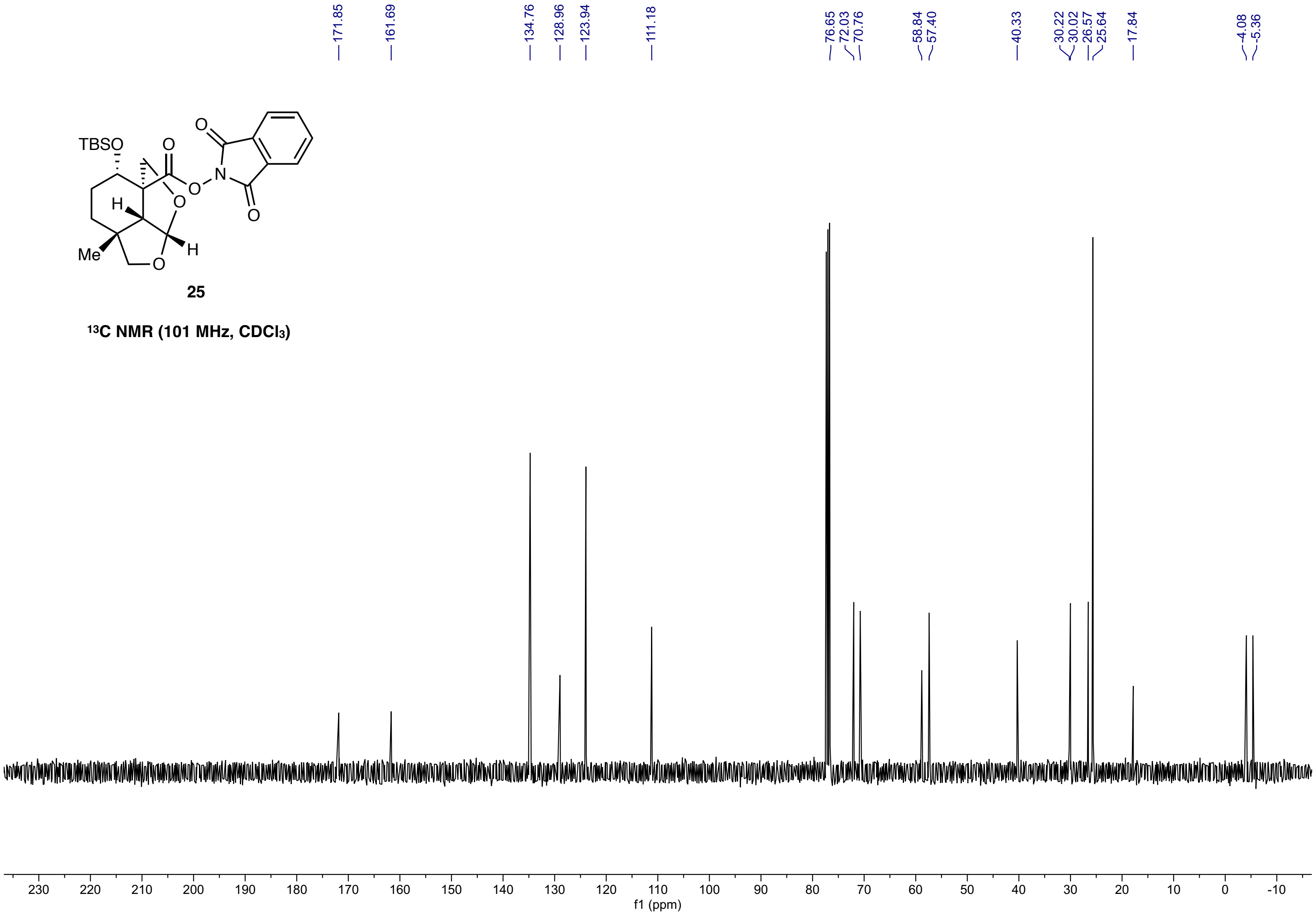
171.85
161.69
134.76
128.96
123.94
111.18
76.65
72.03
70.76
58.84
57.40
40.33
30.22
30.02
26.57
25.64
17.84
-4.08
-5.36
TBSO
O
O
O
N
O
H
O
H
Me
O
25
13C NMR (101 MHz, CDCl3)
230
220
210
200
190
180
170
160
150
140
130
120
110
100
90
80
70
60
50
40
30
20
10
0
-10
f1 (ppm)

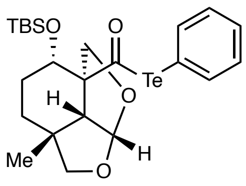

S39

^{1}H NMR (500 MHz, CDCl$_3$)

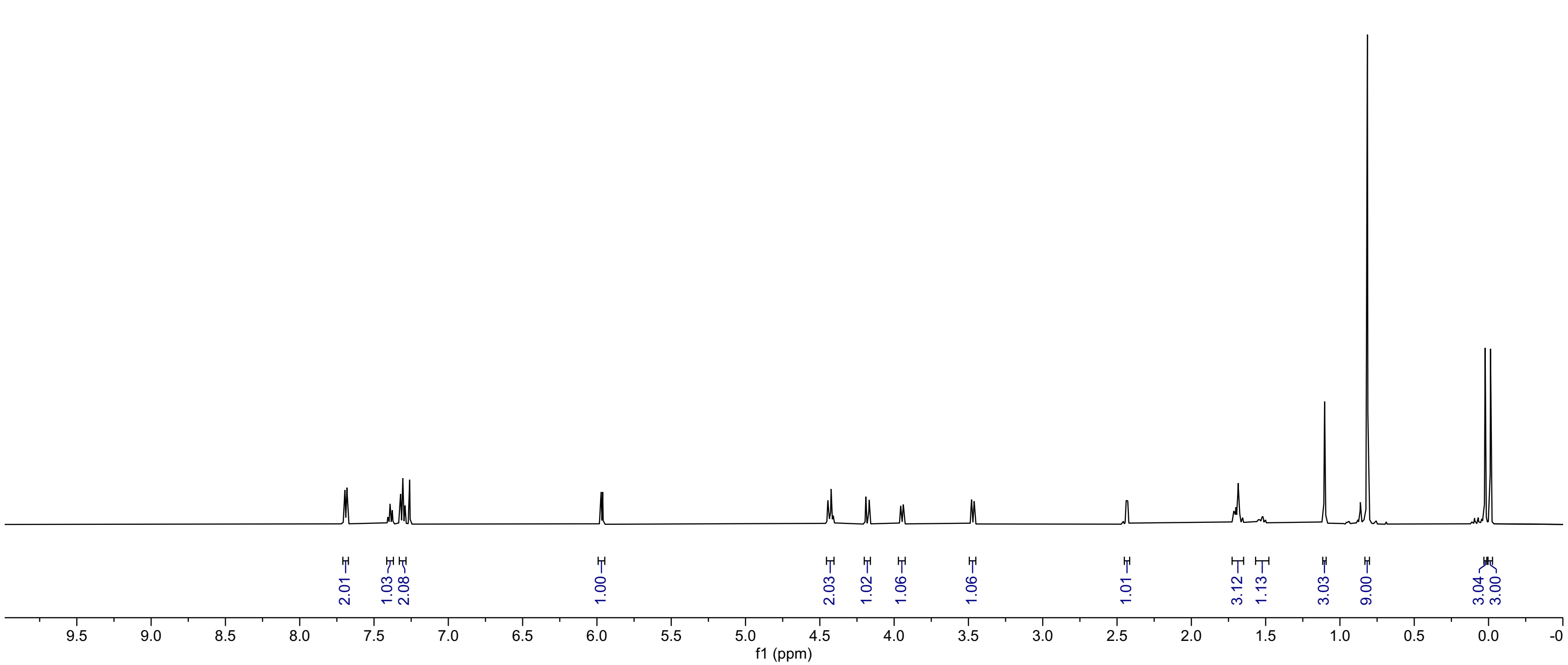

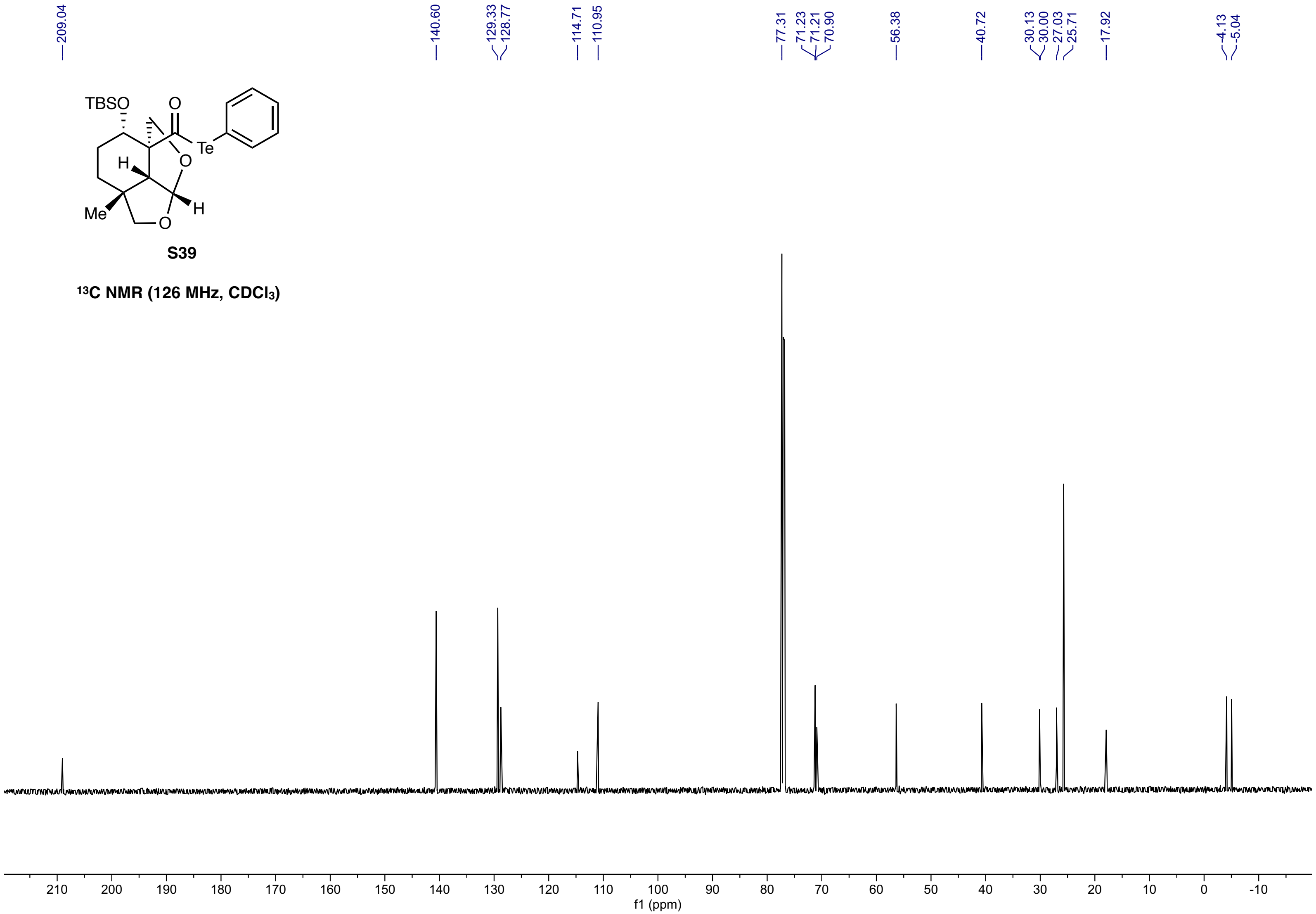
209.04
140.60
129.33
128.77
114.71
110.95
77.31
71.23
71.21
70.90
56.38
40.72
30.13
30.00
27.03
25.71
17.92
-4.13
-5.04
TBSO
O
Te
H
O
Me
O
H
S39
13C NMR (126 MHz, CDCl3)
210 200 190 180 170 160 150 140 130 120 110 100 90 80 70 60 50 40 30 20 10 0 -10
f1 (ppm)

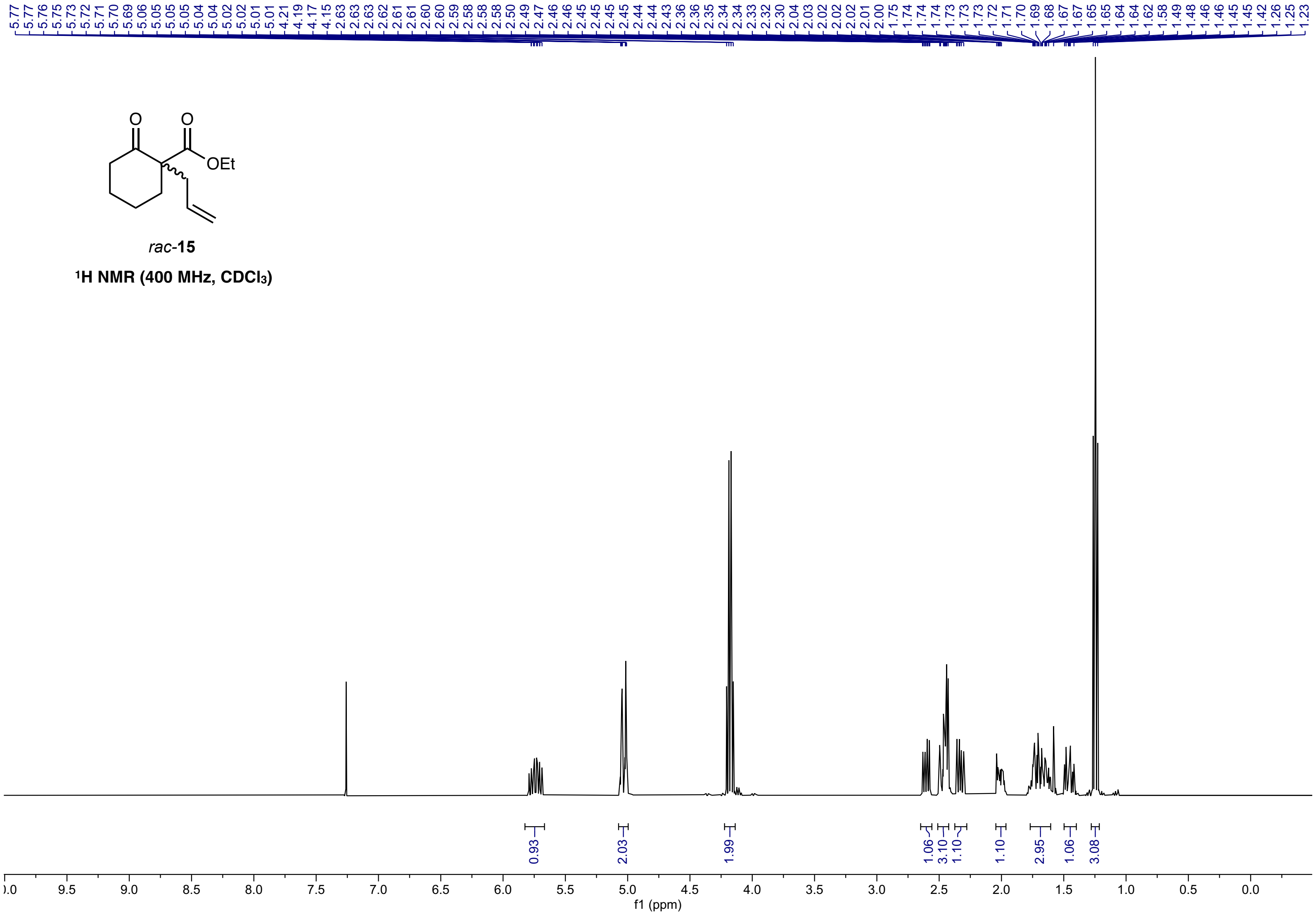

rac-**15**

¹H NMR (400 MHz, CDCl₃)

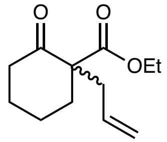

rac-**15**

^{13}C NMR (101 MHz, $CDCl_3$)

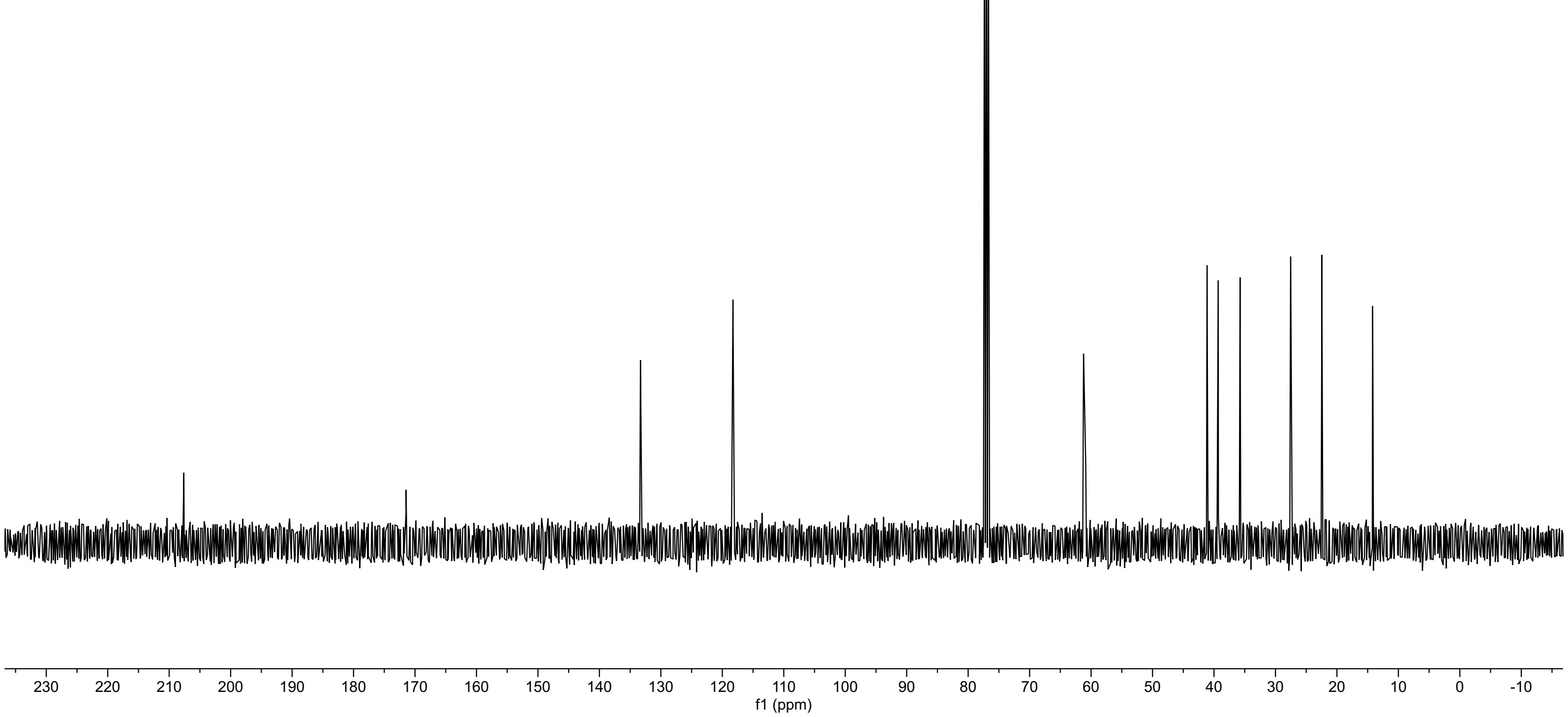

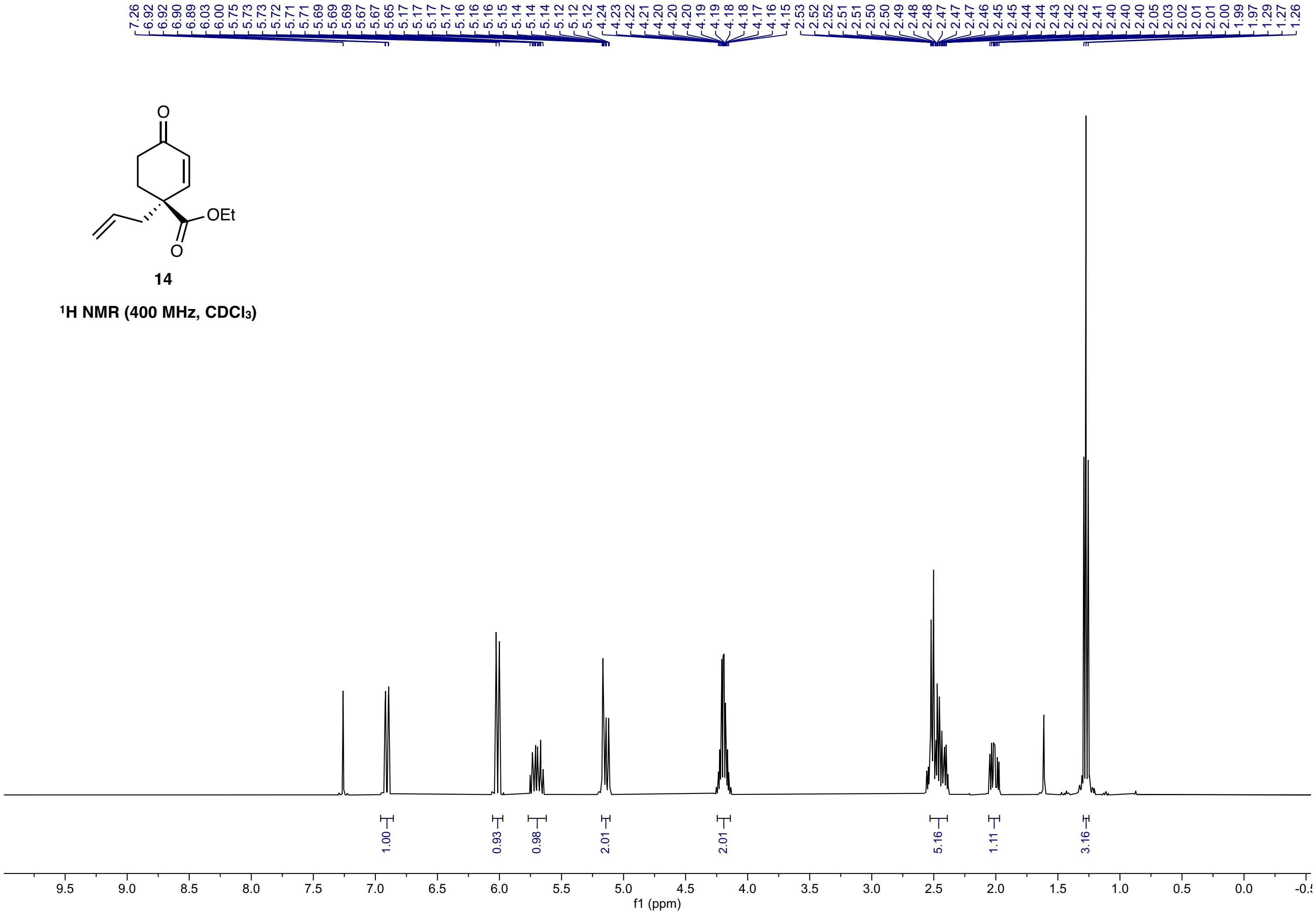
O
OEt
O
14
1H NMR (400 MHz, CDCl3)
1.00
0.93
0.98
2.01
2.01
5.16
1.11
3.16
f1 (ppm)

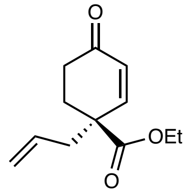

14

^{13}C NMR (101 MHz, CDCl$_3$)

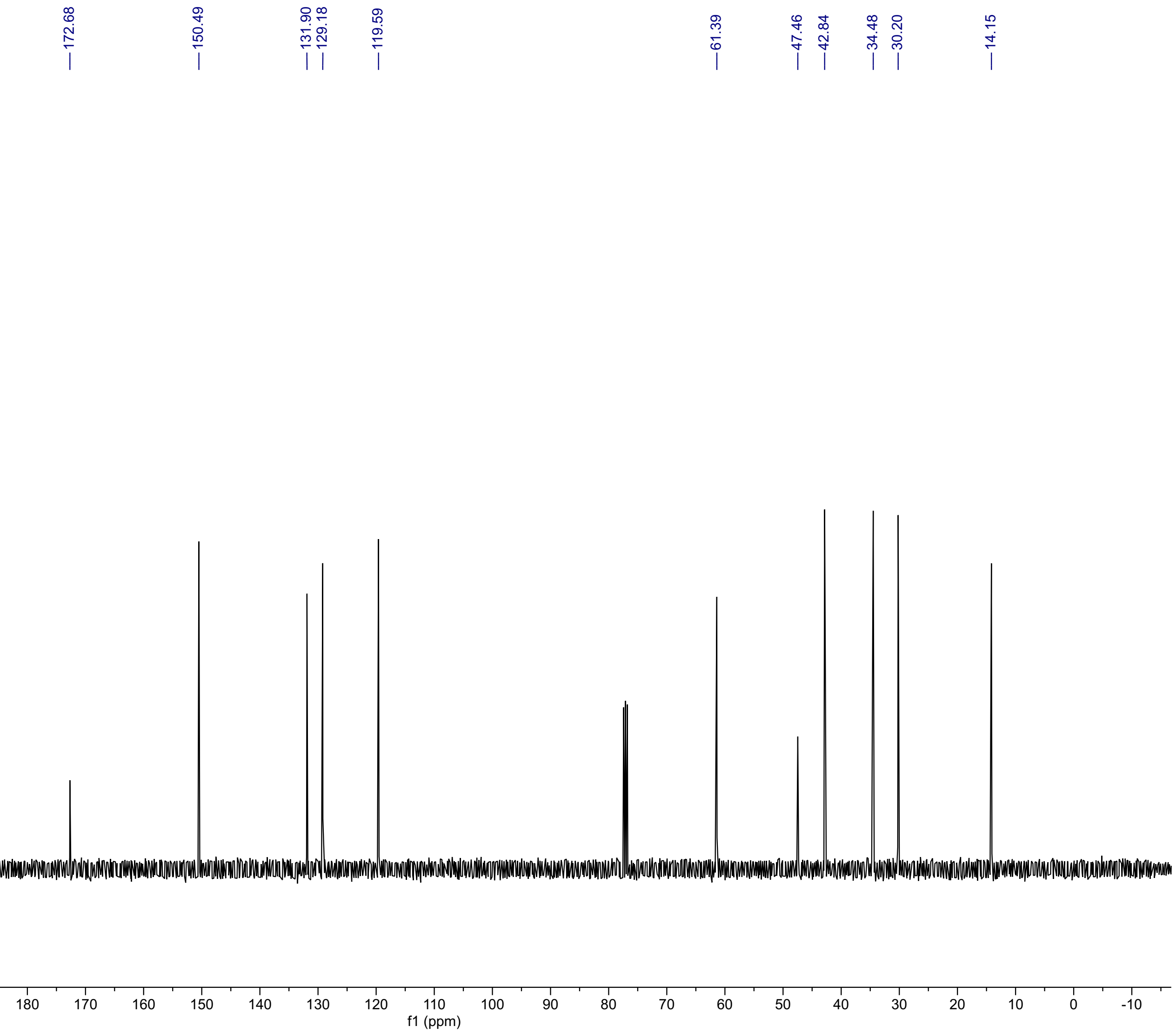

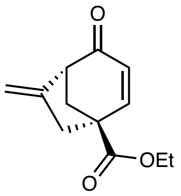

13

^{1}H NMR (400 MHz, $CDCl_3$)

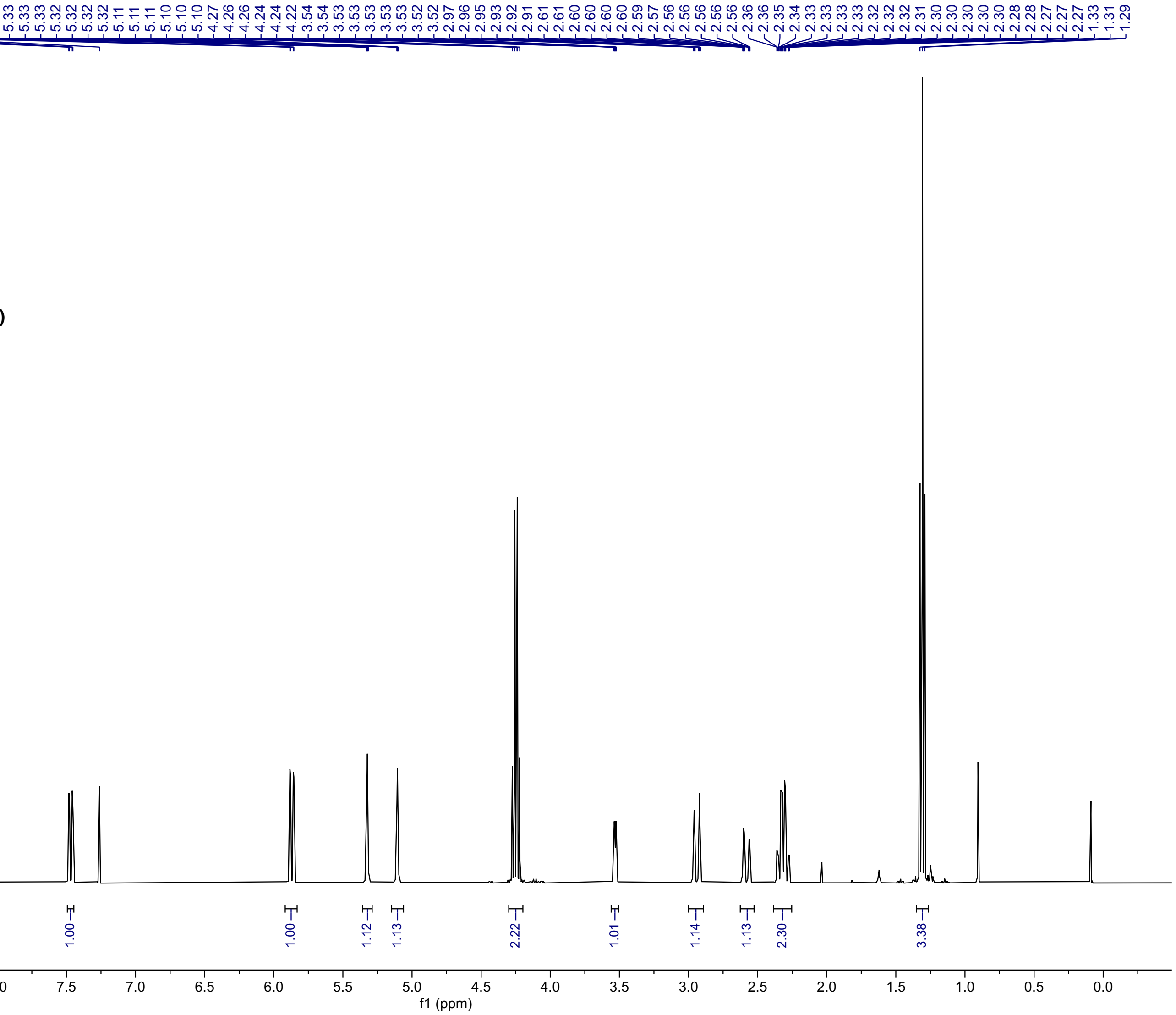

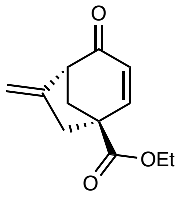

13

^{13}C NMR (101 MHz, CDCl$_3$)

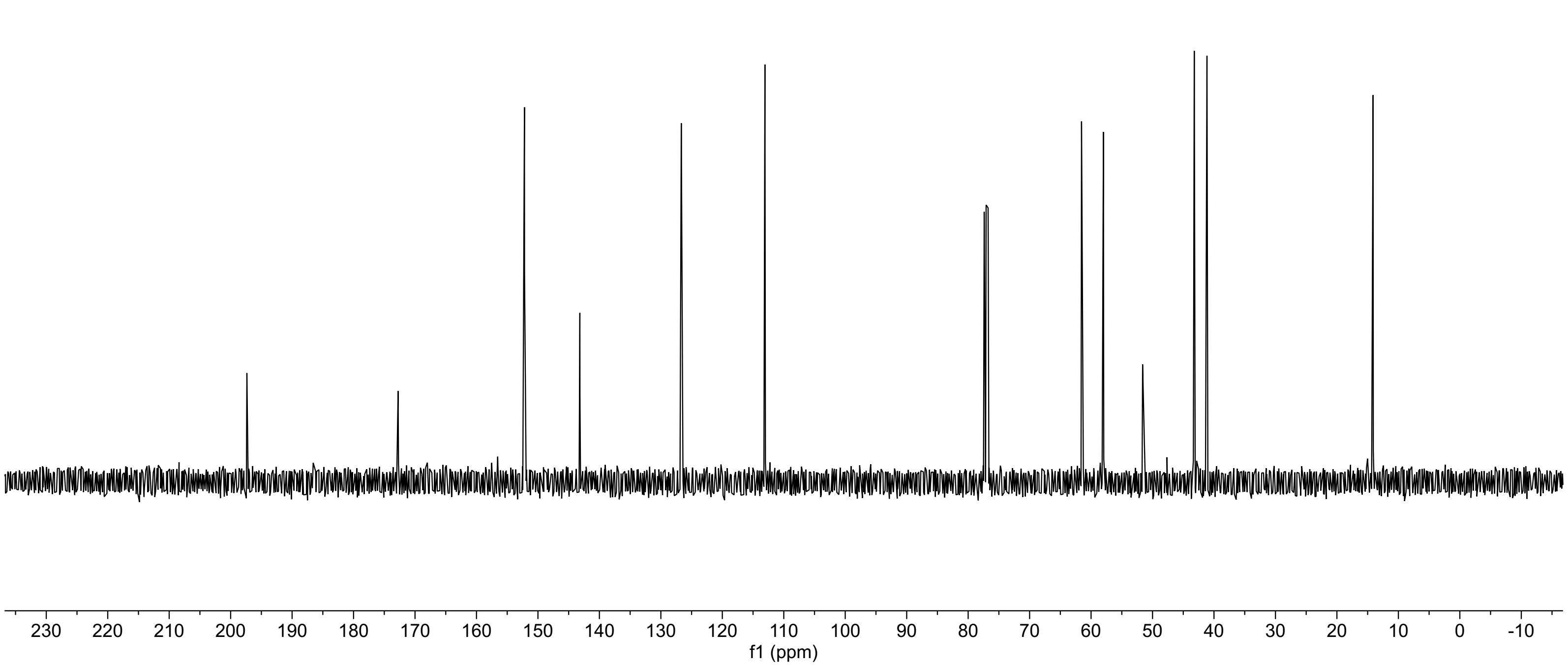

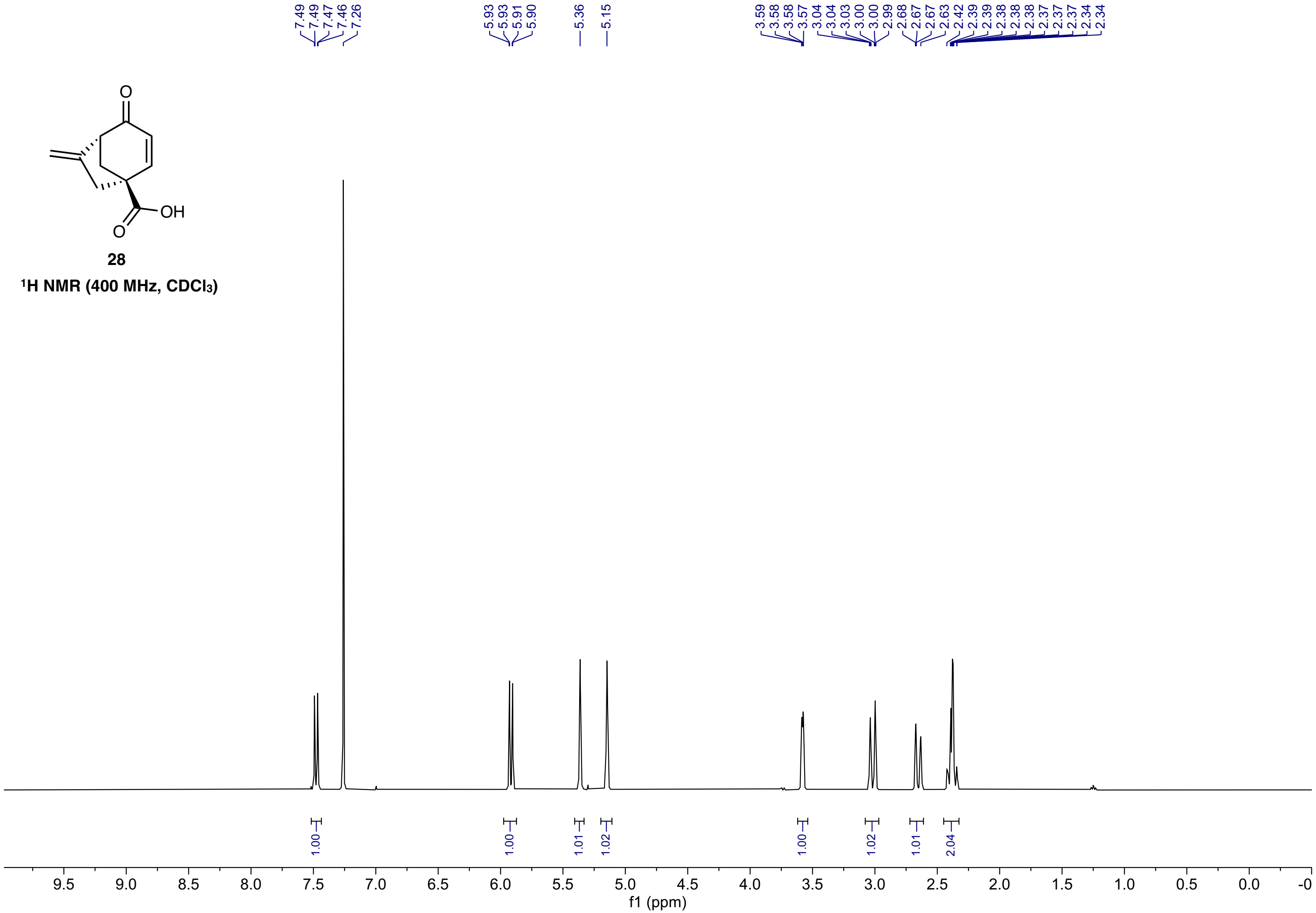

28

^{1}H NMR (400 MHz, $CDCl_3$)

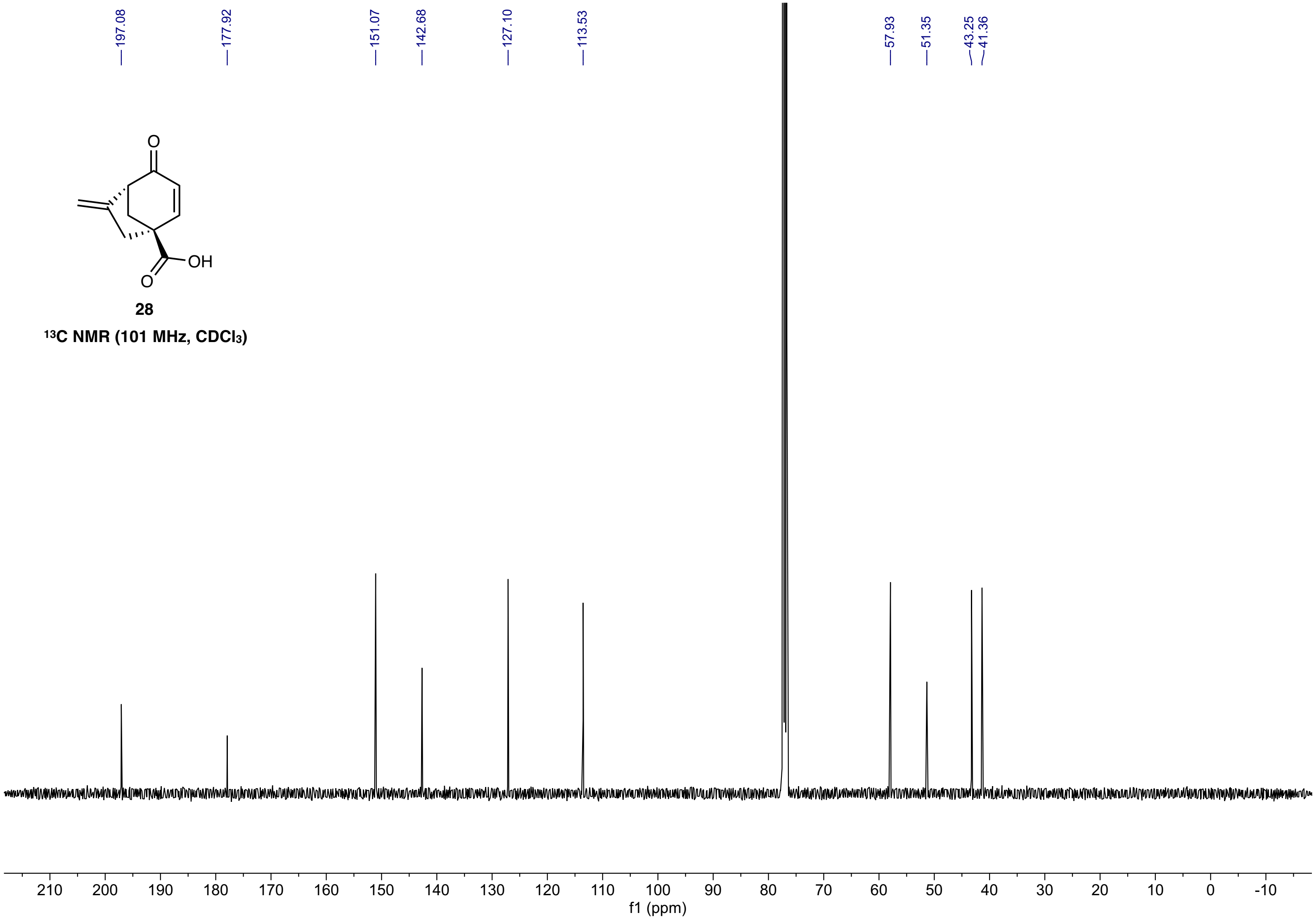

28

^{13}C NMR (101 MHz, $CDCl_3$)

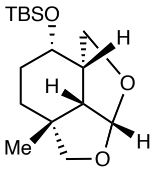

S38

^{1}H NMR (400 MHz, CDCl$_3$)

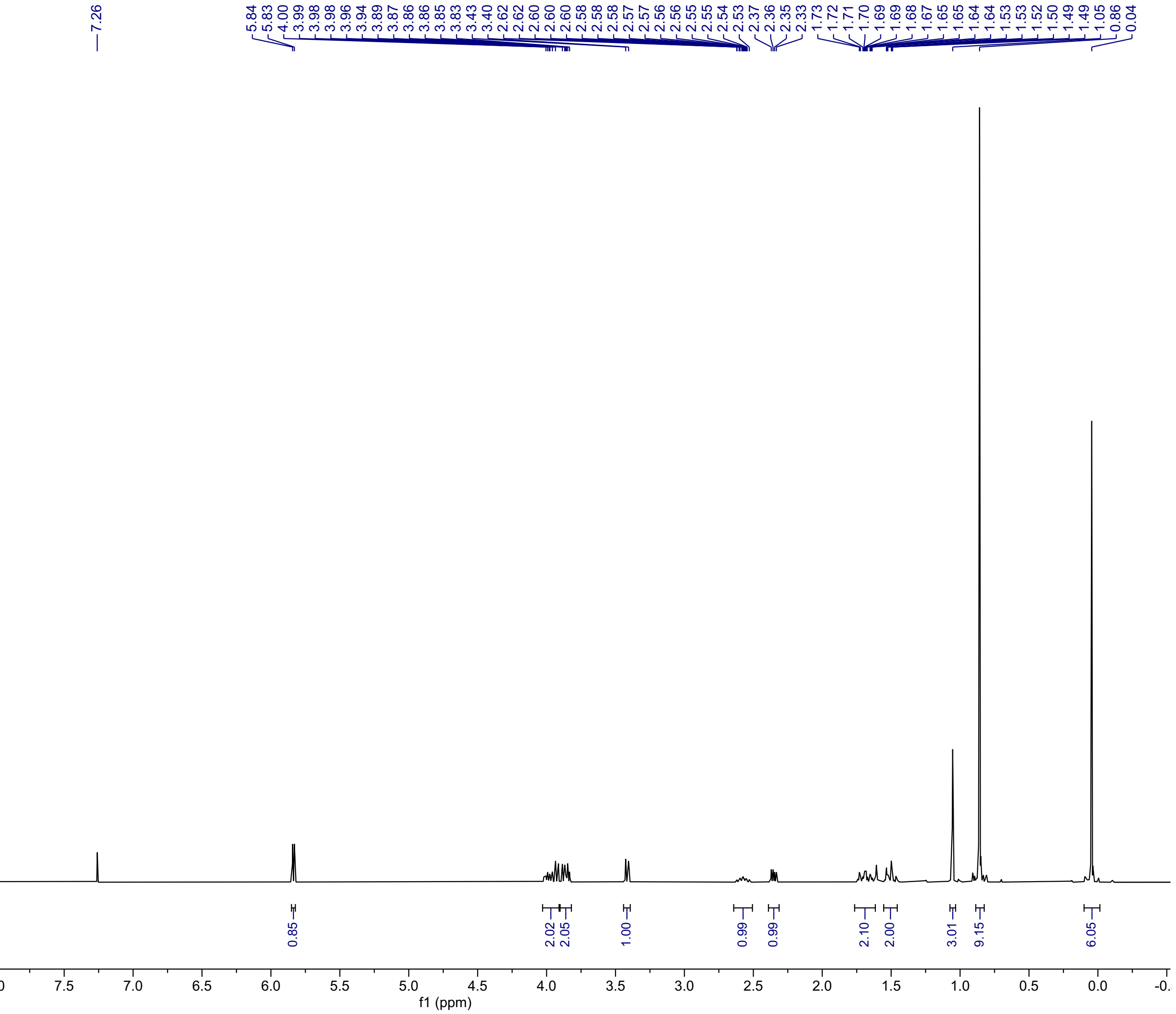

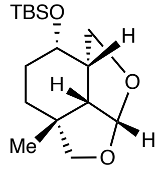

S38

^{13}C NMR (101 MHz, $CDCl_3$)

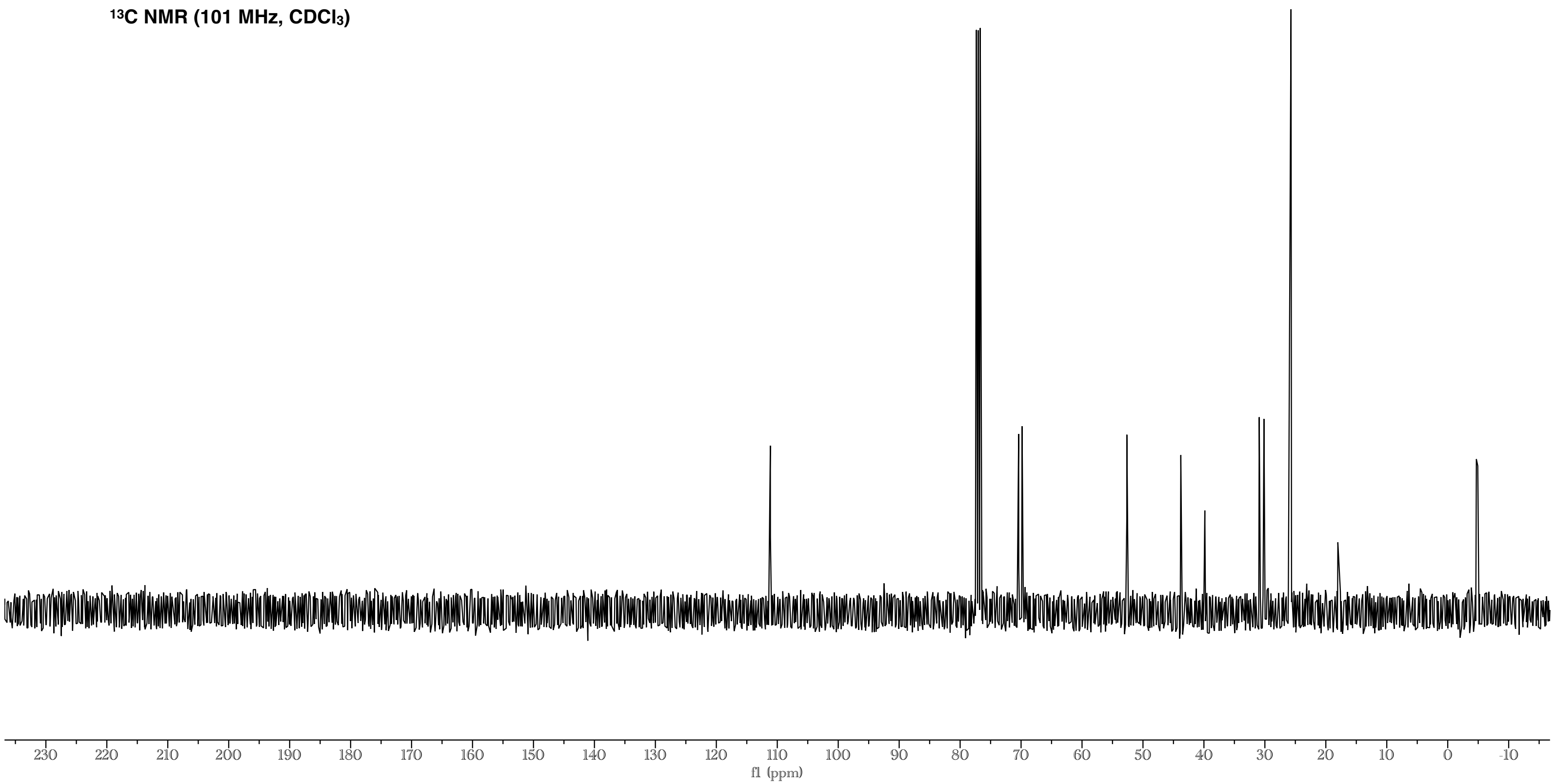

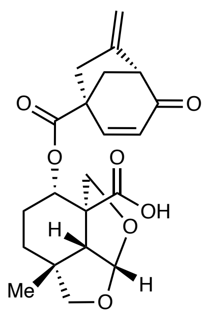

34

¹H NMR (400 MHz, CDCl₃)

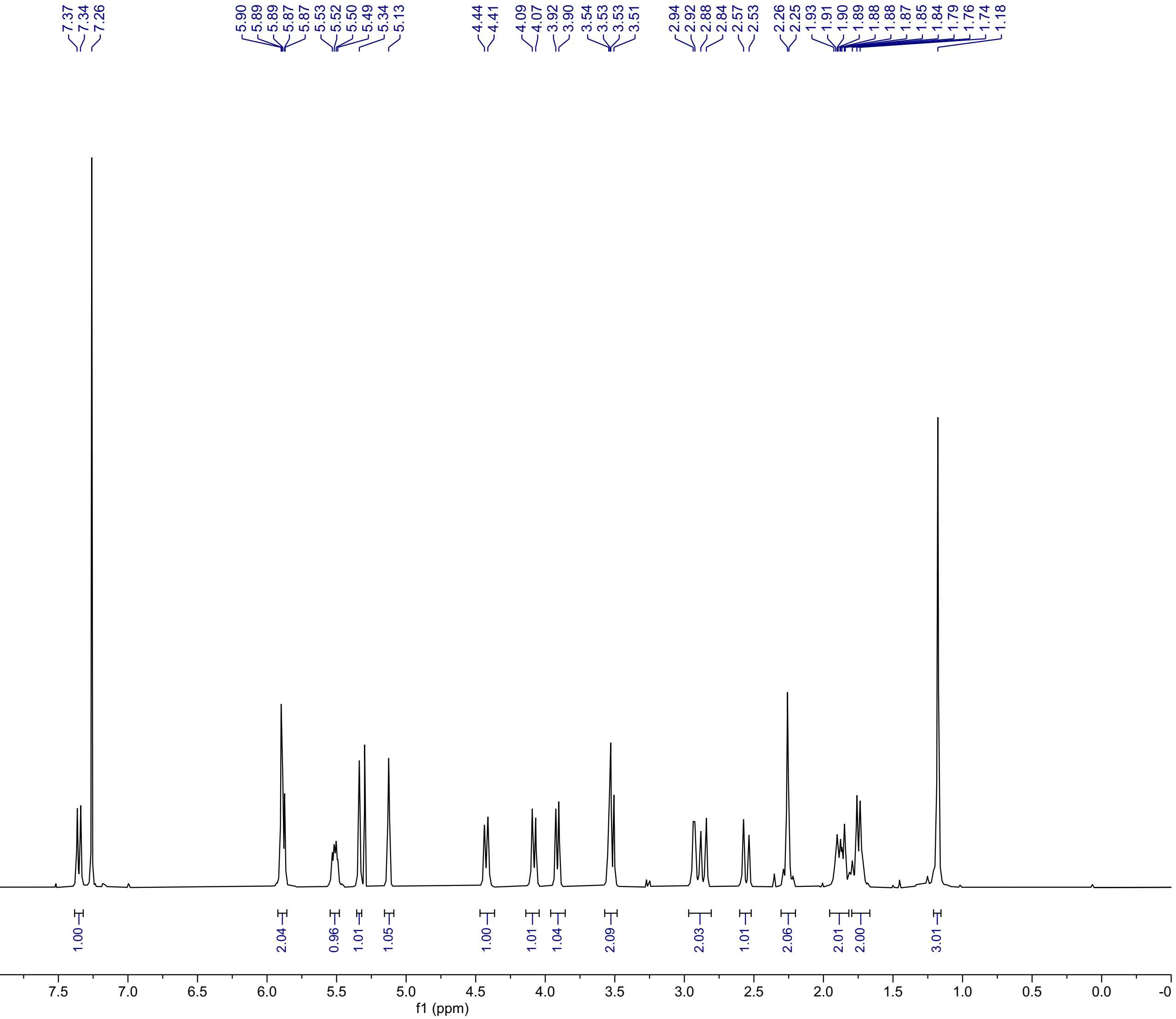

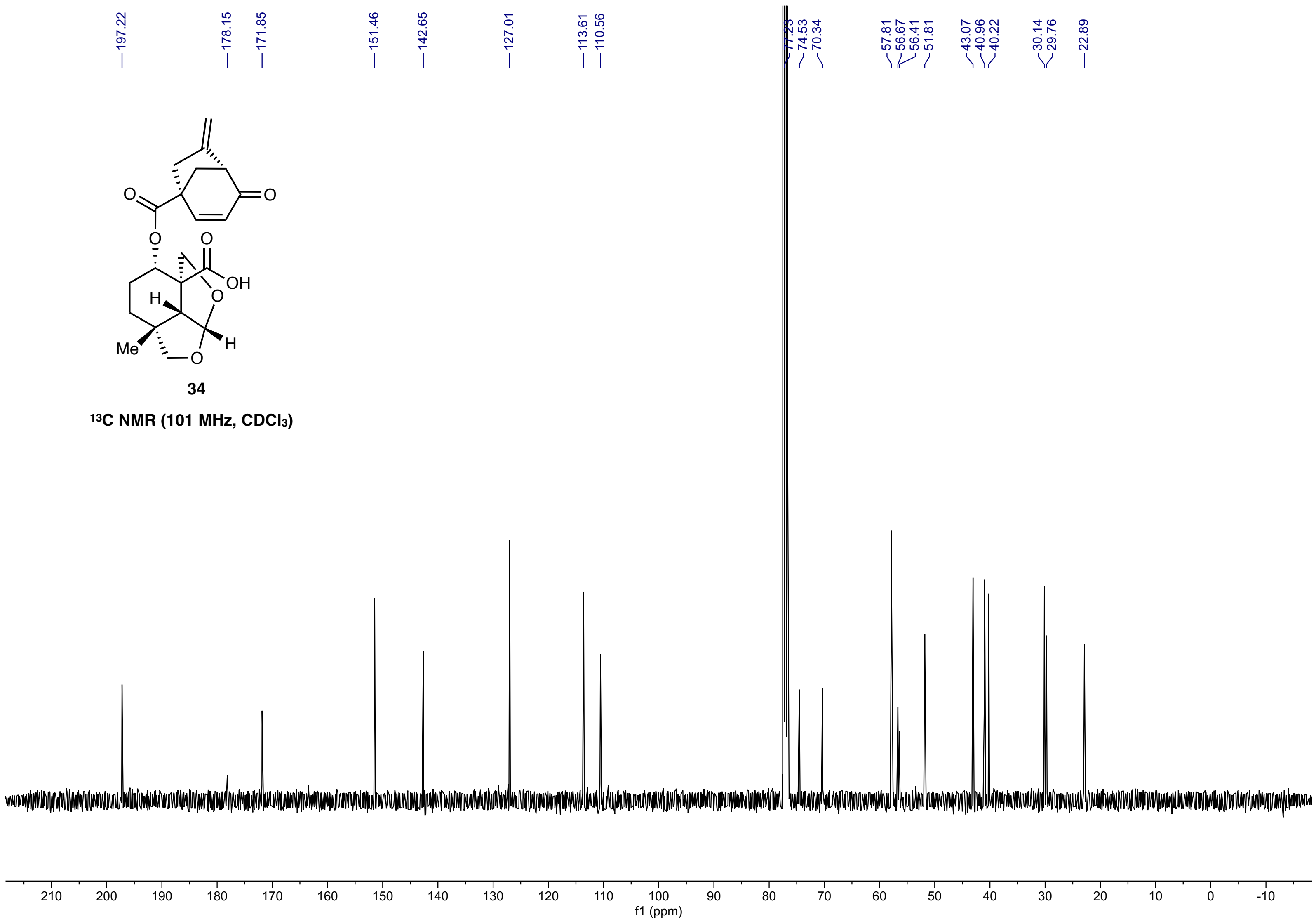
197.22
178.15
171.85
151.46
142.65
127.01
113.61
110.56
74.53
70.34
57.81
56.67
56.41
51.81
43.07
40.96
40.22
30.14
29.76
22.89
O
O
O
OH
H
H
Me
O
O
O
34
13C NMR (101 MHz, CDCl3)
210
200
190
180
170
160
150
140
130
120
110
100
90
80
70
60
50
40
30
20
10
0
-10
f1 (ppm)

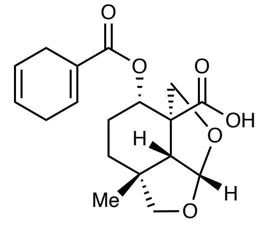

35

^{1}H NMR (400 MHz, CDCl$_3$)

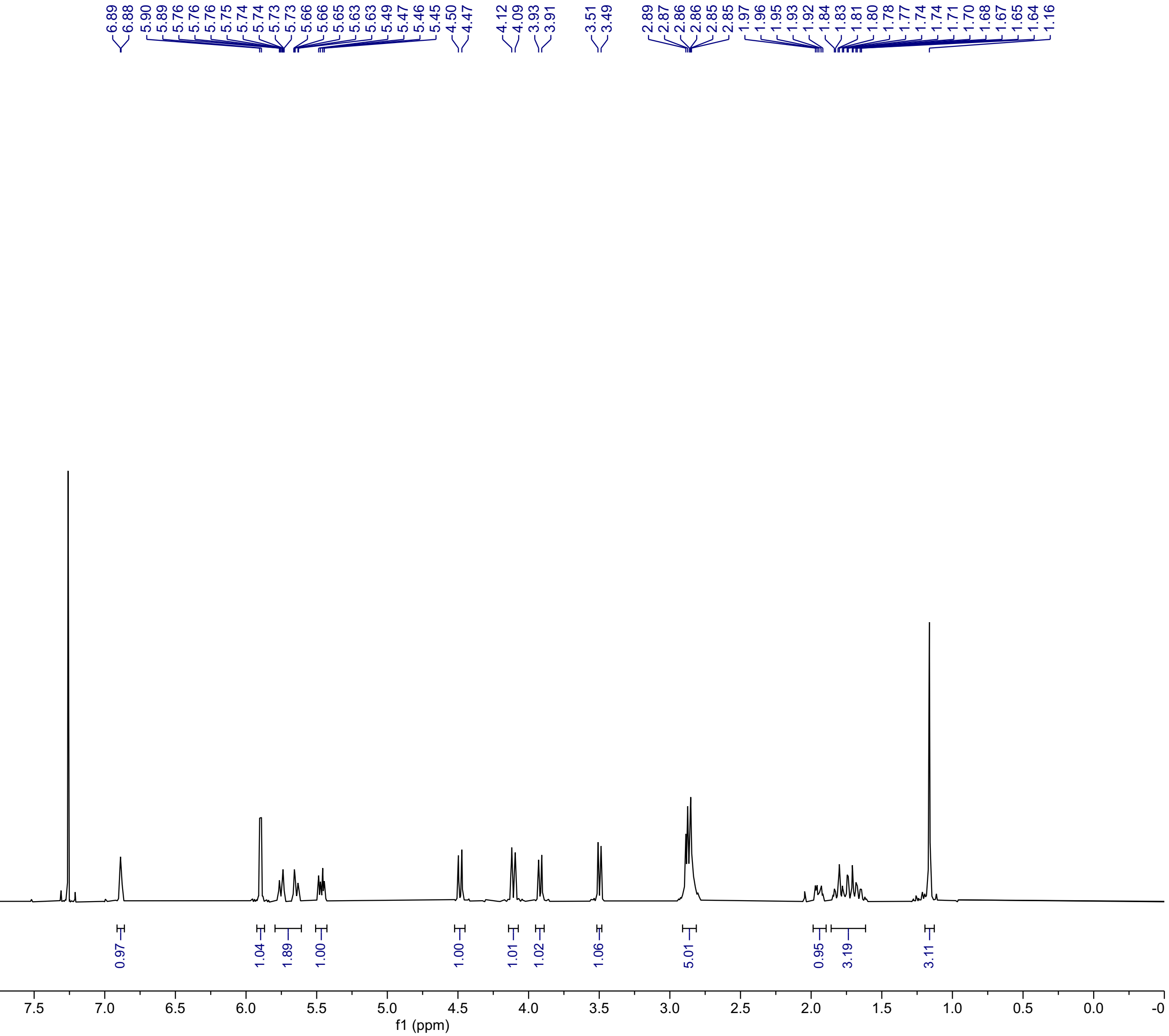

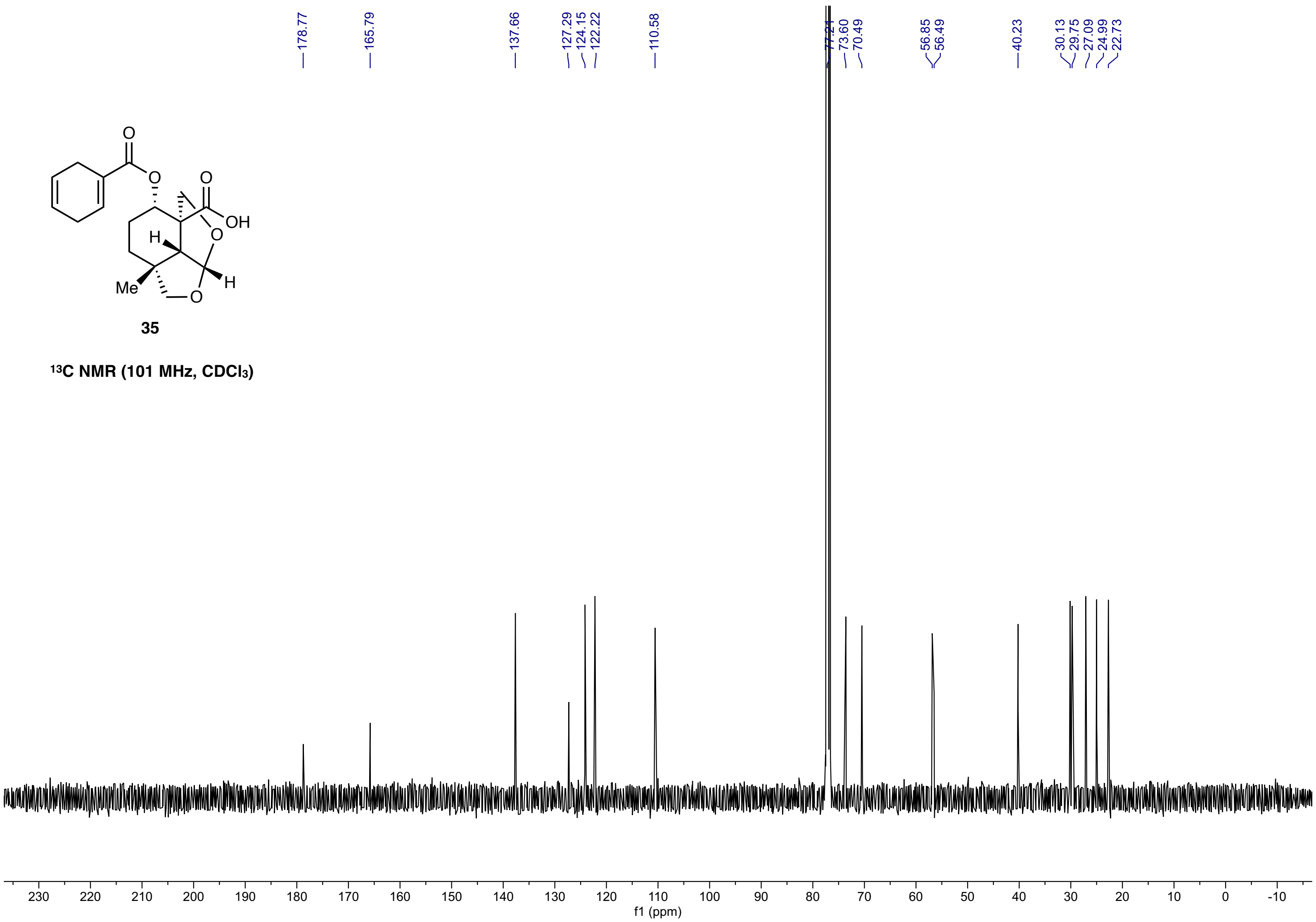

35

^{13}C NMR (101 MHz, CDCl$_3$)

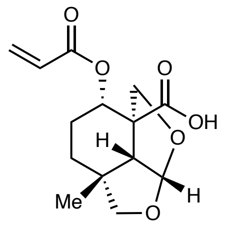

36

^{1}H NMR (400 MHz, $CDCl_3$)

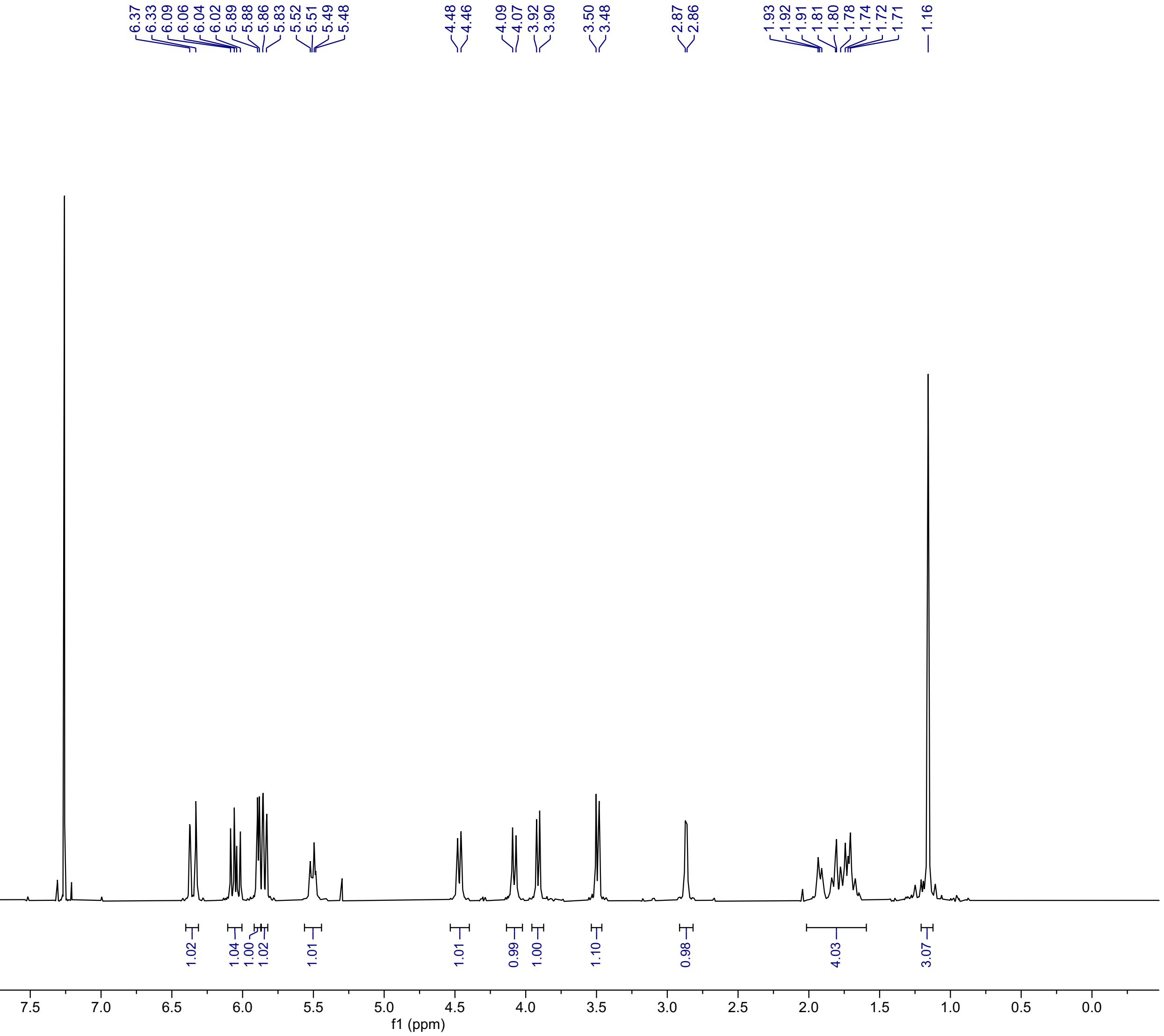

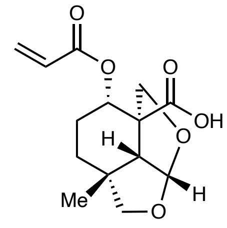

36

^{13}C NMR (101 MHz, CDCl$_3$)

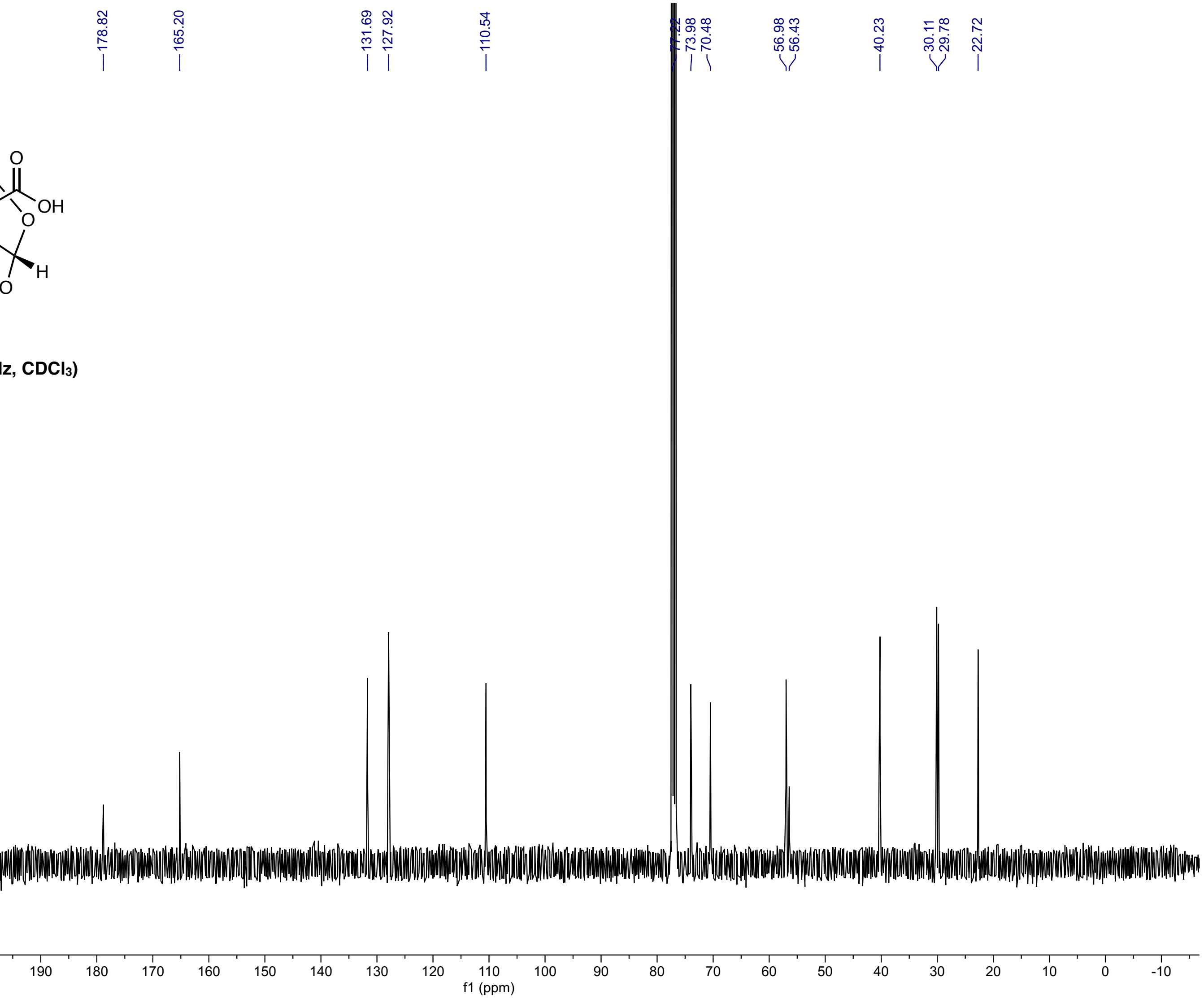

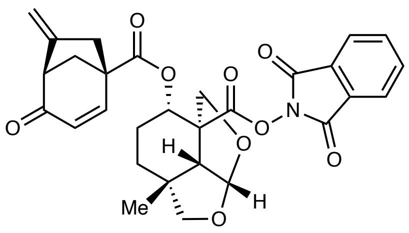

37

^{1}H NMR (400 MHz, CDCl$_3$)

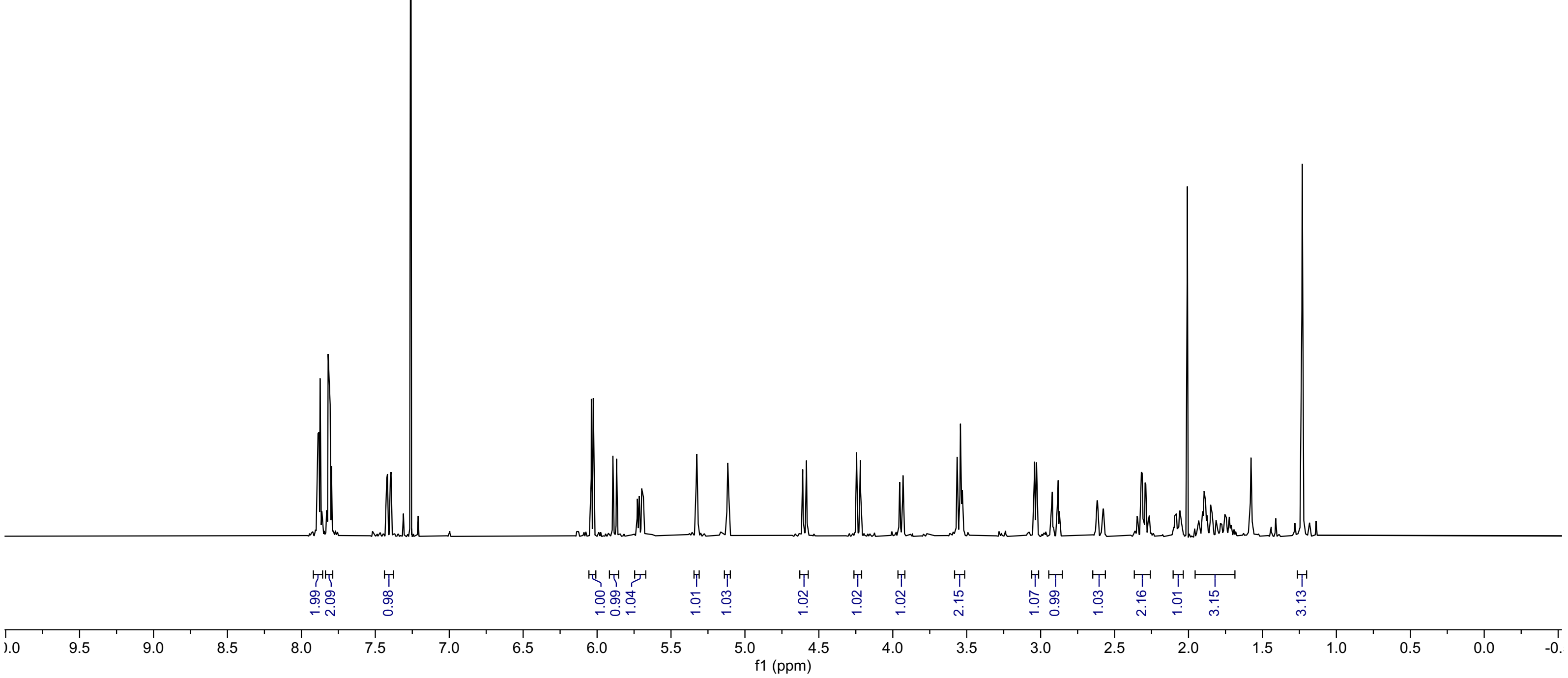

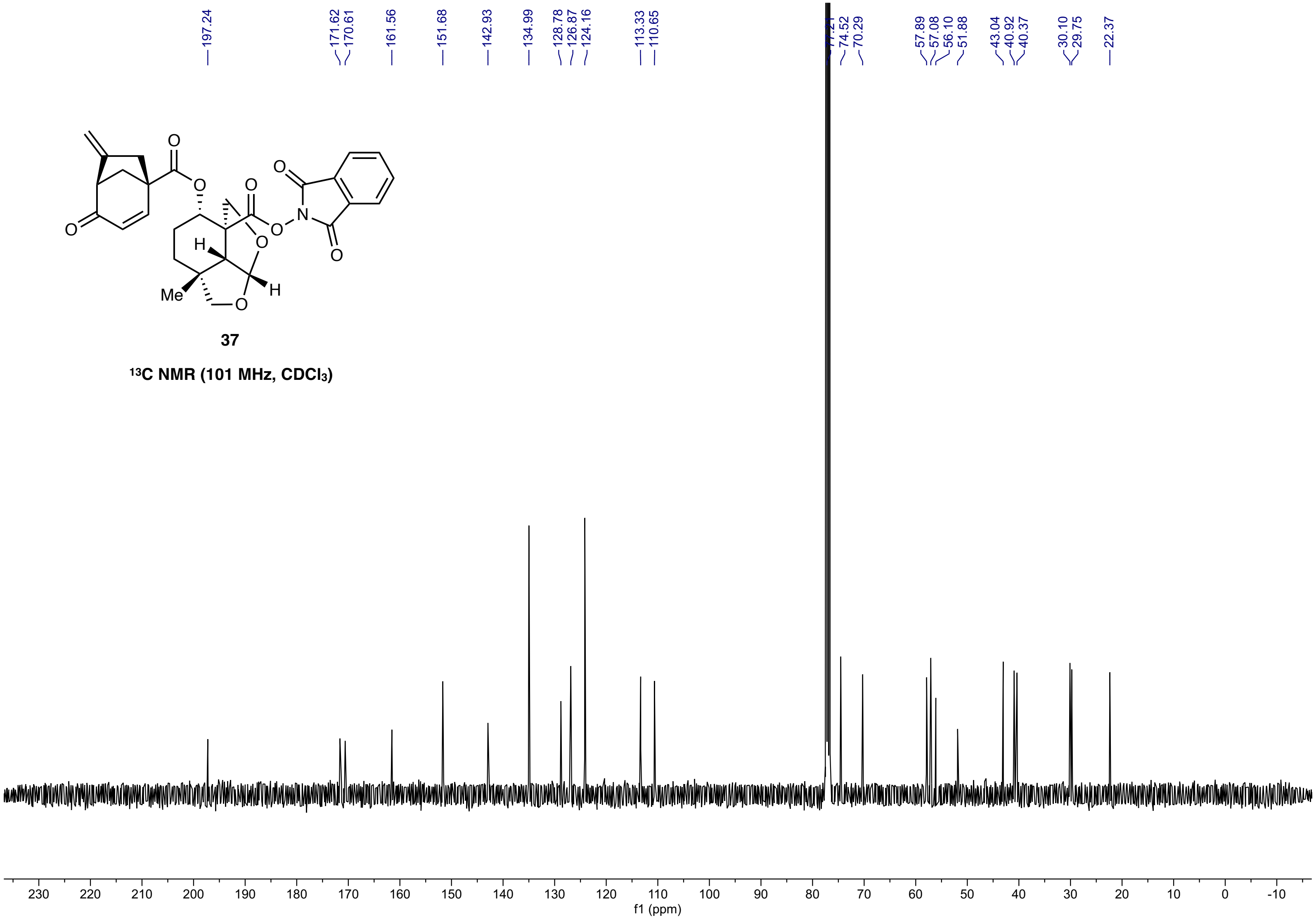

37

^{13}C NMR (101 MHz, $CDCl_3$)

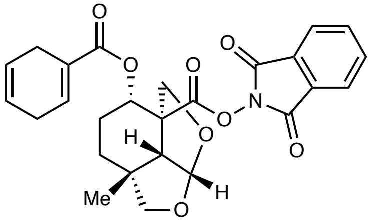

38

^{1}H NMR (400 MHz, $CDCl_3$)

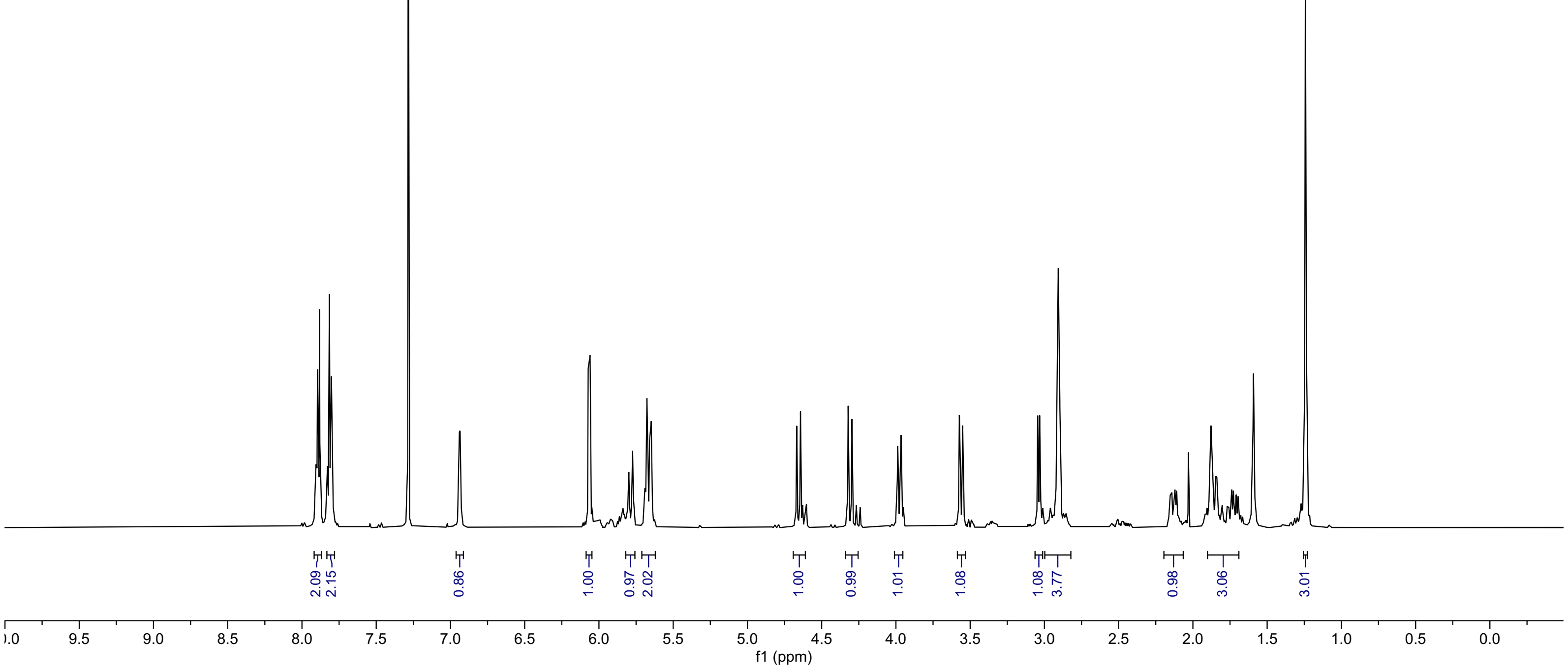

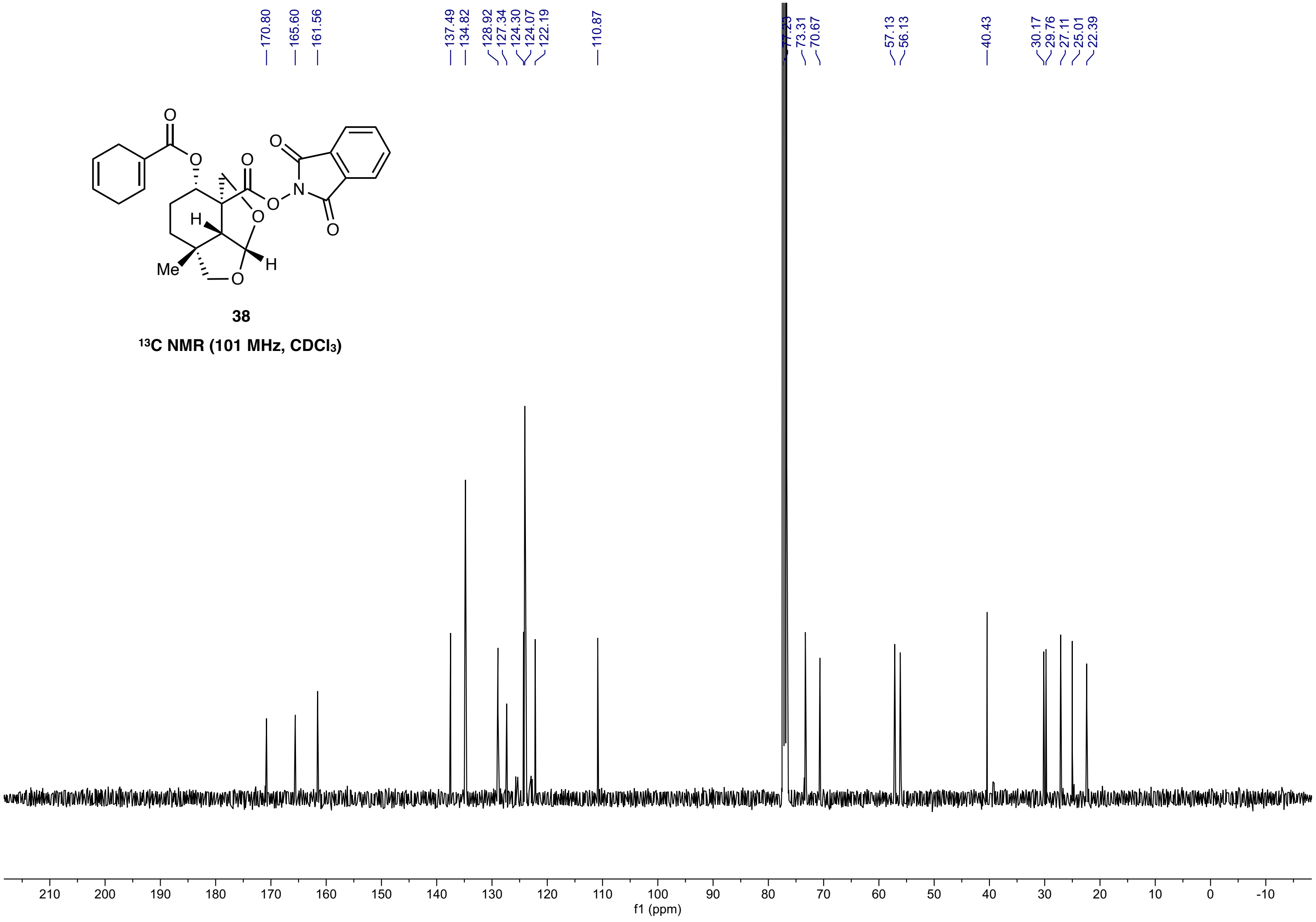

38

^{13}C NMR (101 MHz, CDCl$_3$)

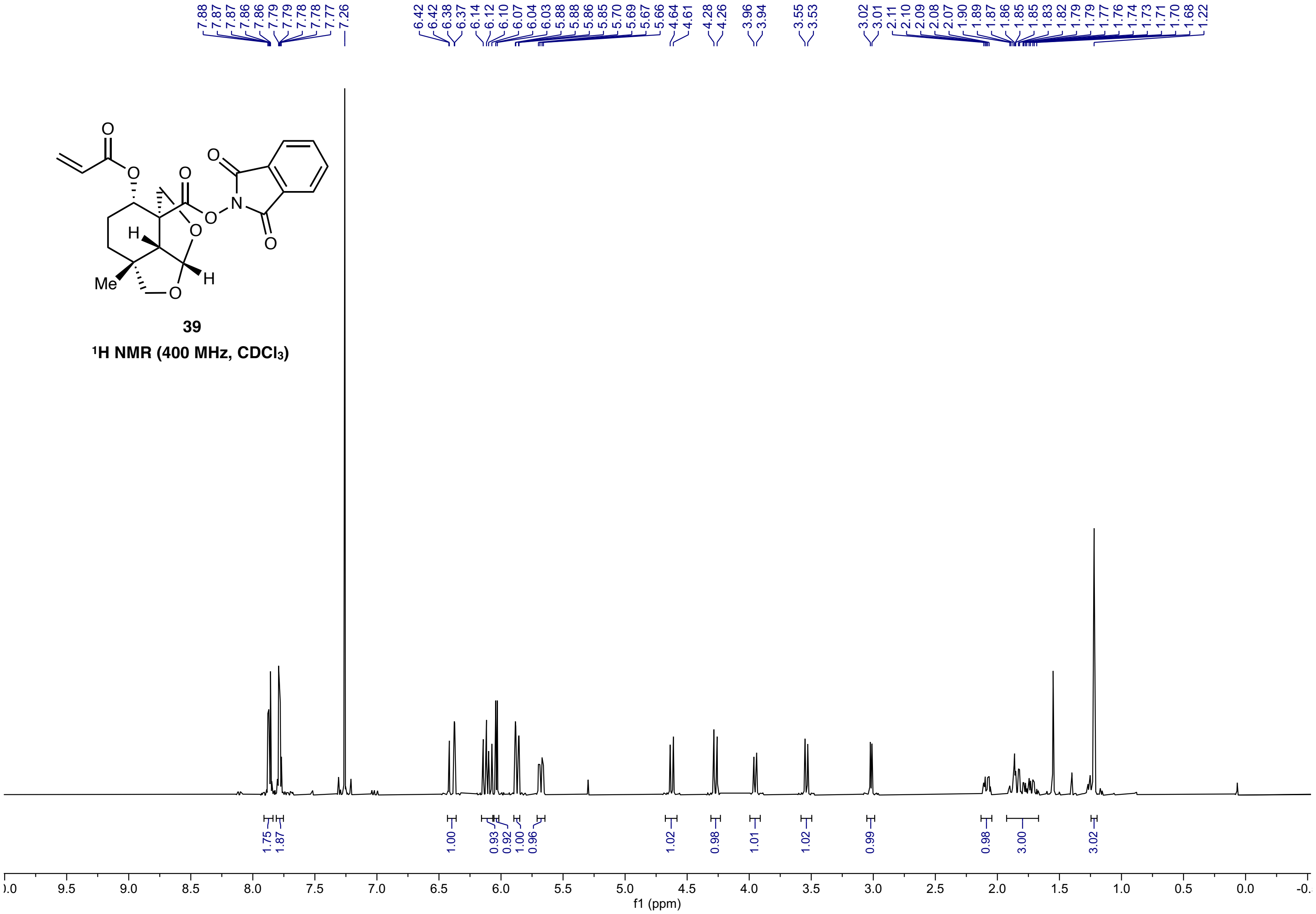
7.88 7.87 7.87 7.86 7.86 7.79 7.79 7.78 7.78 7.77 7.26
6.42 6.42 6.38 6.37 6.14 6.12 6.10 6.07 6.04 6.03 5.88 5.88 5.86 5.85 5.70 5.69 5.67 5.66 4.64 4.61 4.28 4.26 3.96 3.94 3.55 3.53
3.02 3.01 2.11 2.10 2.09 2.08 2.07 1.90 1.89 1.87 1.86 1.85 1.85 1.83 1.82 1.79 1.79 1.77 1.76 1.74 1.73 1.71 1.70 1.68 1.22
O
O
O
O
N
O
O
H
O
Me
O
H
O
39
¹H NMR (400 MHz, CDCl₃)
1.75 1.87 1.00 0.93 0.92 1.00 0.96 1.02 0.98 1.01 1.02 0.99 0.98 3.00 3.02
).0 9.5 9.0 8.5 8.0 7.5 7.0 6.5 6.0 5.5 5.0 4.5 4.0 3.5 3.0 2.5 2.0 1.5 1.0 0.5 0.0 -0.
f1 (ppm)

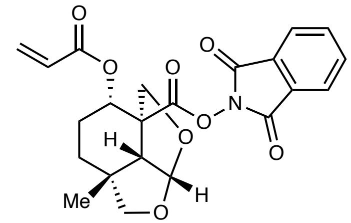

39

^{13}C NMR (101 MHz, CDCl$_3$)

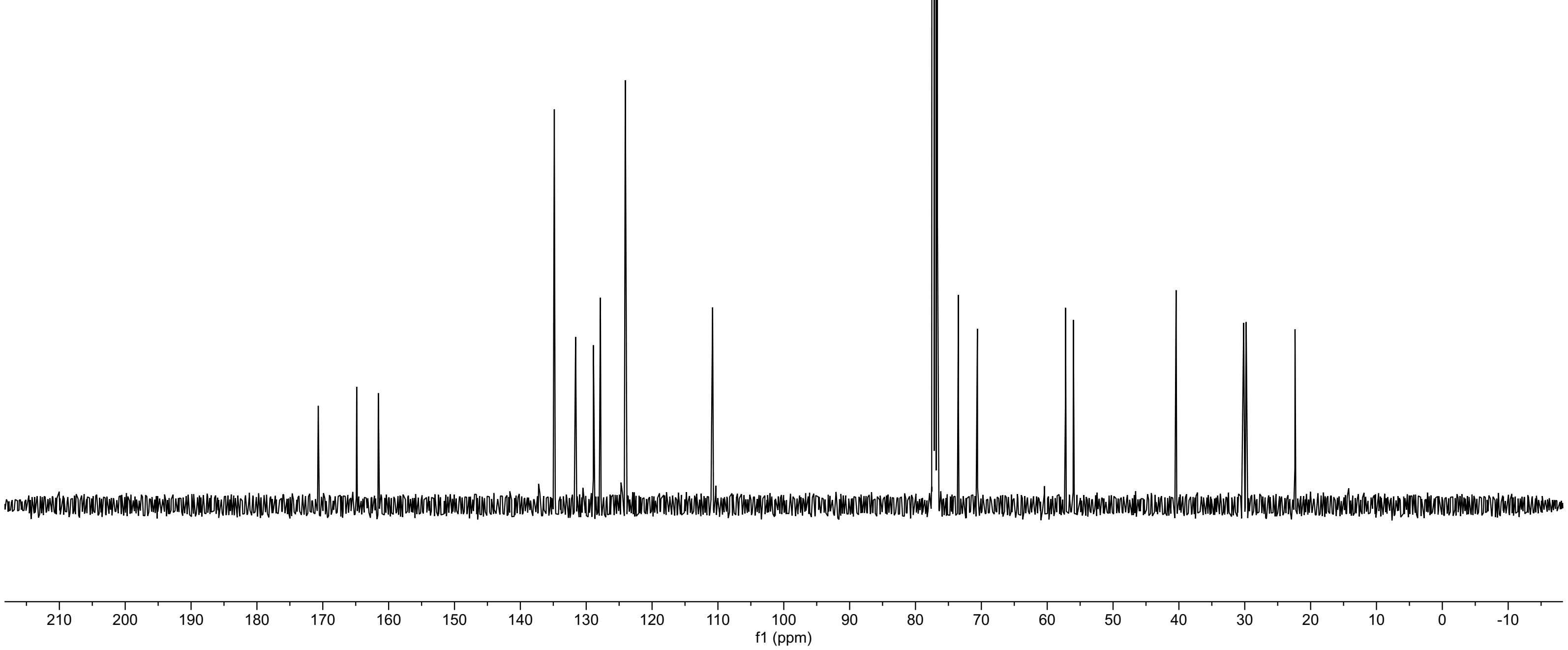

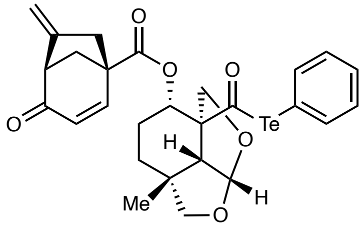

S43

^{1}H NMR (500 MHz, CDCl$_3$)

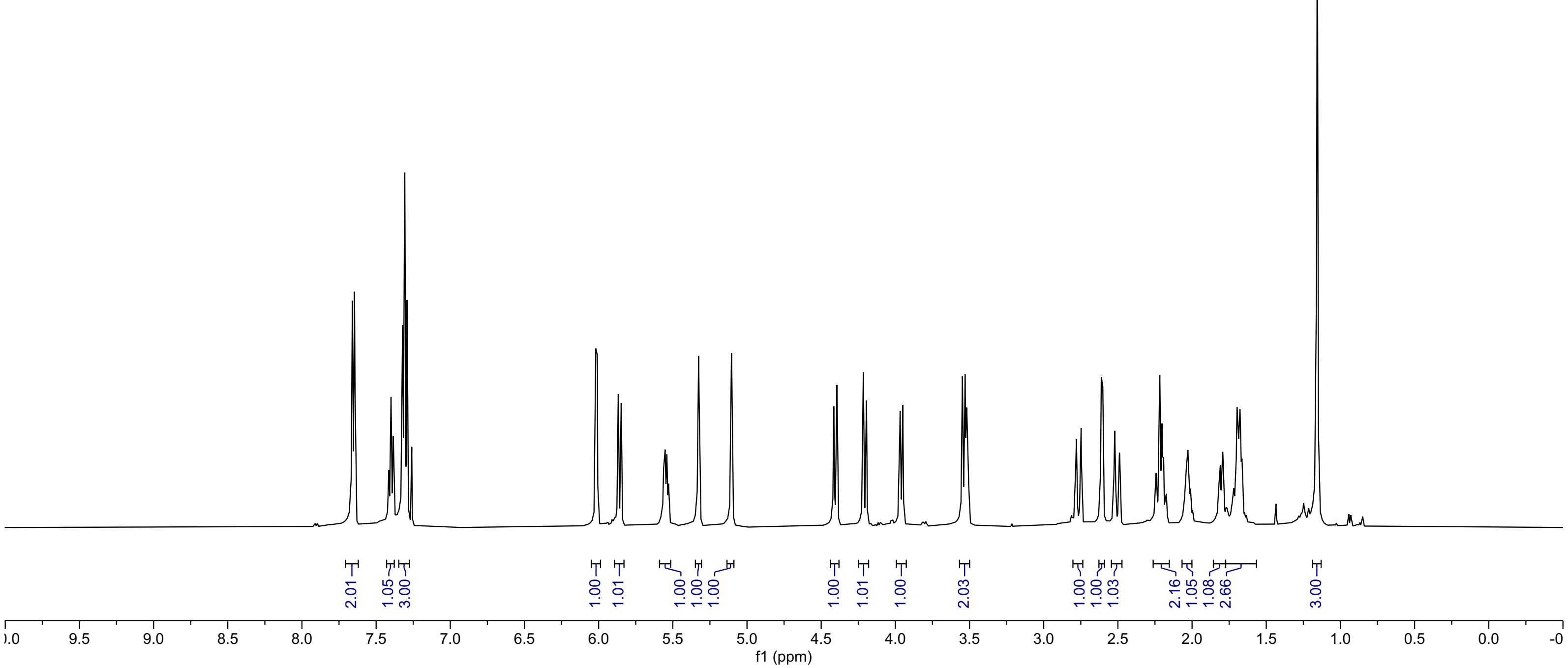

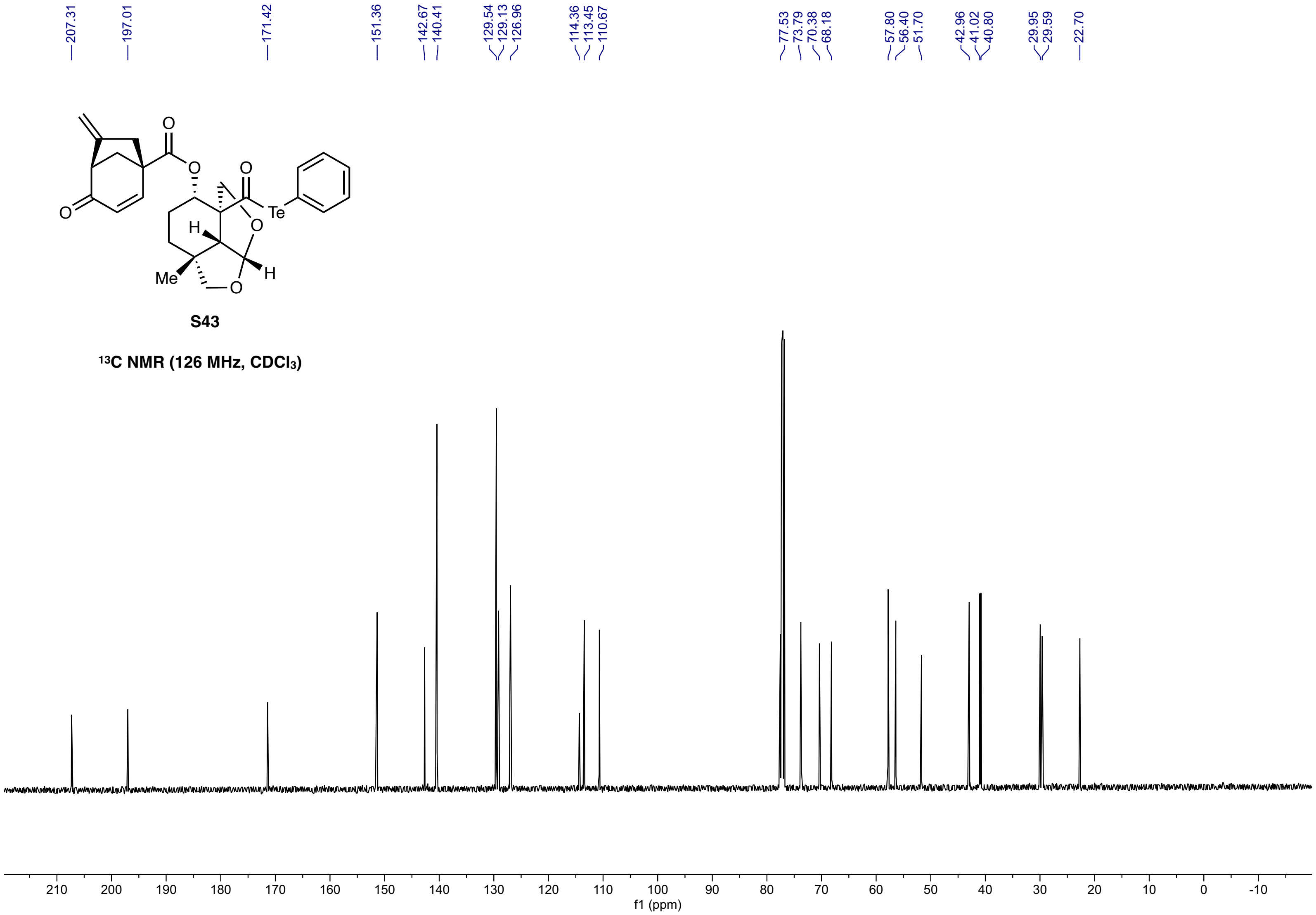

S43

^{13}C NMR (126 MHz, $CDCl_3$)

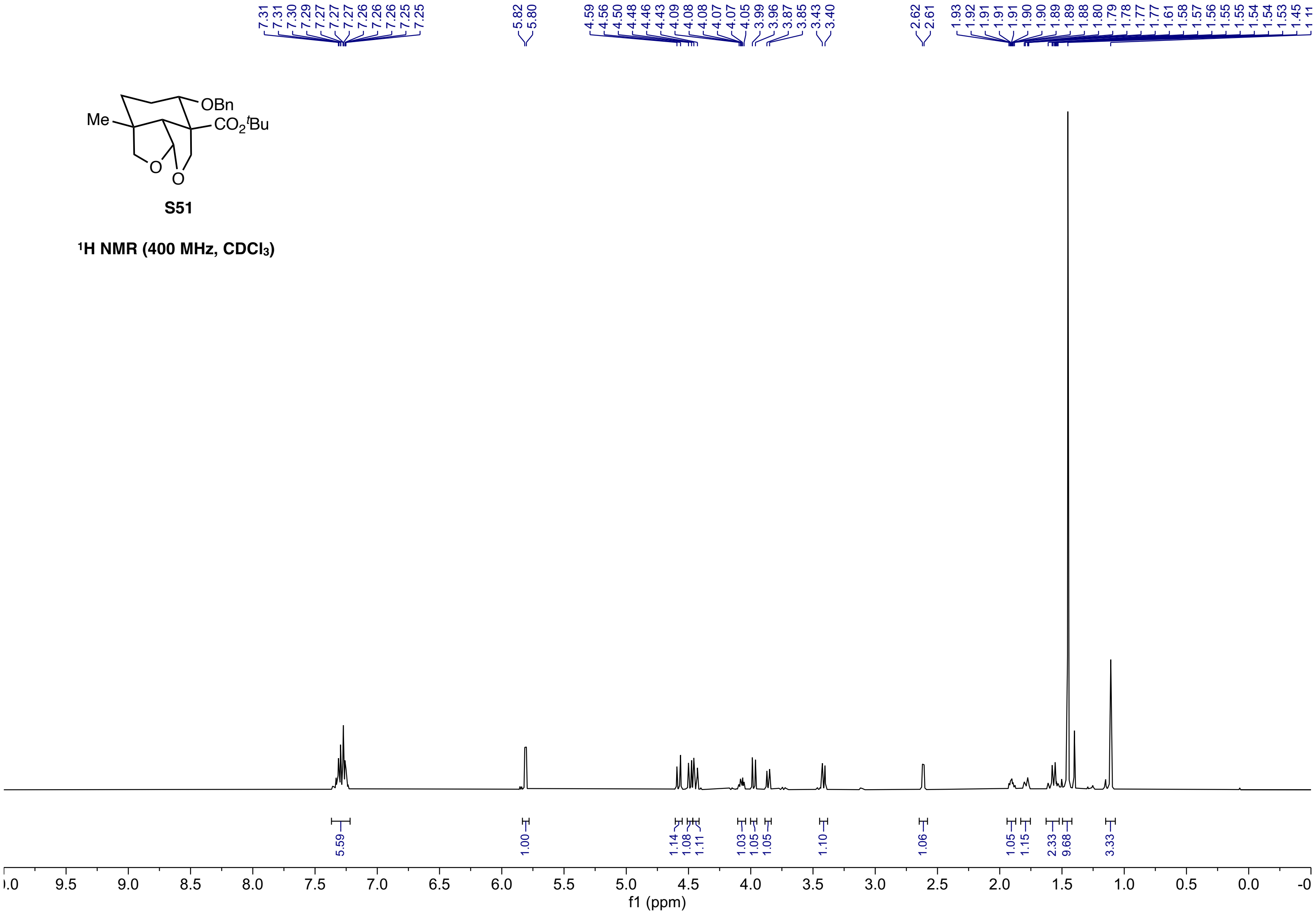
OBn
Me
CO2tBu
O
O
S51
1H NMR (400 MHz, CDCl3)
7.31 7.31 7.30 7.29 7.27 7.27 7.27 7.26 7.26 7.26 7.25 7.25
5.82 5.80
4.59 4.56 4.50 4.48 4.46 4.43 4.09 4.08 4.08 4.07 4.05 3.99 3.96 3.87 3.85 3.43 3.40
2.62 2.61
1.93 1.92 1.91 1.91 1.90 1.90 1.89 1.89 1.88 1.80 1.79 1.78 1.77 1.77 1.61 1.58 1.57 1.56 1.55 1.55 1.54 1.54 1.53 1.45 1.11
5.59 1.00 1.14 1.08 1.11 1.03 1.05 1.05 1.10 1.06 1.05 1.15 2.33 9.68 3.33
f1 (ppm)

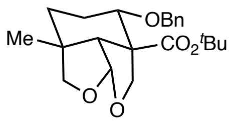

S51

^{13}C NMR (101 MHz, CDCl$_3$)

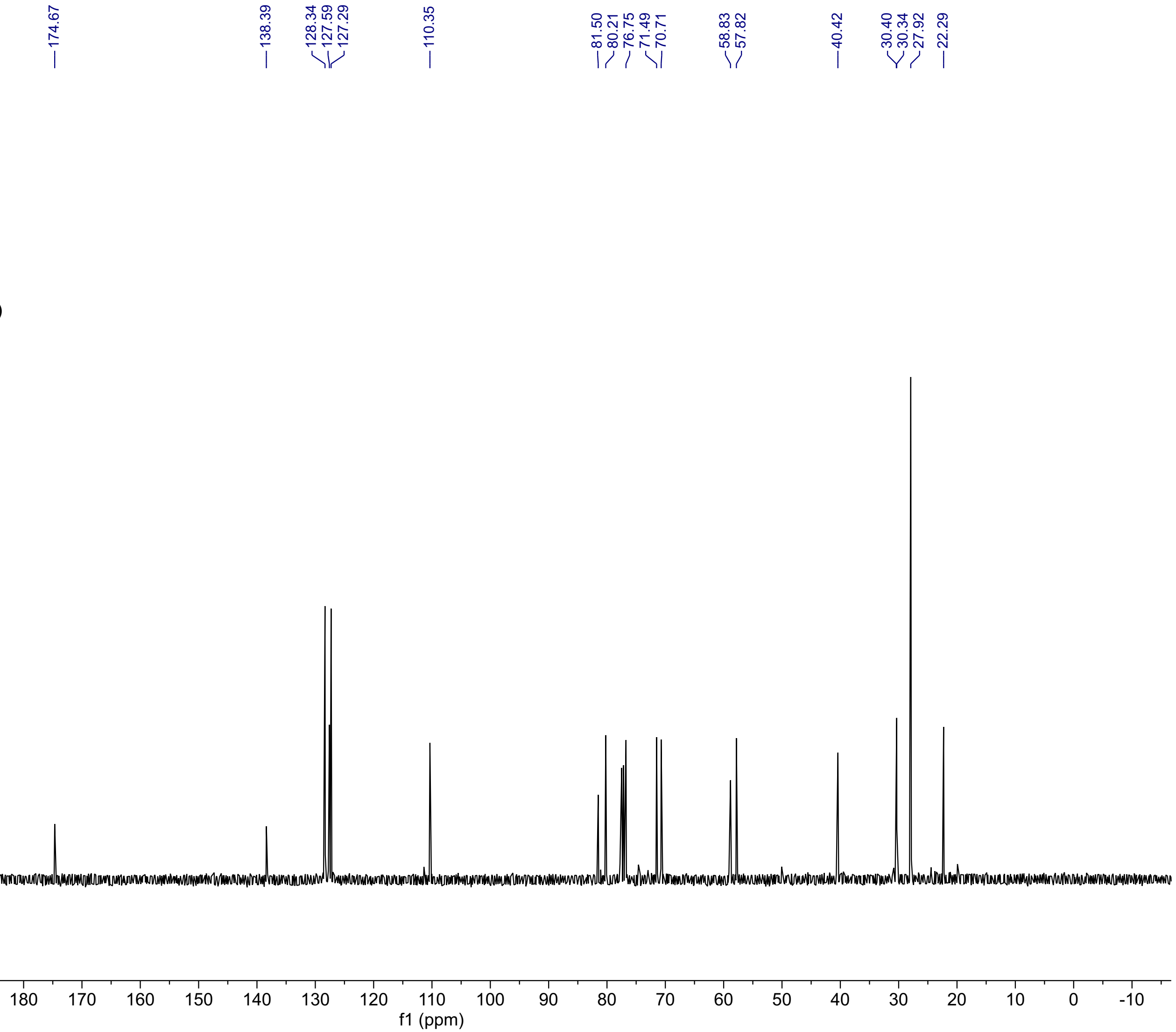

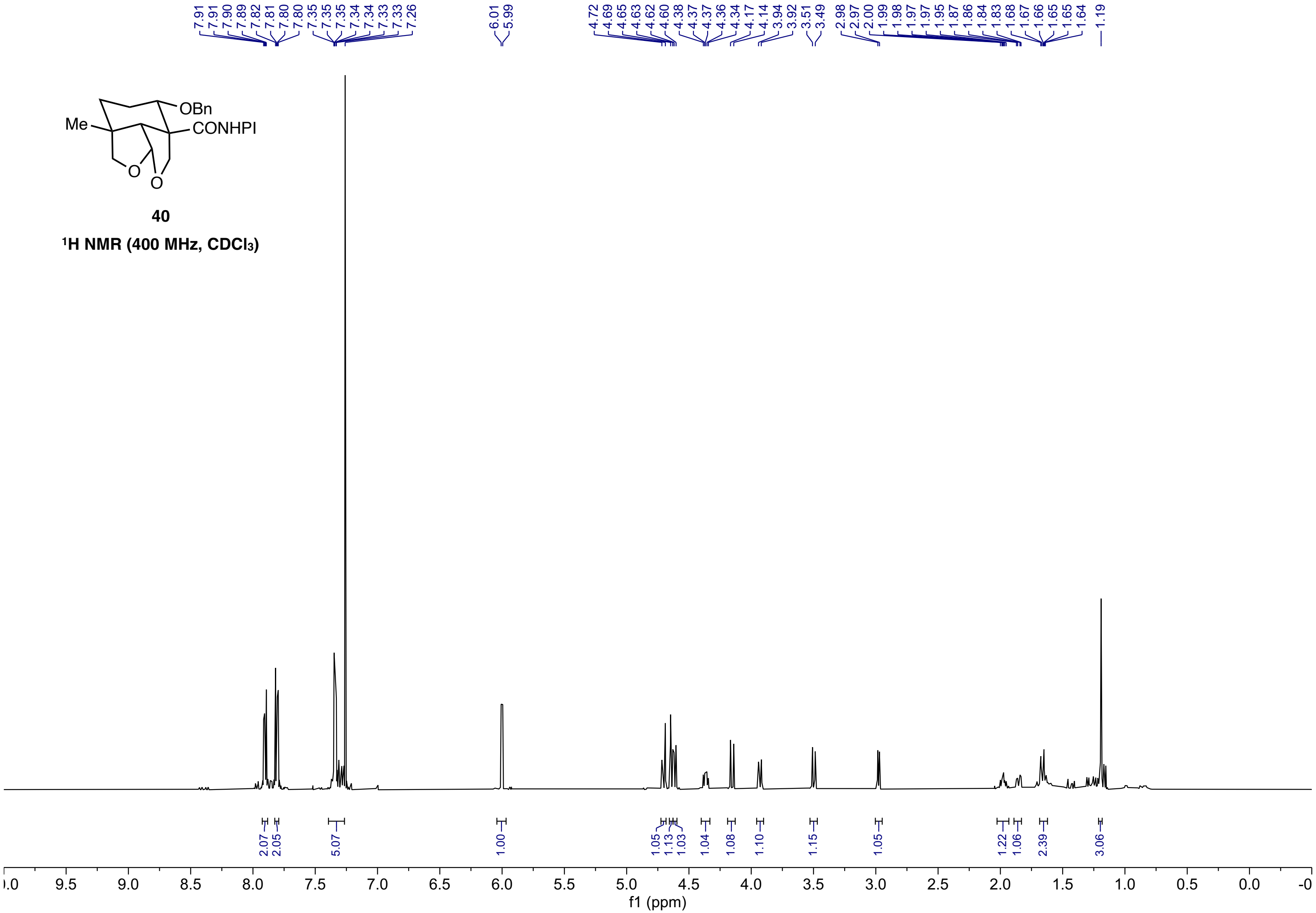
OBn
Me
CONHPI
O
O
40
¹H NMR (400 MHz, CDCl₃)
f1 (ppm)

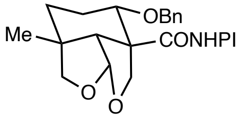

40

^{13}C NMR (101 MHz, CDCl$_3$)

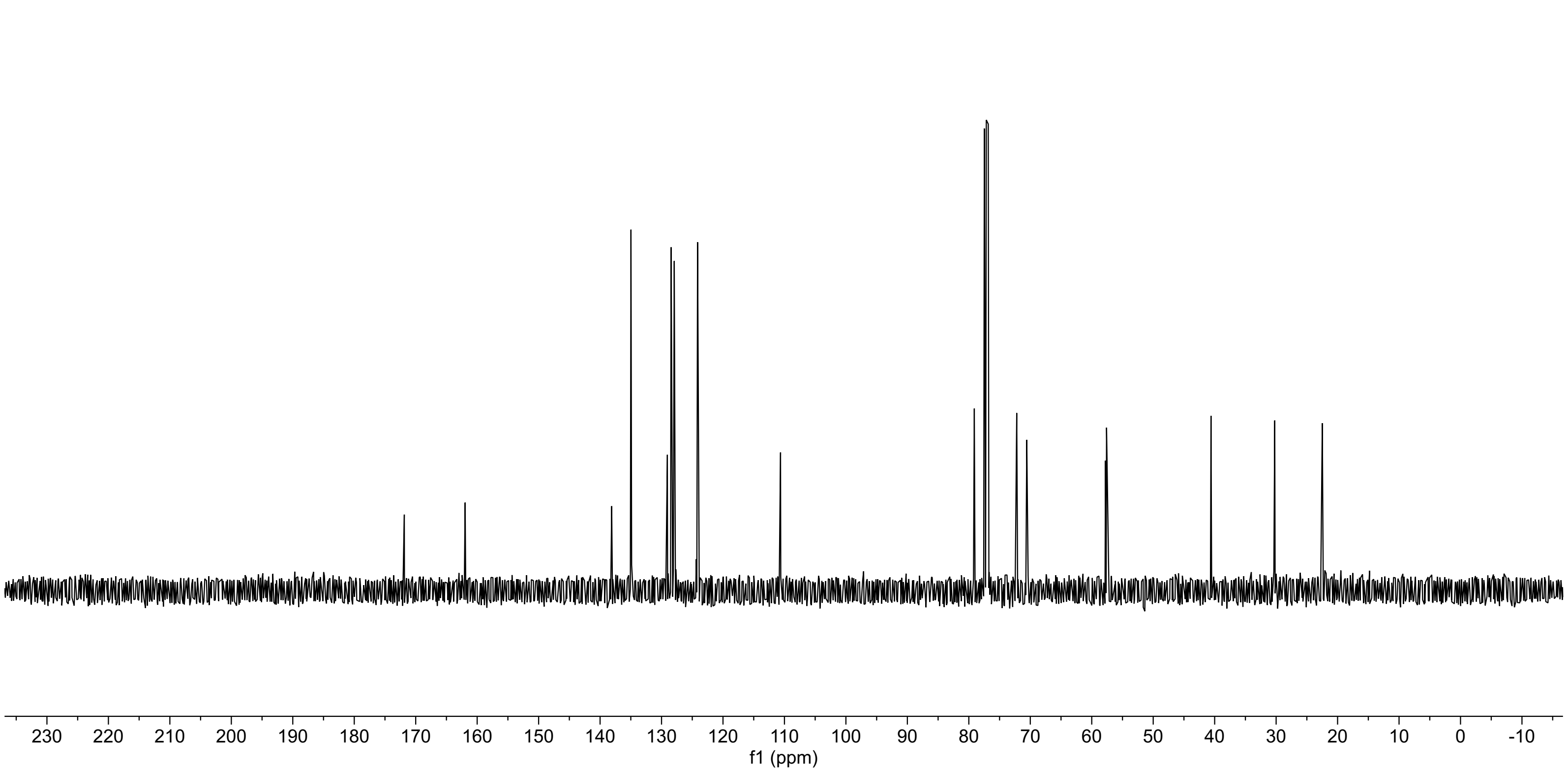

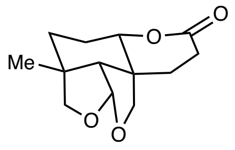

42

^{1}H NMR (400 MHz, CDCl3)

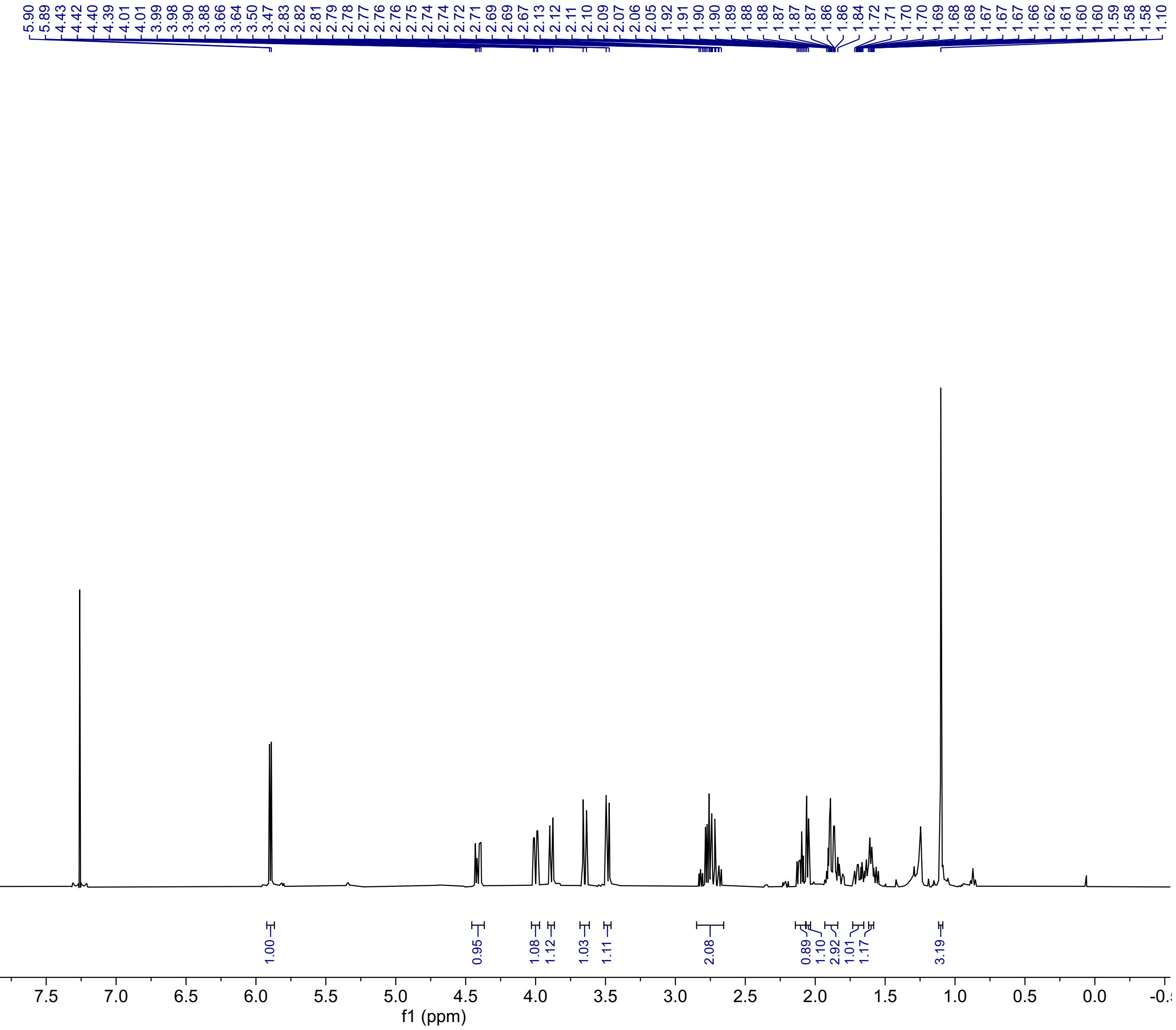

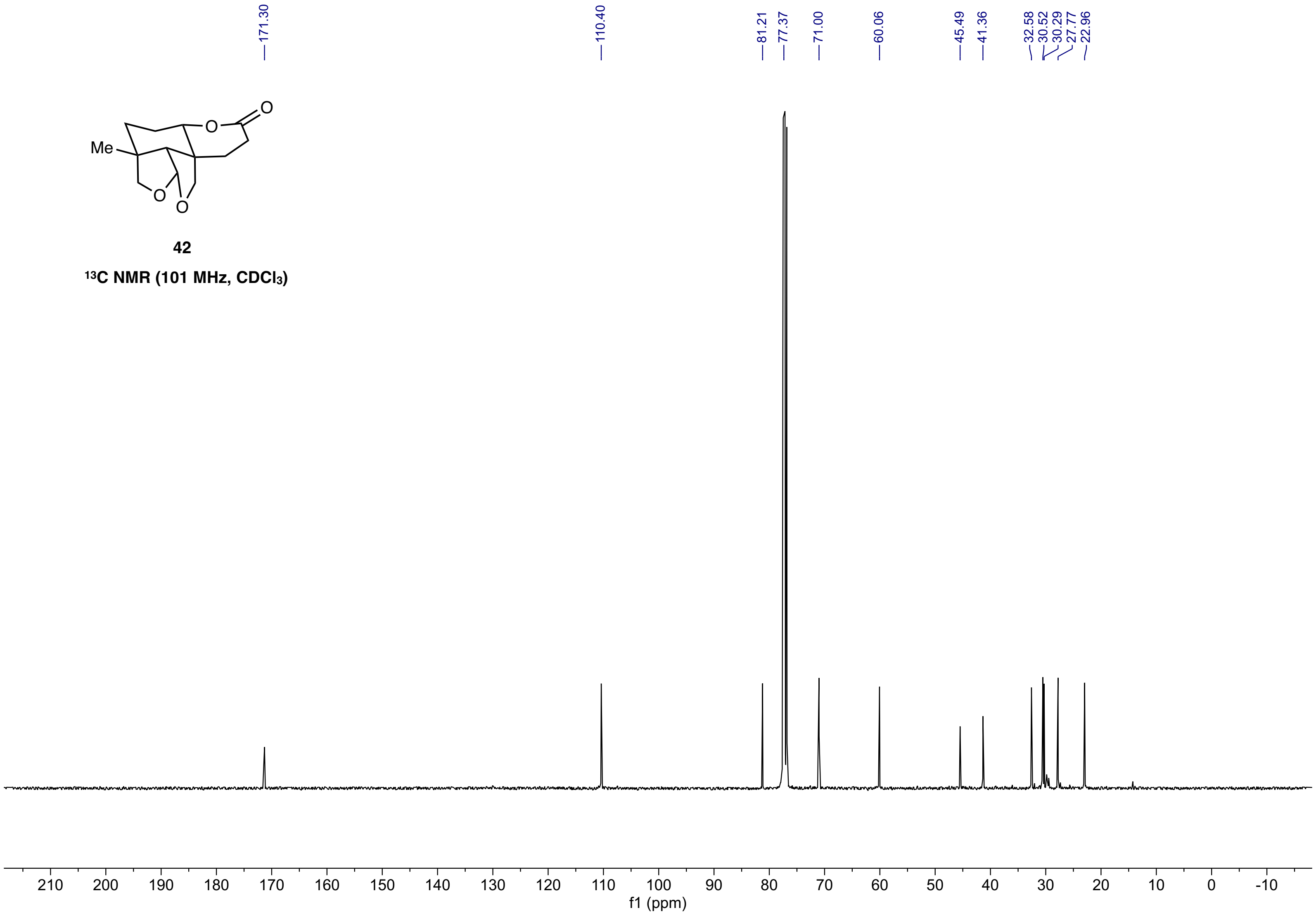

42

^{13}C NMR (101 MHz, $CDCl_3$)

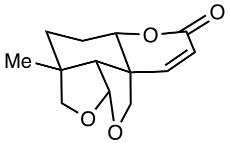

S53

^{1}H NMR (400 MHz, $CDCl_3$)

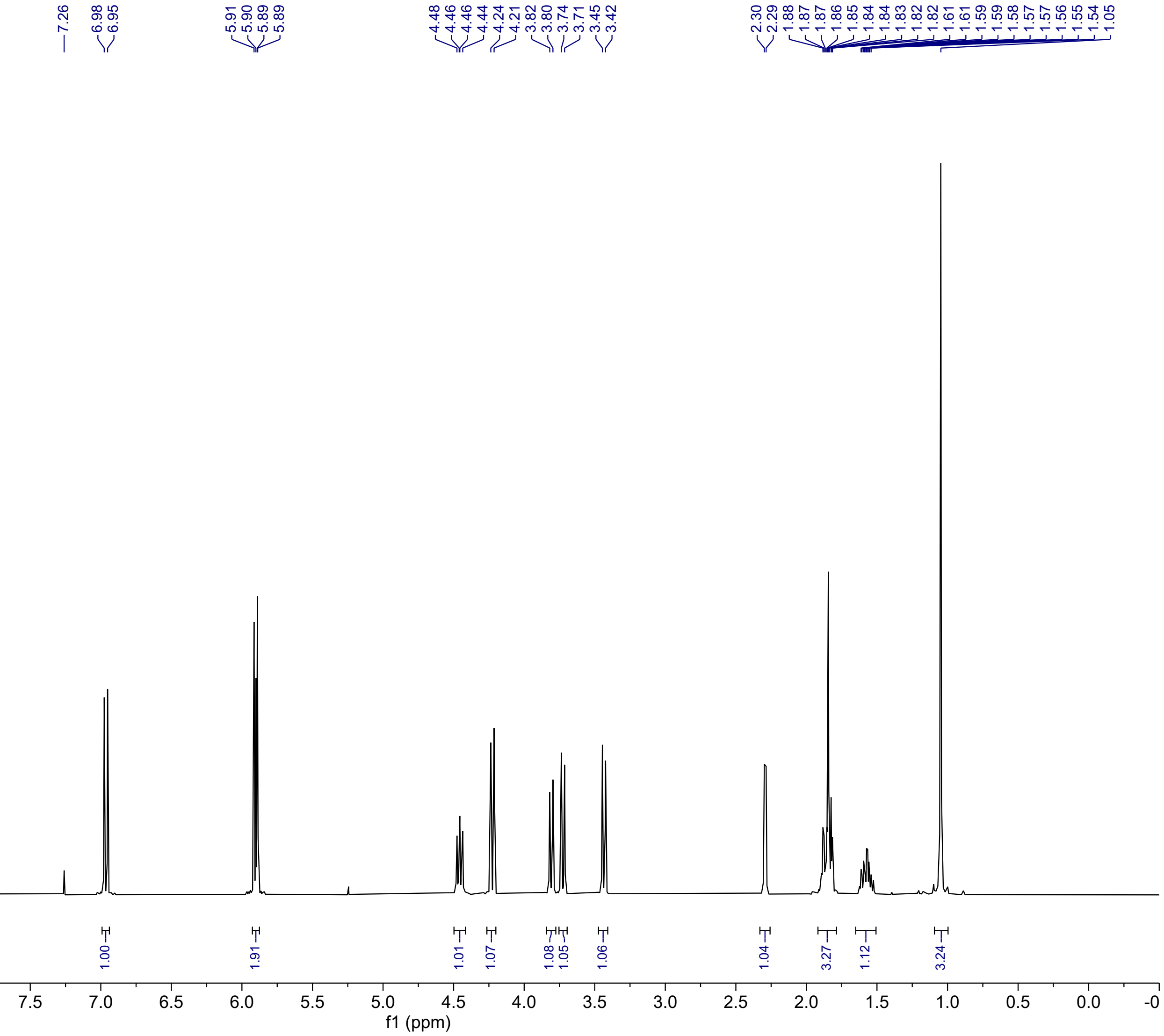

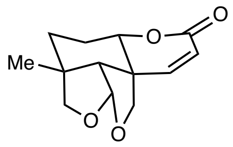

S53

^{13}C NMR (101 MHz, CDCl$_3$)

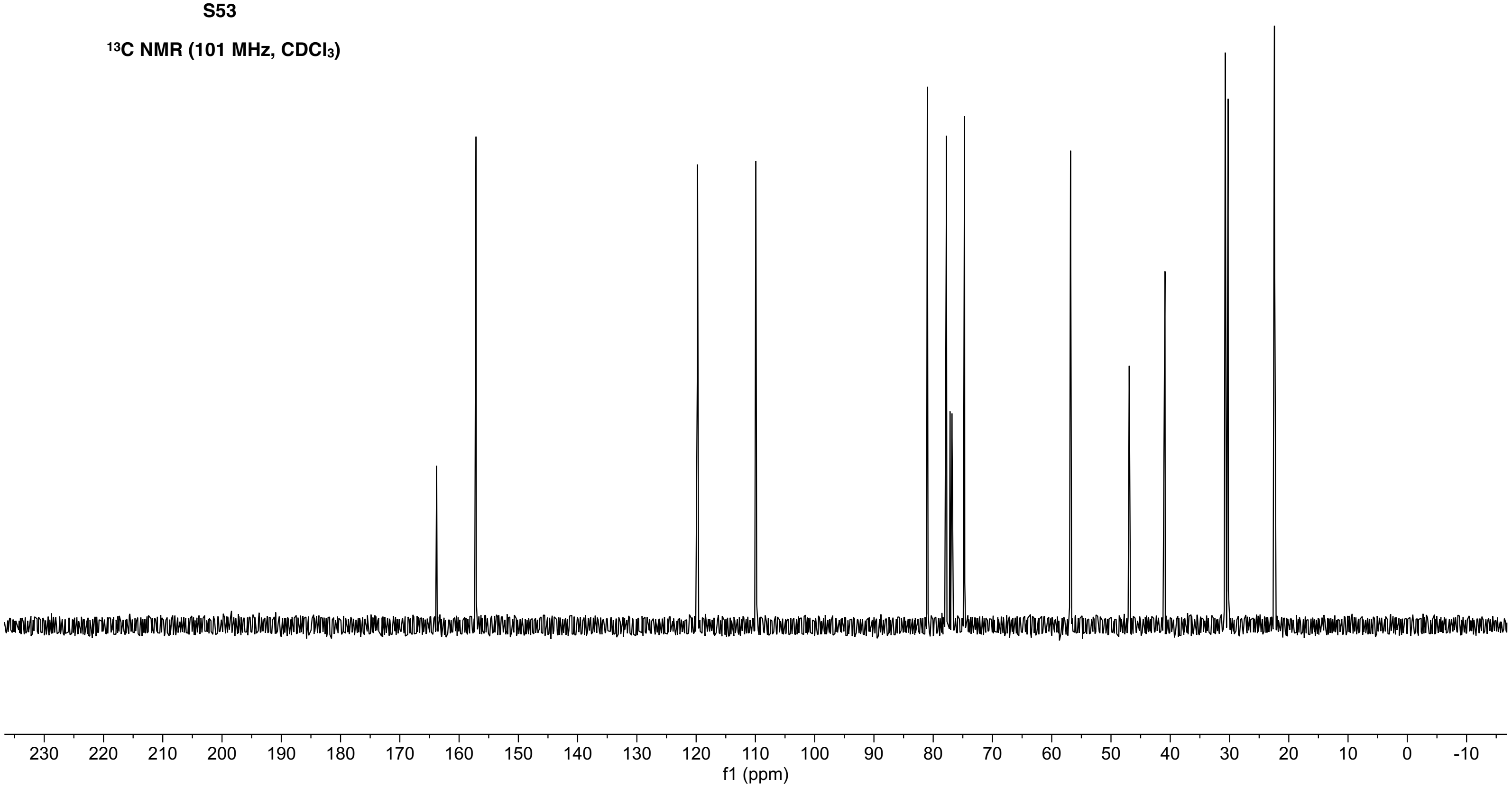

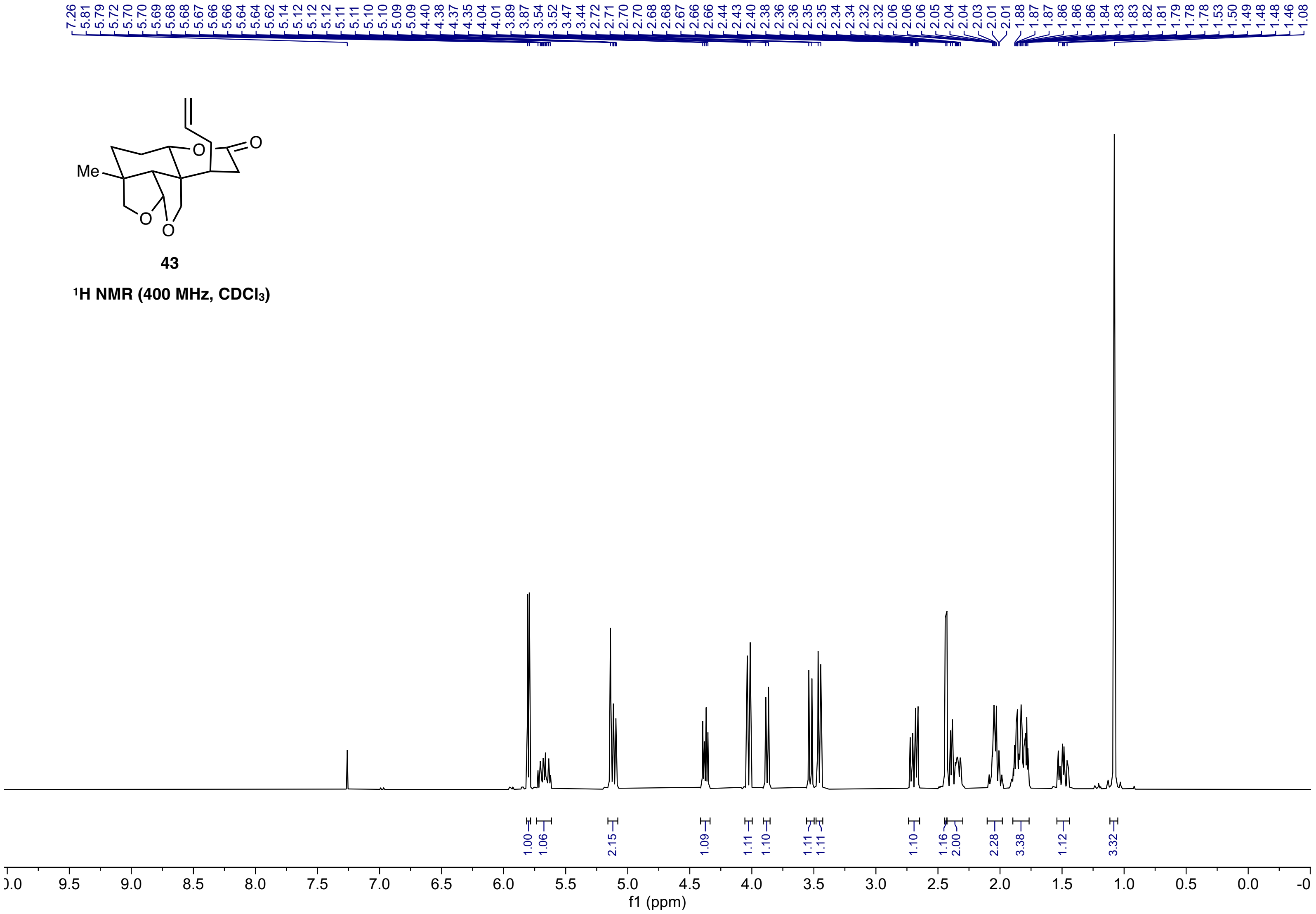

43

^{1}H NMR (400 MHz, CDCl$_3$)

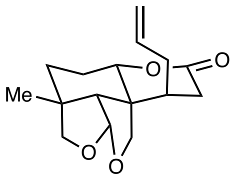

43

^{13}C NMR (101 MHz, CDCl$_3$)

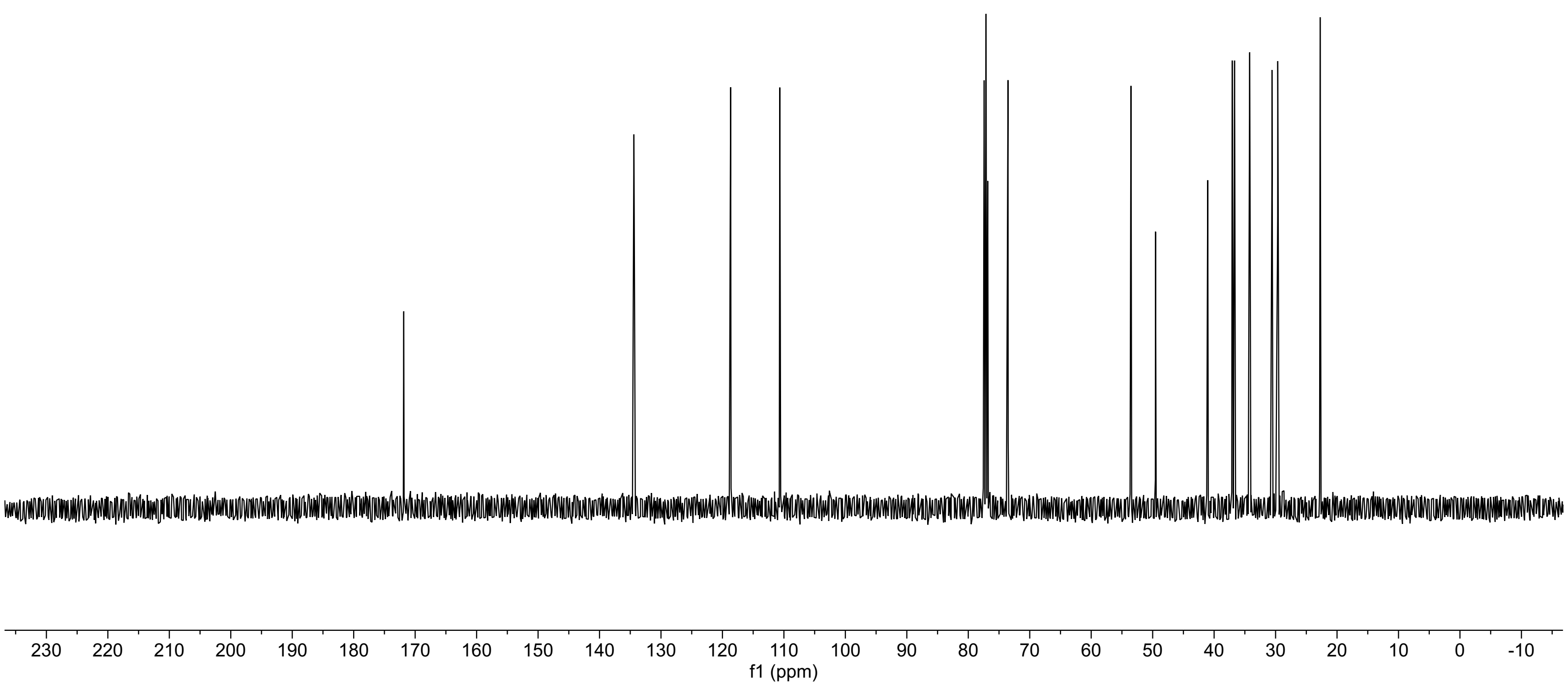

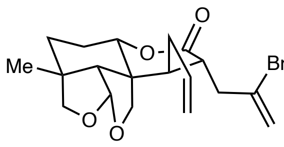

44

^{1}H NMR (400 MHz, CDCl$_3$)

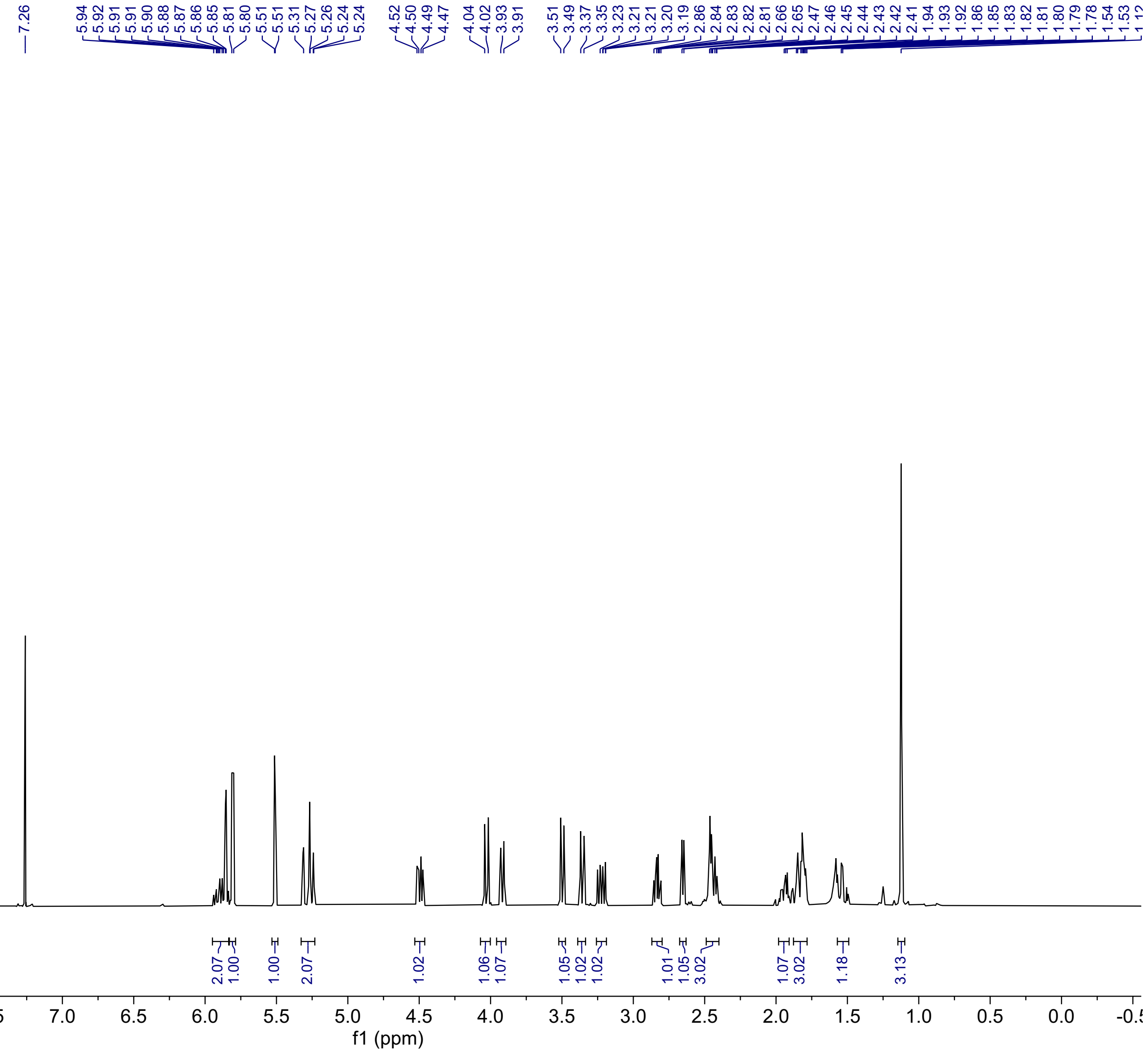

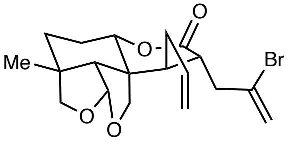

44

^{13}C NMR (101 MHz, CDCl$_3$)

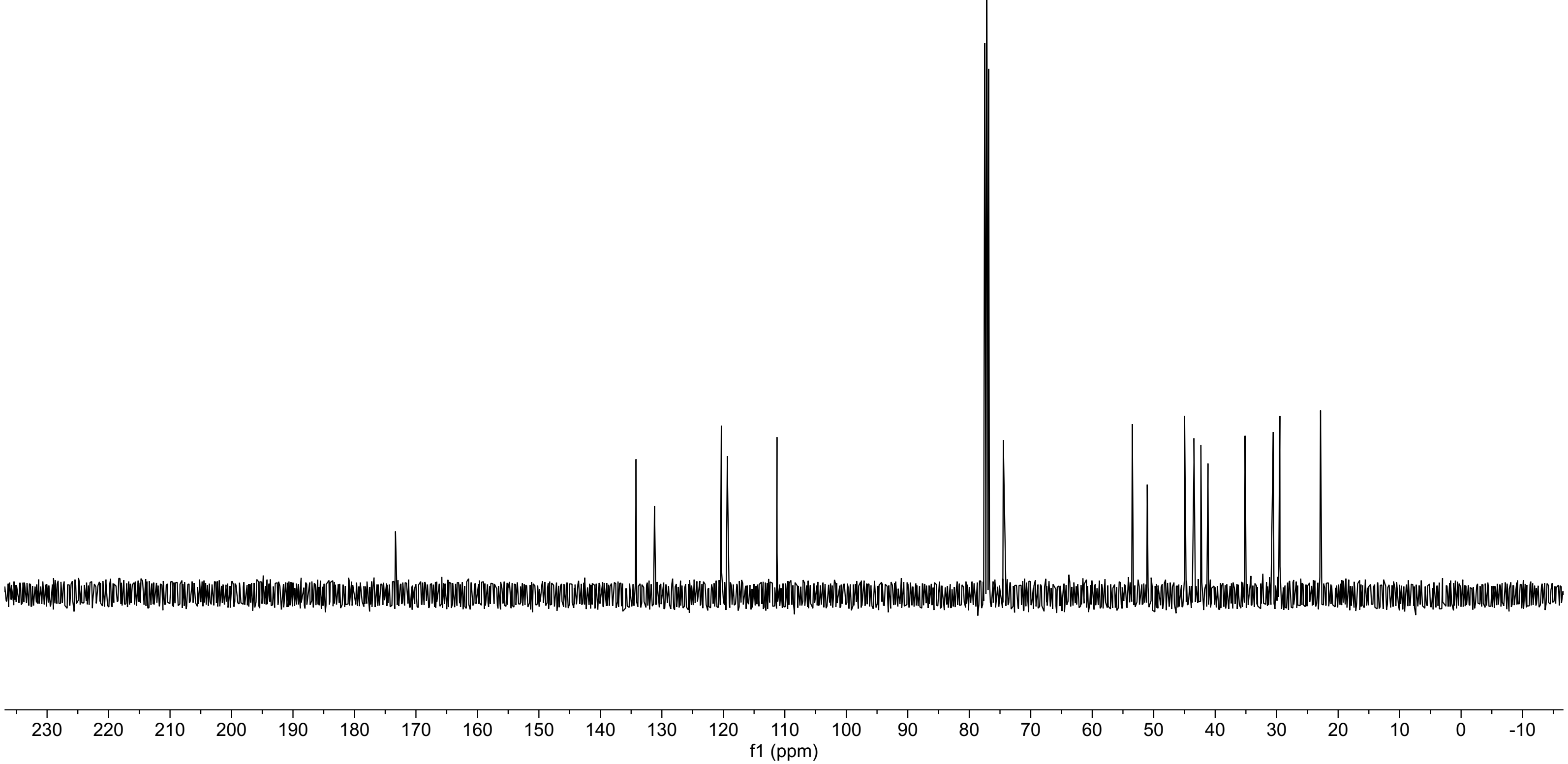

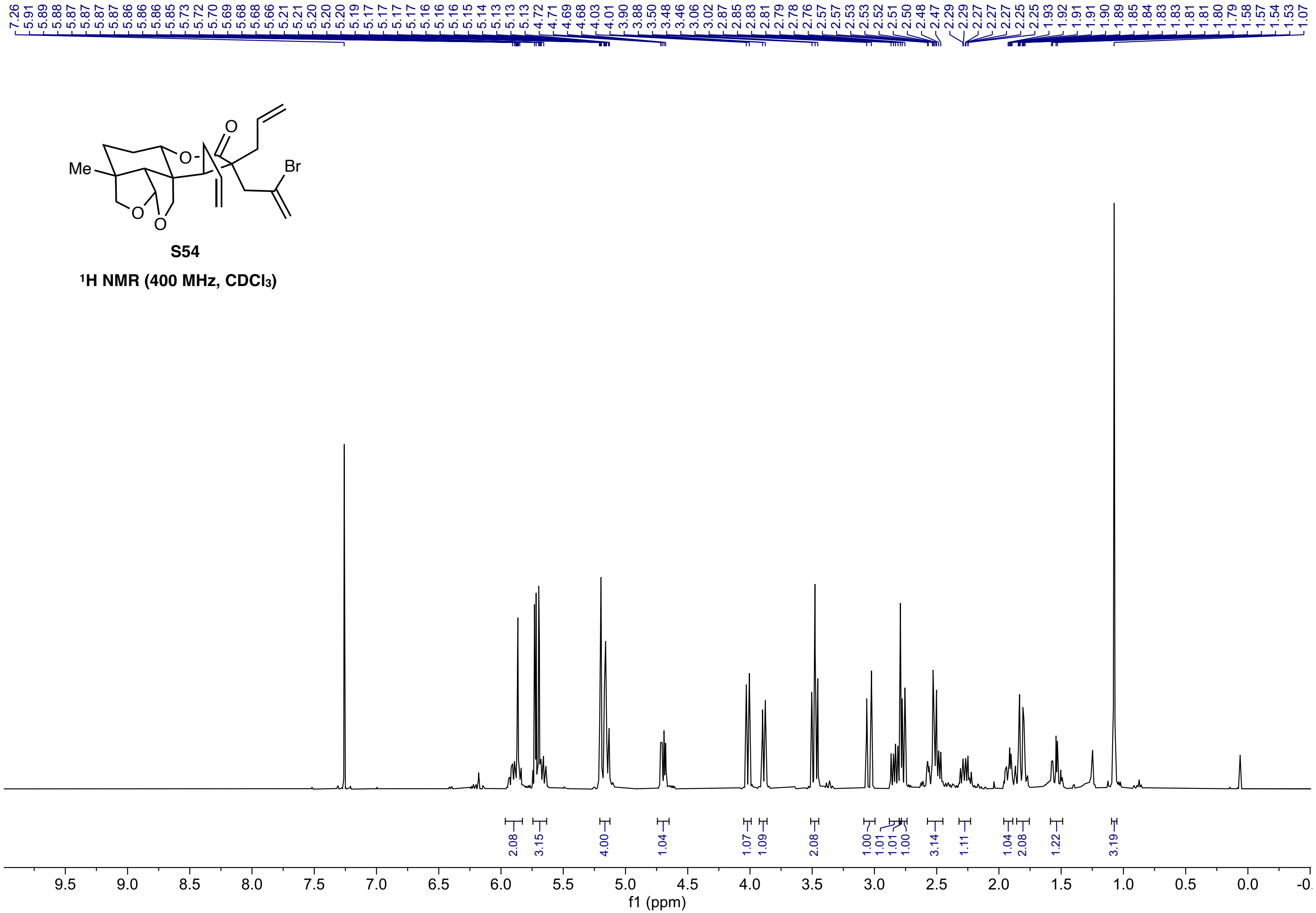
7.26 5.91 5.89 5.88 5.87 5.87 5.87 5.86 5.86 5.86 5.85 5.73 5.72 5.70 5.69 5.68 5.68 5.66 5.21 5.21 5.20 5.20 5.20 5.19 5.17 5.17 5.17 5.17 5.16 5.16 5.16 5.15 5.14 5.13 5.13 5.13 4.72 4.71 4.69 4.68 4.03 4.01 3.90 3.88 3.50 3.48 3.46 3.06 3.02 2.87 2.85 2.83 2.81 2.79 2.78 2.76 2.57 2.57 2.53 2.52 2.51 2.50 2.48 2.47 2.29 2.29 2.27 2.27 2.27 2.25 2.25 1.93 1.92 1.91 1.91 1.90 1.89 1.85 1.84 1.83 1.83 1.81 1.81 1.80 1.79 1.58 1.57 1.54 1.53 1.07
Me
O
Br
S54
1H NMR (400 MHz, CDCl3)
2.08 3.15 4.00 1.04 1.07 1.09 2.08 1.00 1.01 1.01 1.00 3.14 1.11 1.04 2.08 1.22 3.19
9.5 9.0 8.5 8.0 7.5 7.0 6.5 6.0 5.5 5.0 4.5 4.0 3.5 3.0 2.5 2.0 1.5 1.0 0.5 0.0
f1 (ppm)

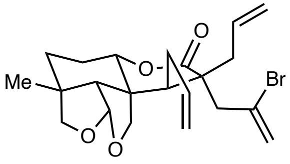

S54

^{13}C NMR (101 MHz, CDCl$_3$)

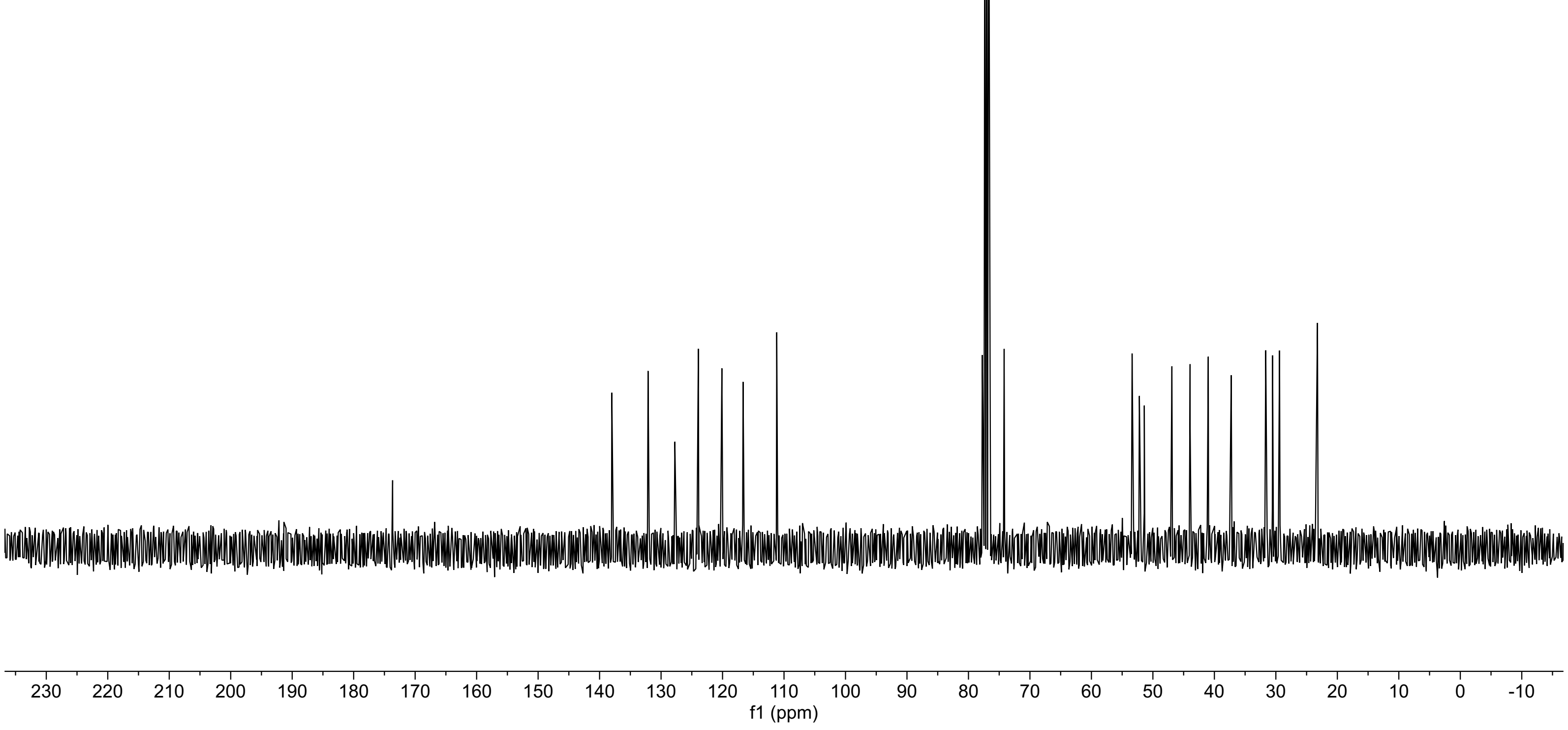

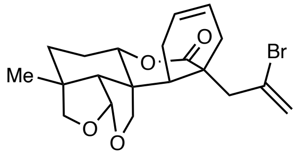

45

^{1}H NMR (400 MHz, CDCl$_3$)

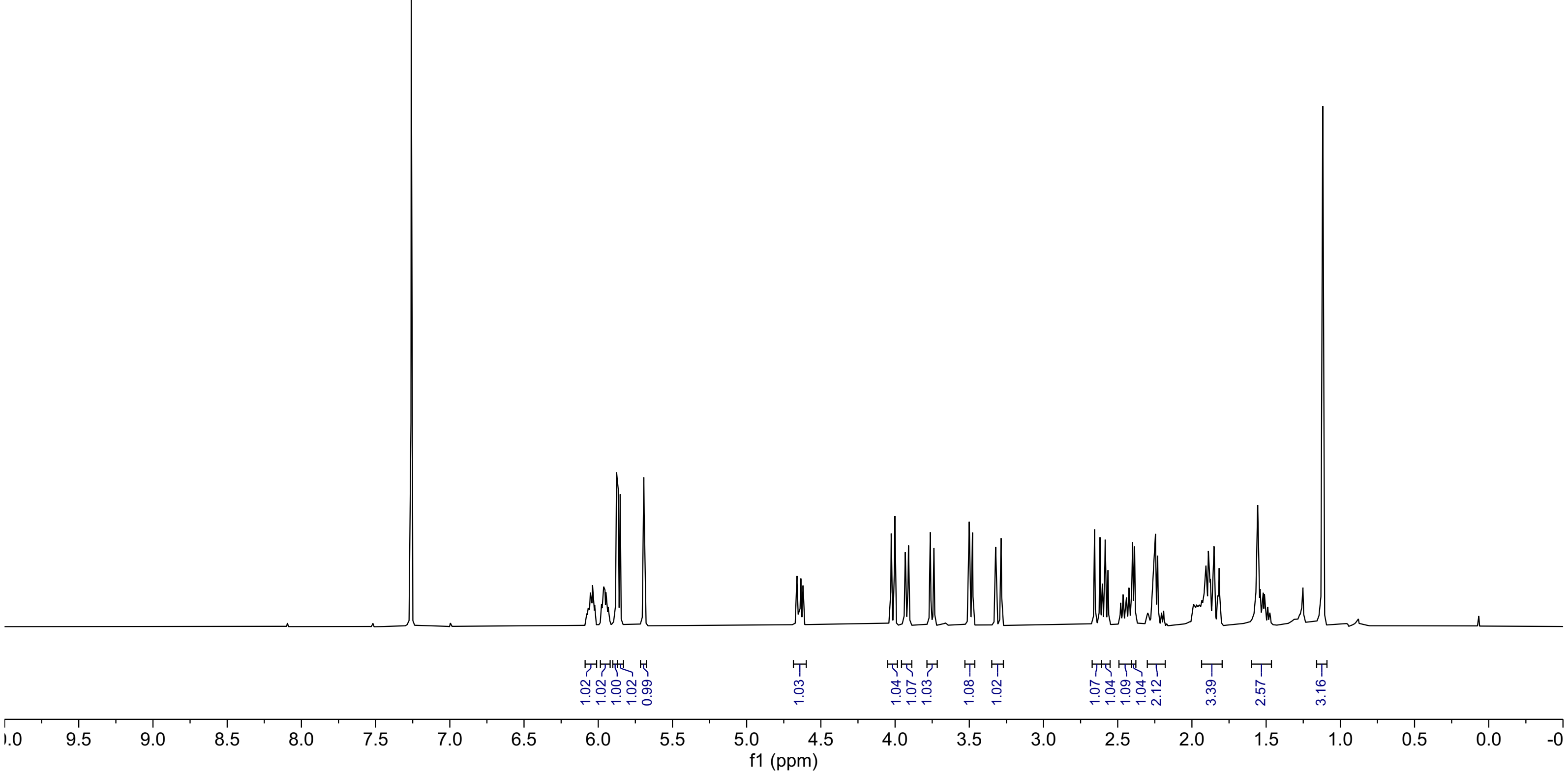

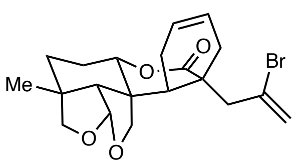

45

^{13}C NMR (101 MHz, CDCl$_3$)

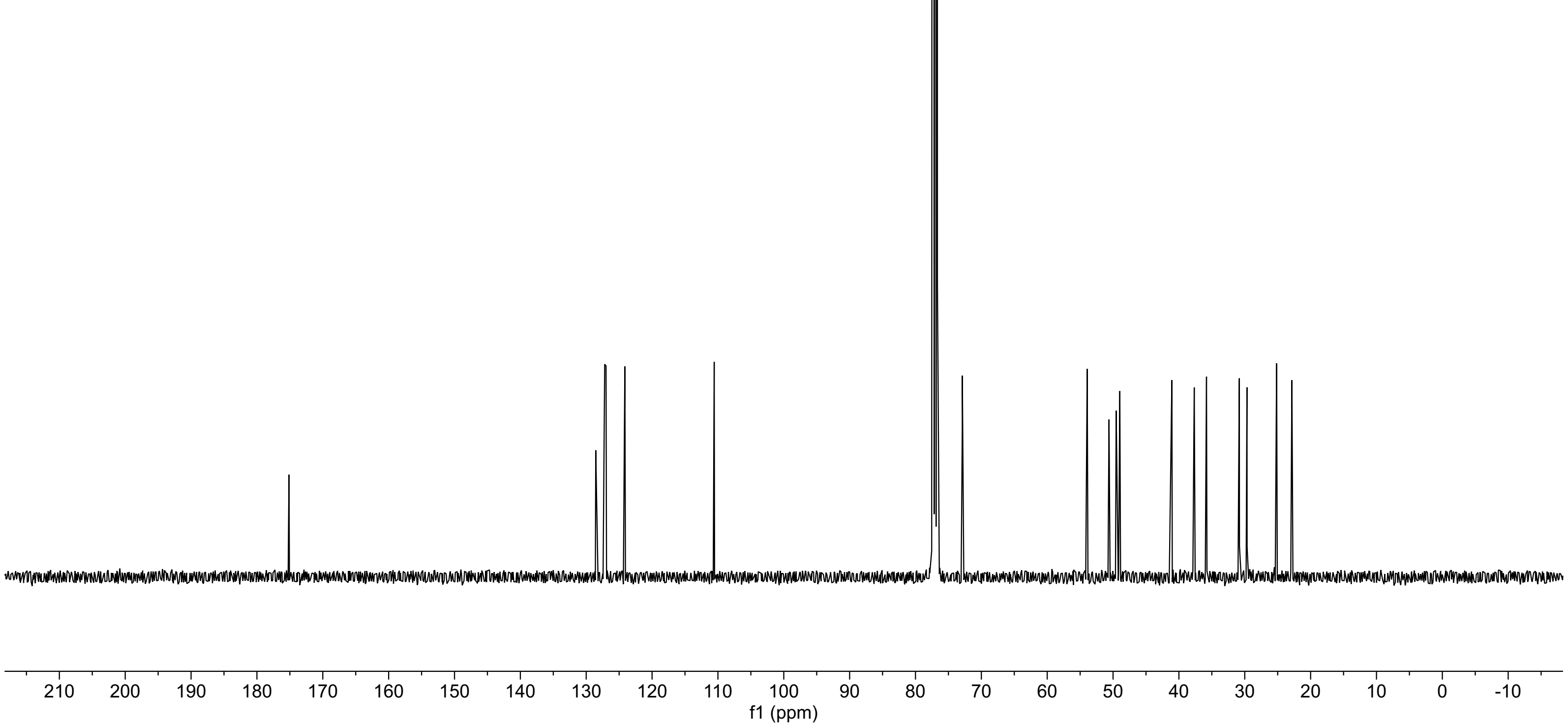

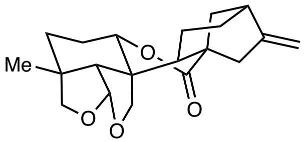

S55

^{1}H NMR (400 MHz, CDCl$_3$)

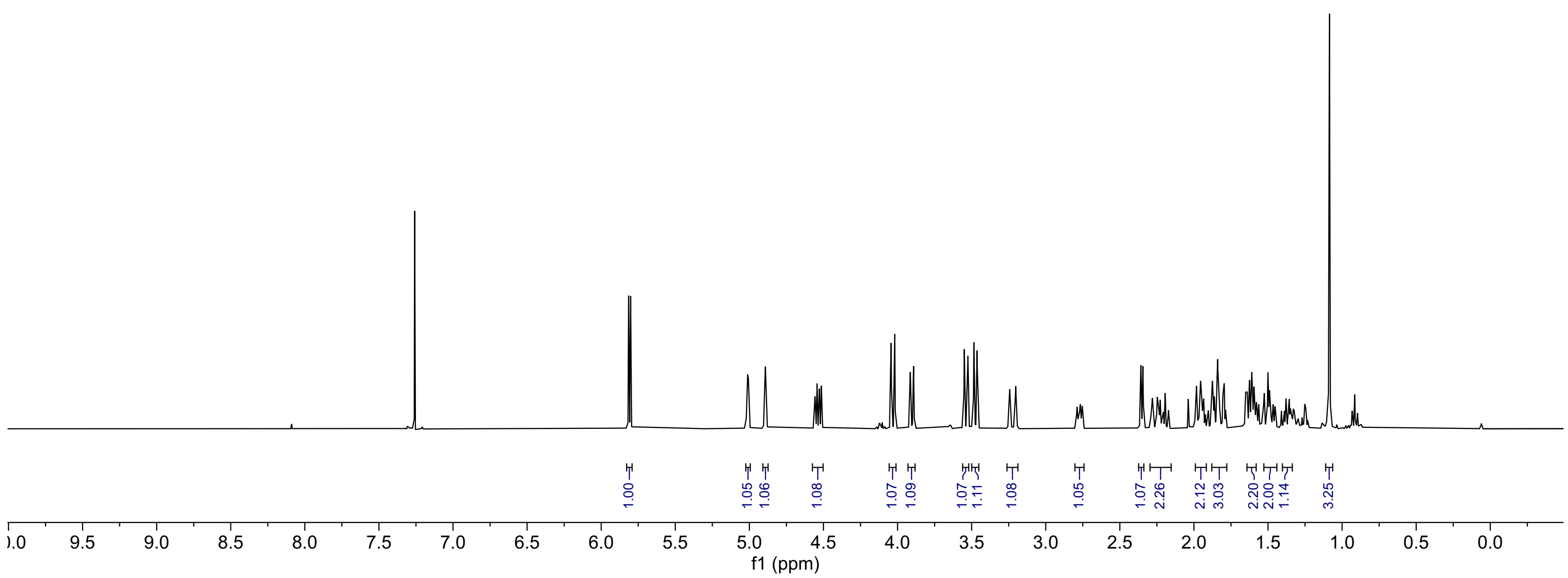

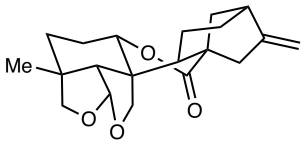

S55

^{13}C NMR (101 MHz, $CDCl_3$)

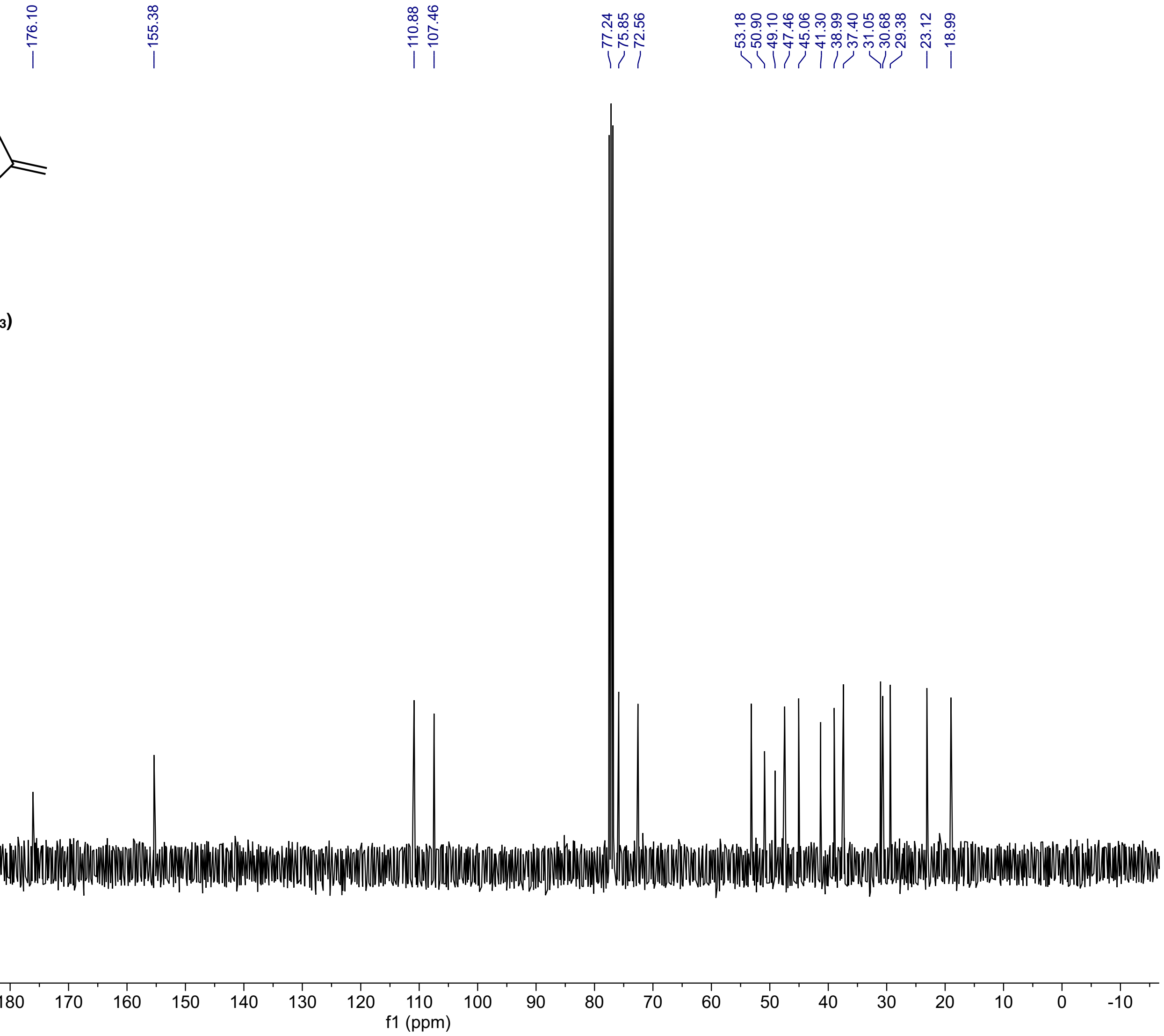

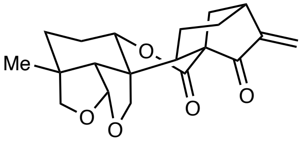

(–)-macrocalyxoformin B (**6**)

^{1}H NMR (400 MHz, $CDCl_3$)

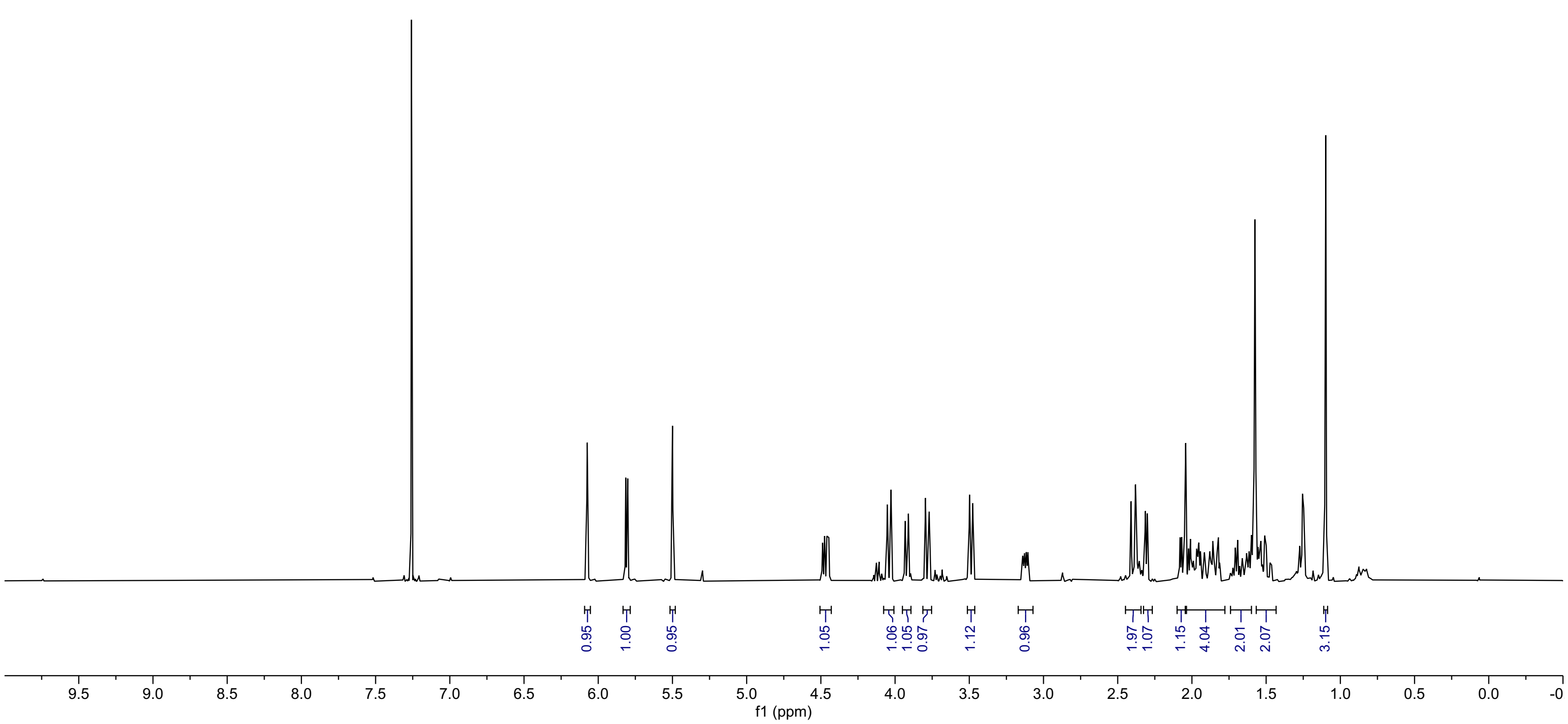

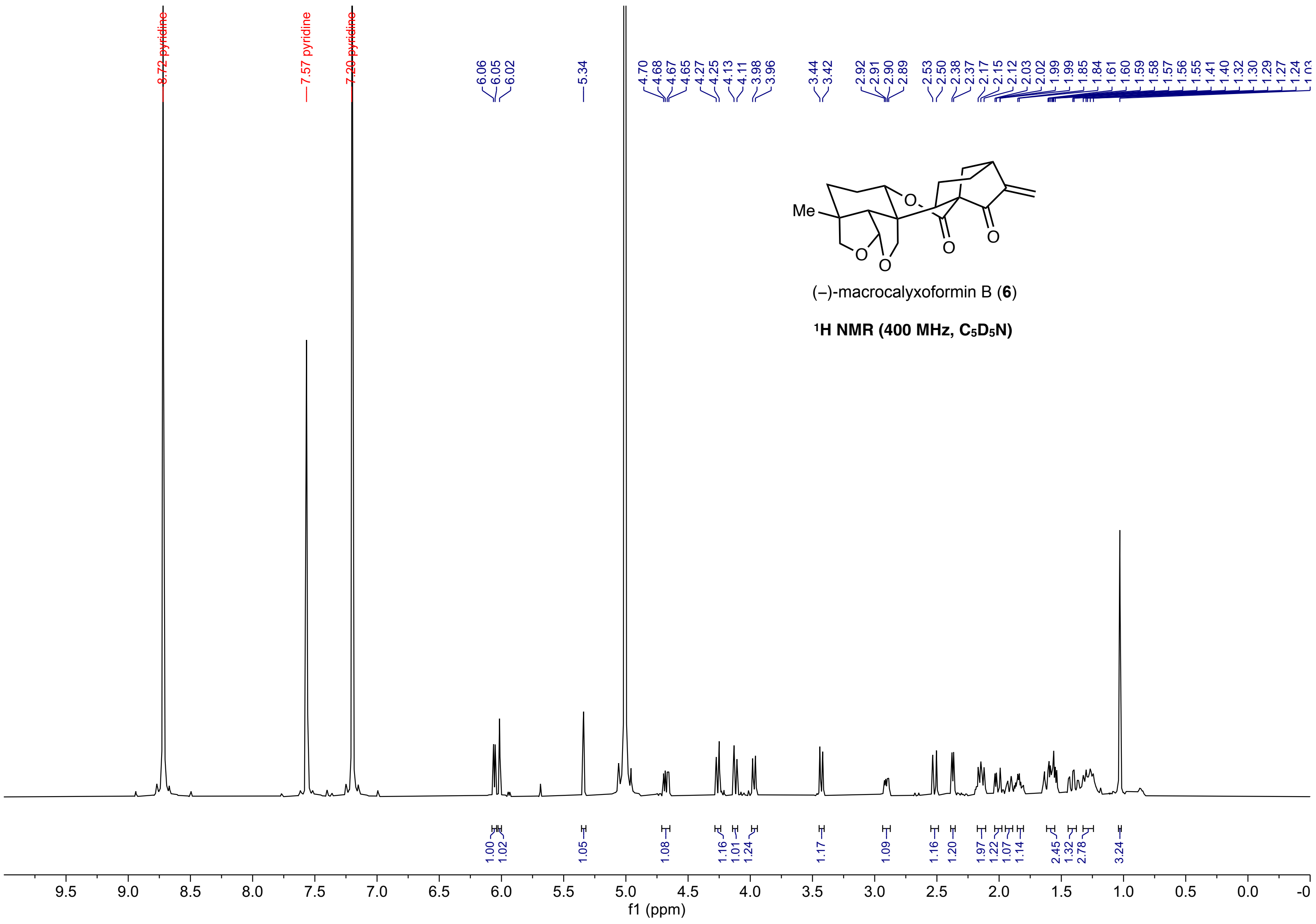
8.72 pyridine
7.57 pyridine
7.20 pyridine
Me
O
(–)-macrocalyxoformin B (**6**)
^{1}H NMR (400 MHz, C_5D_5N)
f1 (ppm)

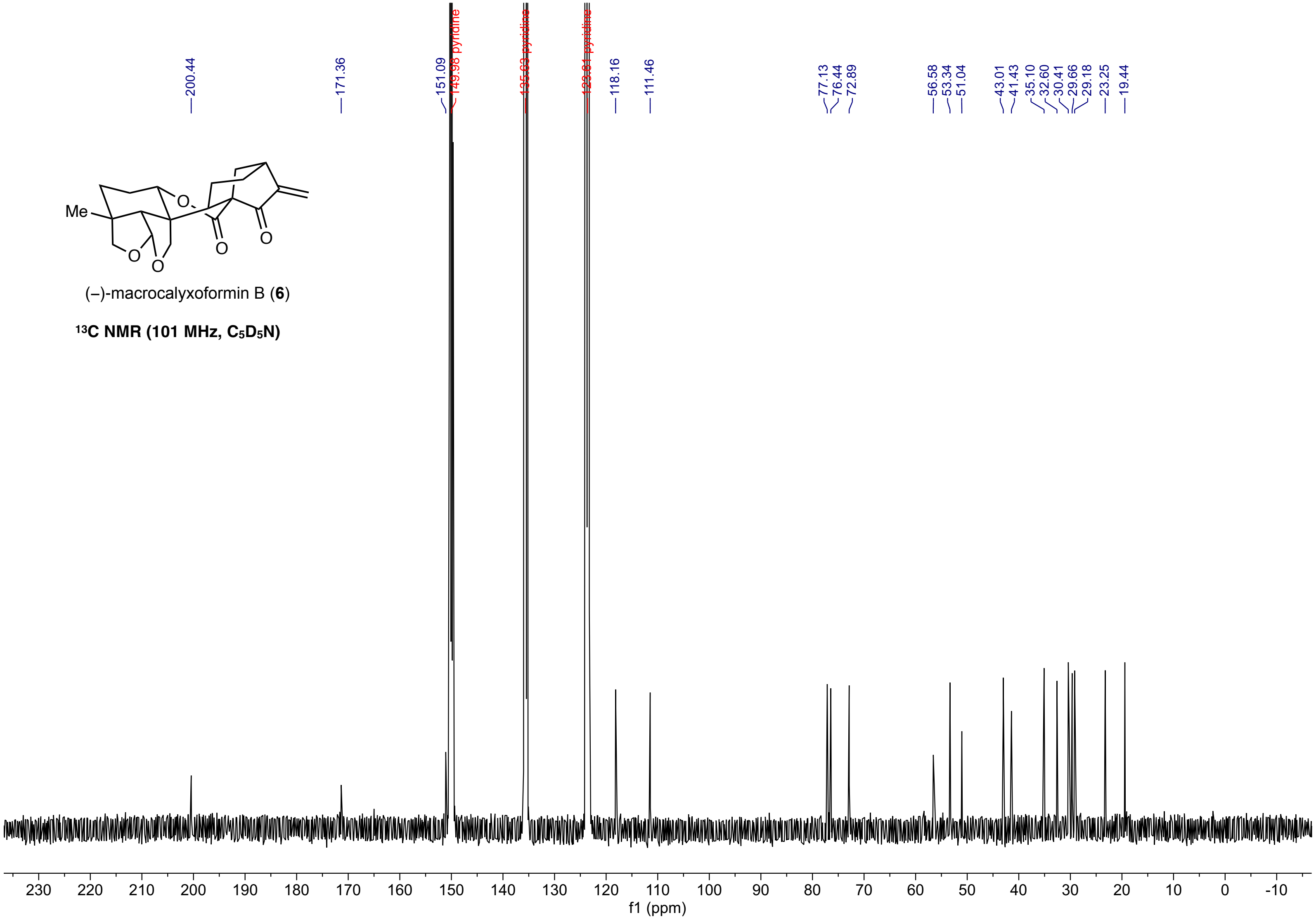
200.44
171.36
151.09
149.98 pyridine
135.63 pyridine
123.61 pyridine
118.16
111.46
77.13
76.44
72.89
56.58
53.34
51.04
43.01
41.43
35.10
32.60
30.41
29.66
29.18
23.25
19.44
Me
O
O
O
O
O
(–)-macrocalyxoformin B (6)
13C NMR (101 MHz, C5D5N)
230 220 210 200 190 180 170 160 150 140 130 120 110 100 90 80 70 60 50 40 30 20 10 0 -10
f1 (ppm)

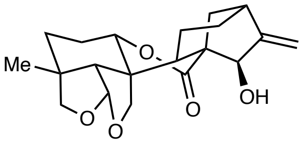

(–)-macrocalyxoformin A (**5**)

^{1}H NMR (400 MHz, CDCl$_3$)

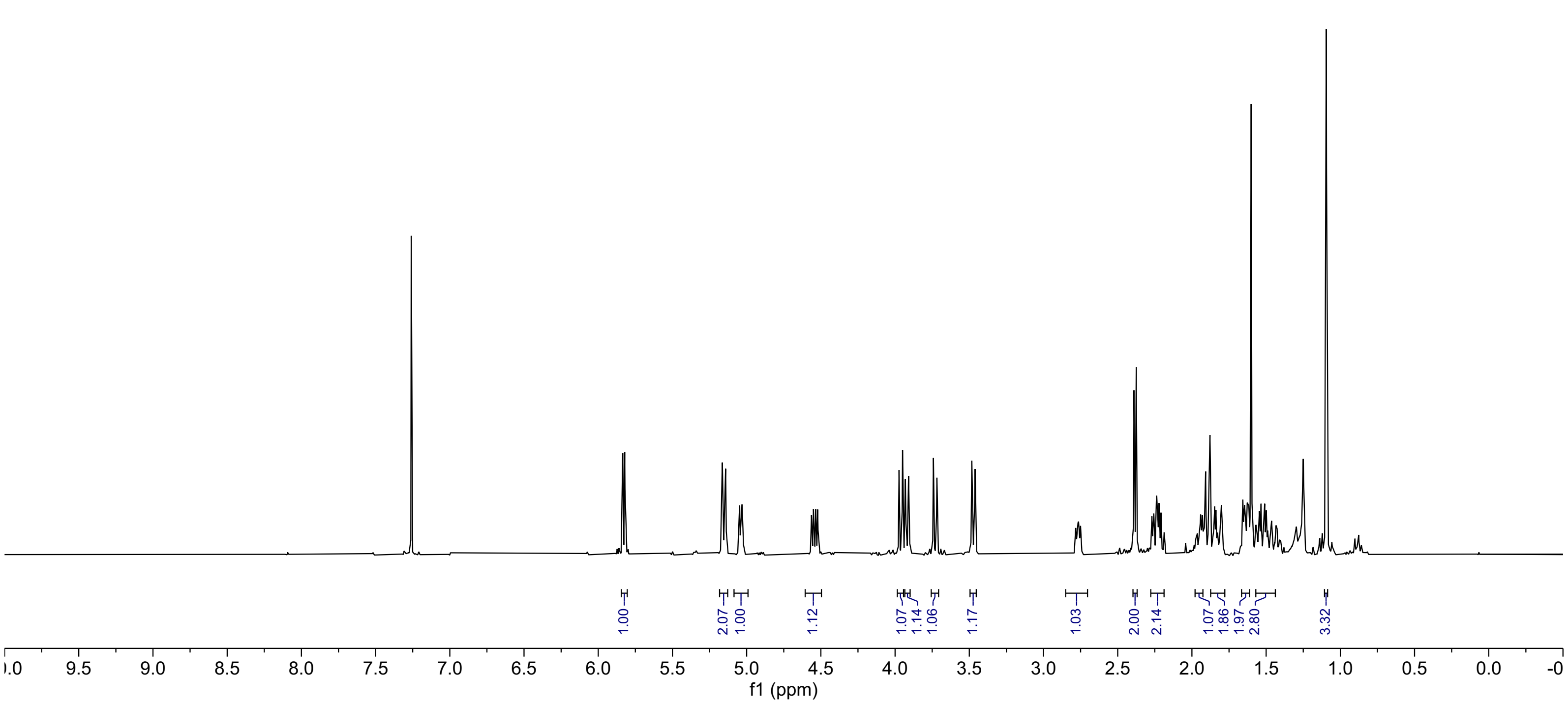

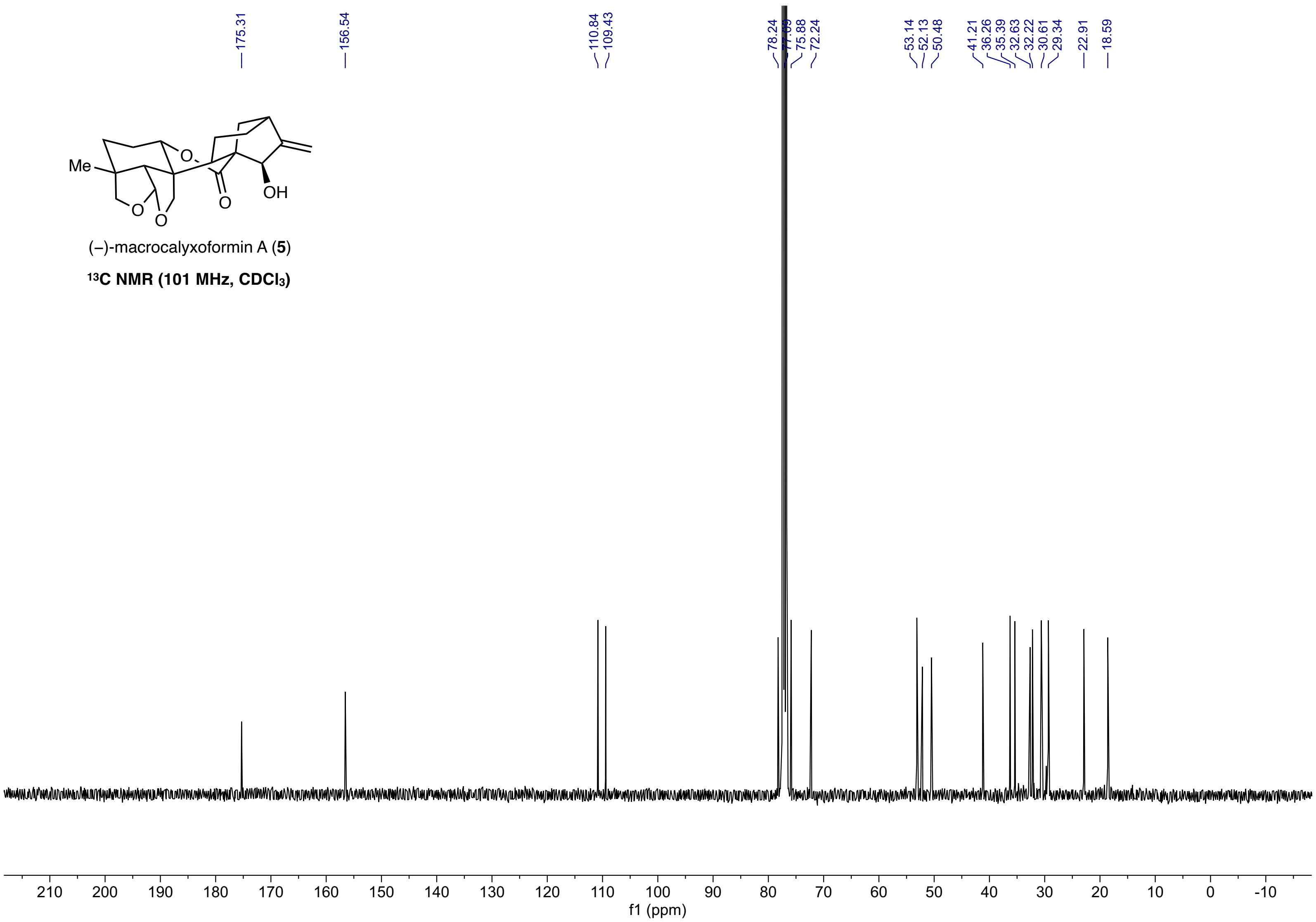
Me
O
O
O
O
OH
(–)-macrocalyxoformin A (5)
13C NMR (101 MHz, CDCl3)
175.31
156.54
110.84
109.43
78.24
75.88
72.24
53.14
52.13
50.48
41.21
36.26
35.39
32.63
32.22
30.61
29.34
22.91
18.59
210 200 190 180 170 160 150 140 130 120 110 100 90 80 70 60 50 40 30 20 10 0 -10
f1 (ppm)

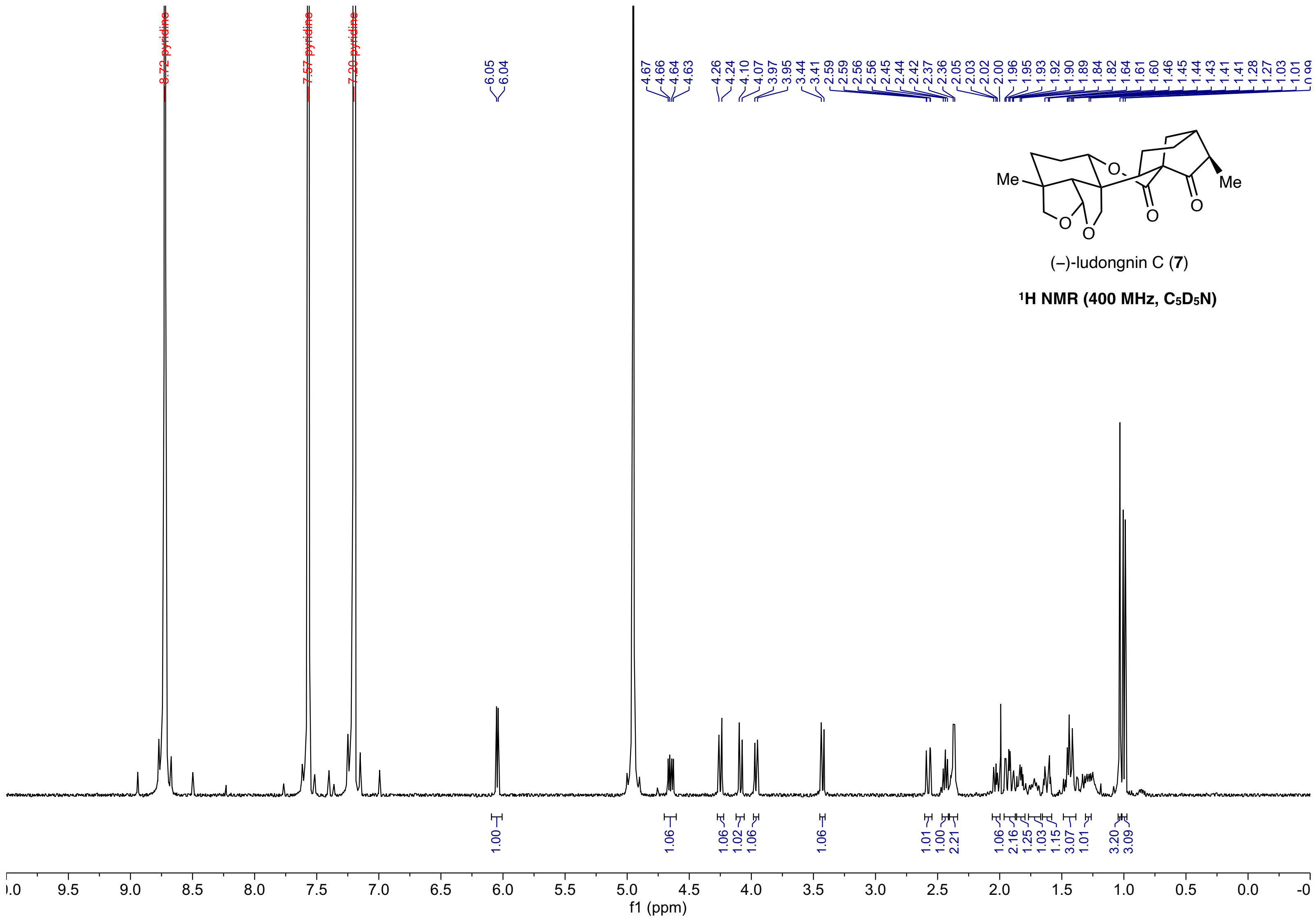
8.72 pyridine
7.57 pyridine
7.20 pyridine
Me
Me
O
O
O
O
O
(–)-ludongnin C (7)
¹H NMR (400 MHz, C₅D₅N)
f1 (ppm)

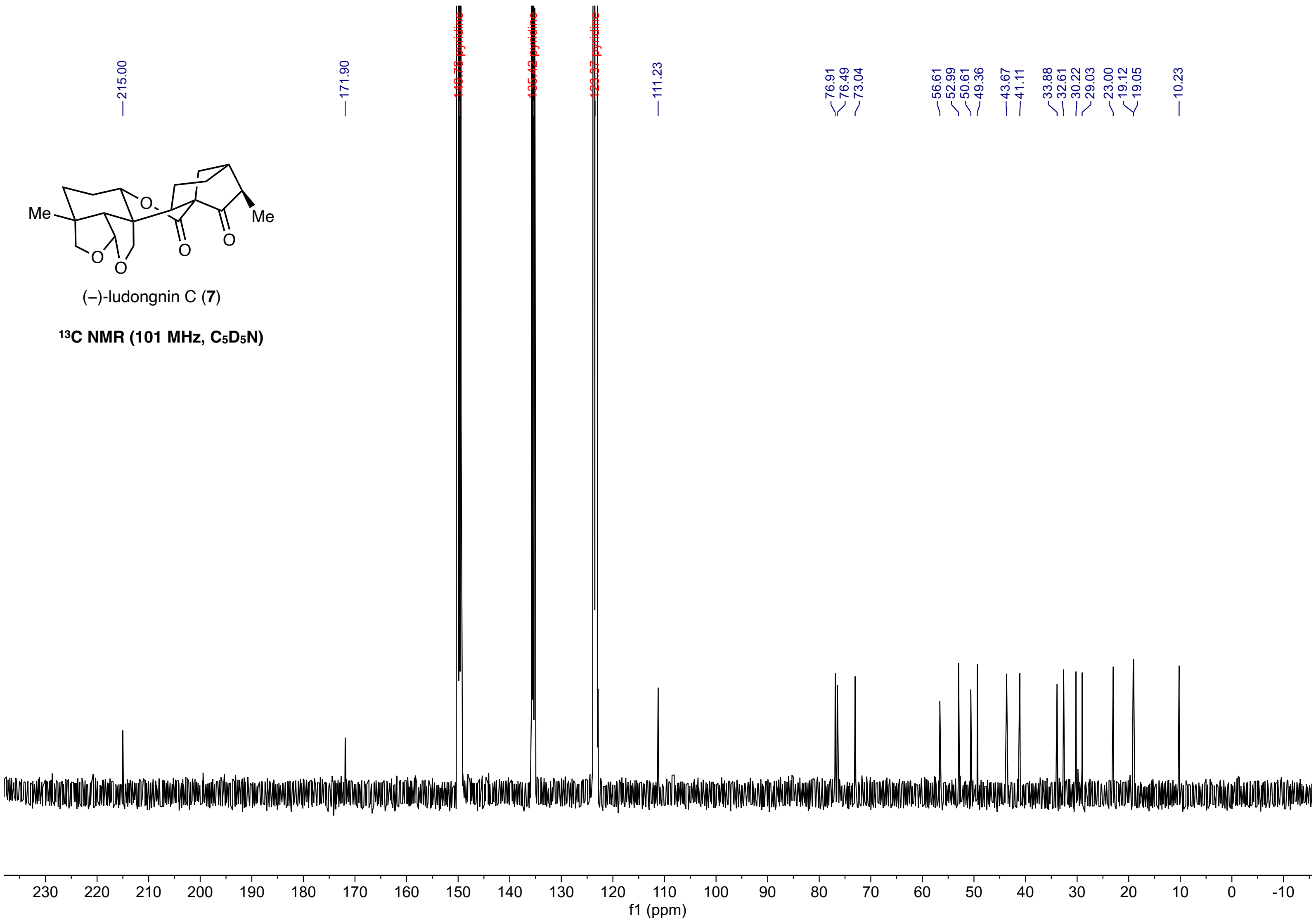

(–)-ludongnin C (**7**)

^{13}C NMR (101 MHz, C_5D_5N)

4. Supplementary References

1. Kozmin, S. A. & Rawal, V. H. Preparation and Diels-Alder reactivity of 1-amino-3-siloxy-1,3-butadienes. *J. Org. Chem.* **62**, 5252–5253 (1997).
2. Richter, M. J. R., Schneider, M., Brandstätter, M., Krautwald, S. & Carreira, E. M. Total synthesis of (–)-mitrephorone A. *J. Am. Chem. Soc.* **140**, 16704–16710 (2018).
3. Kurimoto, I. & Masayoshi, M. Process for preparing di-*tert*-butyl-ocarbonate. U. S. Patent 5151542, July 16 (1991).
4. Creemers, A. F. L. & Lugtenburg, J. The preparation of all-trans uniformly ^{13}C-labeled retinal via a modular total organic synthetic strategy. Emerging central contribution of organic synthesis toward the structure and function study with atomic resolution in protein research. *J. Am. Chem. Soc.* **124**, 6324–6334 (2002).
5. Yamamoto, Y., Shirai, T. & Miyaura, N. Asymmetric addition of arylboronic acids to glyoxylate catalyzed by a ruthenium/Me-BIPAM complex. *Chem. Commun.* **48**, 2803–2805 (2012).
6. Rawal, V. H., Huang, Y., Unni A. K. & Thadani, A. N. Methods of performing cycloadditions, reaction mixtures, and methods of performing asymmetric catalytic reactions. U. S. Patent 7230125, June 12 (2007).
7. Nicolaou, K. C., Tria, G. S., Edmonds, D. J. & Kar, M. Total syntheses of (±)-platencin and (−)-platencin. *J. Am. Chem. Soc.* **131**, 15909–15917 (2009).
8. Yuan, Y., Zhang, X., Zhou, D. & Zheng, G. Discovery of a novel BCL-X_L PROTAC degrader with enhanced BCL-2 inhibition. *J. Med. Chem.* **64**, 14230–14246 (2021).
9. Li, W. et al. New monocyclic, bicyclic, and tricyclic ethynylcyanodienones as activators of the Keap1/Nrf2/ARE pathway and inhibitors of inducible nitric oxide synthase. *J. Med. Chem.* **58**, 4738–4748 (2015).
10. Roush, W. R., Adam, M. A., Walts, A. E. & Harris, D. J. Stereochemistry of the reactions of substituted allylboronates with chiral aldehydes. Factors influencing aldehyde diastereofacial selectivity. *J. Am. Chem. Soc.* **108**, 3422–3434 (1986).
11. Karnekanti, R., Hanumaiah, M. & Sharma, G. V. M. Stereoselective total synthesis of (+)-anamarine and 8-*epi*-(–)-anamarine from D-mannitol. *Synthesis* **47**, 2997–3008 (2015).
12. Chu, L., Ohta, C., Zuo, Z. & MacMillan, D. W. C. Carboxylic acids as a traceless activation group for conjugate additions: A three-step synthesis of (±)-pregabalin. *J. Am. Chem. Soc.* **136**, 10886–10889 (2014).
13. Minagawa, K., Kamakura, D., Hagiwara, K. & Inoue, M. Construction of the ABCE-ring structure of talatisamine via decarboxylative radical cyclization. *Tetrahedron* **76**, 131385 (2020).
14. Anderson, J. M. & Kochi, J. K. Silver(II) complexes in oxidative decarboxylation of acids. *J. Org. Chem.* **35**, 986–989 (1970).
15. Ramirez, N. P. & Gonzalez-Gomez, J. C. Decarboxylative Giese-type reaction of carboxylic acids promoted by visible light: A sustainable and photoredox-neutral protocol. *Eur. J. Org. Chem.* 2154−2163 (2017).
16. Qin, T. et al. Nickel-catalyzed Barton decarboxylation and Giese Reactions: A practical take on classic transforms. *Angew Chem. Int. Ed. Engl.* **56**, 260–265 (2017).

17. Pratsch, G., Lackner, G. L. & Overman, L. E. Constructing quaternary carbons from *N*-(acyloxy)phthalimide precursors of tertiary radicals using visible-light photocatalysis. *J. Org. Chem.* **80**, 6025–6036 (2015).
18. Pitre, S. P., Allred, T. K. & Overman, L. E. Lewis acid activation of fragment-coupling reactions of tertiary carbon radicals promoted by visible-light irradiation of EDA complexes. *Org. Lett.* **23**, 1103–1106 (2021).
19. Fu, M., Shang, R., Zhao, B., Wang, B. & Fu, Y. Photocatalytic decarboxylative alkylations mediated by triphenylphosphine and sodium iodide. *Science* **363**, 1429-1434 (2019).
20. Yoshida, M. Asymmetric α-allylation of α-substituted β-ketoesters with allyl alcohols. *J. Org. Chem.* **82**, 12821–12826 (2017).
21. Hu, Y. et al. Synthesis of novel *ent*-kaurane-type diterpenoid derivatives effective for highly aggressive tumor cells. *Molecules* **23**, 3216 (2018).
22. Wang, Z., Wang, X., Dong, J. & Xue, Z. Chemical structures of macrocalyxoformin B, C and E. *J. Integr. Plant Biol*. **28**, 79–85 (1986).
23. Wang, Z., Wang, X. & Dong, G. New antibiotic diterpenoid from *Rabdosia macrocalyx* (Dunn) Hara–macrocalyxoformin A. *Chinese Traditional and Herbal Drugs* **14**, 481–485 (1983).
24. Han, Q., Li, S., Peng, L. & Sun, H. *Ent*-kaurane diterpenoids from *Isodon rubescens* var. *lushiennsis*. *Heterocycles* **60**, 933–938 (2003).
